# Combining Organometallic Reagents, the Sulfur Dioxide Surrogate DABSO, and Amines: A One-Pot Preparation of Sulfonamides, Amenable to Array Synthesis**

**DOI:** 10.1002/anie.201409283

**Published:** 2014-11-27

**Authors:** Alex S Deeming, Claire J Russell, Michael C Willis

**Affiliations:** Department of Chemistry, University of Oxford, Chemistry Research LaboratoryMansfield Road, Oxford, OX1 3TA (UK); Syngenta, Jealott's Hill International Research CentreBracknell, Berkshire, RG42 6EY (UK)

**Keywords:** amines, array synthesis, organometallics, physiochemical properties, sulfonamides

## Abstract

We describe a method for the synthesis of sulfonamides through the combination of an organometallic reagent, a sulfur dioxide equivalent, and an aqueous solution of an amine under oxidative conditions (bleach). This simple reaction protocol avoids the need to employ sulfonyl chloride substrates, thus removing the limitation imposed by the commercial availability of these reagents. The resultant method allows access to new chemical space, and is also tolerant of the polar functional groups needed to impart favorable physiochemical properties required for medicinal chemistry and agrochemistry. The developed chemistry is employed in the synthesis of a targeted 70 compound array, prepared using automated methods. The array achieved a 93 % success rate for compounds prepared. Calculated molecular weights, lipophilicities, and polar surface areas are presented, demonstrating the utility of the method for delivering sulfonamides with drug-like properties.

The defining features of sulfonamides—heteroatom-rich composition, balanced lipophilicity, good chemical and metabolic stability, H-bond donor and acceptor ability, and three-dimensional structure—combine to make them one of the pre-eminent functional groups found in designed medicinal and agrochemical agents. Accordingly, sulfonamides are present in molecules active against a diverse range of biological effects (Figure 1 a).[1] Despite the prevalence of sulfonamides, methods for their synthesis are largely confined to the combination of amines with isolated, pre-activated sulfonyl derivatives, most usually sulfonyl chlorides.[2] This is a highly effective way to prepare sulfonamides, but it is not without limitations. Importantly, the chemical space accessible using this approach is defined by the availability of the corresponding sulfonyl chlorides. These, in turn, are limited by the largely classical methods used for their synthesis (Figure 1 b).[3] Alkyl, alkenyl, and heteroaryl sulfonyl chlorides have a narrow commercial availability, reflecting both the methods available for their synthesis and the stability of these reagents. It follows that an effective way to prepare sulfonamides that occupy new areas of chemical space would be to devise a synthetic route that avoids the use of sulfonyl chlorides, and instead employed starting materials of greater diversity. If the focus is on providing sulfonamides applicable as bioactive molecules, then in addition to delivering novel structures—new chemical space[4]—the method must also allow the preparation of molecules with the physiochemical profiles that have been shown to correlate with increased success in achieving drug- and agrochemical-like properties.[5, 6] An additional desirable feature for new methods focused on delivering bioactive molecules is the compatibility of the method for array, or library synthesis.[7, 8] In the following Communication we describe the development of such a process, based on the combination of organometallic reagents, a sulfur dioxide surrogate, and amines (Figure 1 c).

**Figure 1:**
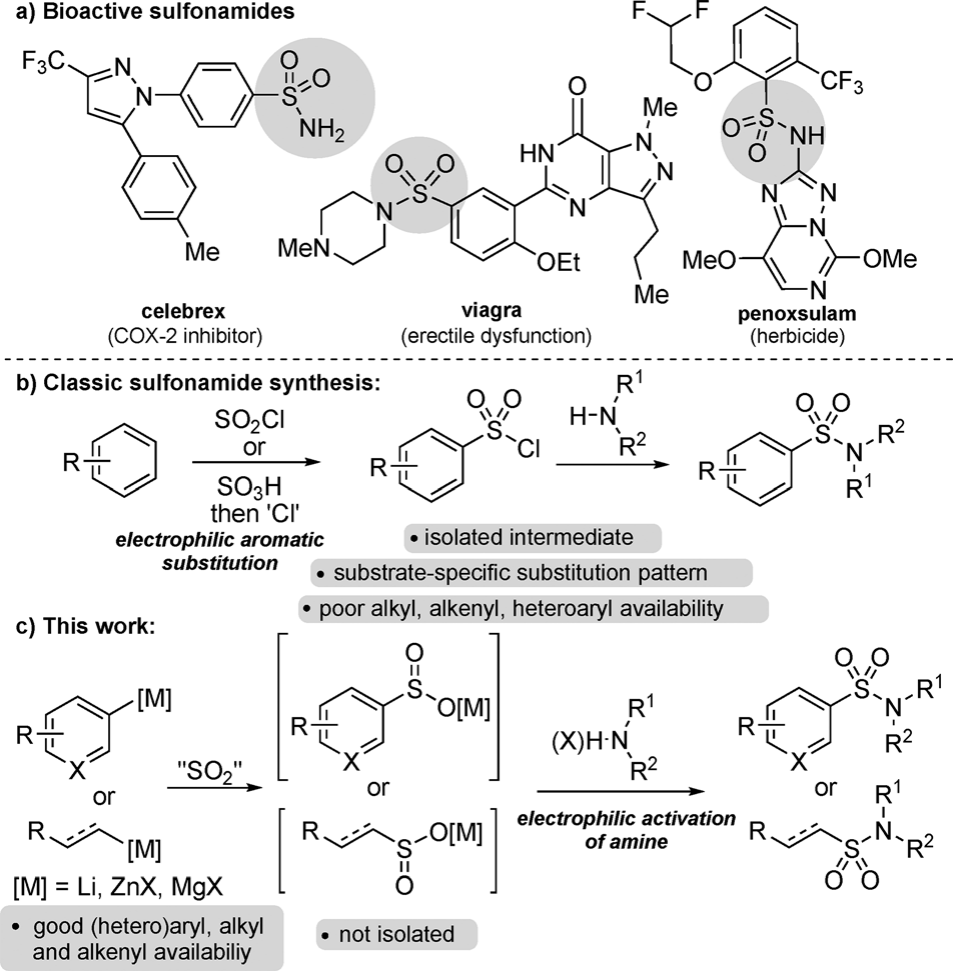
a) Selected bioactive sulfonamides. b) Classic sulfonamide synthesis based on an initial electrophilic aromatic substitution reaction to prepare a sulfonyl chloride. c) Present work: Sulfonamide synthesis based on the combination of an in situ-generated sulfinate intermediate and an electrophilically activated amine.

To develop a sulfonamide synthesis that avoids the use of sulfonyl chlorides, as either substrates or formal intermediates, we were attracted to sulfinates as suitable precursors. Although the commercial availability of sulfinates is poor, they are readily generated in situ from the combination of an organometallic reagent and sulfur dioxide gas,[9] or more attractively, a sulfur dioxide surrogate.[10] Barrett and co-workers reported a sulfonamide synthesis based on the combination of Grignard reagents and sulfur dioxide gas, followed by treatment with sulfuryl chloride and an amine.[11] Our laboratory subsequently reported a related process, in which we were able to replace the gaseous reagent with a sulfur dioxide surrogate, the bis-SO_2_ adduct of 1,4-diazabicyclo[2.2.2]octane (DABCO), DABSO.[12, 13] Although this was effective for simple substrates, it was hampered by the need to employ sulfuryl chloride, a reagent that requires frequent purification and has limited functional group compatibility. To deliver a robust, user-friendly process that would be tolerant against functional groups, led us to consider alternative strategies based on the nucleophilic character of the in situ-generated sulfinates. We had recently shown that such sulfinates could be combined with a broad range of carbon-based electrophiles to achieve an efficient sulfone synthesis,[10] and were intrigued as to whether we could replace the C-electrophiles with a range of N-based electrophiles and therefore access sulfonamides (Figure 1 c).

*N*-Chloroamines have gained popularity as an effective source of electrophilic amine fragments, and have been used in metal- and non-metal-catalyzed coupling reactions.[14] Although some precedent exists for the combination of isolated sodium sulfinates and *N*-chloroamines,[15] we were interested to explore their reactivity with in situ-generated sulfinates. Pleasingly, we found that magnesium sulfinates formed in situ could be successfully coupled with *N*-chloromorpholine, delivering the corresponding sulfonamides **1 a**,**b** in high yields [Eq. (1), Scheme 1]. Although the inherent reactivity achieved in these reactions was encouraging, the use of isolated *N*-chloroamines as electrophilic partners was not ideal, due to the need for each chloroamine to be individually prepared, and also the handling difficulties associated with these molecules. Accordingly, we were attracted to the possibility of generating both a metal sulfinate and a *N*-chloroamine in situ. Such a process is unprecedented, and would allow simple organometallic reagents and simple amines to be the immediate precursors that are combined (along with an SO_2_ surrogate) for the proposed sulfonamide synthesis. To explore this possibility we began by studying the combination of 3-methoxyphenylmagnesium bromide, morpholine, and an aqueous solution of sodium hypochlorite (bleach). After some experimentation[16] we identified reaction conditions that allowed the target sulfonamide **2** to be isolated in good yield [Eq. (2), Scheme 1].

**Scheme 1:**
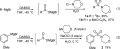
The combination of in situ-generated magnesium sulfinates with *N*-chloroamines.

Our next task was to evaluate the range of organometallic reagents that could be employed (Table 1). Pleasingly, we found that Mg-, Li-, and Zn-based reagents, featuring a range of alkyl, aryl, alkenyl, and heteroaryl substituents, could be efficiently converted to the corresponding sulfonamides. For the alkyl examples, Grignard reagents were the most efficient compared to the corresponding lithium and zinc reagents (entries 1–4). There was less variation in reactivity for the aryl series (entries 7, 10, and 12). The examples shown in Table 1 feature commercial organometallic reagents as well as organometallics generated by halogen–metal exchange with both zero-valent metal and alkyl metals, and by deprotonation.

**Table 1:** Organometallic reagent scope for the one-pot preparation of sulfonamides (3).^[a]^ 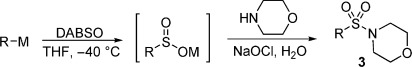

Entry	R—M	Yield [%]	Entry	R—M	Yield [%]
1	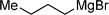	82	9^[b]^	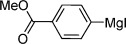	51
2	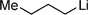	52	10^[c]^	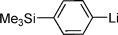	72
3	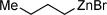	65	11^[c]^	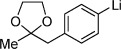	62
4	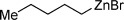	64	12^[d]^	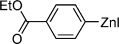	62
5	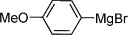	86	13	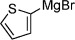	89
6	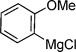	84	14	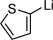	78
7^[b]^	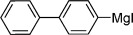	71	15^[e]^	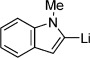	65
8	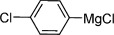	68	16^[f]^	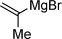	50

[a] Reaction conditions: Organometallic reagent (1 equiv), DABSO (0.6 equiv), THF −40 °C; then amine, H_2_O, and NaOCl at 0 °C followed by stirring at rt; [b] RMgX generated from the corresponding iodide and ^*i*^PrMgCl; [c] RLi generated from the corresponding bromide and ^*n*^BuLi; [d] RZnX generated from the corresponding iodide by zinc insertion; [e] RLi generated through deprotonation with ^*t*^BuLi; [f] Product isolated following wash with 1 m HCl_(aq)_.

We next explored the scope of amines that could be employed (Table 2). A range of primary and secondary amines were found to be compatible with this system (entries 1–4). Pleasingly, aniline derivatives also delivered sulfonamides in good yields (entries 5–9). Amino acids were found to perform well with pure products readily obtained after acidification (entries 10 and 11). The enantiopurity of these substrates was unaffected during the process. Although the formation of *N*-chloro derivatives of amides is known,[17] these nucleophiles were not amenable to the present system, resulting in no sulfonamide formation. We were able to exploit this reactivity difference with the use of an amido-substituted amine nucleophile; the sulfonamide obtained was formed by exclusive reaction at the amine *N*-atom (entry 12).

**Table 2:** Variation of amines employed in the one-pot preparation of sulfonamides 4.^[a]^ 
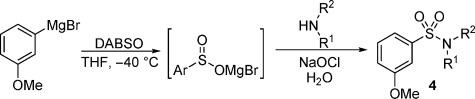

Entry	Amine	Yield [%]	Entry	Amine	Yield [%]
1	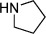	79	7^[c]^	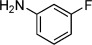	73
2^[b]^	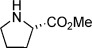	68	8^[c]^	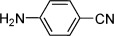	81
3	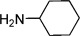	76	9	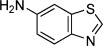	57
4	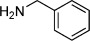	78	10^[d]^	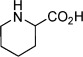	83
5	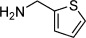	83	11^[b,d]^	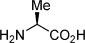	73
6	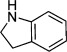	64	12^[c]^	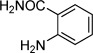	51

[a] Reaction conditions: Grignard (1 equiv), DABSO (0.6 equiv), THF −40 °C; then amine, solvent, and NaOCl at 0 °C followed by stirring at rt; [b] *ee* confirmed by HPLC on a chiral stationary phase; [c] AcOH (5 equiv) added to aid solubility; [d] product isolated following wash with 1 m HCl_(aq)_.

Having examined the general reactivity and scope of the developed sulfonamide synthesis, we next wanted to test the methodology by application to an array synthesis format. In particular, we sought to establish that the developed chemistry could be used to rapidly prepare a collection of molecules featuring the physiochemical properties associated with drug-likeness.[5, 6] This meant selecting a group of functional-group-rich, polar, and relatively small monomers; characteristics often associated as being incompatible with new synthetic methodology.[18] In addition, when selecting the organometallic partners we focused on reagents for which the corresponding sulfonyl chloride was not readily (commercially) available. The reactions were performed as a plate experiment, employing an XT-block reactor, with seven organometallic reagents (M=MgX, Li, and ZnX) and ten amines (Figure 2). Crude compounds were purified by mass-directed preparative HPLC, allowing for isolated product yields to be determined.

**Figure 2:**
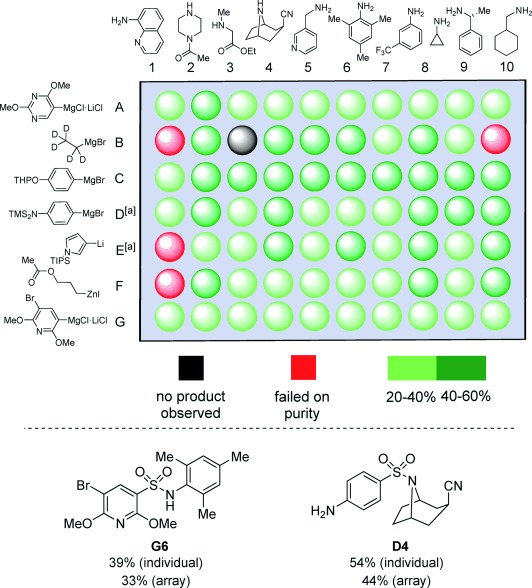
A 70-compound array synthesis of sulfonamides, together with control examples. [a] Desilylated products isolated. THP=tetrahydropyranyl, TMS=trimethylsilyl, TIPS=triisopropylsilyl.

As can be seen from Figure 2, 65 out of a possible 70 organometallic/amine combinations delivered pure sulfonamide products (as determined by LC-MS, and in some cases ^1^H NMR spectroscopy). This equates to a 93 % success rate for the array. Of the five failed examples there was only a single case in which no product was observed. The remaining four “fails” contained impurities from inefficient HPLC purification. To evaluate the effect of performing the reactions in an array format, two substrate combinations were individually prepared, employing the optimized conditions used in Tables 1 and 2. Compounds **G6** and **D4** were obtained in 39 % and 54 % yields, respectively, corresponding to deviations of 6 % and 10 % by comparison with the results from the array synthesis (Figure 2). Although these isolated synthesis yields are respectable, it is the “array yield” of 93 % that should be used to measure the success of the experiment.

From a discovery chemistry perspective it is important to assess the physiochemical properties of the 65 synthesized molecules, and to determine their fit with the various guidelines for medicinal and agrochemical applications.[5, 6] The array had been designed to deliver molecules with logP, topological surface area and molecular weights to fit these criteria; the calculated values are shown graphically in Figure 3.[19] The majority of the products were found to have logP values within the range of 0 and 3, PSA values between 75 and 100 Å^2^, and molecular weights centered on 325 Da; values desirable for drug-like space.[5, 6] The calculated properties for three example sulfonamides prepared in the array are also shown in Figure 3. This encouraging physiochemical profile for the array translated into observed in vivo activity in a range of agrochemical assays. In particular, 67 % of the molecules examined displayed at least partial control in preliminary fungicide, herbicide, and insecticide assays.[16]

**Figure 3:**
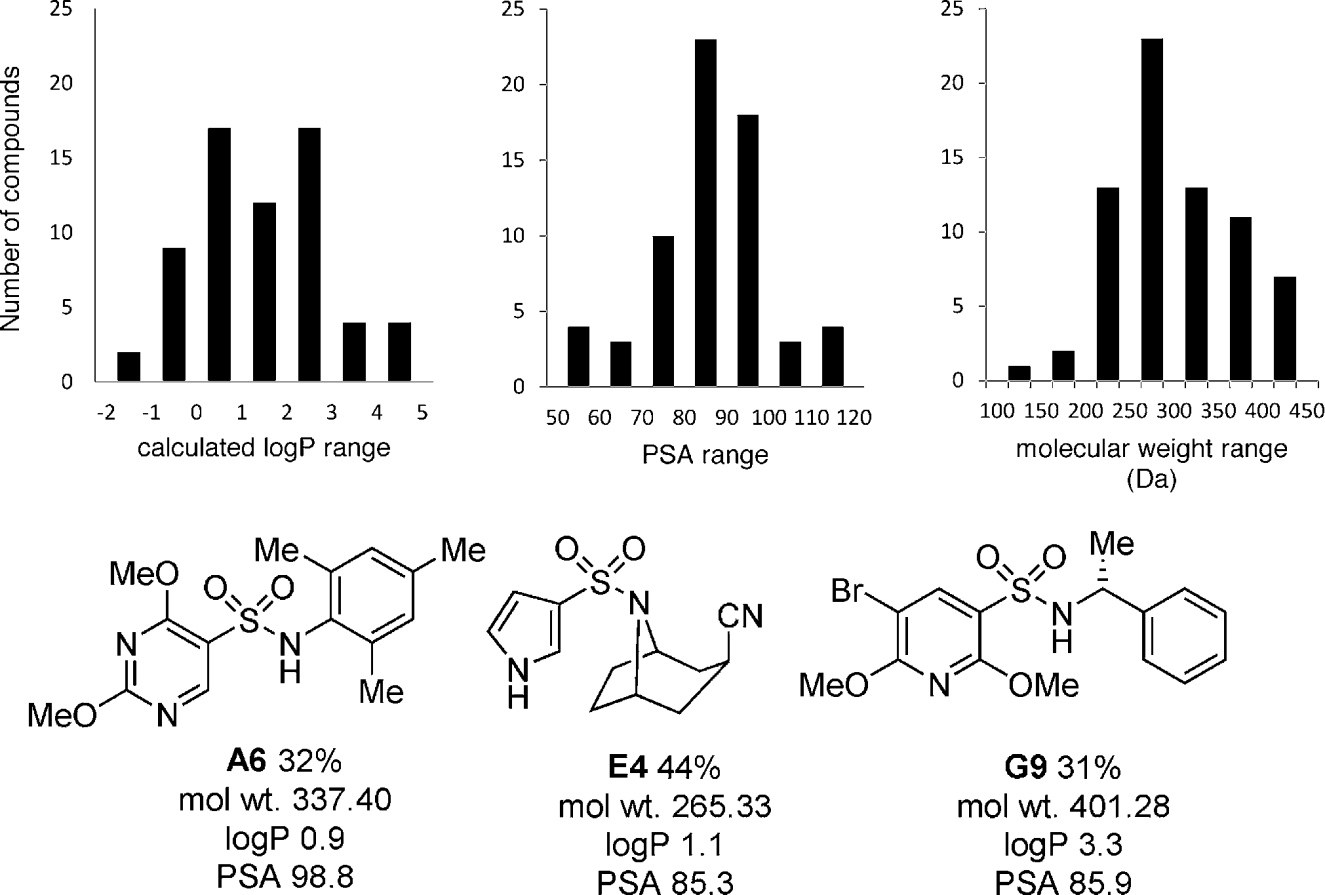
Calculated physiochemical properties for the 65 pure compounds isolated from the array prepared as shown in Figure 2: log*P*, polar surface area (PSA), and molecular weight. Calculated figures for three example synthesized sulfonamides are also shown.

In conclusion, we have developed a simple and efficient one-pot synthesis of alkyl, alkenyl, and (hetero)aryl sulfonamides based on the union of metal sulfinates and *N*-chloroamines, both generated in situ. The robustness of the chemistry allowed the method to be challenged in an array format; this was exemplified by delivering 65 sulfonamides, designed to possess desirable physiochemical properties, corresponding to a 93 % success rate for the array (the “array yield”). The synthesized array, containing 56 previously unknown structures, was shown to possess attractive molecular weights, lipophilicities, and polar surface area values for drug-like space. Stress-testing new methods in this way, i.e., in an array to deliver molecules with drug-like physiochemical properties, provides an alternative way to measure the utility of a new reaction,[18] and could potentially translate directly to the reaction’s usefulness in drug and agrochemical discovery. We believe this practically straightforward method, that employs two classes of readily available substrates, both with potentially diverse members, can serve as an entry into new sulfonamide chemical space, delivering molecules with drug-like properties, and find further applications in array format.[8]
