# Supplementary material for: Combining Organometallic Reagents, the Sulfur Dioxide Surrogate DABSO, and Amines: A One-Pot Preparation of Sulfonamides, Amenable to Array Synthesis
Source: Angew Chem Int Ed Engl. 2014 Nov 27;54(4):1168–71. doi: 10.1002/anie.201409283 (PMC4312890; doi:10.1002/anie.201409283)
Supplement: Supplementary file 1 [file anie0054-1168-sd1.pdf]

Angewandte Chemie
Eine Zeitschrift der Gesellschaft Deutscher Chemiker

Supporting Information
© Wiley-VCH 2015
69451 Weinheim, Germany

Combining Organometallic Reagents, the Sulfur Dioxide Surrogate DABSO, and Amines: A One-Pot Preparation of Sulfonamides, Amenable to Array Synthesis**

*Alex S. Deeming, Claire J. Russell, and Michael C. Willis**

anie_201409283_sm_miscellaneous_information.pdf

Supporting Information

1. Experimental
    - 1.1 General Considerations…………………………………………………………...S2
    - 1.2 Initial screening and optimisation for formation of sulfonamide (2) using morpholine and sodium hypochlorite………………………………………………………….S3
    - 1.3 Characterisation data for compounds prepared in Tables 2 and 3…………………..S3
2. Array Synthesis………………………………………………………………………..S17
    - 2.1 Sulfonamide Data from Array Synthesis………………………………………..S18
3. References…………………………………………………………………………..S33
4. NMR Spectra – General Scope…………………………………………………………..S34
5. Chiral HPLC………………………………………………………………………..S63
6. NMR Spectra – Array Synthesis……………………………………………………………S67
7. LC-MS – Array Synthesis……………………………………………………………….S75
8. Fungicide, Herbicide and Insecticide Assay……………………………………….........S261

Experimental

1.1 General considerations

Chemicals were purchased from Sigma Aldrich, Alfa Aesar or Acros and used without further purification with the exception of DABCO which was sublimed (50 °C, 1 mbar) prior to use and starting materials for organometallic reagent preparation were purified by distillation or re-crystallisation as appropriate. All solvents were purchased from Sigma Aldrich, Fisher Scientific or Rathburn and used directly without further purification. 'Petrol' refers to the fraction of light petroleum ether boiling in the range 40-60 °C. DABSO was prepared from DABCO and SO_2 gas as described in a previous Willis group publication.[1] DABSO was dried under vacuum (10 mbar) for 20 minutes prior to use. 4-Trimethylsilylbromobenzene[2] and 2-(4-bromobenzyl)-2-methyl-1,3-dioxolane[3] were prepared according to literature procedures. Grignard reagents were titrated with salicylaldehyde phenylhydrazone prior to use. Organolithium reagents were titrated against a 1.0 M solution of 2-propanol in toluene with 0.2% 1,10-phenanthroline as the indicator prior to use. The concentration of aqueous sodium hypochlorite was determined by standard sodium thiosulfate/iodine titration.

Reactions were performed with continuous magnetic stirring, under an atmosphere of nitrogen (passed through a Drierite®filled tube), unless otherwise stated, using standard Schlenk techniques and all glassware was dried in an oven (>200 °C, overnight) and allowed to cool under vacuum (10 mbar) prior to use. Microwave heating was carried out using a CEM Discover-S unit. Flash column chromatography was performed using Apollo scientific silica gel 60 (particle size 0.040-0.063 nm) with the indicated eluents. Crude reaction mixtures were dry-loaded and pressure was applied at the column head with hand bellows. Thin Layer Chromatography (TLC) analysis was carried out on Merck Kieselgel 60 PF254 pre-coated aluminium backed sheets and visualised either by UV fluorescence (254 nm) and/or by staining with potassium permanganate ($KMnO_4$).

NMR spectra were recorded at ambient temperature on a Brüker DPX400 (400 MHz) spectrometer. Chemical shifts (δ) are reported in parts per million (ppm) and referenced relative to the residual solvent peak(s) (as specified). Coupling constants (*J*) are given in Hertz (Hz) and rounded to the nearest 0.5 Hz. Assignments were made on the basis of chemical shifts, coupling constants, COSY, HSQC and comparison with spectra of related compounds. Signal multiplicities are denoted as: s, singlet; d, doublet; t, triplet; q, quartet; quin, quintet; sext, sextet; m, multiplet; br, broad; app, apparent. Multiplicities are reported as observed.

Melting points were measured using a Leica Gallen III hot-stage microscope. Low resolution mass spectra were recorded on a Fisons Platform spectrometer (ESI). High resolution mass spectra were measured by the internal service at the University of Oxford using a Bruker Daltronics microTOF spectrometer. *m*/*z* ratio values are reported in Daltons; high resolution values are calculated to four decimal places from the molecular formula, all found within a tolerance of 5 ppm. Infrared spectra were

determined neat using a Bruker Tensor 27 FT spectrometer with an internal range of 600-4000 cm^{-1}. Enantiomeric excess (ee) was determined by HPLC using a Chiralpak AD-H column.

1.2 Initial screening and optimisation for formation of sulfonamide (2) using morpholine and sodium hypochlorite[a]

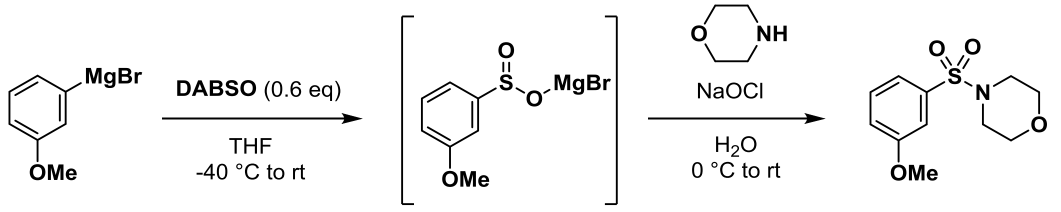

Entry	Solvent	Amine equiv.	NaOCl equiv.	Yield
1	THF/H_2O 1:1	2.0	1.2	38%
2	H_2O	2.0	1.2	51%
3	H_2O	3.0	2.0	60%
4	H_2O	4.0	2.0	66%
5	H_2O	5.0	3.0	78%
6	H_2O	5.0	4.0	78%
7	H_2O	7.5	5.0	79%
8[b]	H_2O	5.0	3.0	79%
9[c]	H_2O	5.0	3.0	73%
10	THF/H_2O 1:1	5.0	3.0	67%

[a] Reaction conditions: 3-MeO-C_6H_4-MgBr (1 equiv.), DABSO (0.6 equiv.), THF -40 °C then amine, solvent and NaOCl at 0 °C, followed by overnight stirring at rt; [b] 2.0 equiv. of DMAP added; [c] 4.0 equiv. of pyridine added.

1.3 Characterisation data for compounds prepared in Tables 2 and 3

General procedure for the synthesis of sulfonamides from organometallic reagent, DABSO, NaOCl and amines, as exemplified by the preparation of 4-[(3-methoxyphenyl)sulfonyl]morpholine

To a reaction tube was added DABSO (36 mg, 0.15 mmol) and THF (1 mL) and the resulting suspension flushed with nitrogen gas for 2 mins. After cooling to -40 °C 3-methoxyphenylmagnesium bromide (0.97 M in THF, 258 μL, 0.25 mmol) was added dropwise and the mixture stirred at this temp for 30 mins. On warming to room temp a strong flow of nitrogen gas was applied to remove the solvent before addition of water (1.5 mL) and morpholine (109 μL, 1.25 mmol). The resulting mixture was cooled to 0-5 °C (ice-water bath) and NaOCl (15.8% aqueous solution, 296 μL, 0.75 mmol) was added dropwise before allowing the reaction to warm to room temp (Note: addition of NaOCl to mixtures containing anilines results in coloured oxidation products which can be flushed off with 100% CH_2Cl_2 on chromatography). After stirring for 16 hrs, sat. $NaS_2O_{3(aq)}$ (10 mL) was added and the mixture stirred for a further 20 mins. The aqueous mixture was then extracted with CH_2Cl_2 (3 × 15 mL) and the combined organic extracts subsequently washed with 1M $HCl_{(aq)}$ (1 × 30 mL). The organic layer was dried ($MgSO_4$), filtered and the solvent removed *in vacuo*. Purification by flash column chromatography (petrol/Et_2O 3:2) afforded the

titled sulfonamide as a white solid (50 mg, 79%); mp 128-129 °C (CH_2Cl_2) [lit.[4] mp 132 °C]; ^{1}H NMR (400 MHz, $CDCl_3$) δ 7.40 (app t, *J* 8.0, 1H, Ar-*H*), 7.26 (d, *J* 8.0, 1H, Ar-*H*) 7.18 (app t, *J* 2.0, 1H, Ar-*H*), 7.08 (ddd, *J* 8.0, 1.5, 1.0, 1H, Ar-*H*), 3.80 (s, 3H, O*Me*), 3.68 (t, *J* 4.5, 4H, NCH_2CH_2), 2.95 (t, *J* 4.5, 4H, NCH_2CH_2); ^{13}C NMR (100 MHz, $CDCl_3$) δ 160.0, 136.2, 130.2, 120.0, 119.1, 112.8, 66.1, 55.7, 46.0; IR ν_{max} (neat)/cm^{-1} 2986, 1582, 1426, 1336 (SO_2), 1240, 1151 (SO_2); LRMS (ESI) *m/z* 258 (50%, $[M+H]^+$), 280 (50%, $[M+Na]^+$), 537 (100%, $[2M+Na]^+$); HRMS (ESI) found *m/z* 280.0617 $[M+Na]^+$, $C_{11}H_{15}NO_4SNa$ requires *m/z* 280.0614. Data in accordance with that previously reported.[4]

4-(Butylsulfonyl)morpholine (Entry **1**, Table **2**)

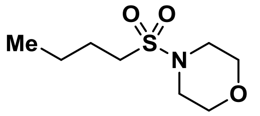

Prepared according to general procedure using *n*-butylmagnesium chloride (2.1 M, 119 µL, 0.25 mmol). Flash column chromatography (petrol/Et_2O 2:3) afforded the titled sulfonamide as a white solid (42 mg, 82%); mp 42-43 °C (CH_2Cl_2) [lit.[5] mp 40-45 °C]; ^{1}H NMR (400 MHz, $CDCl_3$) δ 3.69 (t, *J* 4.5, 4H, NCH_2CH_2), 3.20 (t, *J* 4.5, 4H, NCH_2CH_2), 2.84 (t, *J* 8.0, 2H, SCH_2), 1.78-1.70 (m, 2H, SCH_2CH_2), 1.40 (sext., *J* 7.5, 2H, CH_2CH_3), 0.89 (t, *J* 7.5, 3H, CH_2CH_3); ^{13}C NMR (100 MHz, $CDCl_3$) δ 66.6, 48.6, 45.8, 24.9, 21.8, 13.6; IR ν_{max} (neat)/cm^{-1} 2965, 2863, 1454, 1322 (SO_2), 1259, 1110 (SO_2); LRMS (ESI) *m/z* 208 (70%, $[M+H]^+$), 230 (100%, $[M+Na]^+$); HRMS (ESI) found *m/z* 230.0830 $[M+Na]^+$, $C_8H_{17}NO_3SNa$ requires *m/z* 230.0821. Data in accordance with that previously reported.[5]

4-(Pentylsulfonyl)morpholine (Entry **4**, Table **2**)

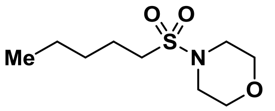

Prepared according to general procedure using *n*-pentylzinc bromide (0.50 M, 250 µL, 0.25 mmol). The aqueous acid wash gave the pure titled sulfonamide as an off-white solid (36 mg, 64%) without further purification; mp 39-40 °C (CH_2Cl_2); ^{1}H NMR (400 MHz, $CDCl_3$) δ 3.69 (t, *J* 4.5, 4H, NCH_2CH_2), 3.20 (t, *J* 4.5, 4H, NCH_2CH_2), 2.84 (t, *J* 8.0, 2H, SCH_2), 1.80-1.72 (m, 2H, SCH_2CH_2), 1.38-1.24 (m, 4H, $CH_2CH_2CH_3$), 0.85 (t, *J* 7.0, 3H, CH_2CH_3); ^{13}C NMR (100 MHz, $CDCl_3$) δ 66.6, 48.8, 45.8, 30.6, 22.6, 22.2, 13.8; IR ν_{max} (neat)/cm^{-1} 2965, 2863, 1454, 1322 (SO_2), 1259, 1110 (SO_2); LRMS (ESI) *m/z* 221 (10%, $[M+H]^+$), 244 (100%, $[M+Na]^+$); HRMS (ESI) found *m/z* 244.0976 $[M+Na]^+$, $C_9H_{19}NO_3SNa$ requires *m/z* 244.0978.

4-[(4-methoxyphenyl)sulfonyl]morpholine (Entry **5**, Table **2**)

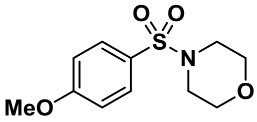

Prepared according to general procedure using 4-methoxyphenylmagnesium bromide (0.51 M in THF, 490 μL, 0.25 mmol). Flash column chromatography (petrol/Et_2O 1:1) afforded the titled sulfonamide as an off-white solid (55 mg, 86%); mp 109-110 °C (CH_2Cl_2) [lit.[6] mp 110-111 °C]; ^1H NMR (400 MHz, $CDCl_3$) δ 7.63 (d, *J* 9.0, 2H, Ar-*H*), 6.95 (d, *J* 9.0, 2H, Ar-*H*), 3.82 (s, 3H, O*Me*), 3.68 (t, *J* 4.5, 4H, NCH_2CH_2), 2.91 (t, *J* 4.5, 4H, NCH_2CH_2); ^{13}C NMR (100 MHz, $CDCl_3$) δ 163.3, 130.0, 126.7, 114.3, 66.1, 55.7, 46.0; LRMS (ESI) *m/z* 258 (90%, $[M+H]^+$), 280 (100%, $[M+Na]^+$); HRMS (ESI) found *m/z* 280.0606 $[M+Na]^+$, $C_{11}H_{15}NO_4SNa$ requires *m/z* 280.0614. Data in accordance with that previously reported.[6]

4-[(2-methoxyphenyl)sulfonyl]morpholine (Entry **6**, Table **2**)

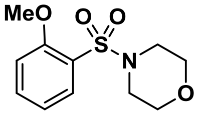

Prepared according to general procedure using 2-methoxyphenylmagnesium bromide (1.0 M in THF, 250 μL, 0.25 mmol). Flash column chromatography (petrol/Et_2O 2:3) afforded the titled sulfonamide as an off-white solid (54 mg, 84%); mp 85-86 °C (CH_2Cl_2) [lit.[6] mp 86-87 °C]; ^1H NMR (400 MHz, $CDCl_3$) δ 7.89 (dd, *J* 4.0, 1.5, 1H, Ar-*H*), 7.55-7.51 (m, 1H, Ar-*H*), 7.06-7.02 (m, 2H, Ar-*H*), 3.93 (s, 3H, O*Me*), 3.72 (t, *J* 4.5, 4H, NCH_2CH_2), 3.24 (t, *J* 4.5, 4H, NCH_2CH_2); ^{13}C NMR (100 MHz, $CDCl_3$) δ 157.0, 134.8, 131.9, 125.8, 120.5, 112.4, 66.8, 56.0, 46.1; LRMS (ESI) *m/z* 258 (20%, $[M+H]^+$), 280 (20%, $[M+Na]^+$), 537 (100%, $[2M+Na]^+$); HRMS (ESI) found *m/z* 280.0621 $[M+Na]^+$, $C_{11}H_{15}NO_4SNa$ requires *m/z* 280.0614. Data in accordance with that previously reported.[6]

4-[(1,1'-biphenyl)-4-ylsulfonyl]morpholine (Entry **7**, Table **2**)

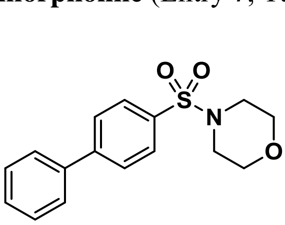

Prepared according to general procedure using (1,1'-biphenyl)-4-ylmagnesium bromide (0.25 mmol). The aryl Grignard reagent was generated *in situ* by dropwise addition of cyclopentylmagnesium chloride (2.0

M, 150 μL, 0.30 mmol) to a solution of 4-iodobiphenyl (70 mg, 0.25 mmol) in THF (1 mL) at -40 °C. The solution was allowed to warm to room temp and was stirred for 1 h before use. Flash column chromatography (petrol/Et_2O 2:3) afforded the titled sulfonamide as an off-white solid (54 mg, 71%); mp 206-207 °C (CH_2Cl_2); ^{1}H NMR (400 MHz, $CDCl_3$) δ 7.75 (d, *J* 8.5, 2H, Ar-*H*), 7.68 (d, *J* 8.5, 2H, Ar-*H*), 7.56-7.53 (m, 2H, Ar-*H*), 7.46-7.40 (m, 2H, Ar-*H*), 7.39-7.34 (m, 1H, Ar-*H*), 3.70 (t, *J* 4.5, 4H, NCH_2CH_2), 2.98 (t, *J* 4.5, 4H, NCH_2CH_2); ^{13}C NMR (100 MHz, $CDCl_3$) δ 146.1, 139.2, 133.6, 129.1, 128.6, 128.4, 127.8, 127.4, 66.1, 46.0; IR ν_{max} (neat)/cm^{-1} 1451, 1346, 1330 (SO_2), 1261, 1108 (SO_2); LRMS (ESI) *m/z* 304 (60%, $[M+H]^+$), 326 (100%, $[M+Na]^+$), 629 (60%, $[2M+Na]^+$); HRMS (ESI) found *m/z* 326.0822 $[M+Na]^+$, $C_{16}H_{17}NO_3SNa$ requires *m/z* 326.0821.

4-[(4-chlorophenyl)sulfonyl]morpholine (Entry **8**, Table **2**)

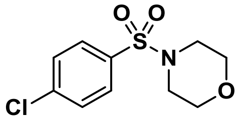

Prepared according to general procedure using 4-chlorophenylmagnesium bromide (1.0 M in THF, 250 μL, 0.25 mmol). Flash column chromatography (petrol/Et_2O 2:3) afforded the titled sulfonamide as an off-white solid (43 mg, 68%); mp 143-144 °C (CH_2Cl_2) [lit.[7] mp 146-147 °C]; ^{1}H NMR (400 MHz, $CDCl_3$) δ 7.63 (d, *J* 8.5, 2H, Ar-*H*), 7.47 (d, *J* 8.5, 2H, Ar-*H*), 3.68 (t, *J* 4.5, 4H, NCH_2CH_2), 2.93 (t, *J* 4.5, 4H, NCH_2CH_2); ^{13}C NMR (100 MHz, $CDCl_3$) δ 139.8, 133.7, 129.5, 129.2, 66.1, 45.9; LRMS (ESI) *m/z* 284 (100%, $[M+Na]^+$); HRMS (ESI) found *m/z* 262.0296 $[M+H]^+$, $C_{10}H_{13}ClNO_3S$ requires *m/z* 262.0299. Data in accordance with that previously reported.[7]

Methyl 4-(morpholinosulfonyl)benzoate (Entry **9**, Table **2**)

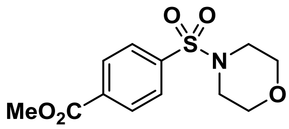

Prepared according to general procedure using 4-[(methoxycarbonyl)phenyl]magnesium bromide (0.25 mmol). The aryl Grignard solution was prepared *in situ* by dropwise addition of isopropylmagnesium bromide (1.85M in THF, 151 μL, 0.28 mmol) to a solution of methyl-4-iodobenzoate (66 mg, 0.25 mmol) in THF (0.8 mL) at -40 °C. The solution was stirred at this temp for 1 h before being transferred *via* syringe to a DABSO-THF suspension. Flash column chromatography (petrol/Et_2O 2:3) afforded the titled sulfonamide as an off-white solid (36 mg, 51%); mp 143 °C (CH_2Cl_2); ^{1}H NMR (400 MHz, $CDCl_3$) δ 8.22 (d, *J* 8.5, 2H, Ar-*H*), 7.84 (d, *J* 8.5, 2H, Ar-*H*), 3.98 (s, 3H, CO_2Me), 3.76 (t, *J* 4.5, 4H, NCH_2CH_2), 3.03 (t, *J* 4.5, 4H, NCH_2CH_2); ^{13}C NMR (100 MHz, $CDCl_3$) δ 160.0, 130.2, 120.0, 119.1, 112.8, 66.1,

55.7, 46.0; IR ν_{max} (neat)/cm^{-1} 2849, 1726 (CO), 1450, 1349 (SO_2), 1260, 1110 (SO_2); LRMS (ESI) *m/z* 286 (30%, $[M+H]^+$), 308 (100%, $[M+Na]^+$); HRMS (ESI) found *m/z* 308.0559 $[M+Na]^+$, $C_{12}H_{15}NO_5SNa$ requires *m/z* 308.0563.

4-[4-(Trimethylsilyl)phenylsulfonyl]morpholine (Entry **10**, Table **2**)

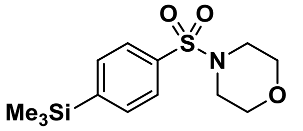

Prepared according to general procedure using 4-[(trimethylsilyl)phenyl]lithium (0.25 mmol). The aryl lithium solution was generated *in situ* by dropwise addition of *n*-butyllithium (1.78 M in hexane, 157 μL, 0.28 mmol) to a solution of 4-trimethylsilylbromobenzene (57 mg, 0.25 mmol) in THF (0.8 mL) at -78 °C. The solution was stirred at this temp for 1 h before being transferred *via* syringe to a DABSO-THF suspension. Flash column chromatography (petrol/Et_2O 1:1) afforded the titled sulfonamide as an off-white solid (54 mg, 72%); mp 144 °C (CH_2Cl_2); ^{1}H NMR (400 MHz, $CDCl_3$) δ 7.65-7.60 (m, 4H, Ar-*H*), 3.68 (t, *J* 4.5, 4H, NCH_2CH_2), 2.94 (t, *J* 4.5, 4H, NCH_2CH_2) 0.23 (s, 9H, Si*Me*$_3$); ^{13}C NMR (100 MHz, $CDCl_3$) δ 148.9, 136.5, 135.3, 128.1, 67.5, 47.3, 0.0; IR ν_{max} (neat)/cm^{-1} 1449, 1348 (SO_2), 1259, 1111 (SO_2), 1085; LRMS (ESI) *m/z* 300 (100%, $[M+H]^+$), 322 (60%, $[M+Na]^+$), 621 (40%, $[2M+Na]^+$) ; HRMS (ESI) found *m/z* 322.0906 $[M+Na]^+$, $C_{13}H_{21}NO_3SSiNa$ requires *m/z* 322.0904.

4-[(4-((2-Methyl-1,3-dioxolan-2-yl)methyl)phenyl)sulfonyl]morpholine (Entry **11**, Table **2**)

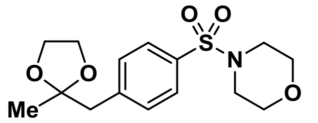

Prepared according to general procedure using 4-[((2-methyl-1,3-dioxolan-2-yl)methyl)phenyl]lithium (0.25 mmol). The aryl lithium solution was generated *in situ* by dropwise addition of *n*-butyllithium (1.8 M in hexane, 153 μL, 0.28 mmol) to a solution of 2-(4-bromobenzyl)-2-methyl-1,3-dioxolane (64 mg, 0.25 mmol) in THF (0.8 mL) at -78 °C. The solution was stirred at this temp for 1 h before being transferred *via* syringe to a DABSO-THF suspension. Flash column chromatography (petrol/Et_2O 2:3) afforded the titled sulfonamide as an off-white solid (59 mg, 62%); mp 115-116 °C (CH_2Cl_2); ^{1}H NMR (400 MHz, $CDCl_3$) δ 7.59 (d, *J* 8.5, 2H, Ar-*H*), 7.40 (d, *J* 8.5, 2H, Ar-*H*), 3.85-3.82 (m, 2H, $COCH_2$), 3.68 (t, *J* 4.5, 4H, NCH_2CH_2), 3.62-3.59 (m, 2H, $COCH_2$), 2.94 (s, 2H, CCH_2), 2.92 (t, *J* 4.5, 4H, NCH_2CH_2), 1.26 (s, 3H, C*Me*); ^{13}C NMR (100 MHz, $CDCl_3$) δ 142.8, 133.0, 131.3, 127.5, 109.2, 66.1, 64.9, 46.0, 45.2, 24.7; IR ν_{max} (neat)/cm^{-1} 1408, 1329 (SO_2), 1224, 1111 (SO_2), 1038; LRMS (ESI) *m/z* 328 (100%, $[M+H]^+$), 350 (20%, $[M+Na]^+$); HRMS (ESI) found *m/z* 350.1032 $[M+Na]^+$, $C_{15}H_{21}NO_5SNa$ requires *m/z* 350.1033.

Ethyl 4-(morpholinosulfonyl)benzoate (Entry **12**, Table **2**)

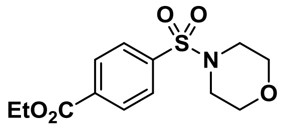

Prepared according to the general procedure using 4-[(ethoxycarbonyl)phenyl)]zinc iodide.[8] Flash column chromatography (CH_2Cl_2/Et_2O 0-5%) afforded the titled sulfonamide as an off-white solid (46 mg, 62%); mp 82-83 °C (CH_2Cl_2); ^{1}H NMR (400 MHz, $CDCl_3$) δ 8.15 (d, *J* 8.5, 2H, Ar-*H*), 7.75 (d, *J* 8.5, 2H, Ar-*H*), 4.38 (q, *J* 7.0, 2H, O$CH_2$$CH_3$), 3.68 (t, *J* 4.5, 4H, NCH_2CH_2), 2.95 (t, *J* 4.5, 4H, NC$H_2$$CH_2$), 1.36 (t, *J* 7.0, 3H, OCH_2CH_3); ^{13}C NMR (100 MHz, $CDCl_3$) δ 165.1, 139.1, 134.6, 130.3, 127.8, 66.1, 61.8, 46.0, 14.3; IR ν_{max} (neat)/cm^{-1} 2855, 1713 (CO), 1451, 1296 (SO_2), 1273, 1108 (SO_2); LRMS (ESI) *m/z* 300 (20%, $[M+H]^+$), 322 (100%, $[M+Na]^+$); HRMS (ESI) found *m/z* 300.0897 $[M+H]^+$, $C_{13}H_{18}NO_5S$ requires *m/z* 300.0900.

4-(Thiophen-2-ylsulfonyl)morpholine (Entry **13**, Table **2**)

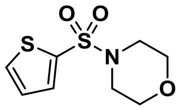

Prepared according to general procedure using 2-thienylmagnesium bromide (0.85 M in THF, 294 μL, 0.25 mmol). Flash column chromatography (petrol/Et_2O 2:3) afforded the titled sulfonamide as an off-white solid (52 mg, 89%); mp 102-103 °C (CH_2Cl_2) [lit.[9] mp 104-105 °C]; ^{1}H NMR (400 MHz, $CDCl_3$) δ 7.59 (dd, *J* 5.0, 1.5, 1H, Ar-*H*), 7.48 (dd, *J* 3.5, 1.5, 1H, Ar-*H*), 7.11 (dd, *J* 5.0, 3.5, 1H, Ar-*H*), 3.71 (t, *J* 4.5, 4H, NCH_2CH_2), 2.99 (t, *J* 4.5, 4H, NC$H_2$$CH_2$); ^{13}C NMR (100 MHz, $CDCl_3$) δ 135.4, 132.8, 132.5, 127.8, 66.0, 46.0; IR ν_{max} (neat)/cm^{-1} 1450, 1401, 1299 (SO_2), 1220, 1112 (SO_2); LRMS (ESI) *m/z* 234 (10%, $[M+H]^+$), 256 (100%, $[M+Na]^+$); HRMS (ESI) found *m/z* 256.0078 $[M+Na]^+$, $C_8H_{11}NO_3S_2Na$ requires *m/z* 256.0073. Data in accordance with that previously reported.[9]

4-[(1-Methyl-1H-indol-2-yl)sulfonyl]morpholine (Entry **15**, Table **2**)

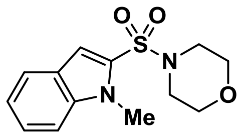

Prepared according to general procedure using 2-lithio-*N*-methylindole (0.31 M in THF, 806 μL, 0.25 mmol). The aryl lithium was prepared as a stock solution by dropwise addition of *t*-butyllithium (1.88 M in hexane, 2.34 mL, 4.40 mmol) to a solution of *N*-methylindole (500 μL, 4.0 mmol) in THF (10 mL) at -78 °C**.** The mixture was then warmed to room temp and stirred for 1 h before use. Flash column

chromatography (petrol/Et_2O 2:3) afforded the titled sulfonamide as an off-white solid (46 mg, 65%); mp 123-124 °C (CH_2Cl_2); ^{1}H NMR (400 MHz, $CDCl_3$) δ 7.62 (d, *J* 8.0, 1H, Ar-*H*), 7.37-7.30 (m, 2H, Ar-*H*), 7.14 (ddd, *J* 8.0, 6.0, 2.0, 1H, Ar-*H*), 7.09 (s, 1H, Ar-*H*), 3.91 (s, 3H, N*Me*) 3.67 (t, *J* 4.5, 4H, NCH_2CH_2), 3.11 (t, *J* 4.5, 4H, NCH_2CH_2); ^{13}C NMR (100 MHz, $CDCl_3$) δ 139.4, 130.2, 125.6, 125.1, 122.6, 121.3, 111.0, 110.4, 66.3, 45.6, 31.6; IR ν_{max} (neat)/cm^{-1} 2855, 1502, 1451, 1349 (SO_2), 1260, 1112 (SO_2); LRMS (ESI) *m/z* 281 (40%, $[M+H]^+$), 303 (100%, $[M+Na]^+$), 583 (80%, $[2M+Na]^+$); HRMS (ESI) found *m/z* 303.0774 $[M+Na]^+$, $C_{13}H_{16}N_2O_3SNa$ requires *m/z* 303.0774.

4-(Prop-1-en-2-ylsulfonyl)morpholine (Entry **16**, Table **2**)

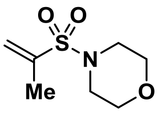

Prepared according to general procedure using isopropenylmagnesium bromide (0.52 M, 480 μL, 0.25 mmol). The acid wash afforded the pure titled sulfonamide as a clear oil (24 mg, 50%) without further purification; ^{1}H NMR (400 MHz, $CDCl_3$) δ 5.91 (m, 1H, C*H*CMe), 5.64 (app q, *J* 1.5, 1H, C*H*CMe), 3.68 (t, *J* 4.5, 4H, NCH_2CH_2), 3.15 (t, *J* 4.5, 4H, NCH_2CH_2), 2.00 (dd, *J* 1.5, 1.0, CH_2C*Me*); ^{13}C NMR (100 MHz, $CDCl_3$) δ 142.69, 124.7, 66.6, 45.7, 17.7; IR ν_{max} (neat)/cm^{-1} 2859, 1449, 1338 (SO_2), 1261, 1219, 1113 (SO_2); LRMS (ESI) *m/z* 214 (100%, $[M+Na]^+$); HRMS (ESI) found *m/z* 192.0688 $[M+H]^+$, $C_7H_{14}NO_3S$ requires *m/z* 192.0689

***N*-[(3-Methoxyphenyl)sulfonyl]pyrrolidine** (Entry **1**, Table **3**)

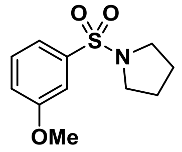

Prepared according to general procedure using 3-methoxyphenylmagnesium bromide (0.97 M in THF, 258 μL, 0.25 mmol) and pyrrolidine (104 μL, 1.25 mmol). Flash column chromatography (petrol/Et_2O 2:3) afforded the titled sulfonamide as an off-white solid (47 mg, 79%); mp 117-118 °C (CH_2Cl_2); ^{1}H NMR (400 MHz, $CDCl_3$) δ 7.39-7.32 (m, 2H, Ar-*H*), 7.28-7.26 (m, 1H, Ar-*H*), 7.04 (app dt, *J* 7.5, 2.0, 1H, Ar-*H*), 3.80 (s, 3H, O*Me*), 3.21-3.17 (m, 4H, NCH_2), 1.72-1.68 (m, 4H, NCH_2CH_2); ^{13}C NMR (100 MHz, $CDCl_3$) δ 159.9, 138.1, 130.1, 119.6, 118.8, 112.4, 55.7, 48.0, 25.3; IR ν_{max} (neat)/cm^{-1} 2977, 1591, 1477, 1333 (SO_2), 1287, 1152 (SO_2); LRMS (ESI) *m/z* 242 (20%, $[M+H]^+$), 264 (10%, $[M+Na]^+$), 505 (100%, $[2M+Na]^+$); HRMS (ESI) found *m/z* 264.0667 $[M+Na]^+$, $C_{11}H_{15}NO_3SNa$ requires *m/z* 264.0665.

(*S*)-Methyl-*N*-[(3-methoxyphenyl)sulfonyl]-pyrrolidine-2-carboxylate (Entry **2**, Table **3**)

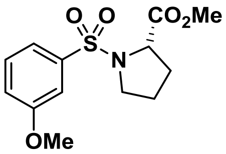

Prepared according to general procedure using 3-methoxyphenylmagnesium bromide (0.97 M in THF, 258 μL, 0.25 mmol) and (*L*)-proline methyl ester hydrochloride (207 mg, 1.25 mmol). Flash column chromatography (petrol/Et_2O 1:3) afforded the titled sulfonamide as a light yellow oil (51 mg, 68%); ^1H NMR (400 MHz, $CDCl_3$) δ 7.40-7.36 (m, 2H, Ar-*H*), 7.34-7.32 (m, 1H, Ar-*H*), 7.05 (app dt, *J* 7.5, 2.0, 1H, Ar-*H*), 4.26 (dd, *J* 8.0, 4.0, 1H, NC*H*CO_2Me), 3.80 (s, 3H, O*Me*), 3.65 (s, 3H, CO_2*Me*), 3.46-3.41 (m, 1H, NCH_2), 3.30-3.24 (m, 1H, NCH_2), 2.00-1.87 (overlapping m, 3H, NCHCH_2 × 2 and NCH_2CH_2), 1.75-1.67 (m, 1H, NCH_2CH_2); ^{13}C NMR (100 MHz, $CDCl_3$) δ 172.5, 159.9, 139.3, 130.1, 119.6, 119.1, 112.2, 60.4, 55.7, 52.4, 48.6, 30.9, 24.7; IR ν_{max} (neat)/cm^{-1} 2954, 1750 (CO), 1596, 1480, 1343 (SO_2), 1153 (SO_2); LRMS (ESI) *m/z* 300 (50%, $[M+H]^+$), 621 (100%, $[2M+Na]^+$); HRMS (ESI) found *m/z* 322.0718 $[M+Na]^+$, $C_{13}H_{17}NO_5SNa$ requires *m/z* 322.0720; ee > 99% (Major = 18.31 mins : Minor = 16.43 mins, *n*-hexane/IPA 90:10, 1 ml/min); $[\alpha]_D$ = -97.13 (0.01 g/mL, $CHCl_3$)

***N*-cyclohexyl-3-methoxybenzenesulfonamide** (Entry **3**, Table **3**)

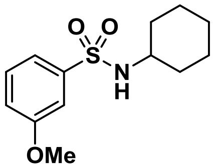

Prepared according to general procedure using 3-methoxyphenylmagnesium bromide (0.97 M in THF, 258 μL, 0.25 mmol) and cyclohexylamine (143 μL, 1.25 mmol). Flash column chromatography (petrol/Et_2O 2:3) afforded the titled sulfonamide as an off-white solid (51 mg, 76%); mp 40 °C (CH_2Cl_2); ^1H NMR (400 MHz, $CDCl_3$) δ 7.40 (app dt, 1H, *J* 7.5, 1.5. Ar-*H*), 7.35-7.31 (m, 2H, Ar-*H*), 7.05 (ddd, *J* 8.5, 2.5, 1.0, 1H, Ar-*H*), 4.62 (d, *J* 7.5, 1H, N*H*), 3.79 (s, 3H, O*Me*), 3.12-3.04 (m, 1H, NHC*H*), 1.72-1.65 (m, 2H, CH_2), 1.59-1.53 (m, 2H, CH_2), 1.48-1.40 (m, 1H, CH_2), 1.22-0.99 (m, 5H, CH_2); ^{13}C NMR (100 MHz, $CDCl_3$) δ 159.9, 142.6, 130.1, 119.1, 118.8, 111.6, 55.7, 52.7, 33.9, 25.1, 24.6; IR ν_{max} (neat)/cm^{-1} 3288 (NH), 2930, 1597, 1476, 1326 (SO_2), 1156 (SO_2); LRMS (ESI) *m/z* 270 (20%, $[M+H]^+$), 292 (10%, $[M+Na]^+$), 561 (100%, $[2M+Na]^+$); HRMS (ESI) found *m/z* 292.0975 $[M+Na]^+$, $C_{13}H_{19}NO_3NaS$ requires *m/z* 292.0978.

***N*-benzyl-3-methoxybenzenesulfonamide** (Entry **4**, Table **3**)

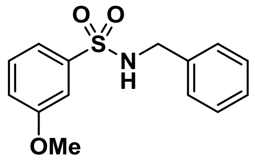

Prepared according to general procedure using 3-methoxyphenylmagnesium bromide (0.97 M in THF, 258 μL, 0.25 mmol) and benzylamine (137 μL, 1.25 mmol). Flash column chromatography (petrol/Et_2O 2:3) afforded the titled sulfonamide as an off-white solid (57 mg, 78%); mp 77-78 °C (CH_2Cl_2) [lit.[10] mp 80 °C]; ^{1}H NMR (400 MHz, $CDCl_3$) δ 7.39 (app dt, 1H, *J* 7.5, 1.5, Ar-*H*), 7.34 (app t, *J* 8.0, 1H, Ar-*H*), 7.29 (t, *J* 2.0, 1H, Ar-*H*), 7.23-7.17 (m, 3H, Ar-*H*), 7.14-7.11 (m, 2H, Ar-*H*), 7.03 (ddd, *J* 8.0, 2.5, 1.5, 1H, Ar-*H*), 4.71 (t, *J* 6.0, 1H, N*H*), 4.08 (d, *J* 6.0, 1H, CH_2NH), 3.76 (s, 3H, O*Me*);^{13}C NMR (100 MHz, $CDCl_3$) δ 160.0, 141.0, 136.2, 130.2, 128.7, 128.0, 127.9, 119.3, 119.2, 111.8, 55.7, 47.4; LRMS (ESI) *m/z* 278 (40%, $[M+H]^+$), 300 (100%, $[M+Na]^+$), 577 (100%, $[2M+Na]^+$); HRMS (ESI) found *m/z* 300.0665 $[M+Na]^+$, $C_{14}H_{15}NO_3SNa$ requires *m/z* 300.0665. Data in accordance with that previously reported.[10]

3-Methoxy-*N*-(thiophen-2-ylmethyl)benzenesulfonamide (Entry **5**, Table **3**)

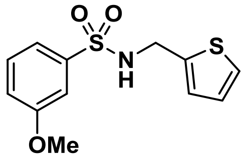

Prepared according to general procedure using 3-methoxyphenylmagnesium bromide (0.97 M in THF, 258 μL, 0.25 mmol) and 2-thiophenemethylamine (120 μL, 1.25 mmol). Flash column chromatography (petrol/Et_2O 1:1) afforded the titled sulfonamide as a white solid (59 mg, 83%); mp 70-71 °C (CH_2Cl_2); ^{1}H NMR (400 MHz, $CDCl_3$) δ 7.49 (app dt, *J* 8.0, 1.5, 1H, Ar-*H*), 7.45 (app t, *J* 8.0, 1H, Ar-*H*), 7.39 (app t, *J* 2.5, 1H, Ar-*H*), 7.23 (dd, *J* 5.0, 1.5, 1H, Ar-*H*), 7.14 (ddd, *J* 8.0, 2.5, 1.5, 1H, Ar-*H*), 6.92-6.88 (overlapping m, 2H, Ar-*H*), 4.80 (t, *J* 6.0, 1H, N*H*), 4.40 (d, *J* 6.0, 2H, CH_2NH), 3.88 (s, 3H, O*Me*); ^{13}C NMR (100 MHz, $CDCl_3$) δ 160.0, 140.9, 138.8, 130.2, 126.9, 126.6, 125.9, 119.4, 119.3, 111.7, 55.7, 42.2; IR ν_{max} (neat)/cm^{-1} 3288 (NH), 1597, 1485, 1417, 1314 (SO_2), 1150 (SO_2); LRMS (ESI) *m/z* 306 (100%, $[M+Na]^+$), 589 (90%, $[2M+Na]^+$); HRMS (ESI) found *m/z* 306.0227 $[M+Na]^+$, $C_{12}H_{13}NO_3S_2Na$ requires *m/z* 306.0229.

***N*-[(3-Methoxyphenyl)sulfonyl]indoline** (Entry **6**, Table **3**)

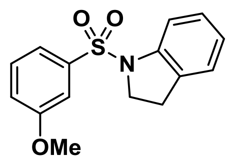

Prepared according to general procedure using 3-methoxyphenylmagnesium bromide (0.97 M in THF, 258 μL, 0.25 mmol) and indoline (149 mg, 1.25 mmol). Flash column chromatography (Et_2O /CH_2Cl_2 0-5%) afforded the titled sulfonamide as an off-white solid (46 mg, 64%); mp 97 °C (CH_2Cl_2); ^{1}H NMR (400 MHz, $CDCl_3$) δ 7.60 (d, *J* 8.0, 1H, Ar-*H*), 7.31 (app dt, *J* 7.5, 1.5, 1H, Ar-*H*), 7.27 (app t, *J* 8.0, 1H, Ar-*H*), 7.16 (app t, *J* 2.0, 1H, Ar-*H*), 7.13 (app t, *J* 8.0, 1H, Ar-*H*), 7.03-6.98 (overlapping m, 2H, Ar-*H*), 6.92 (app td, *J* 7.5, 1.0, 1H, Ar-*H*), 3.86 (t, *J* 8.5, 2H, NCH_2), 3.66 (s, 3H, O*Me*), 2.81 (t, *J* 8.5, 2H, NCH_2CH_2); ^{13}C NMR (100 MHz, $CDCl_3$) δ 159.8, 142.0, 138.0, 132.0, 130.0, 127.7, 125.2, 124.0, 119.7, 119.4, 115.2, 111.8, 55.5, 50.0, 27.9; IR ν_{max} (neat)/cm^{-1} 1578, 1480, 1435, 1349 (SO_2), 1292, 1150 (SO_2); LRMS (ESI) *m/z* 290 (40%, $[M+H]^+$), 312 (80%, $[M+Na]^+$), 601 (100%, $[2M+Na]^+$); HRMS (ESI) found *m/z* 312.0667 $[M+Na]^+$, $C_{15}H_{15}NO_3SNa$ requires *m/z* 312.0665.

***N*-(3-fluorophenyl)-3-methoxybenzenesulfonamide** (Entry **7**, Table **3**)

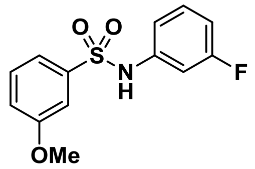

Prepared according to general procedure using 3-methoxyphenylmagnesium bromide (0.97 M in THF, 258 µL, 0.25 mmol), acetic acid (70 μL, 1.25 mmol) and 3-fluoroaniline (120 μL, 1.25 mmol). Flash column chromatography (Et_2O /CH_2Cl_2 0-5%)afforded the titled sulfonamide as an off-white solid (51 mg, 73%); mp 87 °C (CH_2Cl_2); ^{1}H NMR (400 MHz, $CDCl_3$) δ 7.41-7.34 (m, 2H, Ar-*H*), 7.29 (br s, 1H, N*H*), 7.20 (app td, *J* 8.5, 6.5, 1H, Ar-*H*), 7.08 (app dt, *J* 7.5, 2.0, 1H, Ar-*H*), 6.91 (app dt, *J* 10.0, 2.0, 1H, Ar-*H*), 6.83-6.80 (m, 3H, Ar-*H*), 3.78 (s, 3H, O*Me*); ^{13}C NMR (100 MHz, $CDCl_3$) δ 163.1 (d, J_{CF} 247.0), 159.9, 139.8, 138.0 (d, J_{CF} 10.0), 130.6 (d, J_{CF} 9.5), 130.2, 119.9, 119.3, 116.6 (d, J_{CF} 3.0), 112.2 (d, J_{CF} 21.0), 111.7, 108.5 (d, J_{CF} 25.0), 55.6; ^{19}F NMR (377 MHz, $CDCl_3$) δ -110.8; IR ν_{max} (neat)/cm^{-1} 3287 (NH), 1599, 1488, 1324 (SO_2), 1162 (SO_2); LRMS (ESI) *m/z* 282 (10%, $[M+H]^+$), 304 (100%, $[M+Na]^+$); HRMS (ESI) found *m/z* 304.0416 $[M+Na]^+$, $C_{13}H_{12}FNO_3SNa$ requires *m/z* 304.0414.

***N*-(4-cyanophenyl)-3-methoxybenzenesulfonamide** (Entry **8**, Table **3**)

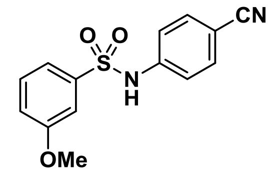

Prepared according to general procedure using 3-methoxyphenylmagnesium bromide (0.97 M in THF, 258 μL, 0.25 mmol), acetic acid (70 μL, 1.25 mmol) and 4-aminobenzonitrile (148 mg, 1.25 mmol). Flash column chromatography (Et_2O /CH_2Cl_2 0-5%) afforded the titled sulfonamide as an off-white solid (59 mg, 81%); mp 156-157 °C (CH_2Cl_2); ^{1}H NMR (400 MHz, CD_3CN) δ 8.44 (br s, 1H, N*H*), 7.62 (d, *J* 9.0, 2H, Ar-*H*), 7.49-7.42 (m, 2H, Ar-*H*), 7.38-7.35 (m, 1H, Ar-*H*), 7.29 (d, *J* 9.0 , 2H, Ar-*H*), 7.19 (ddd, *J* 7.5, 2.5, 2.0, 1H, Ar-*H*), 3.83 (s, 3H, O*Me*);^{13}C NMR (100 MHz, CD_3CN) δ 165.3, 147.0, 145.4, 138.9, 135.9, 124.8, 124.6, 124.4, 123.7, 117.2, 112.4, 60.8; IR ν_{max} (neat)/cm^{-1} 3223 (NH), 2232 (CN), 1597, 1430, 1315 (SO_2), 1288, 1150 (SO_2); LRMS (ESI) *m/z* 289 (10%, $[M+H]^+$), 311 (100%, $[M+Na]^+$); HRMS (ESI) found *m/z* 287.0497 $[M-H]^-$, $C_{14}H_{11}N_2O_3S$ requires *m/z* 287.0496.

***N*-(benzothiazol-6-yl)-3-methoxybenzenesulfonamide** (Entry **9**, Table **3**)

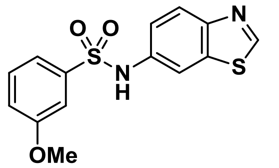

Prepared according to general procedure using 3-methoxyphenylmagnesium bromide (0.97 M in THF, 258 μL, 0.25 mmol) and 6-aminobenzothiazole (188 mg, 1.25 mmol). Flash column chromatography (Et_2O /CH_2Cl_2 0-5%) afforded the titled sulfonamide as an off-white solid (46 mg, 57%); mp 130-131 °C (CH_2Cl_2); ^{1}H NMR (400 MHz, $CDCl_3$) δ 8.93 (s, 1H, Ar-*H*), 8.09 (br s, 1H, N*H*), 7.84 (d, *J* 9.0, 1H, Ar-*H*), 7.76 (d, *J* 2.0, 1H, Ar-*H*), 7.29 (app t, *J* 8.0, 1H, Ar-*H*), 7.24 (app dt, *J* 8.0, 1.5, 1H, Ar-*H*), 7.19-7.15 (m, 2H, Ar-*H*), 7.01 (ddd, *J* 8.0, 2.5, 1.5, 1H, Ar-*H*), 3.66 (s, 3H, O*Me*); ^{13}C NMR (100 MHz, $CDCl_3$) δ 160.4, 155.6, 151.3, 140.7, 135.6, 135.4, 130.9, 124.1, 121.2, 121.1(8), 119.7, 114.9, 112.4, 55.9; IR ν_{max} (neat)/cm^{-1} 3198 (NH), 1595, 1466, 1323 (SO_2), 1245, 1151 (SO_2); LRMS (ESI) *m/z* 321 (100%, $[M+H]^+$), 343 (60%, $[M+Na]^+$), 663 (10%, $[2M+Na]^+$); HRMS (ESI) found *m/z* 321.0360 $[M+H]^+$, $C_{14}H_{13}N_2O_3S_2$ requires *m/z* 321.0362.

1-[(3-Methoxyphenyl)sulfonyl]piperidine-2-carboxylic acid (Entry **10**, Table **3**)

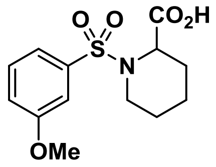

Prepared according to general procedure using 3-methoxyphenylmagnesium bromide (0.97 M in THF, 258 μL, 0.25 mmol) and (*D*)-(*L*) pipecolinic acid (161 mg, 1.25 mmol). The aqueous acid wash gave the pure titled sulfonamide as an off-white solid (62 mg, 83%) without further purification; mp 122-123 °C (CH_2Cl_2); ^{1}H NMR (400 MHz, $CDCl_3$) δ 8.65 (br s, 1H, CO_2H), 7.34-7.30 (m, 2H, Ar-*H*), 7.27-7.25 (m, 1H, Ar-*H*), 7.04-7.00 (m, 1H, Ar-*H*), 4.71 (app d, *J* 5.0, 1H, C*H*CO_2H), 3.78 (s, 3H, O*Me*), 3.72-3.66 (app d, *J* 12.5, 1H, NCH_2), 3.16 (td, *J* 12.5, 3.0, 1H, NCH_2), 2.14-2.07 (m, 1H, CHCH_2), 1.81-1.76 (m, 1H, CHCH_2), 1.71-1.59 (overlapping m, 2H, NCH_2CH_2 and $CHCH_2CH_2$), 1.44-1.23 (overlapping m, 2H, NCH_2CH_2 and $CHCH_2CH_2$); ^{13}C NMR (100 MHz, $CDCl_3$) δ 176.7, 159.8, 141.0, 130.0, 119.3, 118.9, 111.9, 55.6, 54.9, 42.7, 27.5, 24.4, 20.0; IR ν_{max} (neat)/cm^{-1} 2726 (OH), 1706 (CO), 1599, 1488, 1318 (SO_2), 1112 (SO_2); LRMS (ESI) *m/z* 300 (100%, $[M+H]^+$), 621 (50%, $[2M+Na]^+$); HRMS (ESI) found *m/z* 300.0894 $[M+H]^+$, $C_{13}H_{18}NO_5S$ requires *m/z* 300.0900.

(*S*)-[(3-methoxyphenyl)sulfonyl)]alanine (Entry **11**, Table **3**)

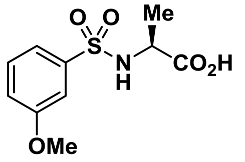

Prepared according to general procedure using 3-methoxyphenylmagnesium bromide (0.97 M in THF, 258 μL, 0.25 mmol) and (*L*)-alanine (111 mg, 1.25 mmol). The aqueous acid wash gave the pure titled sulfonamide as a white solid (47 mg, 73%) without further purification; mp 126-127 °C (CH_2Cl_2); ^{1}H NMR (400 MHz, CD_3OD) δ 7.36-7.30 (m, 2H, Ar-*H*), 7.29-7.27 (m, 1H, Ar-*H*) 7.04 (app dt, *J* 7.0, 2.5, 1H, Ar-*H*), 3.80 (q, *J* 7.0, 1H, C*H*NH), 3.75 (s, 3H, O*Me*), 1.20 (d, *J* 7.0, 3H, CH*Me*); ^{13}C NMR (100 MHz, CD_3OD) δ 173.9, 160.0, 142.0, 129.8, 118.7, 118.4, 111.5, 54.7, 51.4, 18.2; IR ν_{max} (neat)/cm^{-1} 3257 (NH), 1709 (CO), 1476, 1334 (SO_2), 1138 (SO_2); LRMS (ESI) *m/z* 260 (20%, $[M+H]^+$), 282 (100%, $[M+Na]^+$), 541 (60%, $[2M+Na]^+$); HRMS (ESI) found *m/z* 260.0581 $[M+H]^+$, $C_{10}H_{14}NO_5S$ requires *m/z* 260.0587; ee > 99% (Major = 17.97 mins, *n*-hexane/IPA 60:40, 1 ml/min); $[\alpha]_D$ = +29.5 (0.01 g/mL, $CHCl_3$).

2-[(3-Methoxyphenyl)sulfonamido]benzamide (Entry **12**, Table **3**)

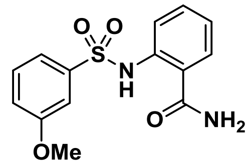

Prepared according to general procedure using 3-methoxyphenylmagnesium bromide (0.97 M in THF, 258 μL, 0.25 mmol), acetic acid (70 μL, 1.25 mmol) and anthranilamide (170 mg, 1.25 mmol). Flash column chromatography (Et_2O /CH_2Cl_2 0-5%) afforded the titled sulfonamide as an off-white solid (39 mg, 51%); mp 128-129 °C (CH_2Cl_2); ^{1}H NMR (400 MHz, $CDCl_3$) δ 10.88 (s, 1H, SO_2NH), 7.65 (dd, *J* 8.5, 1.0, 1H, Ar-H), 7.39-7.32 (overlapping m, 3H, Ar-*H*), 7.26-7.22 (overlapping m, 2H, Ar-*H*), 7.00 (app td, *J* 8.0, 1.0, 1H, Ar-*H*), 6.95 (ddd, *J* 8.5, 2.5, 1.0, 1H, Ar-*H*), 5.92-5.62 (overlapping br s, 2H, CON*H*$_2$), 3.72 (s, 3H, O*Me*); ^{13}C NMR (100 MHz, $CDCl_3$) δ 170.5, 159.8, 140.6, 139.5, 133.4, 130.0, 127.5, 123.5, 121.3, 119.6(3), 119.5(9), 119.5, 111.6, 55.6; IR ν_{max} (neat)/cm^{-1} 3419 (CON-H), 3201 (NH), 1671 (CO), 1484, 1380, 1333 (SO_2), 1113 (SO_2); LRMS (ESI) *m/z* 307 (80%, $[M+H]^+$), 329 (100%, $[M+Na]^+$), 635 (40%, $[2M+Na]^+$); HRMS (ESI) found *m/z* 307.0741 $[M+H]^+$, $C_{14}H_{15}N_2O_4S$ requires *m/z* 307.0747.

8-[(4-Aminophenyl)sulfonyl]-8-azabicyclo[3.2.1]octane-3-carbonitrile (**D4**)

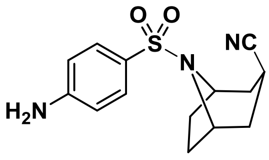

Prepared according to general procedure using 4-[Bis(trimethylsilyl)amino]phenylmagnesium bromide (0.48 M in THF, 520 μL, 0.25 mmol) and exo-8-azabicyclo[3.2.1]octane-3-carbonitrile (170 mg, 1.25 mmol). Flash column chromatography (petrol/EtOAc 1:1) afforded the titled sulfonamide as an off-white solid (40 mg, 54%); mp 181-182 °C (CH_2Cl_2); ^{1}H NMR (400 MHz, $CDCl_3$) δ 7.55 (d, *J* 9.0, 2H, Ar-*H*), 6.69 (d, *J* 9.0, 2H, Ar-*H*), 4.87 (br s, 2H, N*H*$_2$), 4.19-4.15 (m, 2H, NC*H*), 2.96 (tt, *J* 11.5, 6.5, 1H, C*H*CN), 2.00-1.89 (m, 4H, CNCHC*H*$_2$), 1.55-1.44 (m, 4H, NCHC*H*$_2$C*H*$_2$); ^{13}C NMR (100 MHz, $CDCl_3$) δ 153.2, 129.9, 126.6, 122.6, 113.9, 56.5, 36.2, 27.7, 20.8; IR ν_{max} (neat)/cm^{-1} 3542, 3362, 3251, 2968, 1636, 1348 (SO_2), 1148 (SO_2); LRMS (ESI) *m/z* 292 (100%, $[M+H]^+$), 314 (50%, $[M+Na]^+$); HRMS (ESI) found *m/z* 292.1110 $[M+H]^+$, $C_{14}H_{18}N_3O_2S$ requires *m/z* 292.1114.

5-Bromo-*N*-mesityl-2,6-dimethoxypyridine-3-sulfonamide (G6)

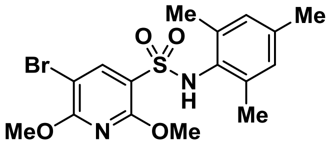

Prepared according to general procedure using 3-bromo-2,6-dimethoxypyridinyl-5-magnesium chloride lithium chloride (0.25 mmol) and 2,4,6-trimethylaniline (181 μL, 1.25 mmol). The aryl Grignard reagent was generated *in situ* by dropwise addition of isopropylmagnesium chloride.LiCl solution (1.17 M in THF, 214 μL, 0.275 mmol) to a solution of 3,5-dibromo-2,6-dimethoxypyridine (89 mg, 0.25 mmol) in THF (1 mL) at 0 °C. The mixture was stirred at room temp for 1 h before being transferred to a DABSO-THF suspension. Flash column chromatography (CH_2Cl_2/Et_2O 0-5%) afforded the titled sulfonamide as an off-white solid (40 mg, 39%); mp 213-214 °C (CH_2Cl_2); ^1H NMR (400 MHz, $CDCl_3$) δ 8.03 (s, 1H, Ar-*H*), 6.78 (s, 2H, Ar-*H*), 6.25 (s, 1H, N*H*), 4.07 (s, 3H, O*Me*), 4.01 (s, 3H, O*Me*), 2.17 (s, 3H, Ar*Me*), 2.05 (s, 6H, 2 × Ar*Me*); ^{13}C NMR (100 MHz, $CDCl_3$) δ 161.2, 158.0, 143.2, 138.0, 137.5, 129.9, 129.5, 117.7, 95.5, 55.3, 54.9, 20.9, 18.8; IR ν_{max} (neat)/cm^{-1} 3273, 2995, 1567, 1311 (SO_2), 1160 (SO_2); LRMS (ESI) *m/z* 415 (90%, $[M+H]^+$), 417 (100%, $[M+H]^+$), 437 (90%, $[M+Na]^+$), 439 (100%, $[M+Na]^+$); HRMS (ESI) found *m/z* 415.0316 $[M+H]^+$ and 417.0294 $[M+H]^+$, $C_{16}H_{20}BrN_2O_4S$ requires *m/z* 415.0322 and 417.0301.

2. Array Synthesis

The array synthesis of sulfonamides was performed as a matrix using a Mettler-Toledo-Bohdan XT block equipped with an inert/purging manifold. The experiments were conducted in two runs using the same ten amines each time with 4 organometallic reagents in the first run and three in the second (1 × 40 combinations and 1 × 30 combinations). Non-commercially available organometallic reagents were prepared as stock solutions. 2,4-Dimethoxypyrimindin-5-ylmagnesium chloride was prepared from 5-iodo-2,4-dimethoxy-pyrimidine and ^{i}PrMgCl.LiCl.[11] d_5-Ethylmagnesium bromide was prepared from d_5-bromoethane and Mg turnings.[12] 3-Lithio-1-(triisopropysilyl)pyrrole was prepared from 3-bromo-1-(triisopropysilyl)pyrrole and ^{t}BuLi. 3-Acetoxypropylzinc iodide was prepared from 3-acetoxypropyl iodide and activated Zn dust.[13] 3-Bromo-2,6-dimethoxypyridinyl-5-magnesium chloride was prepared from 3,5-dibromo-2,6-dimethoxypyridine and ^{i}PrMgCl.LiCl.[14]

Typical Run:

Oven-dried test tubes were placed in the block reactor which was connected to a manifold and the tubes were cooled by flushing with nitrogen gas. DABSO (36 mg, 0.15 mmol) was added to each of the tubes which were subsequently flushed with nitrogen for 2 mins. THF (0.5 mL) was added to the tubes which were then cooled to -40 °C followed by addition of the corresponding organometallic reagent (0.25 mmol). The resulting mixtures were stirred at this temp for 30 mins before being allowed to warm to room temp. To each of the tubes was added sequentially, H_2O (1 mL), amine (1.25 mmol) and NaOCl (15.8% aqueous solution, 296 μL, 0.75 mmol). The resulting mixtures were stirred at room temp for 16 h before being transferred to glass vials containing sat. NaS_2O_3 $_{(aq)}$ (10 mL) and stirred for a further 30 mins. EtOAc (10 mL) was added to each of the vials which were vigorously shaken (robotic assisted agitation) for 1 min before automated extraction of the organic layer *via* syringe. This process was repeated 3 times before removing the solvent by flushing with hot air (40 °C). The resulting crude reaction mixtures were purified using automated preparative liquid chromatography-mass spectrometry to deliver the desired sulfonamides as identified by LC-MS (retention times given in minutes). Representative ^{1}H NMR spectra were obtained for certain examples where LC-MS data contained discrepancies and as confirmation of purity. The four examples that failed by purity were judged to have done so by LC-MS and ^{1}H NMR Spectroscopy.

Purification of samples:

Compounds were purified by mass directed prep HPLC using a mixed trigger of UV with ES+ on a Waters Fraction Lynx system comprising a 2767 injector/collector with a 2525 gradient pump, pump control module two 515 isocratic pumps, CFO, 2996 photodiode array, 2420 ELSD and Micromass

ZQ2000. A Waters XBridge dC18 5micron 19x10mm guard column was used with an XBridge dC18 5micron OBD 30x100mm prep column.

The preparative HPLC was conducted employing a generic 11.4 minute run time using H_2O with 10mM ammonium acetate (solvent A) and CH_3CN (solvent B). Isolated compounds were transferred to individual vials by dissolving in DMSO.

Reaction set-up: XT reactor block

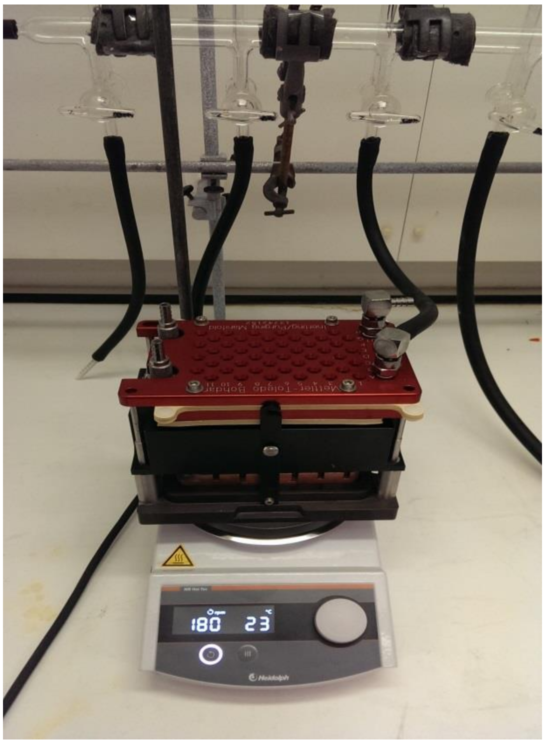

2.1 Sulfonamide Data from Array Synthesis

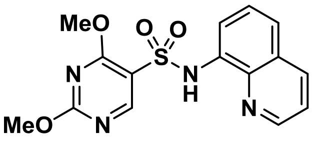

A1

Product obtained as a yellow solid (20 mg, 23%). LC-MS data – Ret. time 0.64: MS ES+ *m/z* 347 (100%, $[M+H]^+$); MS ES- *m/z* 345 ($[M-H]^-$); ^{1}H NMR (400 MHz, $CDCl_3$) δ 9.58 (br s, 1H, SO_2NH), 8.81 (dd, *J* 4.0, 1.5, 1H, Ar-*H*), 8.77 (s, 1H, Ar-*H*), 8.13 (dd, *J* 8.5, 1.5, 1H, Ar-*H*), 7.81 (dd, *J* 7.0, 2.0, 1H, Ar-*H*), 7.53-7.40 (m, 3H, Ar-*H*), 3.99 (s, 3H, O*Me*), 3.95 (s, 3H, O*Me*).

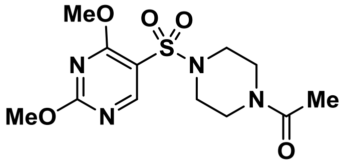

A2

Product obtained as an off-white solid (36 mg, 44%). LC-MS data – Ret. time 0.36: MS ES+ *m/z* 331 (100%, $[M+H]^+$), 353 (40%, $[M+Na]^+$).

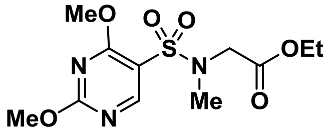

A3

Product obtained as an off-white solid (22 mg, 28%). LC-MS data – Ret. time 0.51: MS ES+ *m/z* 320 (100%, $[M+H]^+$), 342 (20%, $[M+Na]^+$).

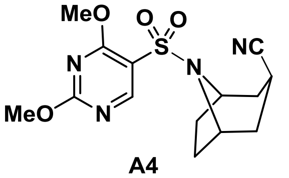

A4

Product obtained as an off-white solid (34 mg, 40%). LC-MS data – Ret. time 0.49: MS ES+ *m/z* 339 (100%, $[M+H]^+$), 361 (20%, $[M+Na]^+$).

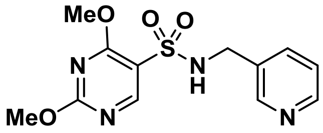

A5

Product obtained as a yellow solid (28 mg, 36%). LC-MS data – Ret. time 0.28: MS ES+ *m/z* 311 (100%, $[M+H]^+$); MS ES- *m/z* 309 (100%, $[M-H]^-$).

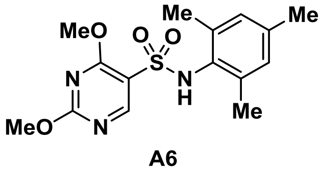

A6

Product obtained as an off-white solid (27 mg, 32%). LC-MS data – Ret. time 0.65: MS ES+ *m/z* 338 (100%, $[M+H]^+$); MS ES- *m/z* 336 (100%, $[M-H]^-$).

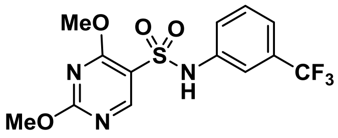

A7

Product obtained as an off-white solid (22 mg, 24%). LC-MS data – Ret. time 0.65: MS ES+ *m/z* 364 (100%, $[M+H]^+$); MS ES- *m/z* 362 (100%, $[M-H]^-$).

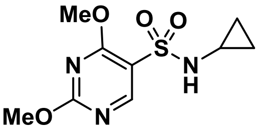

A8

Product obtained as an off-white solid (26 mg, 40%). LC-MS data – Ret. time 0.42: MS ES+ *m/z* 260 (100%, $[M+H]^+$); MS ES- *m/z* 258 (100%, $[M-H]^-$).

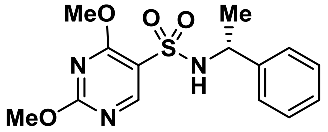

A9

Product obtained as a white solid (28 mg, 35%). LC-MS data – Ret. time 0.55: MS ES+ *m/z* 324 (100%, $[M+H]^+$), 346 (100%, $[M+Na]^+$); MS ES- *m/z* 322 (100%, $[M-H]^-$).

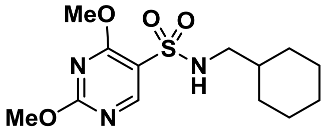

A10

Product obtained as an off-white solid (28 mg, 36%). LC-MS data – Ret. time 0.68: MS ES+ *m/z* 316 (100%, $[M+H]^+$); MS ES- *m/z* 314 (100%, $[M-H]^-$).

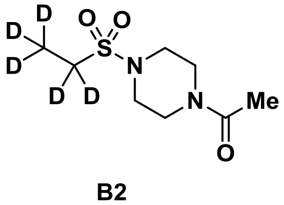

B2

Product obtained as an off-white solid (26 mg, 46%). LC-MS data – Ret. time 0.30: MS ES+ *m/z* 226 (100%, $[M+H]^+$), 248 (40%, $[M+Na]^+$); ^{1}H NMR (400 MHz, $CDCl_3$) δ 3.71 (t, *J* 5.0, 2H, N(COMe)CH_2),

3.55 (t, *J* 5.0, 2H, N(COMe)CH_2), 3.32 (t, *J* 5.0, 2H, N(COMe)CH_2CH_2), 3.28 (t, *J* 5.0, 2H, N(COMe)CH_2CH_2), 2.12 (s, 3H, CO*Me*).

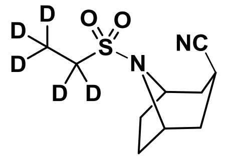

B4

Product obtained as an off-white solid (26 mg, 45%). LC-MS data – Ret. time 0.42: MS ES+ *m/z* 234 (100%, $[M+H]^+$), 256 (25%, $[M+Na]^+$).

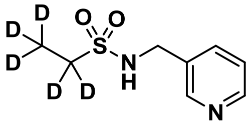

B5

Product obtained as an off-white solid (27 mg, 53%). LC-MS data – Ret. time 0.25: MS ES+ *m/z* 205 (100%, $[M+H]^+$); MS ES- *m/z* 203 (80%, $[M-H]^-$).

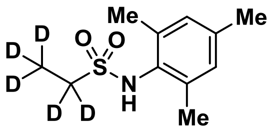

B6

Product obtained as an off-white solid (29 mg, 50%). LC-MS data – Ret. time 0.58: MS ES+ *m/z* 255 (20%, $[M+Na]^+$); MS ES- *m/z* 231 (20%, $[M-H]^-$); ^{1}H NMR (400 MHz, $CDCl_3$) δ 6.84 (s, 2H, Ar-*H*), 5.61 (br s, 1H, N*H*), 2.31 (s, 6H, 2 × Ar*Me*), 2.20 (s, 3H, Ar*Me*).

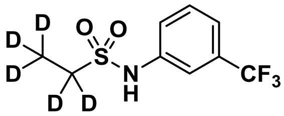

B7

Product obtained as a yellow oil (25 mg, 39%). LC-MS data – Ret. time 0.59: MS ES+ *m/z* 259 (50%, $[M+H]^+$); MS ES- *m/z* 257 (100%, $[M-H]^-$); ^{1}H NMR (400 MHz, $CDCl_3$) δ 7.53-7.40 (m, 4H, Ar-*H*), 7.02 (br s, 1H, N*H*).

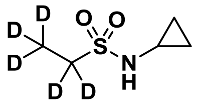

B8

Product obtained as a white solid (18 mg, 47%). LC-MS data – Ret. time 0.34: MS ES+ *m/z* 155 (100%, $[M+H]^+$); ^{1}H NMR (400 MHz, $CDCl_3$) δ 4.70 (br s, 1H, N*H*), 2.65-2.51 (m, 1H, NHC*H*), 0.77-0.65 (m, 4H, CHCH_2).

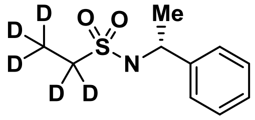

B9

Product obtained as an off-white solid (29 mg, 53%). LC-MS data – Ret. time 0.50: MS ES- *m/z* 217 (60%, $[M-H]^-$); ^{1}H NMR (400 MHz, $CDCl_3$) δ 7.43-7.29 (m, 5H, Ar-*H*), 4.80-4.57 (overlapping br s, 1H, N*H* and q, *J* 7.0, 1H, C*H*Me), 1.57 (d, *J* 7.0, 3H, CH*Me*).

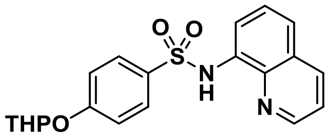

C1

Product obtained as a light brown oil (32 mg, 33%). LC-MS data – Ret. time 0.85: MS ES+ *m/z* 385 (100%, $[M+H]^+$), 407 (20%, $[M+Na]^+$); MS ES- *m/z* 383 (100%, $[M-H]^-$).

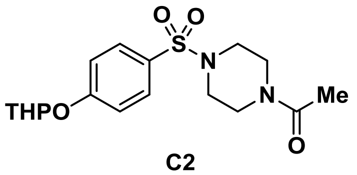

C2

Product obtained as a yellow solid (47 mg, 51%). LC-MS data – Ret. time 0.53: MS ES+ *m/z* 369 (95%, $[M+H]^+$), 391 (70%, $[M+Na]^+$).

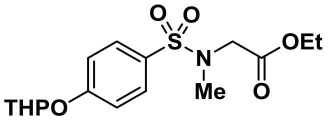

C3

Product obtained as a clear oil (46 mg, 51%). LC-MS data – Ret. time 0.74: MS ES+ *m/z* 358 (90%, $[M+H]^+$), 380 (95%, $[M+Na]^+$).

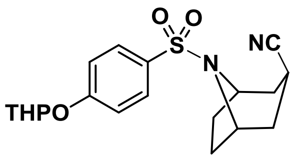

C4

Product obtained as a yellow solid (46 mg, 49%). LC-MS data – Ret. time 0.71: MS ES+ *m/z* 377 (90%, $[M+H]^+$), 399 (100%, $[M+Na]^+$).

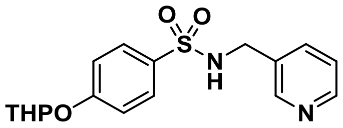

C5

Product obtained as an off-white solid (45 mg, 52%). LC-MS data – Ret. time 0.37: MS ES+ *m/z* 349 (100%, $[M+H]^+$), 371 (10%, $[M+Na]^+$); MS ES- *m/z* 347 (100%, $[M-H]^-$).

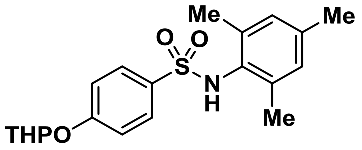

C6

Product obtained as an off-white solid (41 mg, 44%). LC-MS data – Ret. time 0.85: MS ES+ *m/z* 376 (25%, $[M+H]^+$), 398 (30%, $[M+Na]^+$); MS ES- *m/z* 374 (100%, $[M-H]^-$).

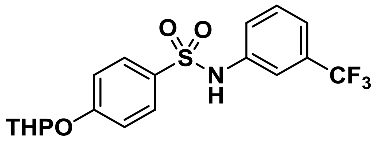

C7

Product obtained as an off-white solid (42 mg, 42%). LC-MS data – Ret. time 0.82: MS ES- *m/z* 400 (100%, $[M-H]^-$).

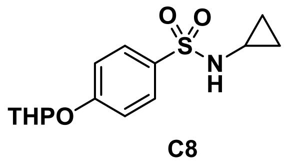

C8

Product obtained as an off-white solid (39 mg, 53%). LC-MS data – Ret. time 0.65: MS ES+ *m/z* 298 (60%, $[M+H]^+$), 320 (25%, $[M+Na]^+$); MS ES- *m/z* 296 (100%, $[M-H]^-$).

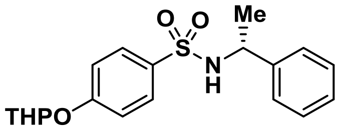

C9

Product obtained as an off-white solid (49 mg, 54%). LC-MS data – Ret. time 0.77: MS ES+ *m/z* 384 (100%, $[M+Na]^+$); MS ES- *m/z* 360 (100%, $[M-H]^-$).

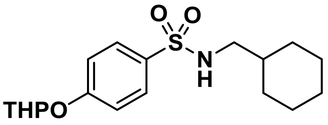

C10

Product obtained as a white solid (52 mg, 59%). LC-MS data – Ret. time 0.87: MS ES+ *m/z* 354 (90%, $[M+H]^+$), 376 (30%, $[M+Na]^+$); MS ES- *m/z* 352 (100%, $[M-H]^-$).

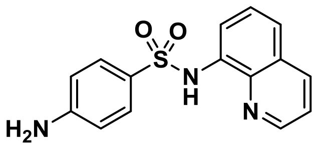

D1

Product obtained as an off-white solid (20 mg, 27%). LC-MS data – Ret. time 0.55: MS ES+ *m/z* 300 (100%, $[M+H]^+$); MS ES- *m/z* 298 (100%, $[M-H]^-$).

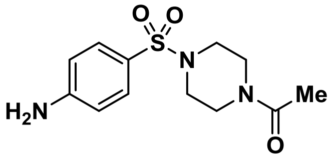

D2

Product obtained as a yellow oil (32 mg, 45%). LC-MS data – Ret. time 0.33: MS ES+ *m/z* 284 (100%, $[M+H]^+$), 306 (20%, $[M+Na]^+$); MS ES- *m/z* 282 (50%, $[M-H]^-$).

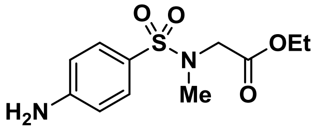

D3

Product obtained as an off-white solid (25 mg, 37%). LC-MS data – Ret. time 0.45: MS ES+ *m/z* 273 (100%, $[M+H]^+$), 295 (20%, $[M+Na]^+$).

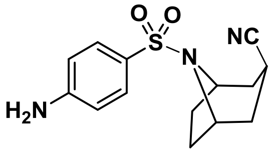

D4

Product obtained as an off-white solid (31 mg, 43%). LC-MS data – Ret. time 0.44: MS ES+ *m/z* 292 (100%, $[M+H]^+$), 314 (20%, $[M+Na]^+$); MS ES- *m/z* 290 (70%, $[M-H]^-$).

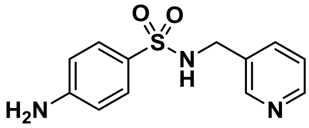

D5

Product obtained as a yellow oil (20 mg, 30%). LC-MS data – Ret. time 0.26: MS ES+ *m/z* 264 (100%, $[M+H]^+$); MS ES- *m/z* 262.0 (100%, $[M-H]^-$).

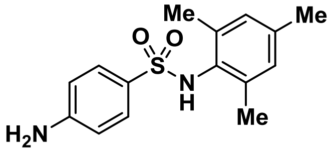

D6

Product obtained as an off-white solid (24 mg, 33%). LC-MS data – Ret. time 0.60: MS ES+ *m/z* 291 (80%, $[M+H]^+$), 313 (45%, $[M+Na]^+$); MS ES- *m/z* 289 (100%, $[M-H]^-$).

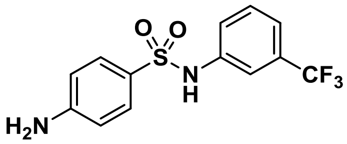

D7

Product obtained as an off-white solid (24 mg, 30%). LC-MS data – Ret. time 0.58: MS ES+ *m/z* 317 (100%, $[M+H]^+$); MS ES- *m/z* 315 (100%, $[M-H]^-$).

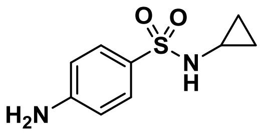

D8

Product obtained as a clear oil (22 mg, 42%). LC-MS data – Ret. time 0.38: MS ES+ *m/z* 213 (90%, $[M+H]^+$).

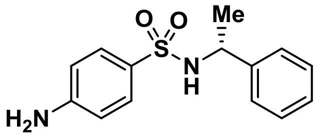

D9

Product obtained as an off-white solid (30 mg, 43%). LC-MS data – Ret. time 0.50: MS ES+ *m/z* 277 (100%, $[M+H]^+$); ES MS- *m/z* 275 (100%, $[M-H]^-$); ^{1}H NMR (400 MHz, $CDCl_3$) δ 7.30 (d, *J* 9.0, 2H, Ar-*H*), 7.12-7.08 (m, 5H, Ar-*H*), 6.47 (d, *J* 9.0, 2H, Ar-*H*), 4.20 (q, *J* 7.0, 1H, NHC*H*), 1.20 (d, *J* 7.0, 3H, CH*Me*).

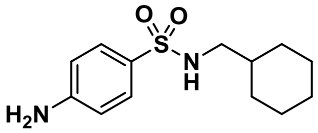

D10

Product obtained as an off-white solid (28 mg, 42%). LC-MS data – Ret. time 0.60: MS ES+ *m/z* 269 (100%, $[M+H]^+$), 291 (10%, $[M+Na]^+$); ES MS- *m/z* 267 (100%, $[M-H]^-$).

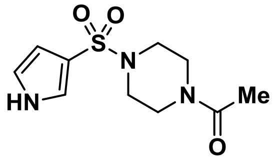

E2

Product obtained as an off-white solid (26 mg, 40%). LC-MS data – Ret. time 0.31: MS ES+ *m/z* 258 (100%, $[M+H]^+$), 280 (30%, $[M+Na]^+$); MS ES- *m/z* 256 (100%, $[M-H]^-$); ^{1}H NMR (400 MHz, CD_3OD) δ 7.32 (app t, *J* 2.0, 1H, Ar-*H*), 6.93 (dd, *J* 3.0, 2.0, 1H, Ar-*H*), 6.42 (dd, *J* 3.0, 2.0, 1H, Ar-*H*), 3.72-3.62 (m, 4H, N(COMe)CH_2), 3.03-2.91 (m, 4H, N(COMe)CH_2CH_2), 2.09 (s, 3H, CO*Me*).

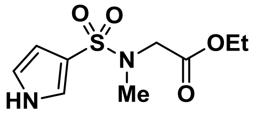

E3

Product obtained as an off-white solid (20 mg, 33%). LC-MS data – Ret. time 0.39: MS ES+ *m/z* 247 (100%, $[M+H]^+$), 269 (40%, $[M+Na]^+$); ES MS- *m/z* 245 (100%, $[M-H]^-$).

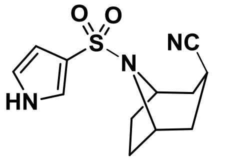

E4

Product obtained as an off-white solid (29 mg, 44%). LC-MS data – Ret. time 0.39: MS ES+ *m/z* 266 (100%, $[M+H]^+$), 288 (40%, $[M+Na]^+$); MS ES- *m/z* 264 (100%, $[M-H]^-$).

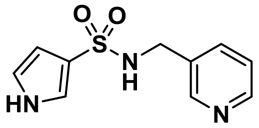

E5

Product obtained as a white solid (19 mg, 32%). LC-MS data – Ret. time 0.26: MS ES+ *m/z* 238 (100%, $[M+H]^+$); ES MS- *m/z* 236 (100%, $[M-H]^-$).

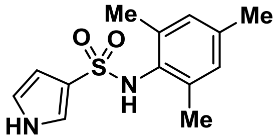

E6

Product obtained as an off-white solid (27 mg, 41%). LC-MS data – Ret. time 0.55: MS ES+ *m/z* 265 (10%, $[M+H]^+$), 287 (20%, $[M+Na]^+$); ES MS- *m/z* 263 (25%, $[M-H]^-$).

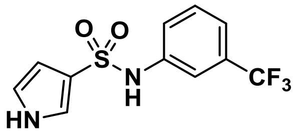

E7

Product obtained as an off-white solid (23 mg, 32%). LC-MS data – Ret. time 0.55: MS ES- *m/z* 289 (70%, $[M-H]^-$).

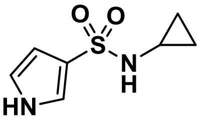

E8

Product obtained as an off-white solid (20 mg, 43%). LC-MS data – Ret. time 0.33: MS ES+ *m/z* 187 (30%, $[M+H]^+$), 209 (30%, $[M+Na]^+$); ^{1}H NMR (400 MHz, $CDCl_3$) δ 8.72 (br s, 1H, ArN*H*), 7.38-7.36 (m, 1H, Ar-*H*), 6.84 (dd, *J* 3.0, 2.5, 1H, Ar-*H*), 6.54-6.52 (m, 1H, Ar-*H*), 4.75 (br s, 1H, N*H*), 2.34-2.29 (m, 1H, NHC*H*), 0.68-0.63 (m, 2H, CHCH_2), 0.62-0.56 (m, 2H, CHCH_2).

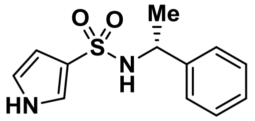

E9

Product obtained as a yellow oil (24 mg, 38%). LC-MS data – Ret. time 0.47: MS ES+ *m/z* 273 (90%, $[M+Na]^+$); MS ES- *m/z* 249 (100%, $[M-H]^-$); ^{1}H NMR (400 MHz, $CDCl_3$) δ 8.46 (br s, ArN*H*), 7.21-7.09 (overlapping m, 6H, Ar-*H*), 6.70 (dd, *J* 4.5, 2.0, 1H, Ar-*H*), 6.33-6.31 (m, 1H, Ar-*H*), 4.56 (br s, 1H, N*H*), 4.43-4.35 (m, 1H, NHC*H*), 1.40 (d, *J* 7.0, 3H, CH*Me*).

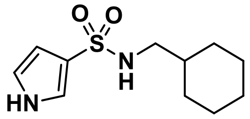

E10

Product obtained as an off-white solid (25 mg, 41%). LC-MS data – Ret. time 0.55: MS ES+ *m/z* 243 (100%, $[M+H]^+$), 265 (40%, $[M+Na]^+$); MS ES- *m/z* 241 (100%, $[M-H]^-$); ^1H NMR (400 MHz, $CDCl_3$) δ 8.73 (br s, 1H, ArN*H*), 7.24-7.22 (m, 1H, Ar-*H*), 6.76 (dd, *J* 5.0, 2.5, 1H, Ar-*H*), 6.43-6.41 (m, 1H, Ar-*H*), 4.27 (br s, 1H, N*H*), 2.71 (d, *J* 6.5, 2H, NHCH_2), 1.65-1.58 (m, 5H, CH_2), 1.42-1.32 (m, 1H, C*H*), 1.18-1.00 (m, 3H, CH_2), 0.86-0.75 (m, 2H, CH_2).

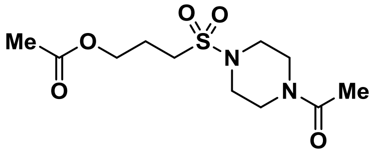

F2

Product obtained as an off-white solid (31 mg, 42%). LC-MS data – Ret. time 0.32: MS ES+ *m/z* 293 (100%, $[M+H]^+$), 315 (30%, $[M+Na]^+$); ^1H NMR (400 MHz, $CDCl_3$) δ 4.18 (t, *J* 6.0, 2H, OCH_2), 3.72 (t, *J* 5.0, 2H, N(COMe)CH_2), 3.57 (t, *J* 5.0, 2H, N(COMe)CH_2), 3.32 (t, *J* 5.0, 2H, N(COMe)CH_2CH_2), 3.28 (t, *J* 5.0, 2H, N(COMe)CH_2CH_2), 3.02-2.98 (m, 2H, SCH_2), 2.19-2.13 (m, 2H, OCH_2CH_2), 2.12 (s, 3H, NCO*Me*), 2.07 (s, 3H, OCO*Me*).

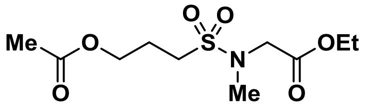

F3

Product obtained as a yellow oil (25 mg, 36%). LC-MS data – Ret. time 0.46: MS ES+ *m/z* 282 (50%, $[M+H]^+$), 304 (80%, $[M+Na]^+$).

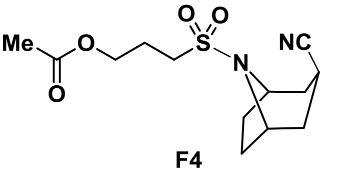

F4

Product obtained as a yellow oil (31 mg, 41%). LC-MS data – Ret. time 0.44: MS ES+ *m/z* 301 (75%, $[M+H]^+$), 323 (65%, $[M+Na]^+$).

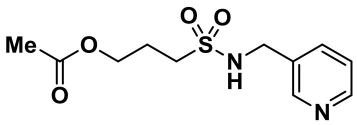

F5

Product obtained as a light brown oil (27 mg, 40%). LC-MS data – Ret. time 0.26: MS ES+ *m/z* 273 (100%, $[M+H]^+$), 295 (10%, $[M+Na]^+$).

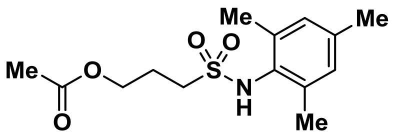

F6

Product obtained as an off-white solid (30 mg, 40%). LC-MS data – Ret. time 0.60: MS ES+ *m/z* 322 (75%, $[M+Na]^+$); MS ES- *m/z* 298 (60%, $[M-H]^-$); ^{1}H NMR (400 MHz, $CDCl_3$) δ 6.85 (s, 2H, Ar-*H*), 5.68 (br s, 1H, N*H*), 4.14 (t, *J* 6.0, 2H, OCH_2), 3.19-3.14 (m, 2H, SCH_2), 2.30 (s, 6H, Ar*Me* × 2), 2.23-2.15 (overlapping s, 3H, Ar*Me* and m, 2H, OCH_2CH_2), 2.00 (s, 3H, OCO*Me*).

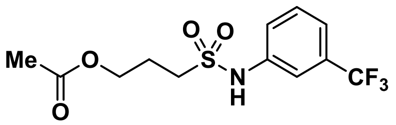

F7

Product obtained as a light brown oil (33 mg, 40%). LC-MS data – Ret. time 0.59: MS ES+ *m/z* 348 (80%, $[M+Na]^+$); MS ES- *m/z* 324 (70%, $[M-H]^-$); ^{1}H NMR (400 MHz, $CDCl_3$) δ 7.44-7.35 (m, 4H, Ar-*H*), 7.14 (br s, 1H, N*H*), 4.10 (t, *J* 6.0, 2H, OCH_2), 3.18-3.14 (m, 2H, SCH_2), 2.14-2.03 (m, 2H, OCH_2CH_2), 1.93 (s, 3H, OCO*Me*).

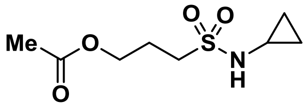

F8

Product obtained as a light yellow oil (28 mg, 51%). LC-MS data – Ret. time 0.37: MS ES+ *m/z* 222 (10%, $[M+H]^+$), 244 (80%, $[M+Na]^+$).

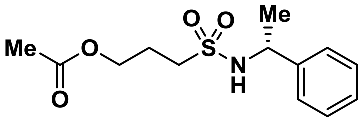

F9

Product obtained as an off-white solid (27 mg, 38%). LC-MS data – Ret. time 0.51: MS ES+ *m/z* 308 (100%, $[M+Na]^+$).

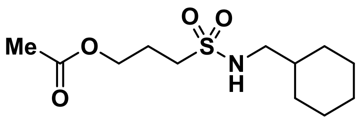

F10

Product obtained as an off-white solid (30 mg, 43%). LC-MS data – Ret. time 0.60: MS ES+ *m/z* 278 (75%, $[M+H]^+$), 300 (85%, $[M+Na]^+$).

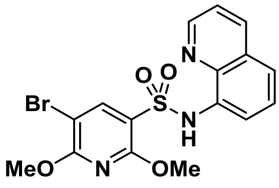

G1

Product obtained as an off-white solid (27 mg, 25%). LC-MS data – Ret. time 0.87: MS ES+ *m/z* 424 (75%, [M(^{79}Br)+H]$^+$), 426 (100%, [M(^{81}Br)+H]$^+$); MS ES- *m/z* 422 (85%, [M(^{79}Br)-H]$^-$), 424 (100%, [M(^{81}Br)-H]$^-$).

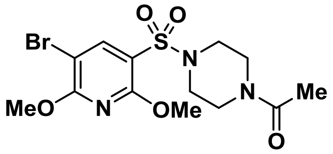

G2

Product obtained as an off-white solid (36 mg, 35%). LC-MS data – Ret. time 0.54: MS ES+ *m/z* 408 (75%, [M(^{79}Br)+H]$^+$), 410 (100%, [M(^{81}Br)+H]$^+$).

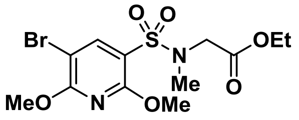

G3

Product obtained as a yellow oil (28 mg, 28%). LC-MS data – Ret. time 0.72: MS ES+ *m/z* 397 (75%, [M(^{79}Br)+H]$^+$), 399 (100%, [M(^{81}Br)+H]$^+$), 319 (50%, [M(^{79}Br)+Na]$^+$), 321 (60%, [M(^{81}Br)+Na]$^+$)

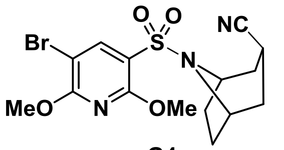

G4

Product obtained as a white solid (30 mg, 29%). LC-MS data – Ret. time 0.71: MS ES+ *m/z* 416 (75%, [M(^{79}Br)+H]$^+$), 418 (100%, [M(^{81}Br)+H]$^+$), 438 (40%, [M(^{79}Br)+Na]$^+$), 440 (50%, [M(^{81}Br)+Na]$^+$)

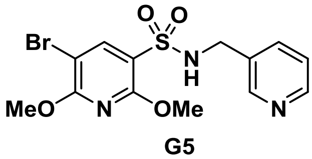

G5

Product obtained as an off-white solid (30 mg, 31%). LC-MS data – Ret. time 0.37: MS ES+ *m/z* 388 (75%, [M(^{79}Br)+H]$^+$), 390 (100%, [M(^{81}Br)+H]$^+$); MS ES- *m/z* 386 (80%, [M(^{79}Br)-H]$^-$), 388 (100%, [M(^{81}Br)-H]$^-$).

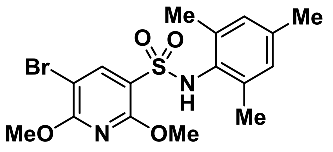

G6

Product obtained as an off-white solid (35 mg, 34%). LC-MS data – Ret. time 0.85: MS ES+ *m/z* 415 (10%, [M(^{79}Br)+H]$^{+}$), 417 (10%, [M(^{81}Br)+H]$^{+}$); MS MS ES- *m/z* 413 (80%, [M(^{79}Br)-H]$^{-}$), 415 (100%, [M(^{81}Br)-H]$^{-}$).

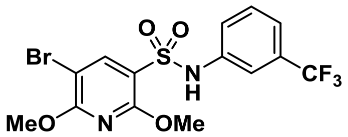

G7

Product obtained as an off-white solid (29 mg, 26%). LC-MS data – Ret. time 0.82: MS ES+ *m/z* 441 (25%, [M(^{79}Br)+H]$^{+}$), 443 (30%, [M(^{81}Br)+H]$^{+}$); MS ES- *m/z* 439 (80%, [M(^{79}Br)-H]$^{-}$), 441 (100%, [M(^{81}Br)-H]$^{-}$).

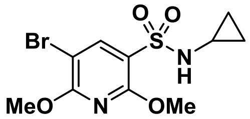

G8

Product obtained as an off-white solid (27 mg, 32%). LC-MS data – Ret. time 0.66: MS ES+ *m/z* 337 (65%, [M(^{79}Br)+H]$^{+}$), 339 (75%, [M(^{81}Br)+H]$^{+}$); MS ES- *m/z* 335 (95%, [M(^{79}Br)-H]$^{-}$), 337 (100%, [M(^{81}Br)-H]$^{-}$); ^{1}H NMR (400 MHz, $CDCl_3$) δ 8.23 (s, 1H, Ar-*H*), 5.20 (br s, 1H, N*H*), 4.04 (s, 3H, O*Me*), 4.00 (s, 3H, O*Me*), 2.07-2.00 (m, 1H, NHC*H*), 0.66-0.63 (m, 2H, CHC*H*$_2$), 0.55-0.51 (m, 2H, CHC*H*$_2$).

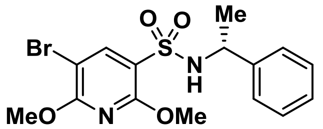

G9

Product obtained as an off-white solid (31 mg, 31%). LC-MS data – Ret. time 0.87: MS ES+ *m/z* 423 (25%, [M(^{79}Br)+Na^{+}]), 425 (30%, [M(^{81}Br)+Na]$^{+}$); MS ES- *m/z* 399 (80%, [M(^{79}Br)-H]$^{-}$), 401 (100%, [M(^{81}Br)-H]$^{-}$).

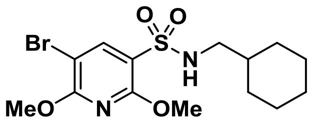

G10

Product obtained as an off-white solid (32 mg, 33%). LC-MS data – Ret. time 0.90: MS ES+ *m/z* 393 (75%, $[M(^{79}Br)+H]^+$), 395 (100%, $[M(^{81}Br)+H]^+$); MS ES- *m/z* 391 (85%, $[M(^{79}Br)-H]^-$), 393 (100%, $[M(^{81}Br)-H]^-$).

3. References

1) B. Nguyen, E. J. Emmett, M. C. Willis, *J. Am. Chem. Soc.* **2010**, *132*, 16372
2) K. Itami, K. Terakawa, J-I. Yoshida, O. Kajimoto, *J. Am. Chem. Soc.* **2003**, *125*, 6058
3) M. S. Cloonan, J. J. Keating, S. G. Butler, A. J. S. Knox, A. M. Jorgensen, G. H. Peters, D. Rai, D. Corrigan, D. G. Lloyd, C. Williams, M. J. Meegan, *Eur. J. Med. Chem.* **2009**, *44*, 4862
4) A. Lube, W. P. Neumann, M. Niestroj, *Chem. Ber.* **1995**, 128, 1195
5) H. Woolven, C. Gonzalez-Rodriguez, I. Marco, A. L. Thompson, M. C. Willis, *Org Lett.* **2011**, 13, 4876
6) J. R. DeBergh, N. Niljianskul, S. L. Buchwald, *J. Am. Chem. Soc.* **2013**, 135, 10638
7) E. L. Clennan, H. Zhang, *J. Am. Chem. Soc.* **1995**, 117, 4218
8) A. Krasovskiy, V. Malakhov, A. Gavryushin, P. Knochel, *Angew. Chem. Int. Ed.* **2006**, 45, 6040
9) R. J. Cremlyn, *Phosphorous, Sulfur Silicon Relat. Elem.* **1981**, 10, 111
10) X. Cui, F. Shi, M. K. Tse, D. Goerdes, K. Thurow, M. Beller, Y. Deng, *Adv. Synth. Catal.* **2009**, 351, 2949
11) G. Manolikakes, P. Knochel, *Angew. Chem. Int. Ed.* **2008**, 48, 205
12) E. Johansson, P. T. Hurley, B. S. Brunschwig, N. S. Lewis, *J. Phys. Chem. C* **2009**, 113, 15239
13) Y. Sasaki, A. Niida, T. Tsuji, A. Shigenaga, N. Fujii, A. Otaka, *J. Org. Chem.* **2006**, 71, 4969
14) S. Yamada, A. Gavryushin, P. Knochel, *Angew. Chem. Int. Ed.* **2010**, 122, 2261

4. NMR Spectra – General Scope

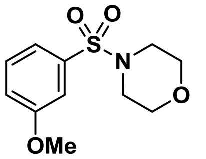

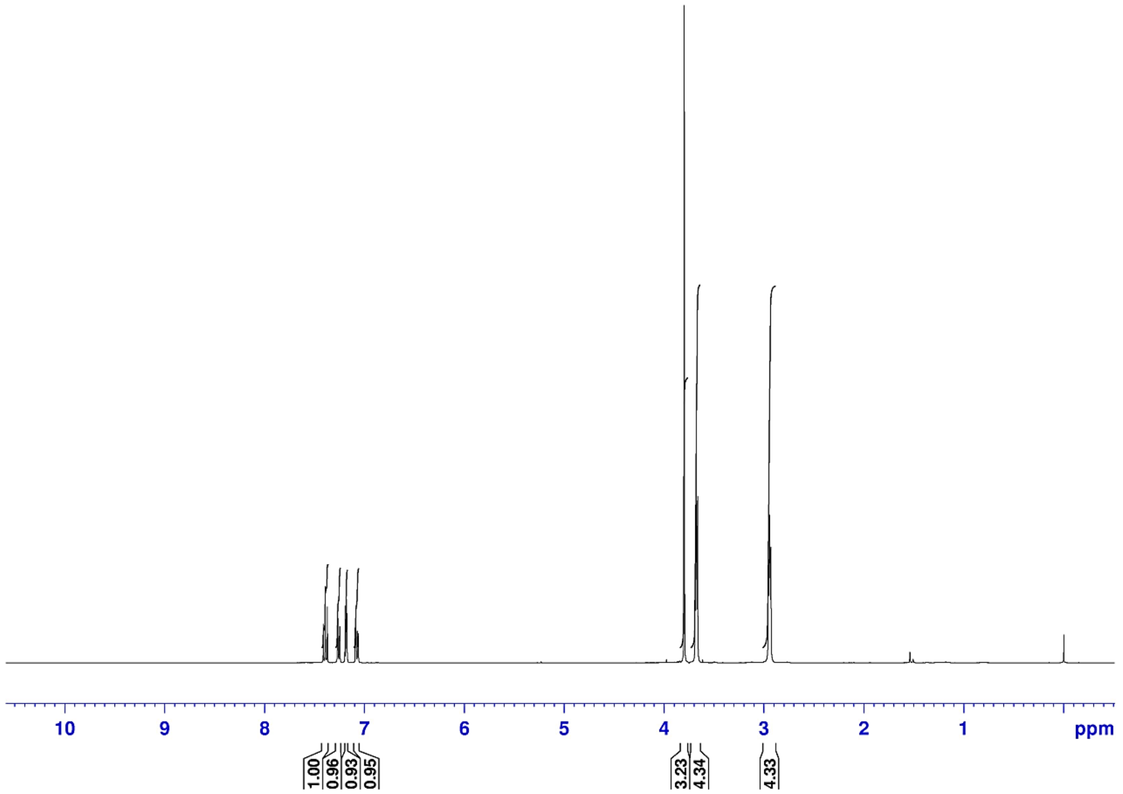

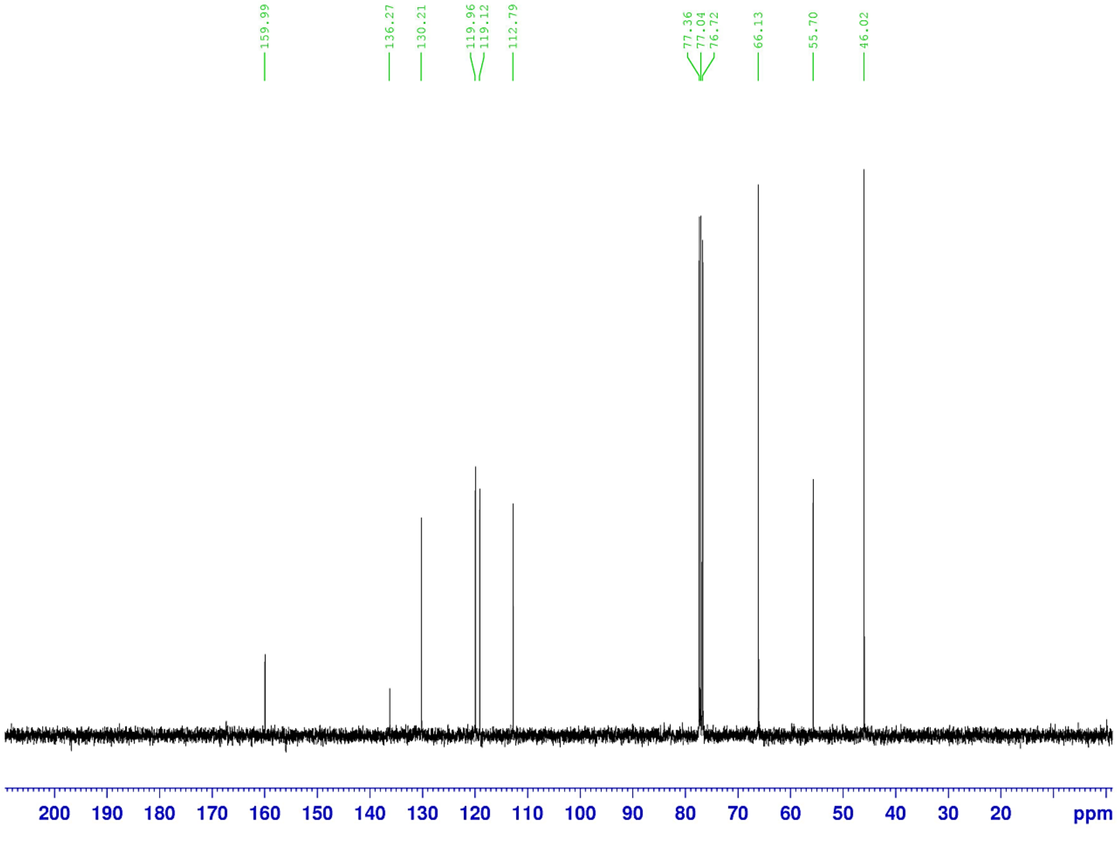

Entry 1, Table 2

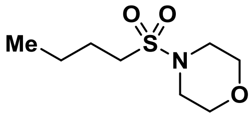

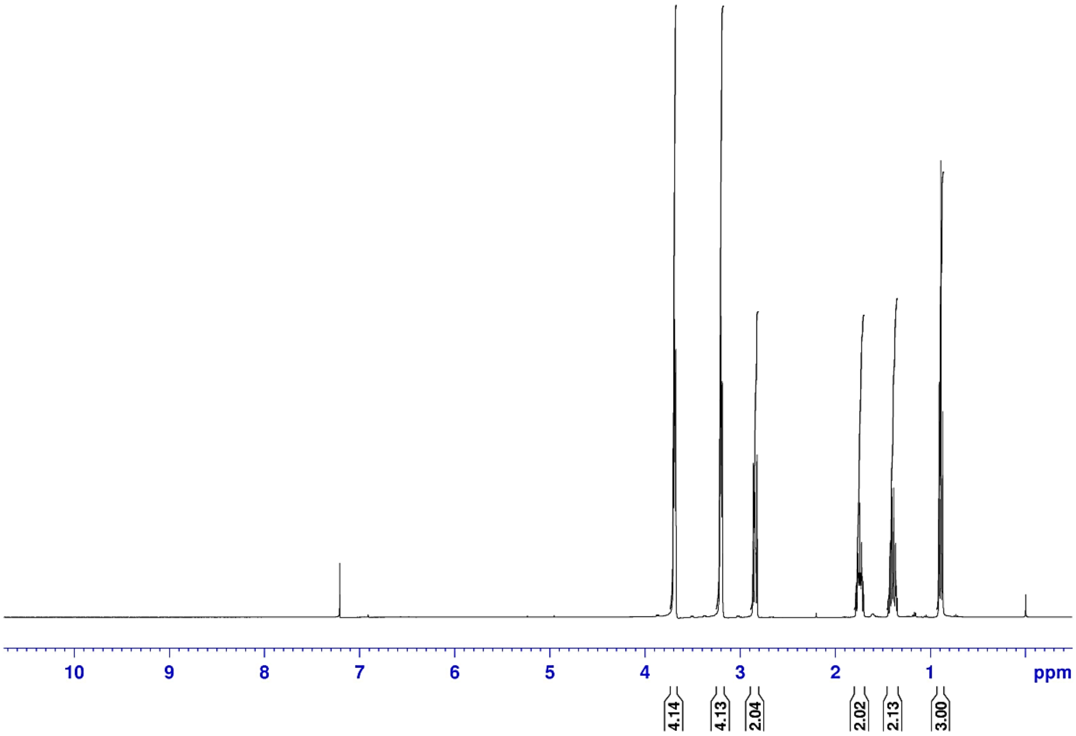

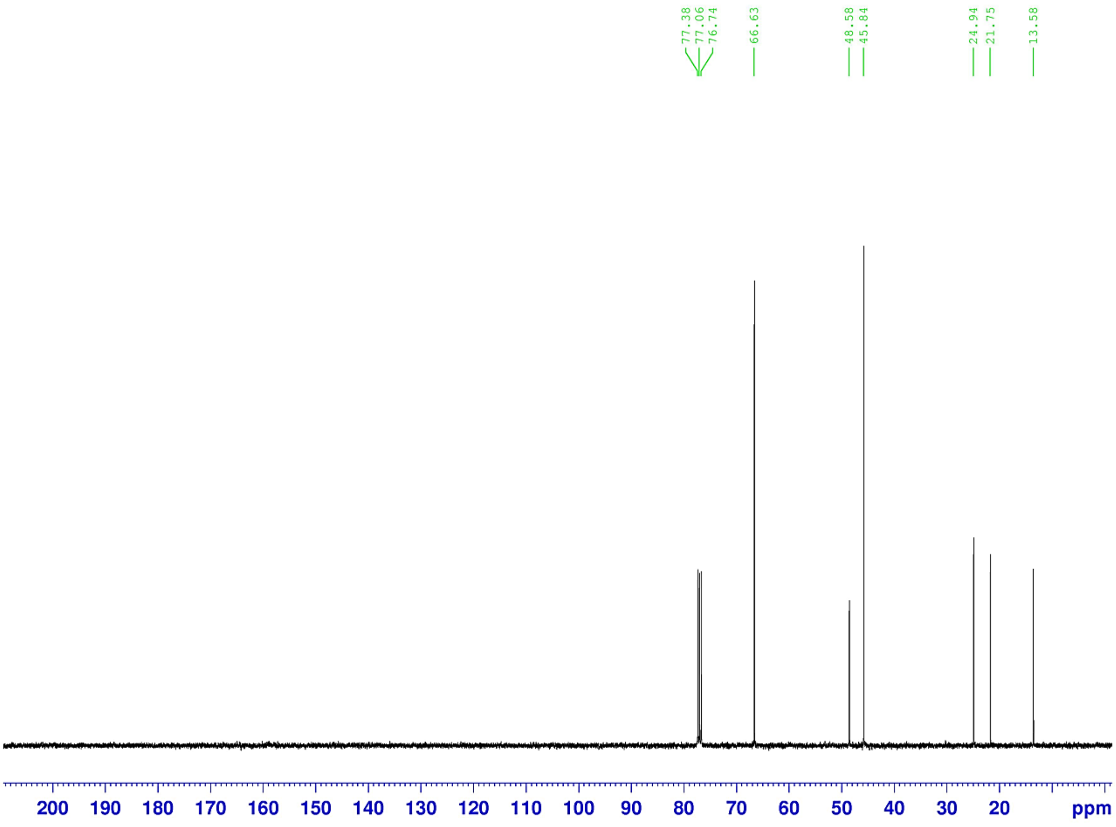

Entry 4, Table 2

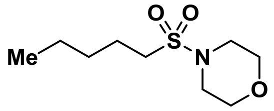

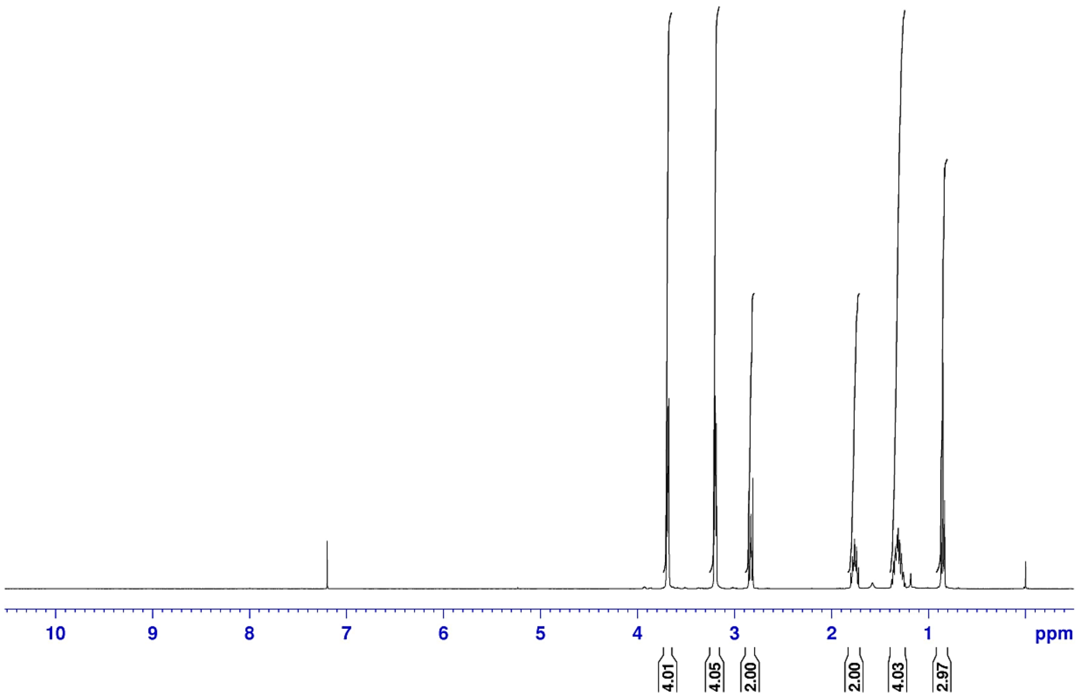

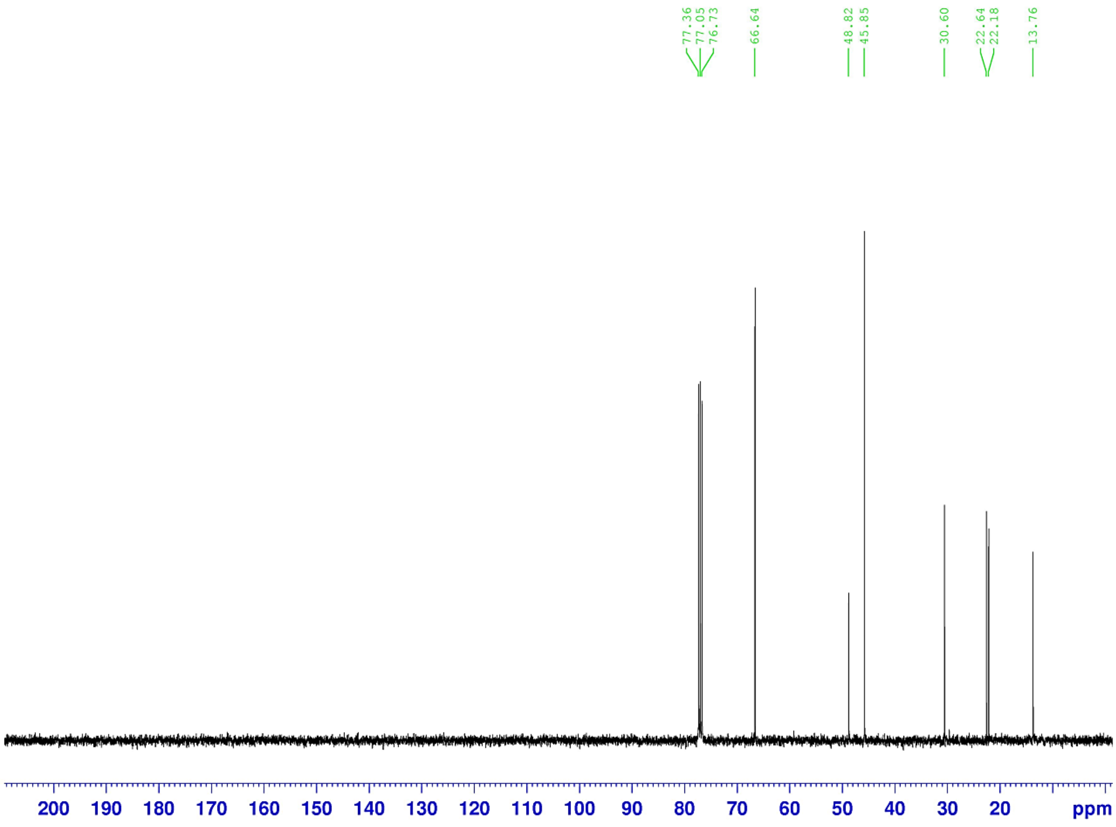

Entry 5, Table 2

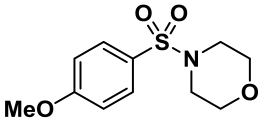

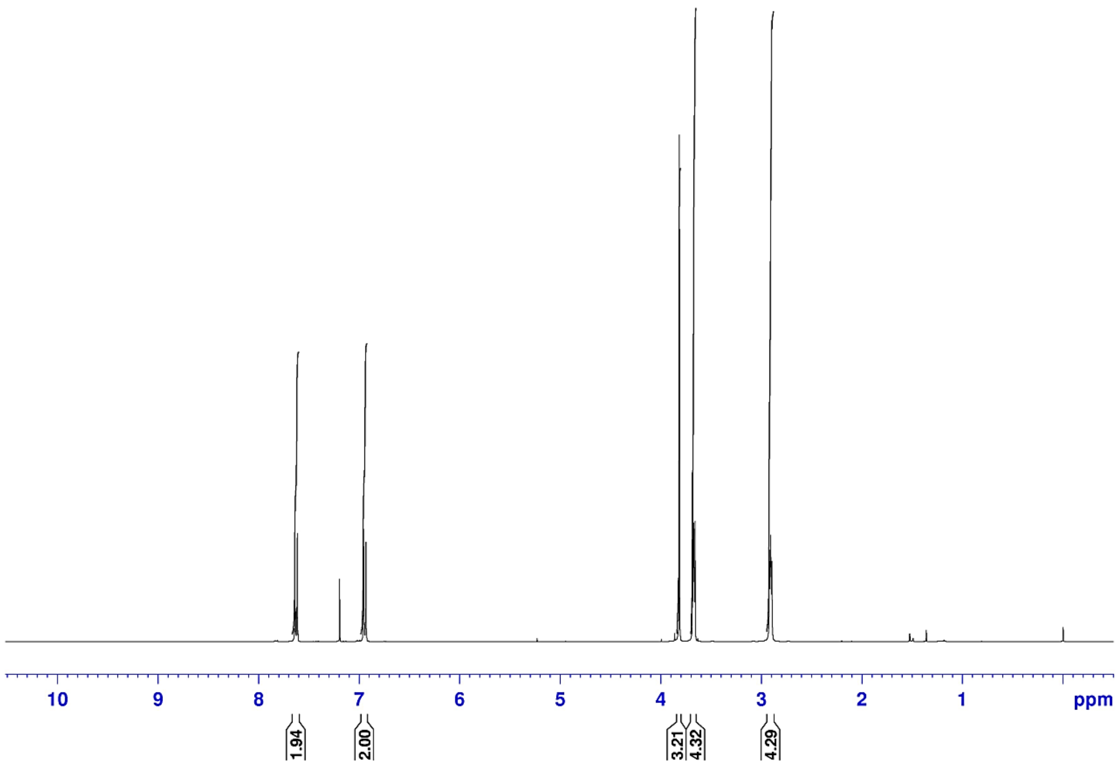

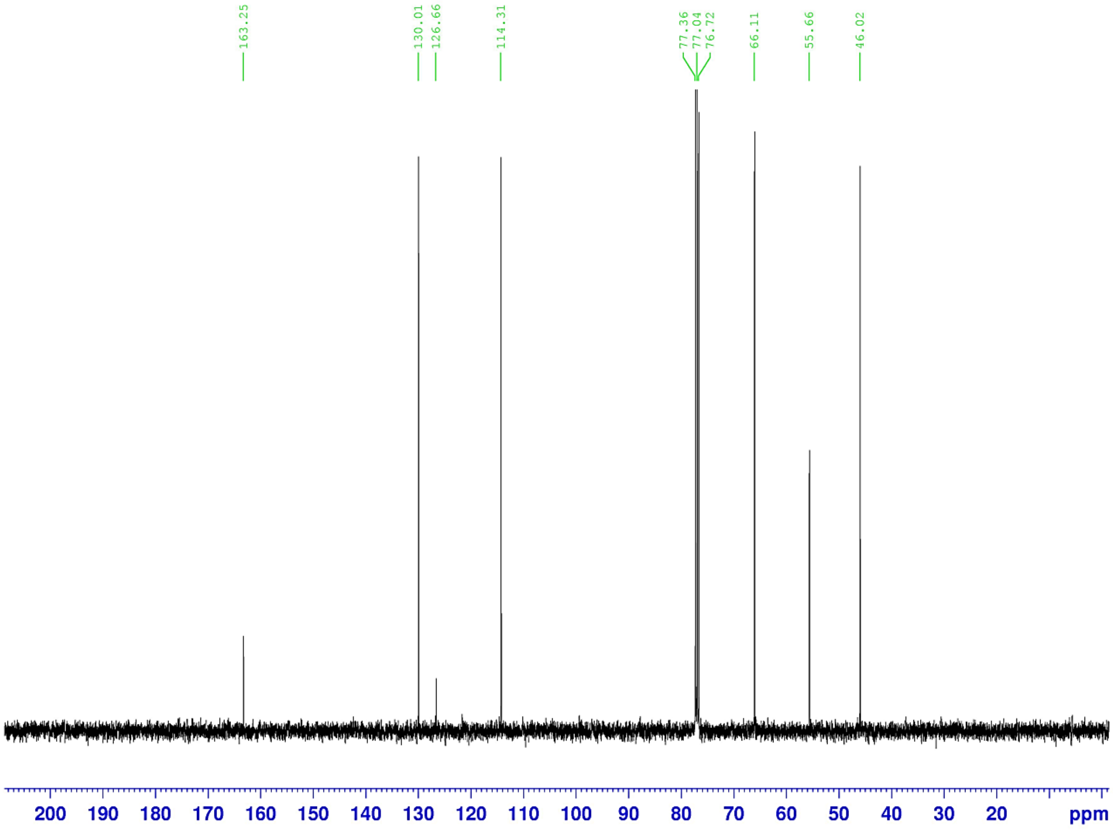

Entry 6, Table 2

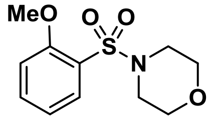

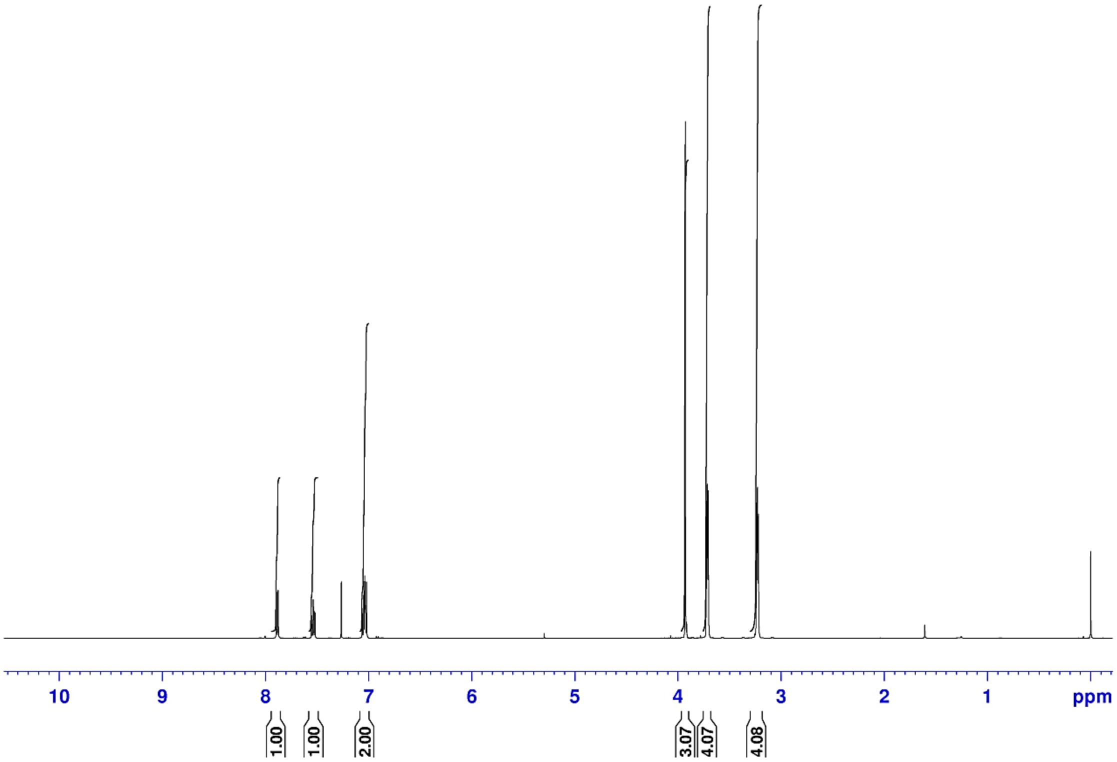

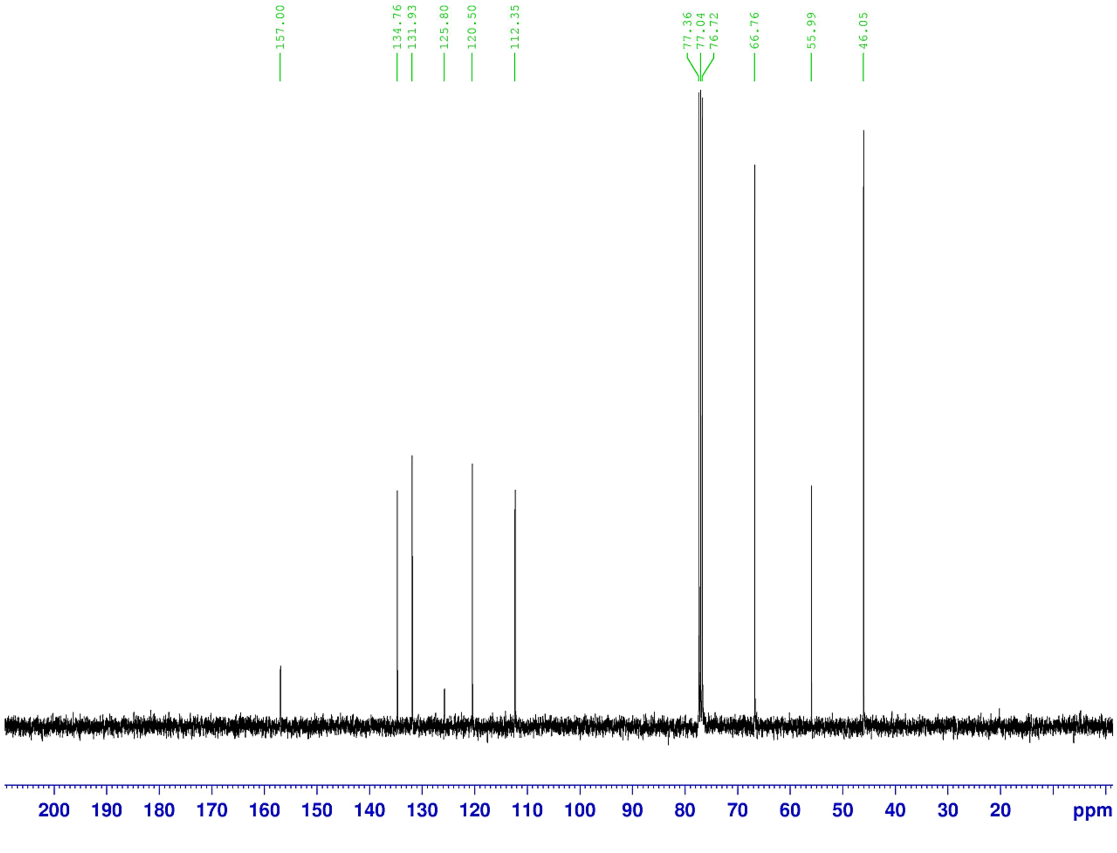

Entry 7, Table 2

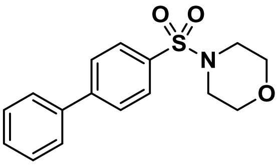

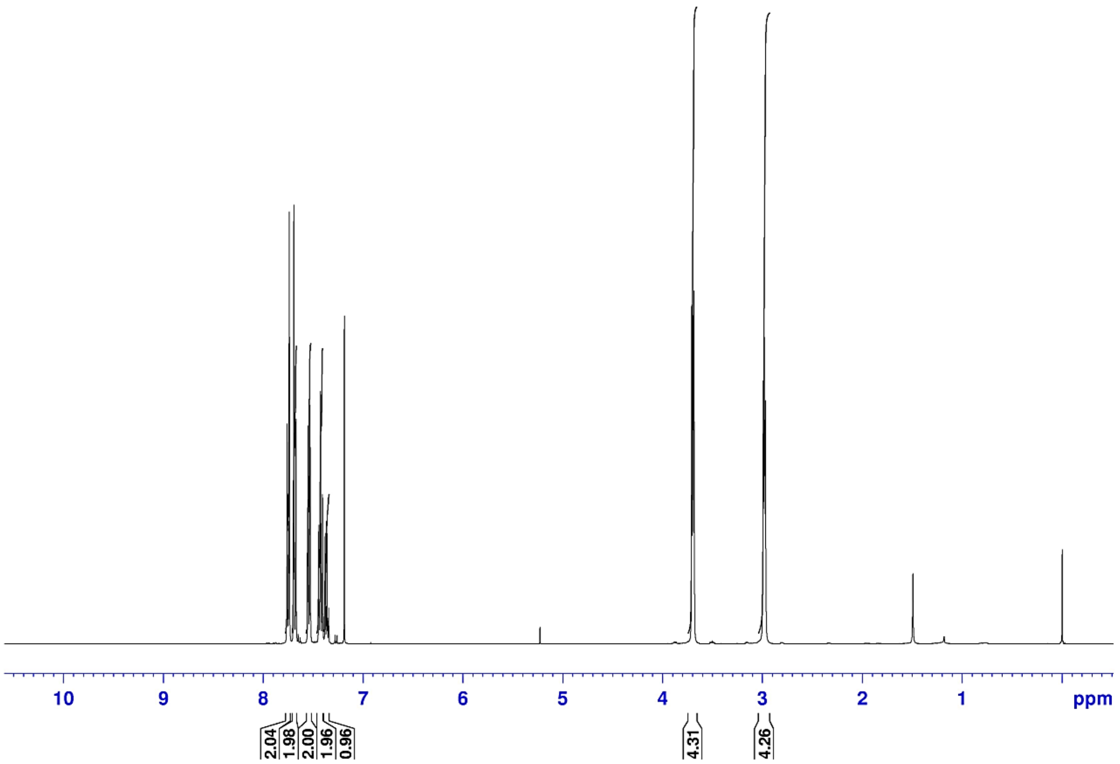

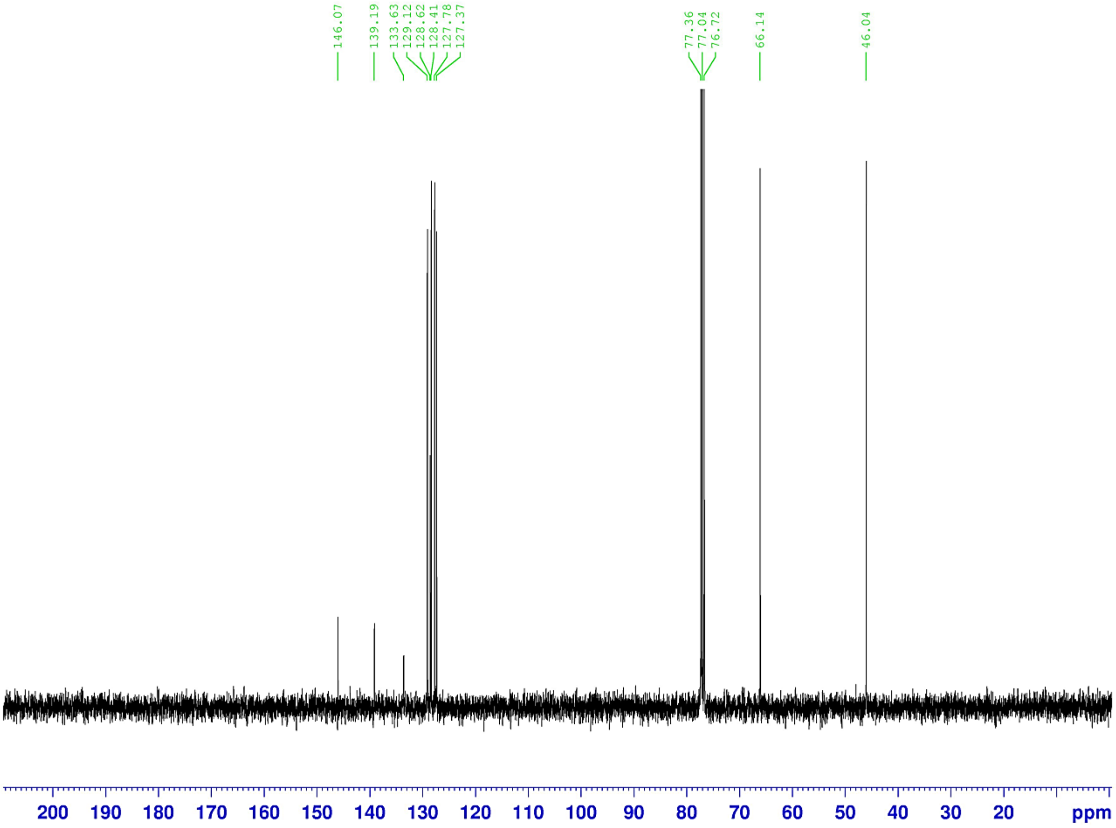

Entry 8, Table 2

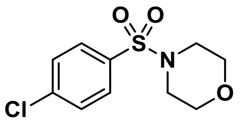

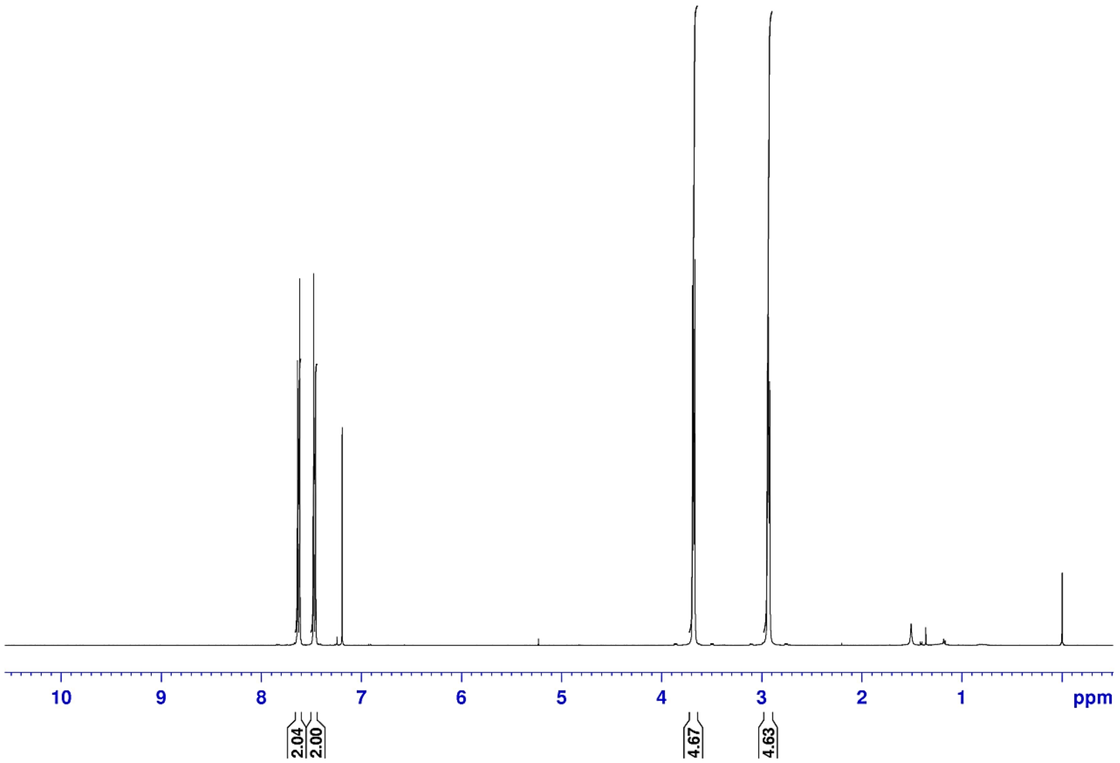

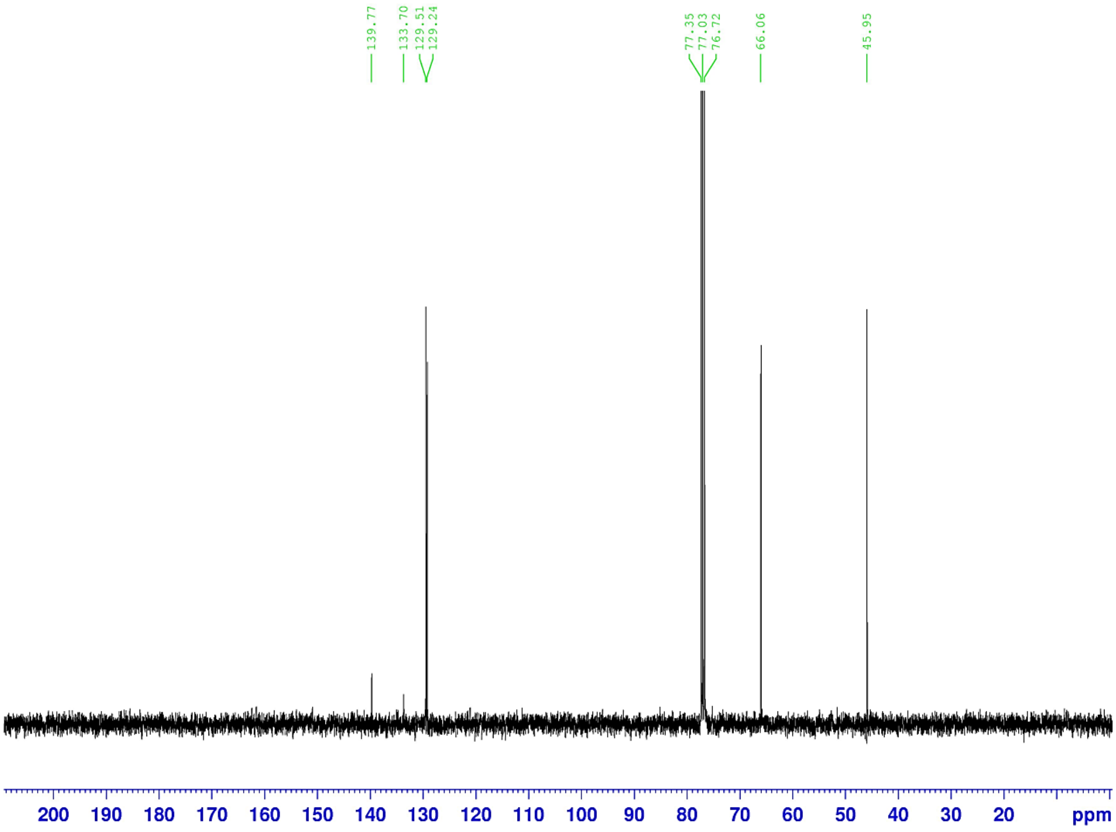

Entry 9, Table 2

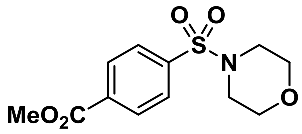

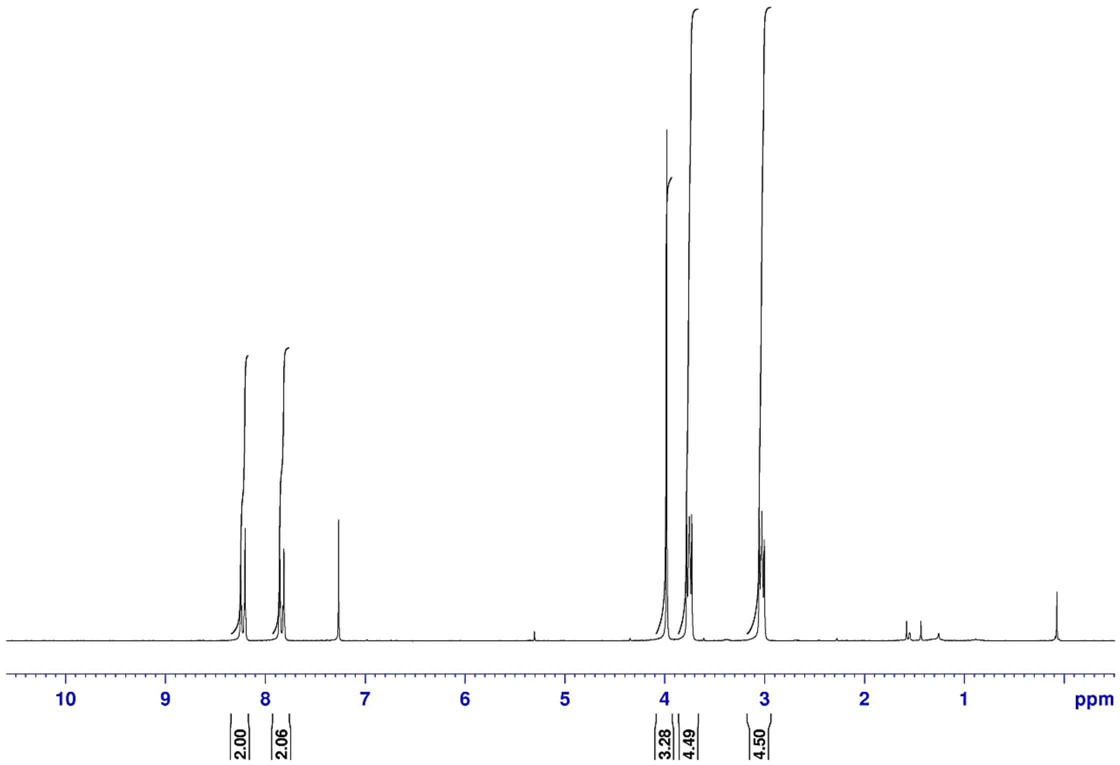

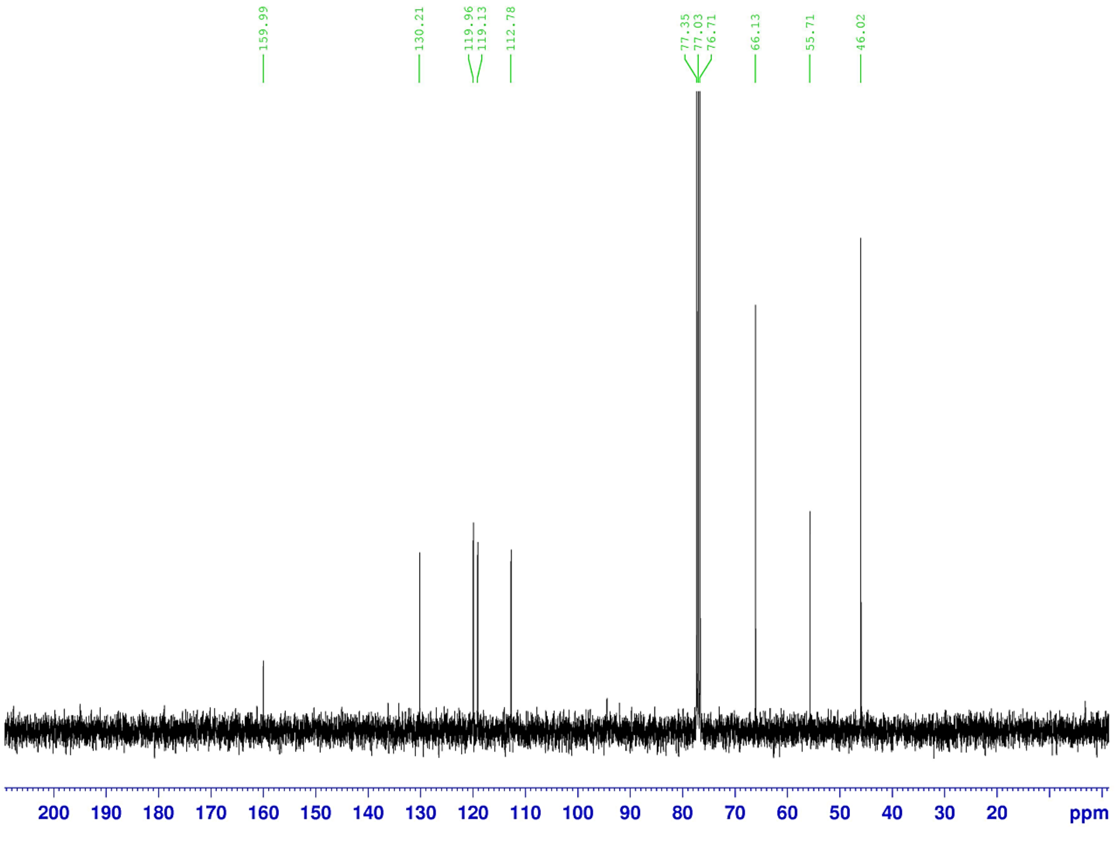

Entry 10, Table 2

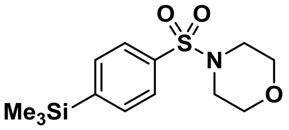

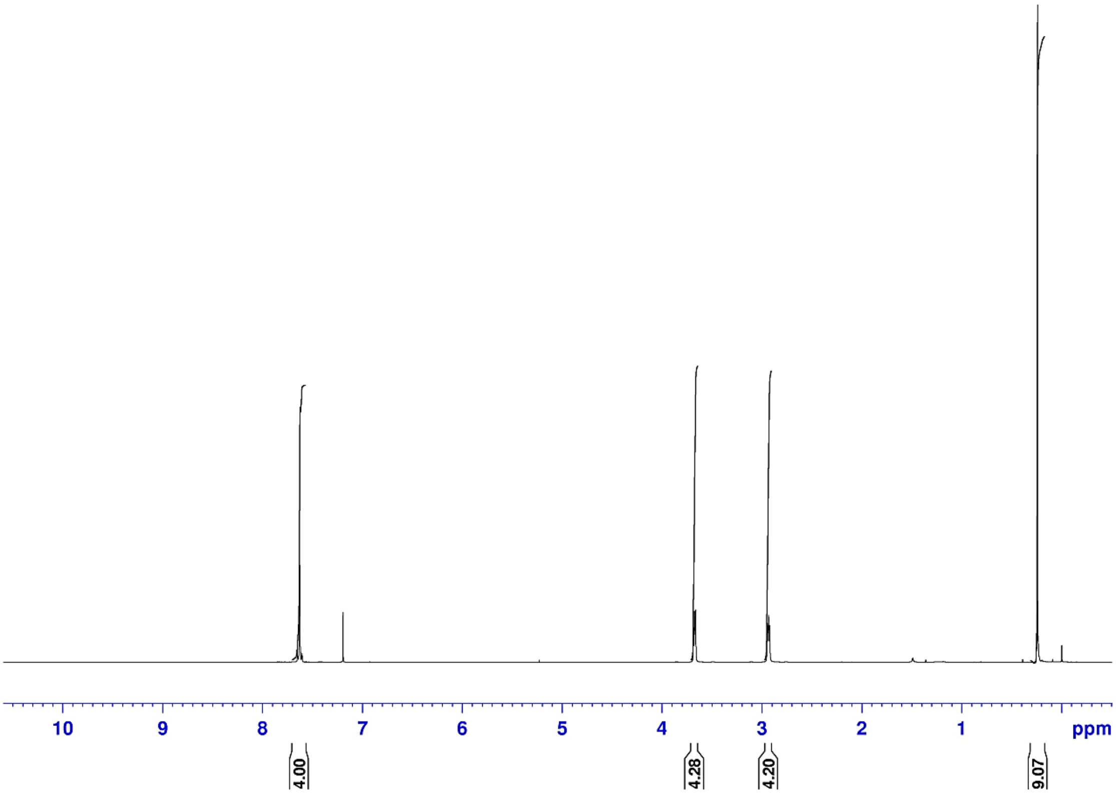

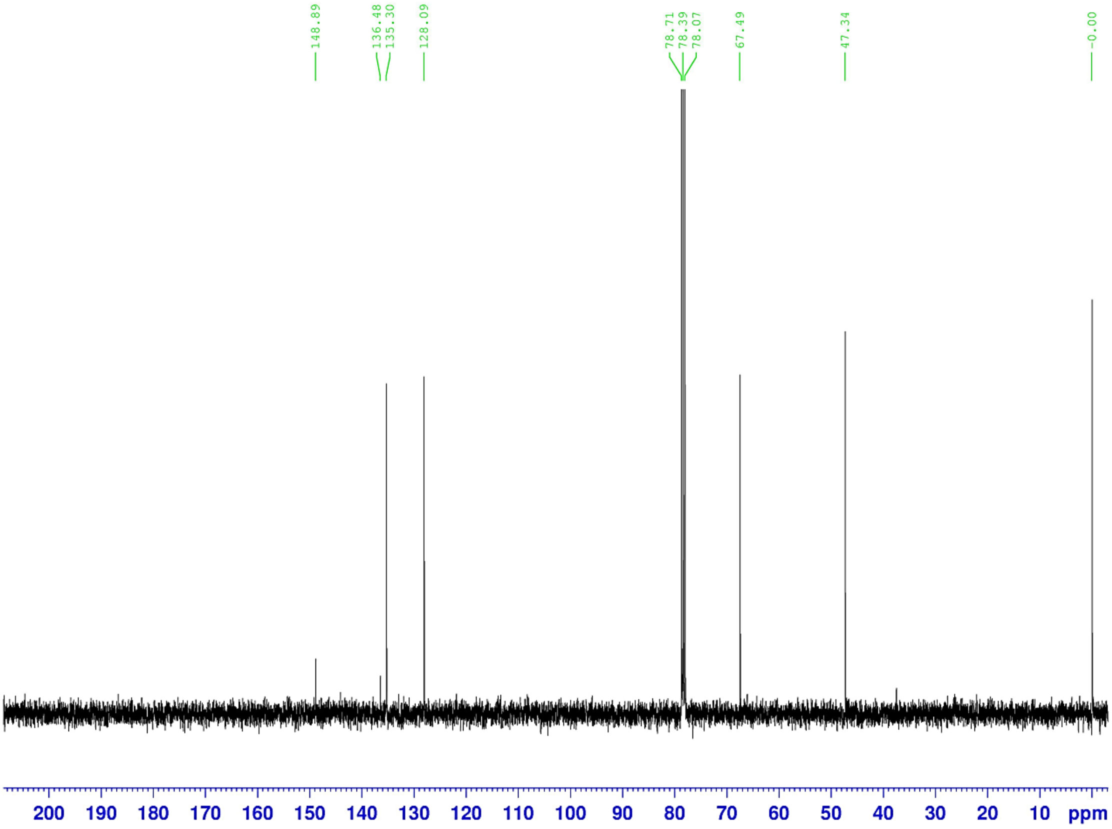

Entry 11, Table 2

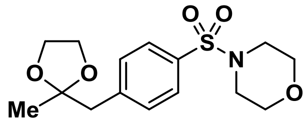

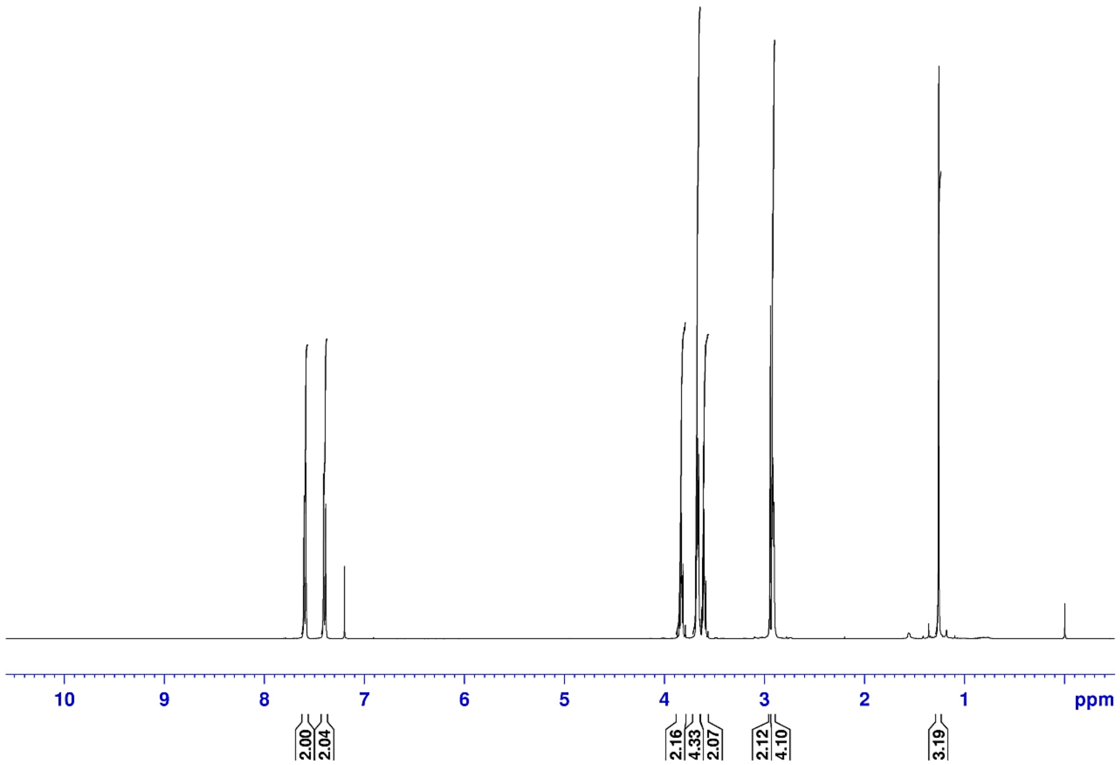

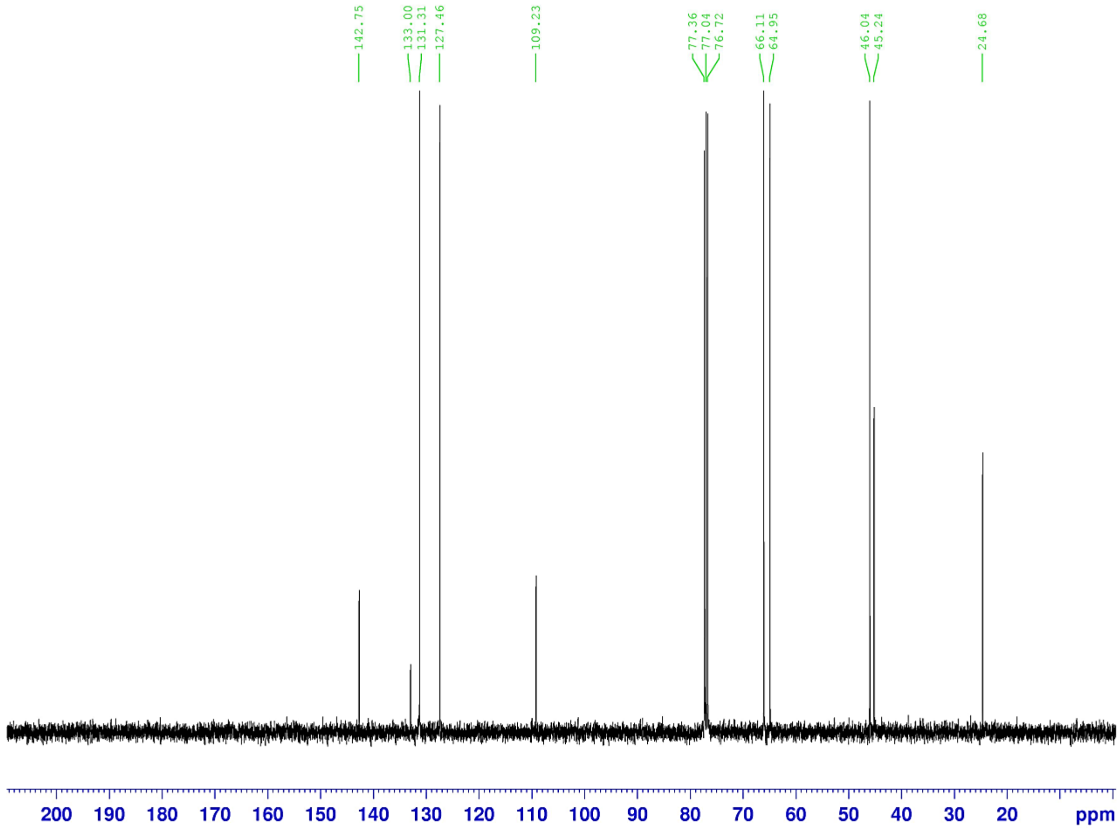

Entry 12, Table 2

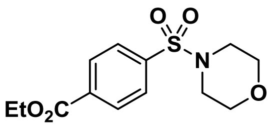

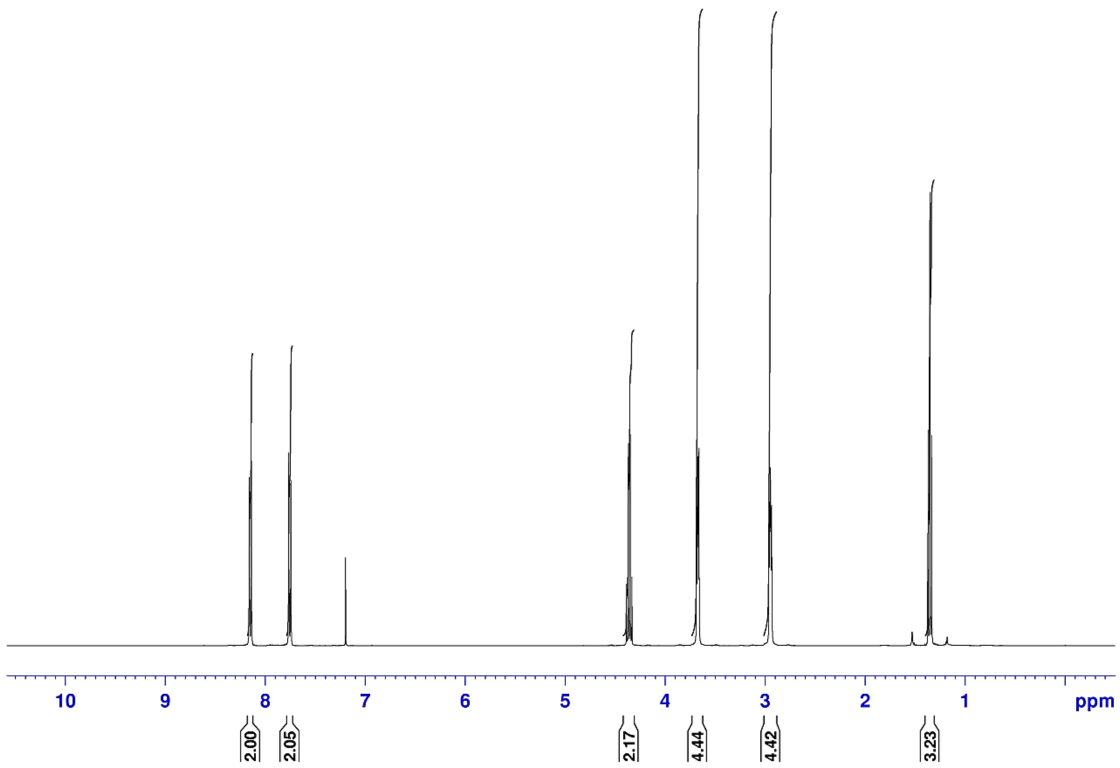

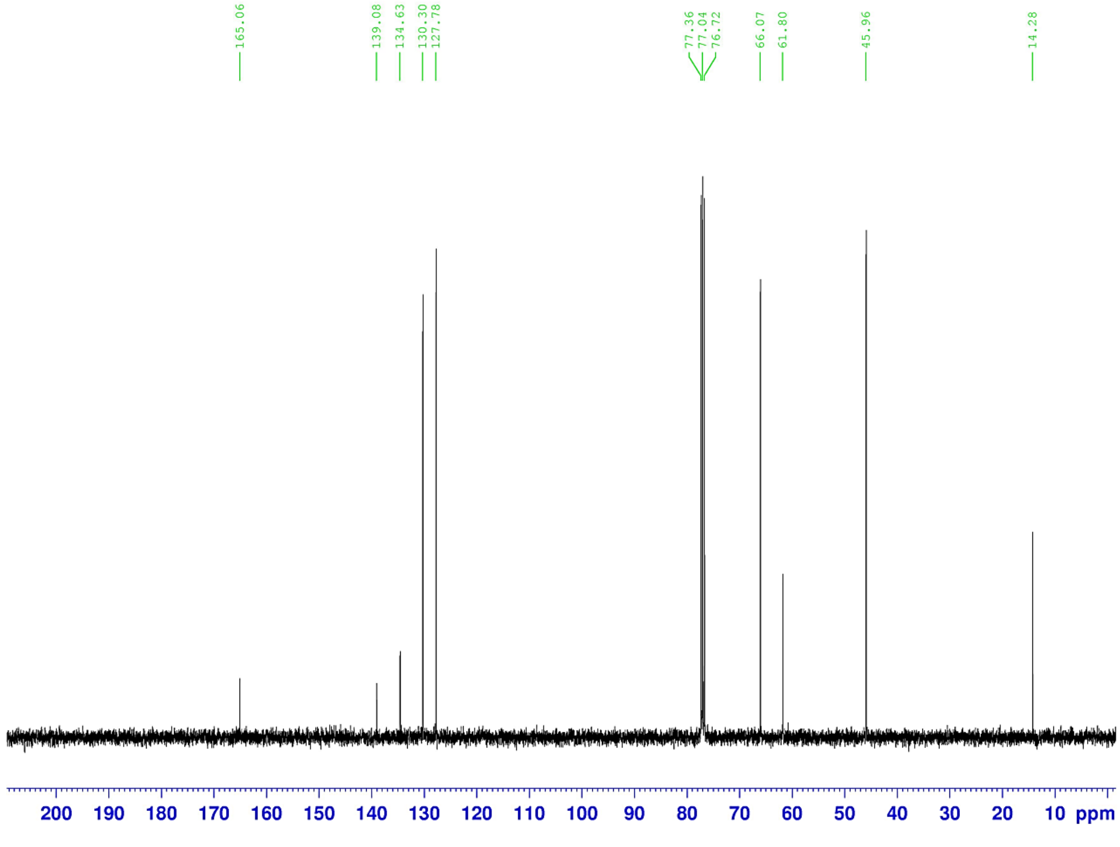

Entry 13, Table 2

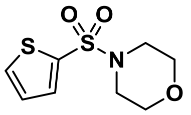

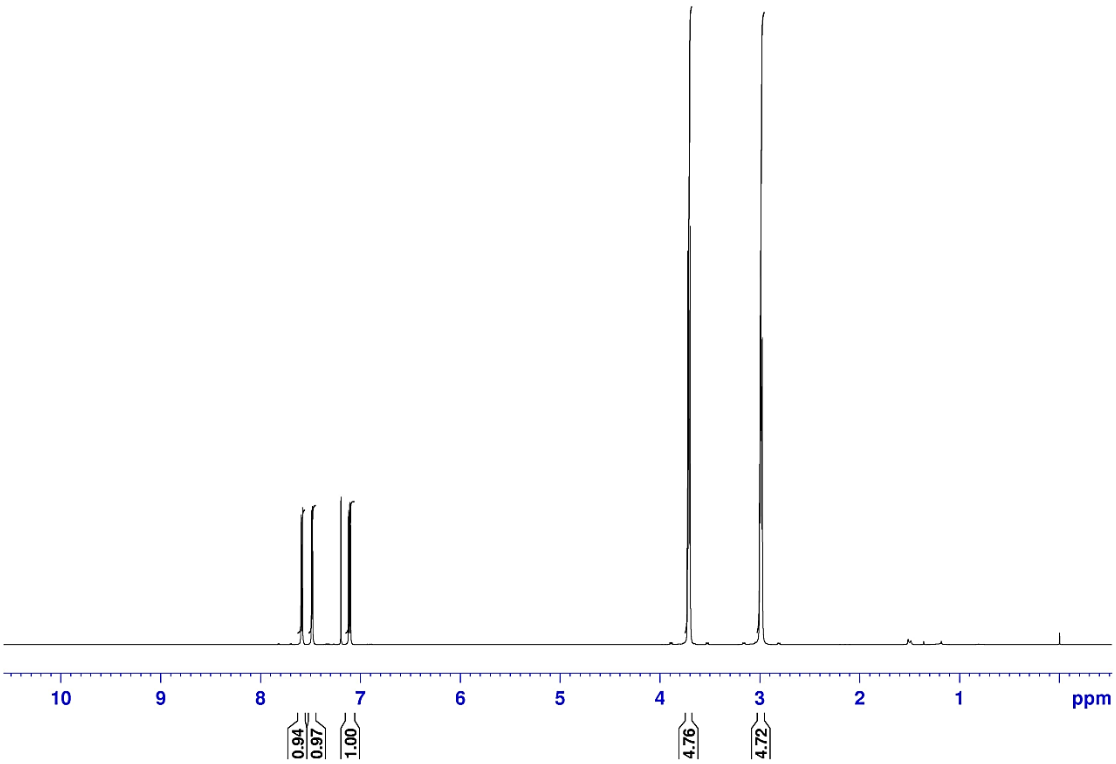

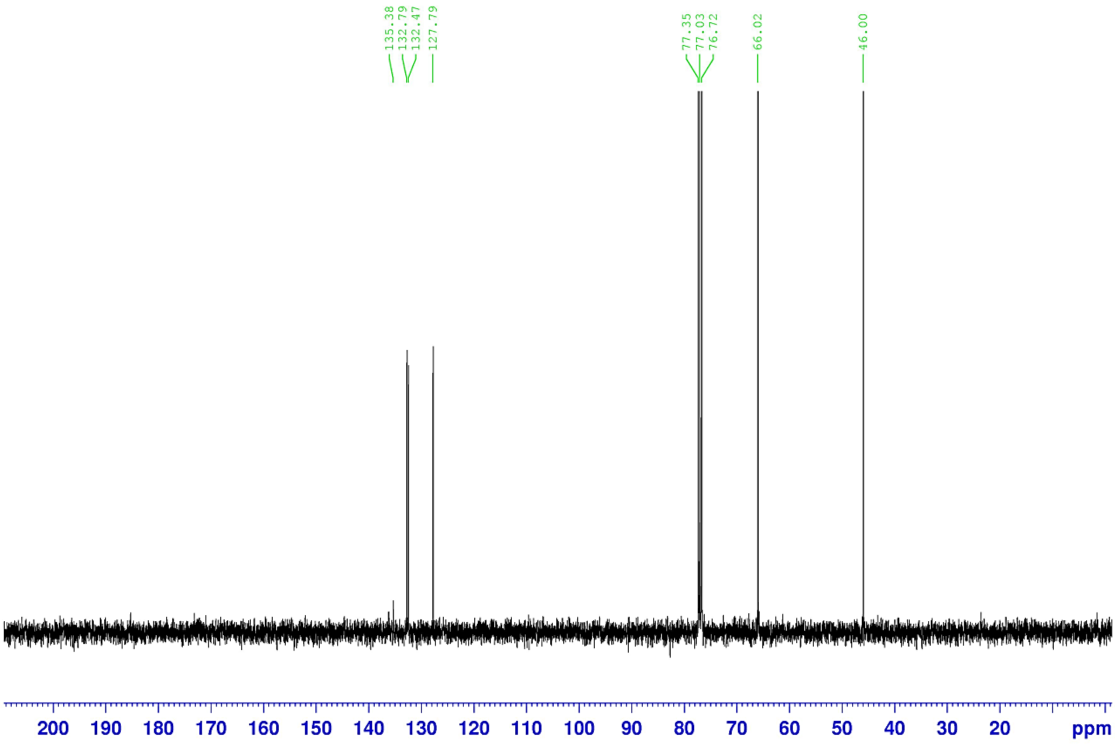

Entry 15, Table 2

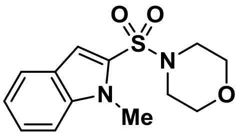

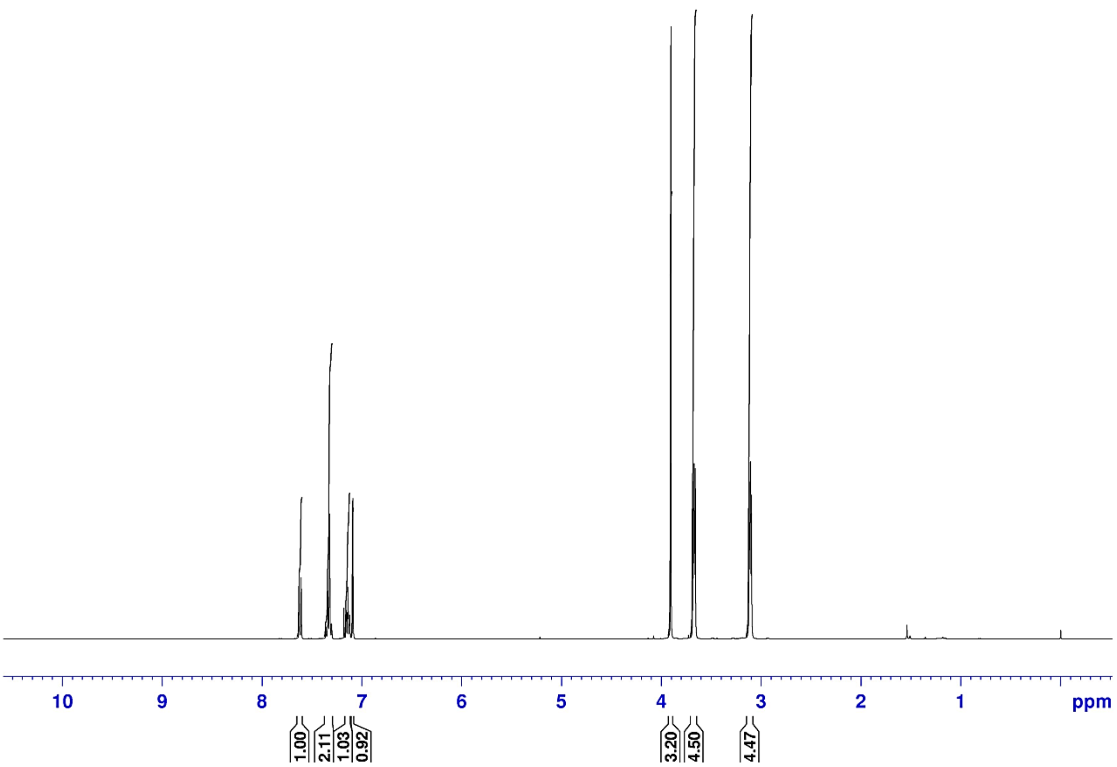

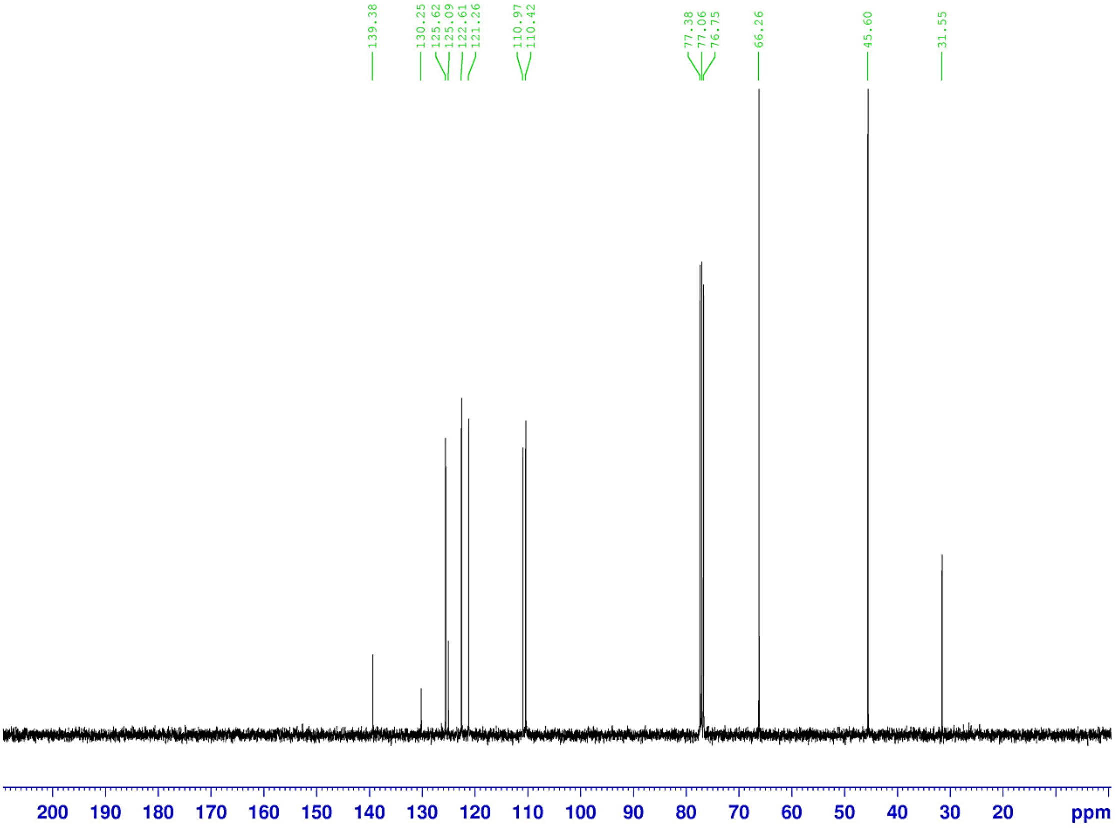

Entry 16, Table 2

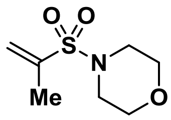

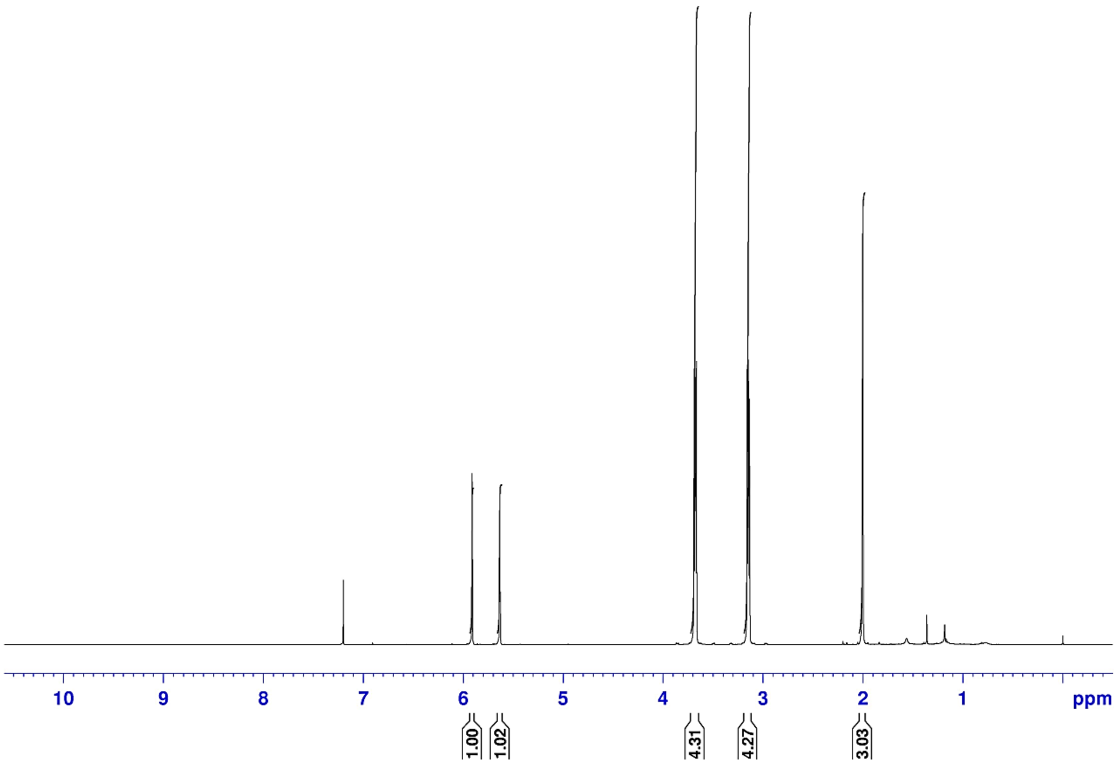

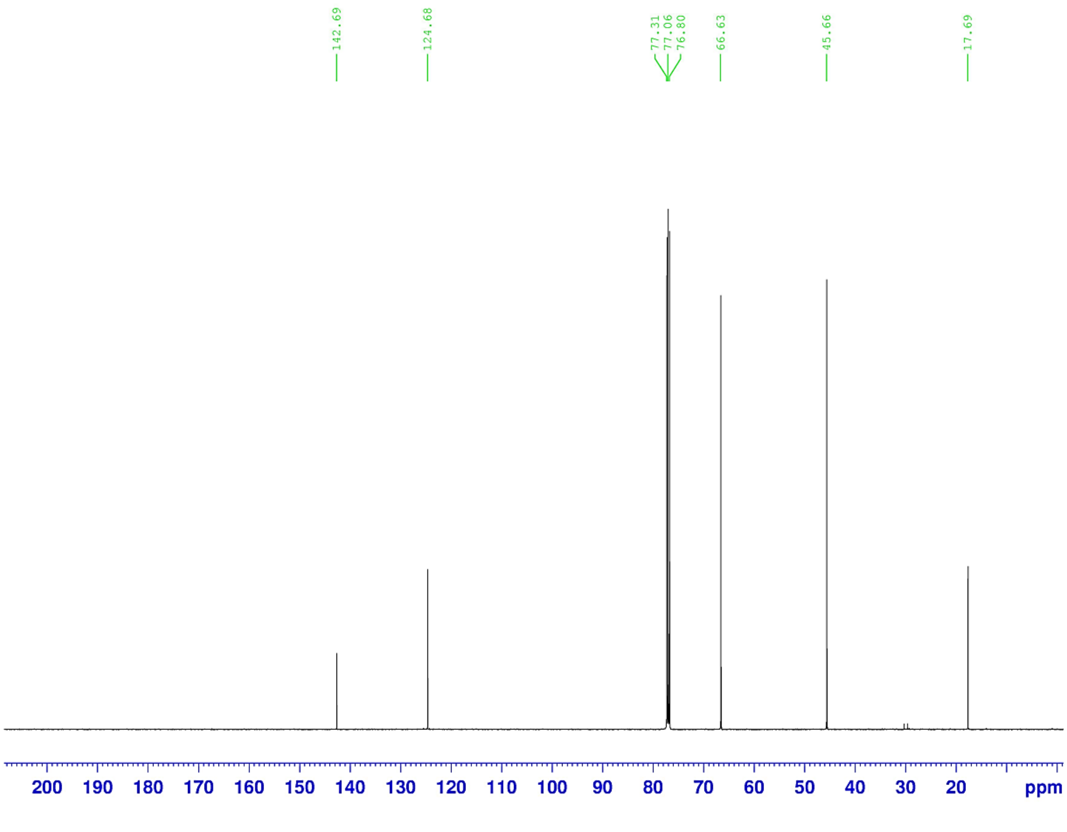

Entry 1, Table 3

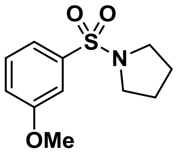

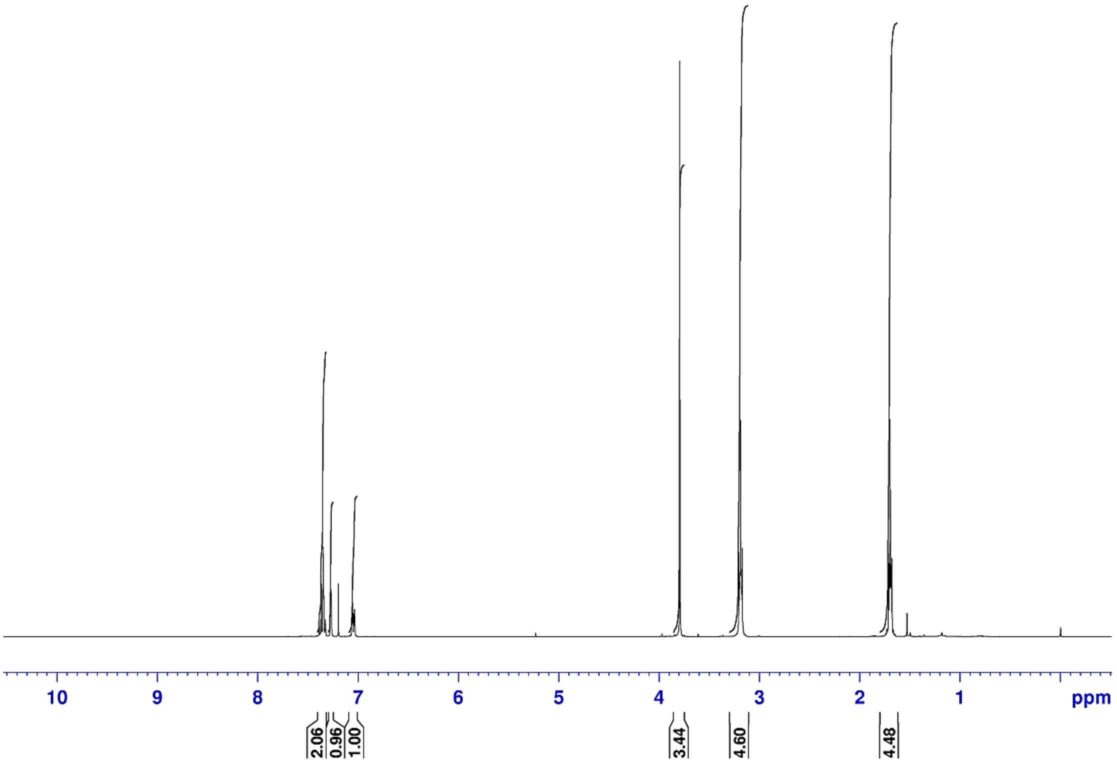

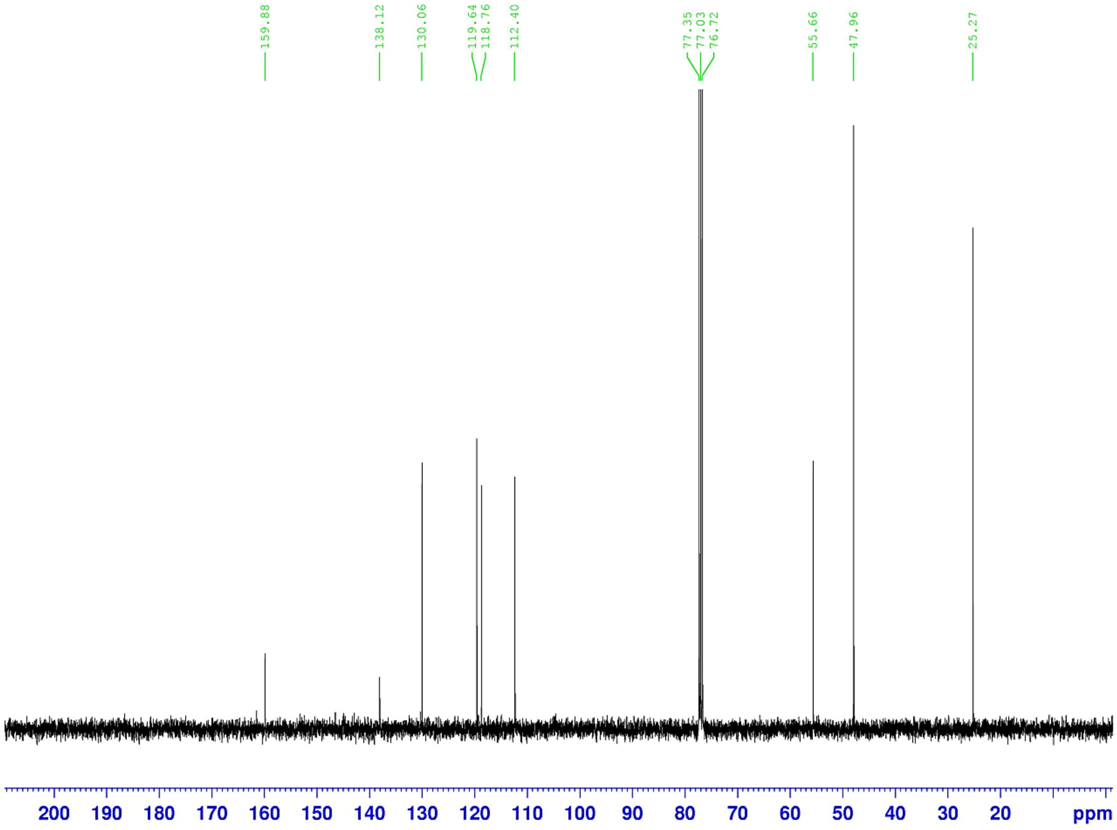

Entry 2, Table 3

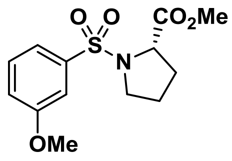

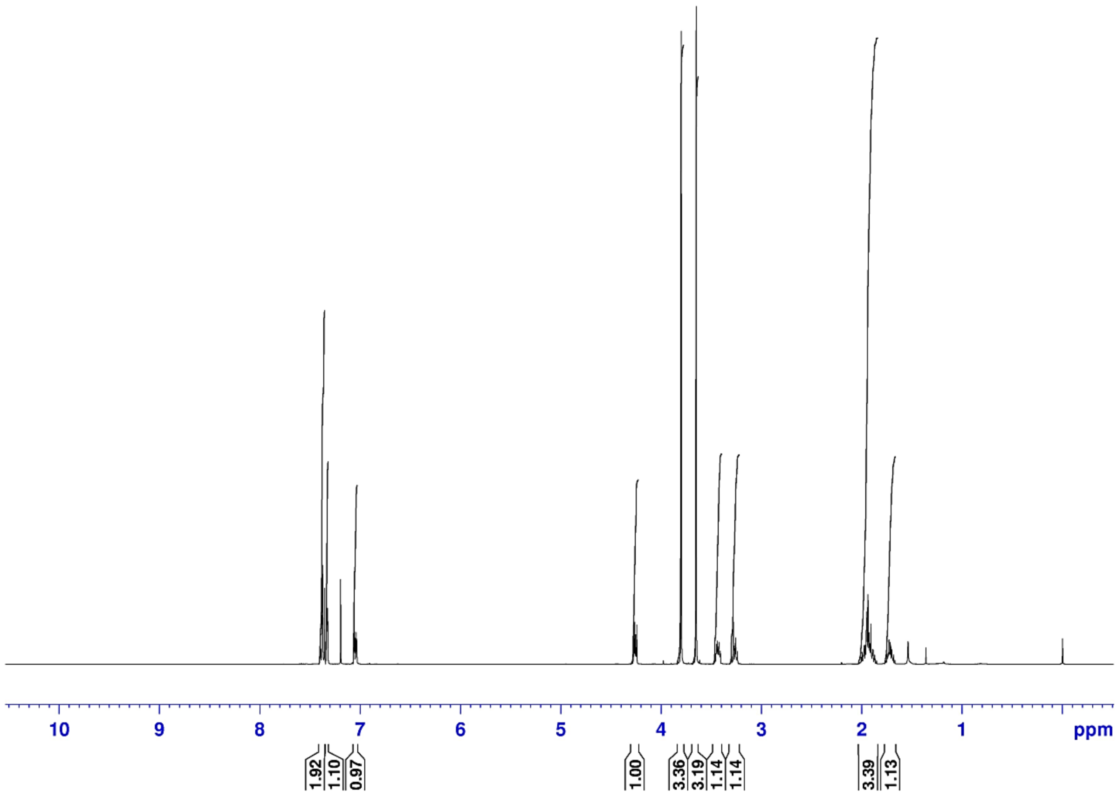

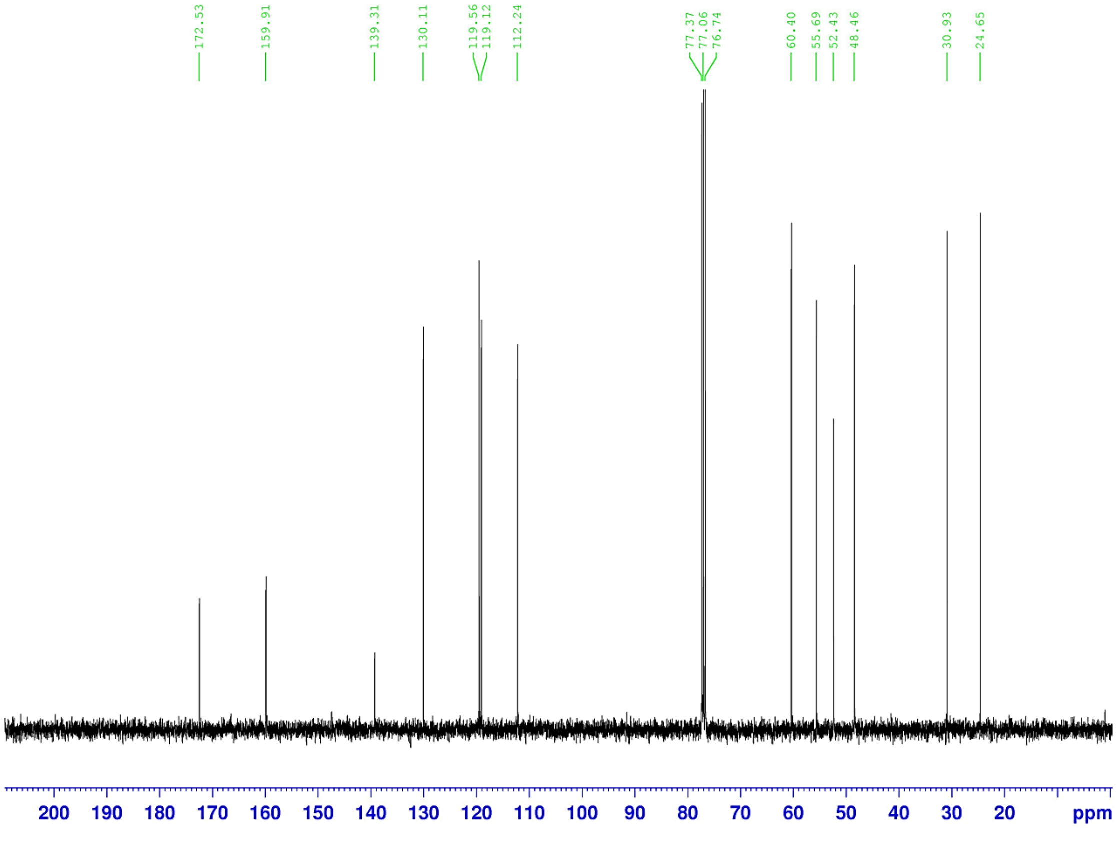

Entry 3, Table 3

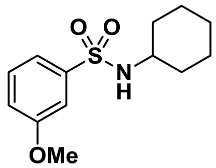

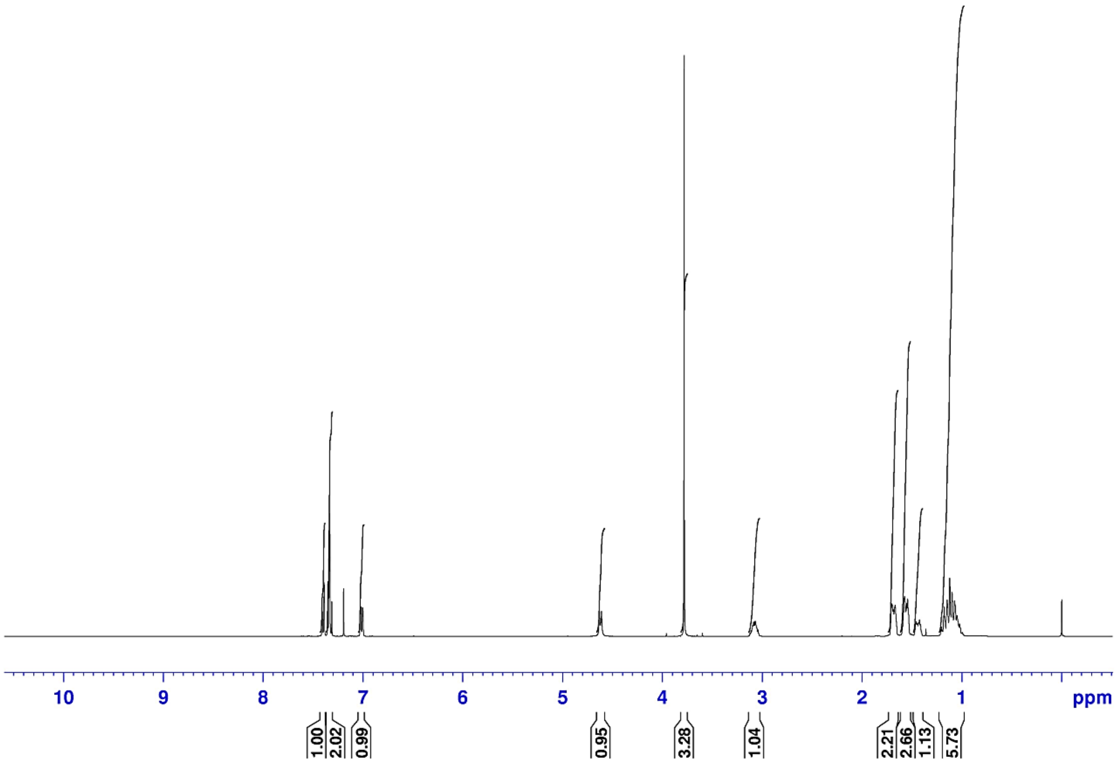

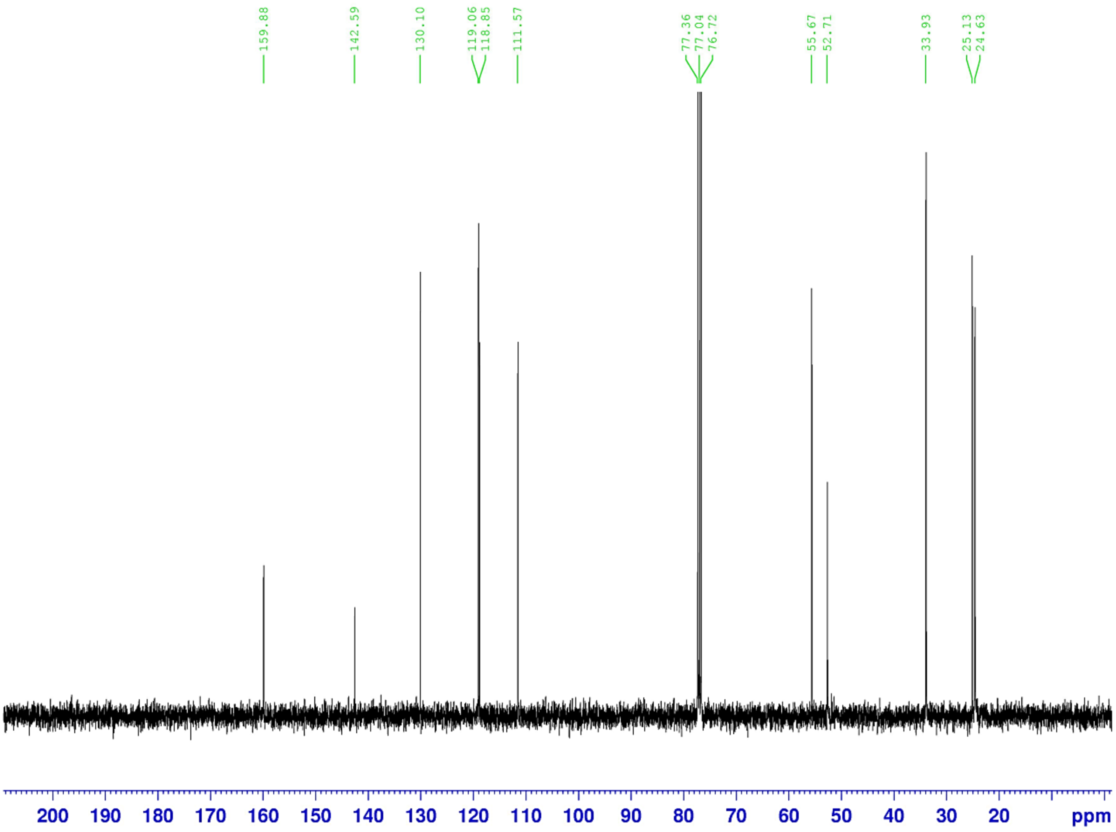

Entry 4, Table 3

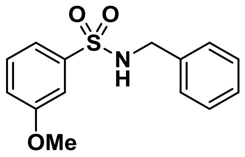

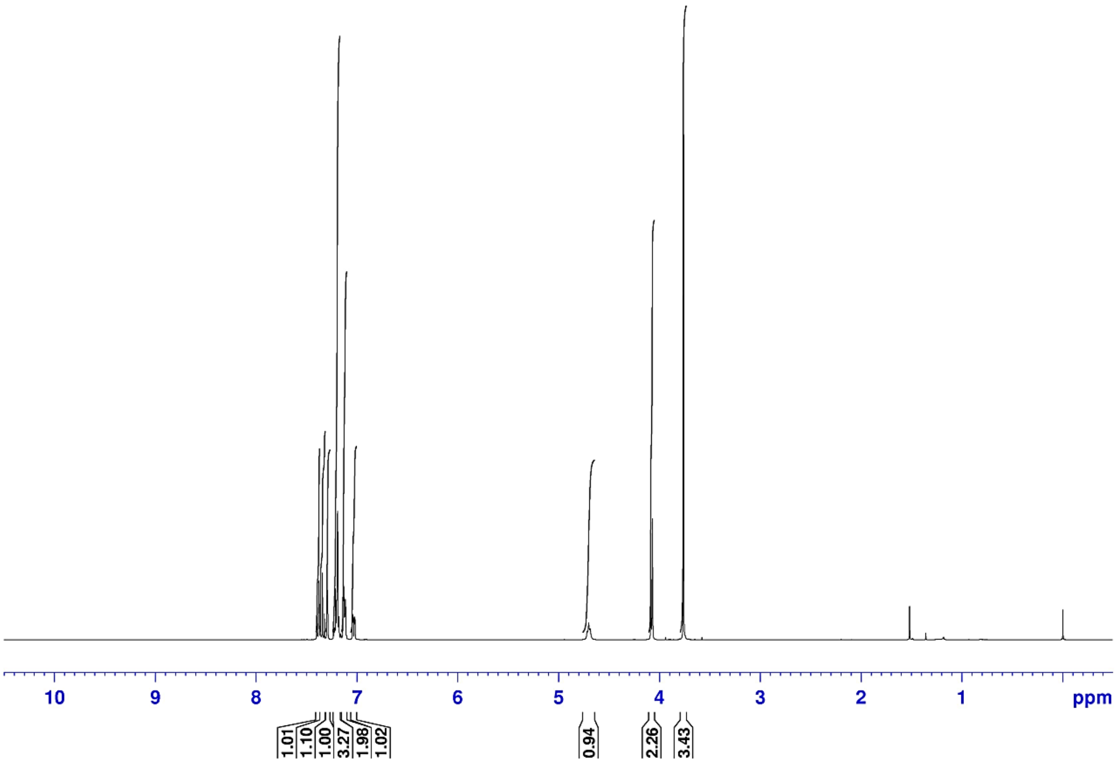

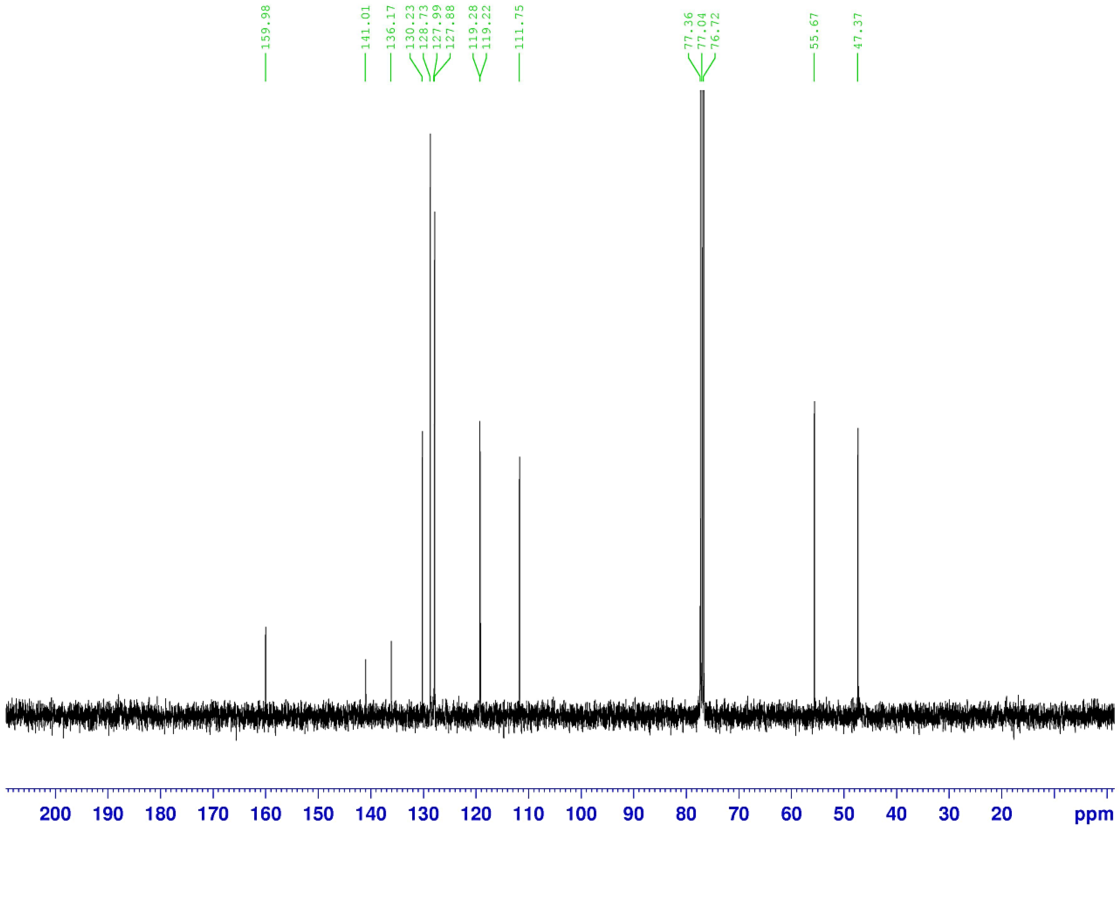

Entry 5, Table 3

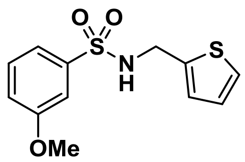

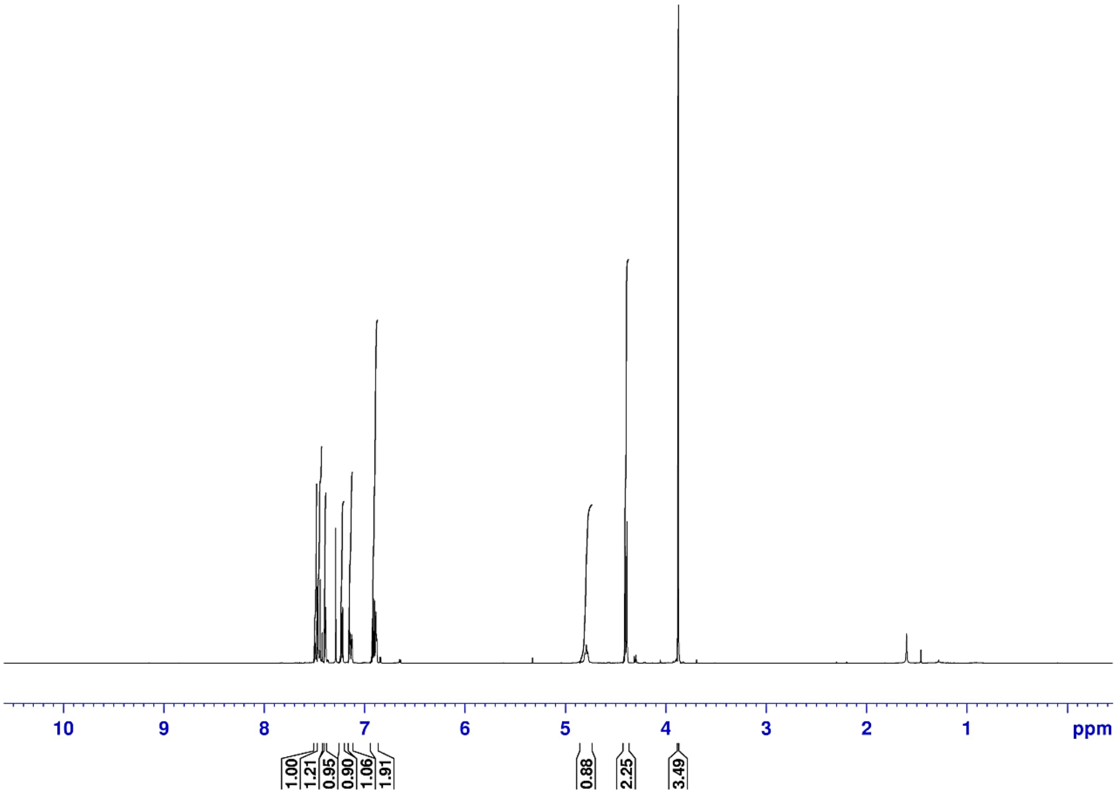

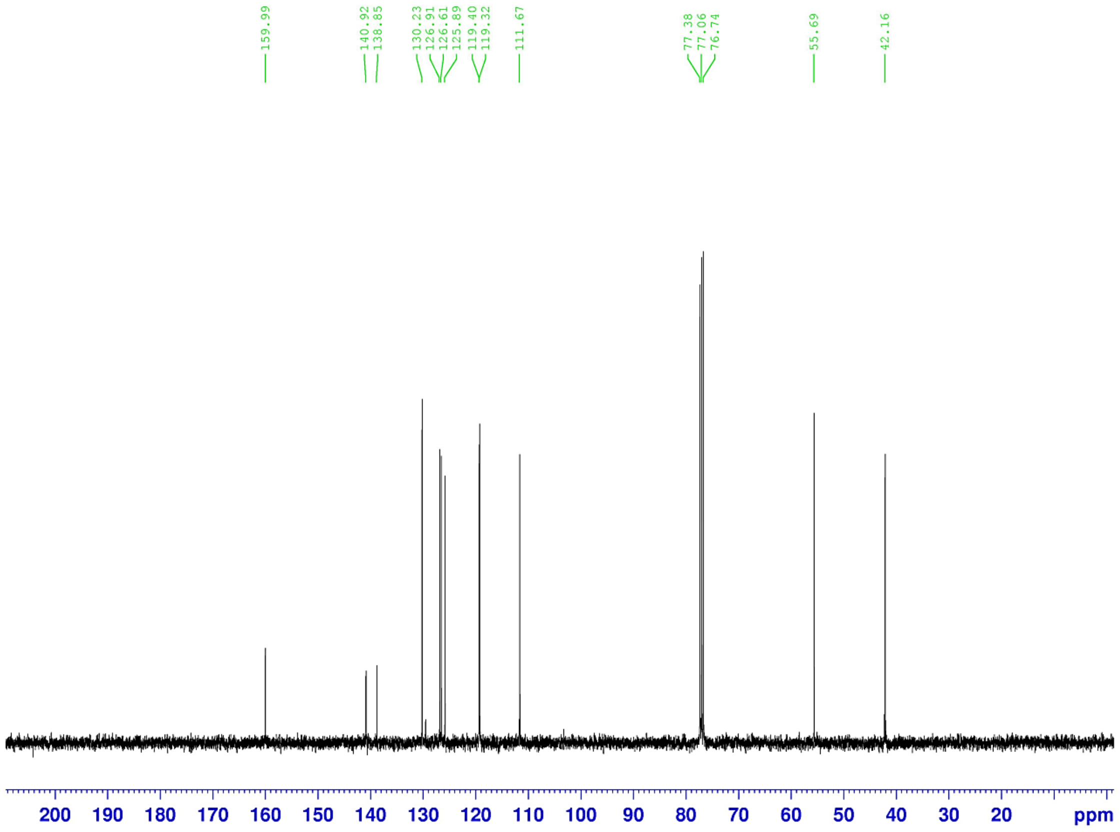

Entry 6, Table 3

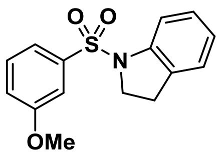

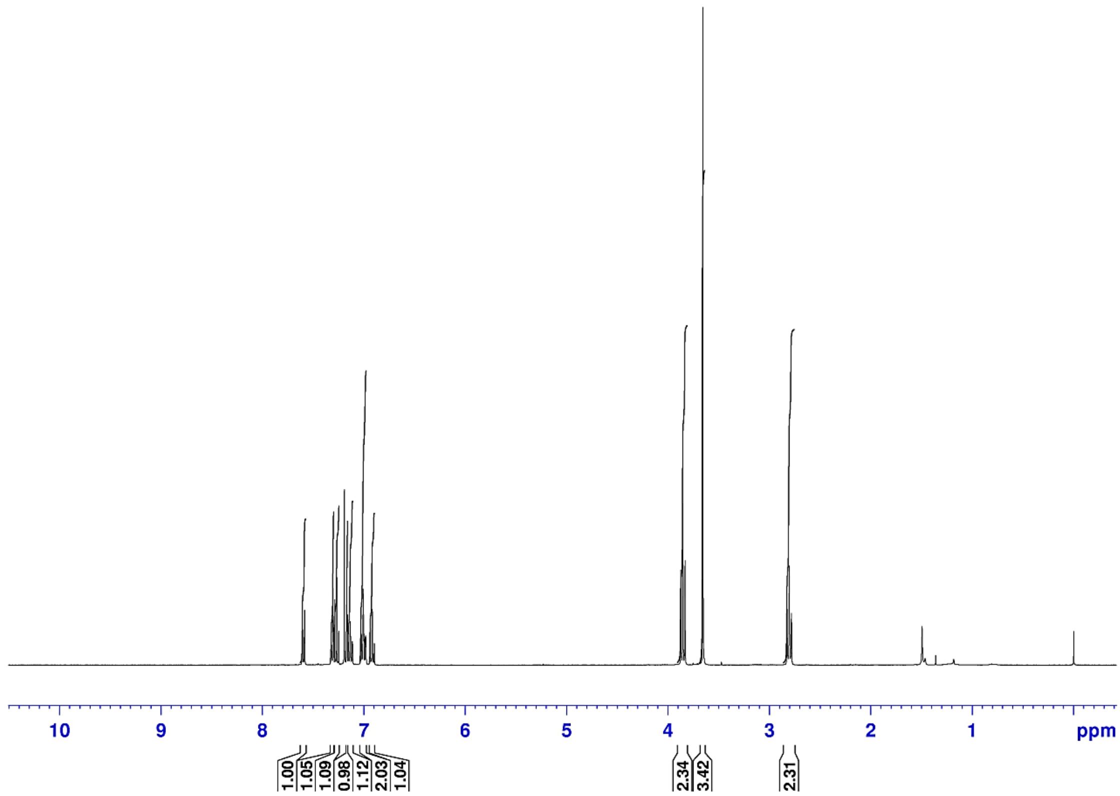

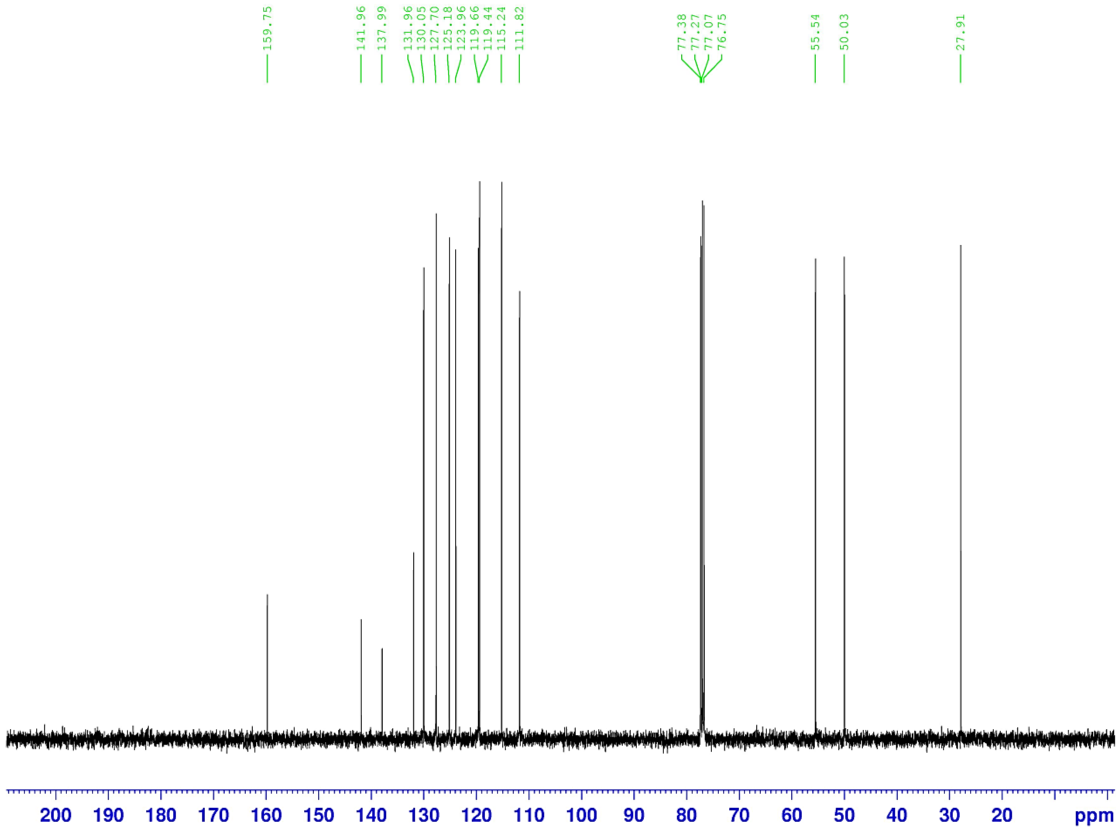

Entry 7, Table 3

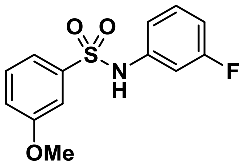

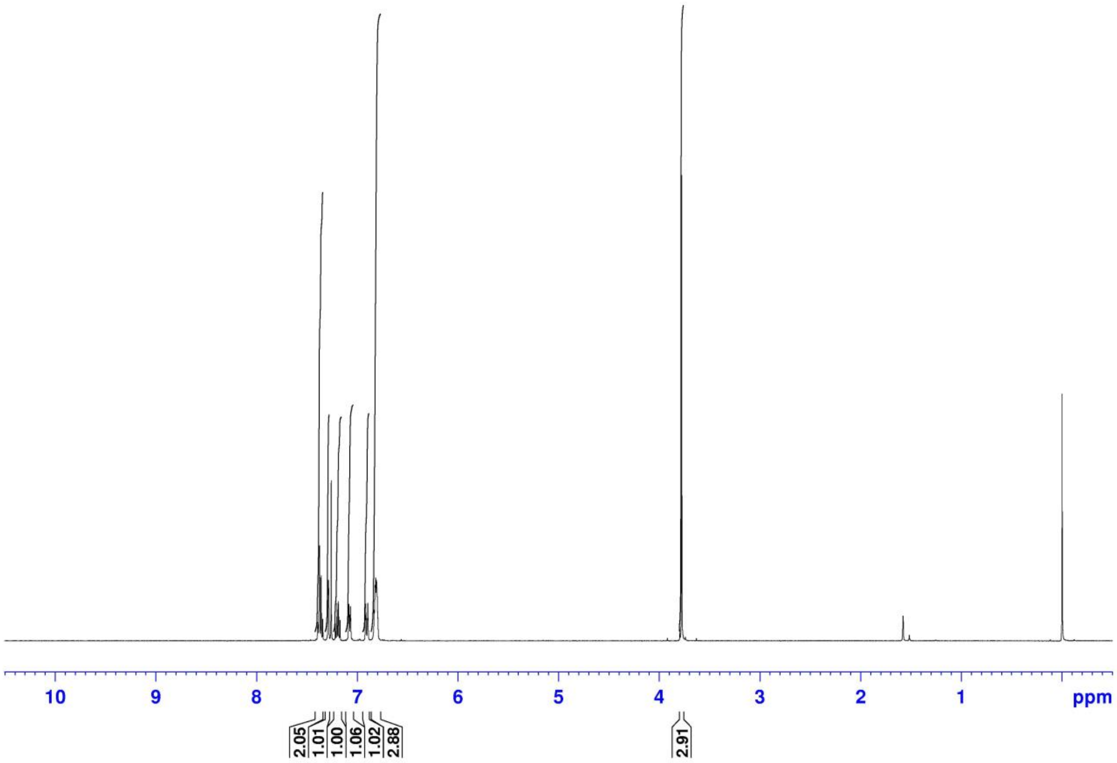

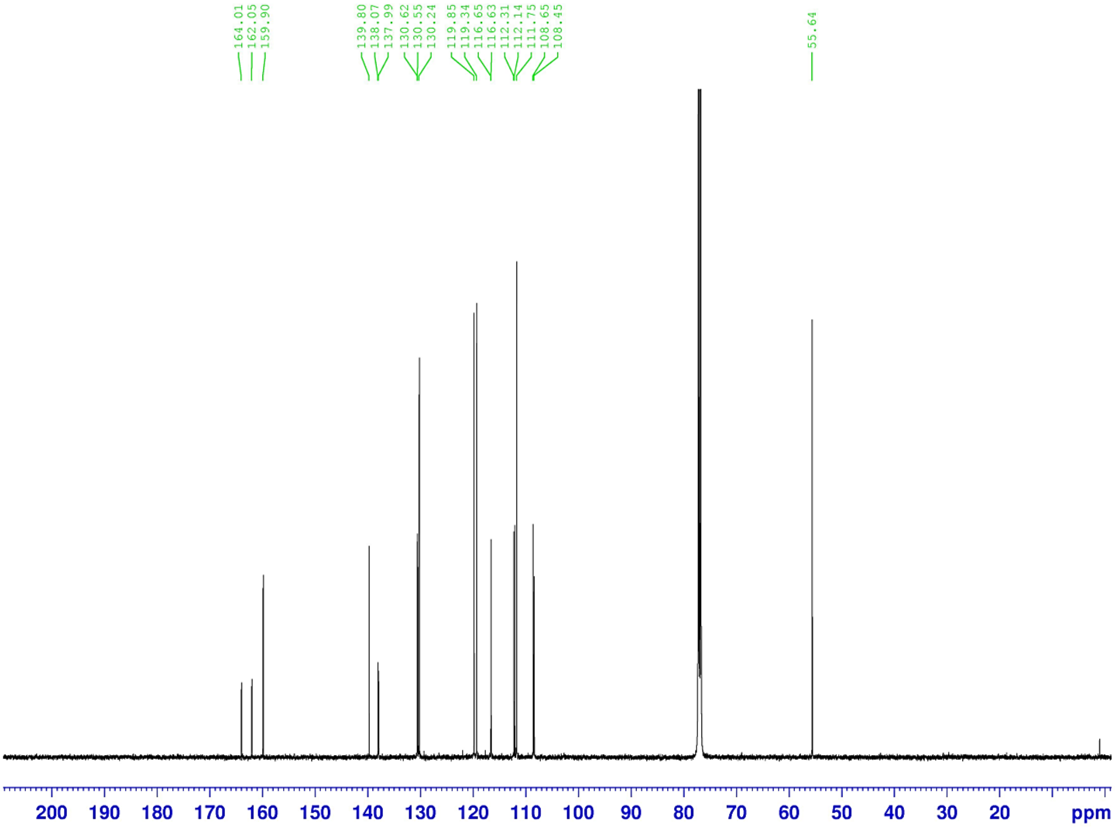

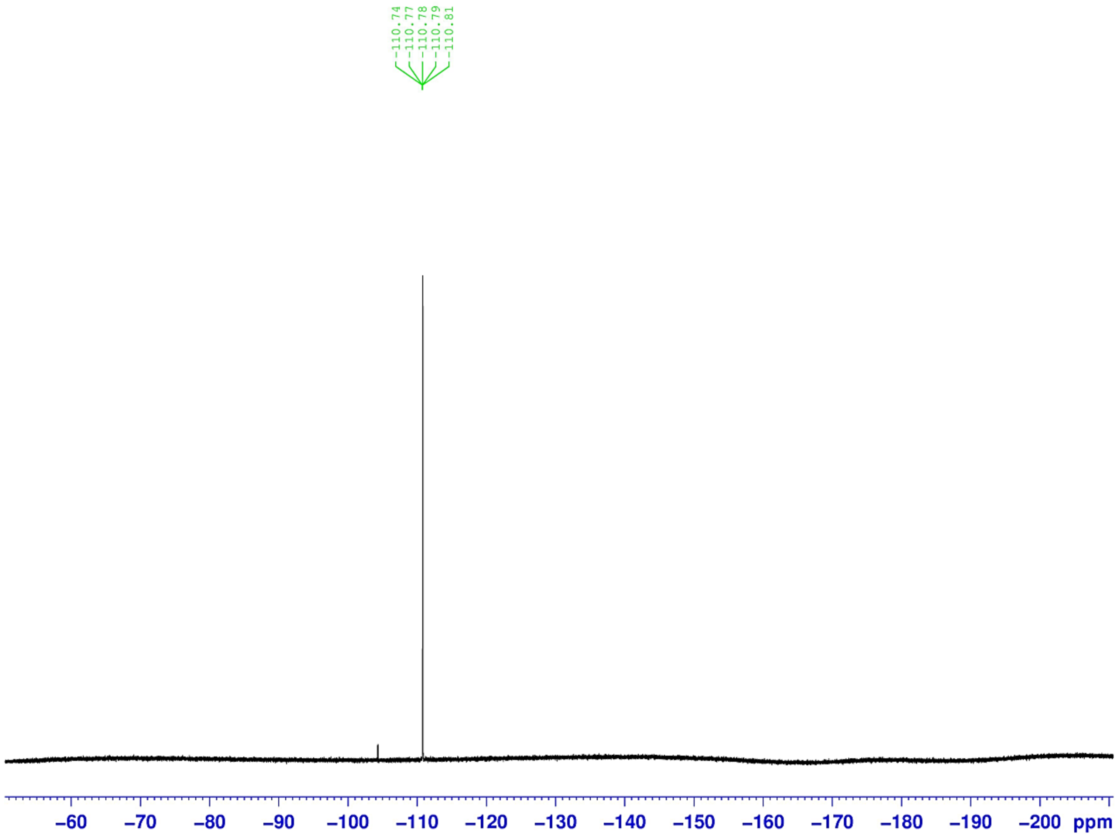

-110.74
-110.77
-110.78
-110.79
-110.81
-60
-70
-80
-90
-100
-110
-120
-130
-140
-150
-160
-170
-180
-190
-200
ppm

Entry 8, Table 3

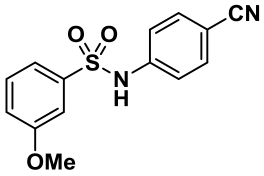

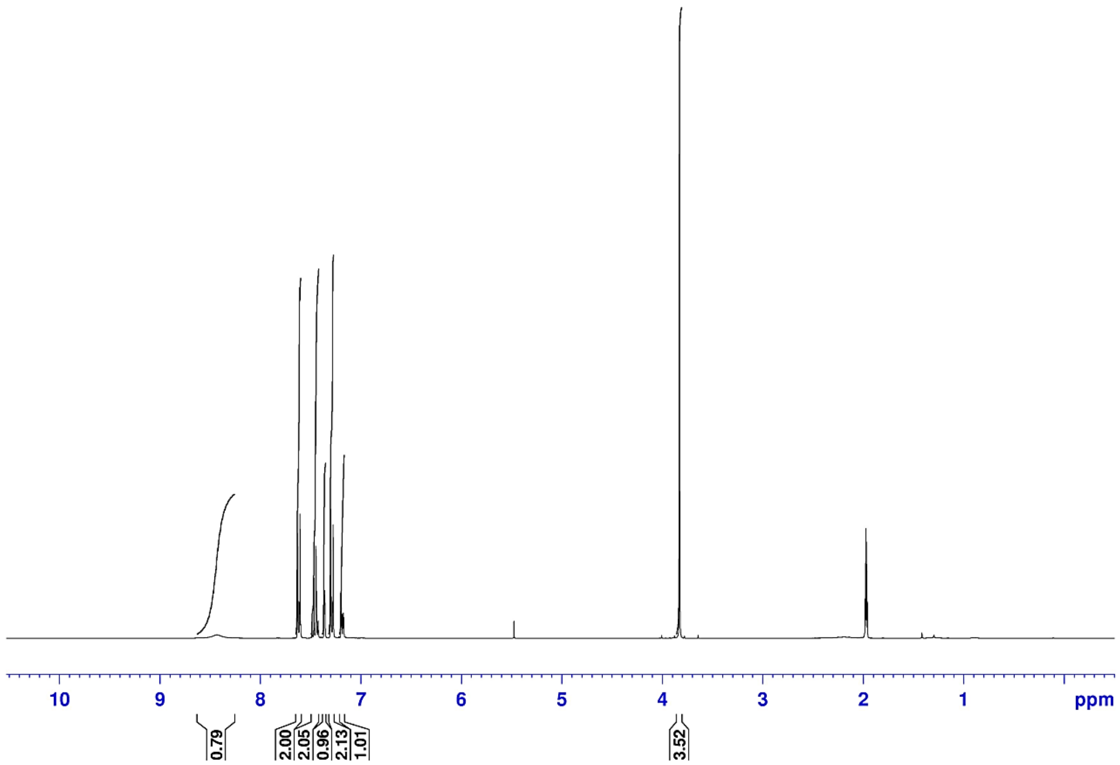

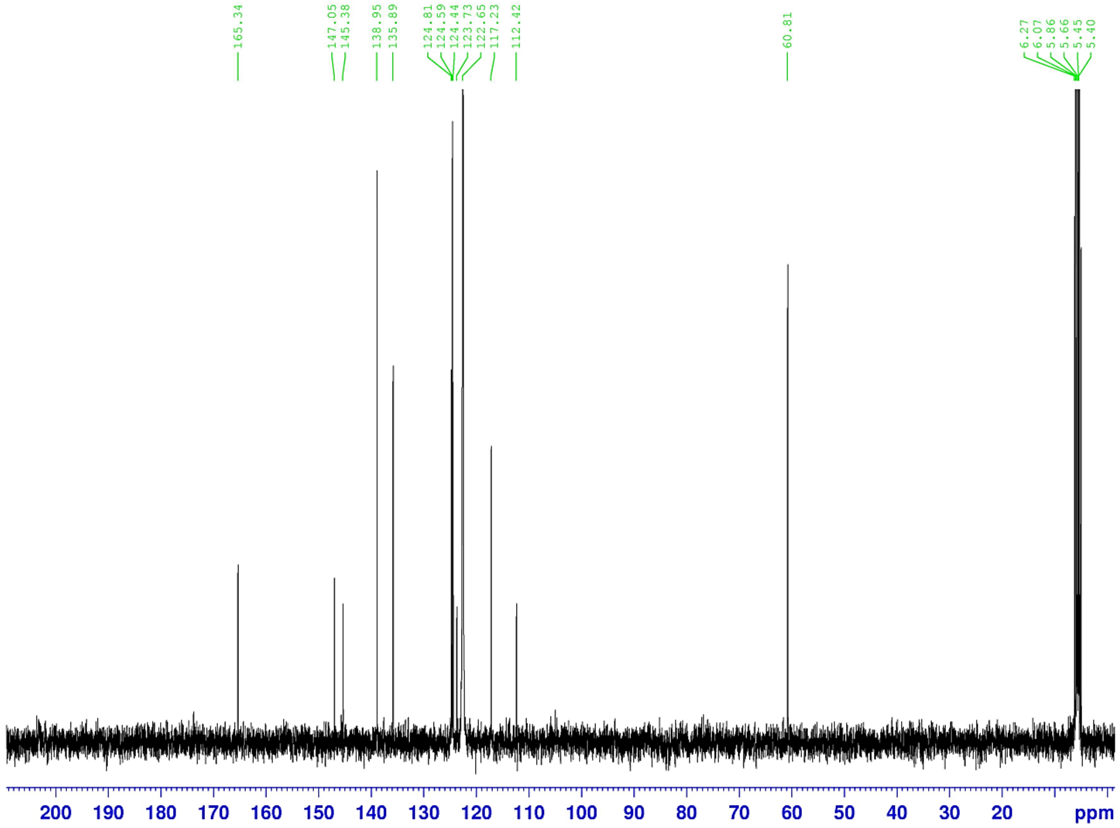

Entry 9, Table 3

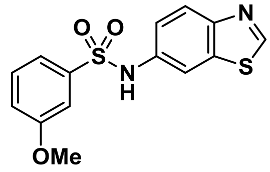

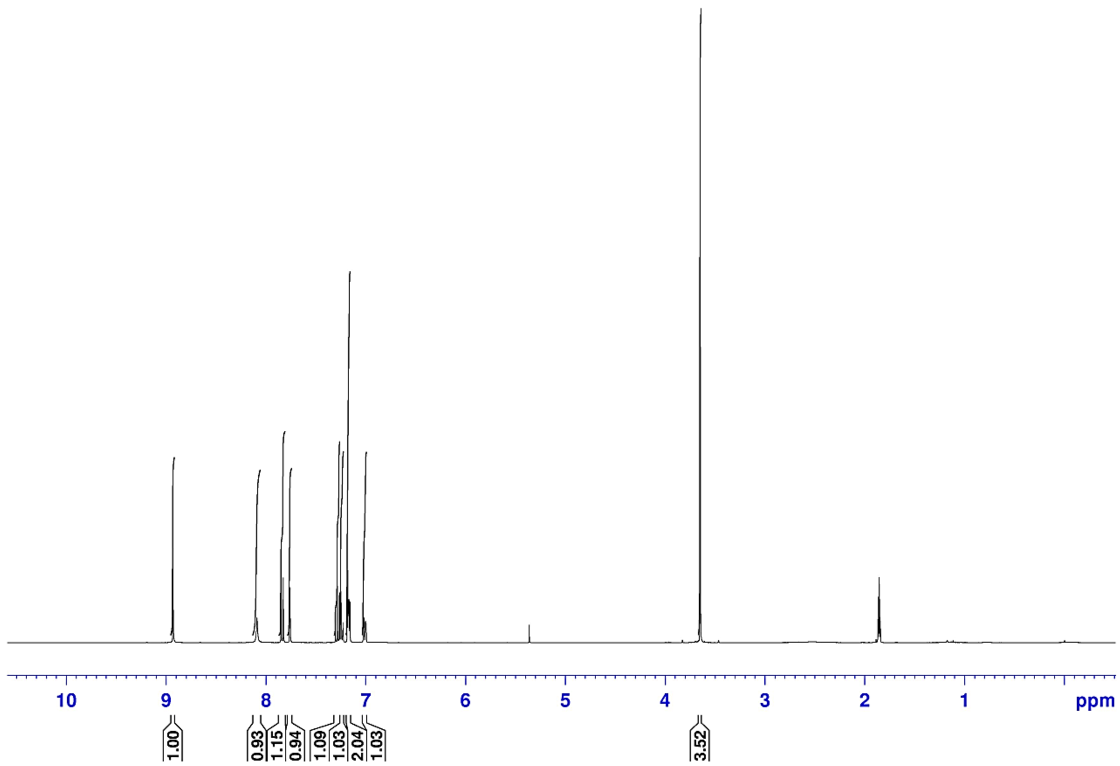

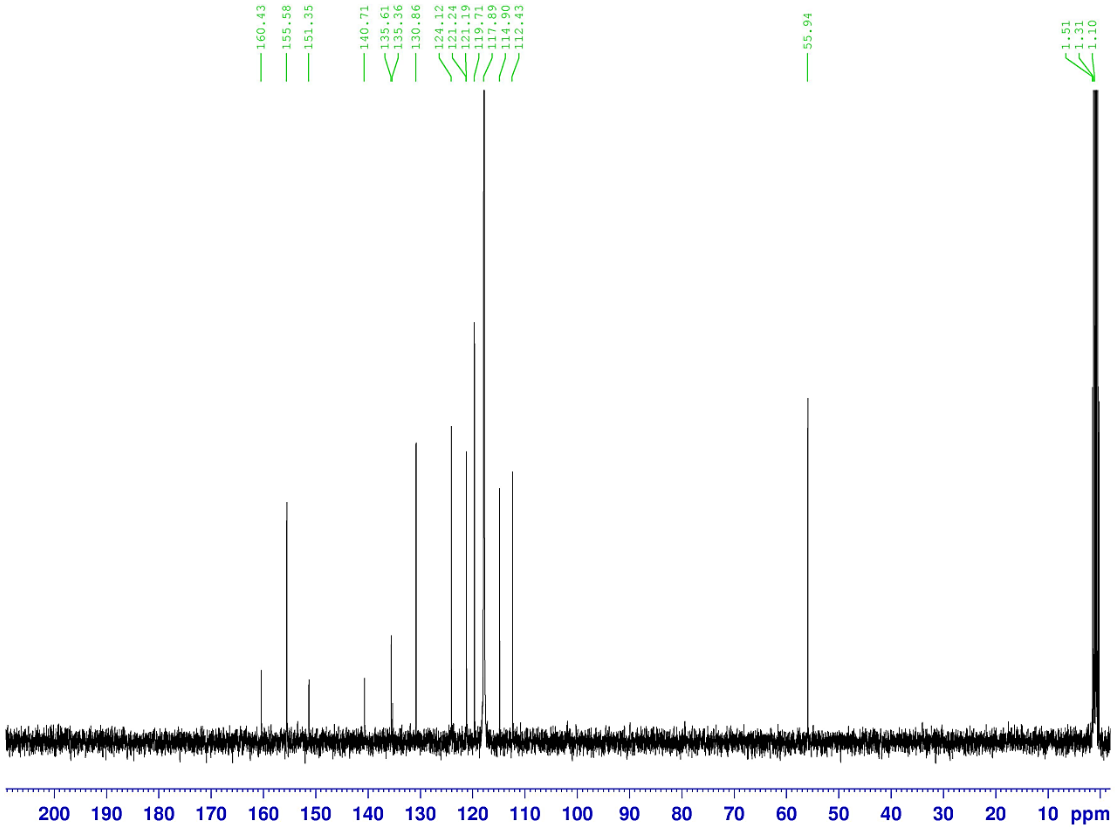

Entry 10, Table 3

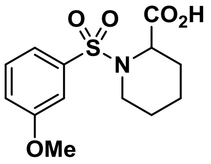

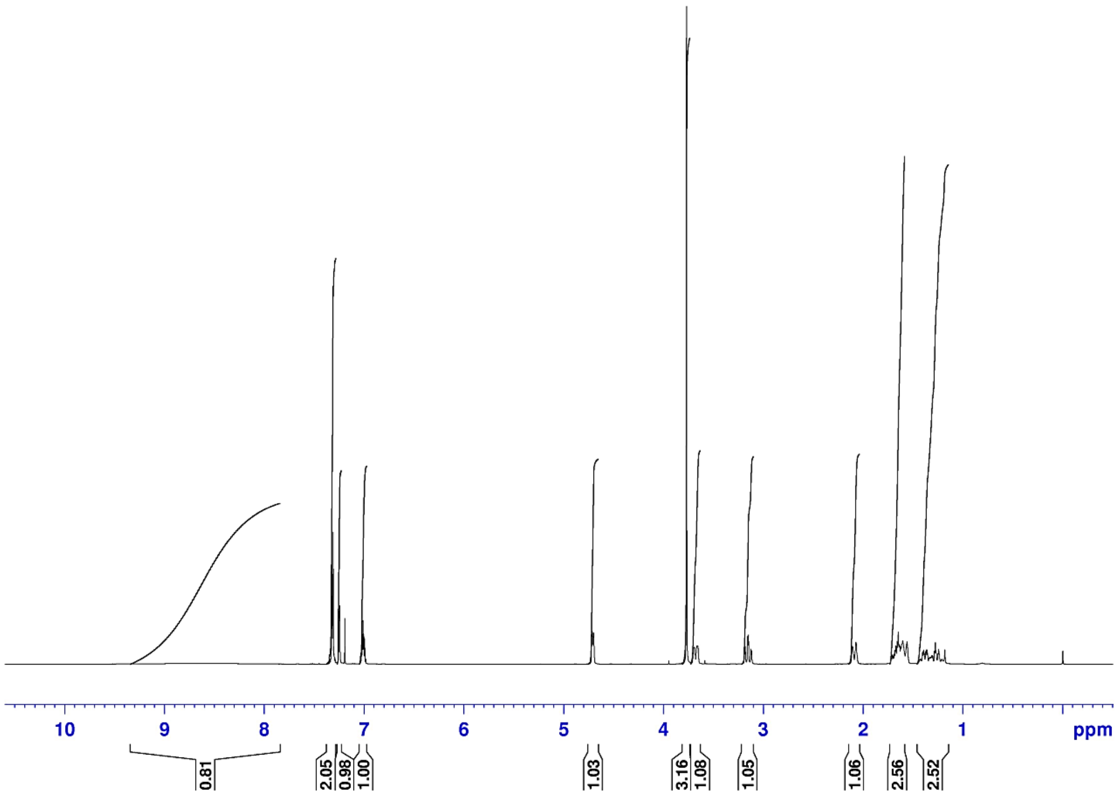

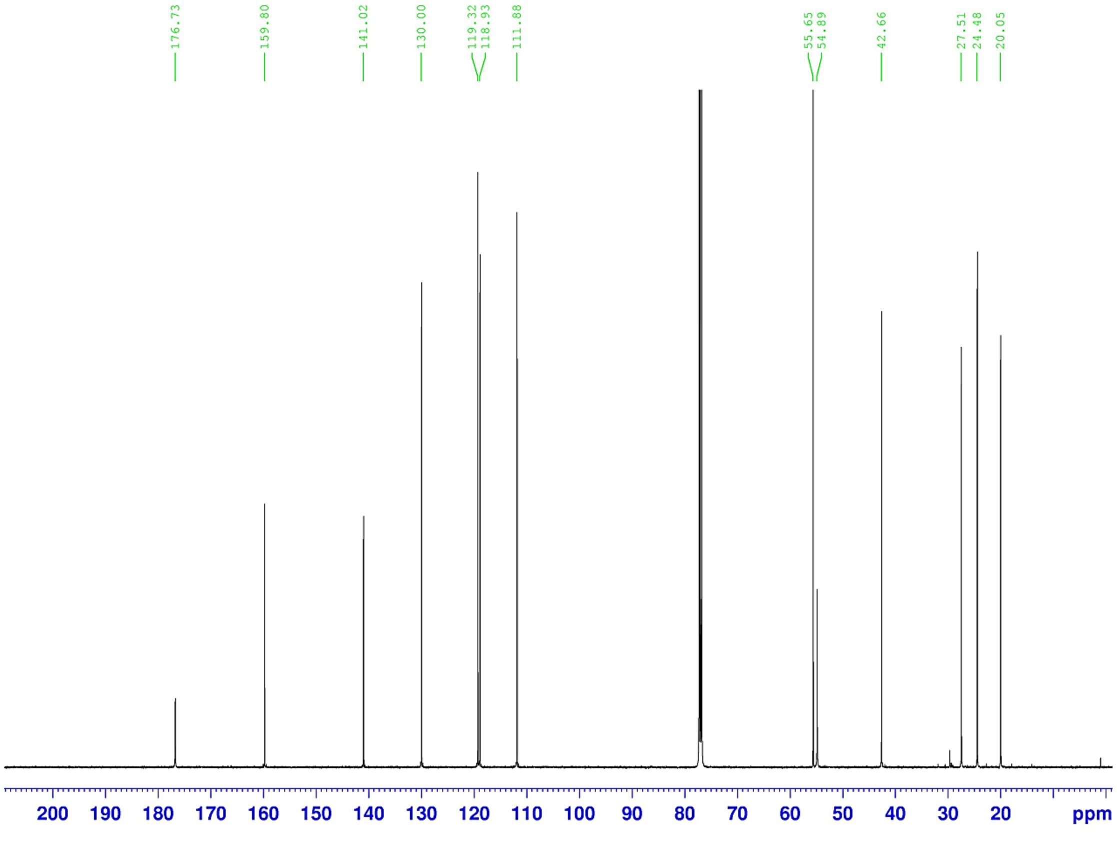

Entry 11, Table 3

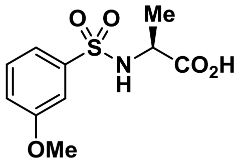

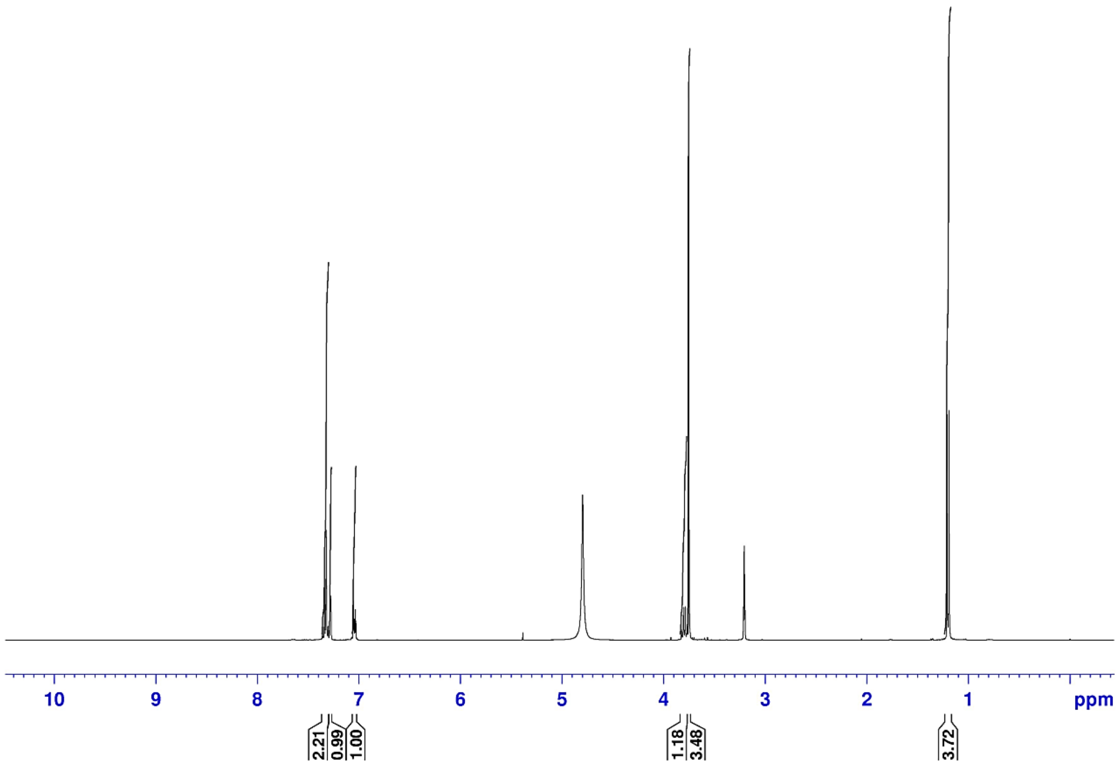

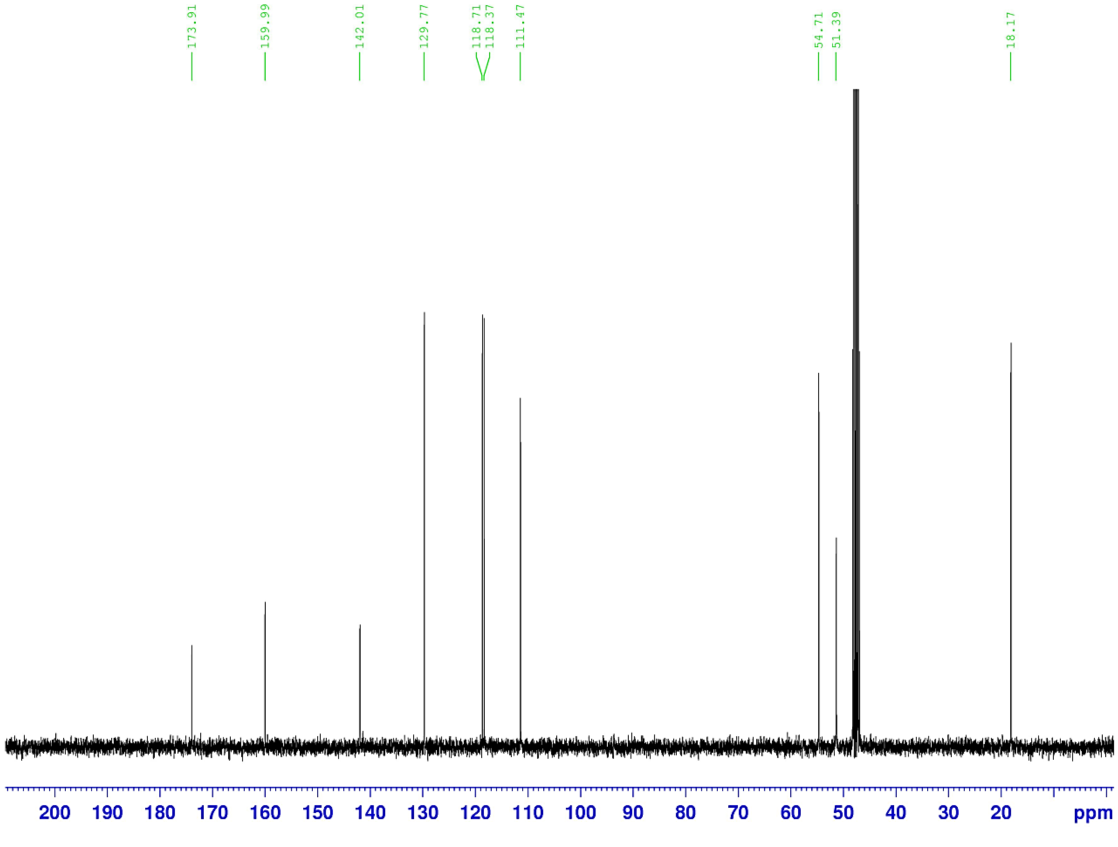

Entry 12, Table 3

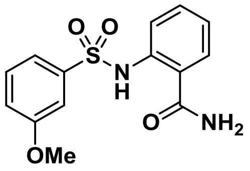

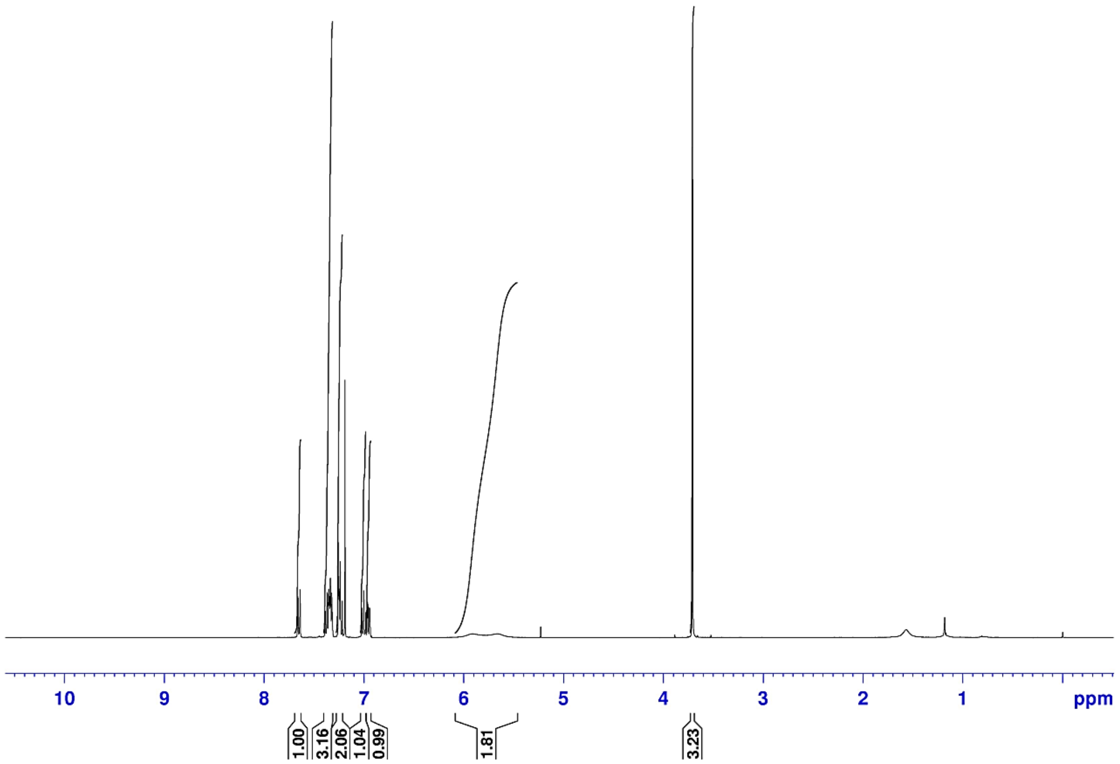

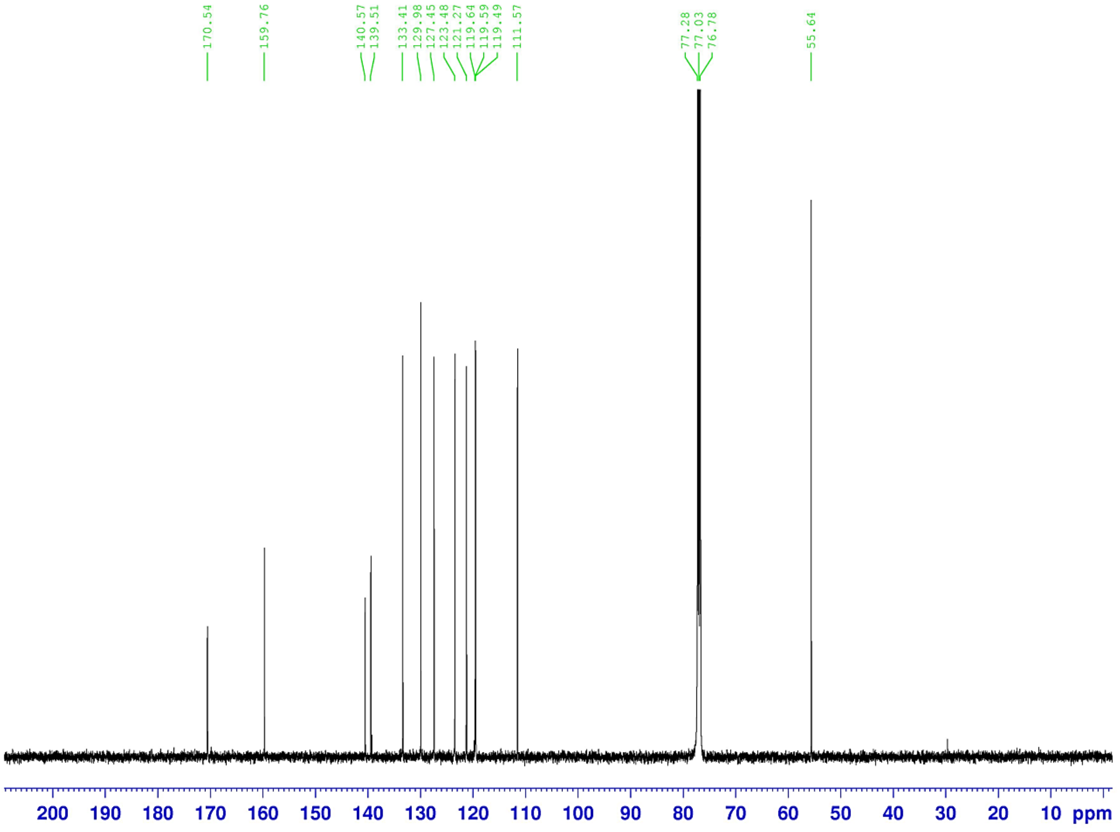

Control Examples

D4

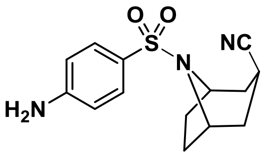

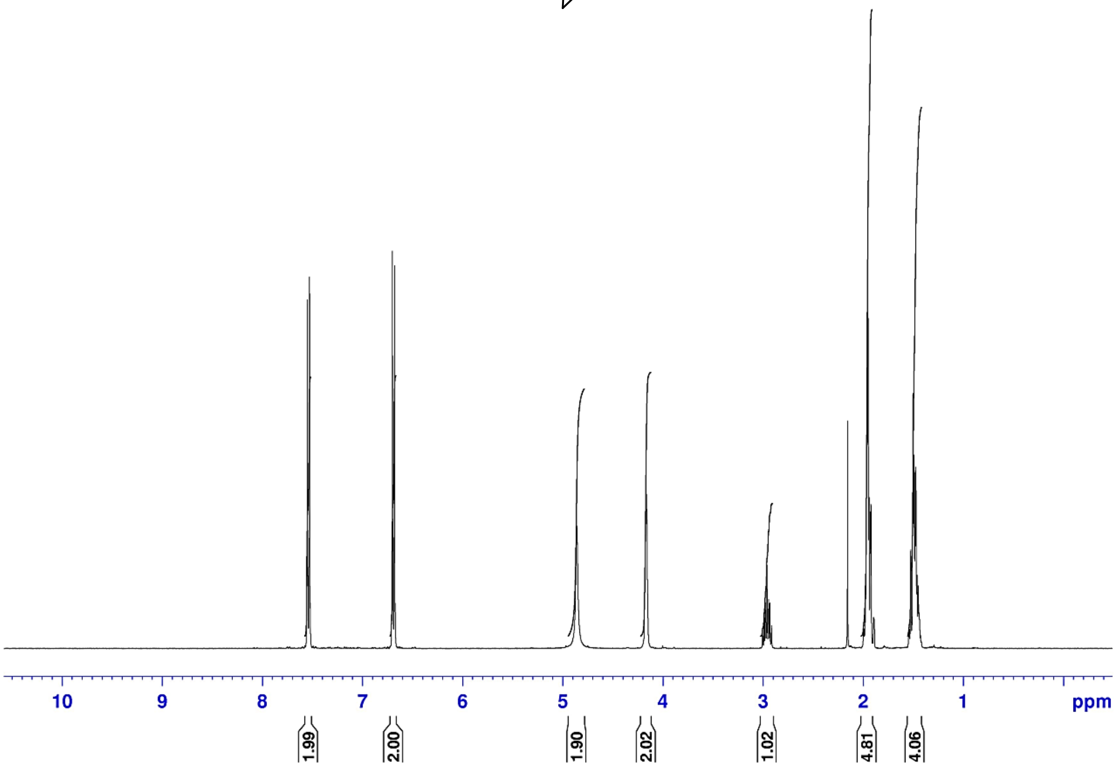

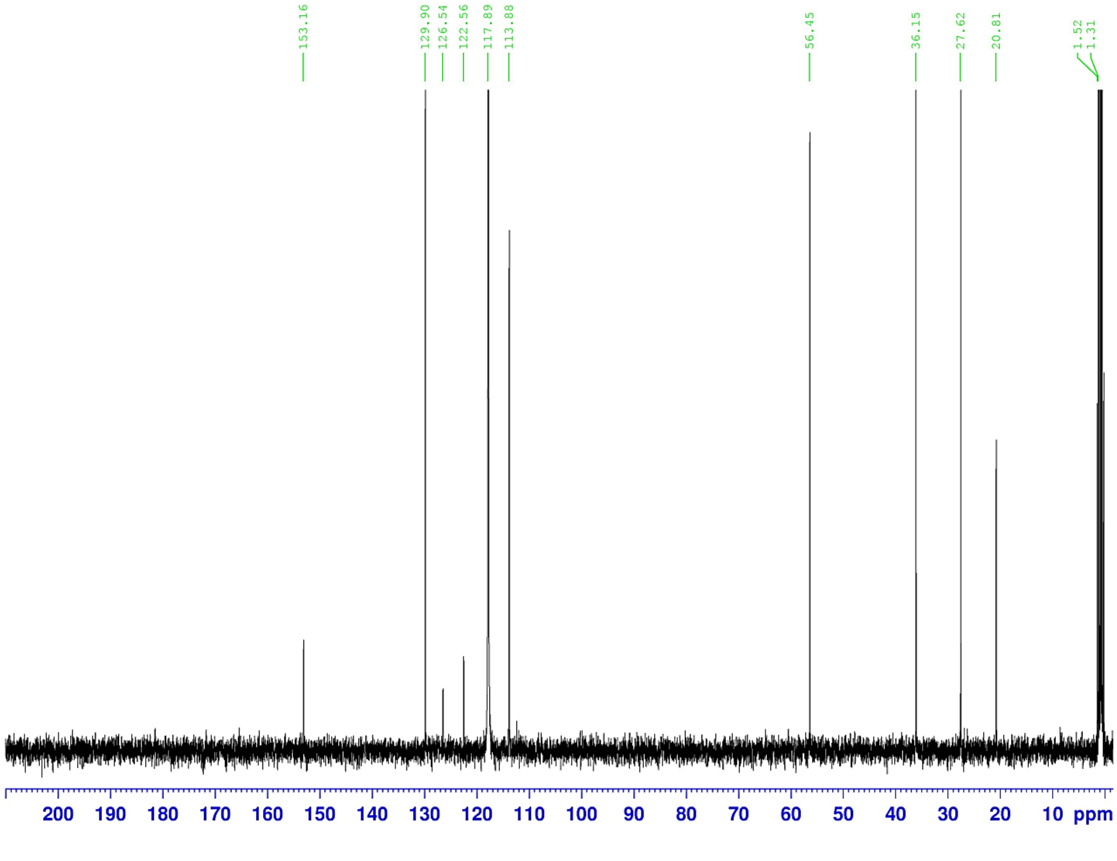

G6

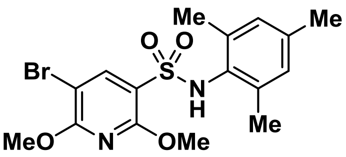
Br
MeO
N
OMe
O
O
S
N
H
Me
Me
Me

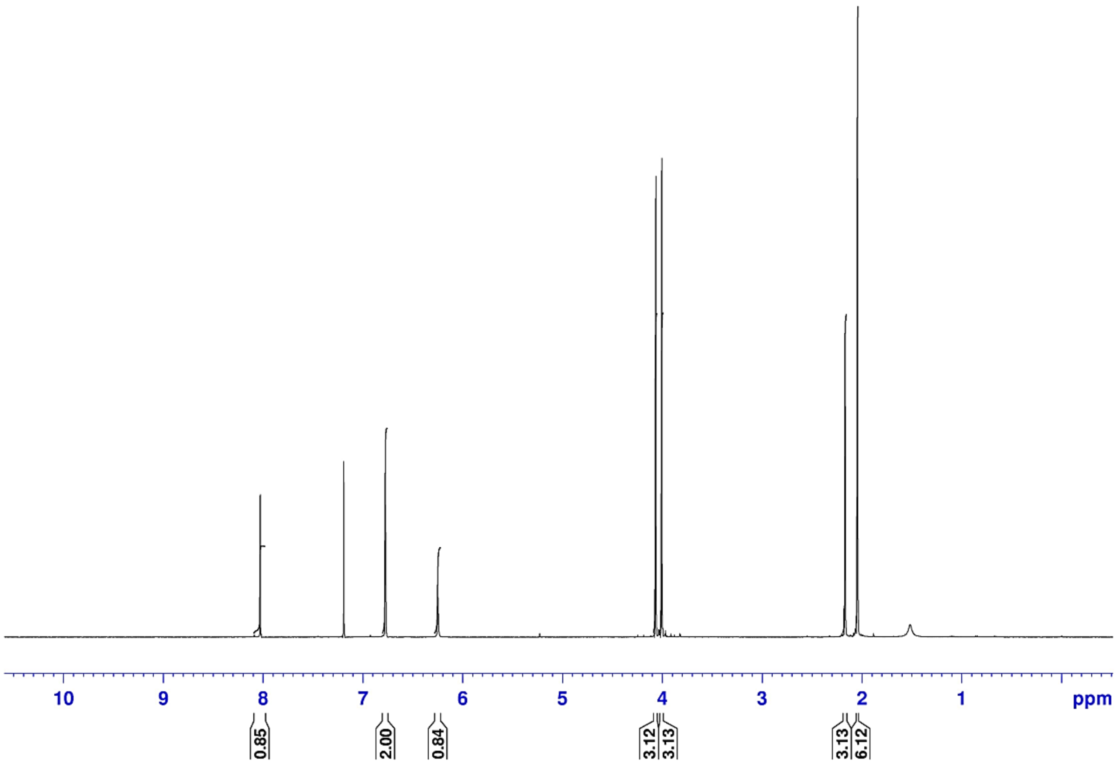
10
9
8
7
6
5
4
3
2
1
ppm
0.85
2.00
0.84
3.12
3.13
3.13
6.12

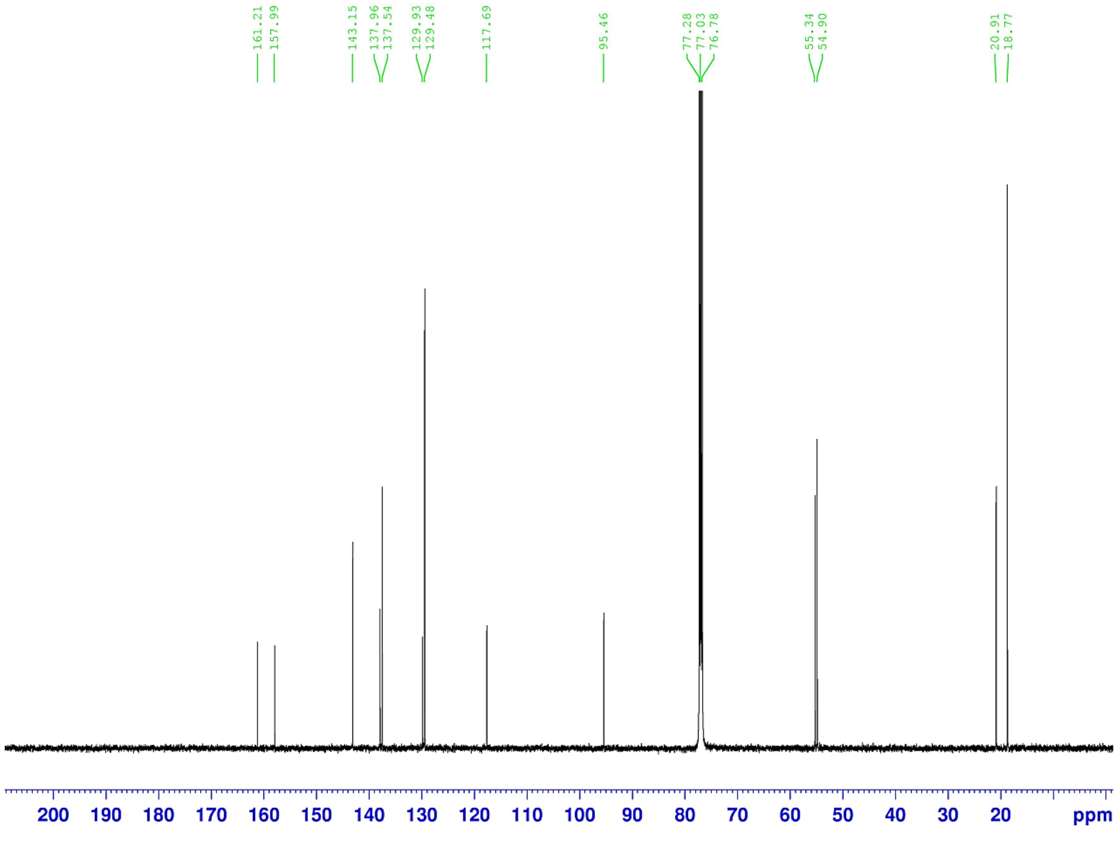
161.21
157.99
143.15
137.96
137.54
129.93
129.48
117.69
95.46
77.28
77.03
76.78
55.34
54.90
20.91
18.77
200
190
180
170
160
150
140
130
120
110
100
90
80
70
60
50
40
30
20
ppm

Entry 2, Table 3

Sample Name:	**product**	*Injection Volume:*	**10.0**
Vial Number:	**GB1**	*Channel:*	**UV_VIS_1**
Sample Type:	**unknown**	*Wavelength:*	**225**
Control Program:	**Col_A 10% isocratic**	*Bandwidth:*	**1**
Quantif. Method:	**Standard**	*Dilution Factor:*	**1.0000**
Recording Time:	**21/8/2014 11:35**	*Sample Weight:*	**1.0000**
Run Time (min):	**30.00**	*Sample Amount:*	**1.0000**

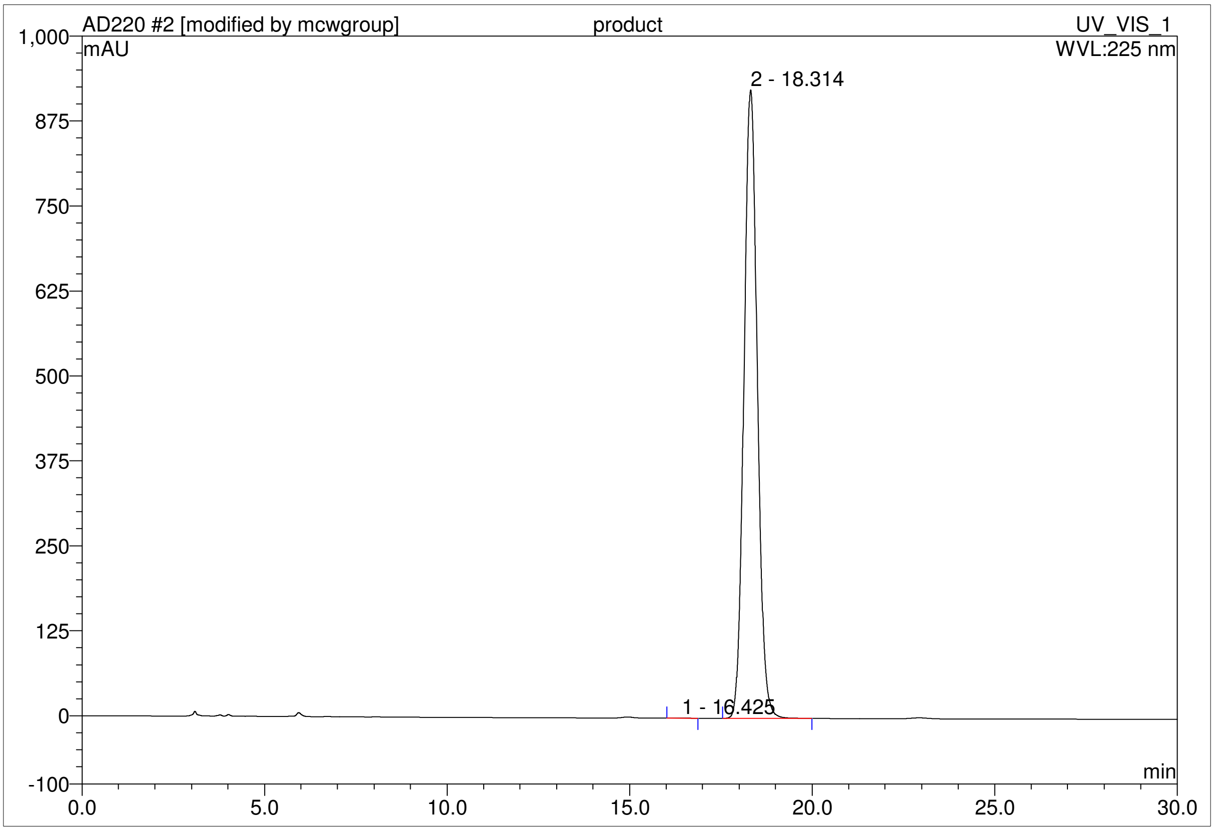

No.	Ret.Time min	Peak Name	Height mAU	Area mAU*min	Rel.Area %	Amount	Type
1	16.42	n.a.	0.245	0.091	0.02	n.a.	BMB
2	18.31	n.a.	924.277	394.542	99.98	n.a.	BMB
**Total:**			924.522	394.633	100.00	0.000	

Entry 2, Table 3 (racemic)

Sample Name:	**rac**	*Injection Volume:*	**10.0**
Vial Number:	**GA1**	*Channel:*	**UV_VIS_1**
Sample Type:	**unknown**	*Wavelength:*	**225**
Control Program:	**Col_A 10% isocratic**	*Bandwidth:*	**1**
Quantif. Method:	**Standard**	*Dilution Factor:*	**1.0000**
Recording Time:	**22/8/2014 17:25**	*Sample Weight:*	**1.0000**
Run Time (min):	**30.00**	*Sample Amount:*	**1.0000**

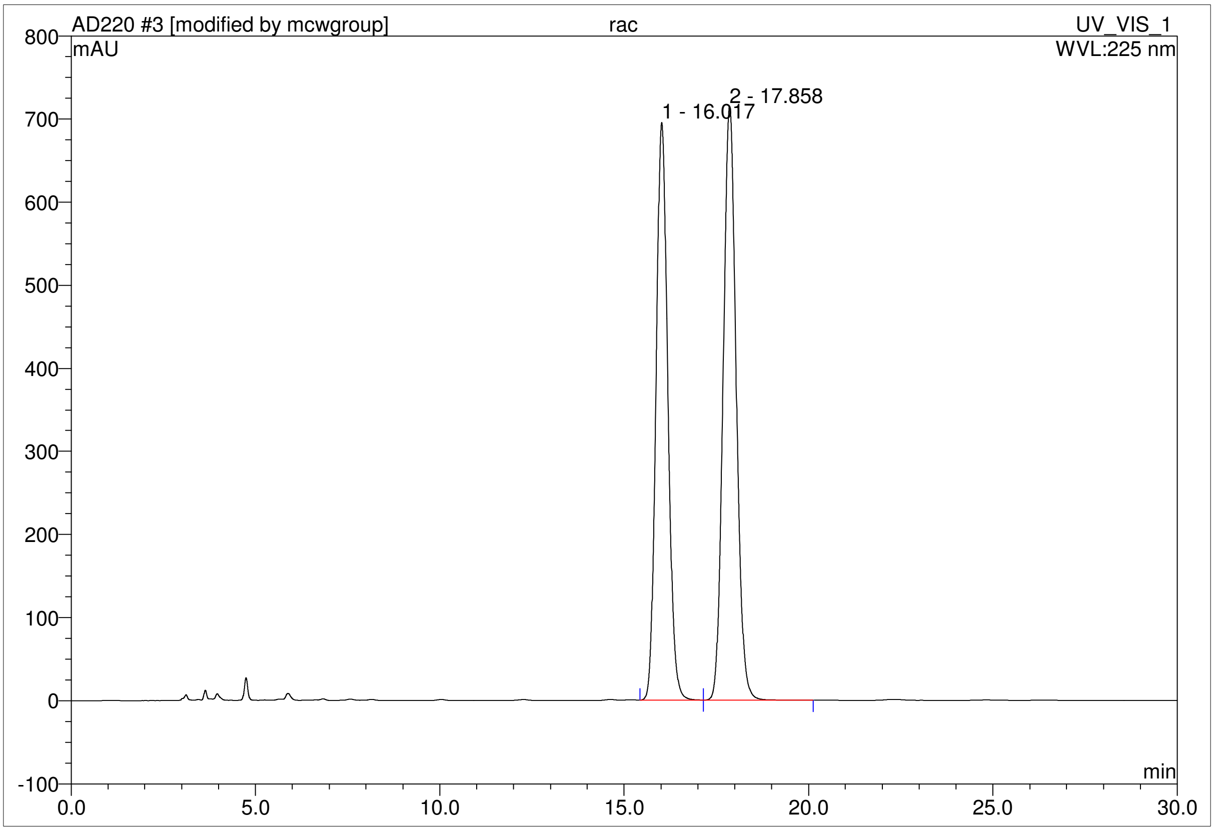

No.	Ret.Time min	Peak Name	Height mAU	Area mAU*min	Rel.Area %	Amount	Type
1	16.02	n.a.	695.013	258.757	46.84	n.a.	BM *
2	17.86	n.a.	713.852	293.697	53.16	n.a.	MB*
**Total:**			1408.865	552.454	100.00	0.000	

Entry 11, Table 3

Sample Name:	**AD336-pure IC 40% 50min**	*Injection Volume:*	**20.0**
Vial Number:	**GC12**	*Channel:*	**UV_VIS_1**
Sample Type:	**unknown**	*Wavelength:*	**225**
Control Program:	**col a 1% 1 5 ml 30min**	*Bandwidth:*	**1**
Quantif. Method:	**Ph-pent**	*Dilution Factor:*	**1.0000**
Recording Time:	**14/3/2014 14:16**	*Sample Weight:*	**1.0000**
Run Time (min):	**50.00**	*Sample Amount:*	**1.0000**

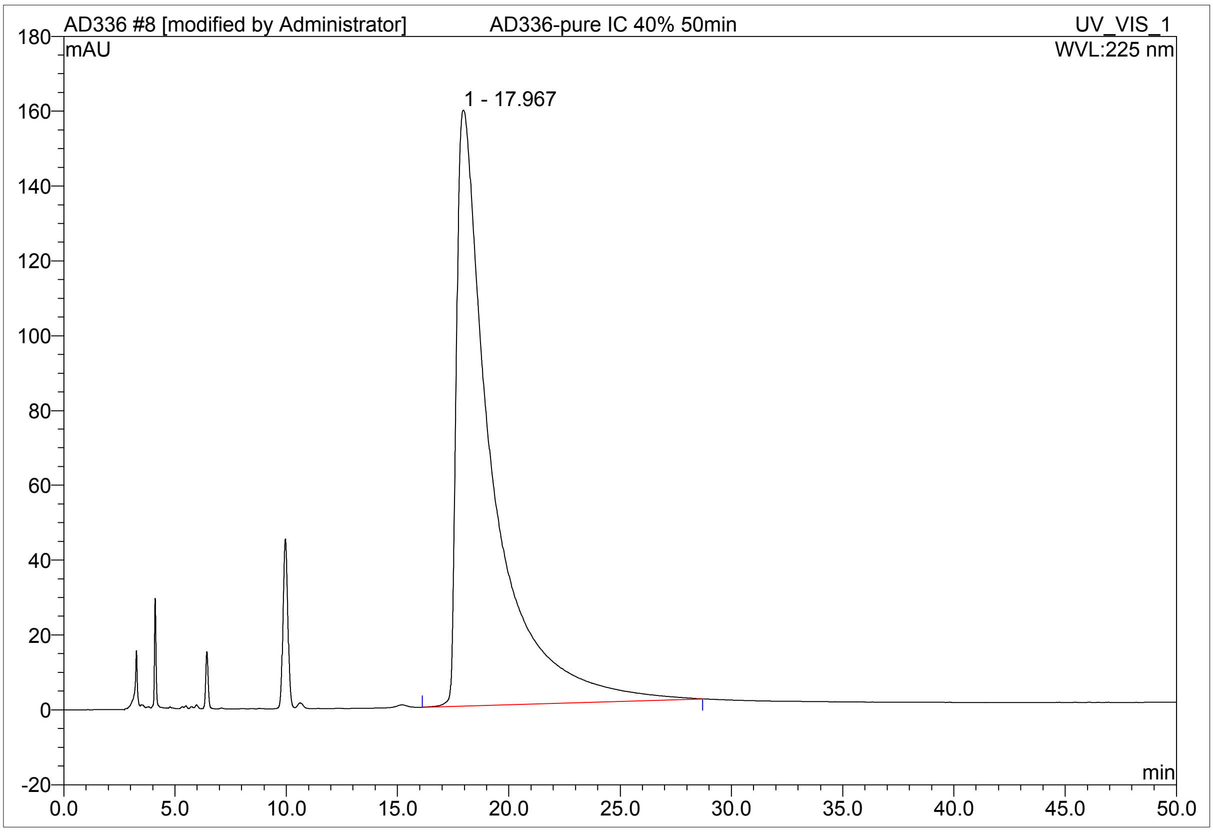

No.	Ret.Time min	Peak Name	Height mAU	Area mAU*min	Rel.Area %	Amount	Type
1	17.97	n.a.	159.298	295.202	100.00	n.a.	BMB
**Total:**			159.298	295.202	100.00	0.000	

Entry 11, Table 3 (racemic)

Sample Name:	**AD336-rac IC 40% 60min**	*Injection Volume:*	**20.0**
Vial Number:	**GC12**	*Channel:*	**UV_VIS_1**
Sample Type:	**unknown**	*Wavelength:*	**225**
Control Program:	**col a 1% 1 5 ml 30min**	*Bandwidth:*	**1**
Quantif. Method:	**Ph-pent**	*Dilution Factor:*	**1.0000**
Recording Time:	**14/3/2014 13:32**	*Sample Weight:*	**1.0000**
Run Time (min):	**41.28**	*Sample Amount:*	**1.0000**

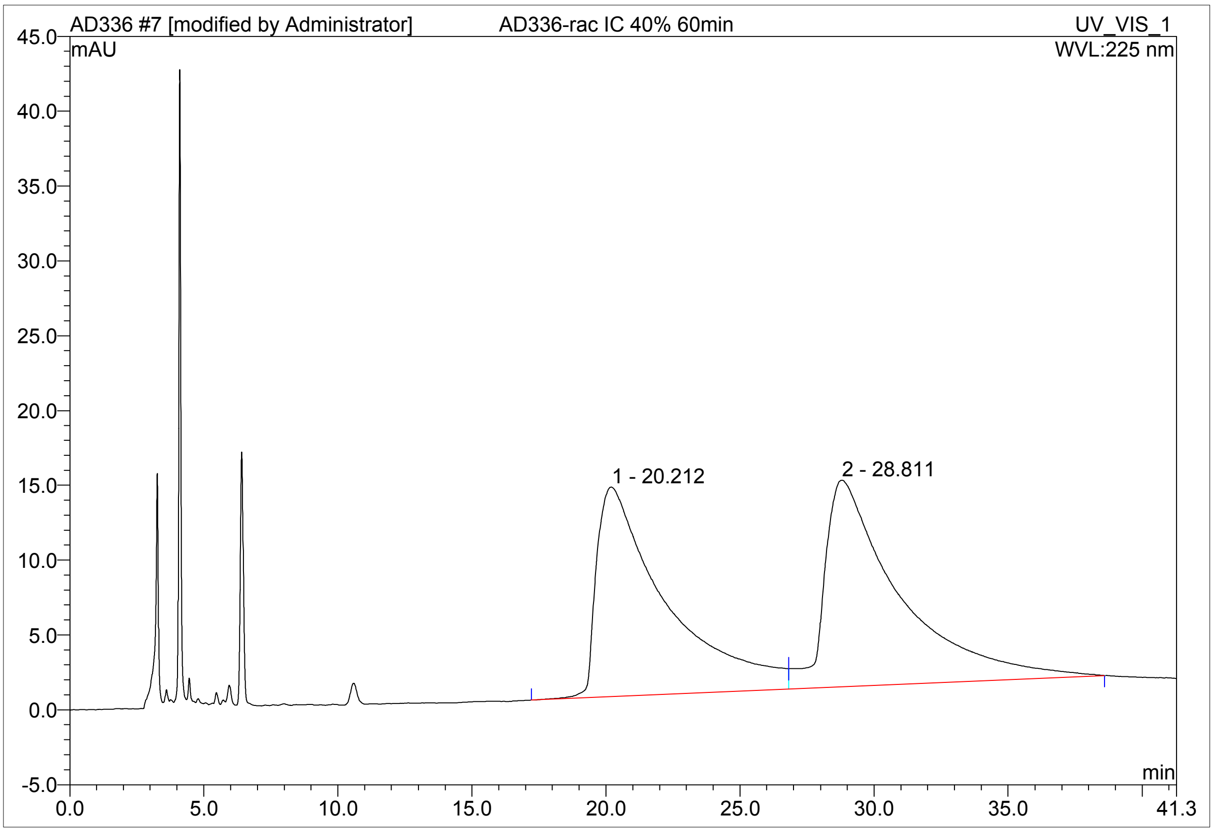

No.	Ret.Time min	Peak Name	Height mAU	Area mAU*min	Rel.Area %	Amount	Type
1	20.21	n.a.	13.998	42.054	48.38	n.a.	BM *
2	28.81	n.a.	13.796	44.863	51.62	n.a.	MB*
**Total:**			27.794	86.917	100.00	0.000	

6. Array Synthesis ^{1}H NMR and LC-MS

A1

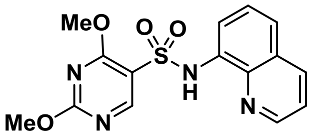

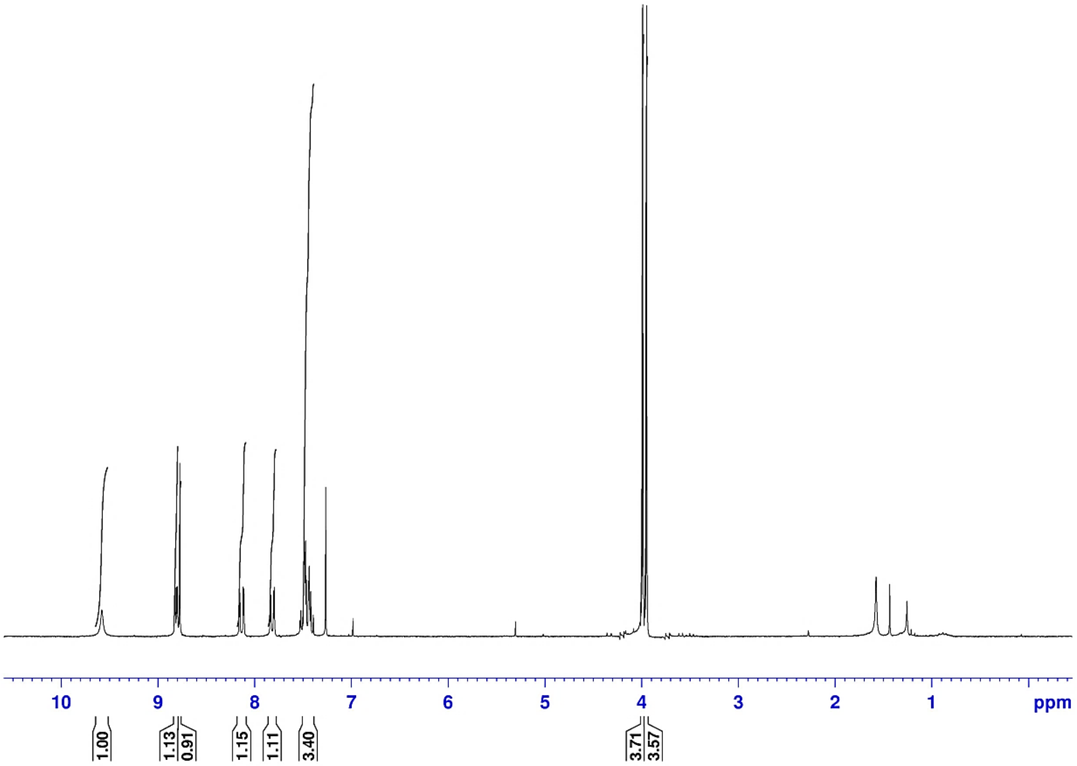

B2

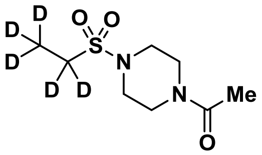

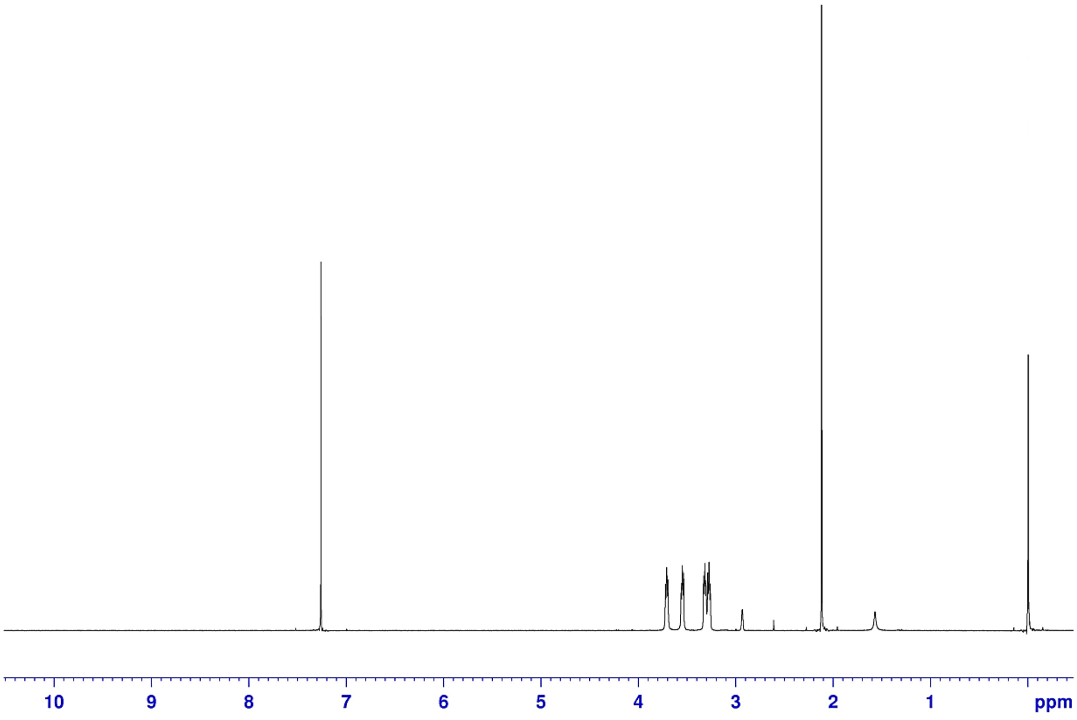

B6

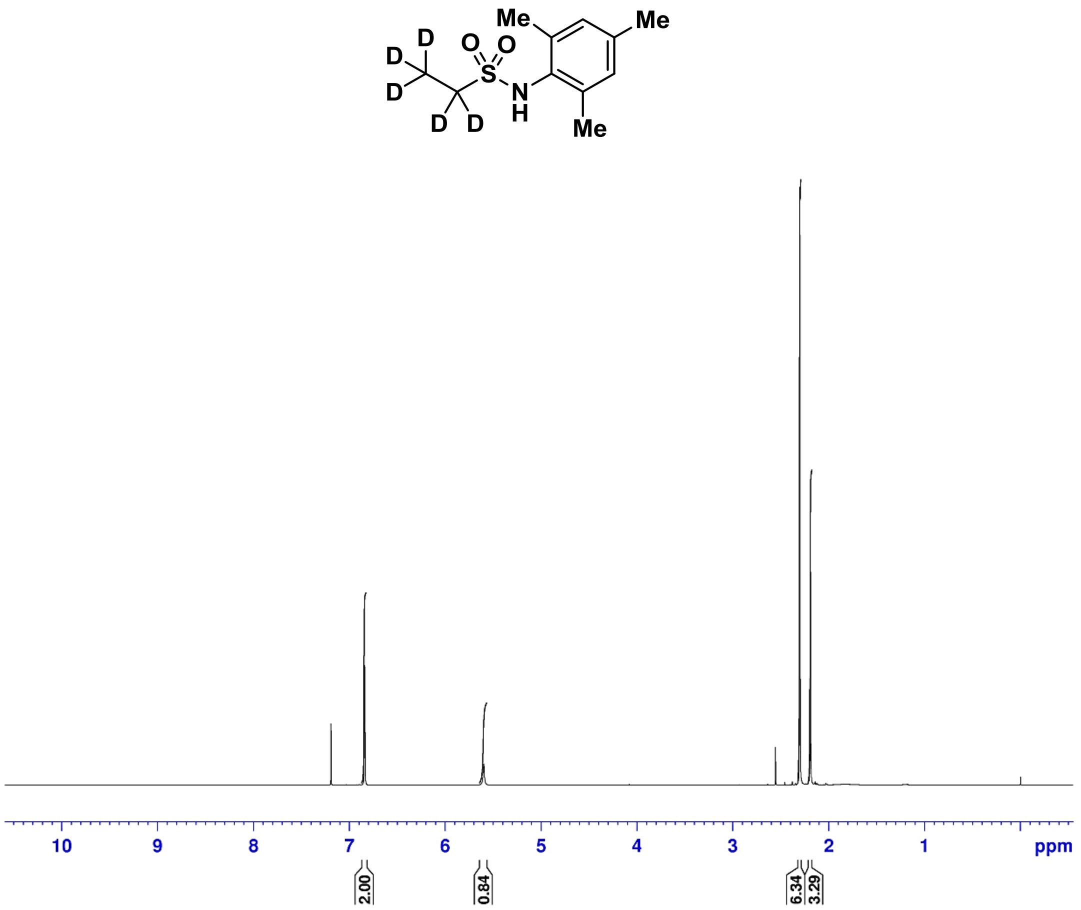
Me
Me
Me
D
D
D
D
D
O
O
S
N
H
10
9
8
7
6
5
4
3
2
1
ppm
2.00
0.84
6.34
3.29

B7

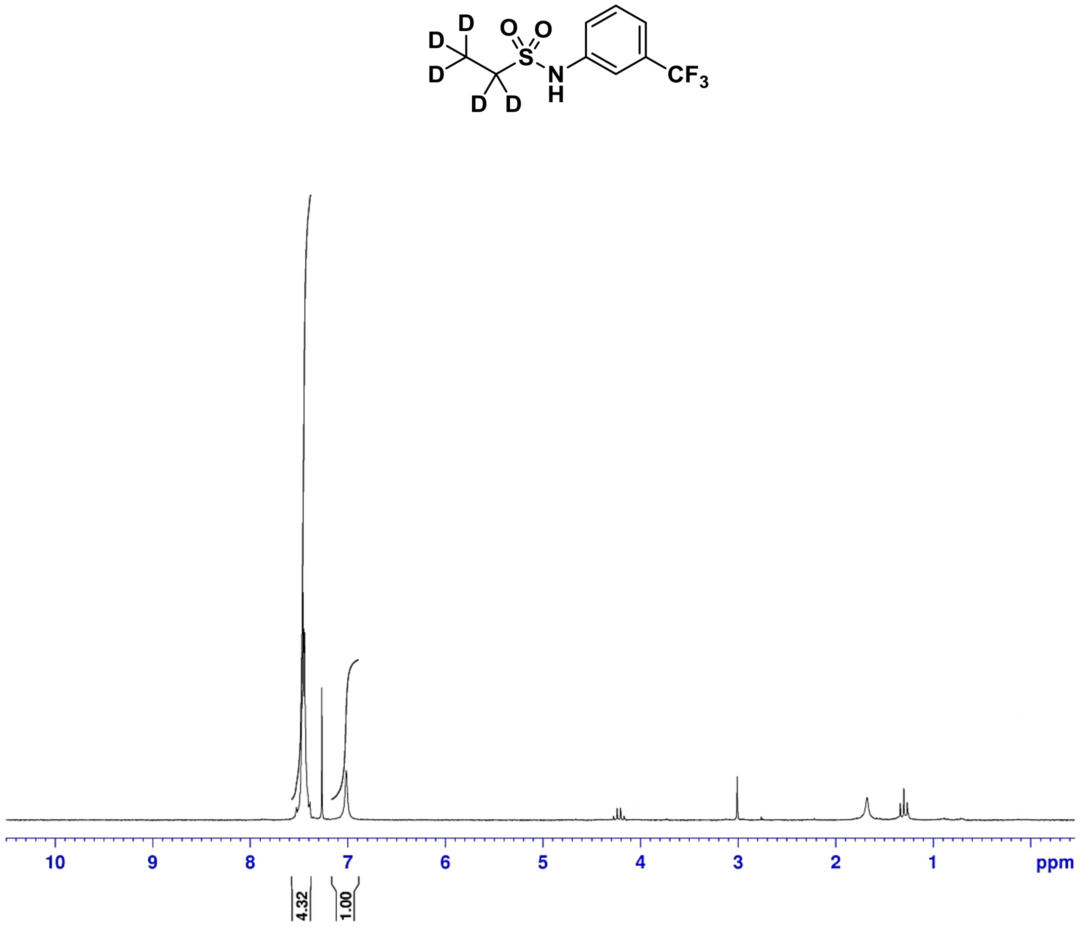
D
D
D
D
D
O
O
S
N
H
CF3
10
9
8
7
6
5
4
3
2
1
ppm
4.32
1.00

B8

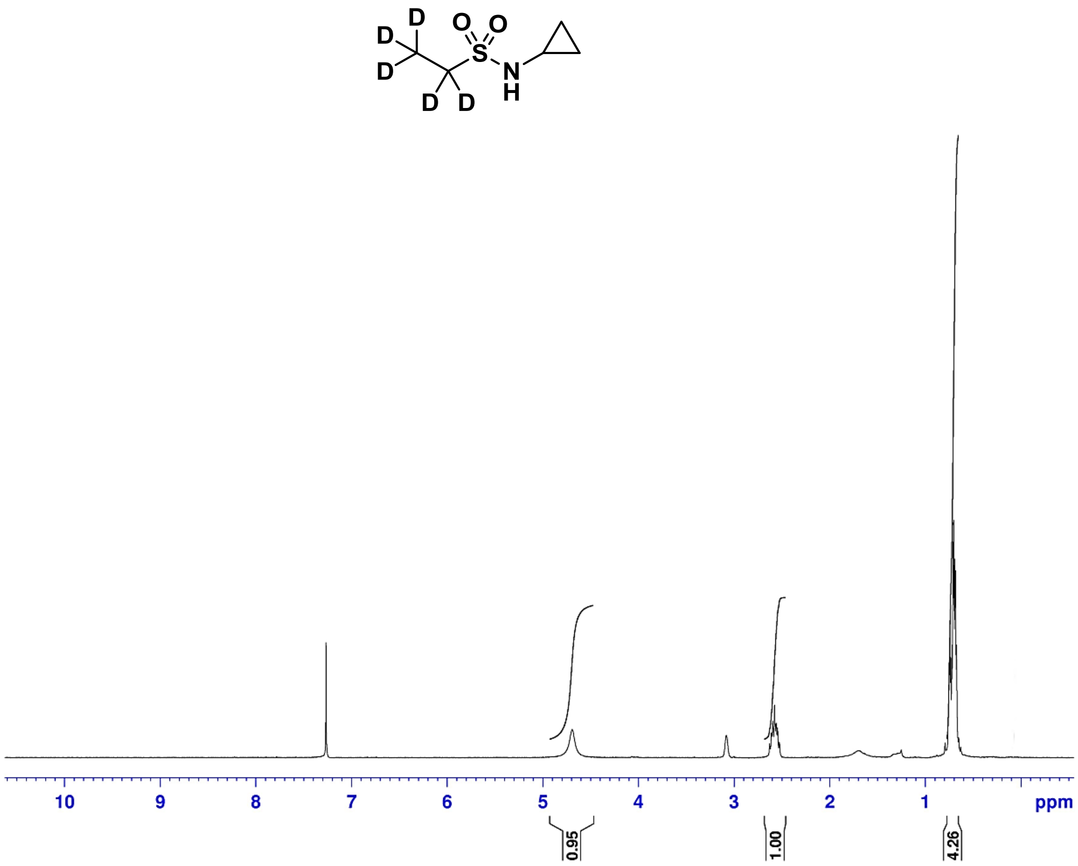

D
D
D
D
D
O
O
S
N
H
10
9
8
7
6
5
4
3
2
1
ppm
0.95
1.00
4.26

B9

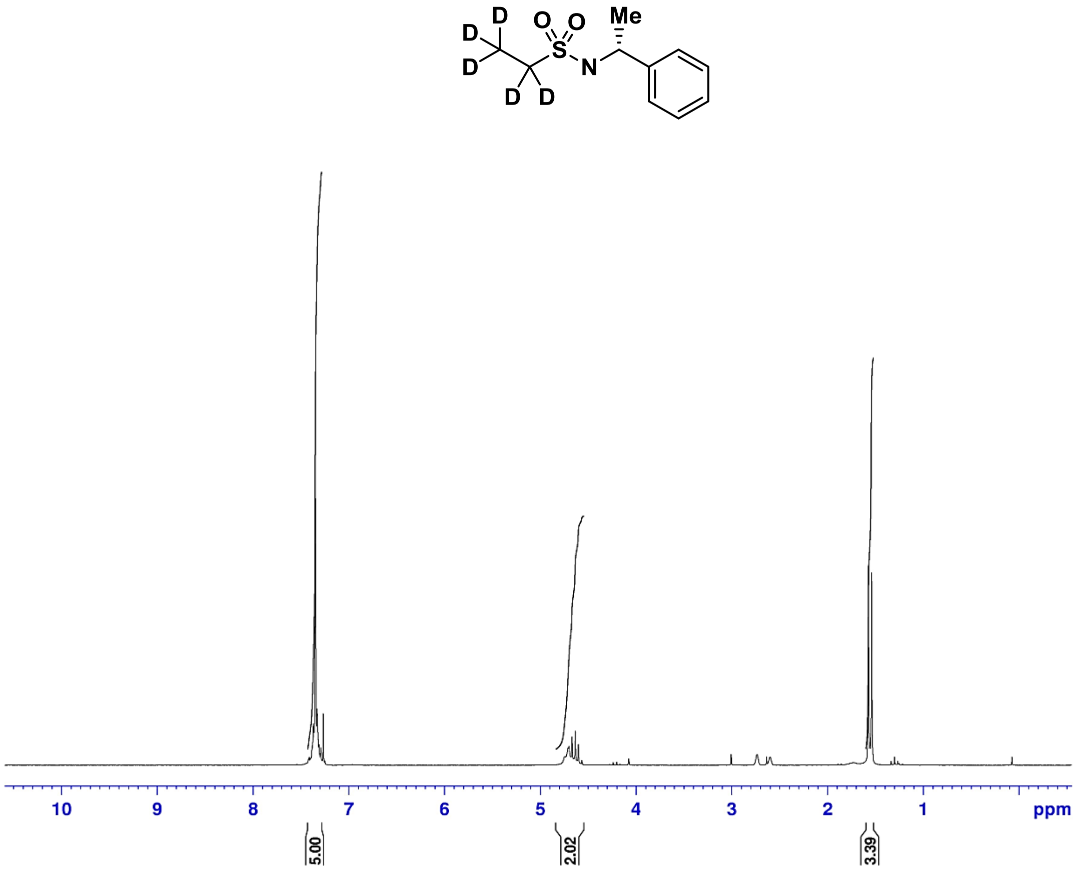

D
D
D
D
D
O
O
S
N
Me
10
9
8
7
6
5
4
3
2
1
ppm
5.00
2.02
3.39

D9

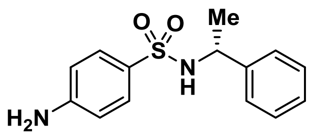

O
O
S
N
H
Me
H2N

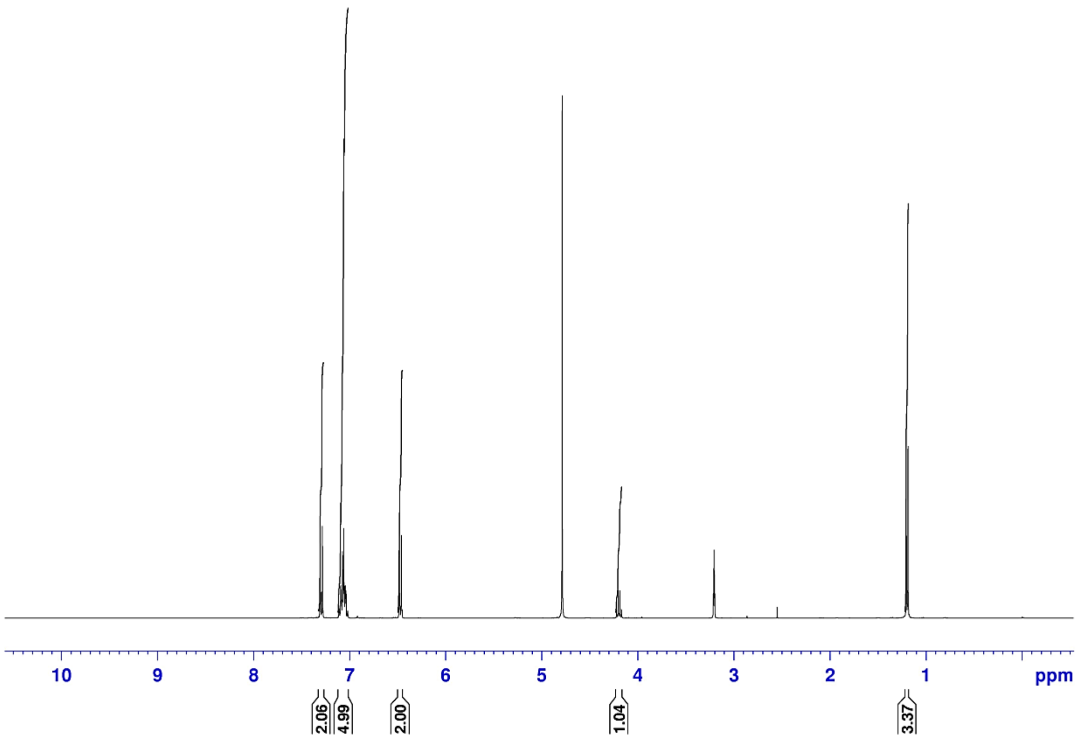

10
9
8
7
6
5
4
3
2
1
ppm
2.06
4.99
2.00
1.04
3.37

E2

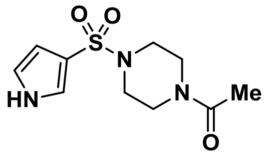

O
O
S
N
N
Me
O
HN

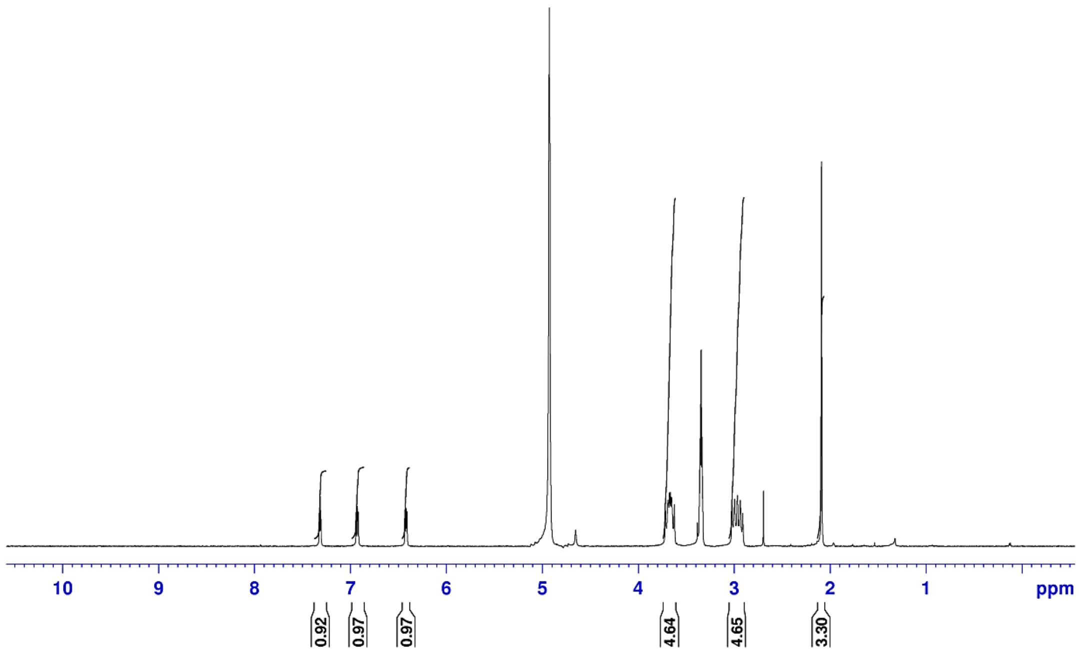

10
9
8
7
6
5
4
3
2
1
ppm
0.92
0.97
0.97
4.64
4.65
3.30

E8

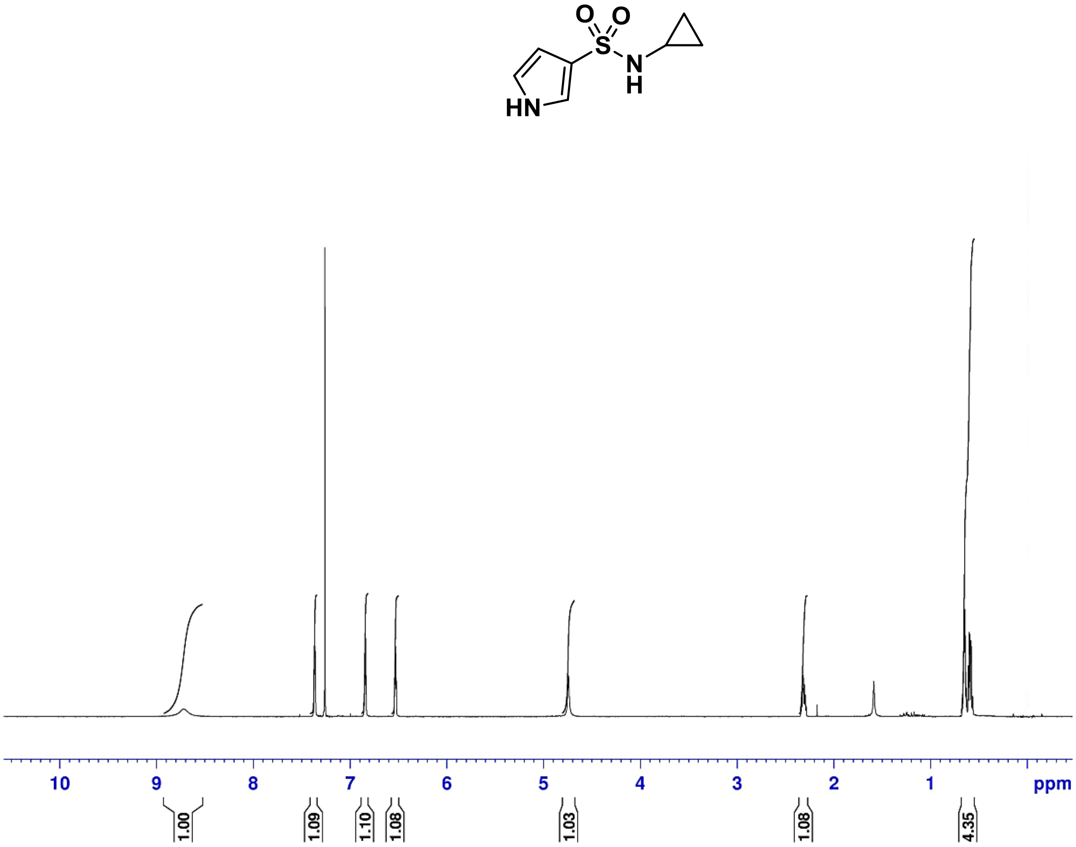

O O
S
N
H
HN
10 9 8 7 6 5 4 3 2 1 ppm
1.00
1.09
1.10
1.08
1.03
1.08
4.35

E9

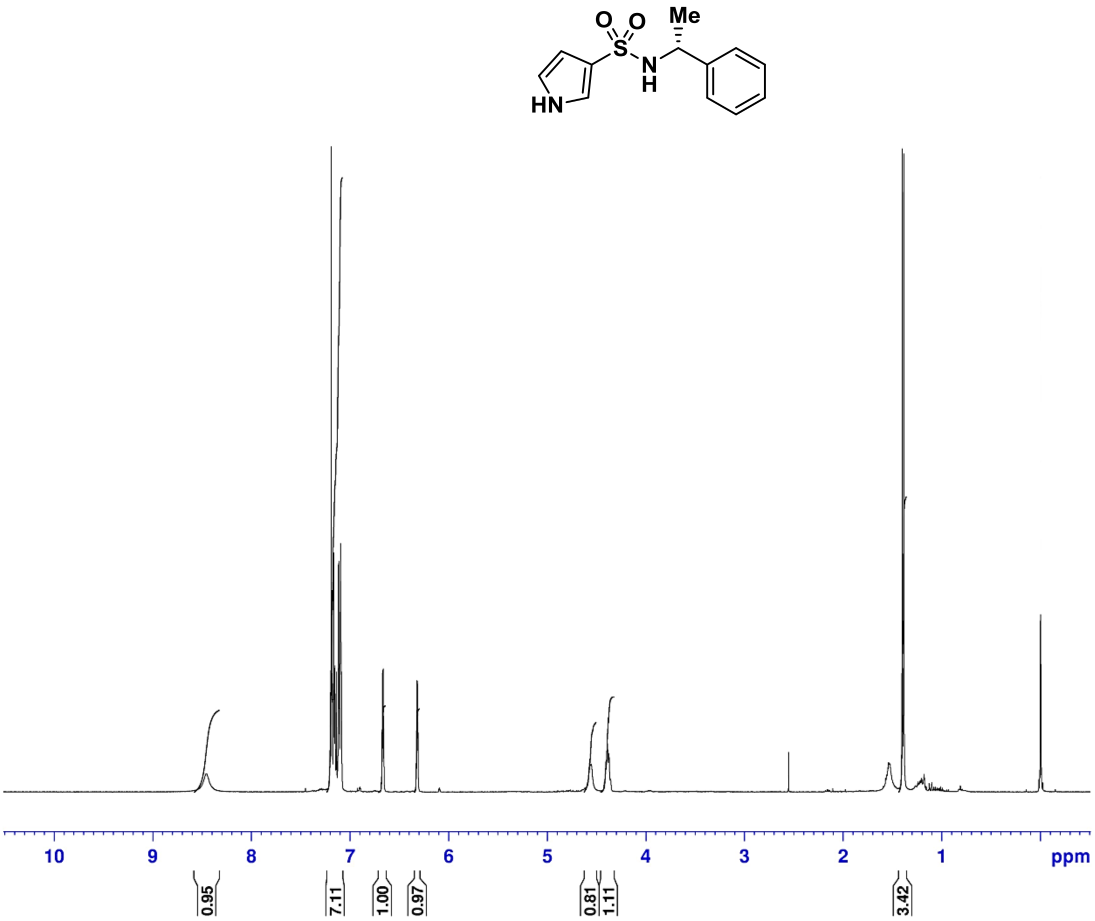

O O
S
Me
N
H
HN
10 9 8 7 6 5 4 3 2 1 ppm
0.95
7.11
1.00
0.97
0.81
1.11
3.42

E10

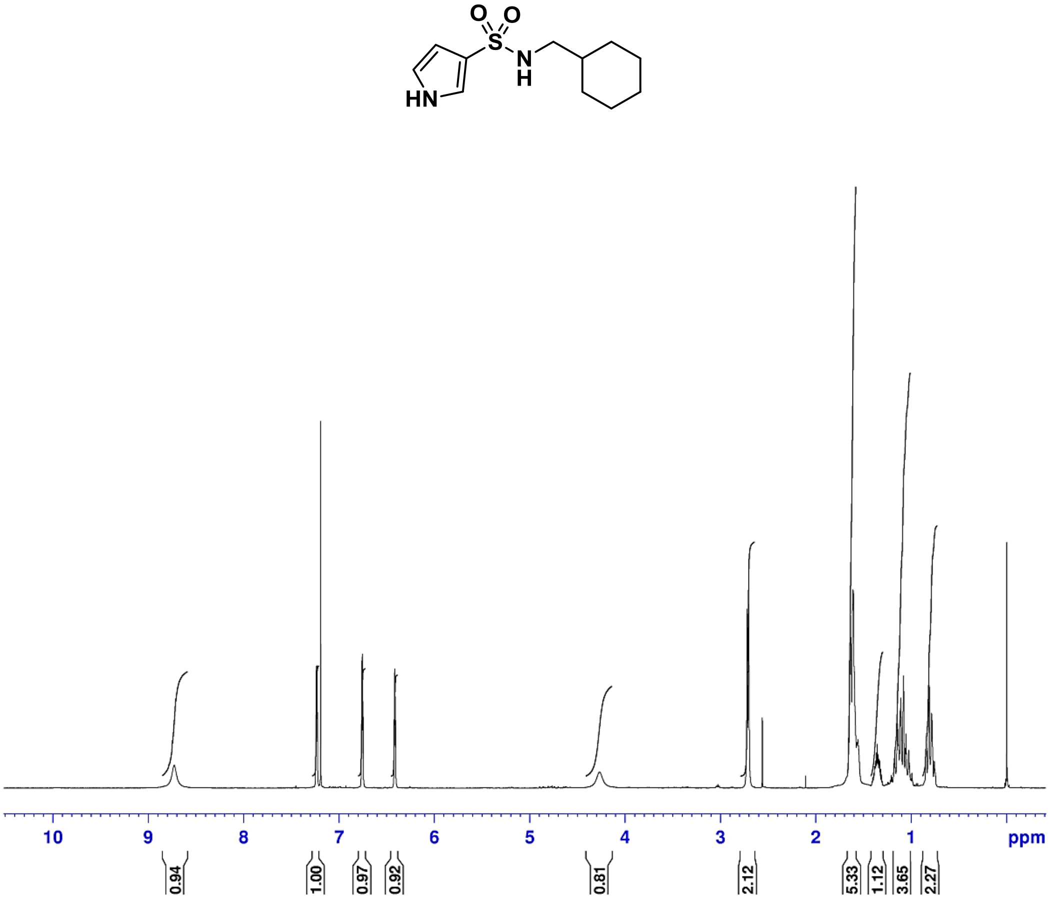
O O
S
N
H
HN
10 9 8 7 6 5 4 3 2 1 ppm
0.94 1.00 0.97 0.92 0.81 2.12 5.33 1.12 3.65 2.27

F2

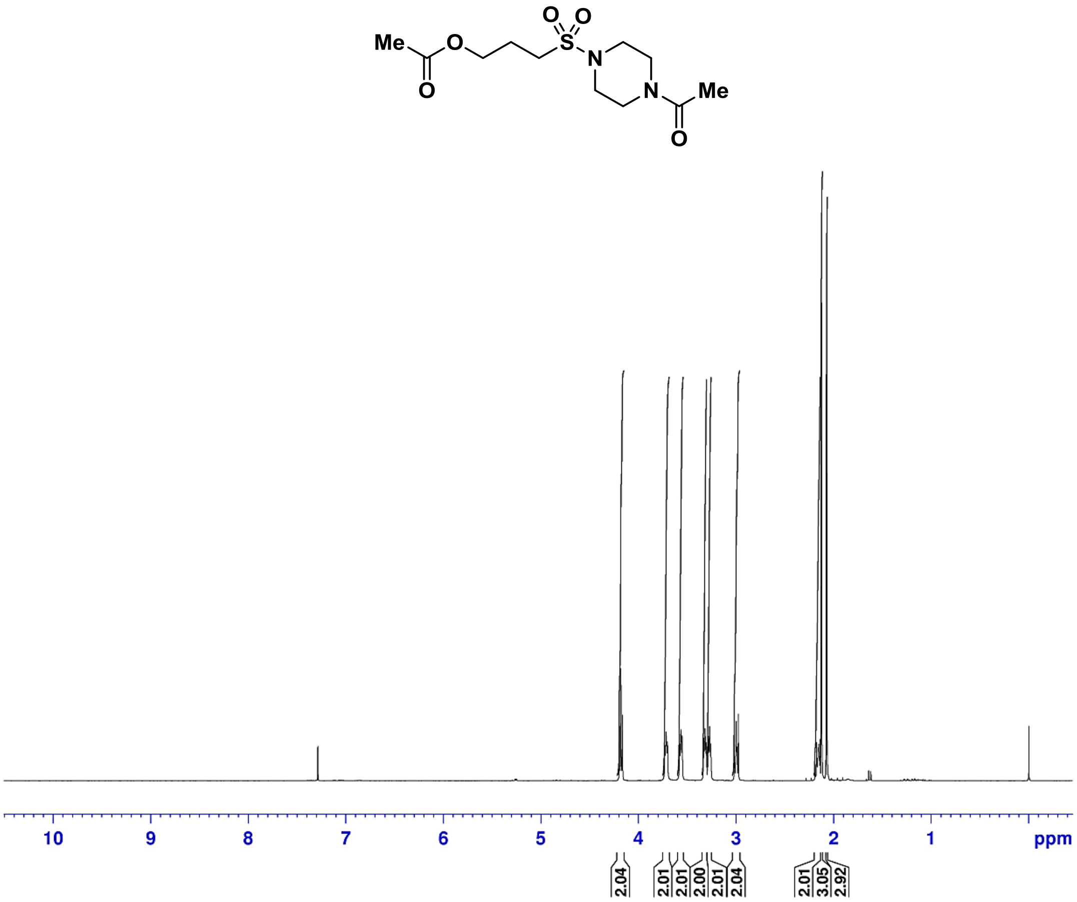
Me
O
O
O O
S
N
N
Me
O
10 9 8 7 6 5 4 3 2 1 ppm
2.04 2.01 2.01 2.00 2.01 2.04 2.01 3.05 2.92

F6

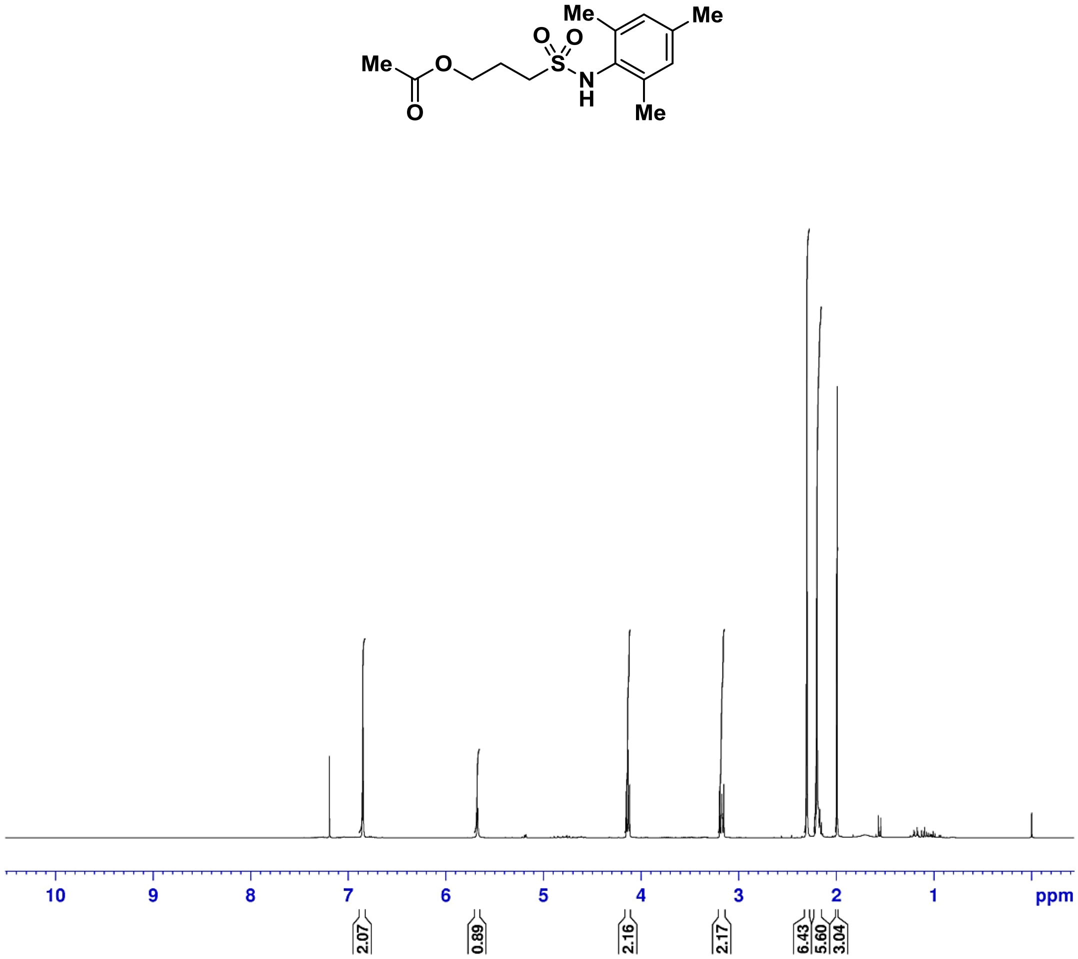
Me
Me
O O
Me O S N
H
O Me
10 9 8 7 6 5 4 3 2 1 ppm
2.07
0.89
2.16
2.17
6.43
5.60
3.04

F7

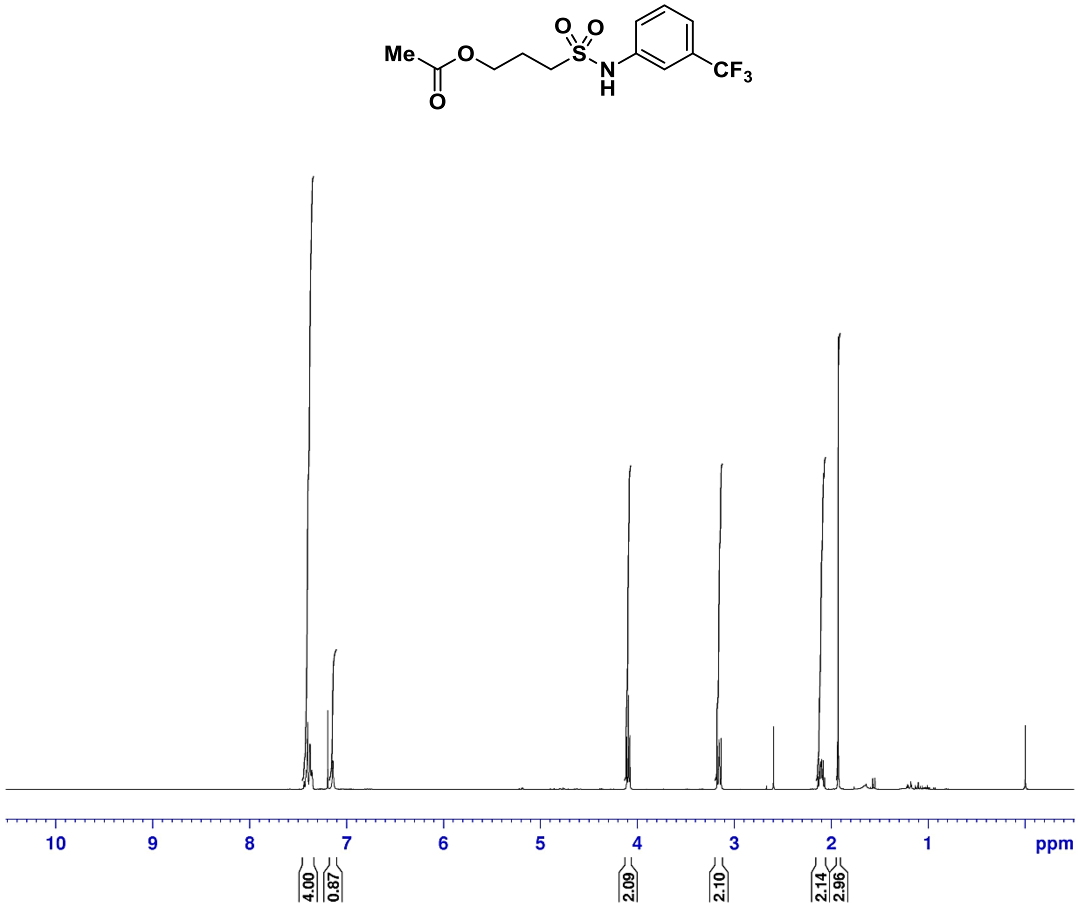
O O
Me O S N CF3
H
O
10 9 8 7 6 5 4 3 2 1 ppm
4.00
0.87
2.09
2.10
2.14
2.96

G8

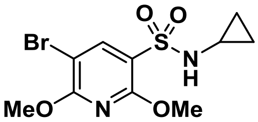

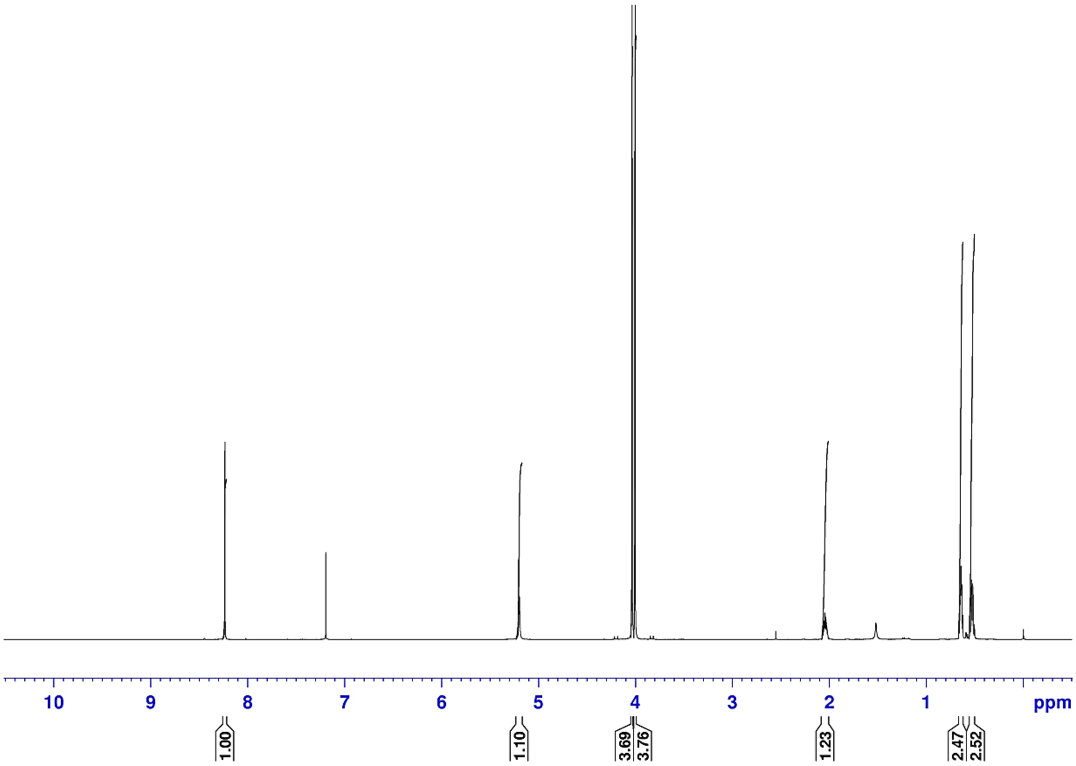

File:13zp545l1 Vial:5:50 ID: **A1**

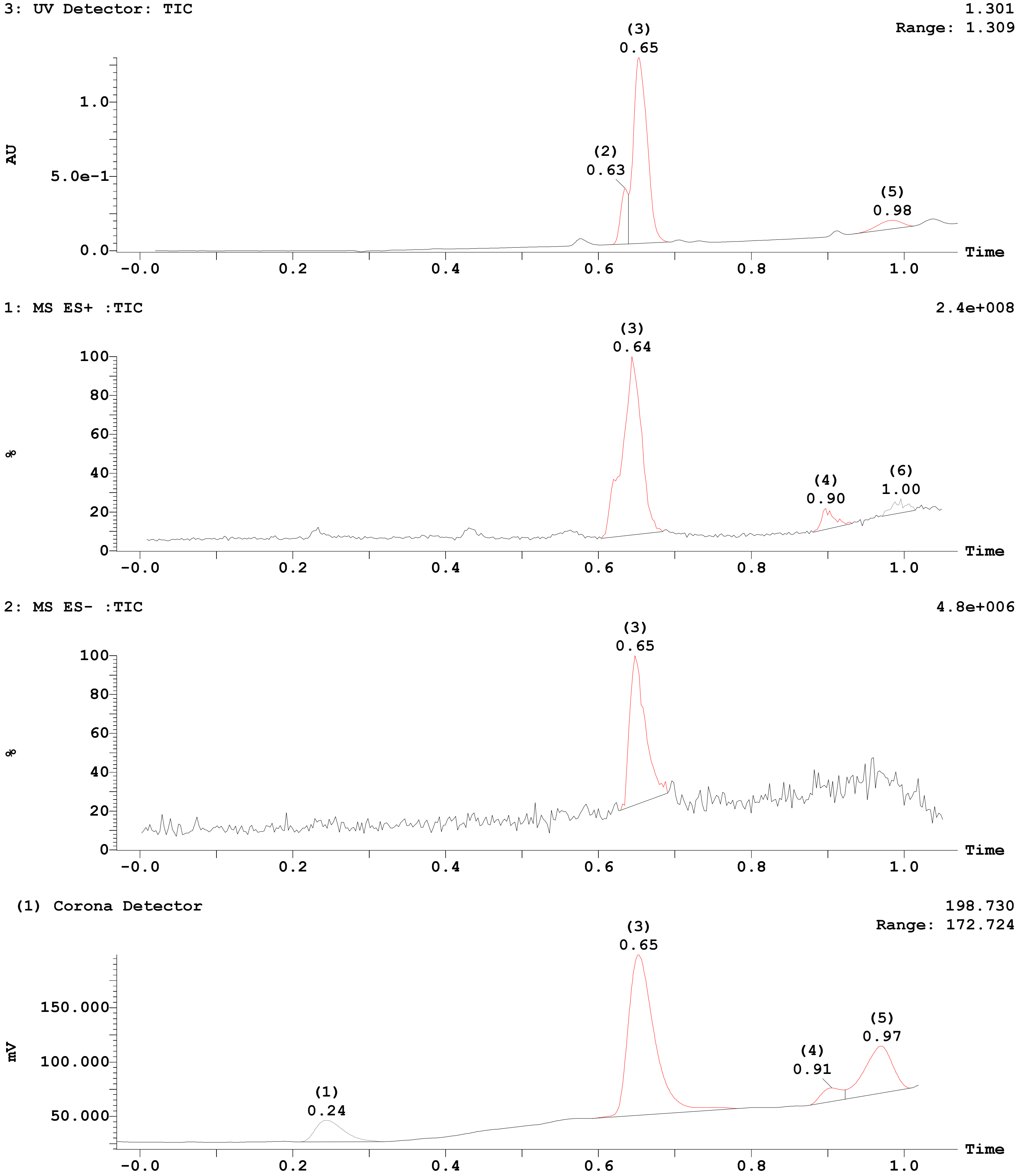

Peak ID **Time**
2 0.63

2:(Time: 0.63) Combine (231:246-(149:157+315:323)) 1:MS ES+ 5.5e+007

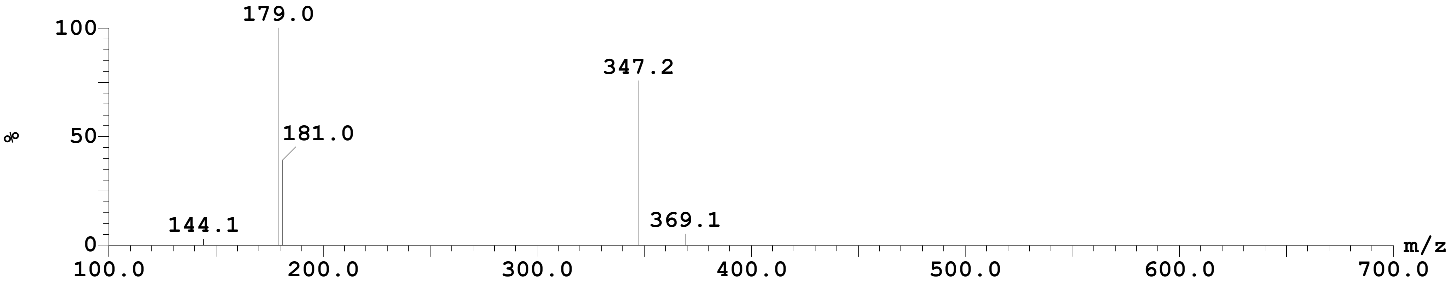

Peak ID **Time**
2 0.63

2:(Time: 0.63) Combine (231:246-(149:156+315:322)) 2:MS ES- 9.3e+005

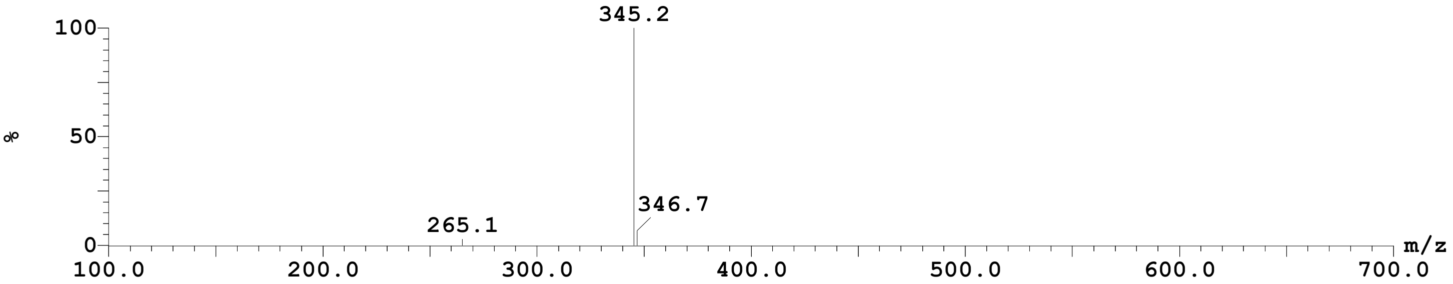

Peak ID **Time**
3 0.64

3:(Time: 0.64) Combine (234:249-(145:152+332:339)) 1:MS ES+ 5.3e+007

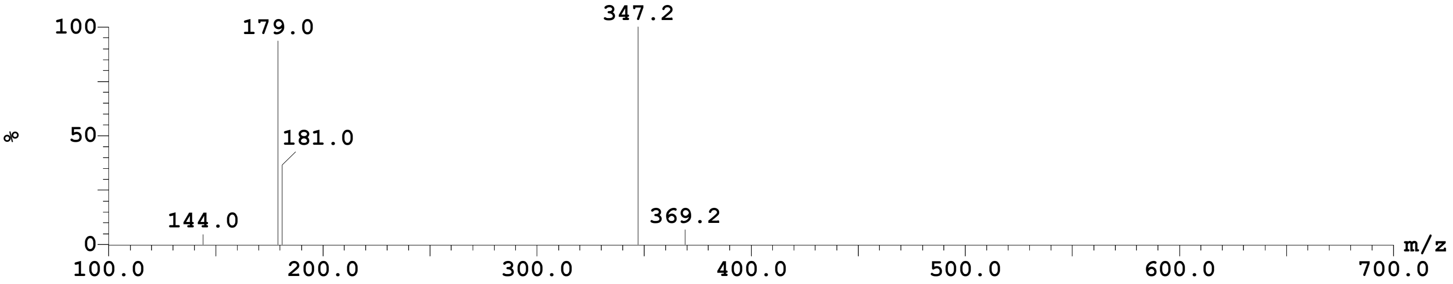

Peak ID **Time**
3 0.64

3:(Time: 0.65) Combine (235:250-(154:161+334:341)) 2:MS ES- 1.1e+006

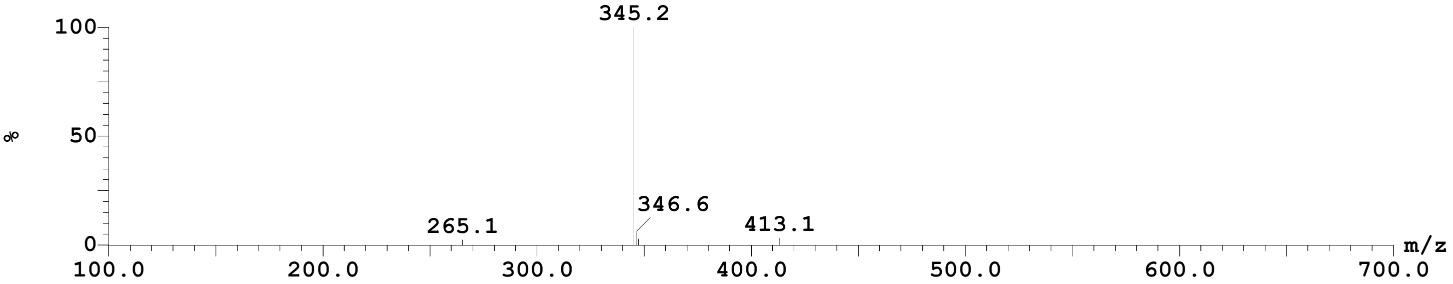

File:13zp545l1 Vial:5:50 ID:A1
Method:C:\MASSLYNX\1minLC_MS.olp

Peak ID **Time**
4 0.90

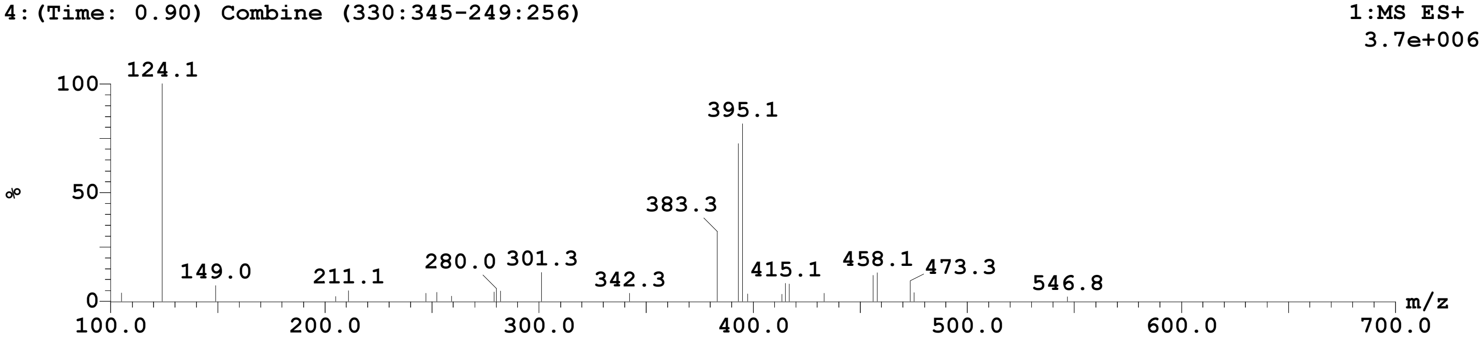

Peak ID **Time**
5 0.98

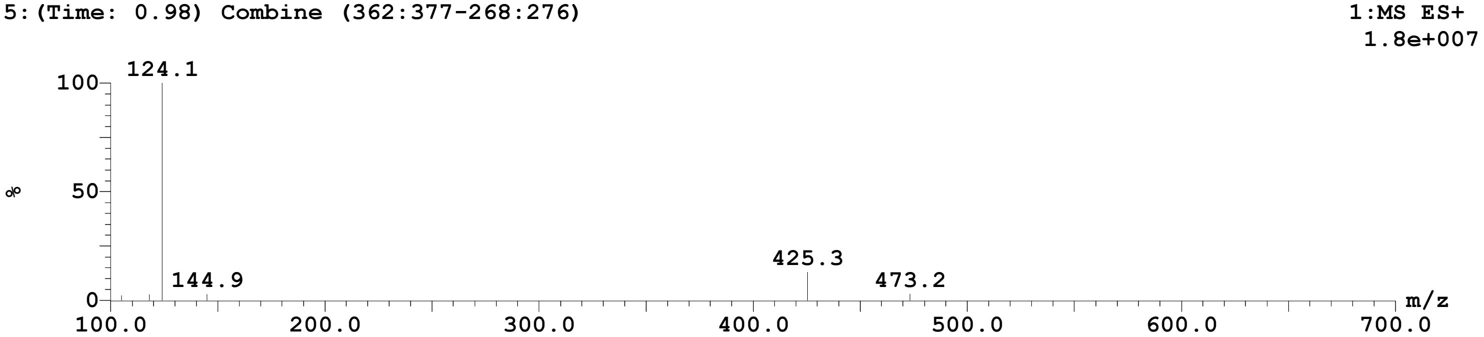

Peak ID **Time**
5 0.98

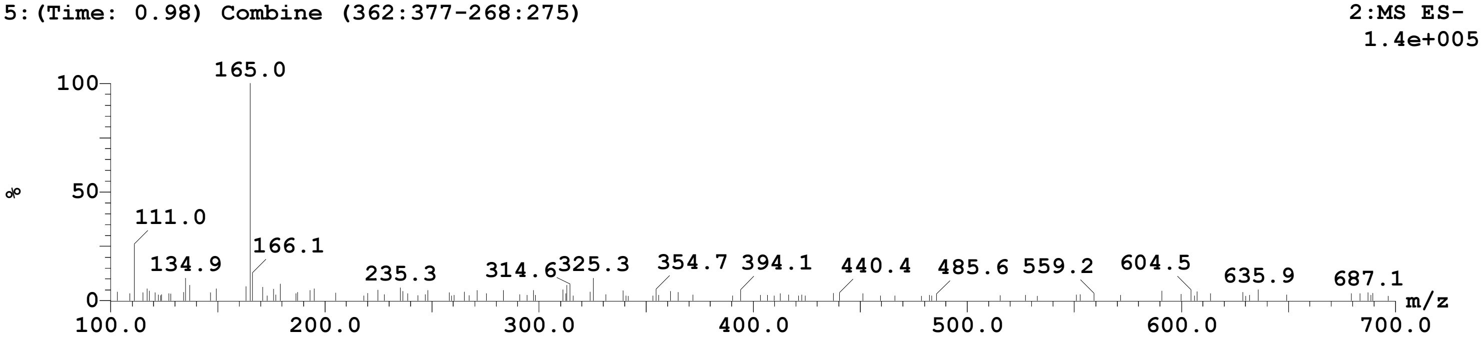

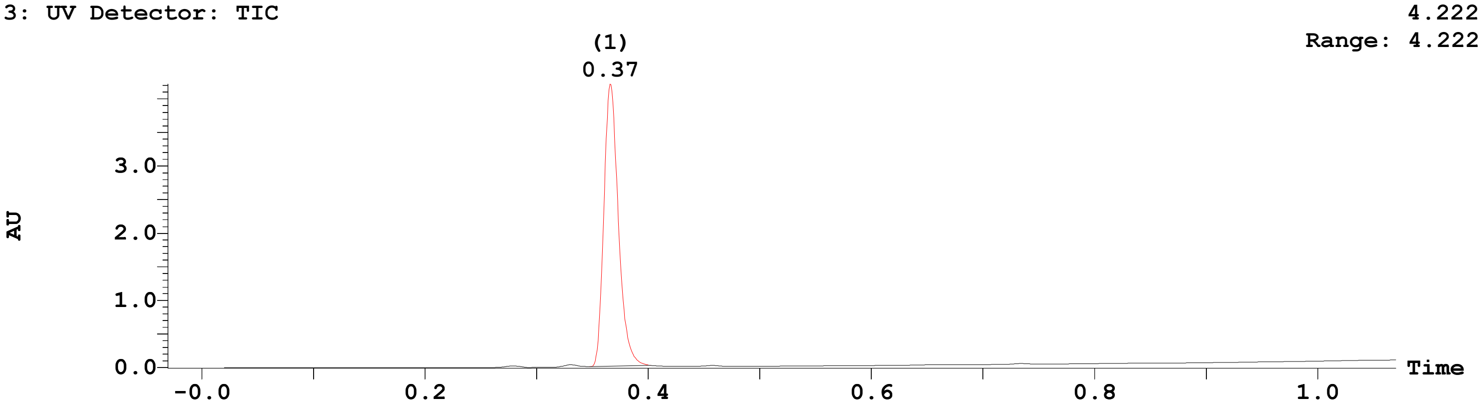
3: UV Detector: TIC
4.222
Range: 4.222
(1)
0.37
AU
3.0
2.0
1.0
0.0
-0.0
0.2
0.4
0.6
0.8
1.0
Time

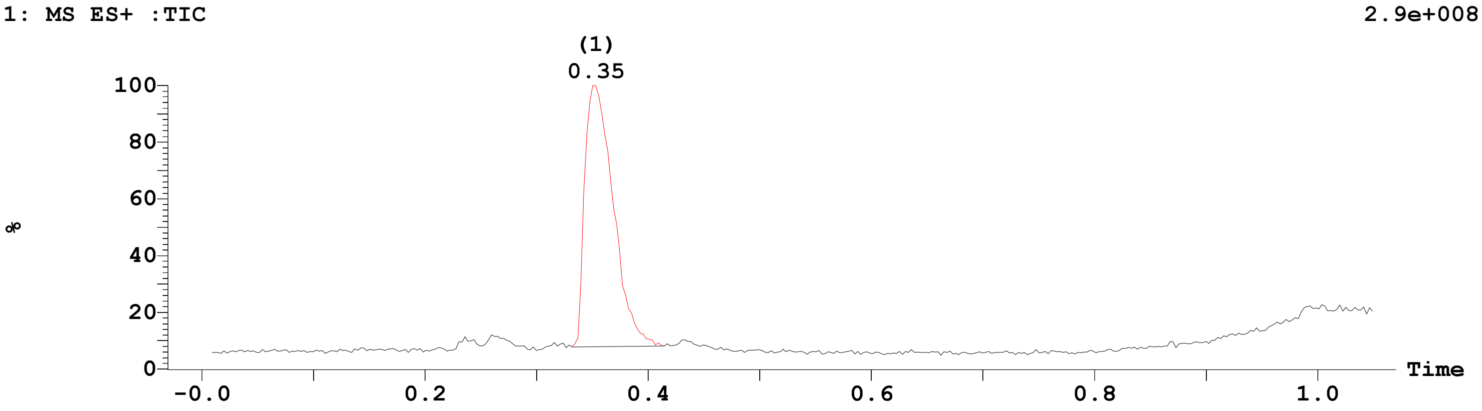
1: MS ES+ :TIC
2.9e+008
(1)
0.35
%
100
80
60
40
20
0
-0.0
0.2
0.4
0.6
0.8
1.0
Time

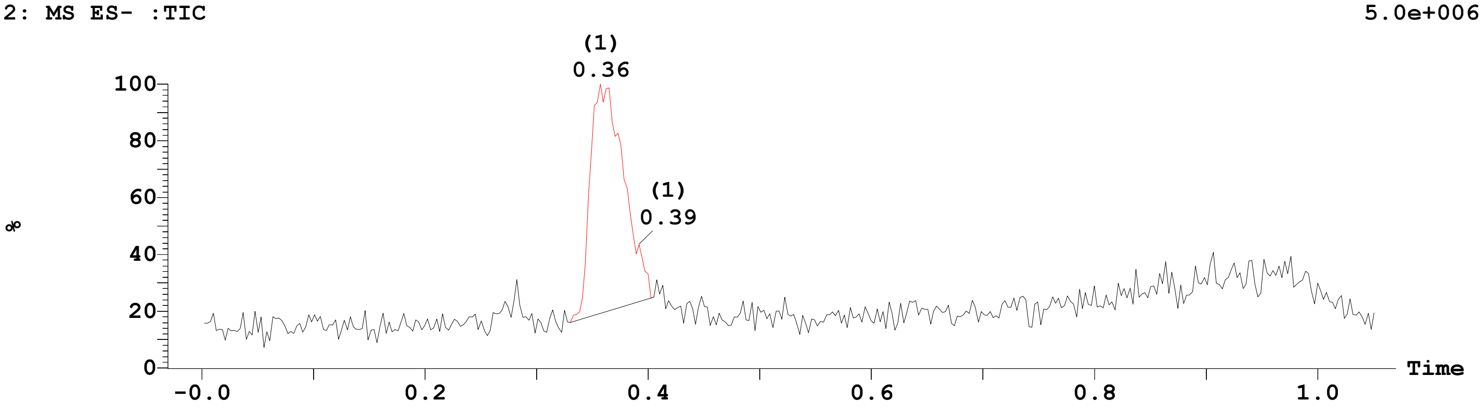
2: MS ES- :TIC
5.0e+006
(1)
0.36
(1)
0.39
%
100
80
60
40
20
0
-0.0
0.2
0.4
0.6
0.8
1.0
Time

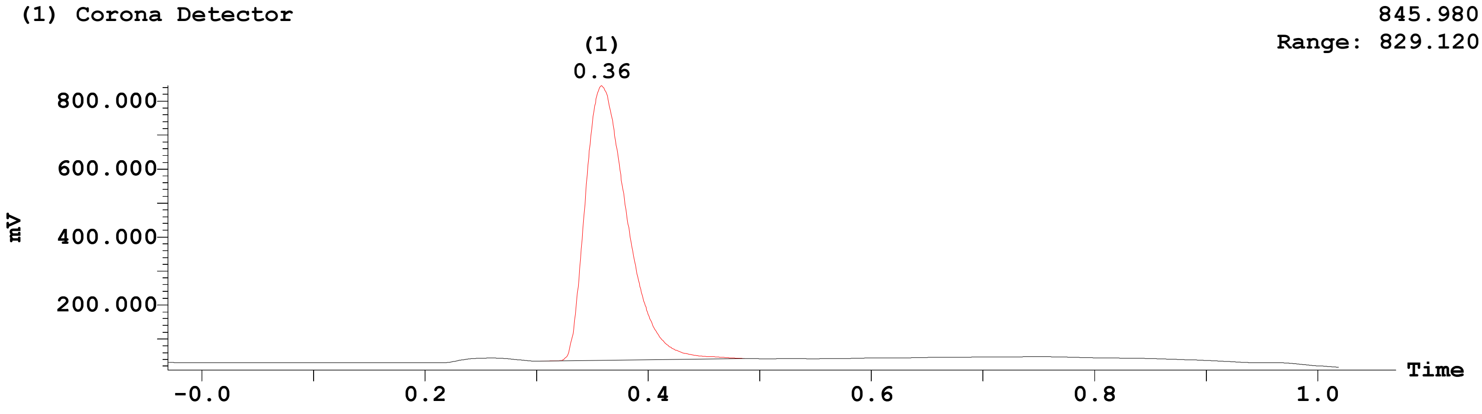
(1) Corona Detector
845.980
Range: 829.120
(1)
0.36
mV
800.000
600.000
400.000
200.000
-0.0
0.2
0.4
0.6
0.8
1.0
Time

File:13zn320l3 Vial:5:51 ID:A2
Method:C:\MASSLYNX\1minLC_MS.olp

Peak ID **Time**
1 0.35

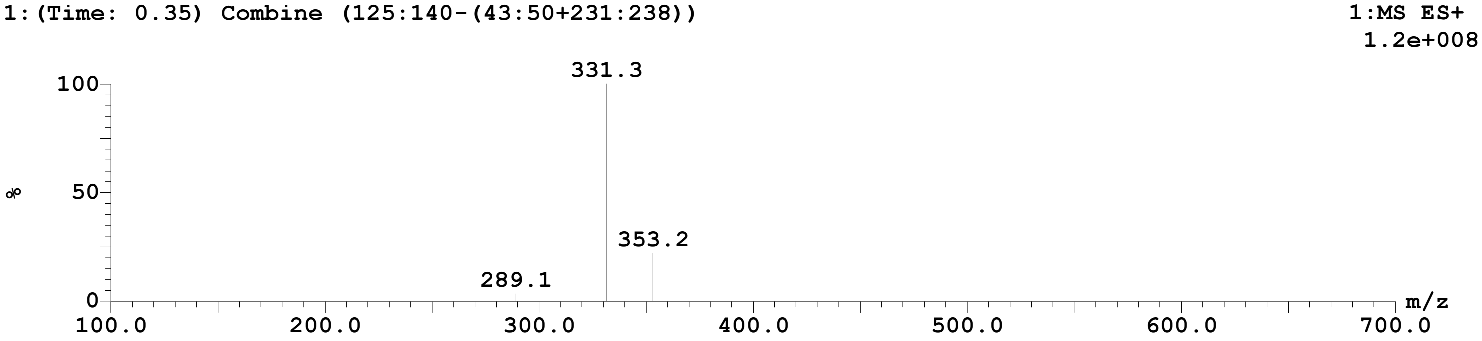

Peak ID **Time**
1 0.35

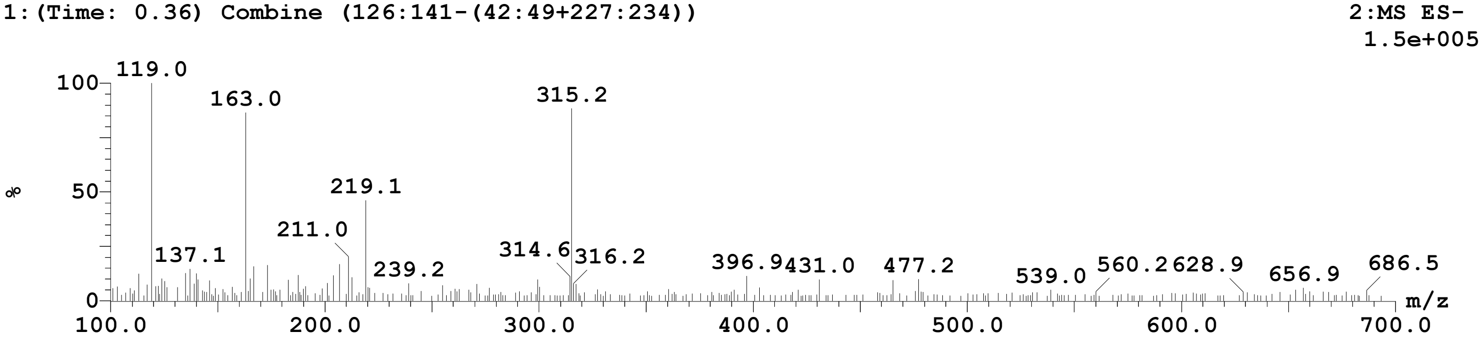

File:13zo525l2 Vial:5:52 ID: **A3**

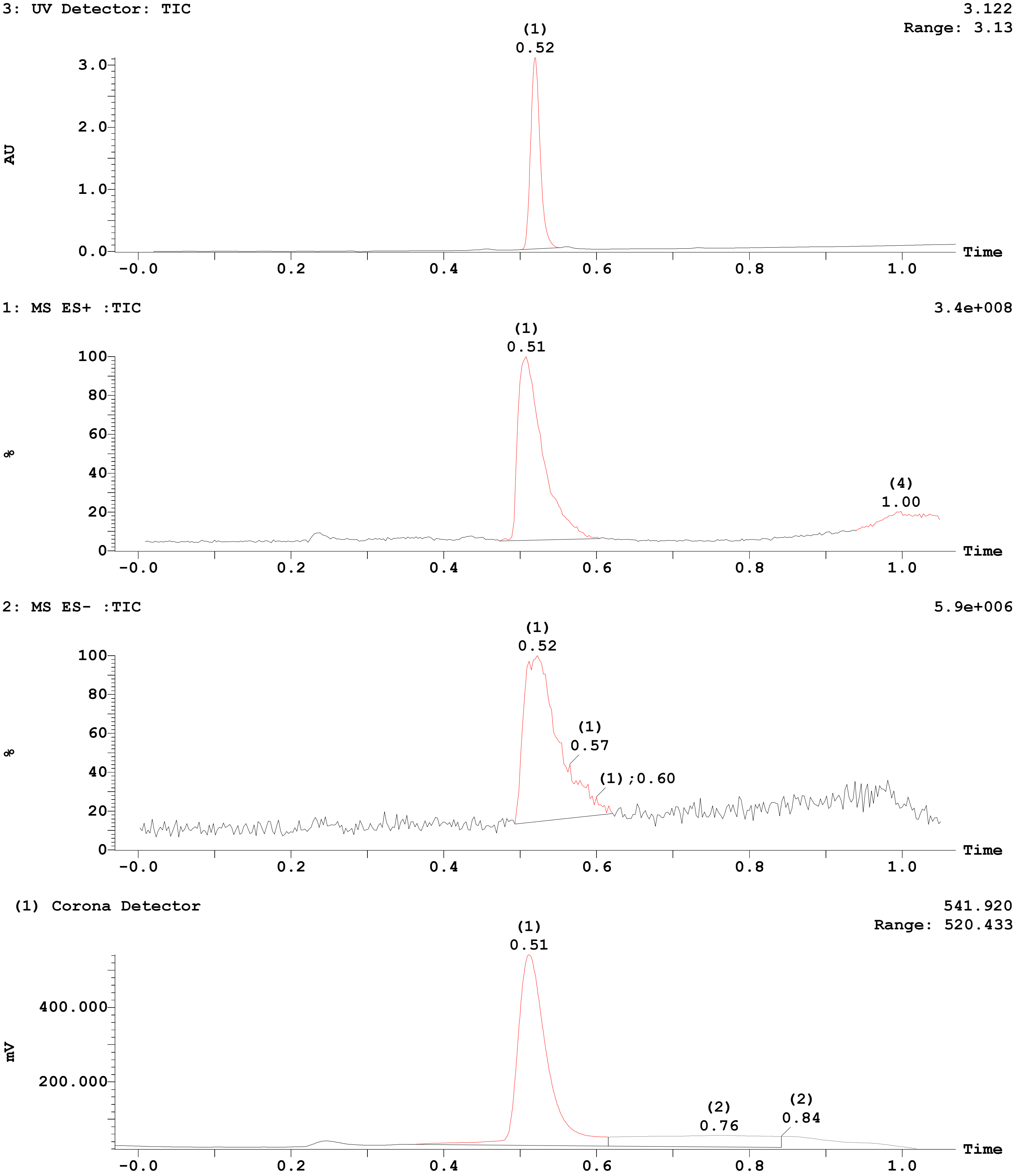

File:13zo525l2 Vial:5:52 ID:A3
Method:C:\MASSLYNX\1minLC_MS.olp

Peak ID **Time**
1 0.51

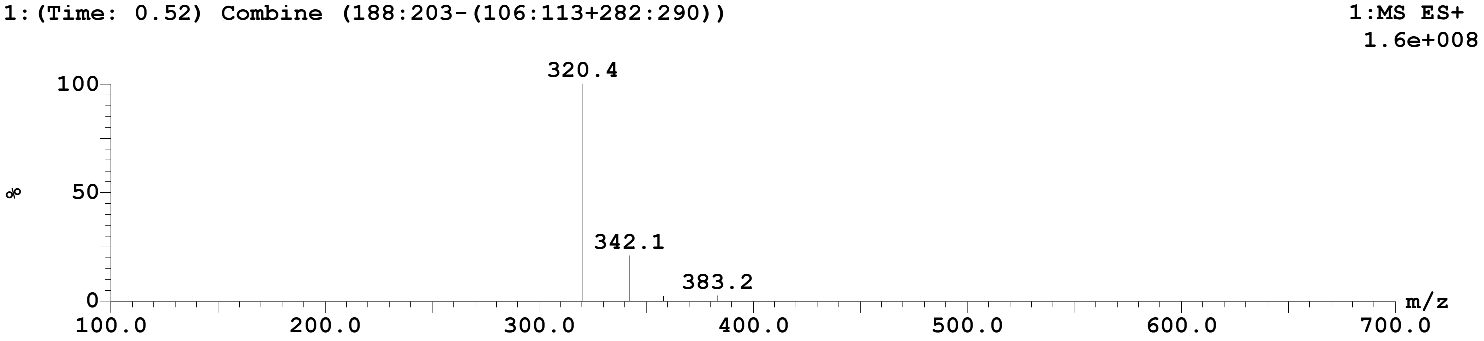

Peak ID **Time**
1 0.51

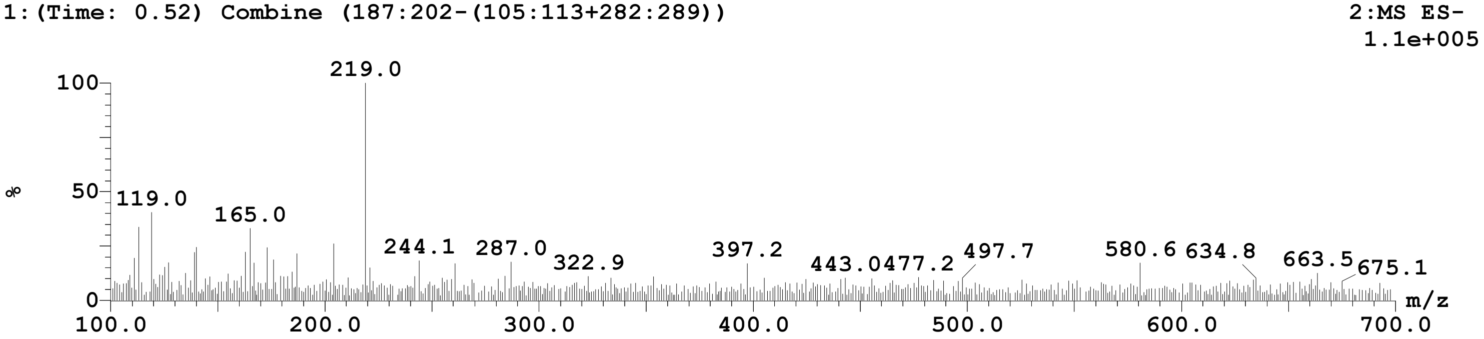

Peak ID **Time**
4 1.00

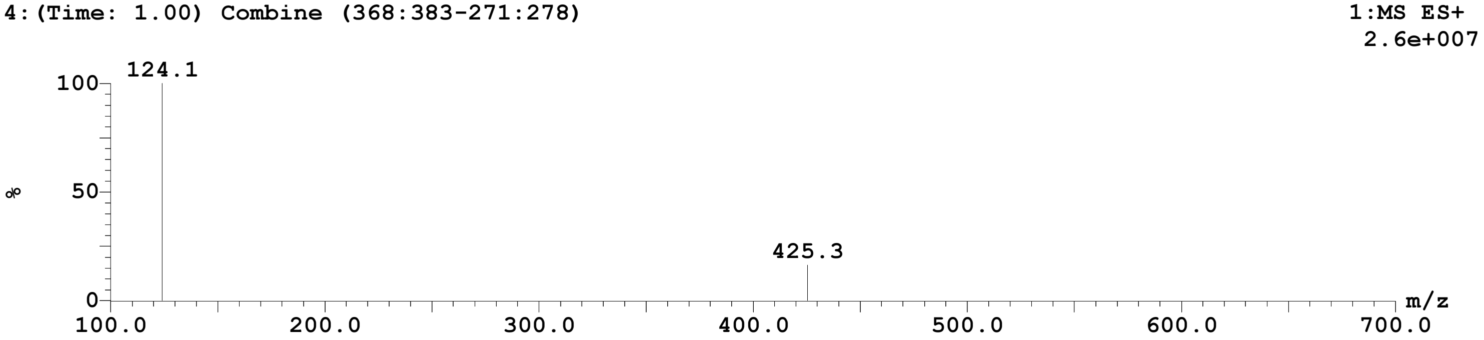

SYNGENTA SQD OPEN ACCESS LC-MS Page 1
File:13zp569l1 Vial:5:53 ID: **A4**

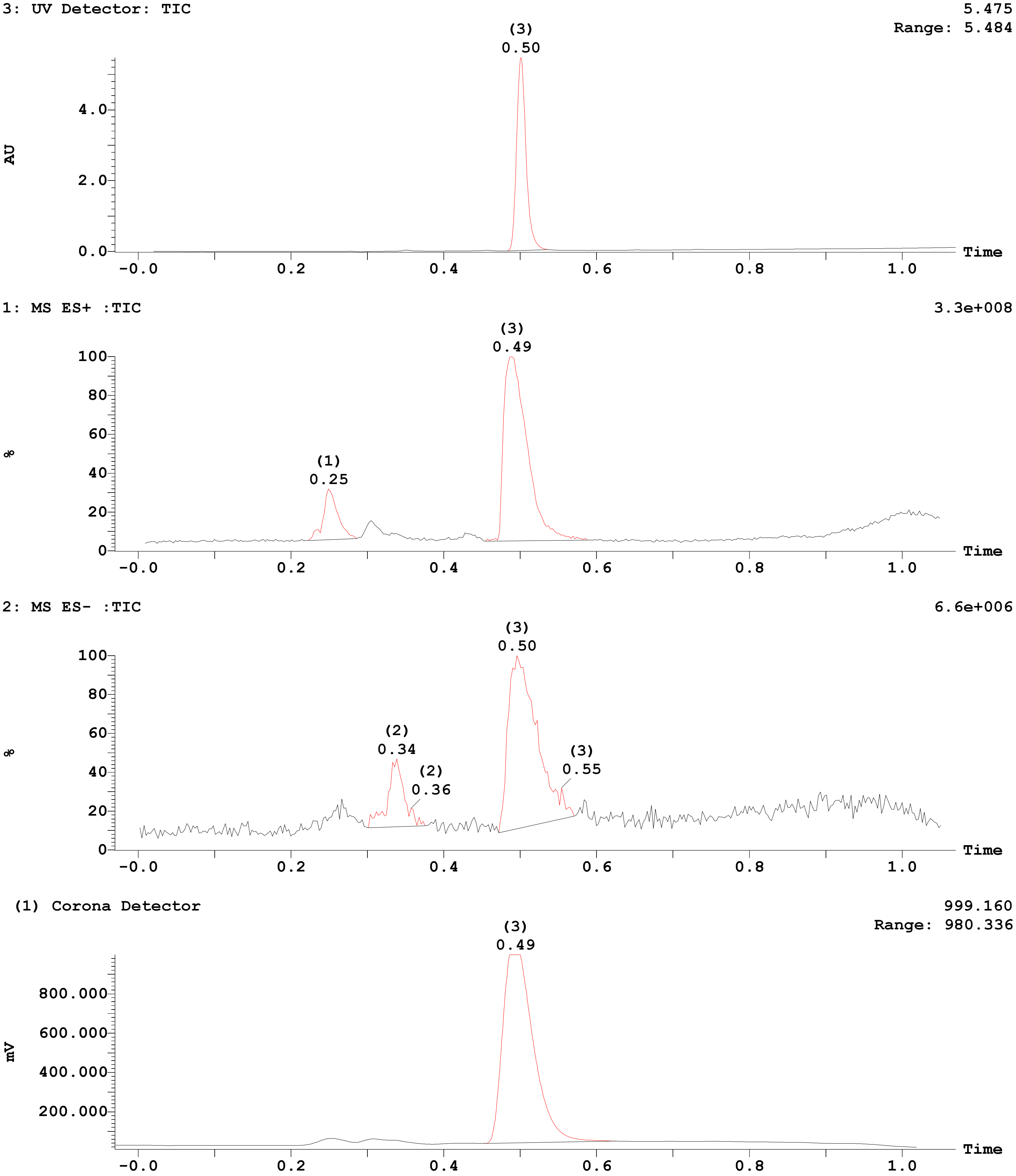

File:13zp569l1 Vial:5:53 ID:A4
Method:C:\MASSLYNX\1minLC_MS.olp

Peak ID **Time**
1 0.25

1:(Time: 0.25) Combine (87:101-(2:9+183:190)) 1:MS ES+
1.0e+007

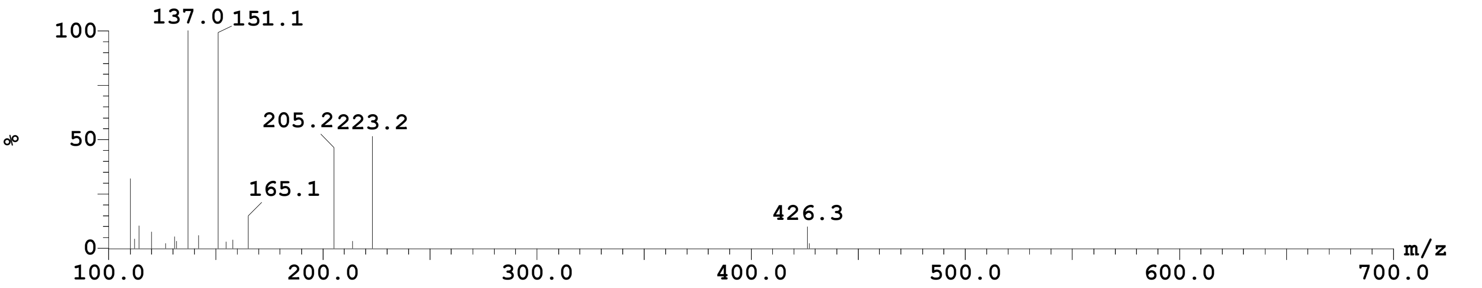

Peak ID **Time**
2 0.34

2:(Time: 0.34) Combine (119:134-(31:38+216:223)) 2:MS ES-
7.2e+005

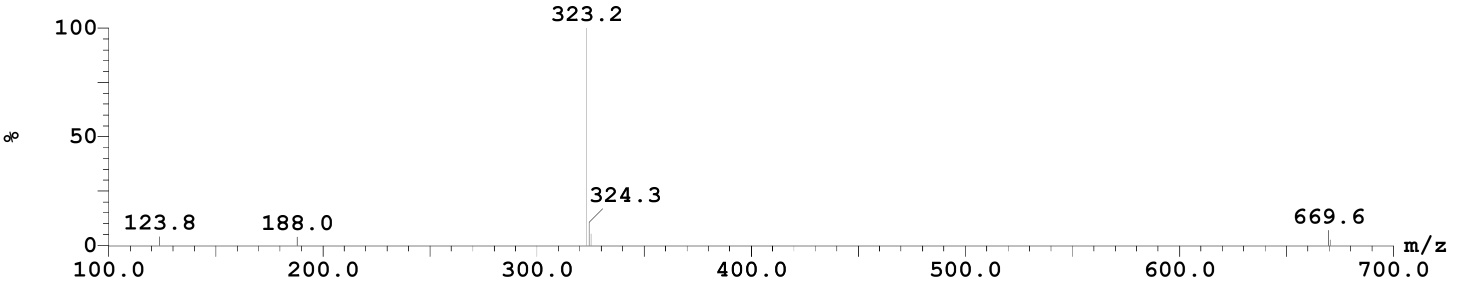

Peak ID **Time**
3 0.49

3:(Time: 0.50) Combine (181:196-(99:107+277:284)) 1:MS ES+
1.6e+008

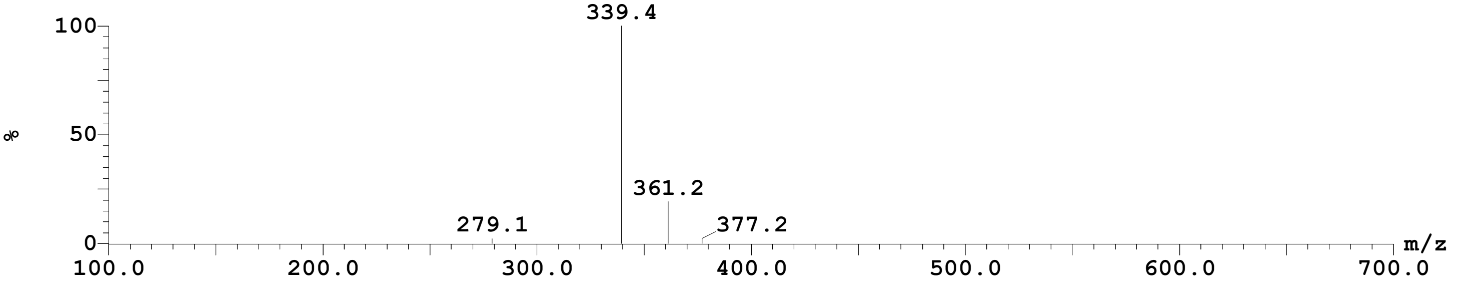

Peak ID **Time**
3 0.49

3:(Time: 0.50) Combine (180:195-(99:106+276:284)) 2:MS ES-
2.2e+005

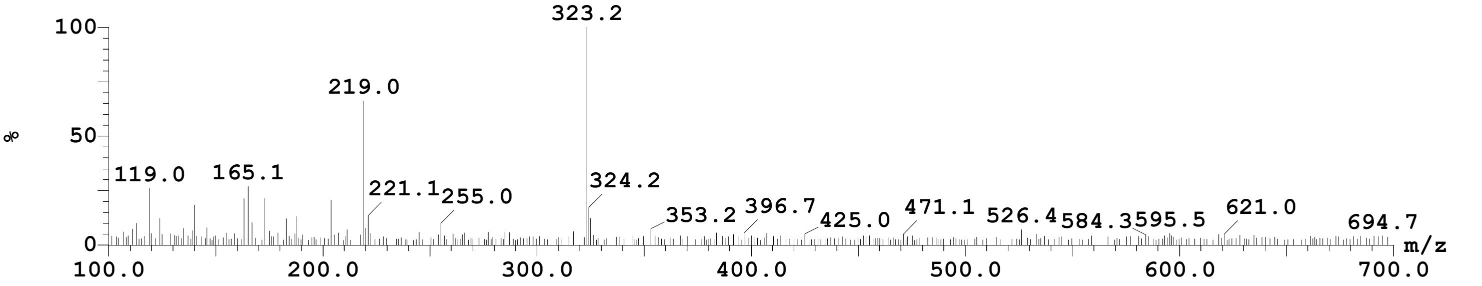

SYNGENTA SQD OPEN ACCESS LC-MS Page 1

File:13zn226l4 Vial:5:2 ID: **A5**

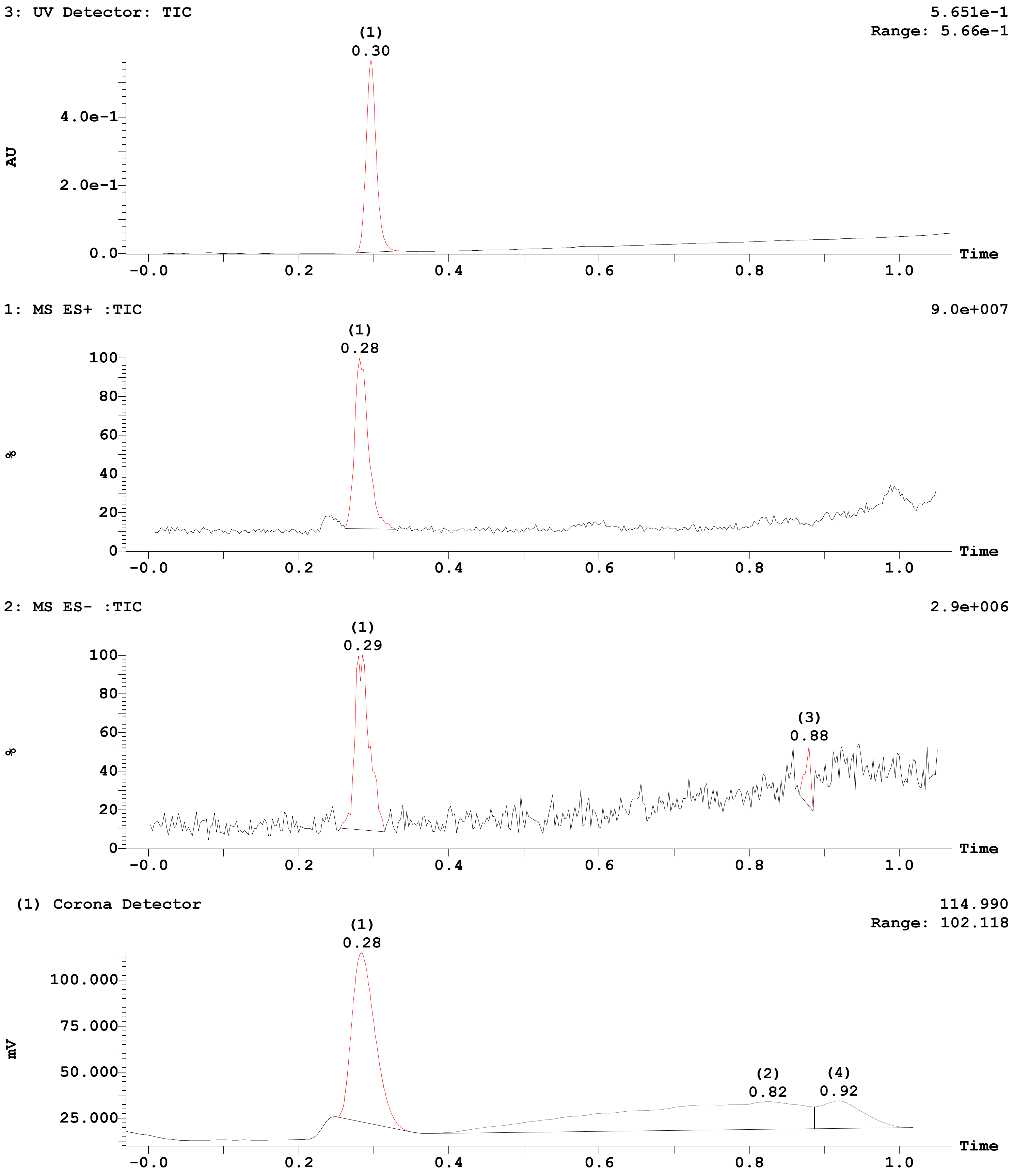

File:13zn226l4 Vial:5:2 ID:A5
Method:C:\MASSLYNX\1minLC_MS.olp

Peak ID	Time
1	0.28

1:(Time: 0.28) Combine (98:113-(17:24+199:206)) 1:MS ES+ 3.2e+007

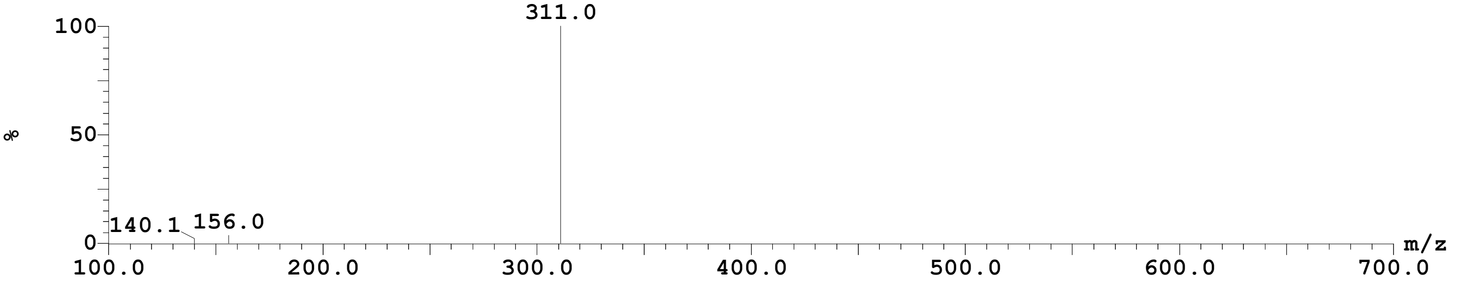

Peak ID	Time
1	0.28

1:(Time: 0.30) Combine (104:119-(20:28+200:208)) 2:MS ES- 4.9e+005

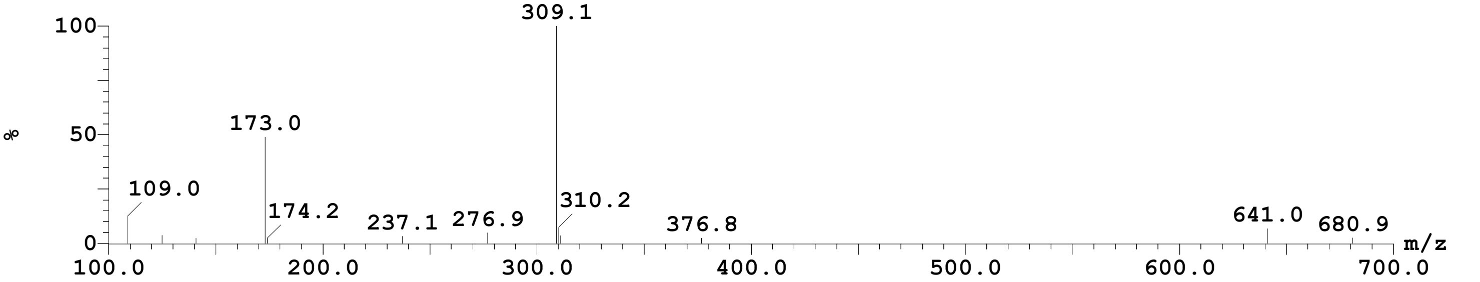

Peak ID	Time
3	0.88

3:(Time: 0.88) Combine (323:337-(243:250+407:413)) 2:MS ES- 3.1e+004

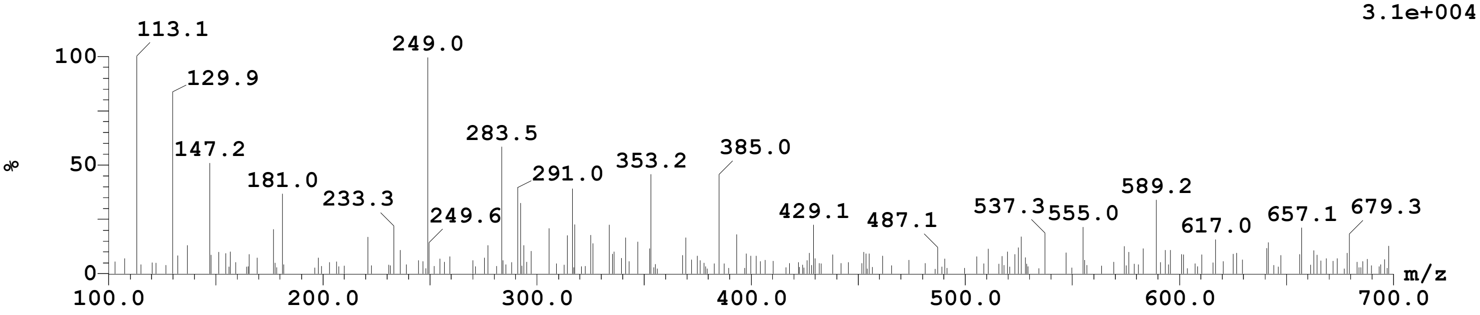

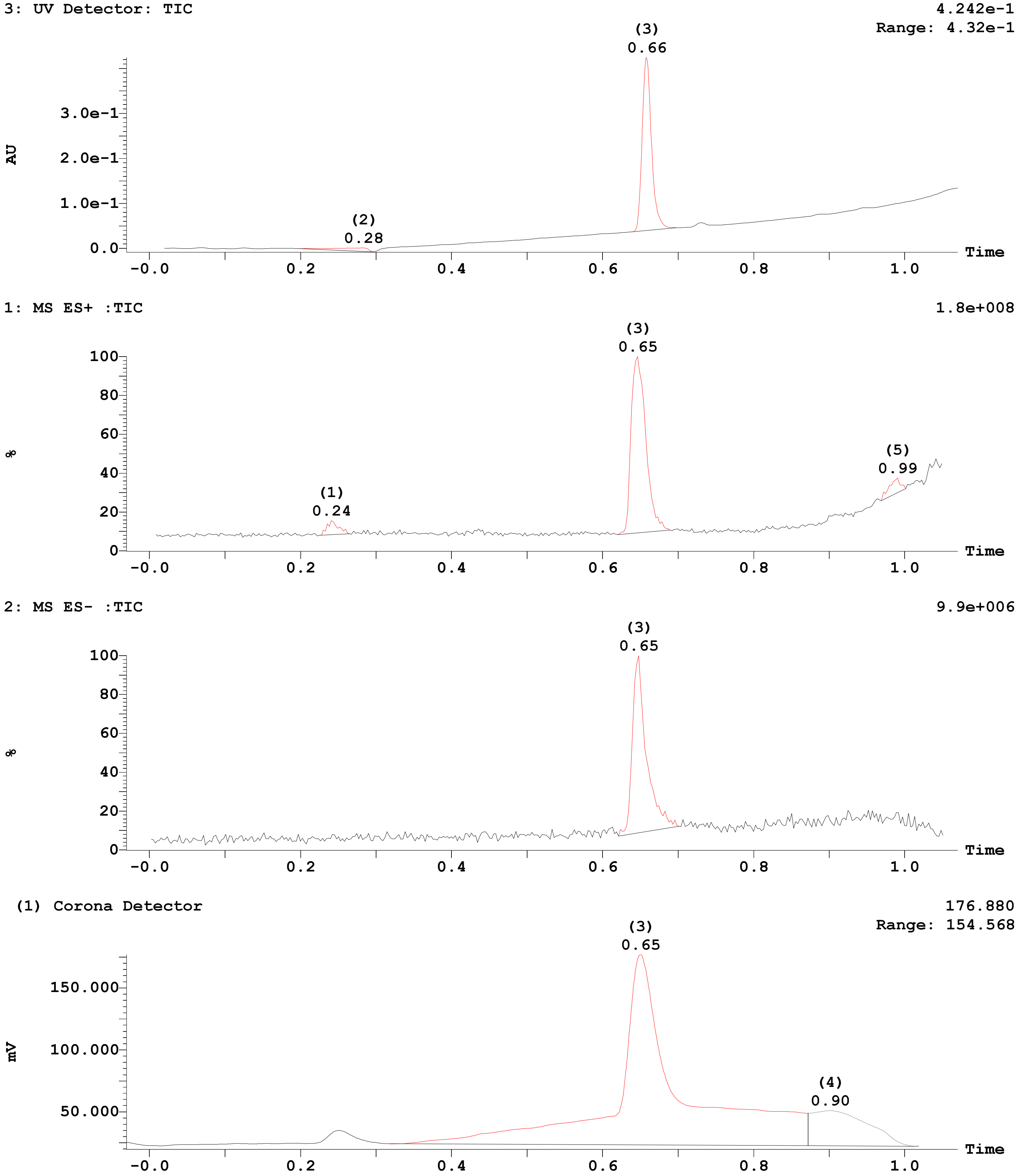
3: UV Detector: TIC
4.242e-1
Range: 4.32e-1
AU
3.0e-1
2.0e-1
1.0e-1
0.0
(2) 0.28
(3) 0.66
Time
-0.0
0.2
0.4
0.6
0.8
1.0
1: MS ES+ :TIC
1.8e+008
%
100
80
60
40
20
0
(1) 0.24
(3) 0.65
(5) 0.99
2: MS ES- :TIC
9.9e+006
(3) 0.65
(1) Corona Detector
176.880
Range: 154.568
mV
150.000
100.000
50.000
(3) 0.65
(4) 0.90

File:13zp546l3 Vial:5:7 ID:A6
Method:C:\MASSLYNX\1minLC_MS.olp

Peak ID **Time**
1 0.24

1:(Time: 0.24) Combine (84:98-(3:10+175:182)) 1:MS ES+ 2.3e+006

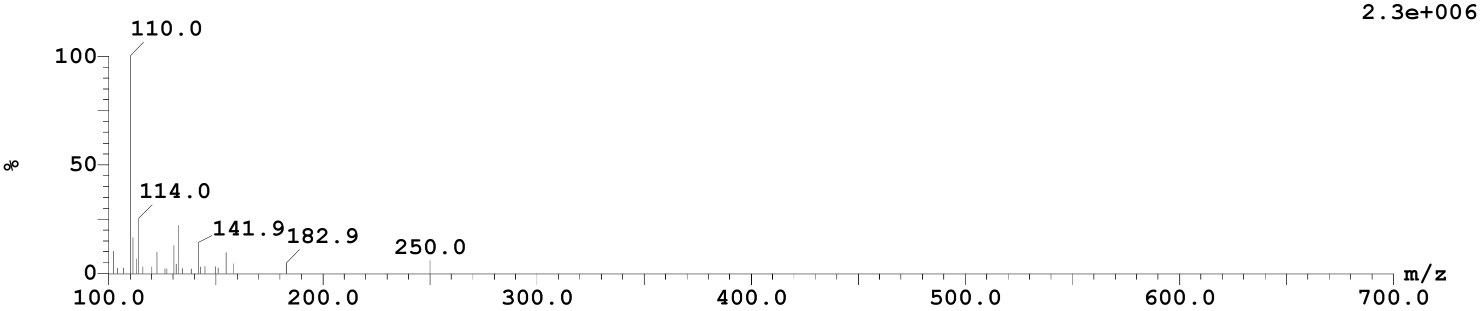

Peak ID **Time**
2 0.28

2:(Time: 0.28) Combine (99:114-187:194) 1:MS ES+ 6.6e+005

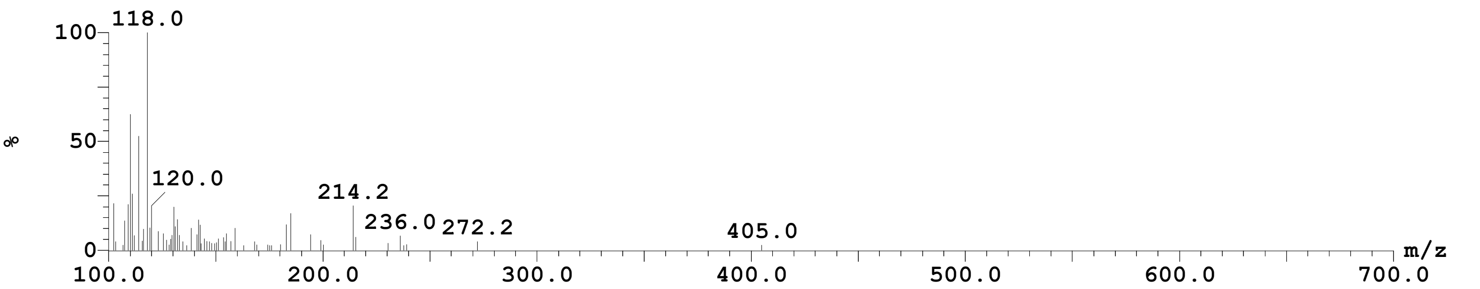

Peak ID **Time**
2 0.28

2:(Time: 0.28) Combine (99:114-186:194) 2:MS ES- 9.6e+003

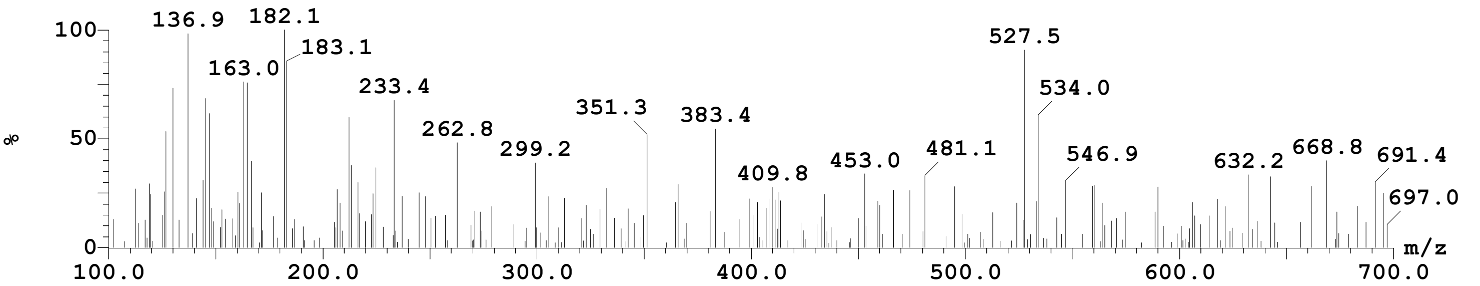

Peak ID **Time**
3 0.65

3:(Time: 0.66) Combine (240:255-(157:165+337:345)) 1:MS ES+ 6.9e+007

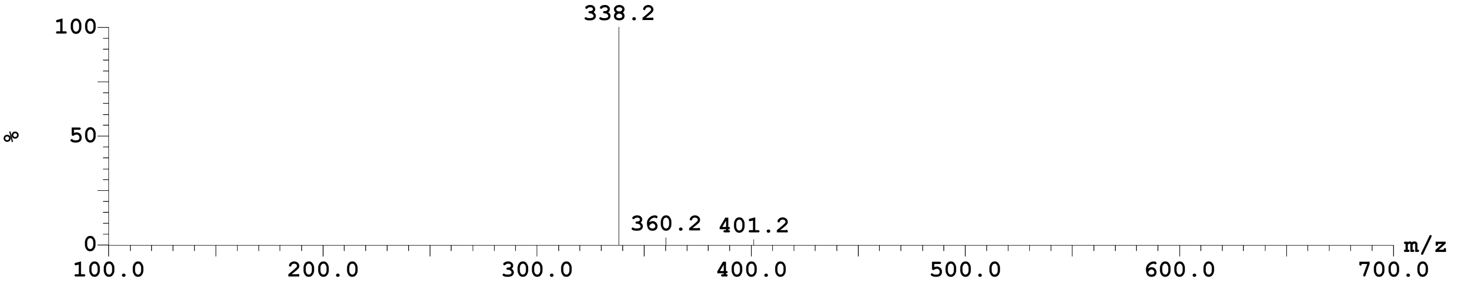

File:13zp546l3 Vial:5:7 ID:A6
Method:C:\MASSLYNX\1minLC_MS.olp

Peak ID **Time**
3 0.65

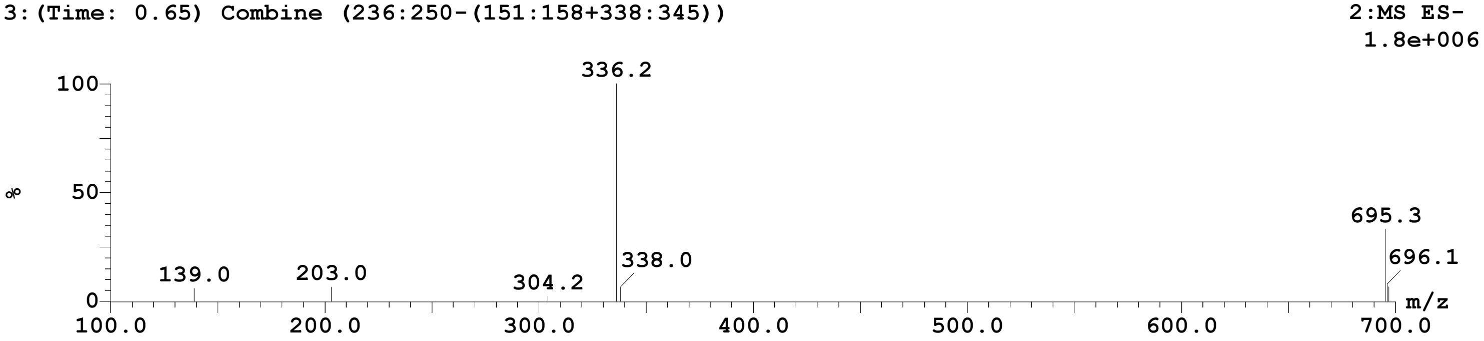

Peak ID **Time**
5 0.99

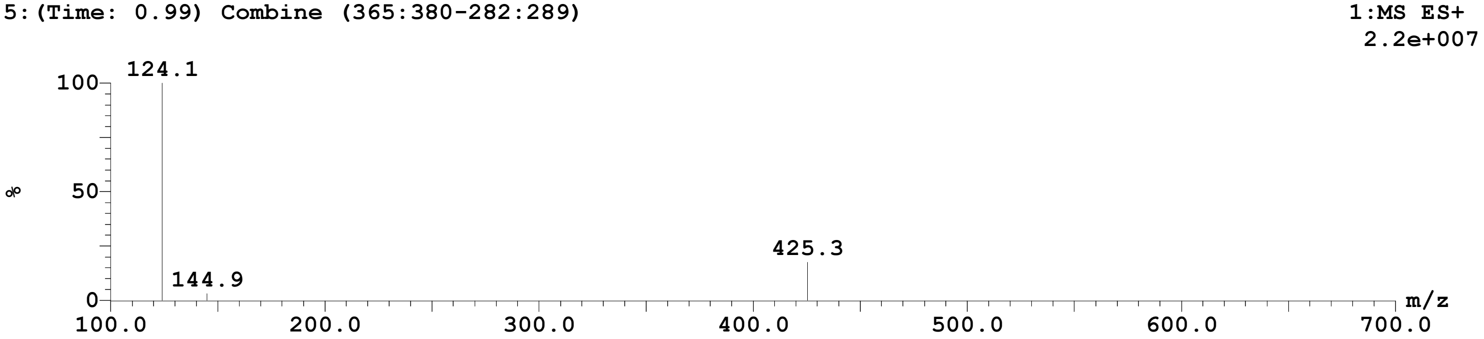

SYNGENTA SQD OPEN ACCESS LC-MS Page 1

File:13zp573l1 Vial:5:51 ID: **A7**

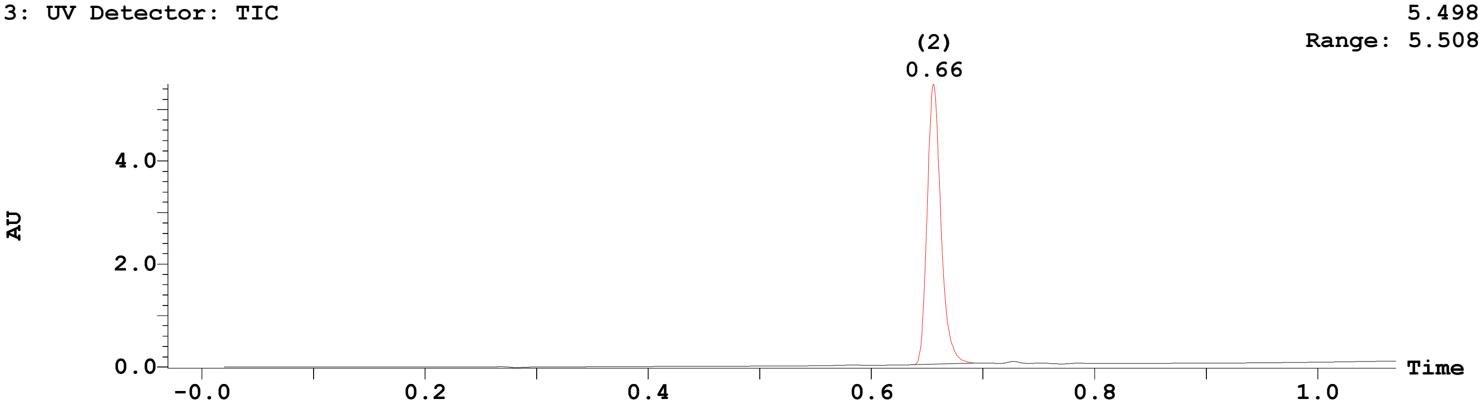

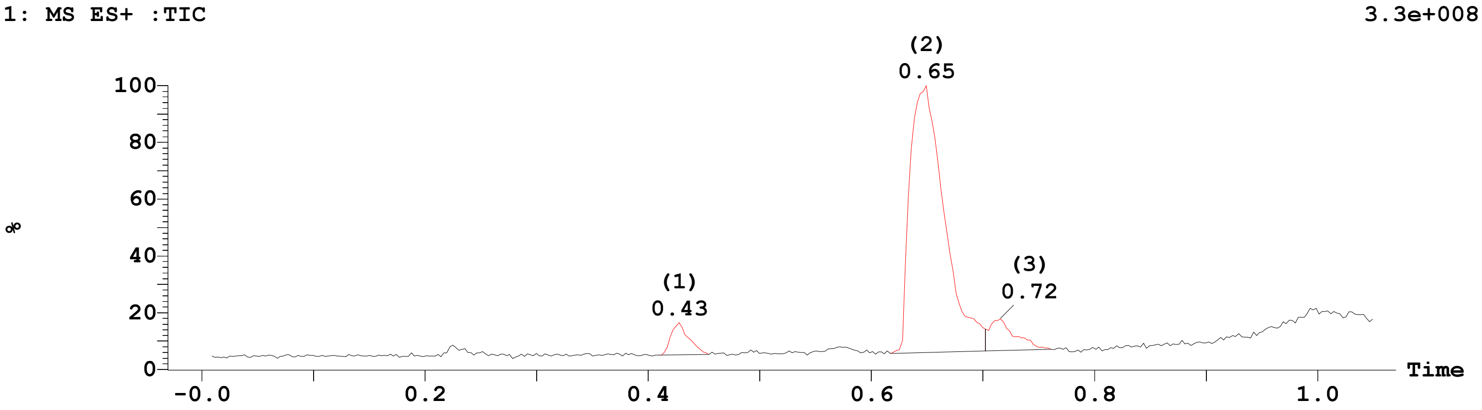

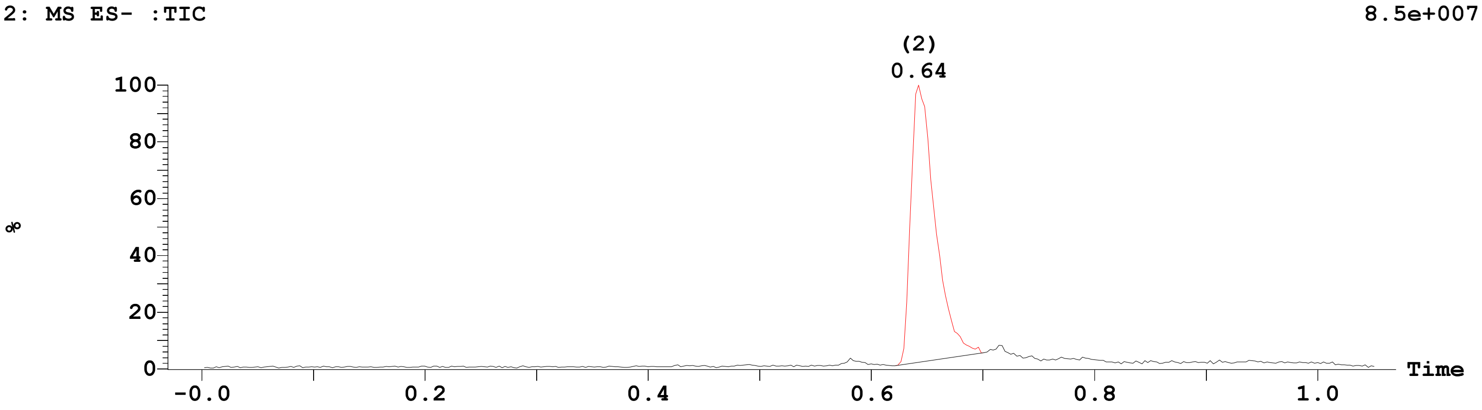

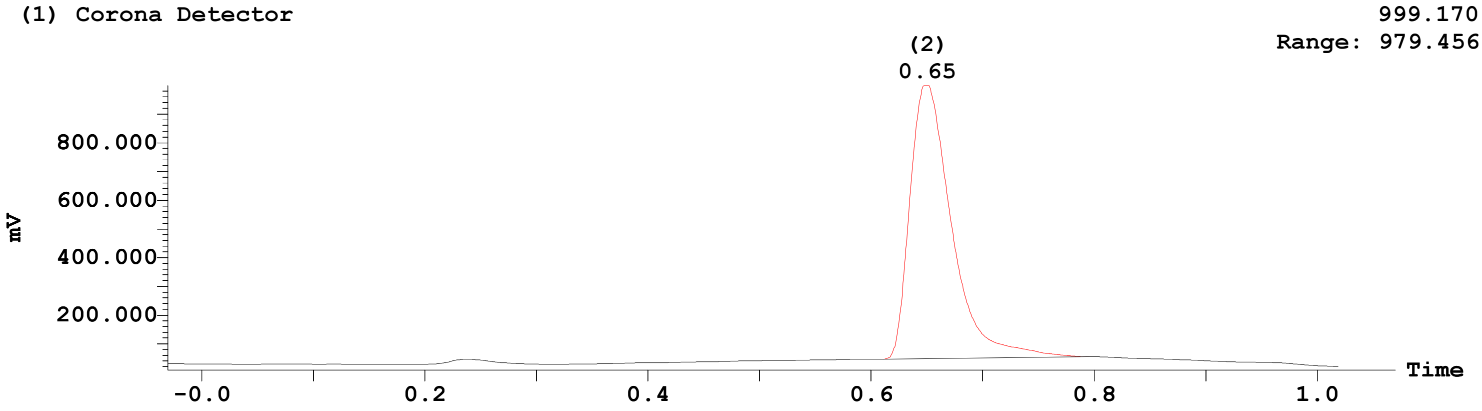

File:13zp573l1 Vial:5:51 ID:A7
Method:C:\MASSLYNX\1minLC_MS.olp

Peak ID **Time**
1 0.43

1:(Time: 0.43) Combine (154:169-(72:79+246:253)) 1:MS ES+ 7.5e+006

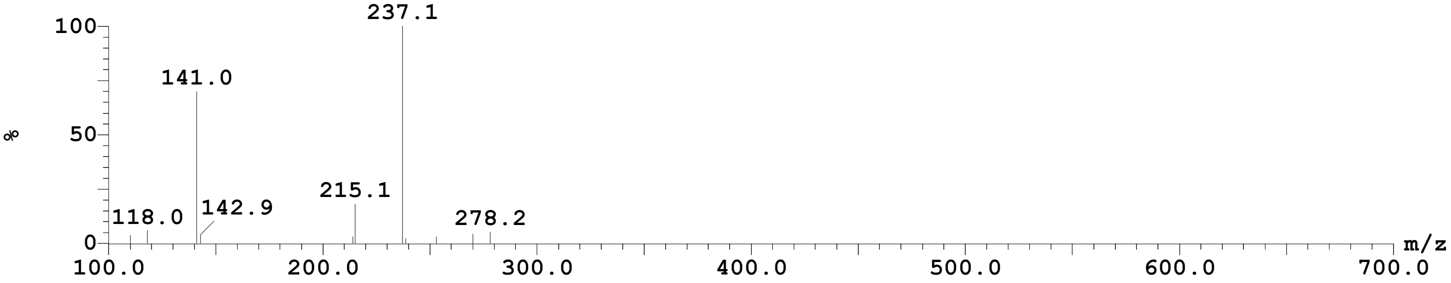

Peak ID **Time**
2 0.65

2:(Time: 0.66) Combine (239:254-(157:164+335:342)) 1:MS ES+ 1.7e+008

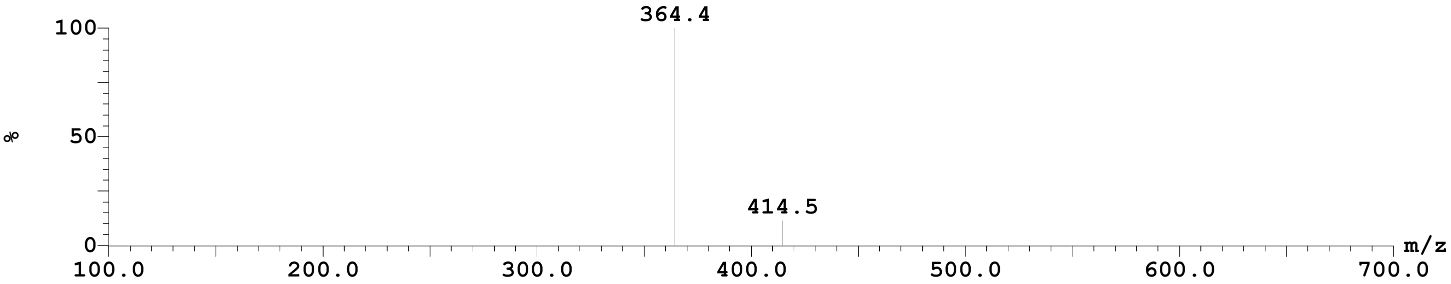

Peak ID **Time**
2 0.65

2:(Time: 0.64) Combine (233:248-(151:158+338:345)) 2:MS ES- 3.7e+007

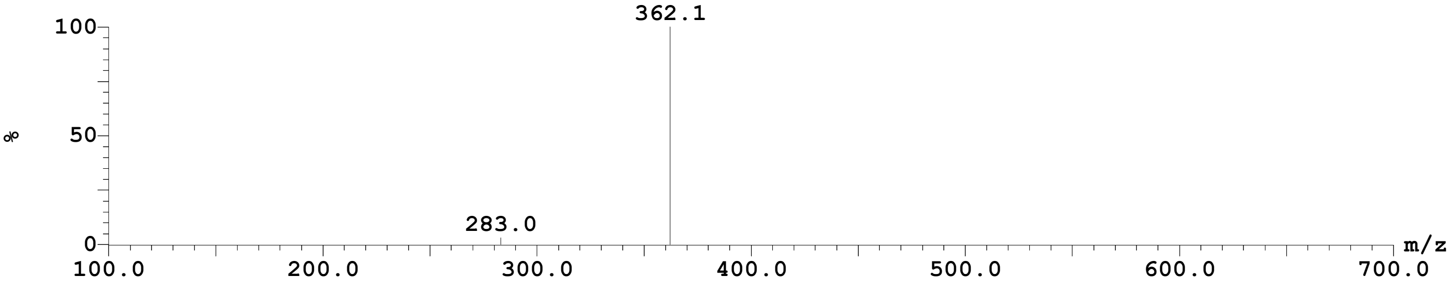

Peak ID **Time**
3 0.72

3:(Time: 0.72) Combine (262:276-(182:189+361:368)) 1:MS ES+ 9.3e+006

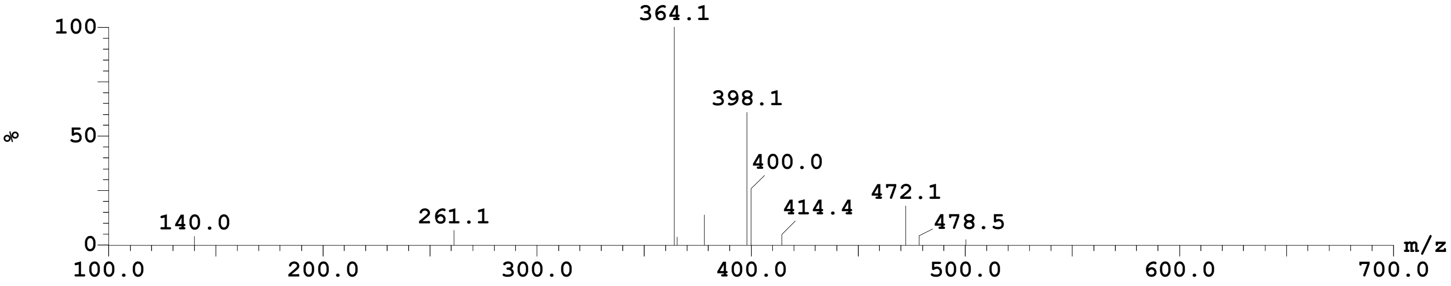

SYNGENTA SQD OPEN ACCESS LC-MS Page 1

File:13zp574l1 Vial:5:52 ID: **A8**

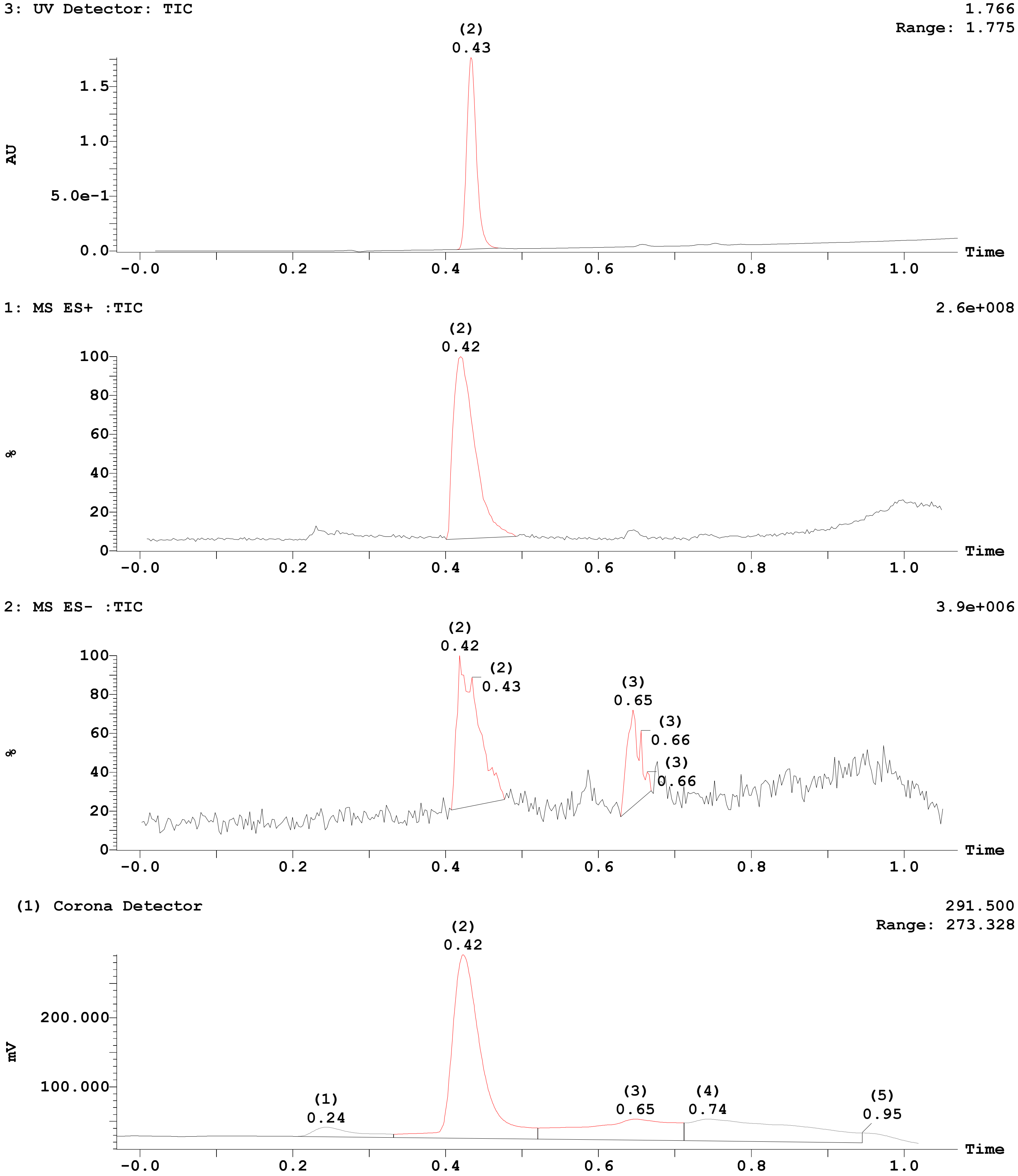

File:13zp574l1 Vial:5:52 ID:A8
Method:C:\MASSLYNX\1minLC_MS.olp

Peak ID 2 **Time** 0.42

2:(Time: 0.42) Combine (150:166-(69:76+260:267)) 1:MS ES+ 1.2e+008

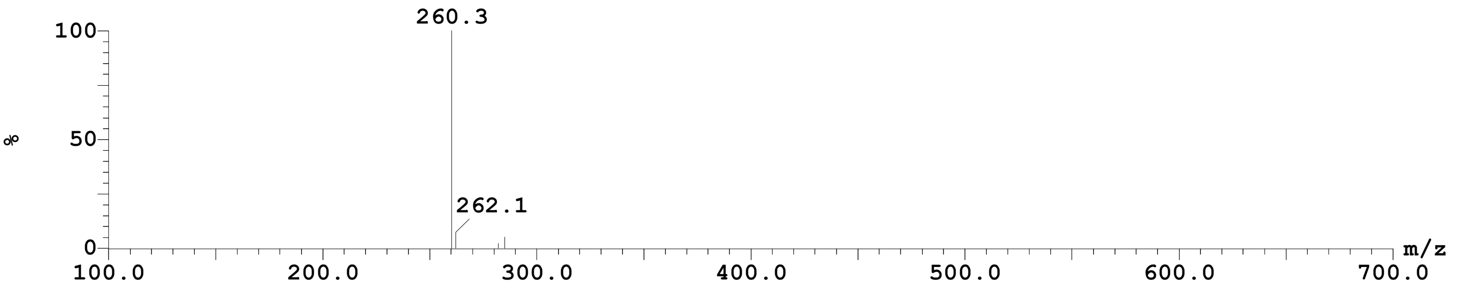

Peak ID 2 **Time** 0.42

2:(Time: 0.43) Combine (155:170-(73:80+251:259)) 2:MS ES- 1.2e+005

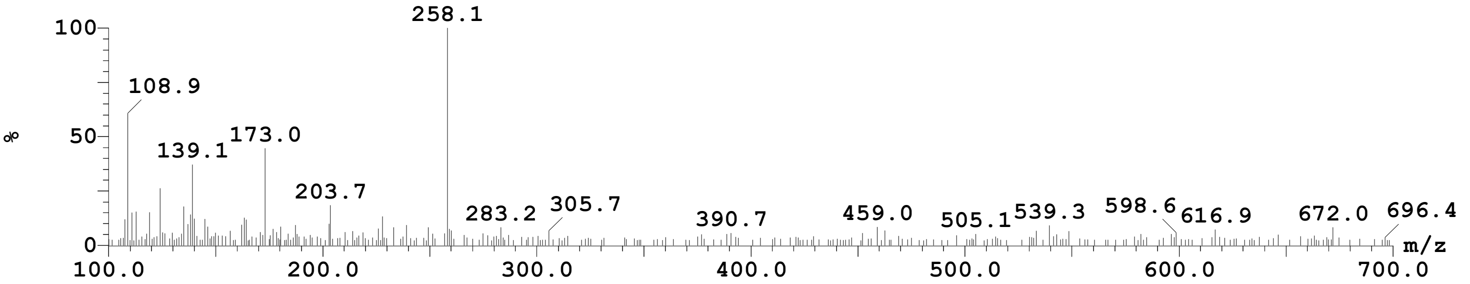

Peak ID 3 **Time** 0.65

3:(Time: 0.65) Combine (235:250-(154:161+326:333)) 2:MS ES- 8.3e+005

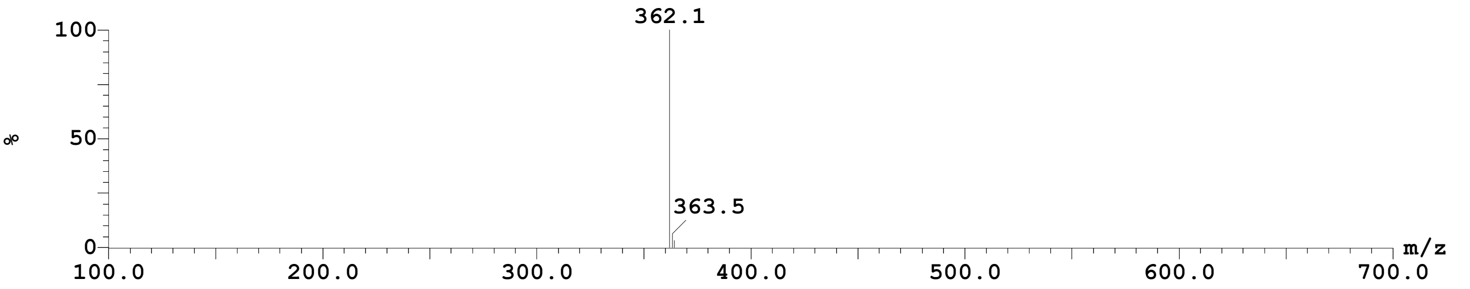

File:13zp575l1 Vial:5:53 ID: **A9**

3: UV Detector: TIC 4.522

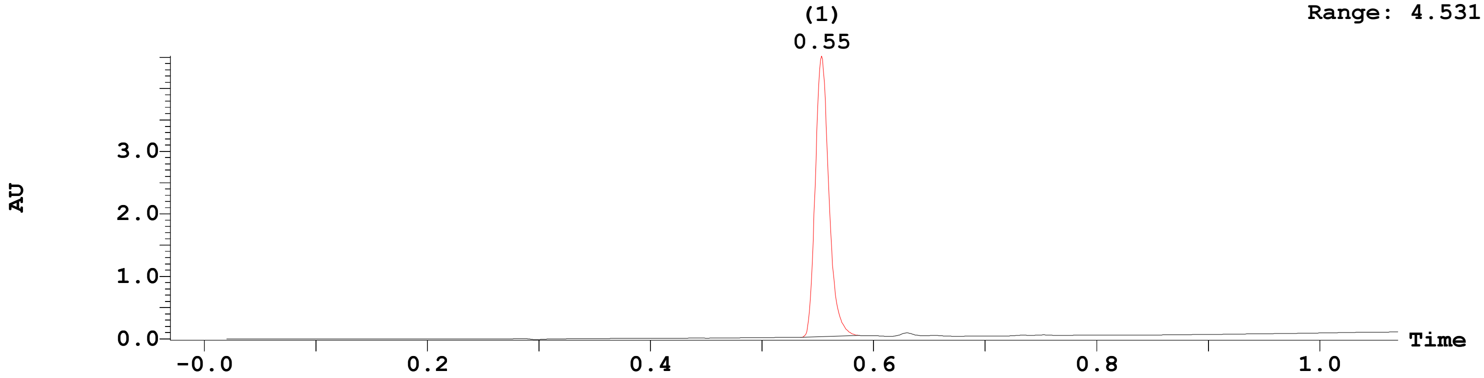

1: MS ES+ :TIC 3.4e+008

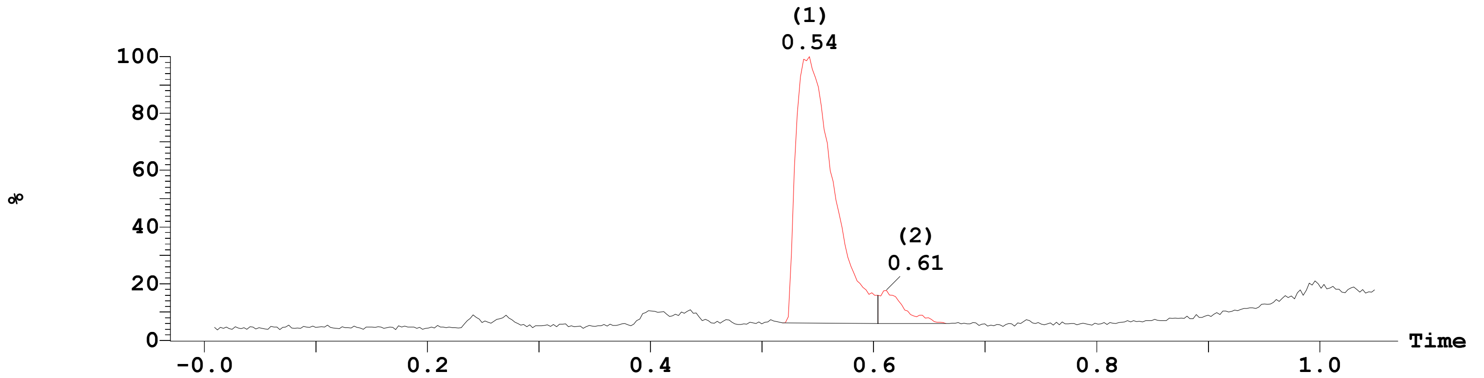

2: MS ES- :TIC 2.8e+007

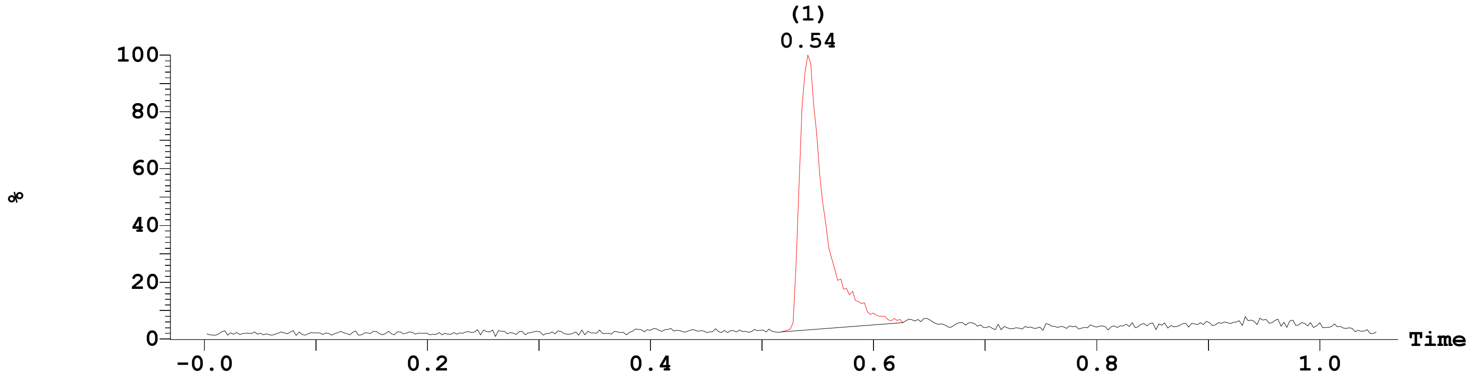

(1) Corona Detector 999.170

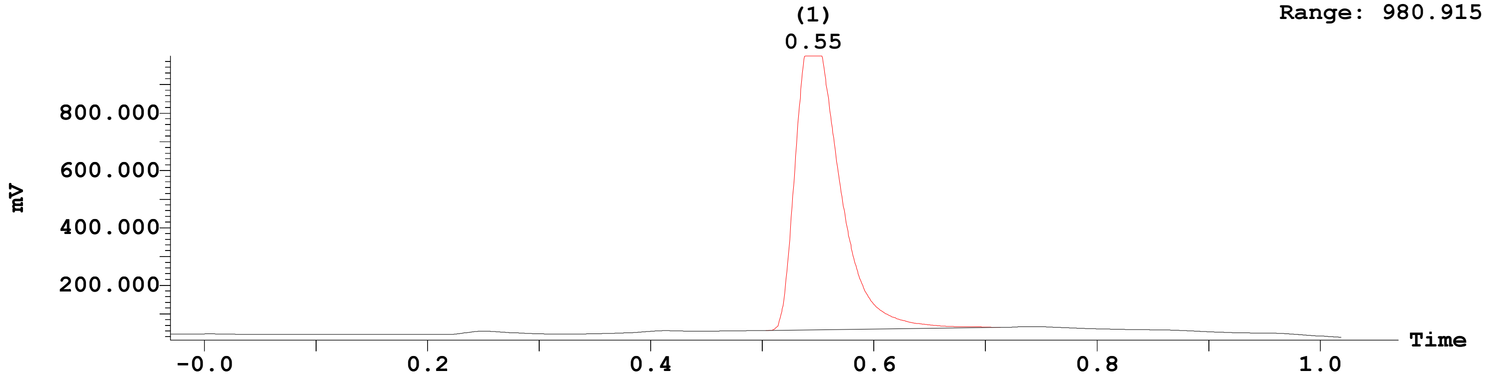

File:13zp575l1 Vial:5:53 ID:A9
Method:C:\MASSLYNX\1minLC_MS.olp

Peak ID	Time
1	0.54

1:(Time: 0.55) Combine (201:216-(118:126+296:303)) 1:MS ES+ 1.9e+008

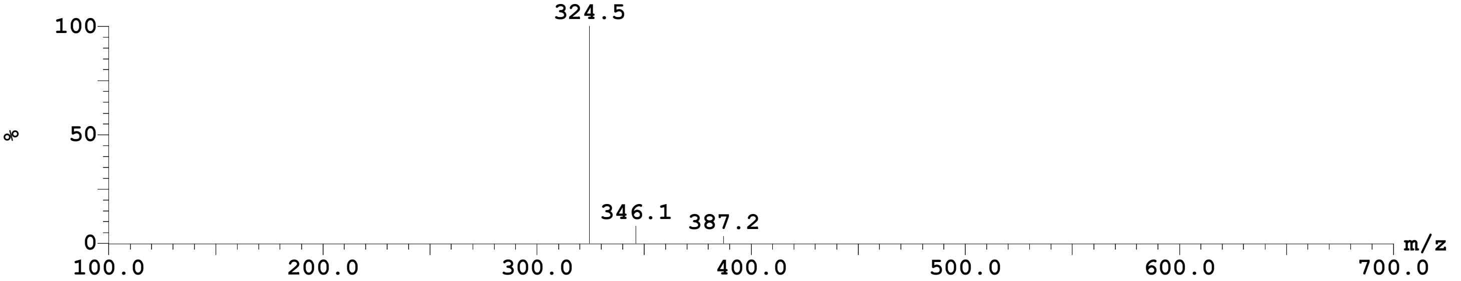

Peak ID	Time
1	0.54

1:(Time: 0.54) Combine (196:210-(112:119+310:317)) 2:MS ES- 5.4e+006

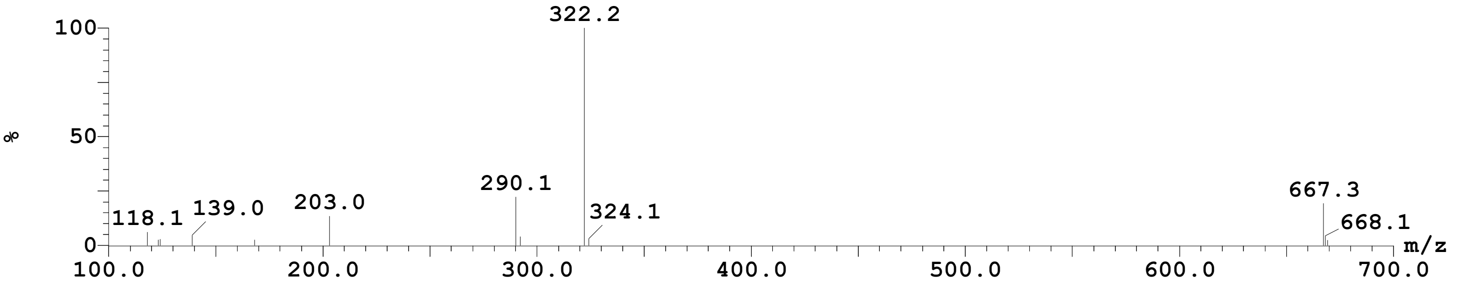

Peak ID	Time
2	0.61

2:(Time: 0.61) Combine (222:237-(145:152+325:332)) 1:MS ES+ 1.8e+007

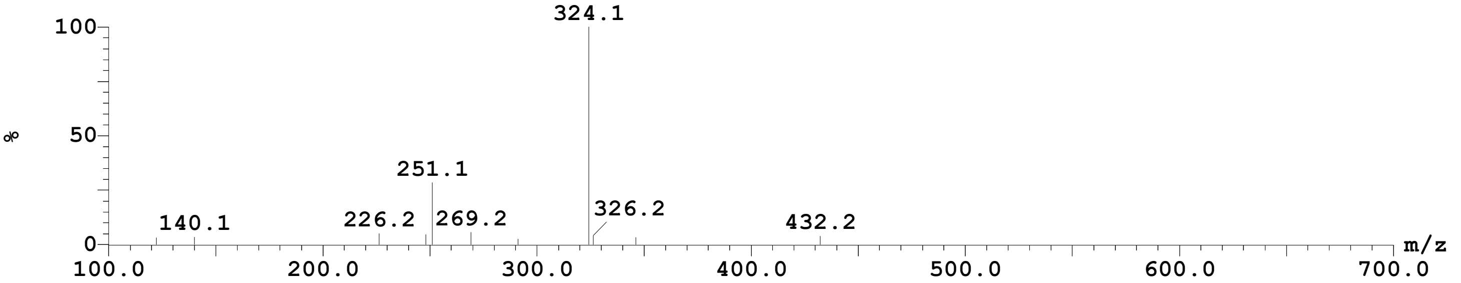

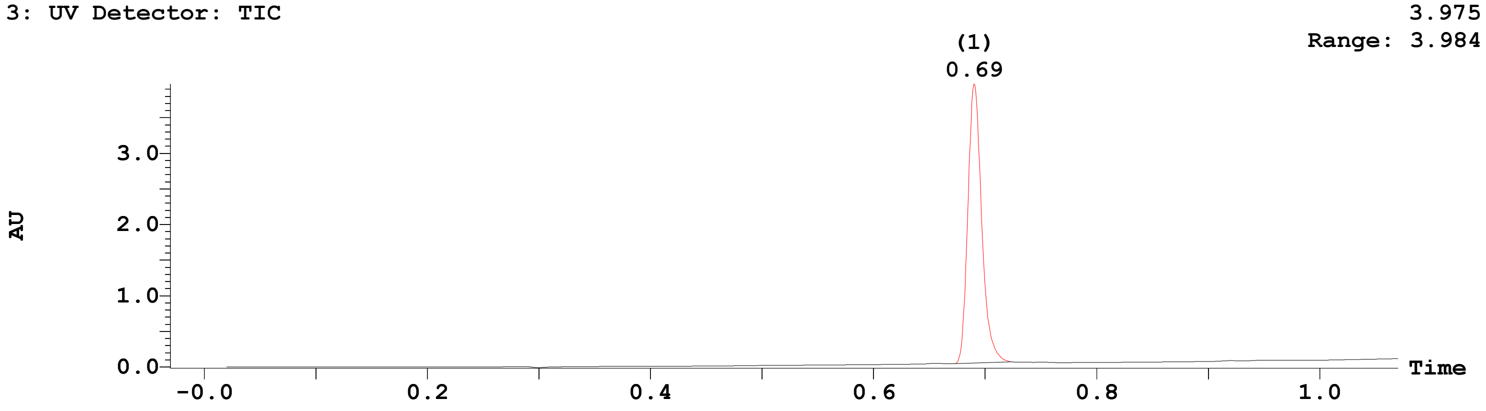
3: UV Detector: TIC
3.975
Range: 3.984
(1)
0.69
AU
3.0
2.0
1.0
0.0
Time
-0.0
0.2
0.4
0.6
0.8
1.0

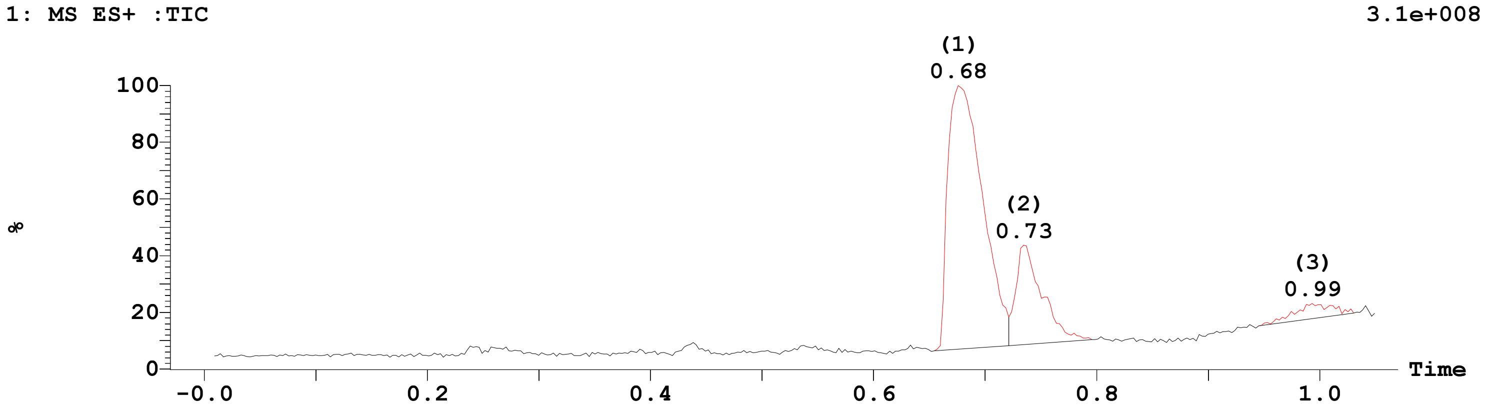
1: MS ES+ :TIC
3.1e+008
(1)
0.68
(2)
0.73
(3)
0.99
%
100
80
60
40
20
0
Time
-0.0
0.2
0.4
0.6
0.8
1.0

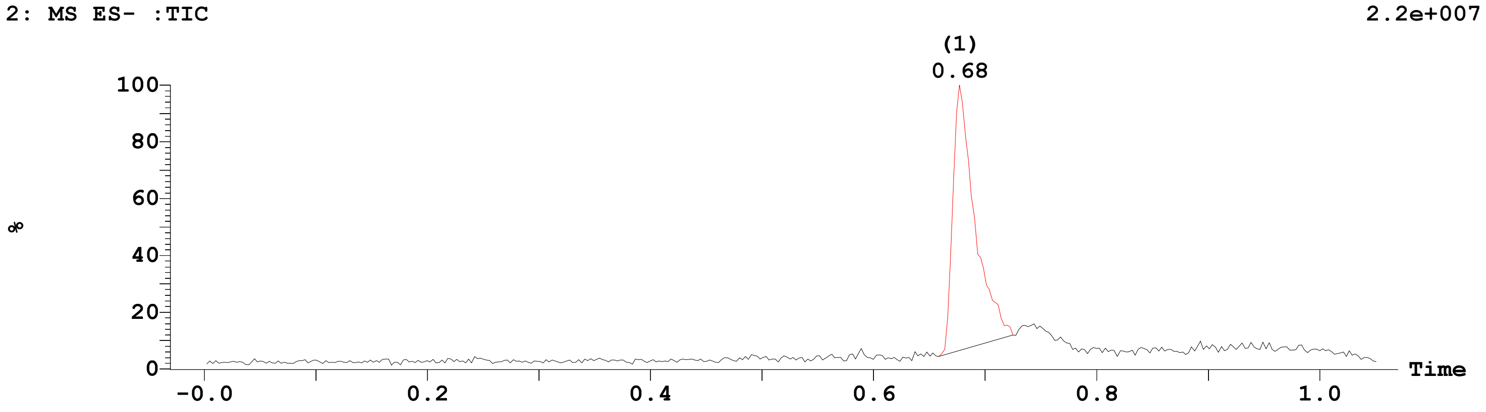
2: MS ES- :TIC
2.2e+007
(1)
0.68
%
100
80
60
40
20
0
Time
-0.0
0.2
0.4
0.6
0.8
1.0

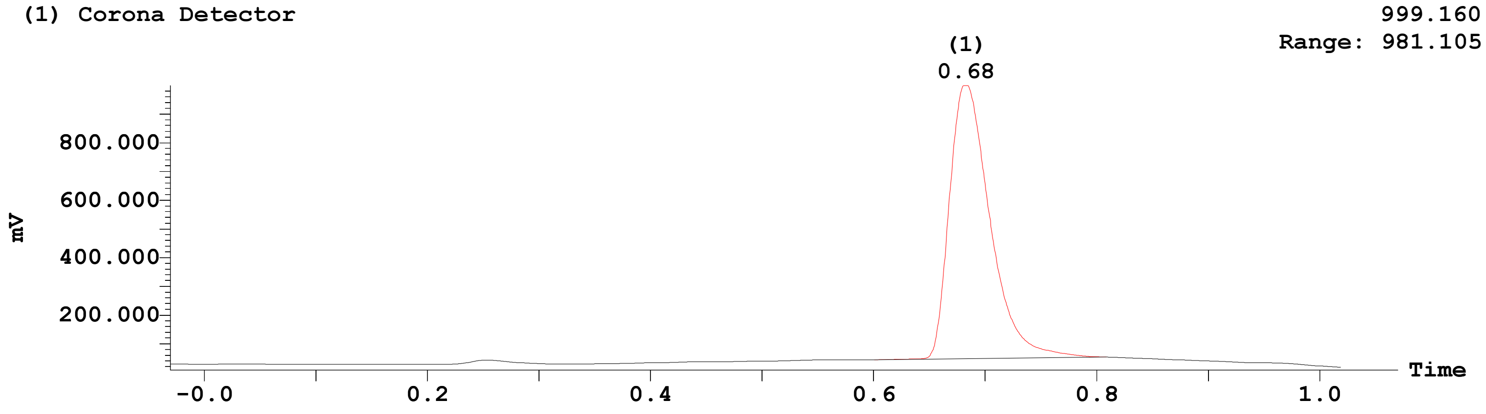
(1) Corona Detector
999.160
Range: 981.105
(1)
0.68
mV
800.000
600.000
400.000
200.000
Time
-0.0
0.2
0.4
0.6
0.8
1.0

File:13zo274l2 Vial:5:50 ID:A10
Method:C:\MASSLYNX\1minLC_MS.olp

Peak ID **Time**
1 0.68

1:(Time: 0.69) Combine (252:267-(170:177+347:355)) 1:MS ES+
1.8e+008

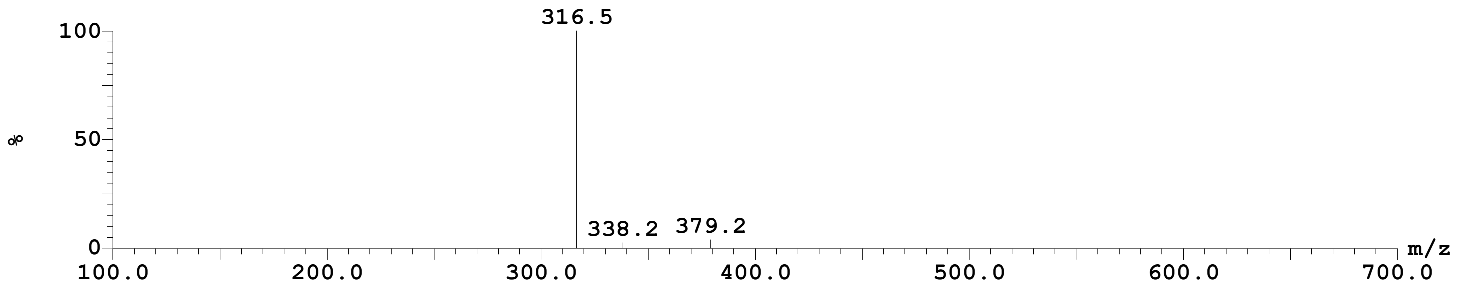

Peak ID **Time**
1 0.68

1:(Time: 0.68) Combine (247:262-(165:172+347:354)) 2:MS ES-
2.8e+006

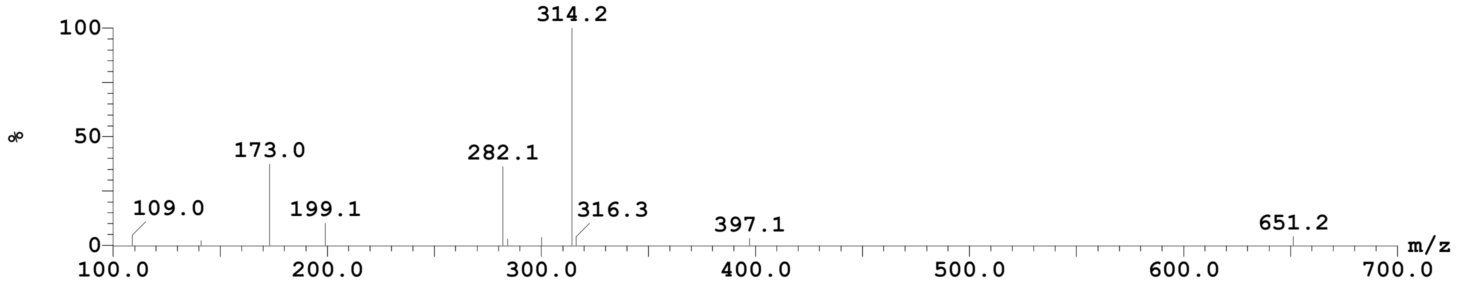

Peak ID **Time**
2 0.73

2:(Time: 0.73) Combine (268:283-(189:196+374:381)) 1:MS ES+
3.1e+007

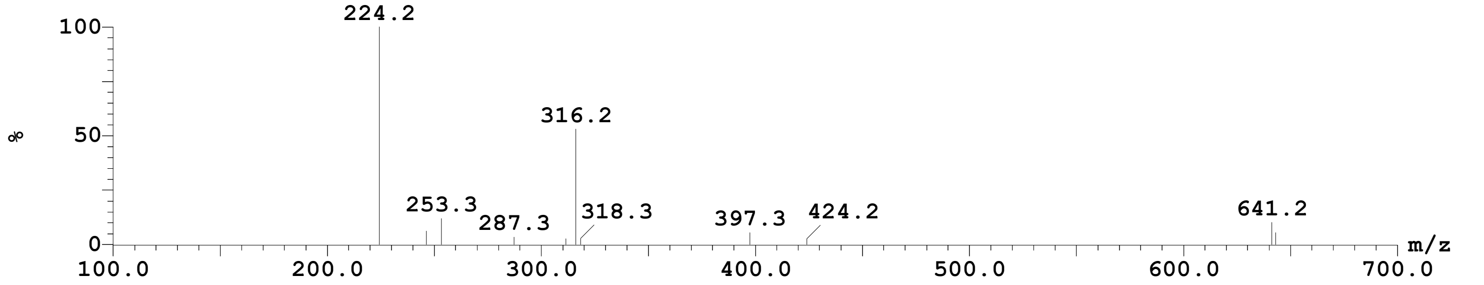

Peak ID **Time**
3 0.99

3:(Time: 0.99) Combine (365:380-273:280) 1:MS ES+
2.5e+007

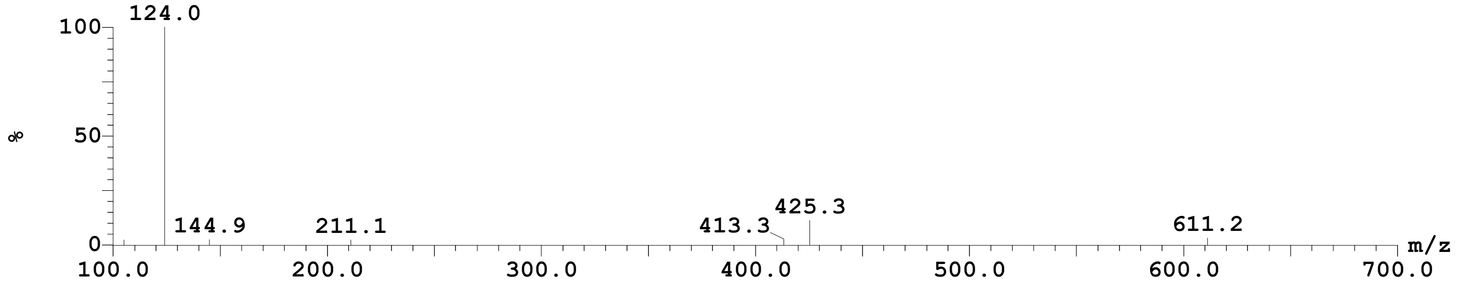

File:13zn454l2 Vial:5:50 ID: **B2**

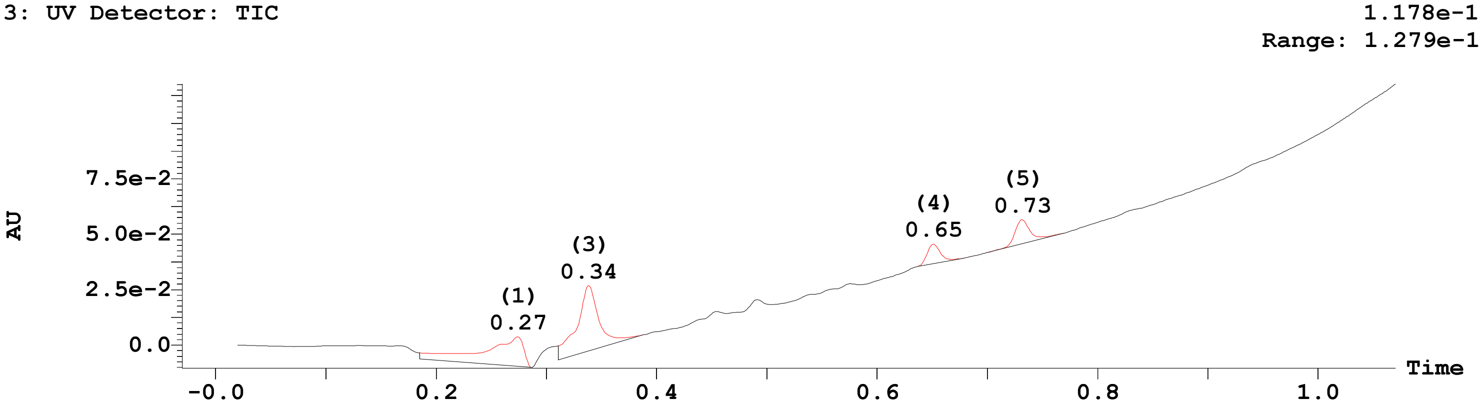

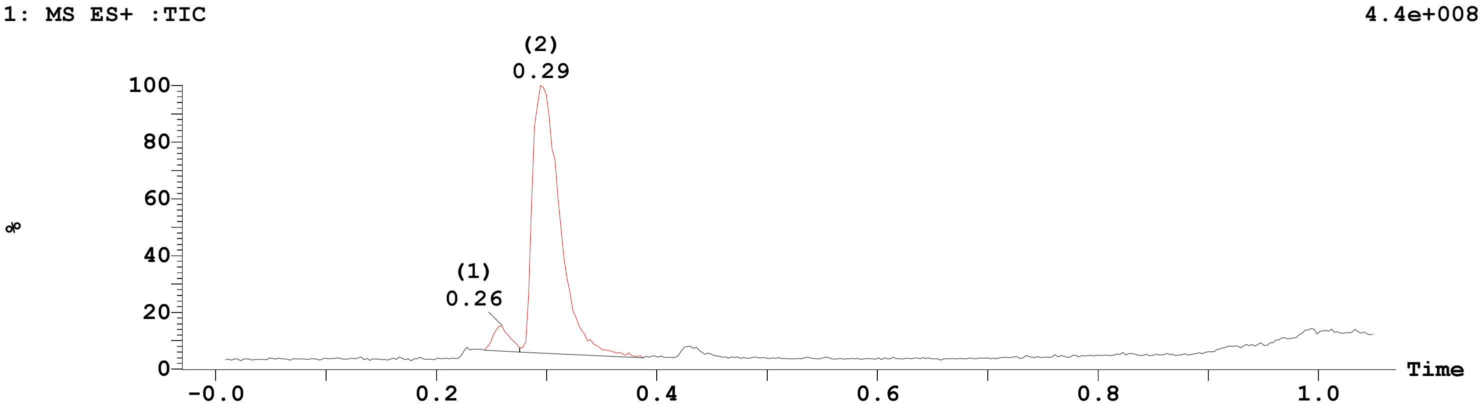

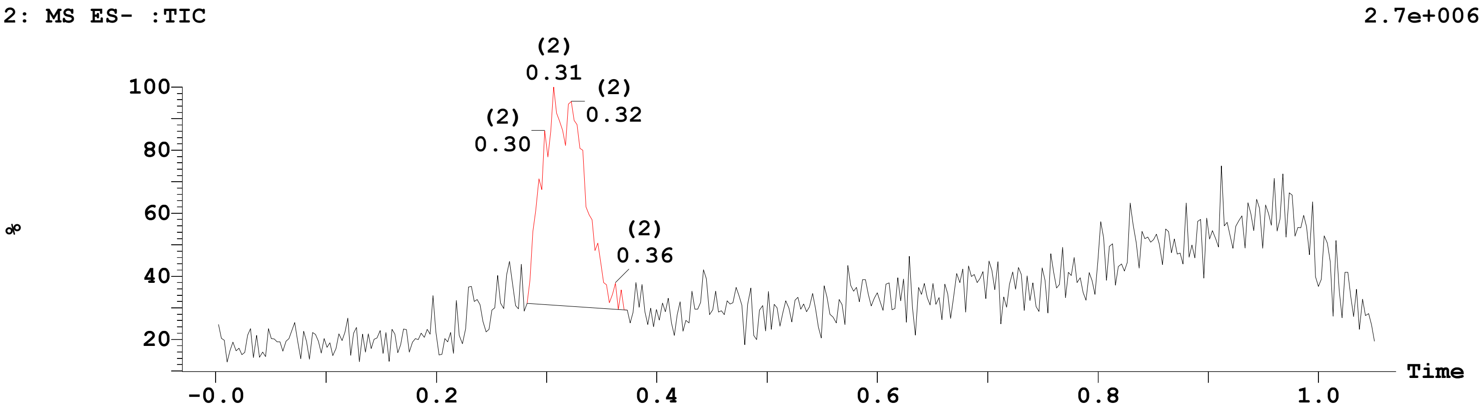

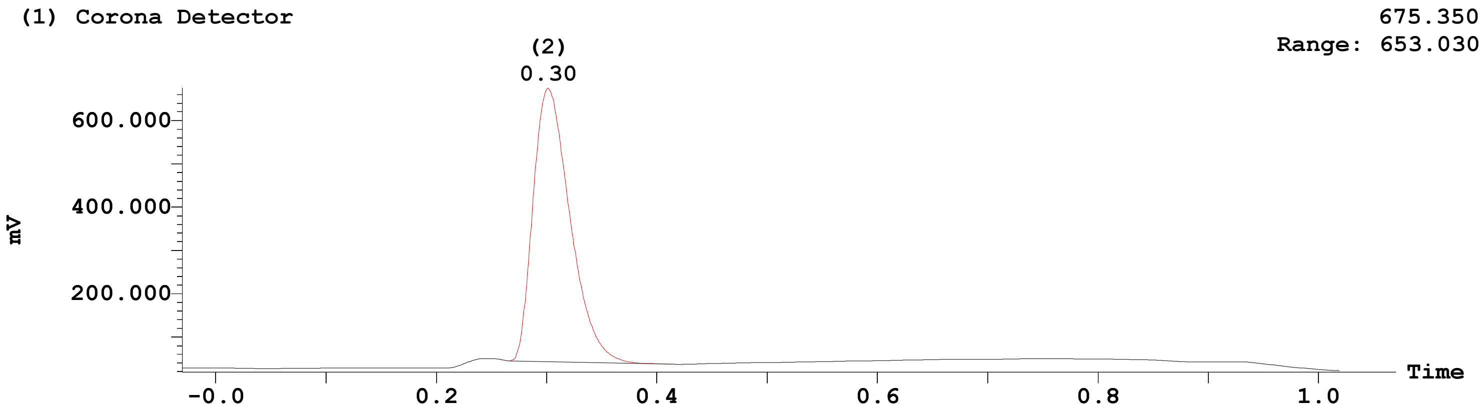

File:13zn454l2 Vial:5:50 ID:B2
Method:C:\MASSLYNX\1minLC_MS.olp

Peak ID **Time**
1 0.26

1:(Time: 0.27) Combine (96:111-183:190) 1:MS ES+
4.0e+007

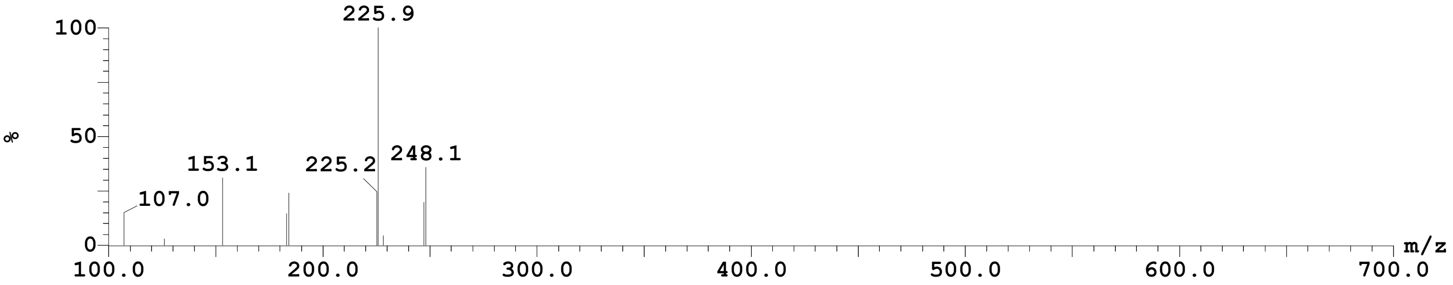

Peak ID **Time**
1 0.26

1:(Time: 0.27) Combine (95:110-182:190) 2:MS ES-
2.0e+004

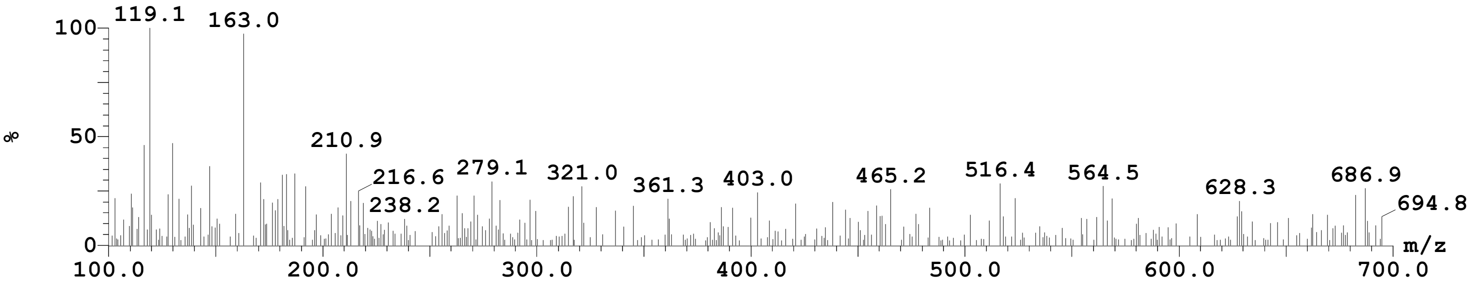

Peak ID **Time**
2 0.29

2:(Time: 0.29) Combine (104:119-(22:29+221:228)) 1:MS ES+
9.1e+007

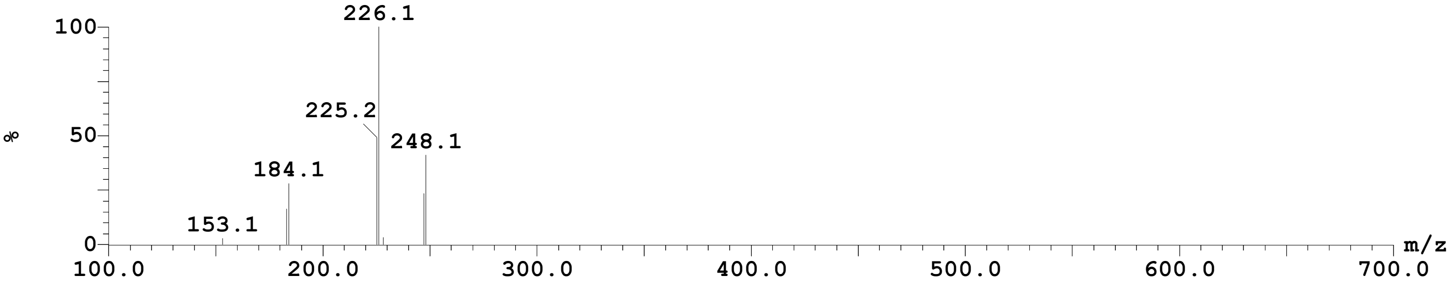

Peak ID **Time**
2 0.29

2:(Time: 0.31) Combine (108:123-(24:31+215:222)) 2:MS ES-
1.1e+005

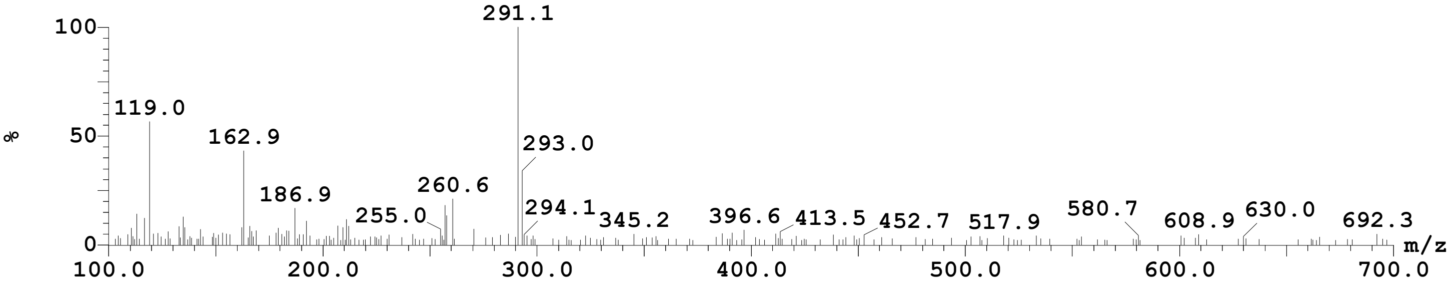

File:13zn454l2 Vial:5:50 ID:B2
Method:C:\MASSLYNX\1minLC_MS.olp

Peak ID **Time**
3 0.34

3:(Time: 0.34) Combine (120:135-(35:42+221:229)) 1:MS ES+ 1.4e+007

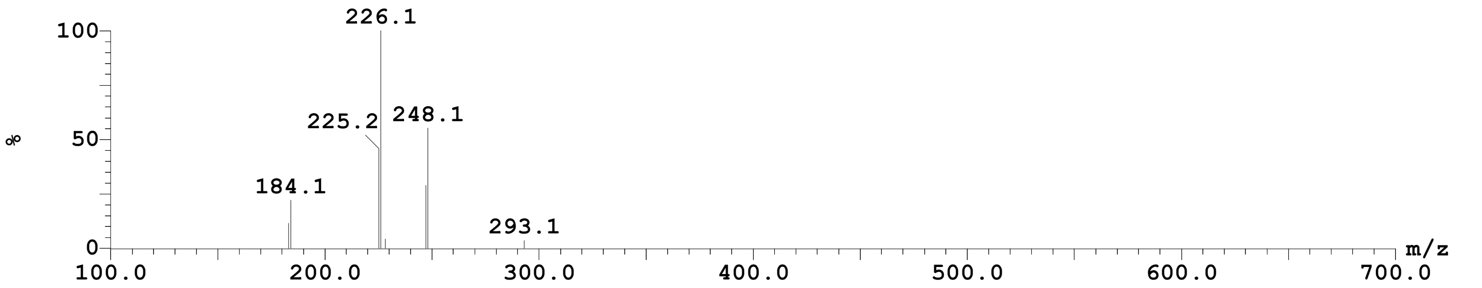

Peak ID **Time**
3 0.34

3:(Time: 0.34) Combine (119:134-(34:42+221:228)) 2:MS ES- 1.5e+005

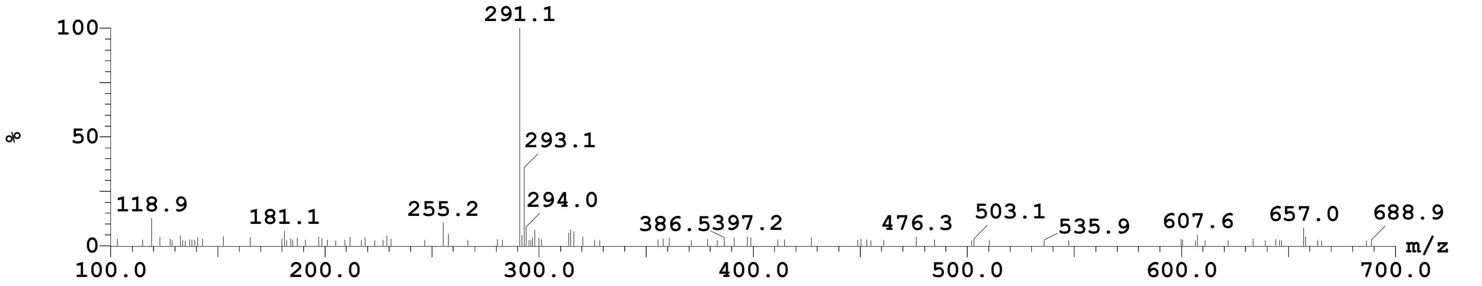

Peak ID **Time**
4 0.65

4:(Time: 0.65) Combine (237:252-(157:164+330:338)) 1:MS ES+ 4.7e+005

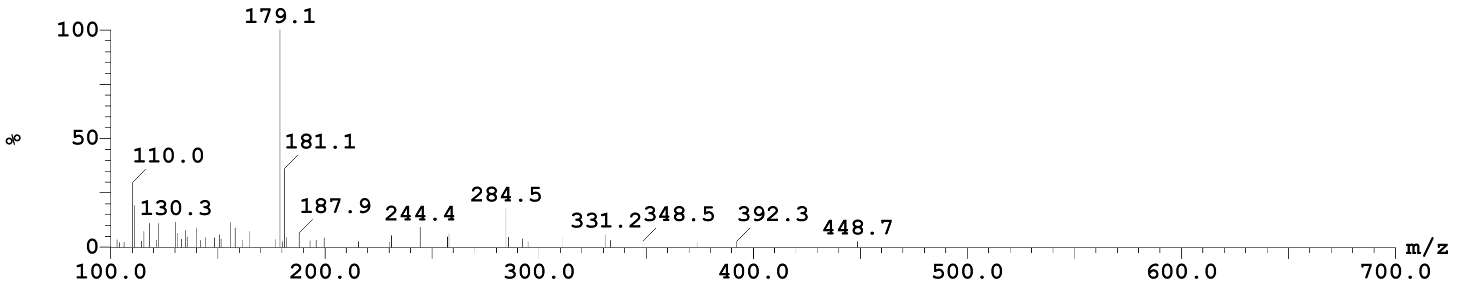

Peak ID **Time**
4 0.65

4:(Time: 0.65) Combine (237:252-(156:164+330:337)) 2:MS ES- 1.0e+004

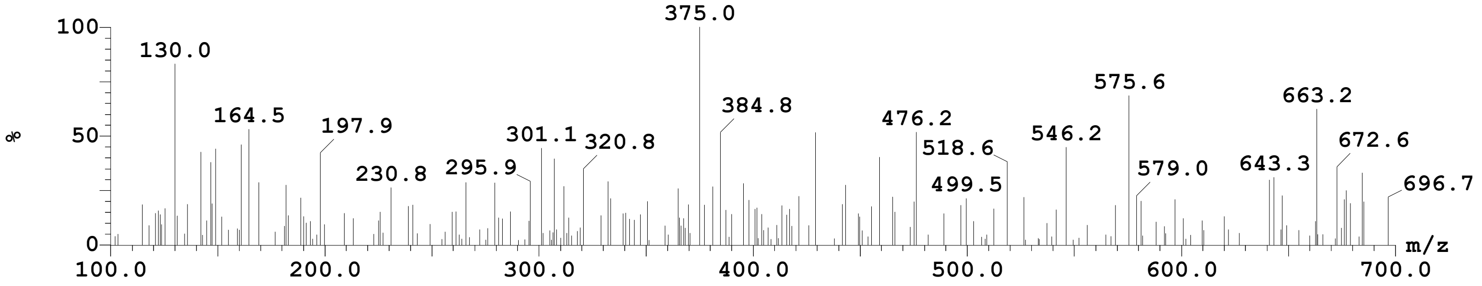

File:13zn454l2 Vial:5:50 ID:B2
Method:C:\MASSLYNX\1minLC_MS.olp

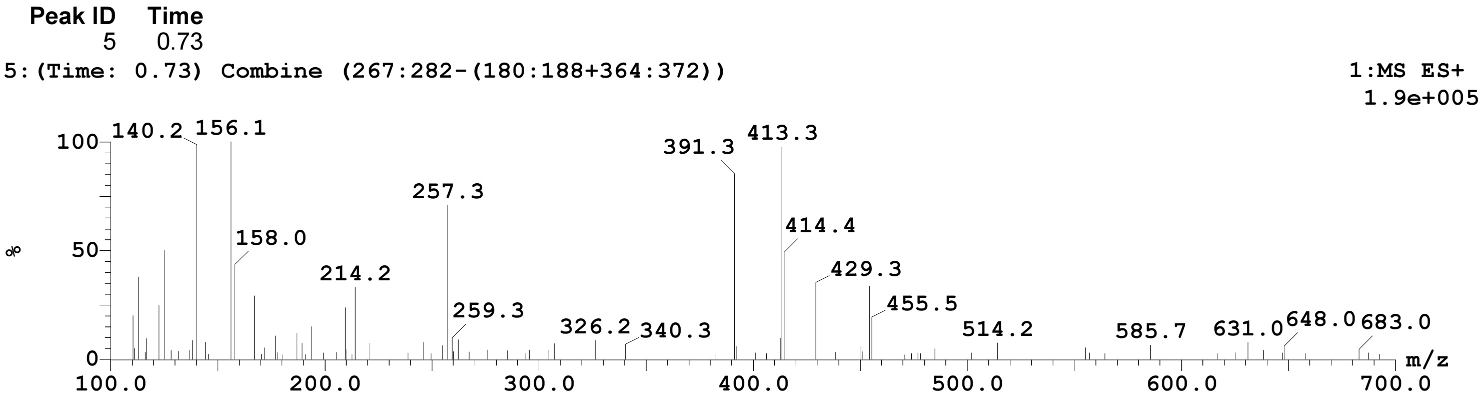

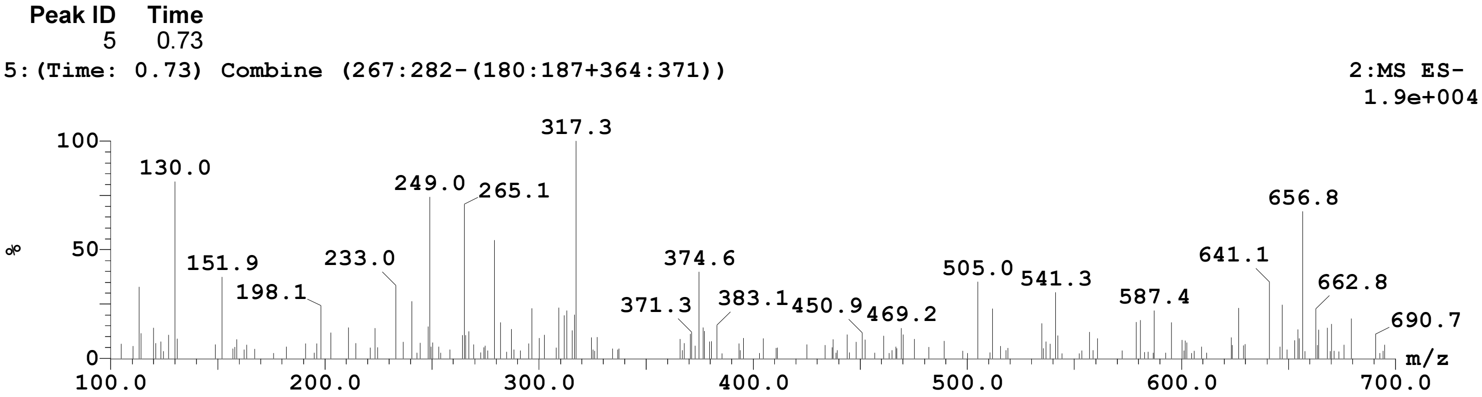

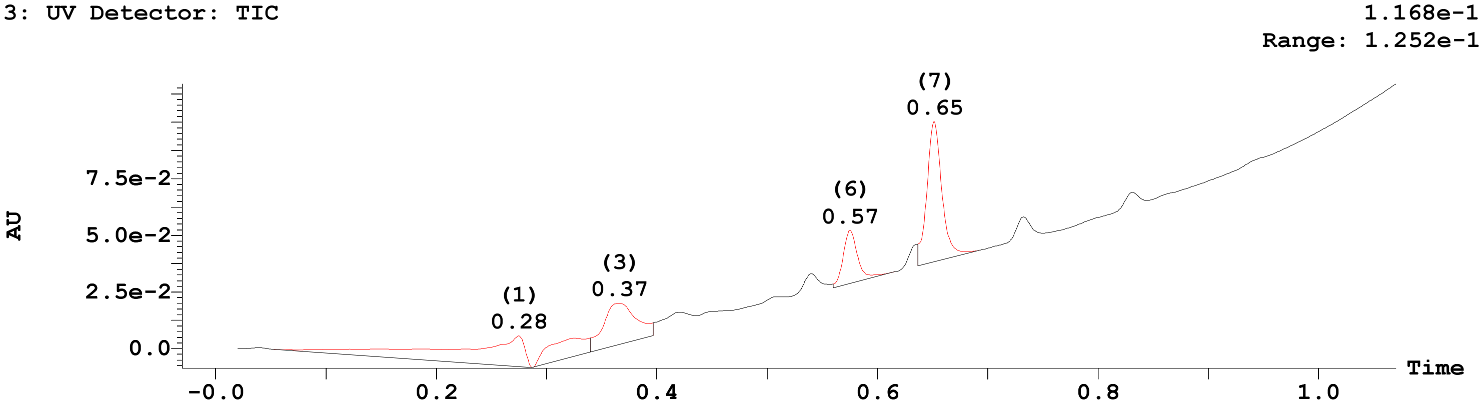
3: UV Detector: TIC
1.168e-1
Range: 1.252e-1
AU
7.5e-2
5.0e-2
2.5e-2
0.0
(1) 0.28
(3) 0.37
(6) 0.57
(7) 0.65
-0.0 0.2 0.4 0.6 0.8 1.0
Time

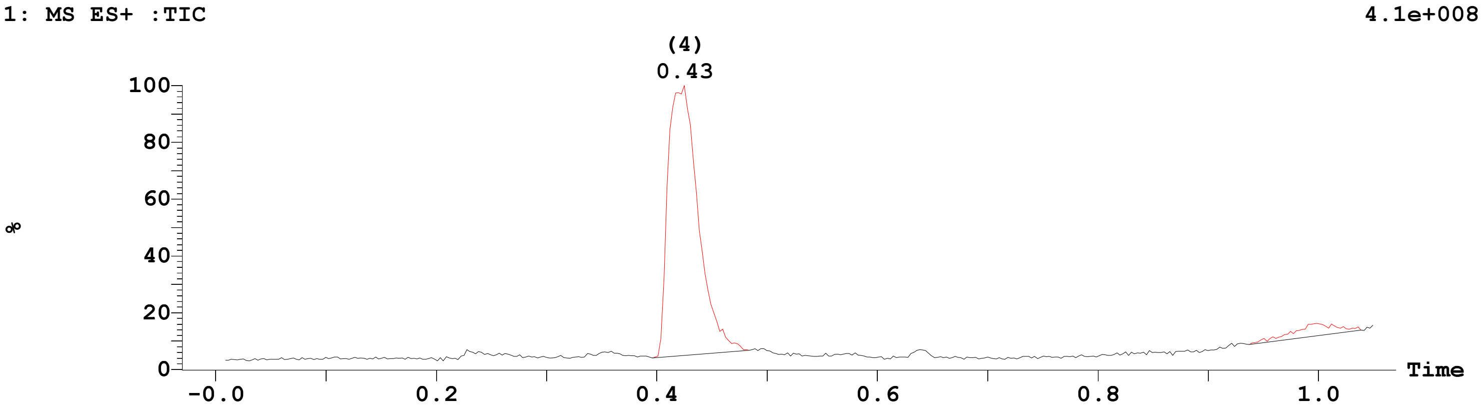
1: MS ES+ :TIC
4.1e+008
%
100
80
60
40
20
0
(4) 0.43
-0.0 0.2 0.4 0.6 0.8 1.0
Time

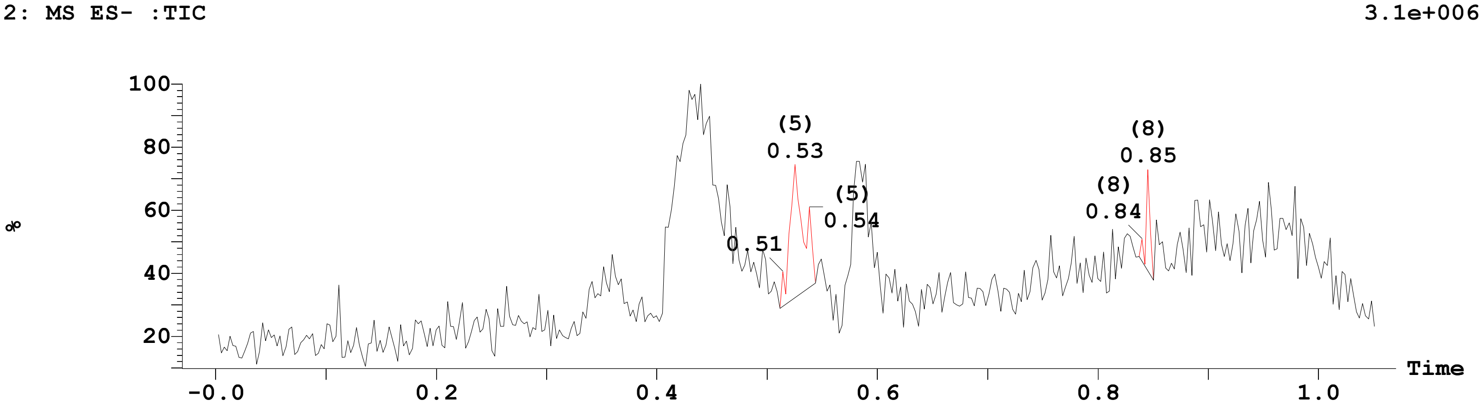
2: MS ES- :TIC
3.1e+006
%
100
80
60
40
20
(5) 0.53
(5) 0.54
0.51
(8) 0.85
(8) 0.84
-0.0 0.2 0.4 0.6 0.8 1.0
Time

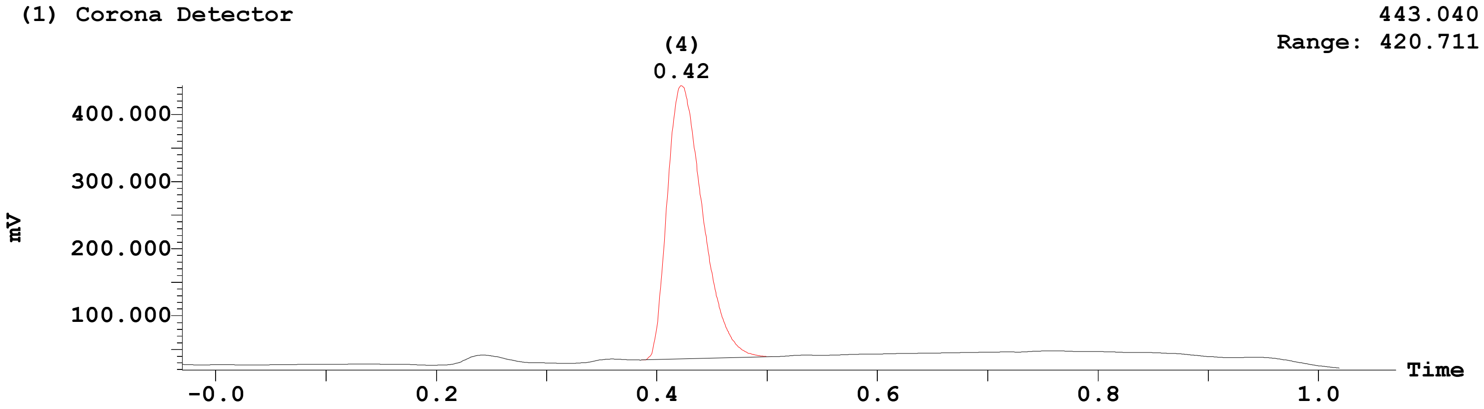
(1) Corona Detector
443.040
Range: 420.711
mV
400.000
300.000
200.000
100.000
(4) 0.42
-0.0 0.2 0.4 0.6 0.8 1.0
Time

File:13zn456l2 Vial:5:51 ID:B4
Method:C:\MASSLYNX\1minLC_MS.olp

Peak ID **Time**
1 0.28
1:(Time: 0.28) Combine (96:111-183:191) 1:MS ES+
8.4e+005

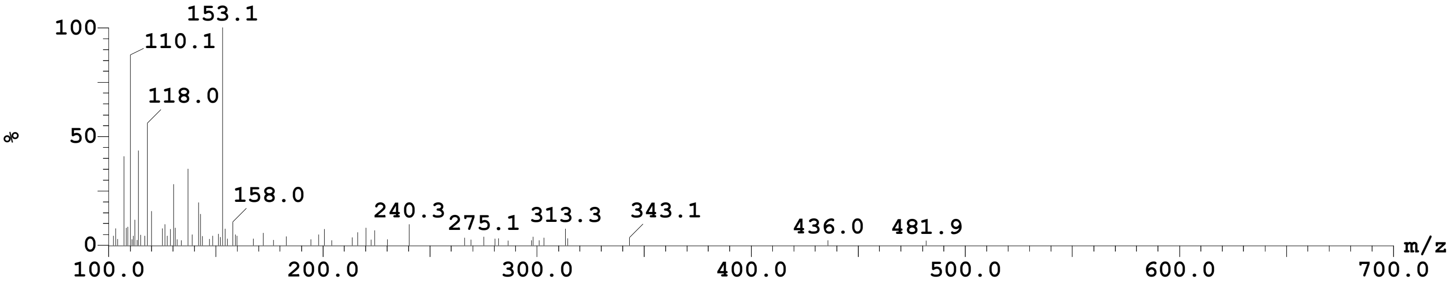

Peak ID **Time**
2 0.34
2:(Time: 0.34) Combine (121:136-(26:34+203:211)) 1:MS ES+
2.0e+006

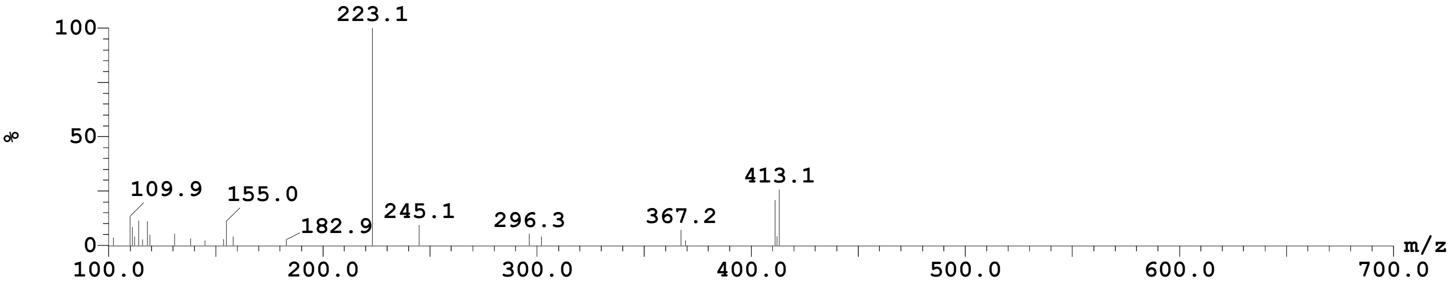

Peak ID **Time**
3 0.37
3:(Time: 0.37) Combine (130:145-(46:53+224:232)) 1:MS ES+
2.9e+006

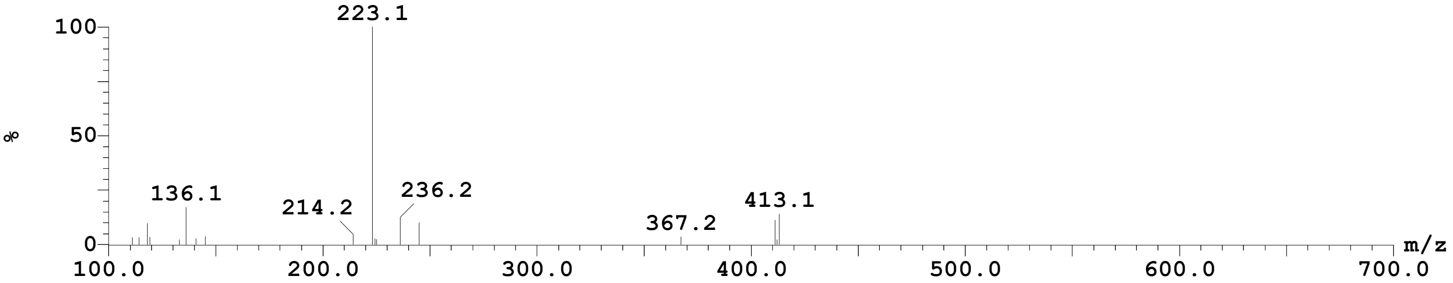

Peak ID **Time**
3 0.37
3:(Time: 0.37) Combine (130:145-(45:53+224:231)) 2:MS ES-
1.3e+005

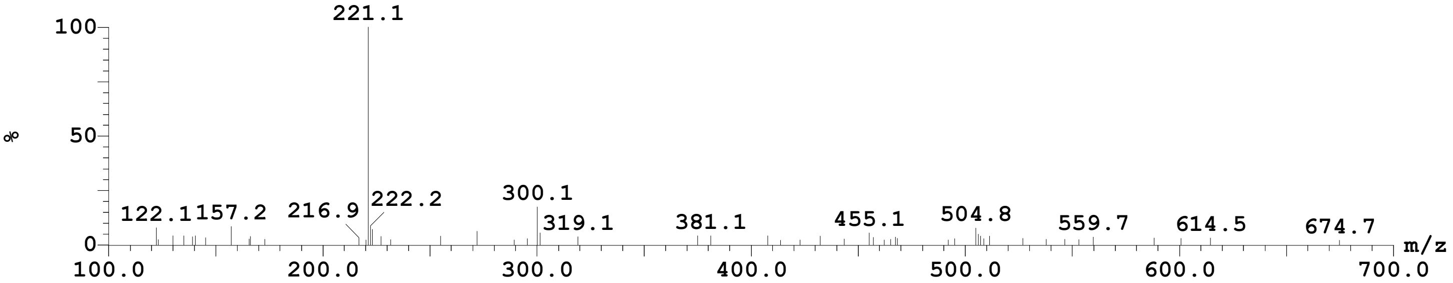

File:13zn456l2 Vial:5:51 ID:B4
Method:C:\MASSLYNX\1minLC_MS.olp

Peak ID **Time**
4 0.43

4:(Time: 0.43) Combine (153:168-(67:74+257:264)) 1:MS ES+ 1.1e+008

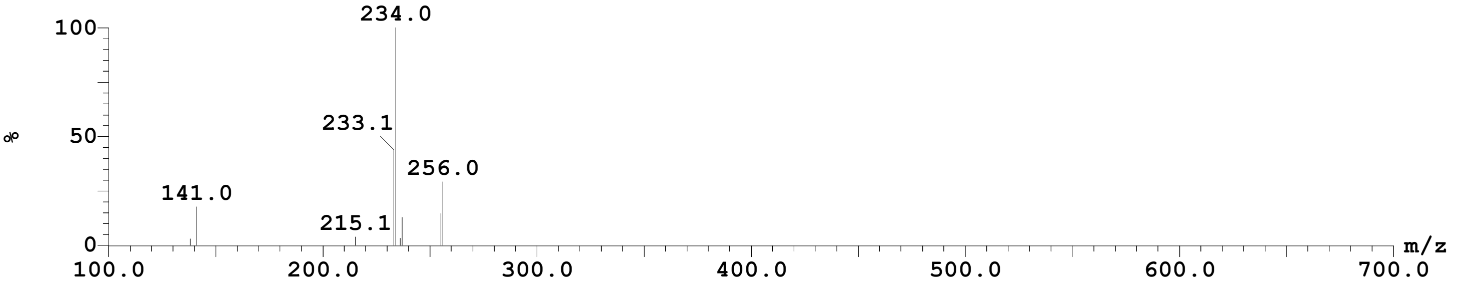

Peak ID **Time**
5 0.53

5:(Time: 0.53) Combine (190:204-(110:117+279:286)) 2:MS ES- 2.6e+005

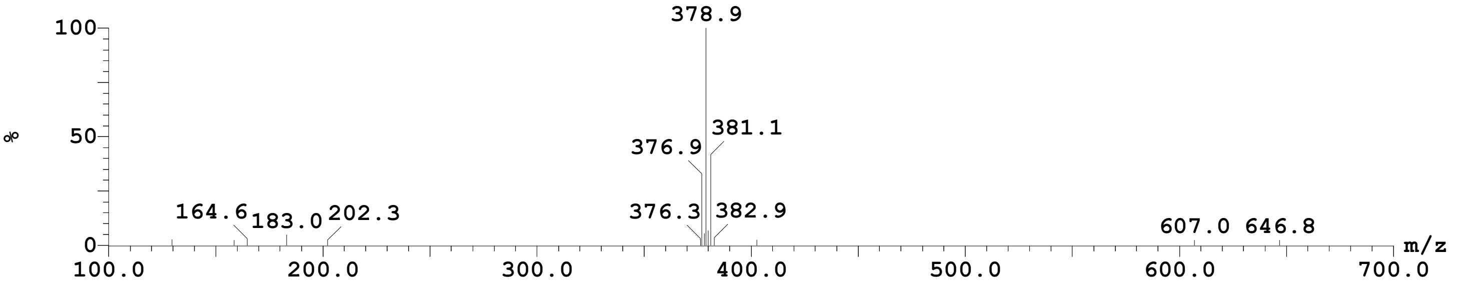

Peak ID **Time**
6 0.57

6:(Time: 0.57) Combine (209:224-(128:136+307:314)) 1:MS ES+ 7.1e+005

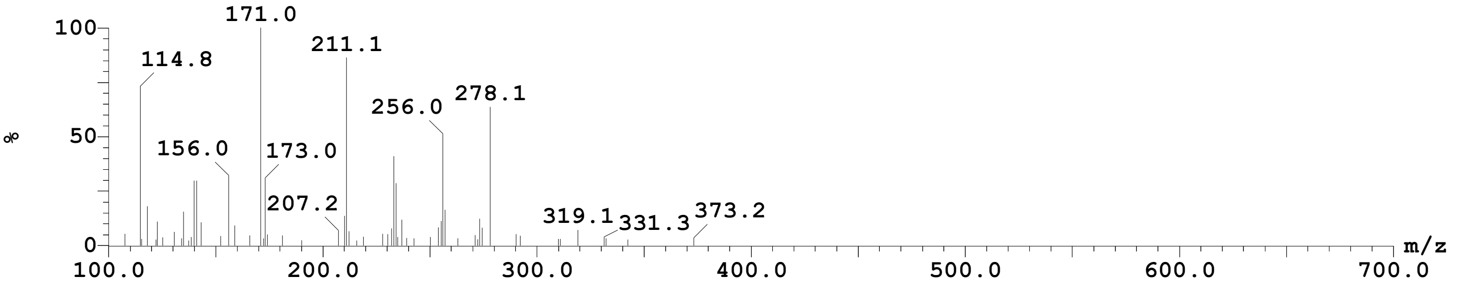

Peak ID **Time**
6 0.57

6:(Time: 0.57) Combine (208:223-(128:135+306:314)) 2:MS ES- 2.6e+005

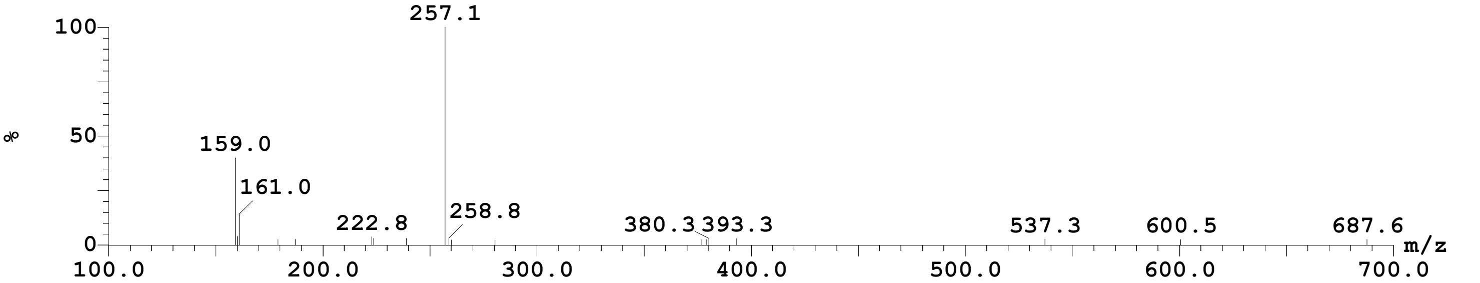

File:13zn456l2 Vial:5:51 ID:B4
Method:C:\MASSLYNX\1minLC_MS.olp

Peak ID **Time**
7 0.65

7: (Time: 0.65) Combine (237:252-(157:164+335:342)) 1:MS ES+ 3.3e+006

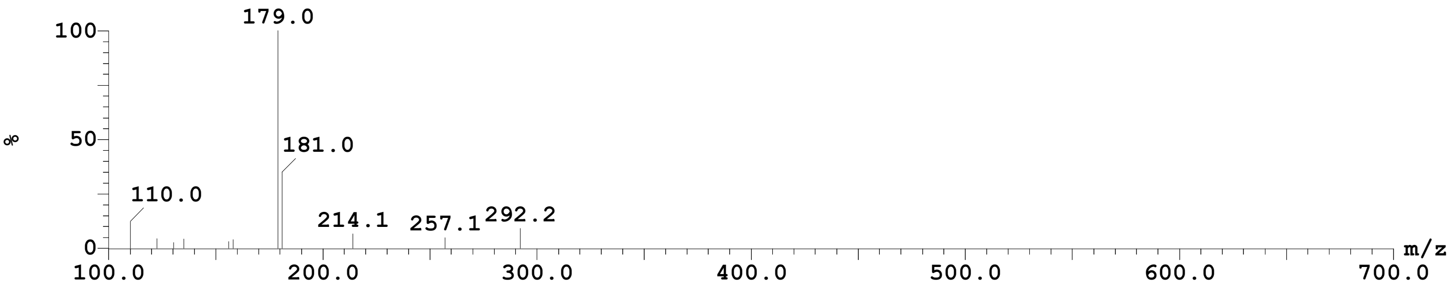

Peak ID **Time**
9 1.00

9: (Time: 1.00) Combine (368:382-270:277) 1:MS ES+ 2.5e+007

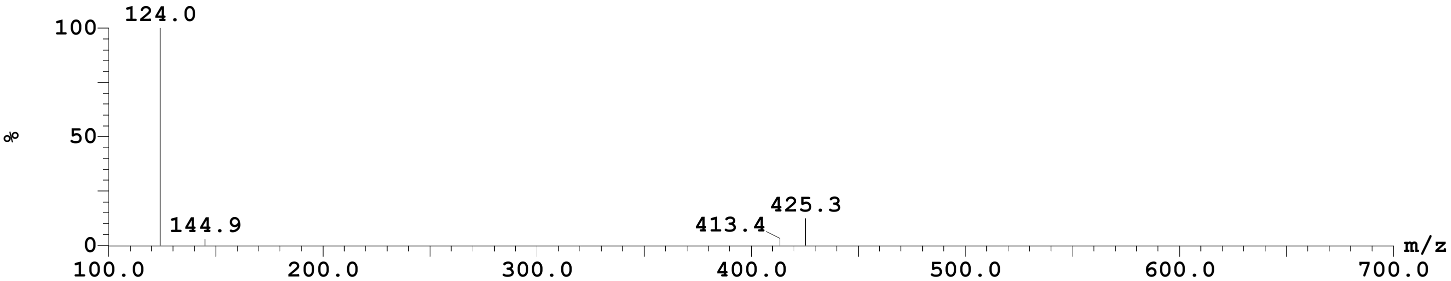

SYNGENTA SQD OPEN ACCESS LC-MS Page 1

File:13zn227l3 Vial:5:51 ID: **B5**

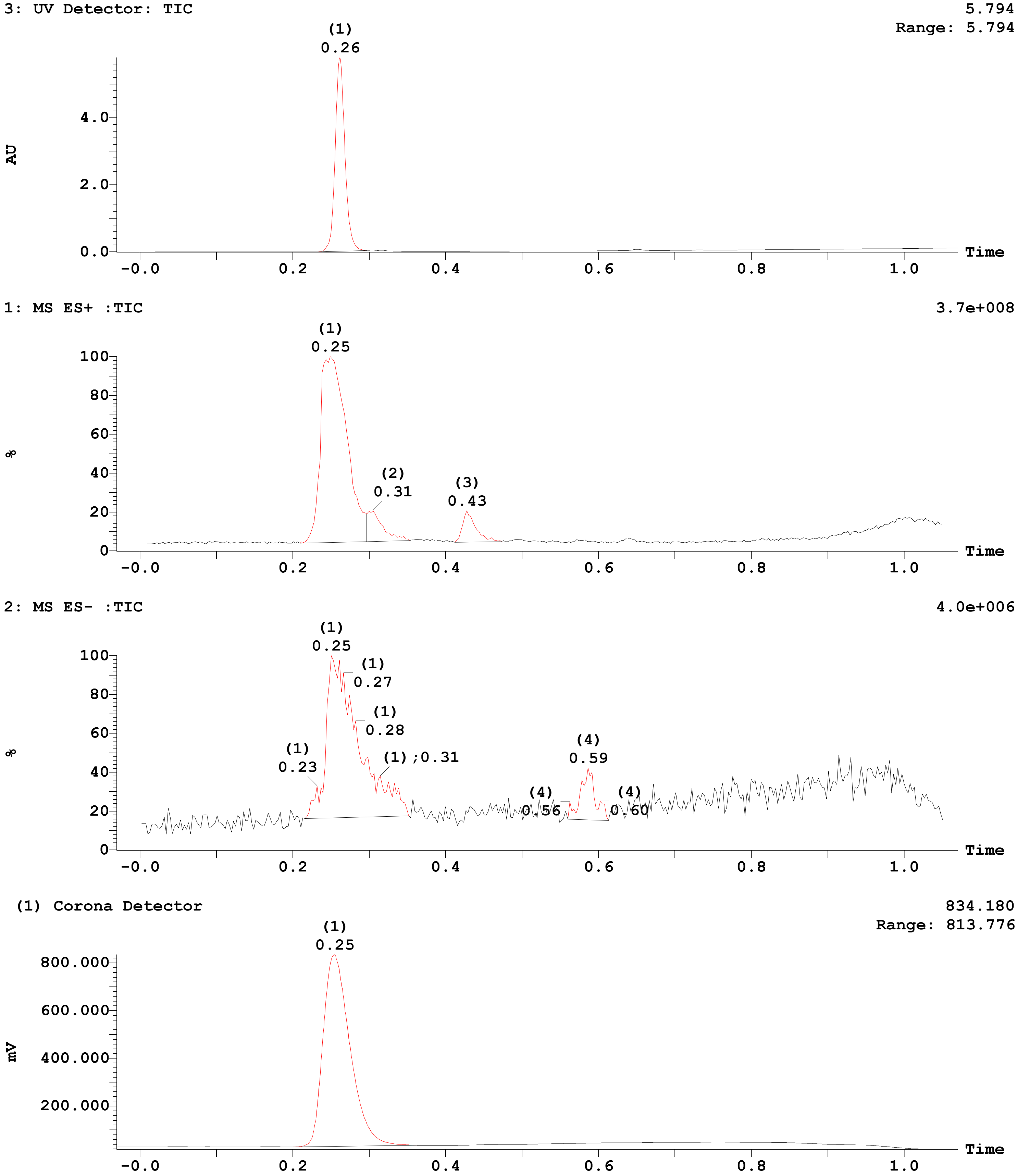

File:13zn227l3 Vial:5:51 ID:B5
Method:C:\MASSLYNX\1minLC_MS.olp

Peak ID Time
1 0.25

1:(Time: 0.26) Combine (91:106-(6:13+187:194)) 1:MS ES+ 1.8e+008

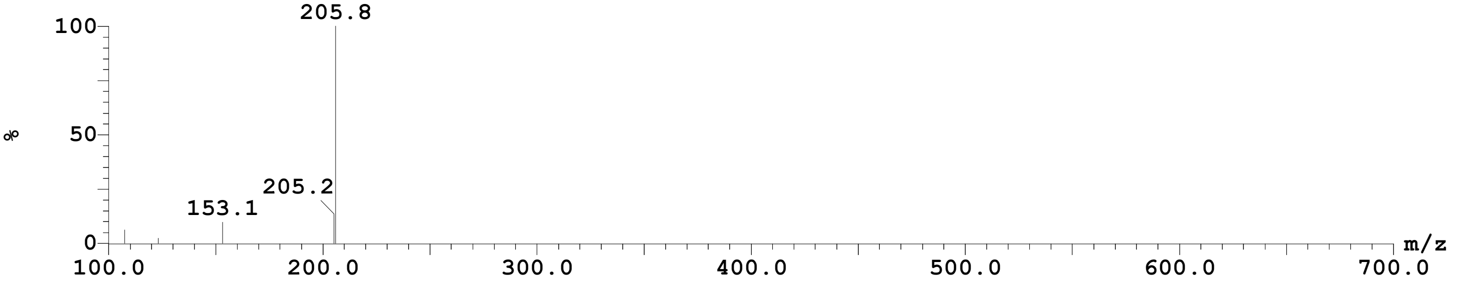

Peak ID Time
1 0.25

1:(Time: 0.26) Combine (91:106-(5:13+186:194)) 2:MS ES- 1.4e+005

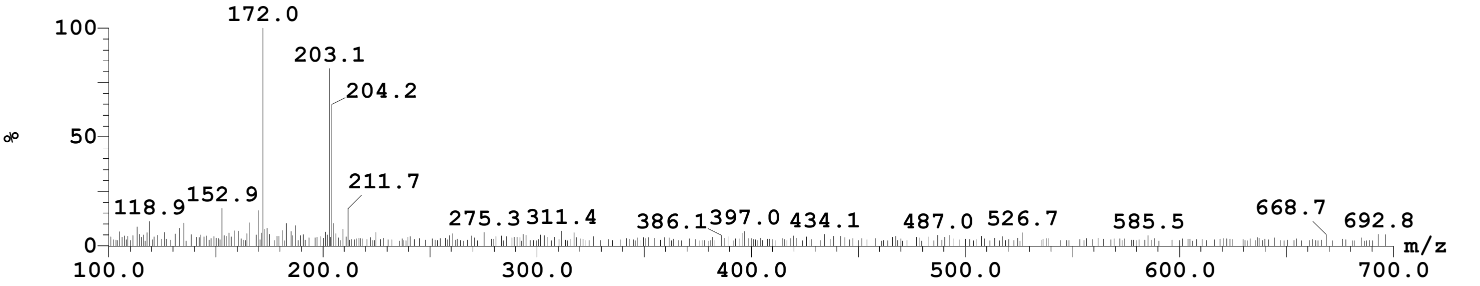

Peak ID Time
2 0.31

2:(Time: 0.31) Combine (107:123-(30:37+208:215)) 1:MS ES+ 1.5e+007

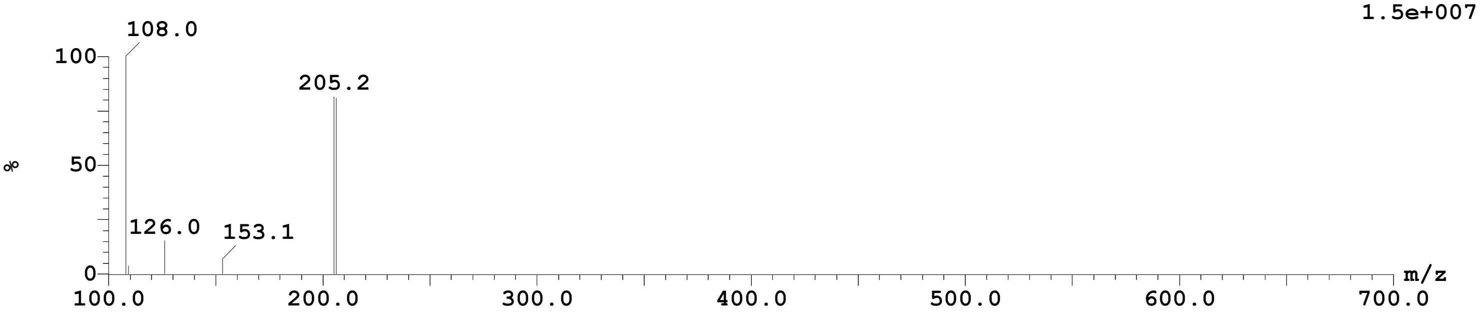

Peak ID Time
3 0.43

3:(Time: 0.43) Combine (153:168-(73:80+253:260)) 1:MS ES+ 1.1e+007

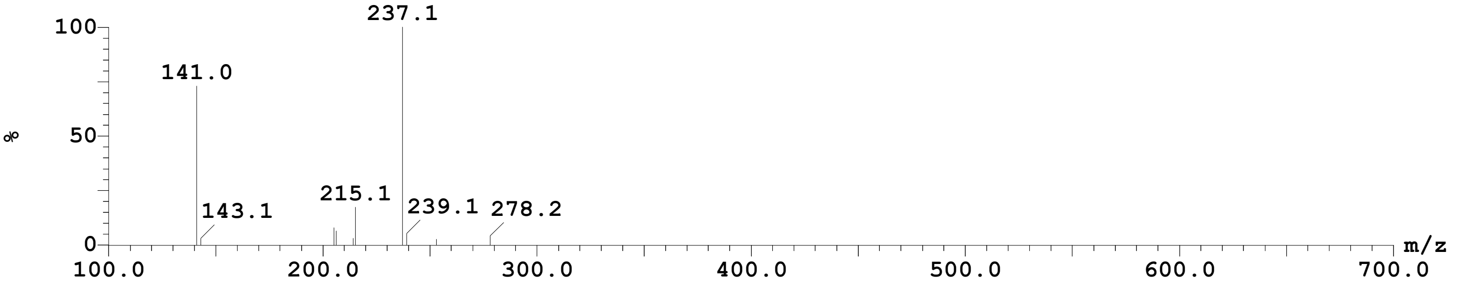

File:13zn227l3 Vial:5:51 ID:B5
Method:C:\MASSLYNX\1minLC_MS.olp

Peak ID	Time
4	0.59

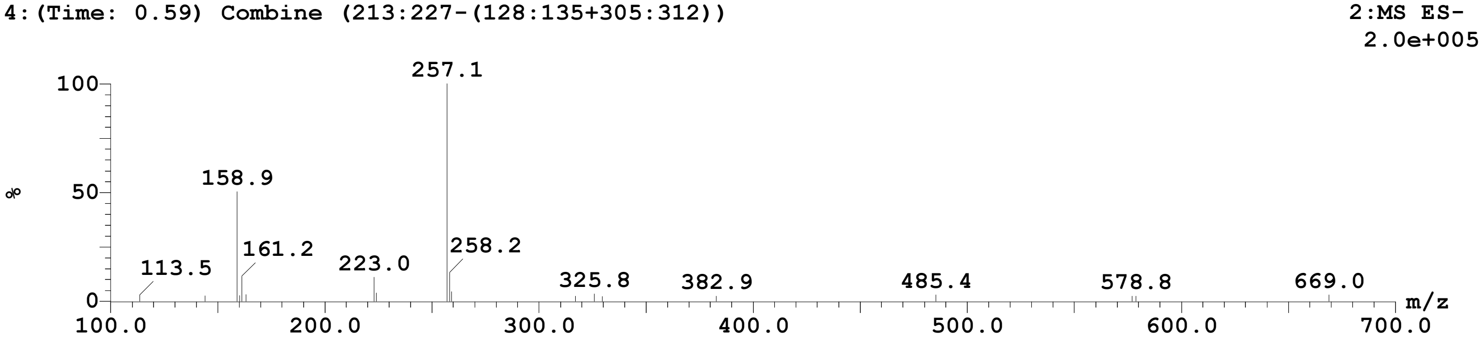

SYNGENTA SQD OPEN ACCESS LC-MS Page 1

File:13zn233l4 Vial:5:52 ID: **B6**

3: UV Detector: TIC 1.619e-1

Range: 1.699e-1

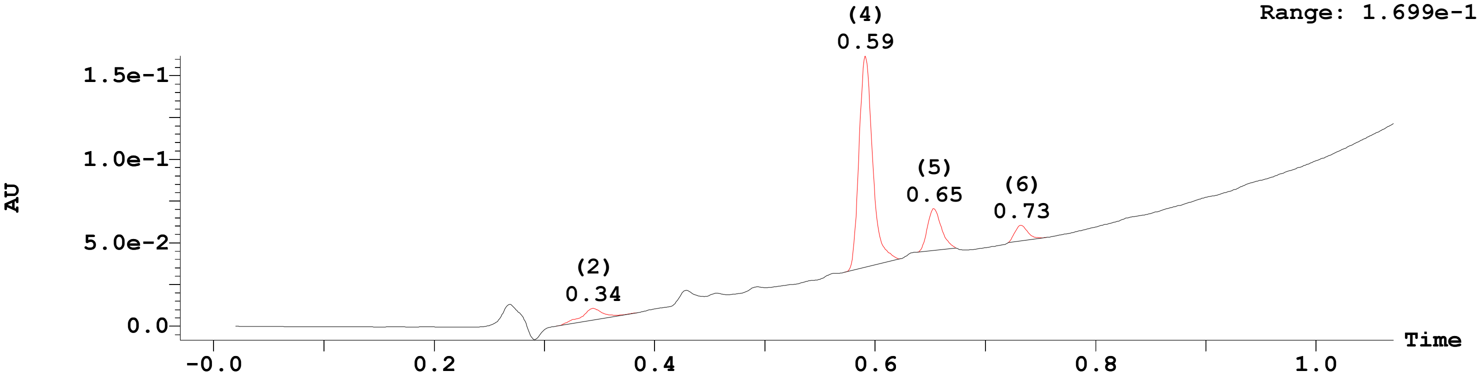

1: MS ES+ :TIC 2.5e+008

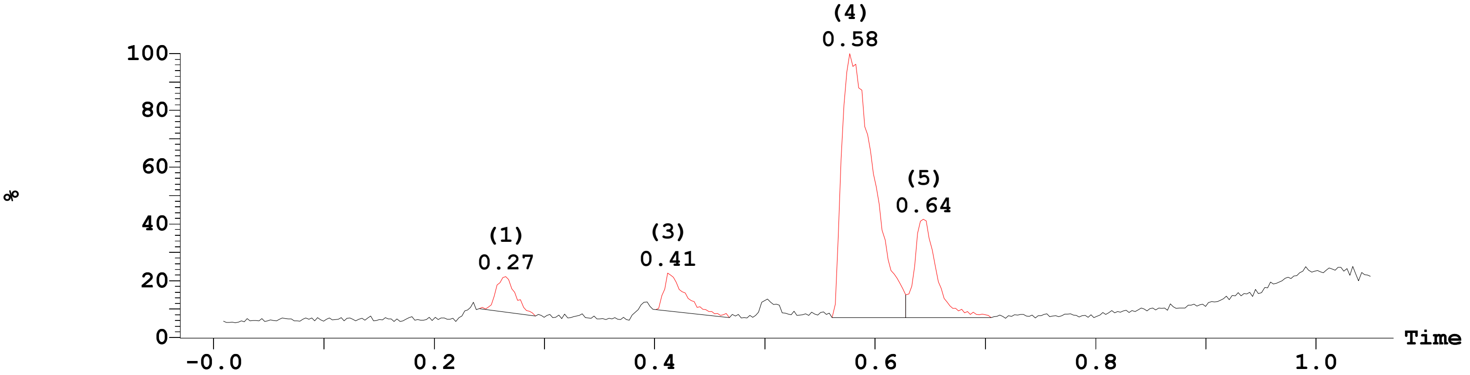

2: MS ES- :TIC 8.2e+006

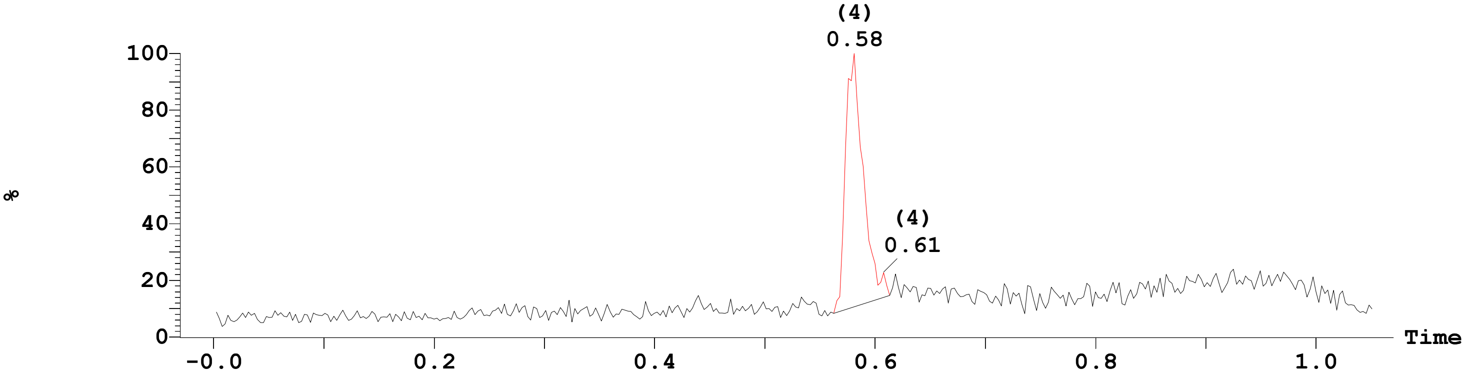

(1) Corona Detector 311.090

Range: 294.017

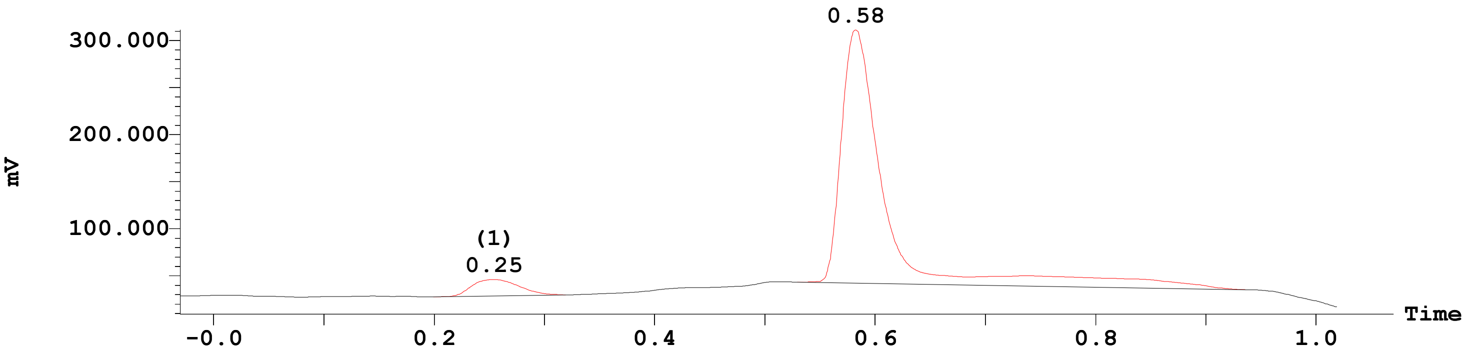

File:13zn233l4 Vial:5:52 ID:B6
Method:C:\MASSLYNX\1minLC_MS.olp

Peak ID Time
1 0.27

1:(Time: 0.27) Combine (92:107-(9:16+185:192)) 1:MS ES+ 1.2e+007

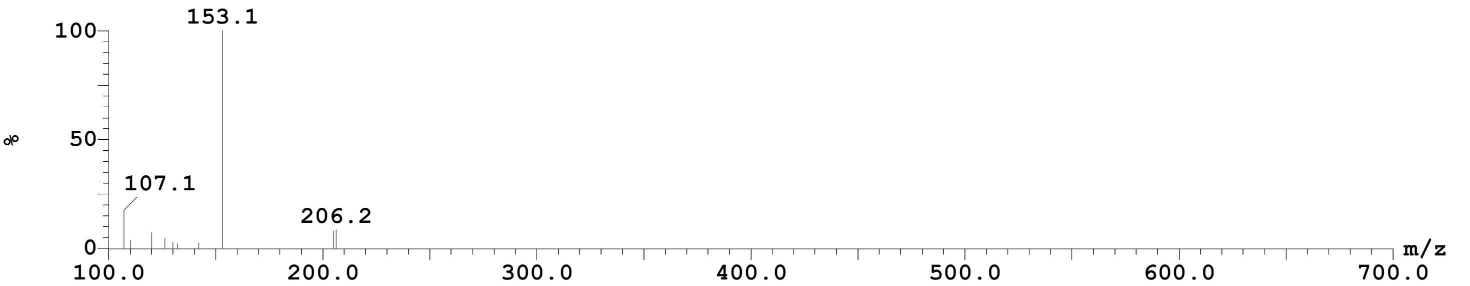

Peak ID Time
2 0.34

2:(Time: 0.34) Combine (122:137-(35:42+220:227)) 1:MS ES+ 6.4e+005

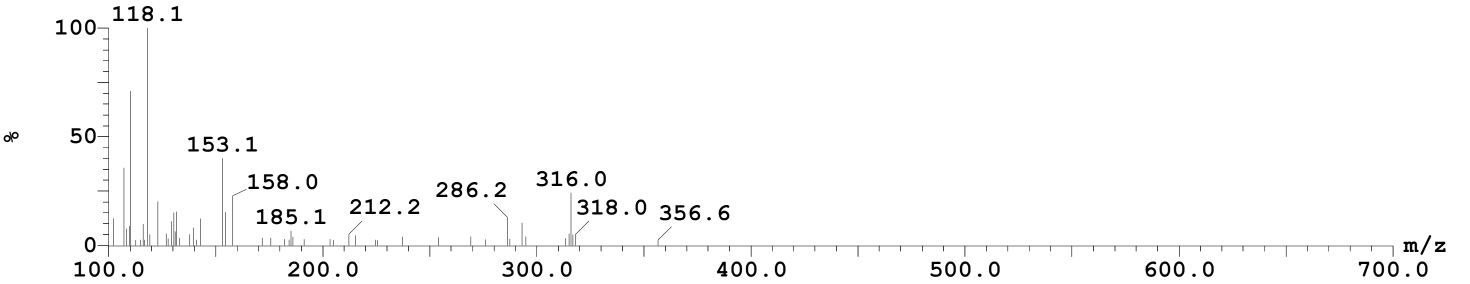

Peak ID Time
2 0.34

2:(Time: 0.34) Combine (122:137-(34:42+219:227)) 2:MS ES- 1.1e+004

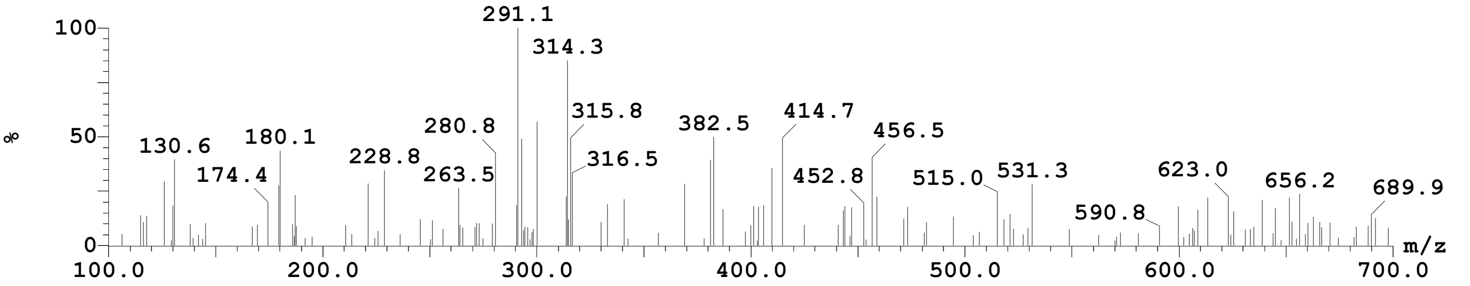

Peak ID Time
3 0.41

3:(Time: 0.41) Combine (147:162-(69:76+251:258)) 1:MS ES+ 9.9e+006

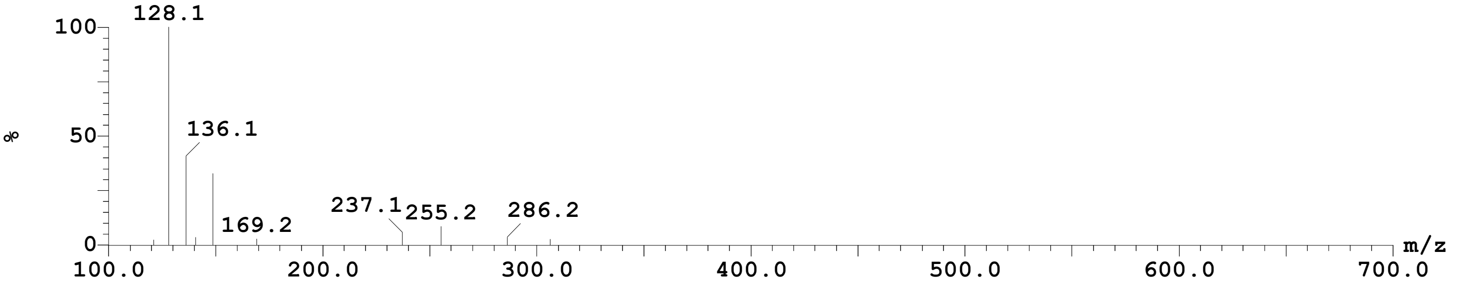

File:13zn233l4 Vial:5:52 ID:B6
Method:C:\MASSLYNX\1minLC_MS.olp

Peak ID **Time**
4 0.58

4:(Time: 0.58) Combine (209:224-(129:136+311:318)) 1:MS ES+
1.1e+008

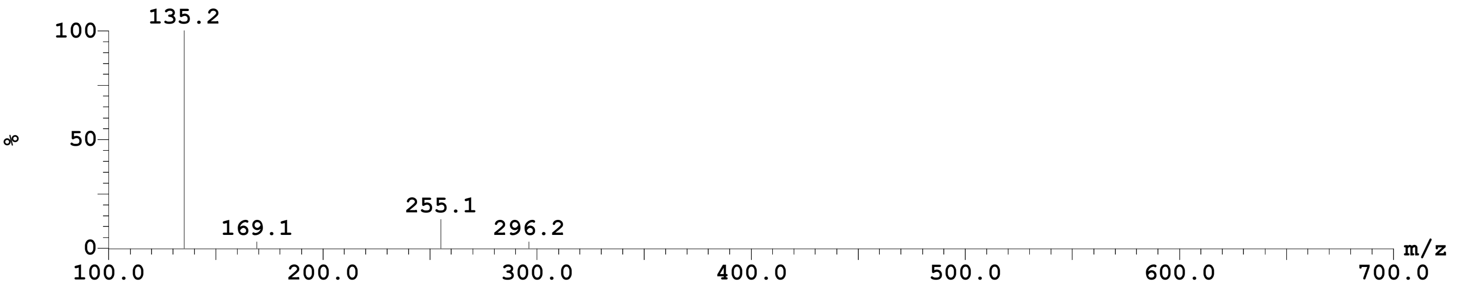

Peak ID **Time**
4 0.58

4:(Time: 0.58) Combine (210:225-(129:136+305:312)) 2:MS ES-
1.4e+006

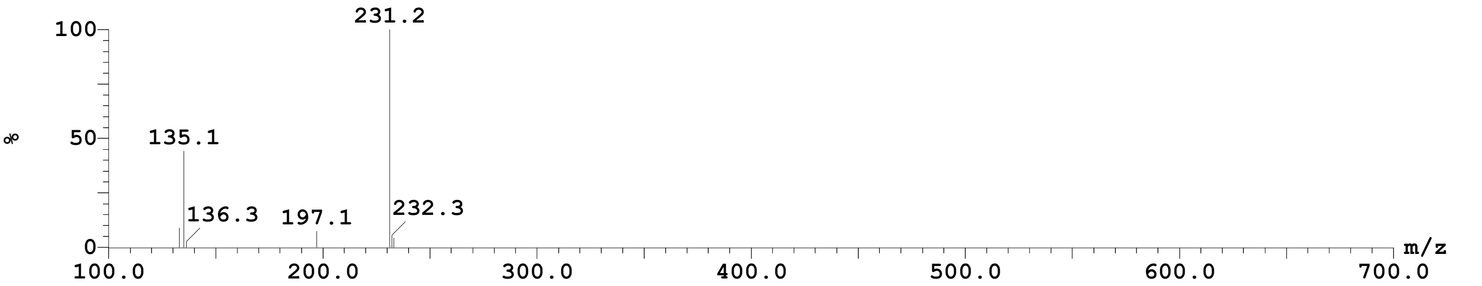

Peak ID **Time**
5 0.64

5:(Time: 0.64) Combine (235:249-(154:161+340:347)) 1:MS ES+
3.7e+007

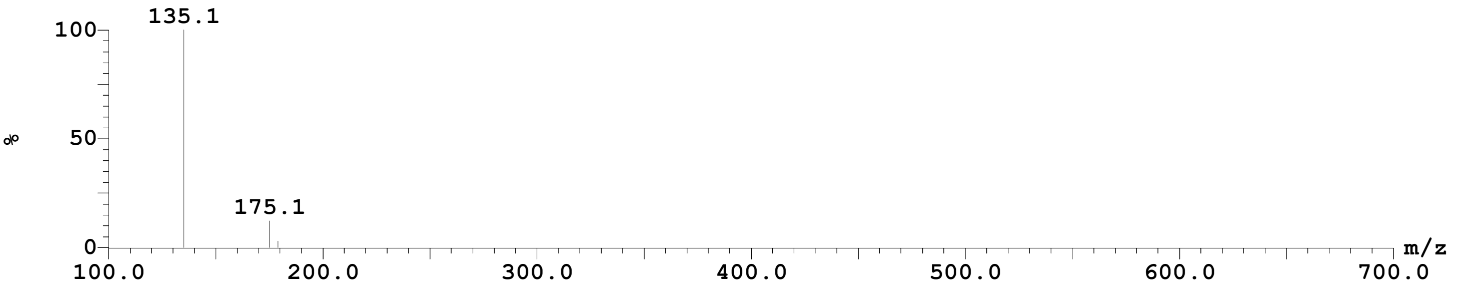

Peak ID **Time**
5 0.64

5:(Time: 0.65) Combine (238:253-(157:164+328:335)) 2:MS ES-
1.8e+004

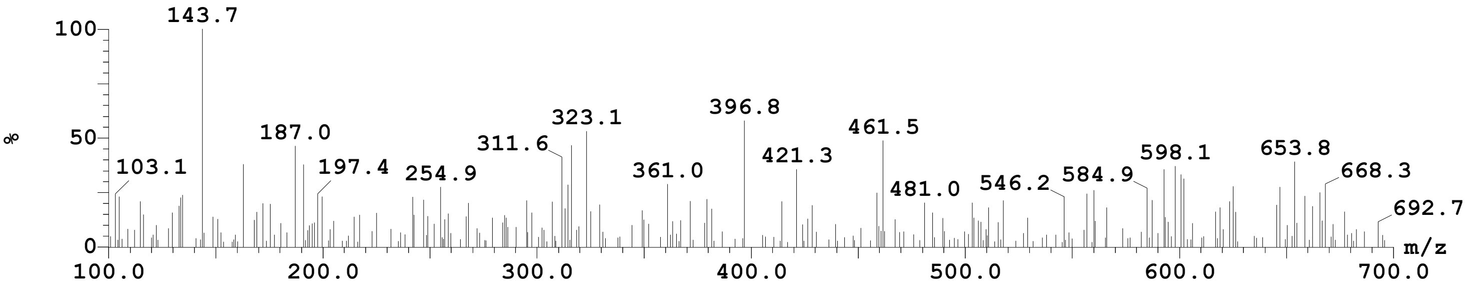

File:13zn233l4 Vial:5:52 ID:B6
Method:C:\MASSLYNX\1minLC_MS.olp

Peak ID Time
6 0.73

6:(Time: 0.73) Combine (268:283-(188:196+359:366)) 1:MS ES+ 8.8e+005

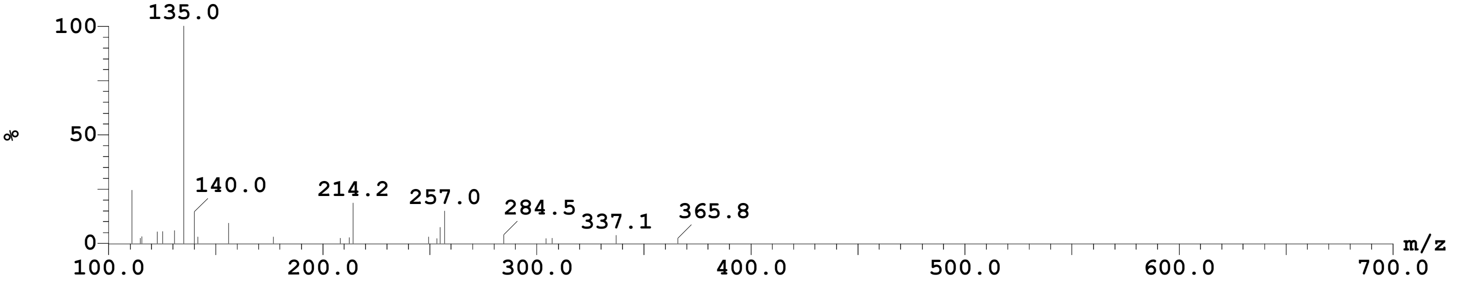

Peak ID Time
6 0.73

6:(Time: 0.73) Combine (267:282-(188:195+358:366)) 2:MS ES- 2.4e+004

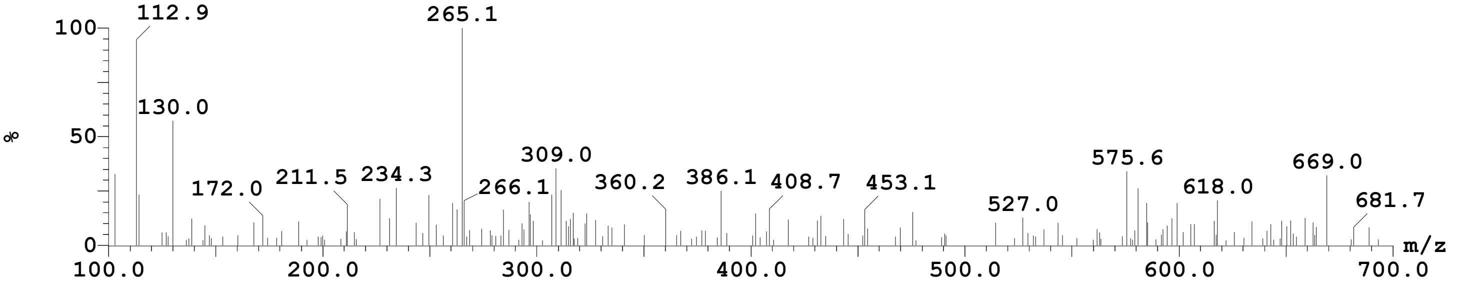

File:13zn467l3 Vial:5:50 ID: **B7**

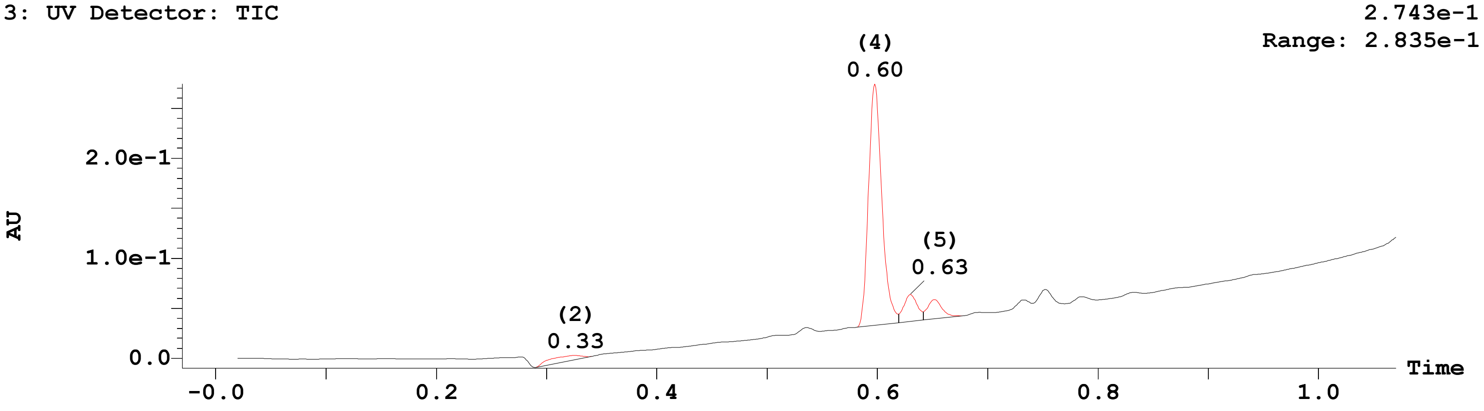

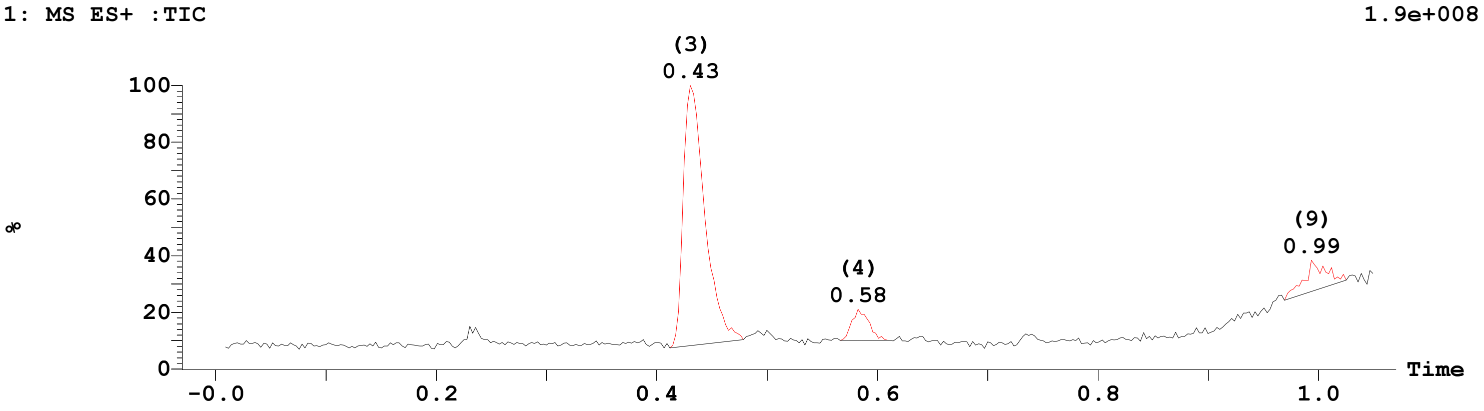

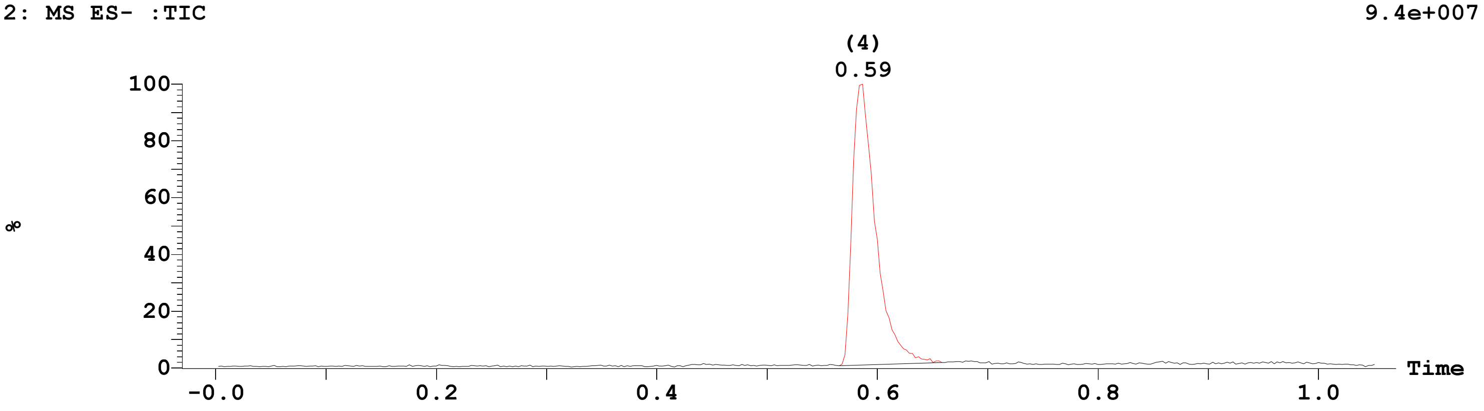

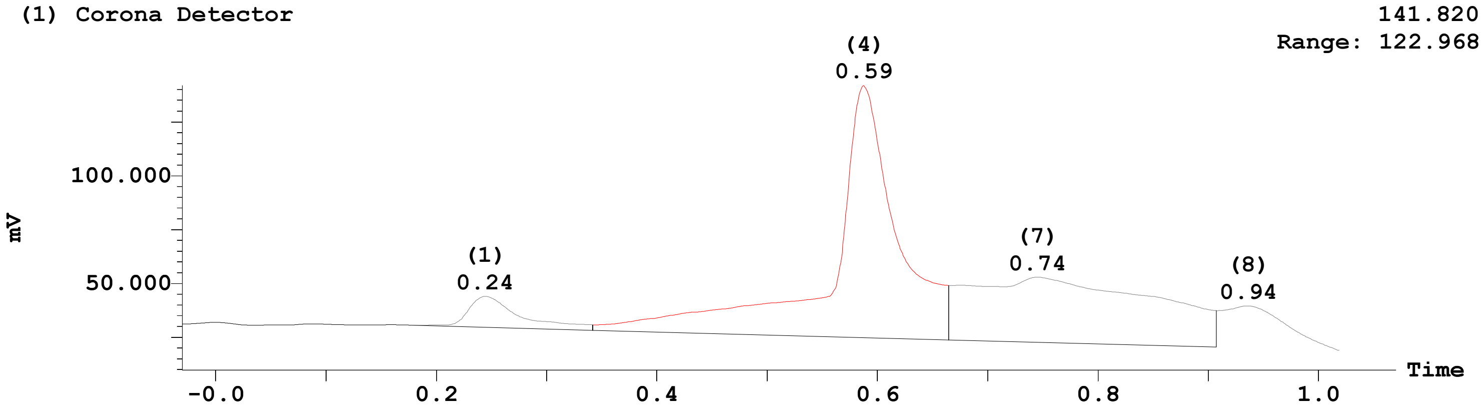

File:13zn467l3 Vial:5:50 ID:B7
Method:C:\MASSLYNX\1minLC_MS.olp

Peak ID **Time**
2 0.33

2:(Time: 0.33) Combine (115:130-(27:34+203:211)) 1:MS ES+ 7.5e+005

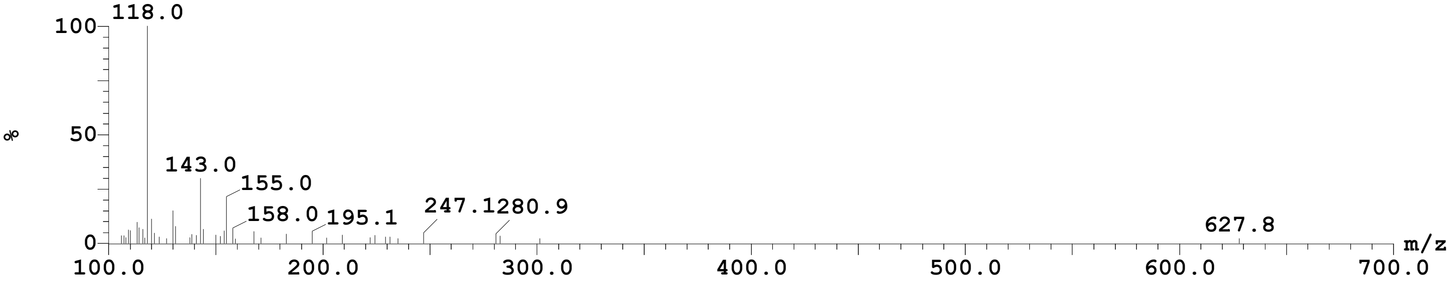

Peak ID **Time**
2 0.33

2:(Time: 0.33) Combine (115:130-(26:34+203:210)) 2:MS ES- 7.9e+003

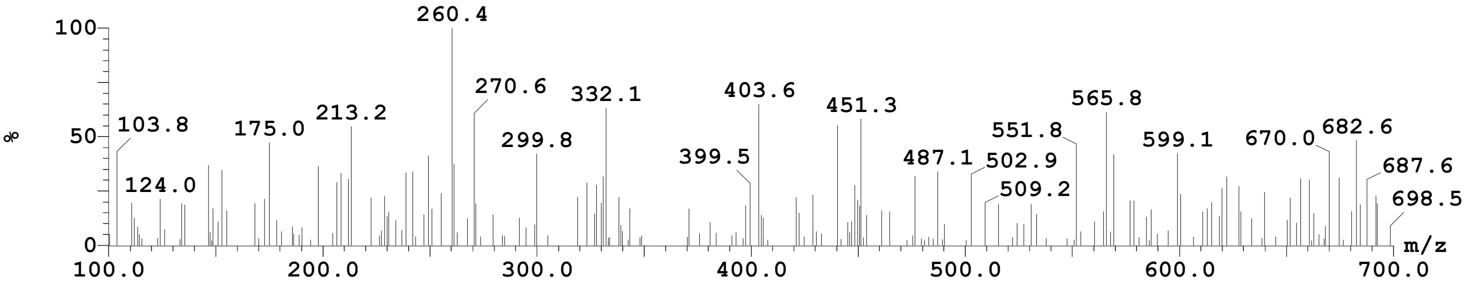

Peak ID **Time**
3 0.43

3:(Time: 0.43) Combine (154:169-(73:80+255:262)) 1:MS ES+ 3.5e+007

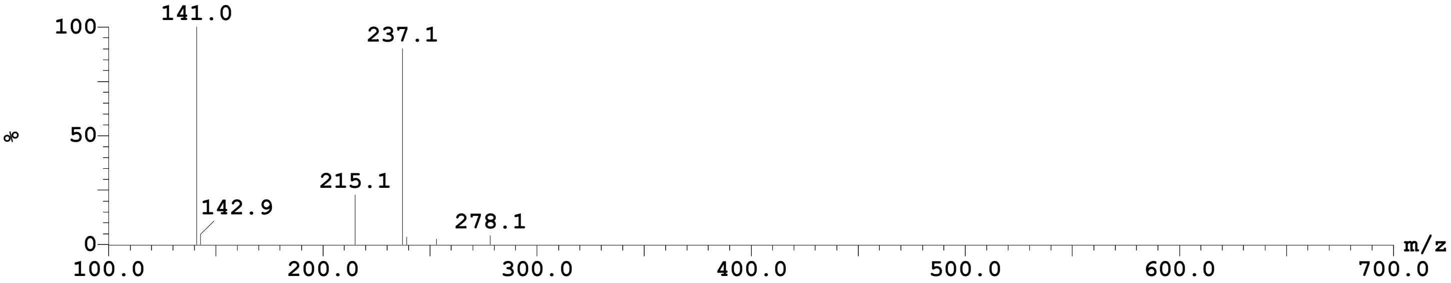

Peak ID **Time**
4 0.58

4:(Time: 0.60) Combine (217:232-(136:143+308:315)) 1:MS ES+ 1.5e+006

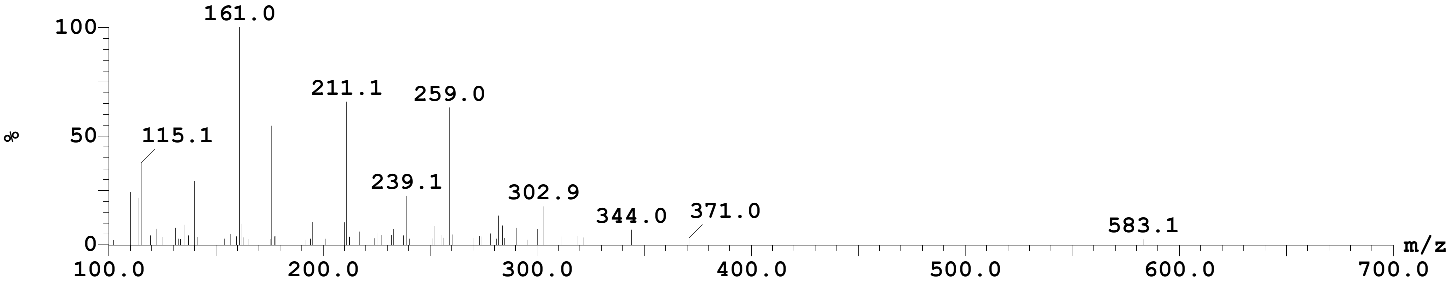

File:13zn467l3 Vial:5:50 ID:B7
Method:C:\MASSLYNX\1minLC_MS.olp

Peak ID **Time**
4 0.58

4:(Time: 0.59) Combine (212:227-(130:137+322:329)) 2:MS ES- 2.3e+007

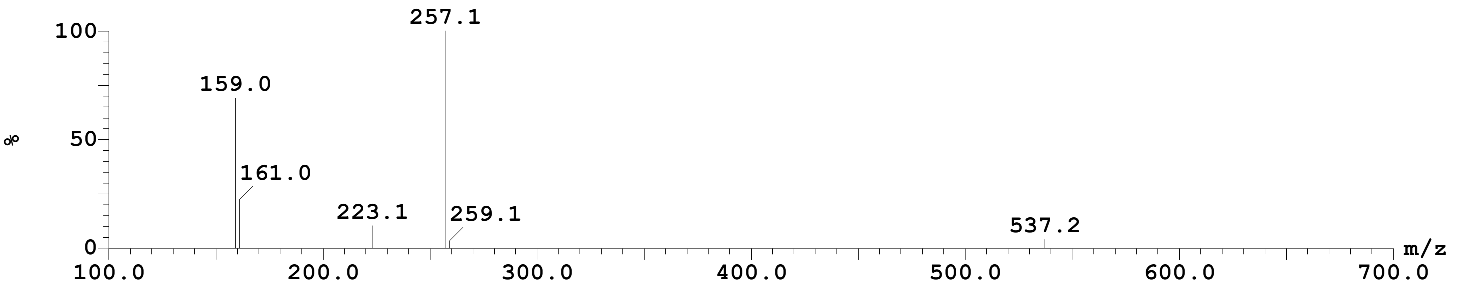

Peak ID **Time**
5 0.63

5:(Time: 0.63) Combine (229:244-(150:158+316:324)) 1:MS ES+ 9.8e+005

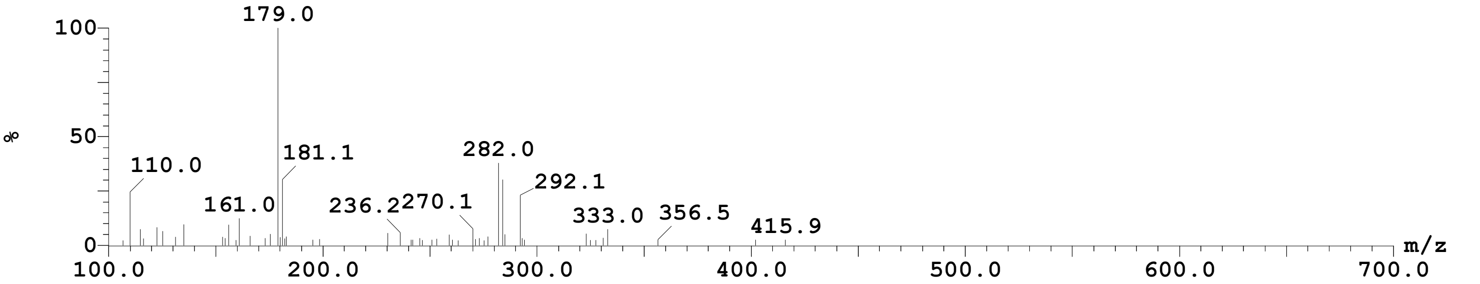

Peak ID **Time**
5 0.63

5:(Time: 0.63) Combine (229:244-(150:157+316:323)) 2:MS ES- 2.4e+006

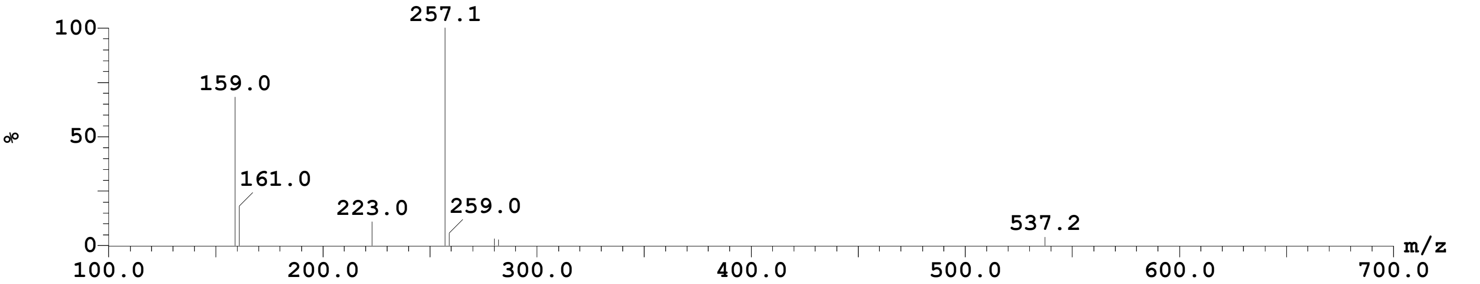

Peak ID **Time**
6 0.65

6:(Time: 0.65) Combine (237:252-(159:166+330:337)) 1:MS ES+ 9.8e+005

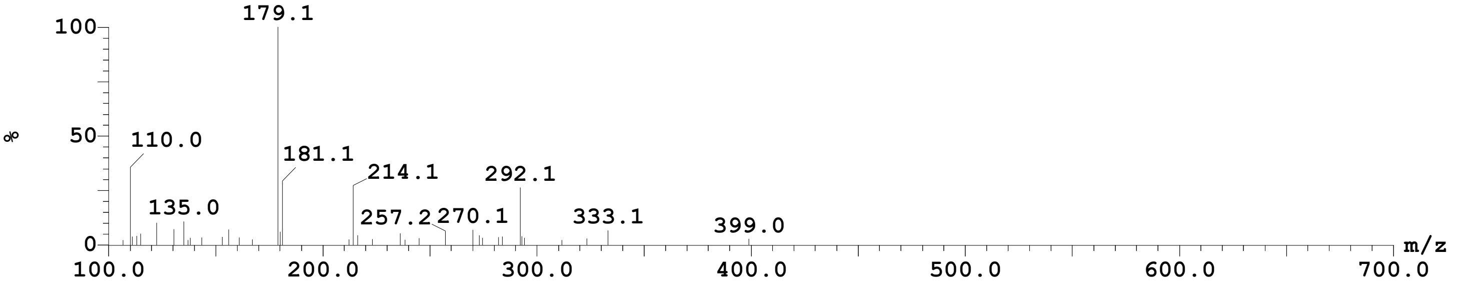

File:13zn467l3 Vial:5:50 ID:B7
Method:C:\MASSLYNX\1minLC_MS.olp

Peak ID **Time**
6 0.65

6: (Time: 0.65) Combine (237:252-(158:166+329:337)) 2:MS ES- 7.8e+005

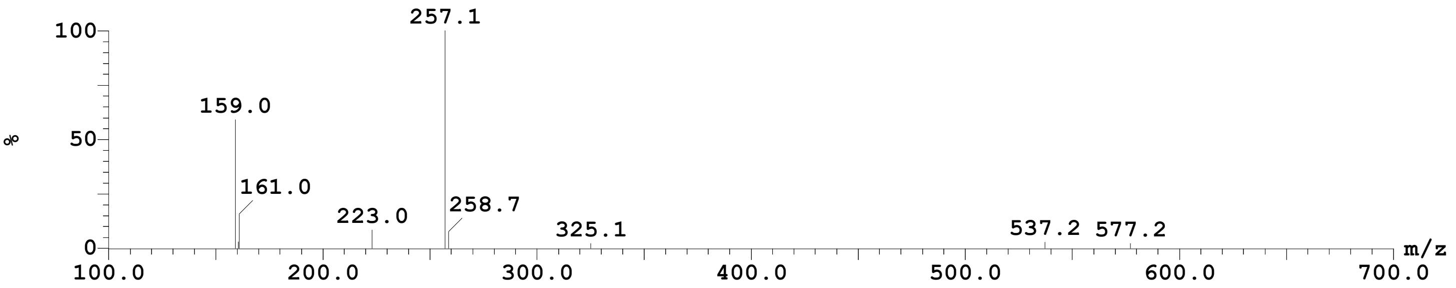

Peak ID **Time**
9 0.99

9: (Time: 0.99) Combine (366:381-282:289) 1:MS ES+ 2.5e+007

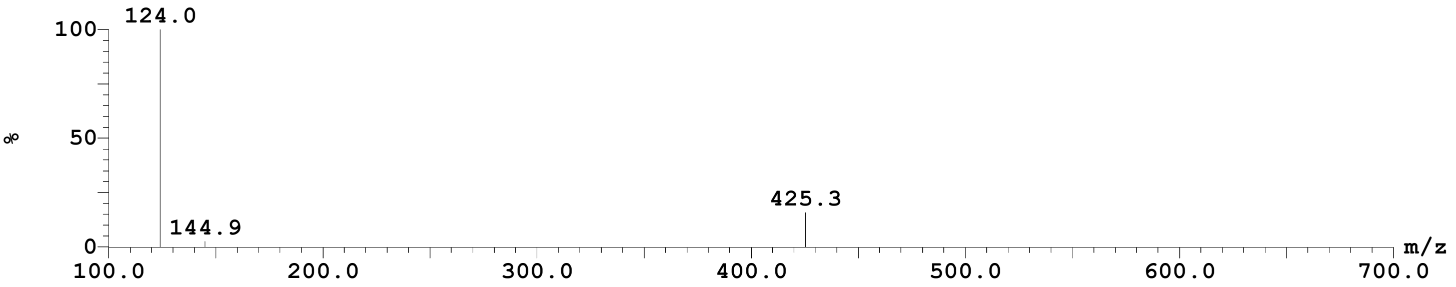

SYNGENTA SQD OPEN ACCESS LC-MS Page 1

File:13zn468l2 Vial:5:52 ID: **B8**

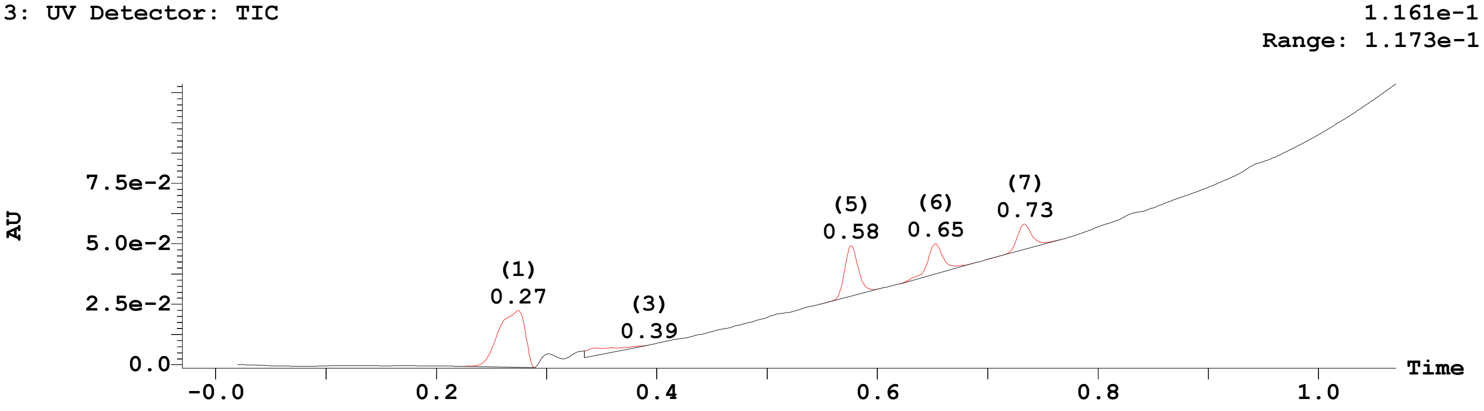

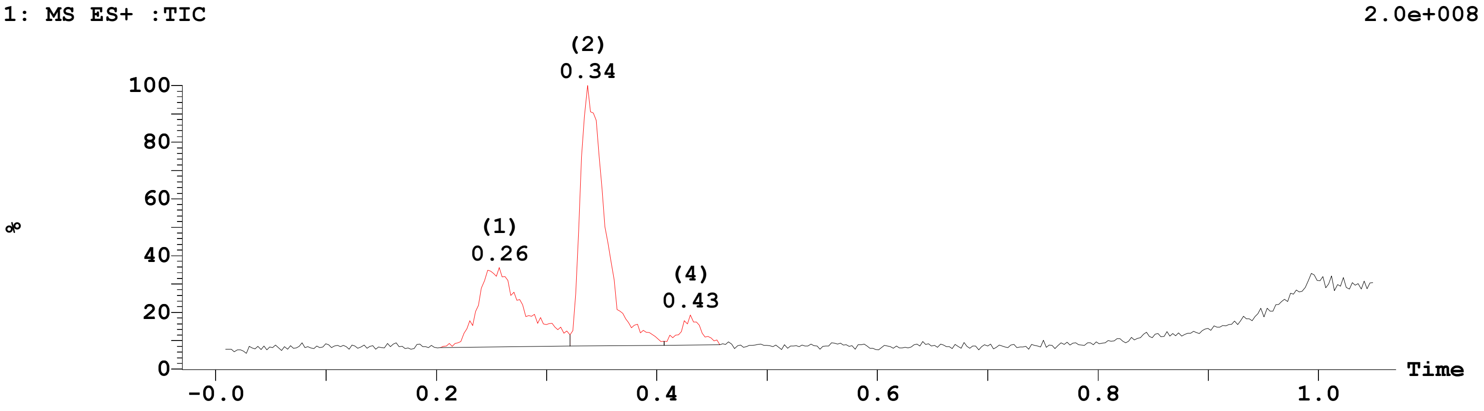

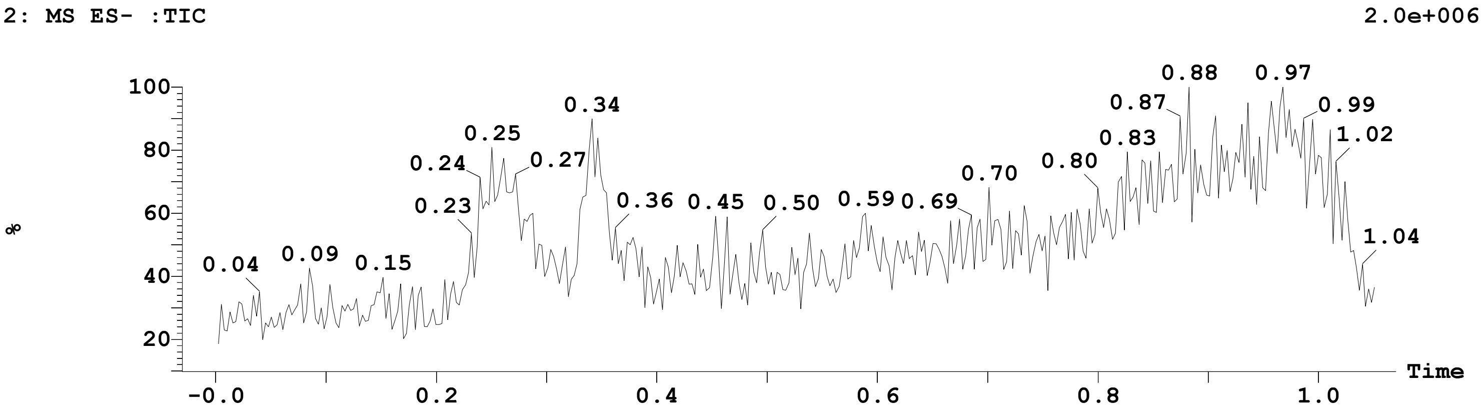

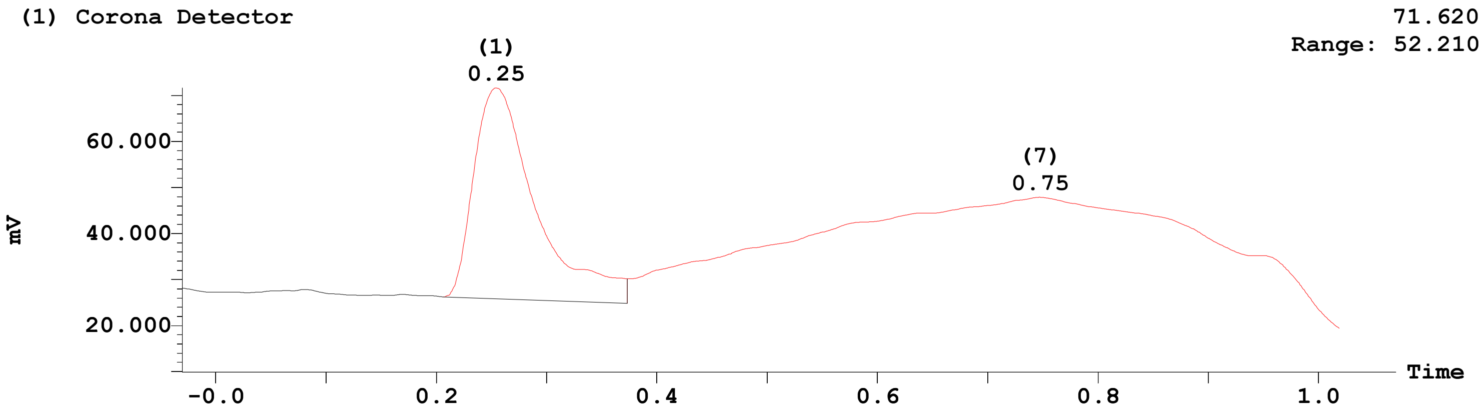

File:13zn468l2 Vial:5:52 ID:B8
Method:C:\MASSLYNX\1minLC_MS.olp

Peak ID **Time**
1 0.26

1:(Time: 0.27) Combine (96:111-(1:7+184:192)) 1:MS ES+
2.6e+006

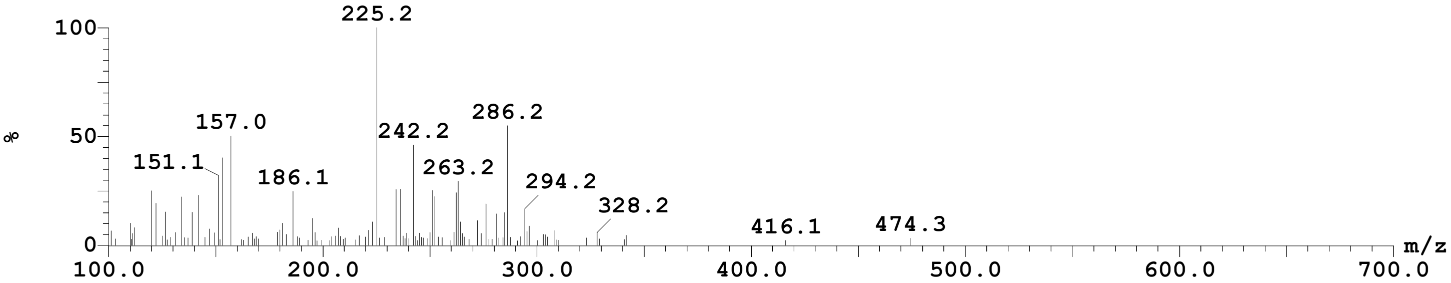

Peak ID **Time**
2 0.34

2:(Time: 0.34) Combine (119:134-(39:46+228:236)) 1:MS ES+
7.4e+007

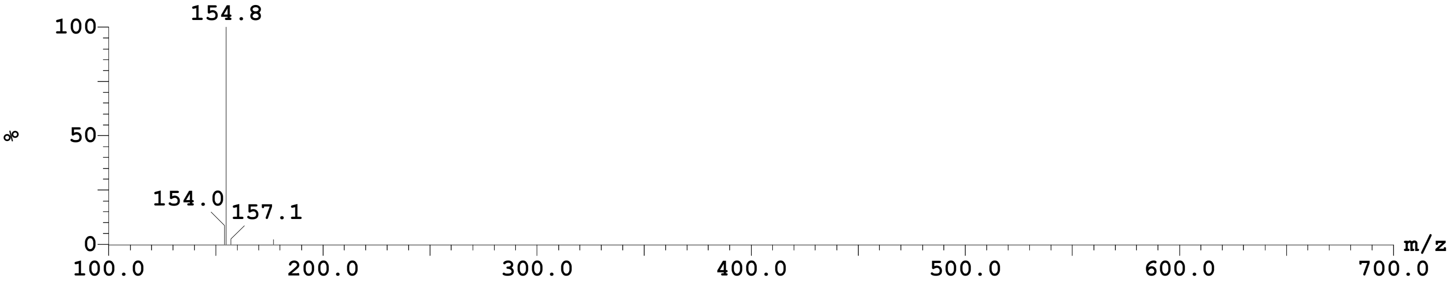

Peak ID **Time**
3 0.39

3:(Time: 0.39) Combine (140:155-(43:51+223:230)) 1:MS ES+
4.4e+006

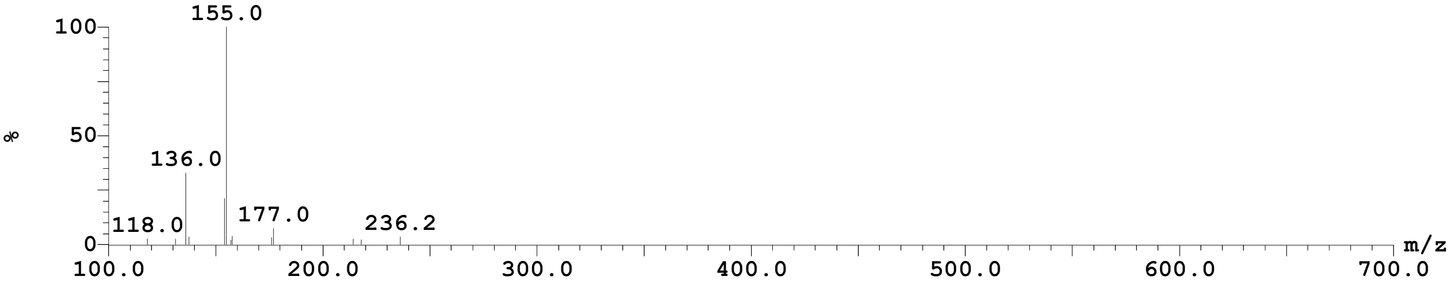

Peak ID **Time**
4 0.43

4:(Time: 0.43) Combine (154:170-(71:78+247:254)) 1:MS ES+
3.3e+006

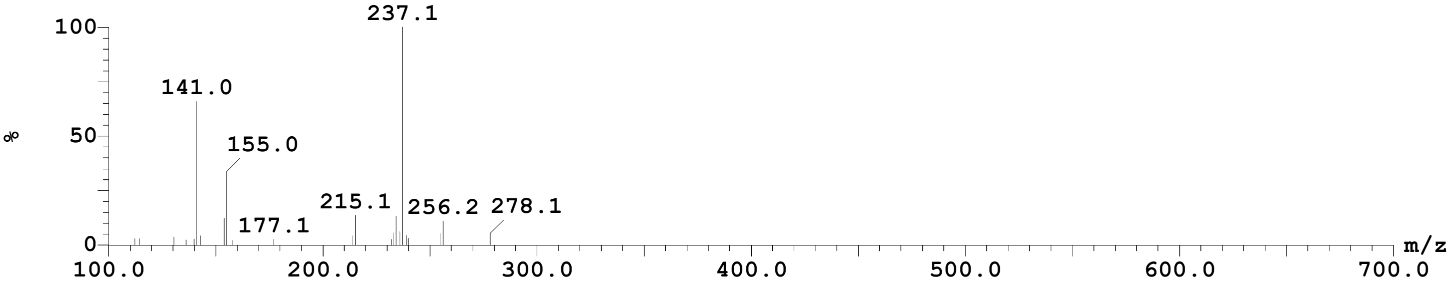

File:13zn468l2 Vial:5:52 ID:B8
Method:C:\MASSLYNX\1minLC_MS.olp

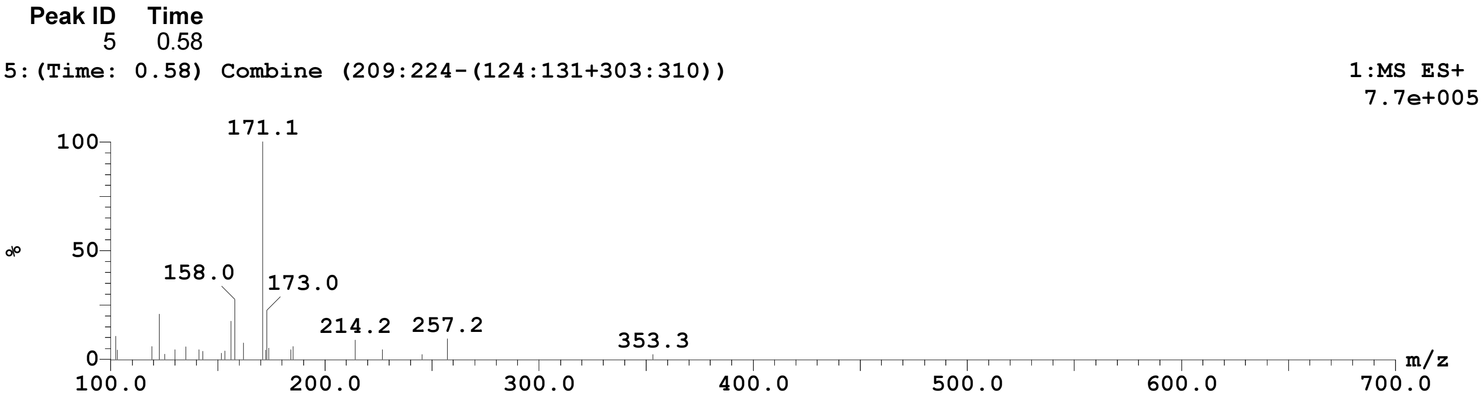

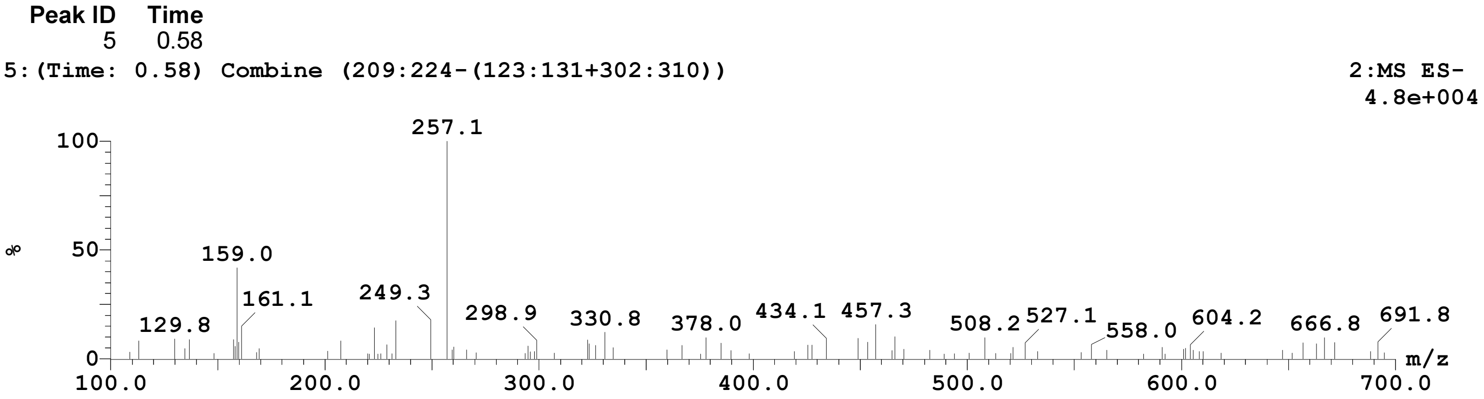

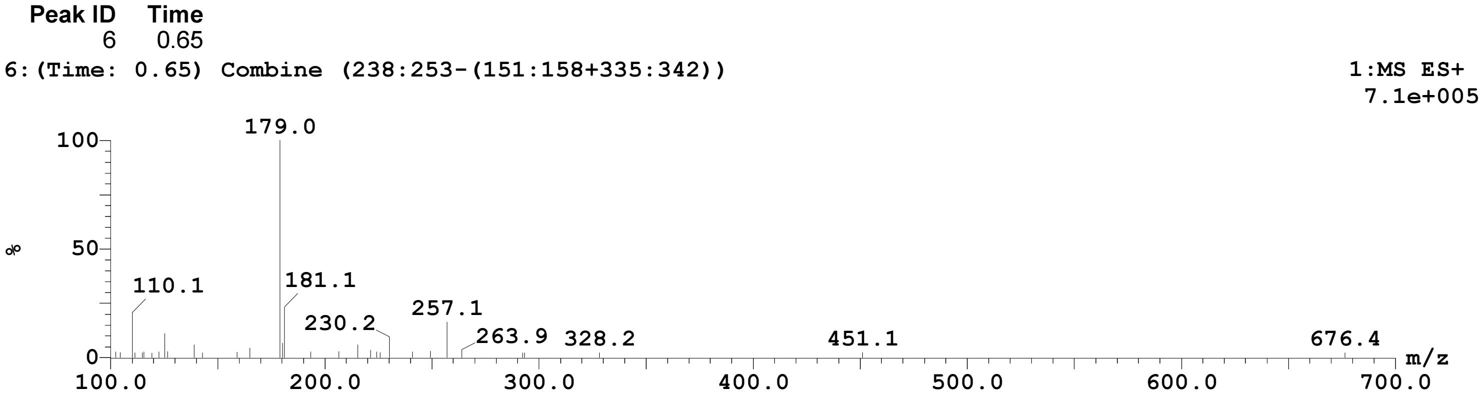

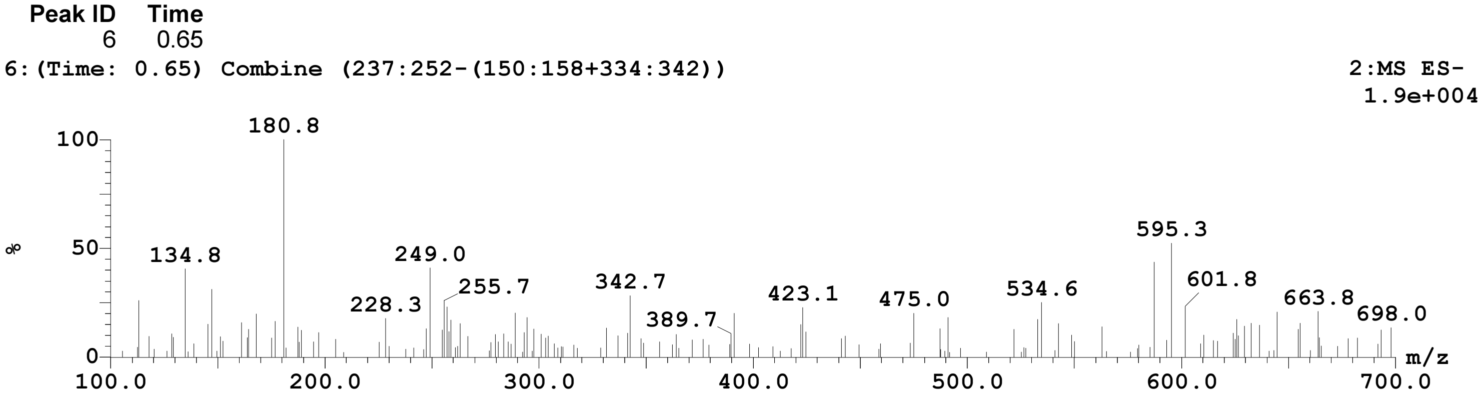

File:13zn468l2 Vial:5:52 ID:B8
Method:C:\MASSLYNX\1minLC_MS.olp

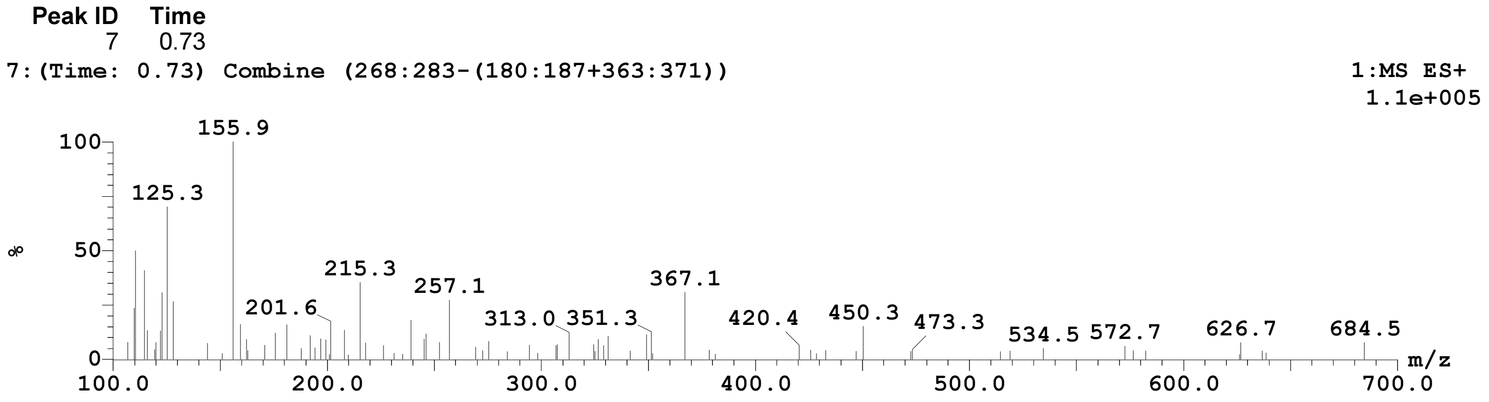

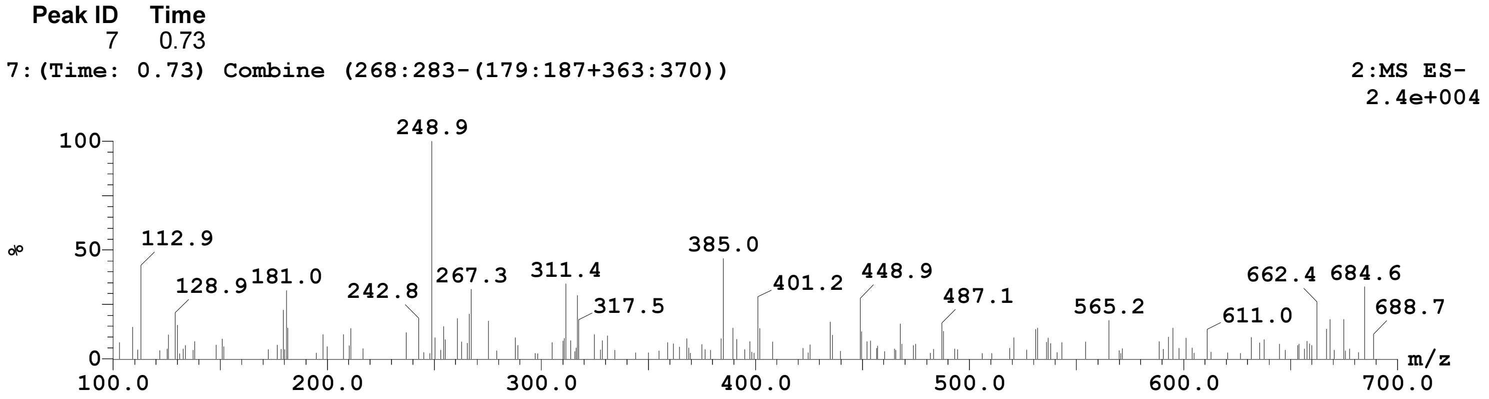

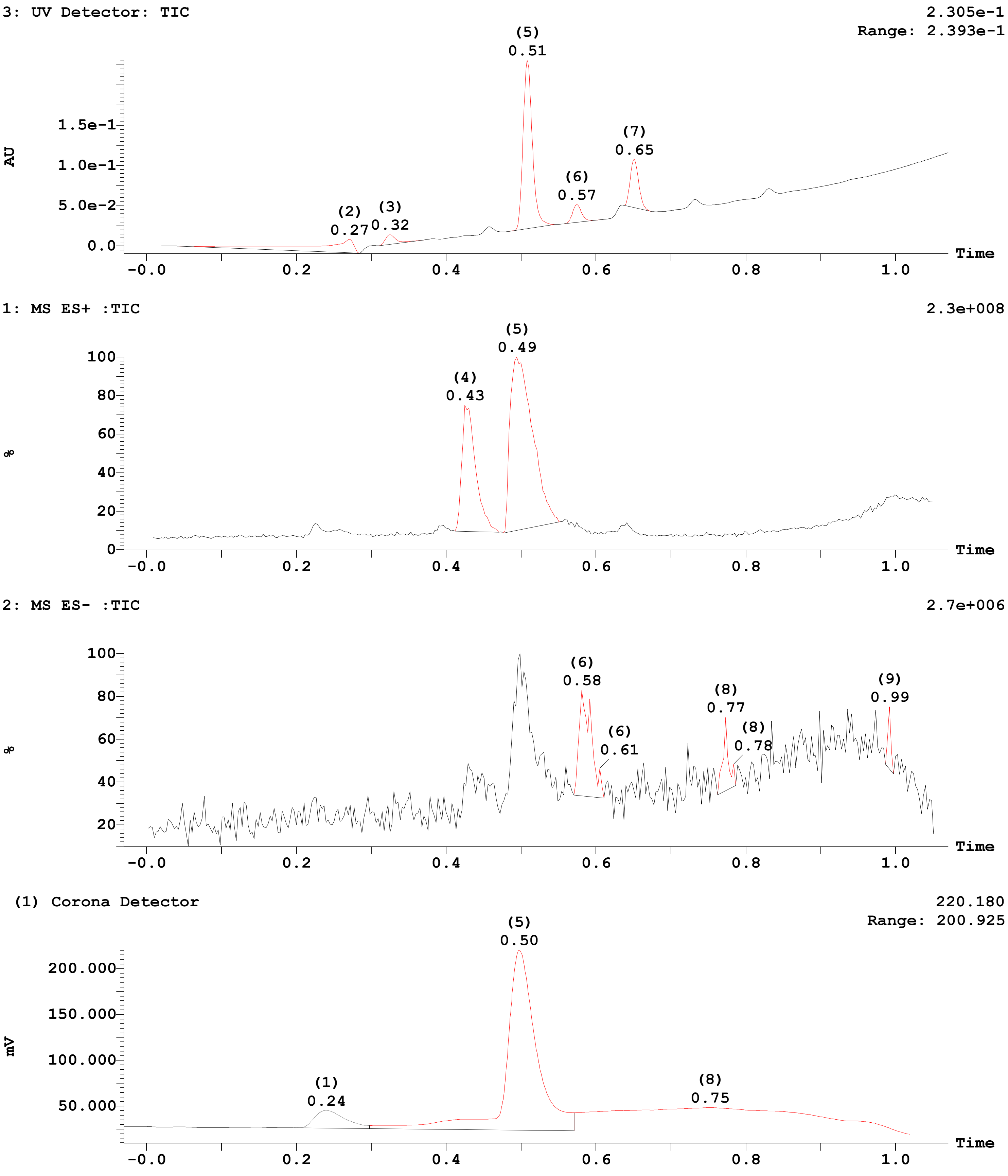
3: UV Detector: TIC
2.305e-1
Range: 2.393e-1
AU
Time
(2) 0.27
(3) 0.32
(5) 0.51
(6) 0.57
(7) 0.65
1: MS ES+ :TIC
2.3e+008
%
(4) 0.43
(5) 0.49
2: MS ES- :TIC
2.7e+006
(6) 0.58
(6) 0.61
(8) 0.77
(8) 0.78
(9) 0.99
(1) Corona Detector
220.180
Range: 200.925
mV
(1) 0.24
(5) 0.50
(8) 0.75

File:13zn480l3 Vial:5:53 ID:B9
Method:C:\MASSLYNX\1minLC_MS.olp

Peak ID **Time**
2 0.27

2:(Time: 0.27) Combine (95:110-182:189) 1:MS ES+ 1.2e+006

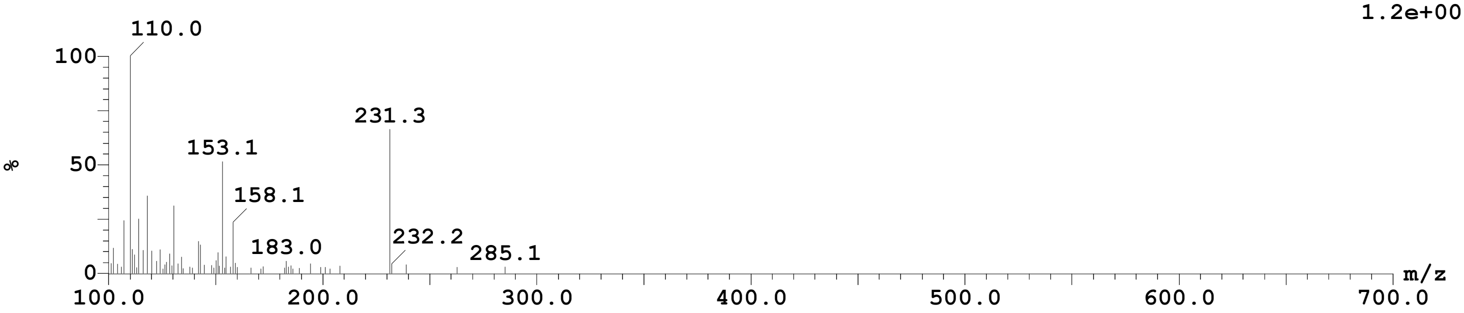

Peak ID **Time**
3 0.32

3:(Time: 0.32) Combine (115:130-(35:42+214:222)) 1:MS ES+ 8.7e+005

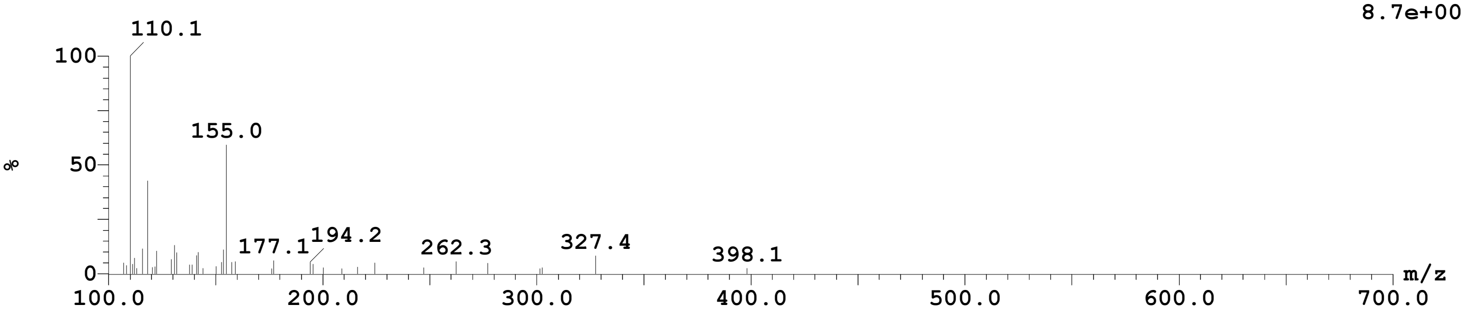

Peak ID **Time**
4 0.43

4:(Time: 0.43) Combine (152:168-(73:80+252:259)) 1:MS ES+ 2.8e+007

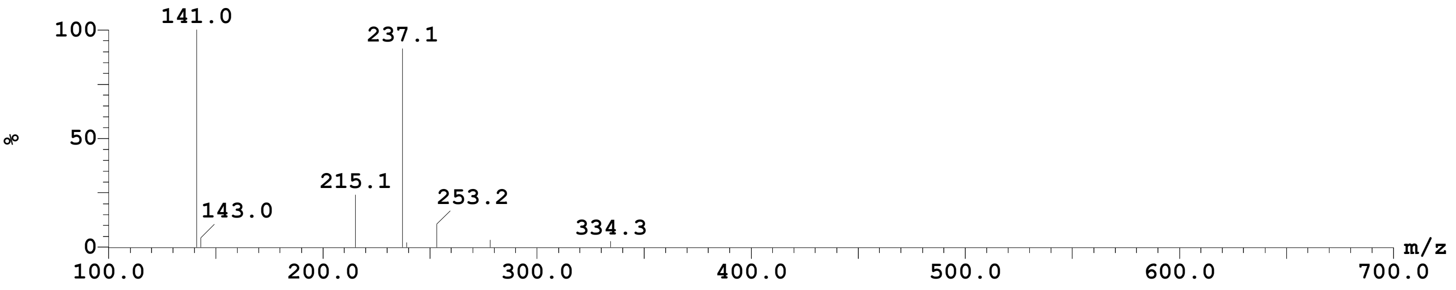

Peak ID **Time**
5 0.49

5:(Time: 0.49) Combine (179:194-(97:104+283:290)) 1:MS ES+ 1.1e+008

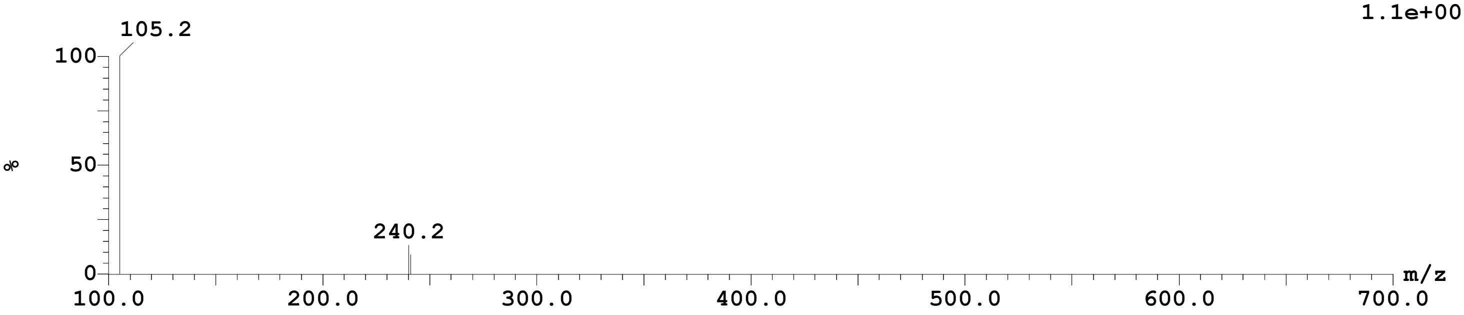

File:13zn480l3 Vial:5:53 ID:B9
Method:C:\MASSLYNX\1minLC_MS.olp

Peak ID **Time**
5 0.49

5:(Time: 0.51) Combine (183:198-(99:107+280:287)) 2:MS ES- 6.6e+004

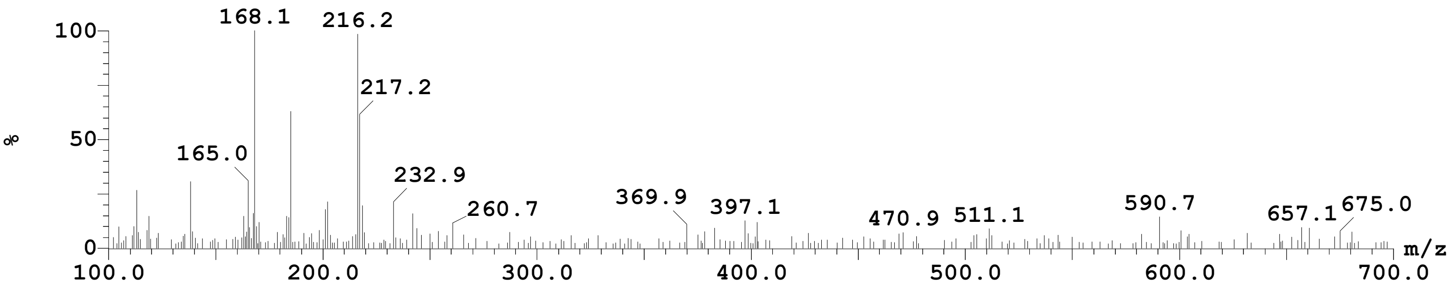

Peak ID **Time**
6 0.58

6:(Time: 0.57) Combine (208:223-(127:135+303:311)) 1:MS ES+ 5.4e+006

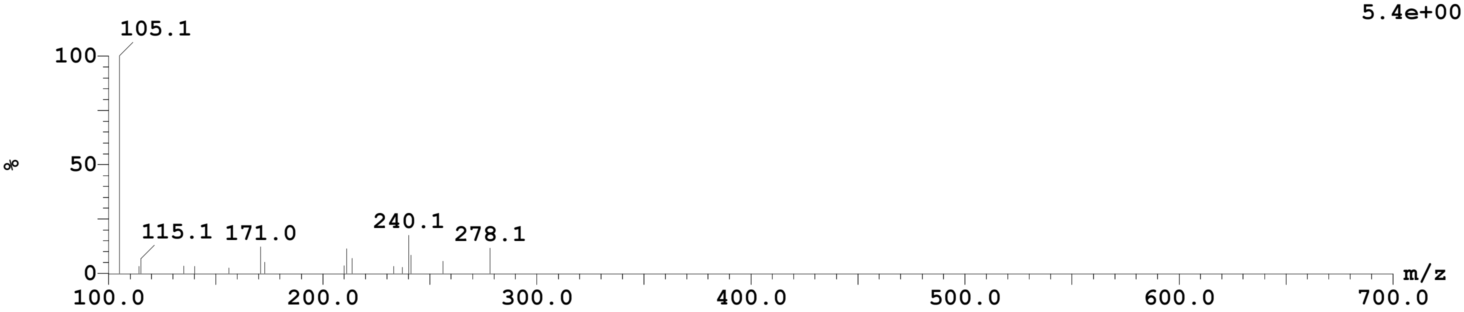

Peak ID **Time**
6 0.58

6:(Time: 0.58) Combine (210:225-(132:139+304:311)) 2:MS ES- 2.4e+005

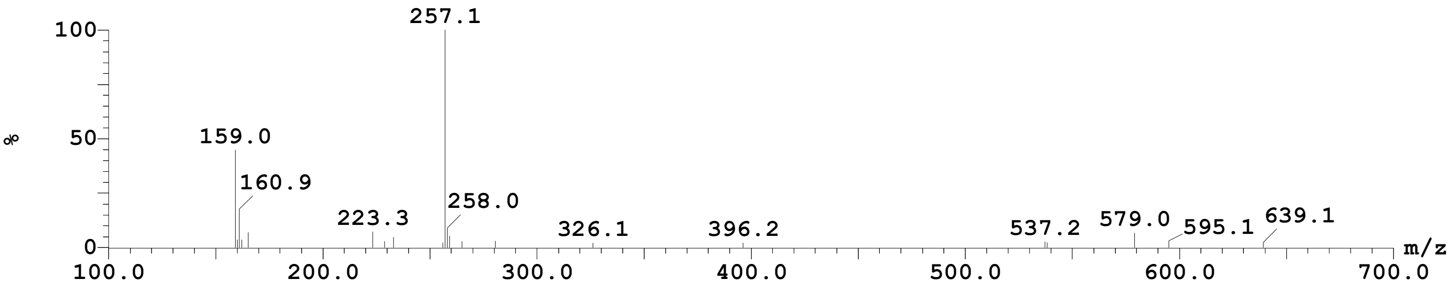

Peak ID **Time**
7 0.65

7:(Time: 0.65) Combine (237:252-(157:165+328:336)) 1:MS ES+ 3.4e+006

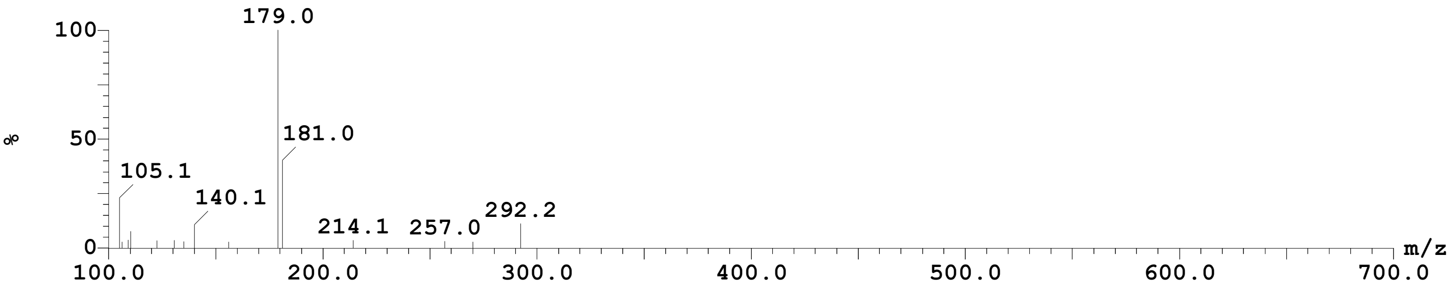

File:13zn480l3 Vial:5:53 ID:B9
Method:C:\MASSLYNX\1minLC_MS.olp

Peak ID **Time**
8 0.77

8: (Time: 0.77) Combine (282:297-(204:211+370:377)) 2:MS ES- 4.2e+004

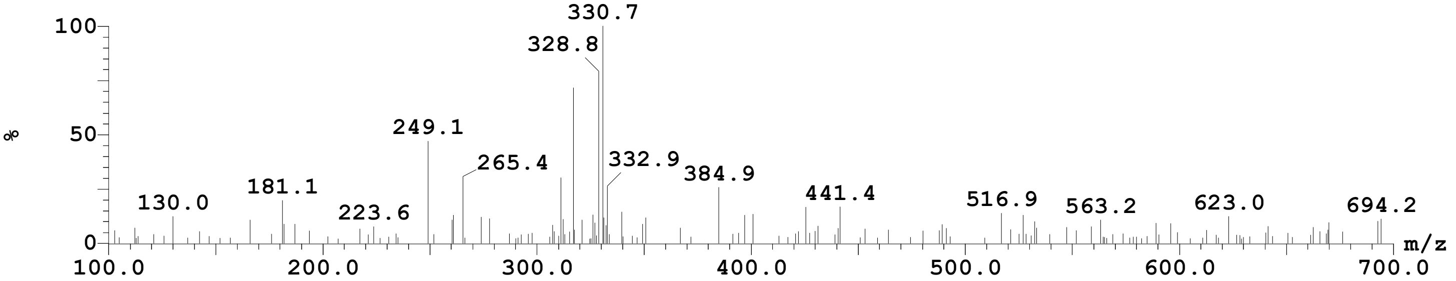

Peak ID **Time**
9 0.99

9: (Time: 0.99) Combine (364:379-288:295) 2:MS ES- 1.4e+005

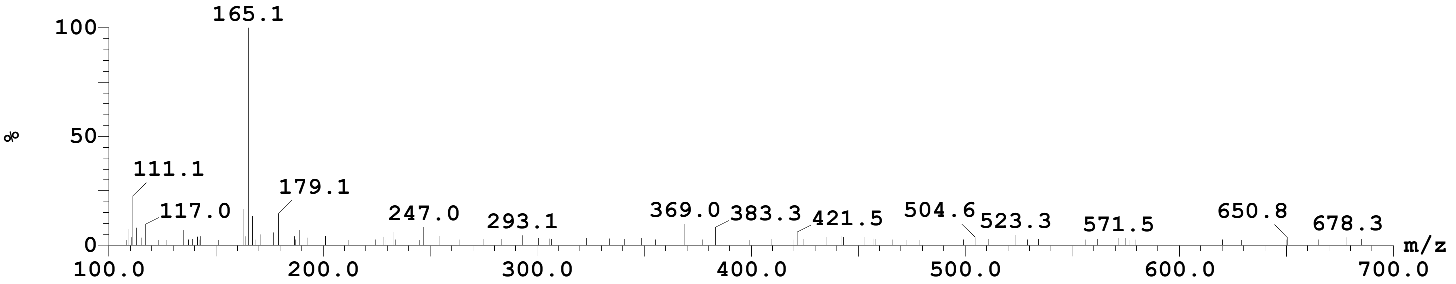

File:13zn320l2 Vial:5:13 ID: **C1**

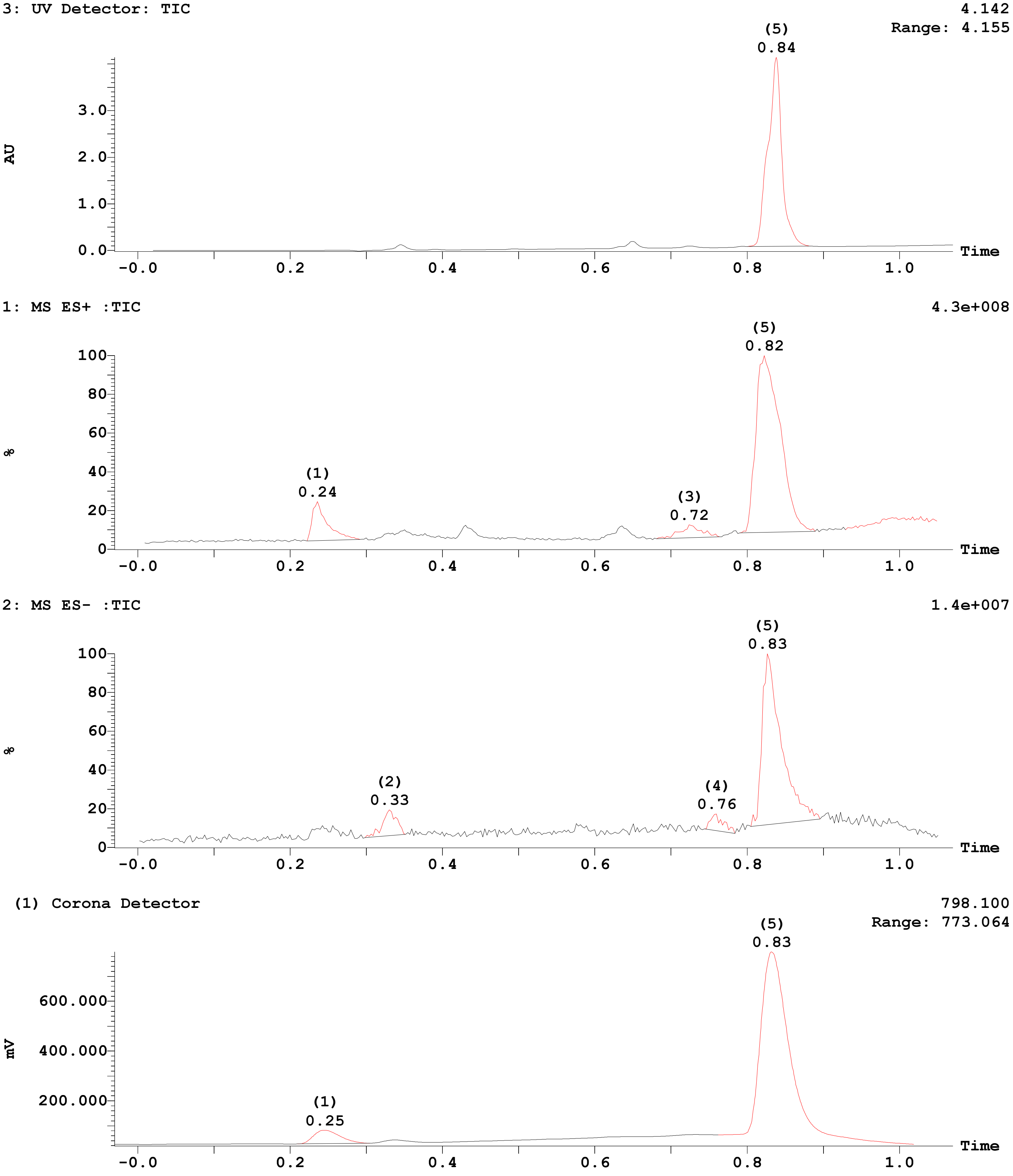

File:13zn320l2 Vial:5:13 ID:C1
Method:C:\MASSLYNX\1minLC_MS.olp

Peak ID	Time
1	0.24

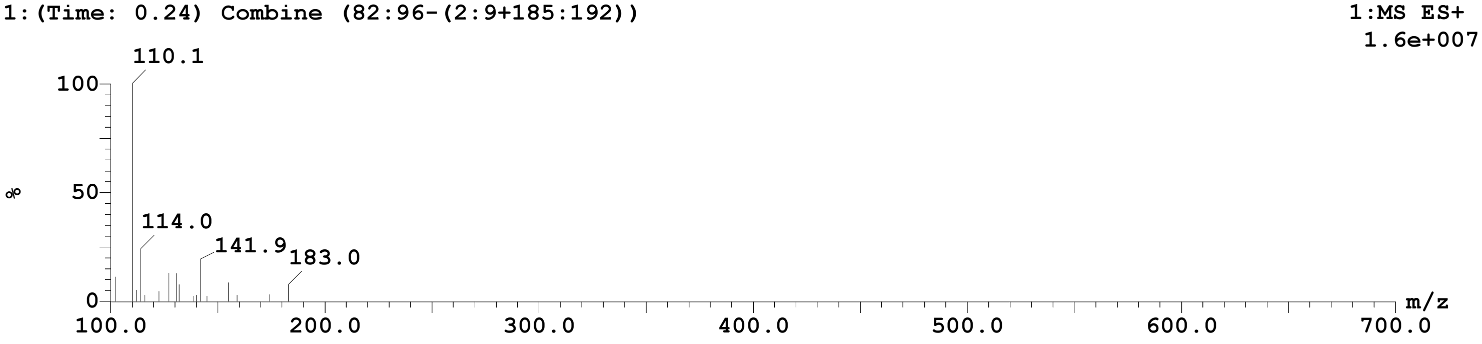

Peak ID	Time
2	0.33

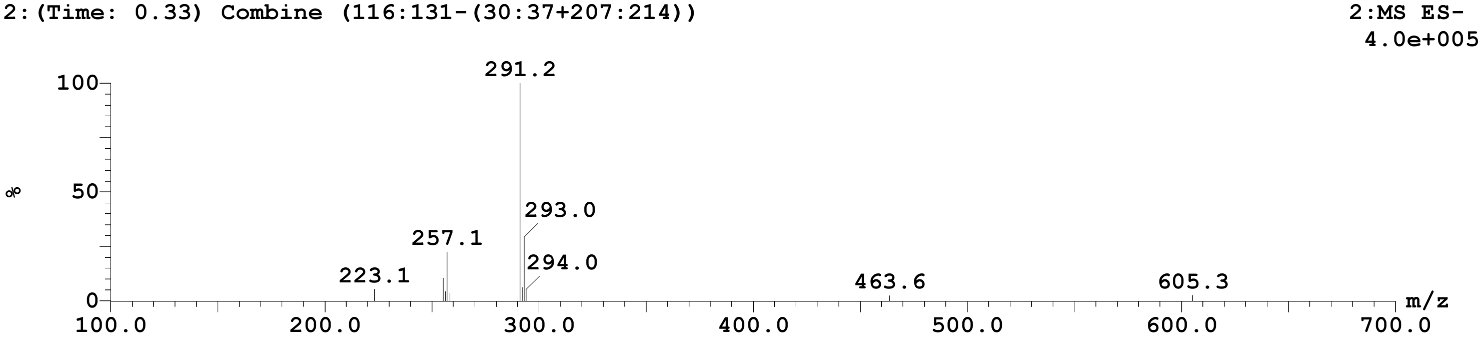

Peak ID	Time
3	0.72

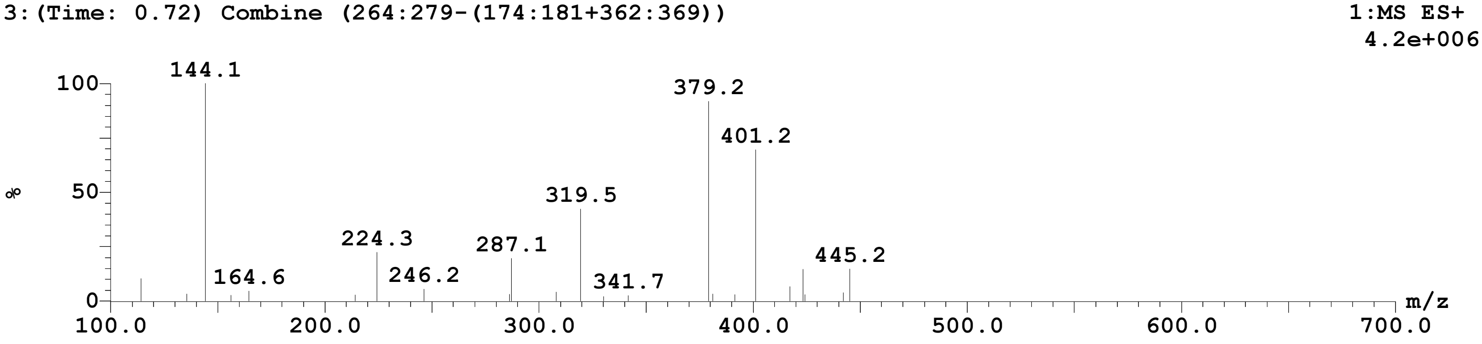

Peak ID	Time
4	0.76

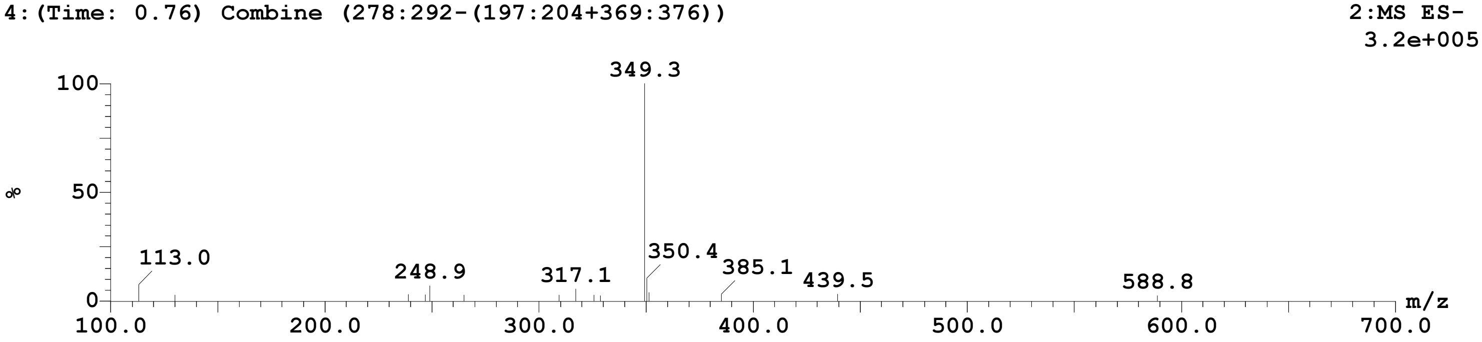

Peak ID	Time
5	0.82

5: (Time: 0.84) Combine (307:322-(218:226+407:413)) 1:MS ES+ 1.8e+008

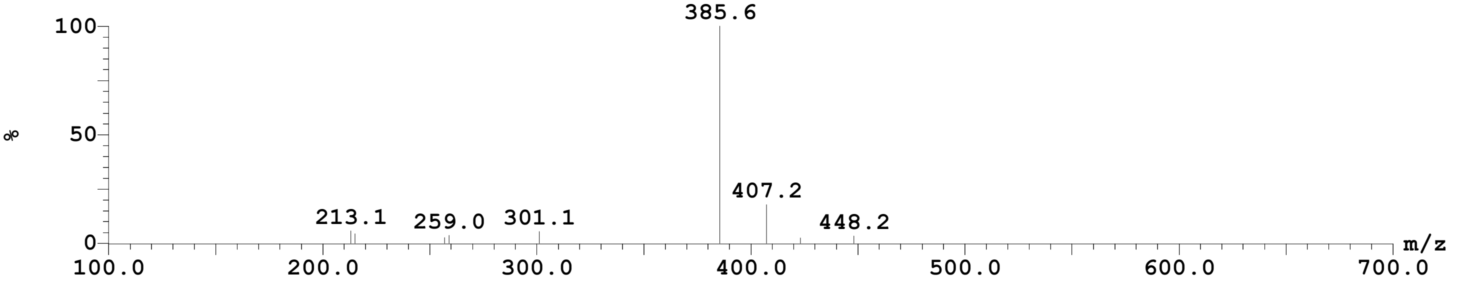

Peak ID	Time
5	0.82

5: (Time: 0.84) Combine (307:322-(218:225+407:413)) 2:MS ES- 2.7e+006

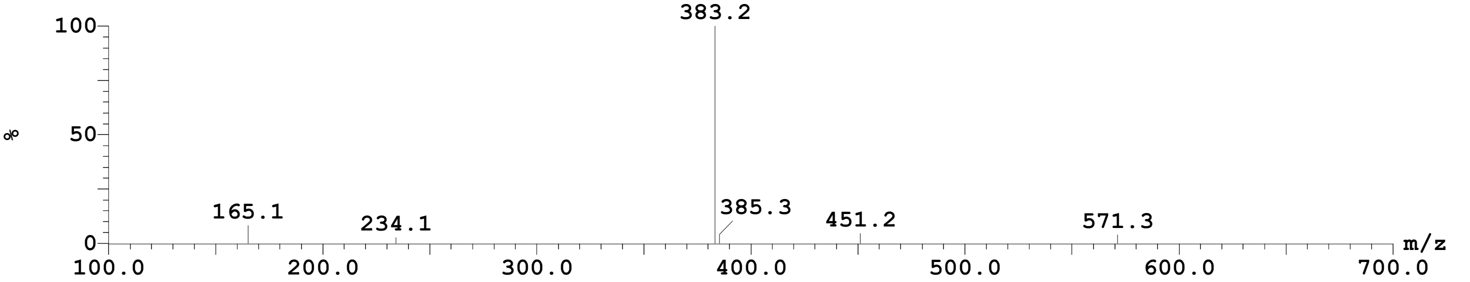

Peak ID	Time
6	1.03

6: (Time: 1.03) Combine (378:393-267:274) 1:MS ES+ 2.5e+007

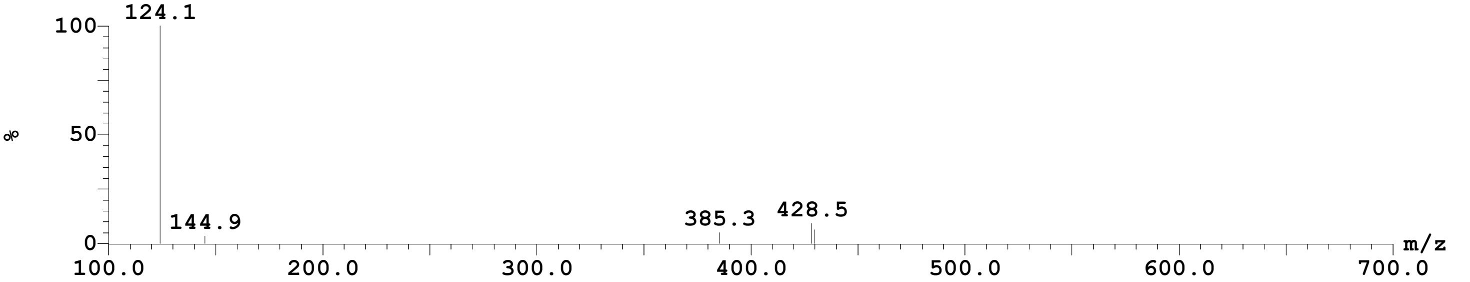

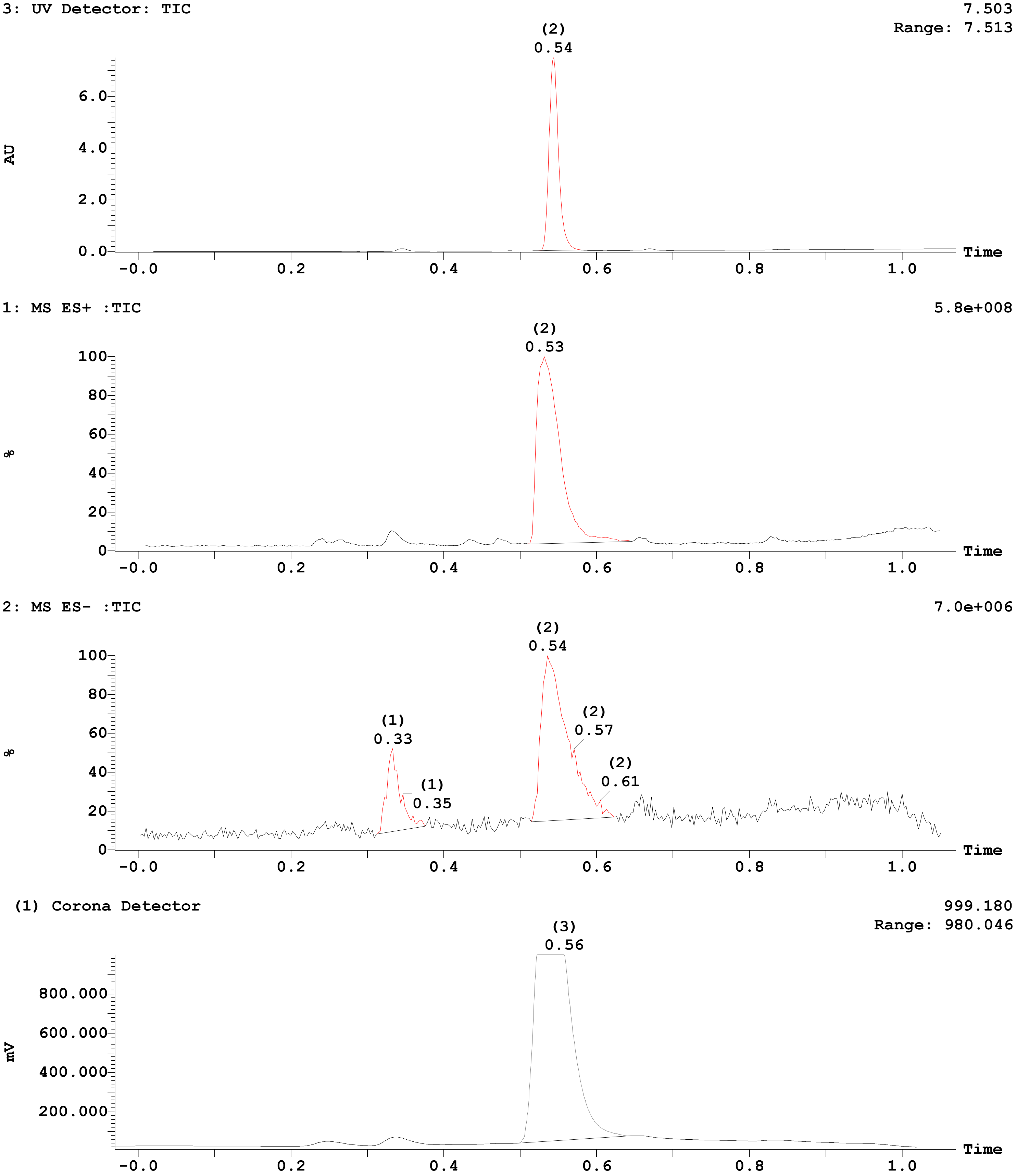
3: UV Detector: TIC
7.503
Range: 7.513
(2)
0.54
AU
6.0
4.0
2.0
0.0
Time
-0.0
0.2
0.4
0.6
0.8
1.0
1: MS ES+ :TIC
5.8e+008
(2)
0.53
%
100
80
60
40
20
0
2: MS ES- :TIC
7.0e+006
(2)
0.54
(1)
0.33
(1)
0.35
(2)
0.57
(2)
0.61
(1) Corona Detector
999.180
Range: 980.046
(3)
0.56
mV
800.000
600.000
400.000
200.000

File:13zo275l2 Vial:5:47 ID:C2
Method:C:\MASSLYNX\1minLC_MS.olp

Peak ID	Time
1	0.33

1:(Time: 0.33) Combine (117:132-(35:42+216:223)) 2:MS ES- 6.2e+005

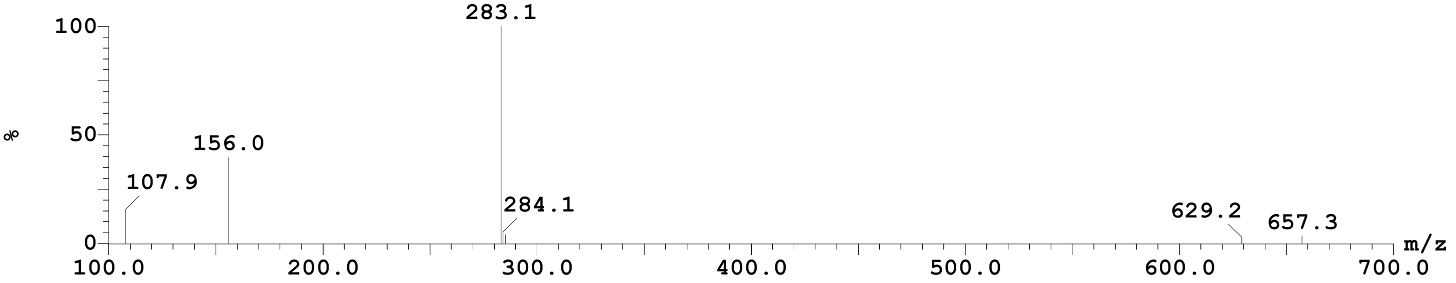

Peak ID	Time
2	0.53

2:(Time: 0.53) Combine (193:207-(110:117+318:325)) 1:MS ES+ 1.3e+008

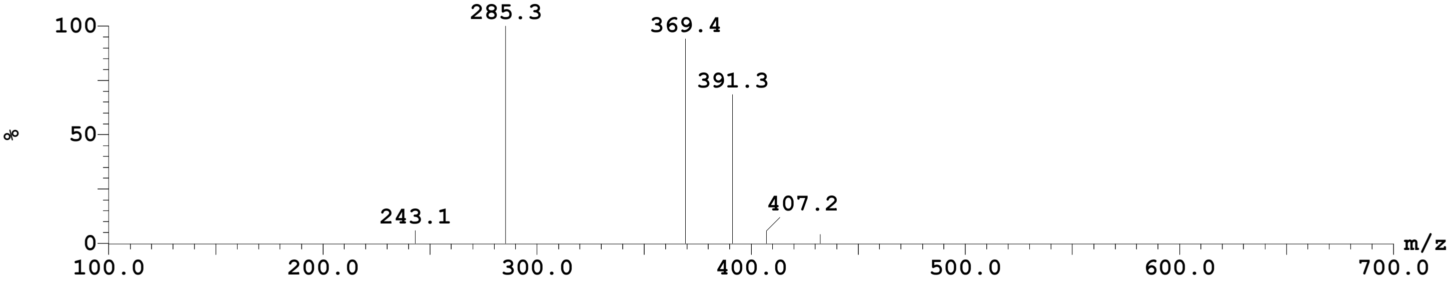

Peak ID	Time
2	0.53

2:(Time: 0.54) Combine (196:211-(114:122+292:300)) 2:MS ES- 5.4e+005

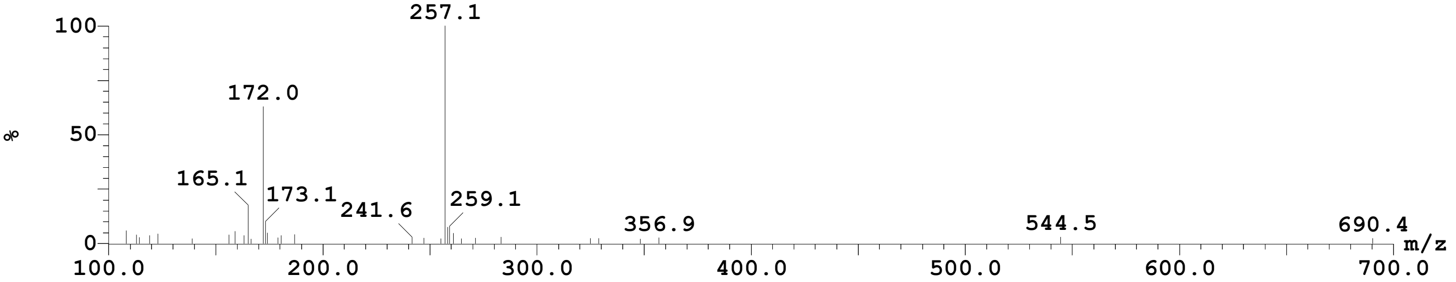

File:13zp883l1 Vial:5:48 ID: **C3**

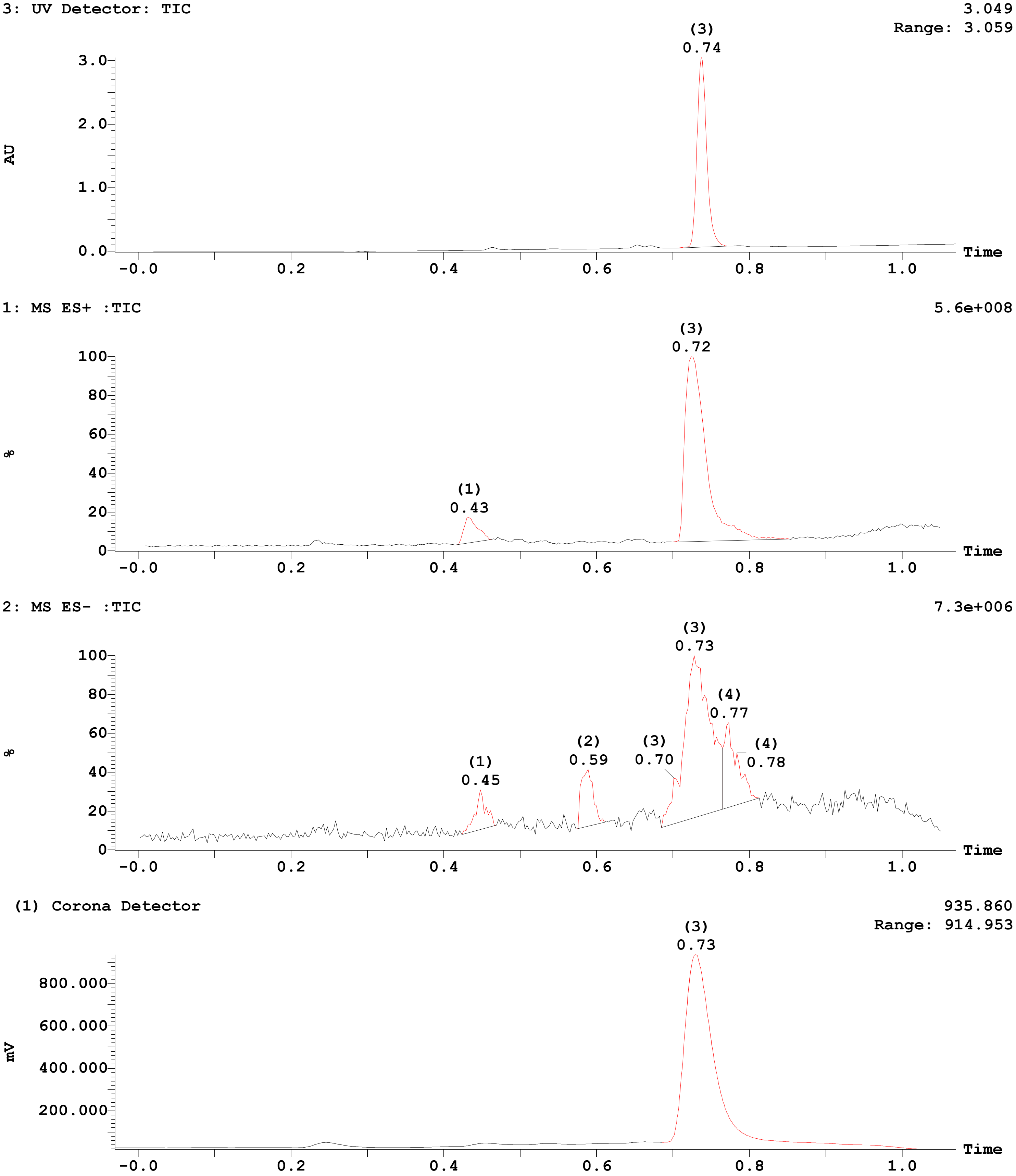

File:13zp883l1 Vial:5:48 ID:C3
Method:C:\MASSLYNX\1minLC_MS.olp

Peak ID **Time**
1 0.43

1:(Time: 0.43) Combine (155:170-(75:82+249:256)) 1:MS ES+ 1.5e+007

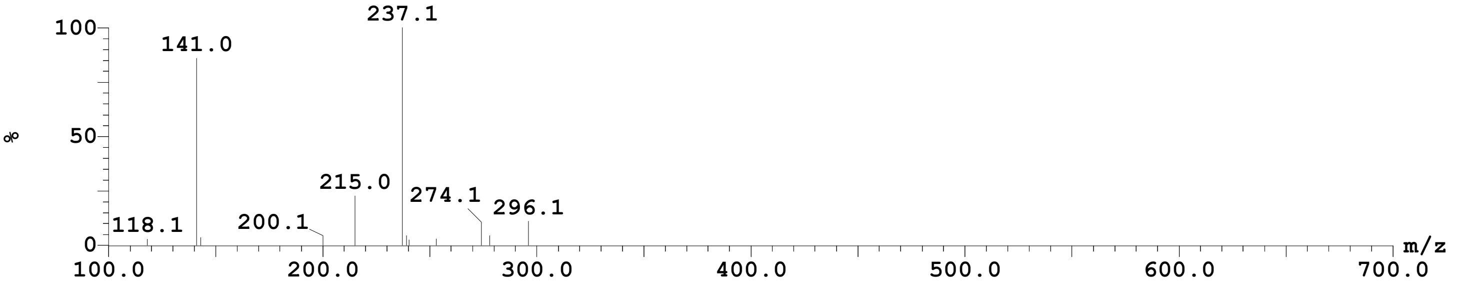

Peak ID **Time**
1 0.43

1:(Time: 0.45) Combine (160:175-(77:84+251:258)) 2:MS ES- 1.5e+005

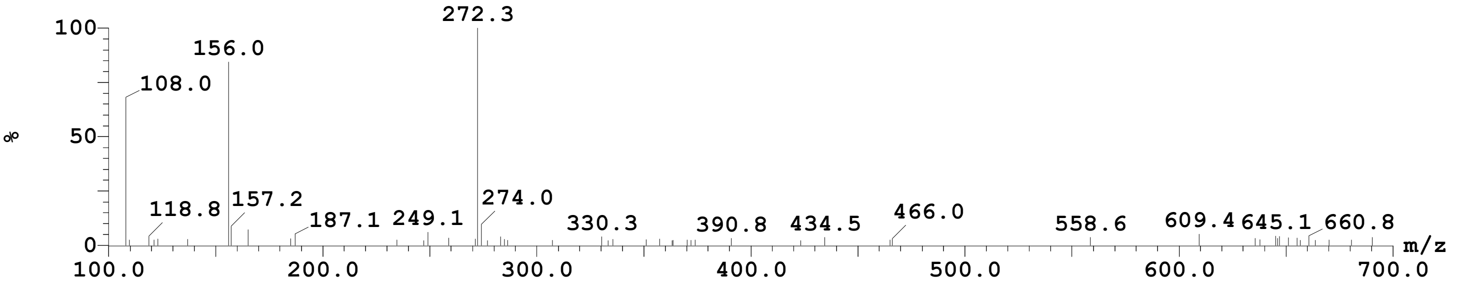

Peak ID **Time**
2 0.59

2:(Time: 0.59) Combine (213:228-(133:140+304:311)) 2:MS ES- 5.1e+005

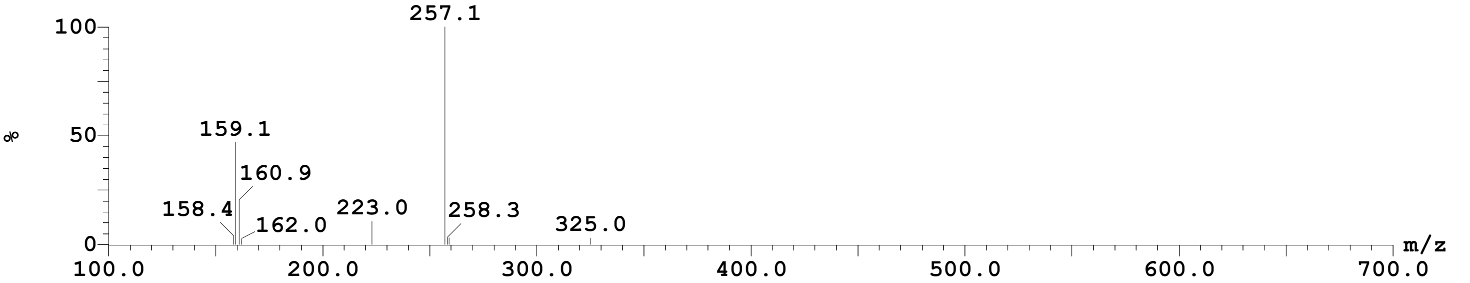

Peak ID **Time**
3 0.72

3:(Time: 0.74) Combine (270:285-(182:190+365:372)) 1:MS ES+ 9.3e+007

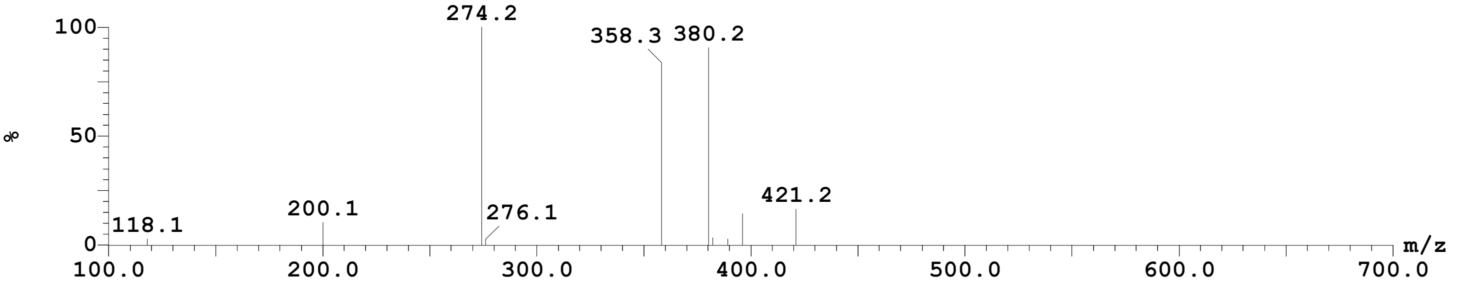

Peak ID **Time**
3 0.72

3:(Time: 0.74) Combine (269:284-(182:189+364:372)) 2:MS ES- 2.9e+005

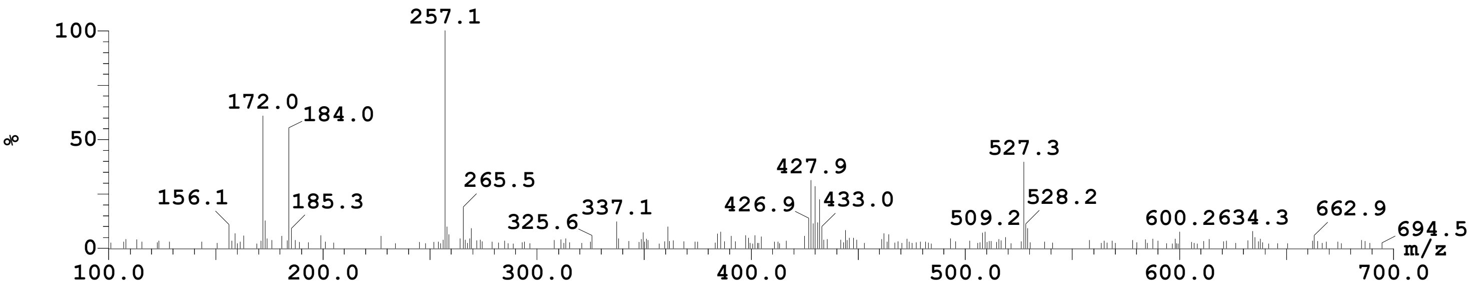

Peak ID **Time**
4 0.77

4:(Time: 0.77) Combine (283:297-(205:212+380:387)) 2:MS ES- 2.5e+005

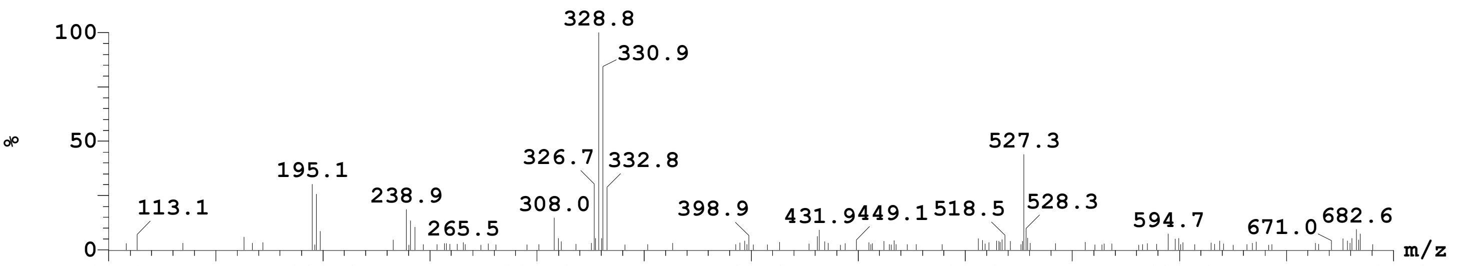

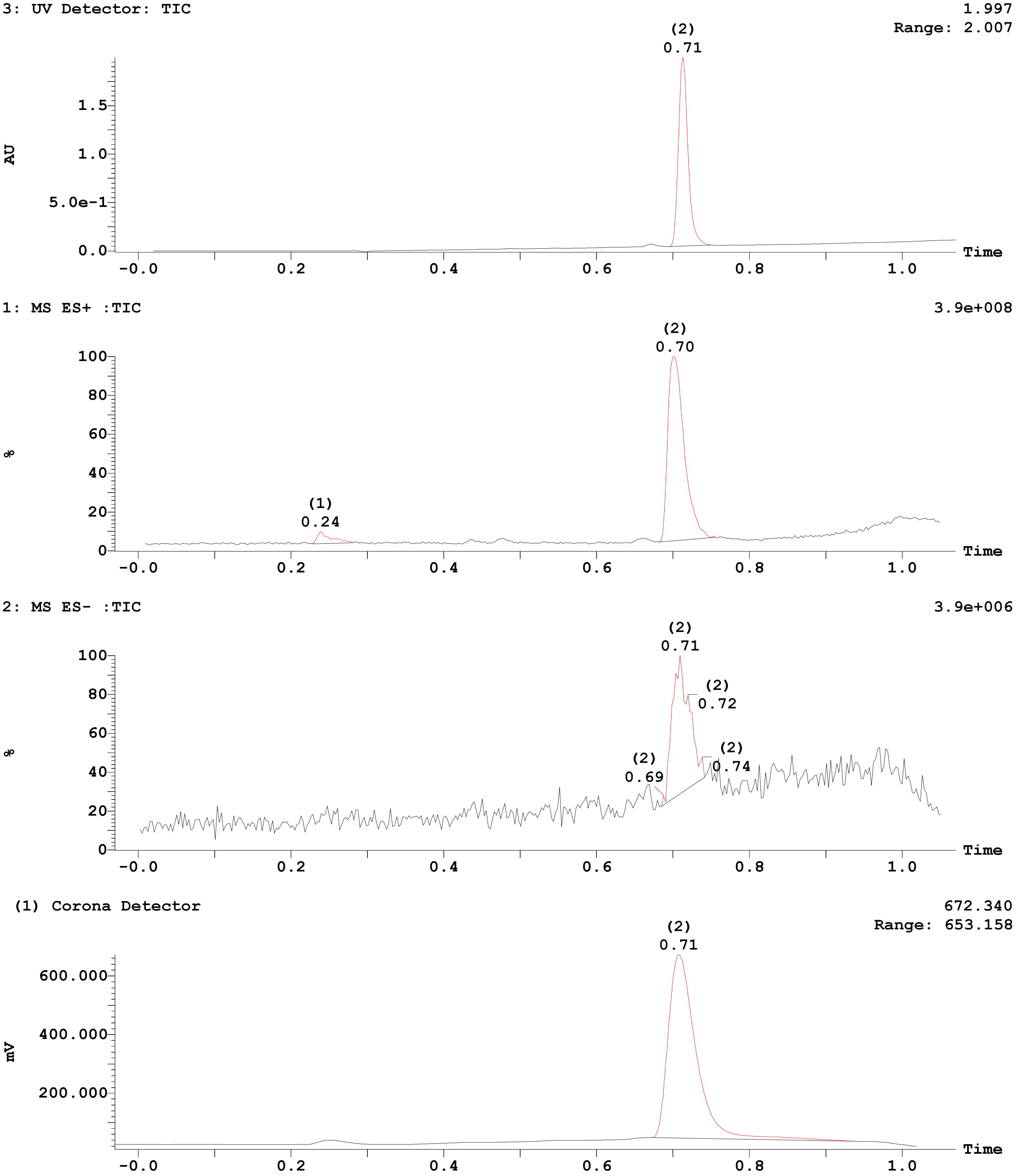
3: UV Detector: TIC
1.997
Range: 2.007
(2)
0.71
AU
Time
1: MS ES+ :TIC
3.9e+008
(1)
0.24
(2)
0.70
%
Time
2: MS ES- :TIC
3.9e+006
(2)
0.71
(2)
0.72
(2)
0.69
(2)
0.74
%
Time
(1) Corona Detector
672.340
Range: 653.158
(2)
0.71
mV
Time

File:13zn229l6 Vial:5:49 ID:C4
Method:C:\MASSLYNX\1minLC_MS.olp

Peak ID **Time**
1 0.24

1:(Time: 0.24) Combine (83:97-(4:11+181:188)) 1:MS ES+ 3.7e+006

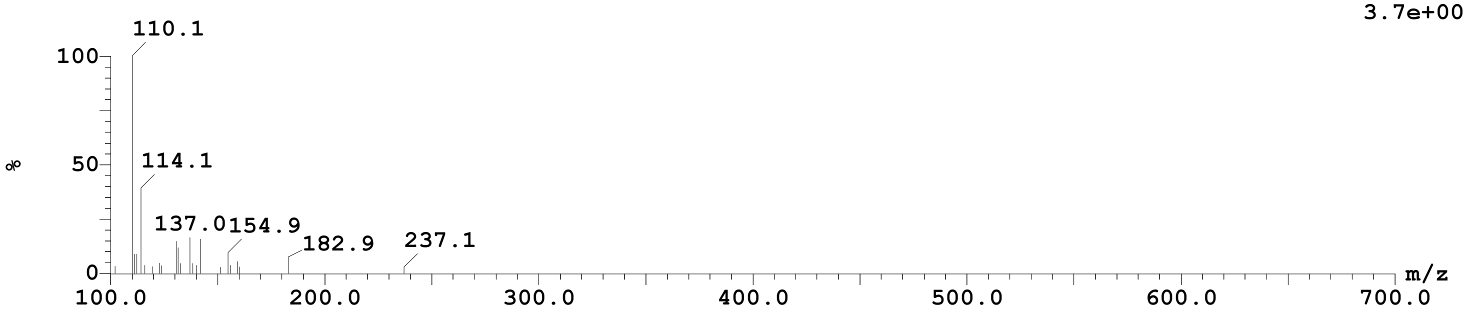

Peak ID **Time**
2 0.70

2:(Time: 0.71) Combine (260:275-(178:186+357:364)) 1:MS ES+ 7.3e+007

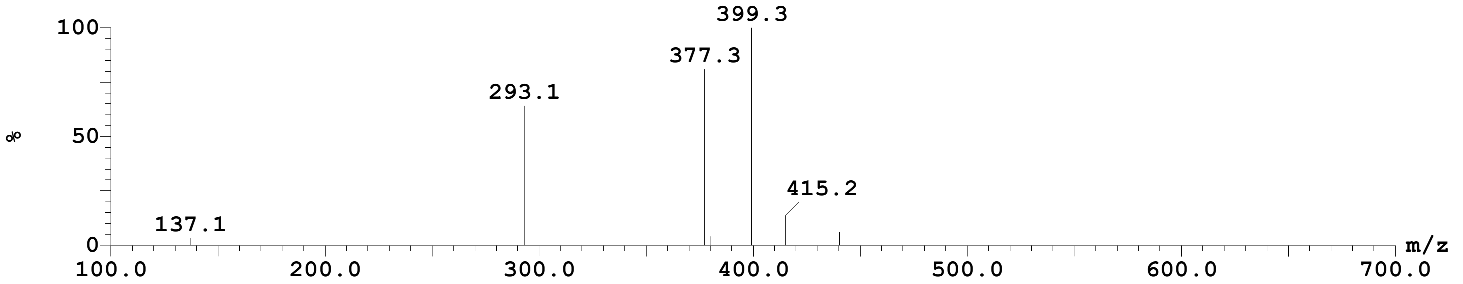

Peak ID **Time**
2 0.70

2:(Time: 0.71) Combine (259:274-(175:182+353:360)) 2:MS ES- 1.1e+005

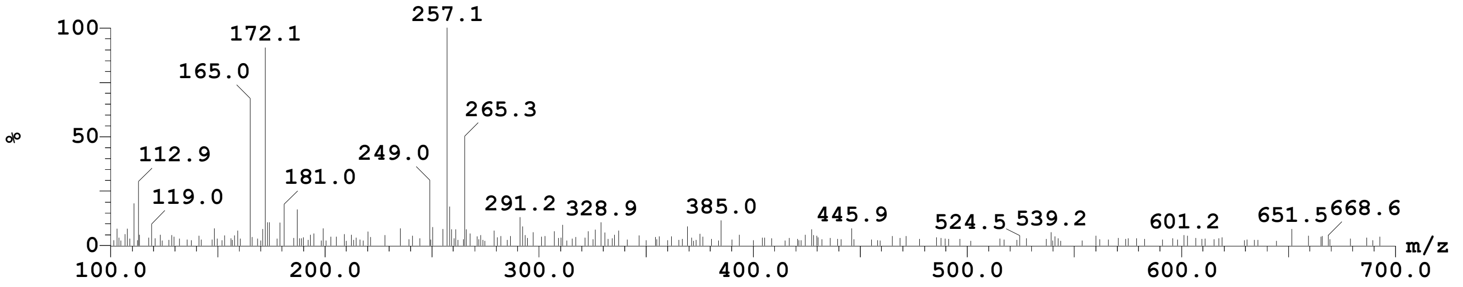

File:13zp903l1 Vial:5:46 ID: **C5**

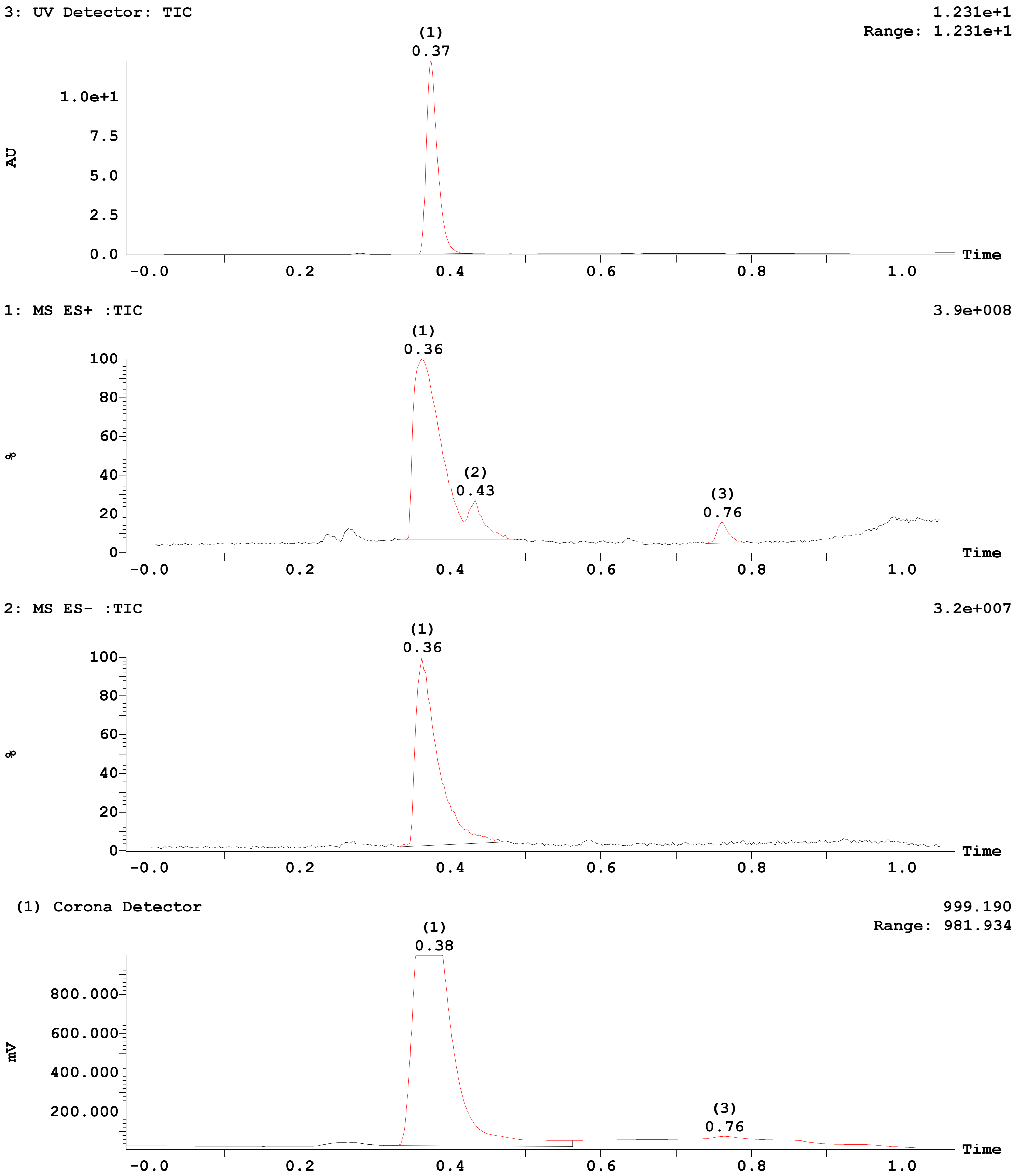

File:13zp903l1 Vial:5:46 ID:C5
Method:C:\MASSLYNX\1minLC_MS.olp

Peak ID **Time**
1 0.36

1:(Time: 0.37) Combine (133:148-(52:59+233:241)) 1:MS ES+
2.4e+008

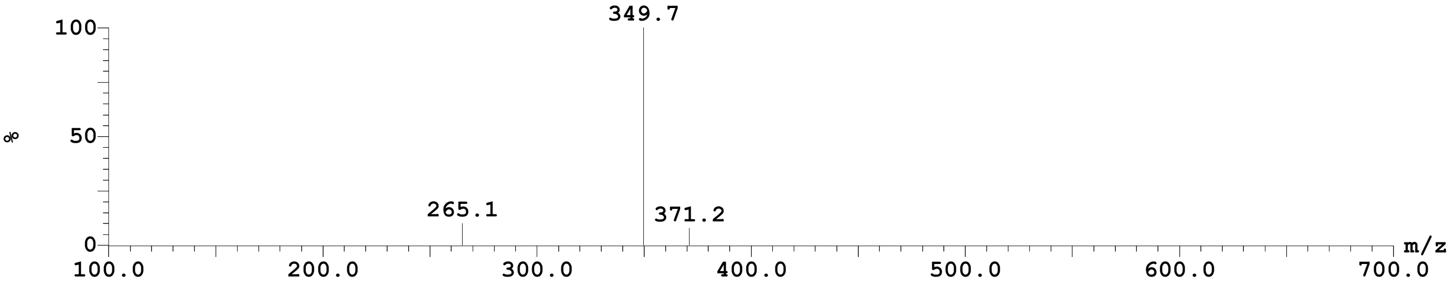

Peak ID **Time**
1 0.36

1:(Time: 0.36) Combine (128:143-(43:50+252:259)) 2:MS ES-
1.1e+007

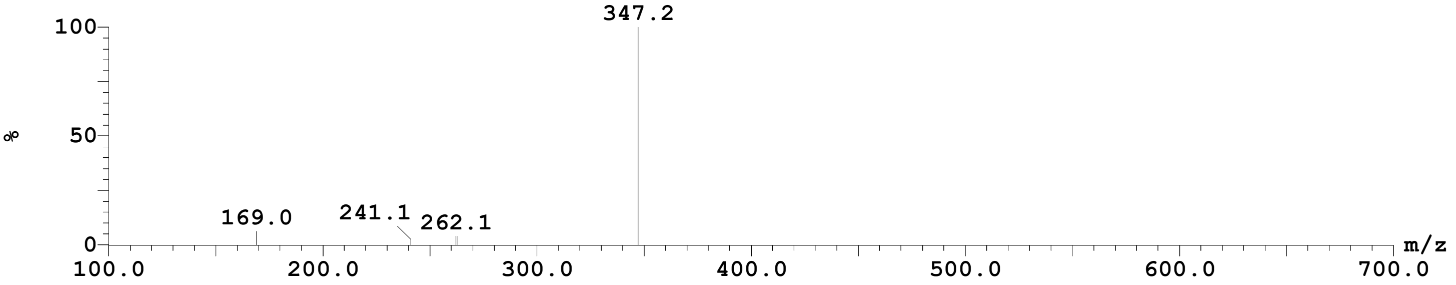

Peak ID **Time**
2 0.43

2:(Time: 0.43) Combine (156:171-(76:83+258:265)) 1:MS ES+
2.3e+007

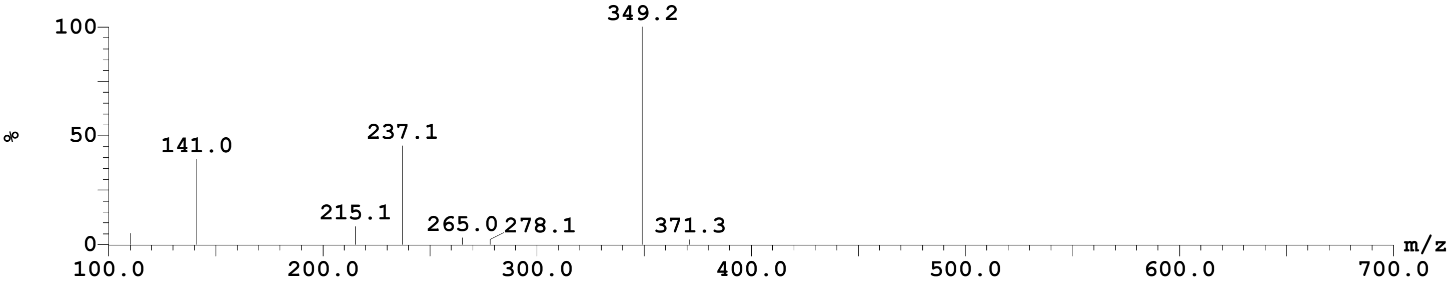

Peak ID **Time**
3 0.76

3:(Time: 0.76) Combine (279:294-(196:203+372:379)) 1:MS ES+
8.3e+006

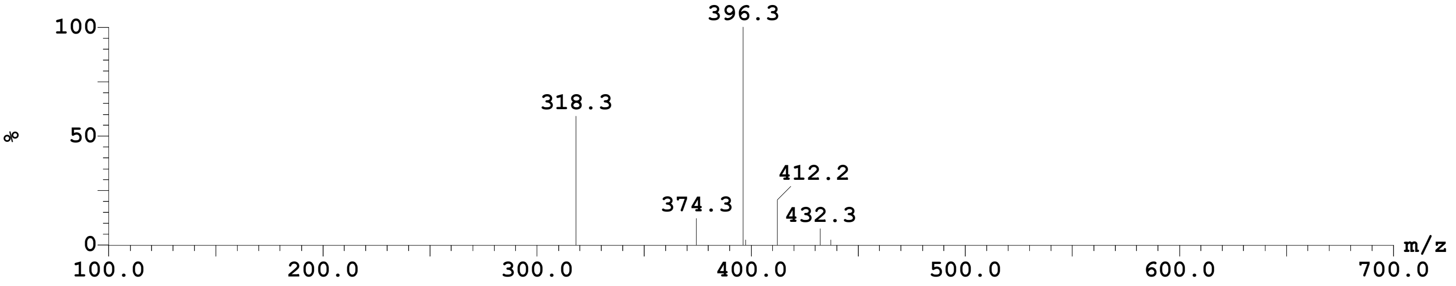

SYNGENTA SQD OPEN ACCESS LC-MS

File:13zp904l1 Vial:5:47 ID: **C6**

3: UV Detector: TIC 6.087

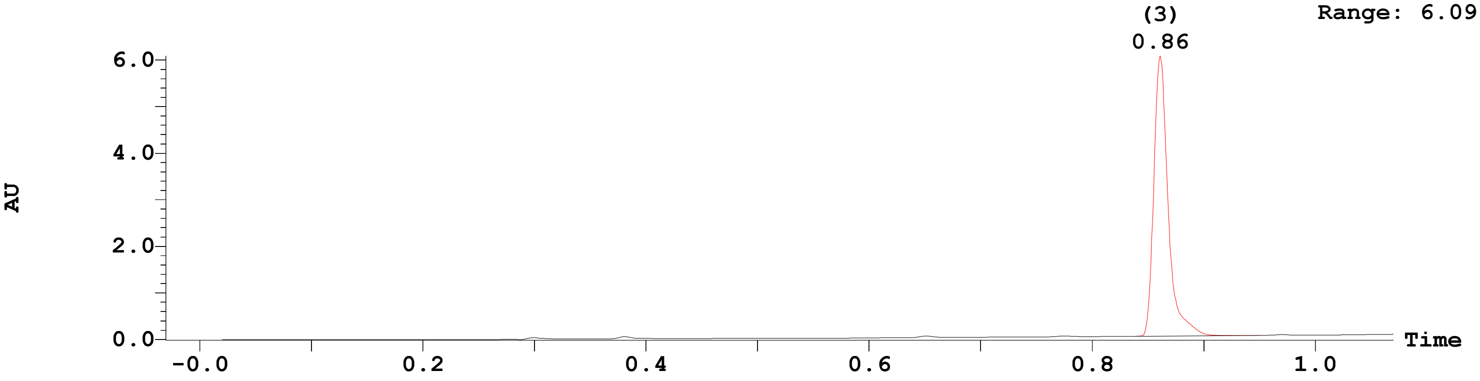

1: MS ES+ :TIC 5.4e+008

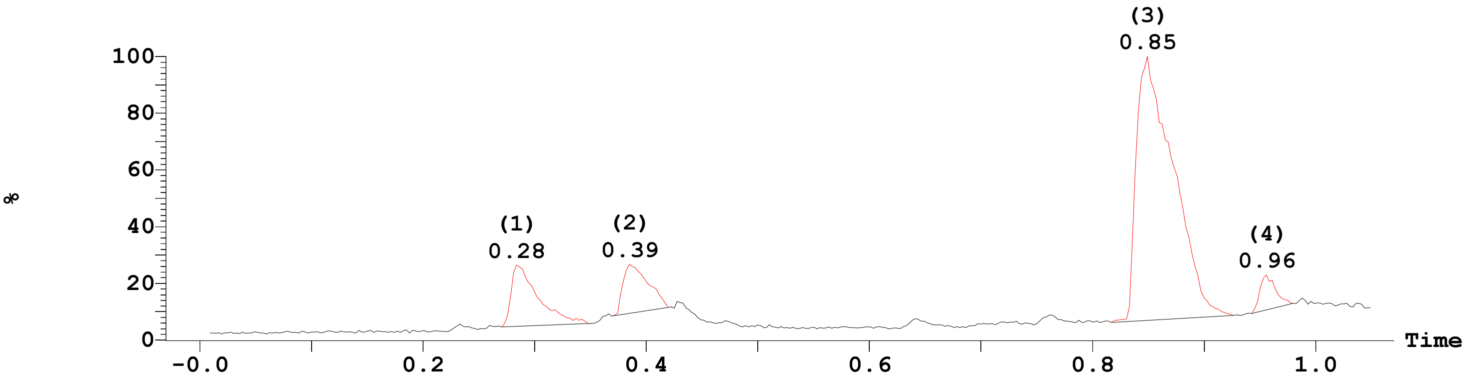

2: MS ES- :TIC 4.0e+007

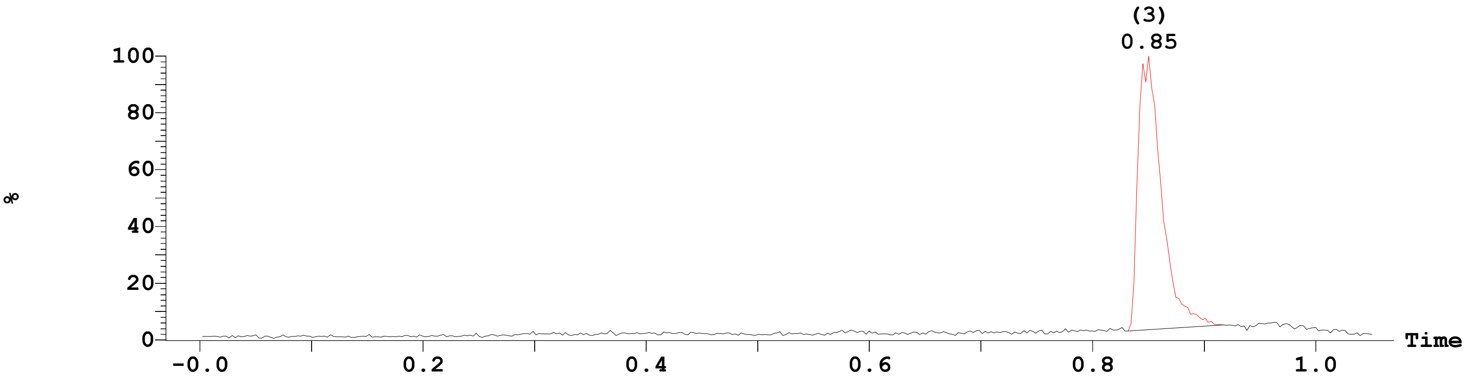

(1) Corona Detector 999.170

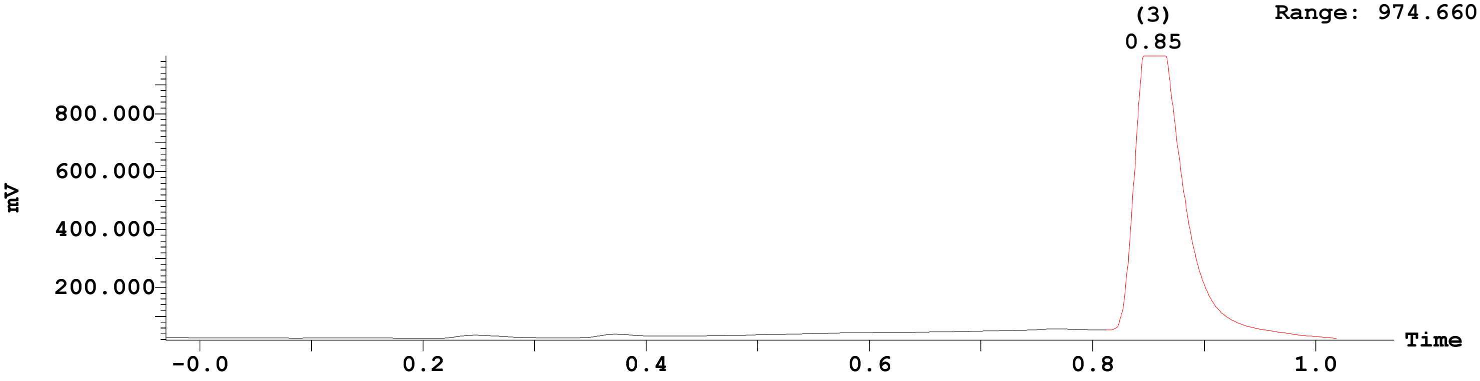

File:13zp904l1 Vial:5:47 ID:C6
Method:C:\MASSLYNX\1minLC_MS.olp

Peak ID **Time**
1 0.28

1:(Time: 0.28) Combine (99:115-(20:27+207:214)) 1:MS ES+ 4.3e+007

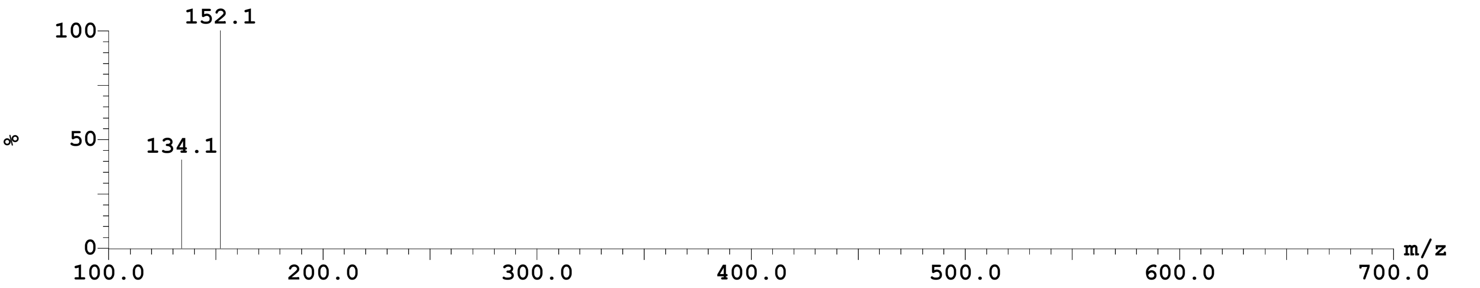

Peak ID **Time**
2 0.39

2:(Time: 0.39) Combine (138:153-(57:64+233:240)) 1:MS ES+ 6.9e+007

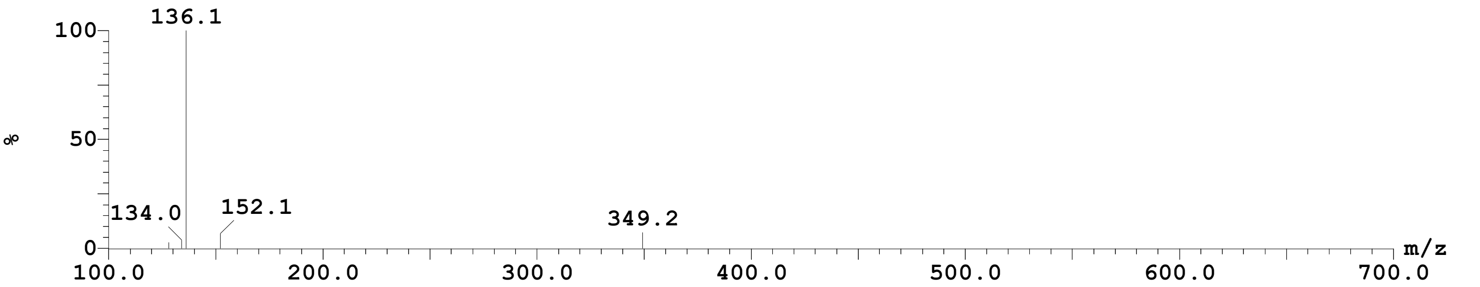

Peak ID **Time**
3 0.85

3:(Time: 0.86) Combine (316:331-232:240) 1:MS ES+ 1.4e+008

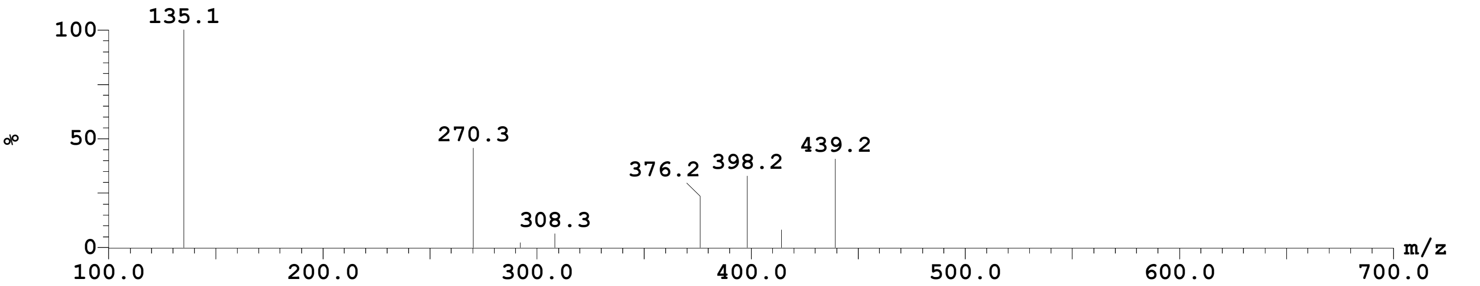

Peak ID **Time**
3 0.85

3:(Time: 0.85) Combine (312:327-230:237) 2:MS ES- 1.6e+007

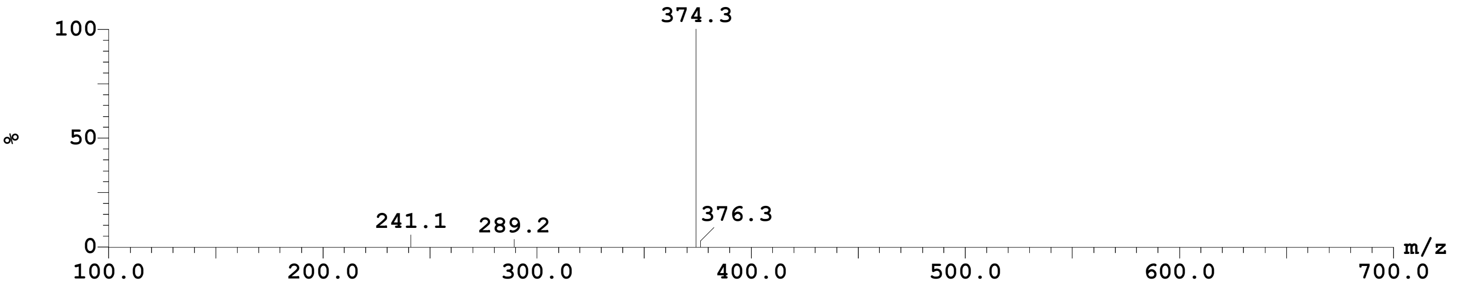

File:13zp904I1 Vial:5:47 ID:C6
Method:C:\MASSLYNX\1minLC_MS.olp

Peak ID **Time**
4 0.96

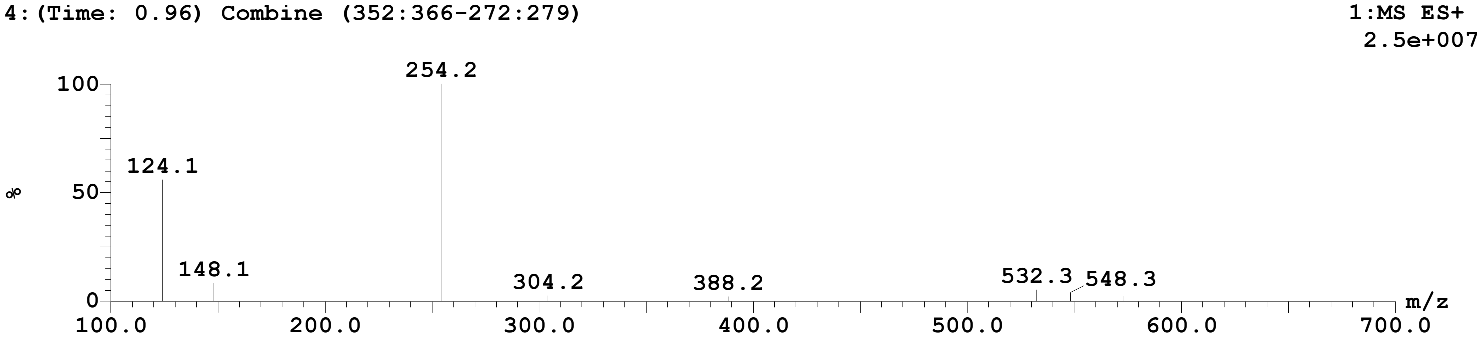

SYNGENTA SQD OPEN ACCESS LC-MS Page 1
File:13zn228l2 Vial:5:14 ID: **C7**

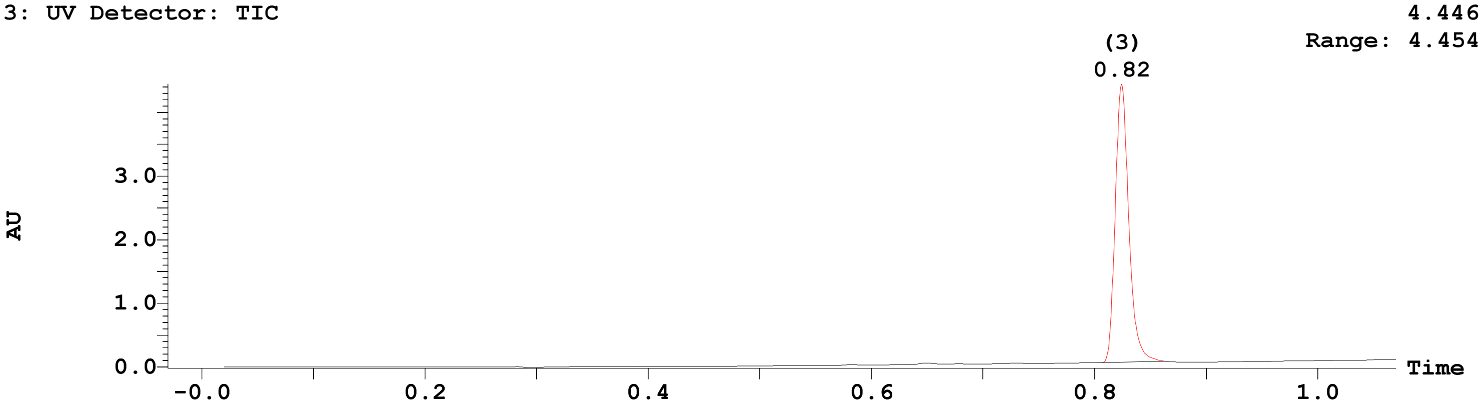

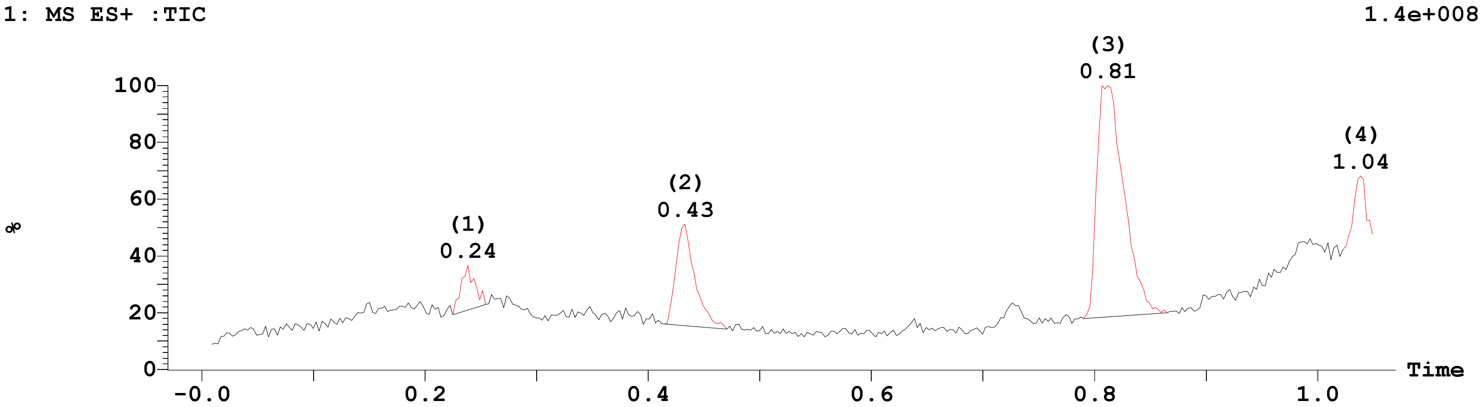

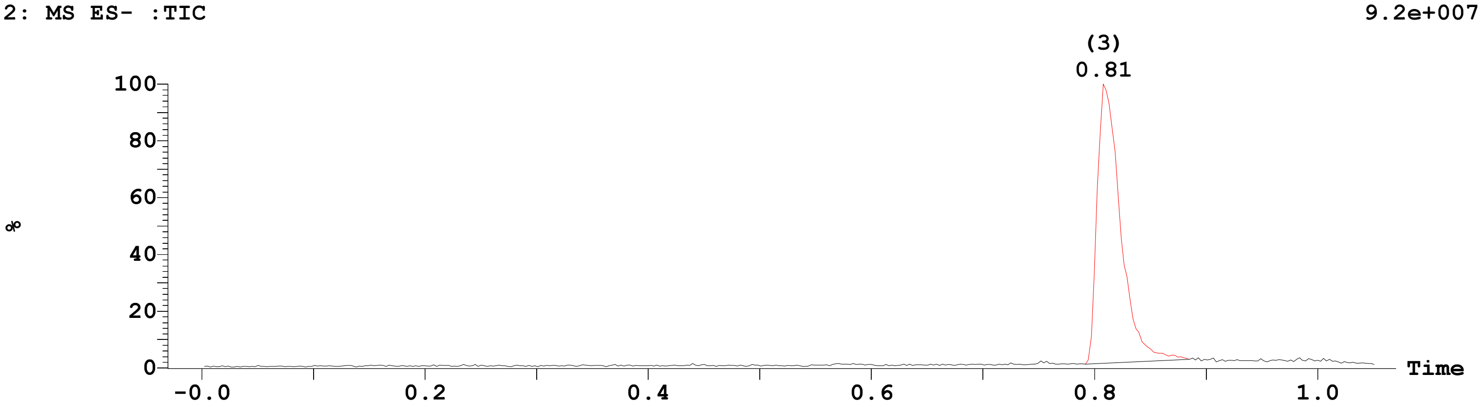

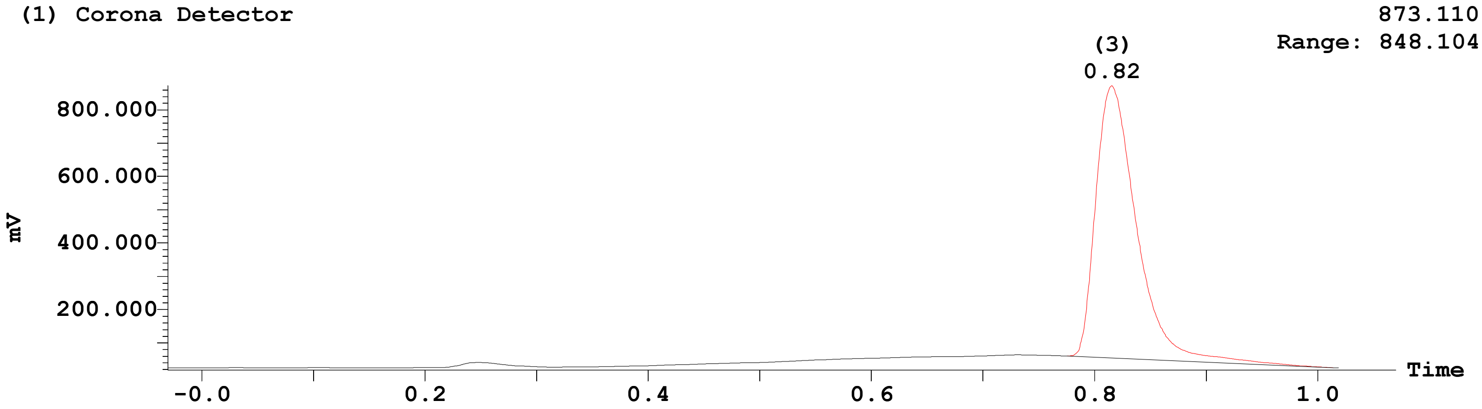

File:13zn228l2 Vial:5:14 ID:C7
Method:C:\MASSLYNX\1minLC_MS.olp

Peak ID	Time
1	0.24

1:(Time: 0.24) Combine (83:97-(3:10+171:178)) 1:MS ES+ 7.7e+006

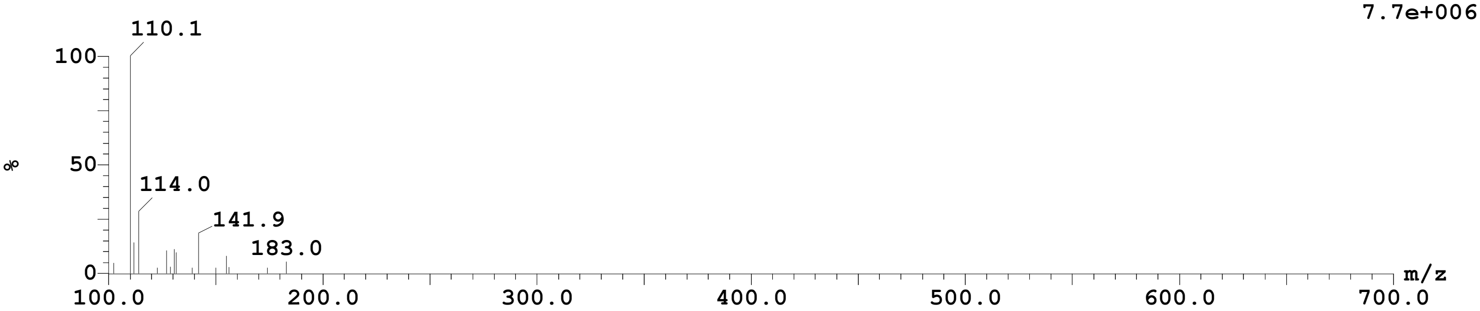

Peak ID	Time
2	0.43

2:(Time: 0.43) Combine (155:170-(74:81+252:259)) 1:MS ES+ 1.0e+007

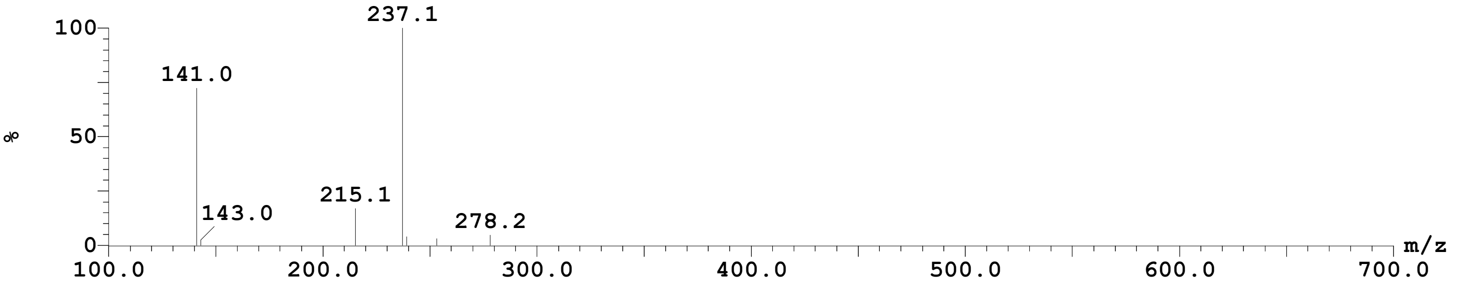

Peak ID	Time
3	0.81

3:(Time: 0.82) Combine (302:317-(218:226+399:407)) 1:MS ES+ 3.4e+007

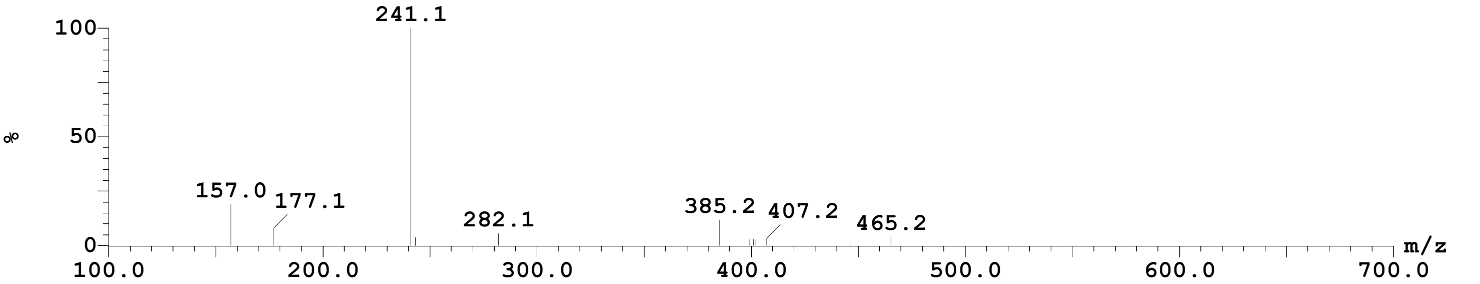

Peak ID	Time
3	0.81

3:(Time: 0.81) Combine (296:310-(215:222+407:413)) 2:MS ES- 4.2e+007

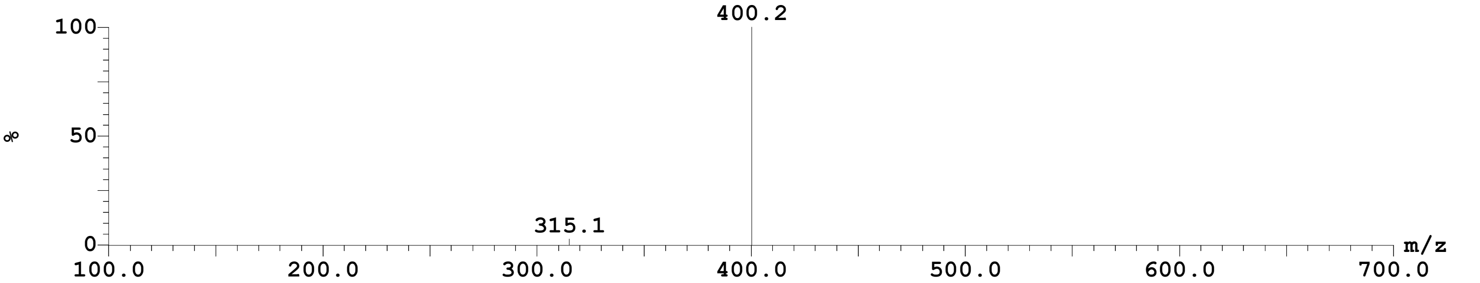

File:13zn228l2 Vial:5:14 ID:C7
Method:C:\MASSLYNX\1minLC_MS.olp

Peak ID **Time**
4 1.04

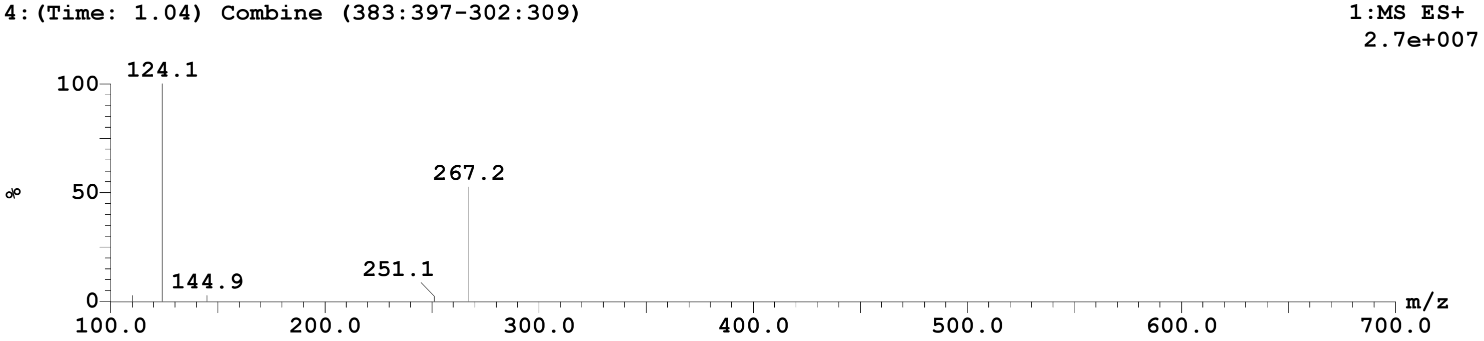

SYNGENTA SQD OPEN ACCESS LC-MS

Page 1

File:13zp679l2 Vial:5:46 ID: **C8**

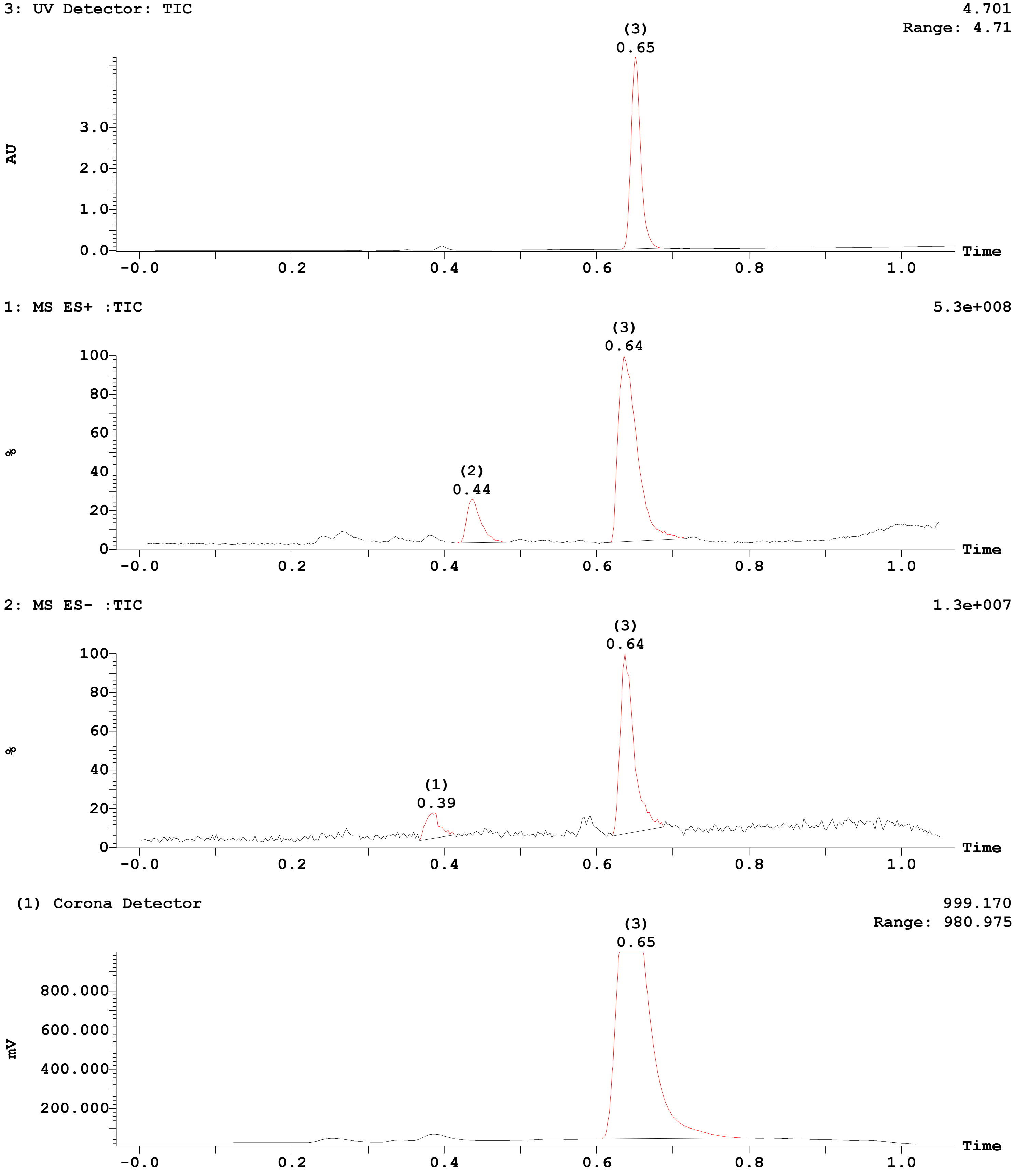

File:13zp679l2 Vial:5:46 ID:C8
Method:C:\MASSLYNX\1minLC_MS.olp

Peak ID **Time**
1 0.39

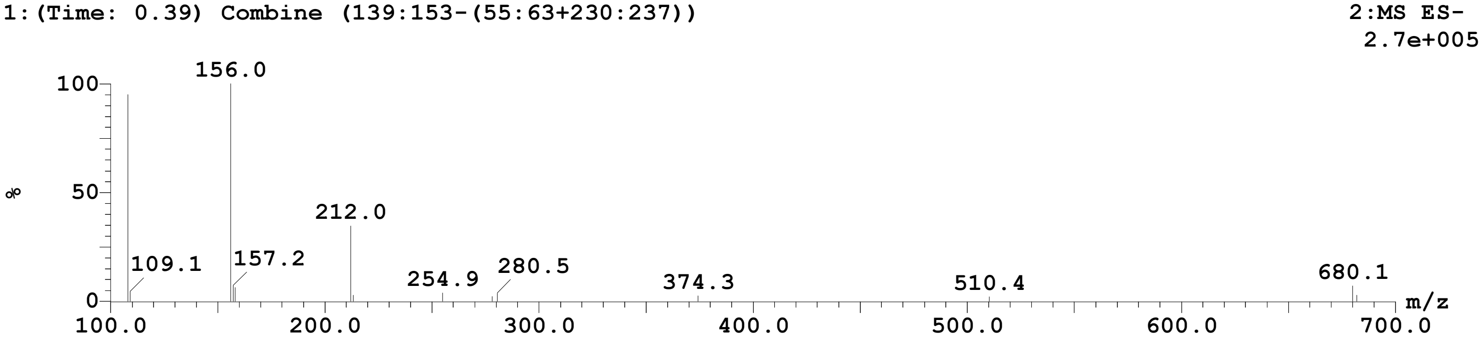

Peak ID **Time**
2 0.44

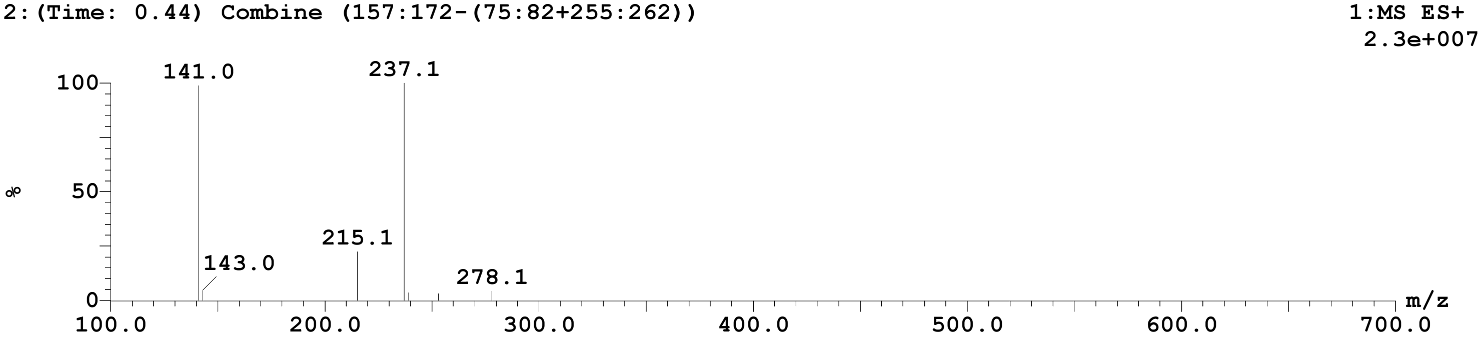

Peak ID **Time**
3 0.64

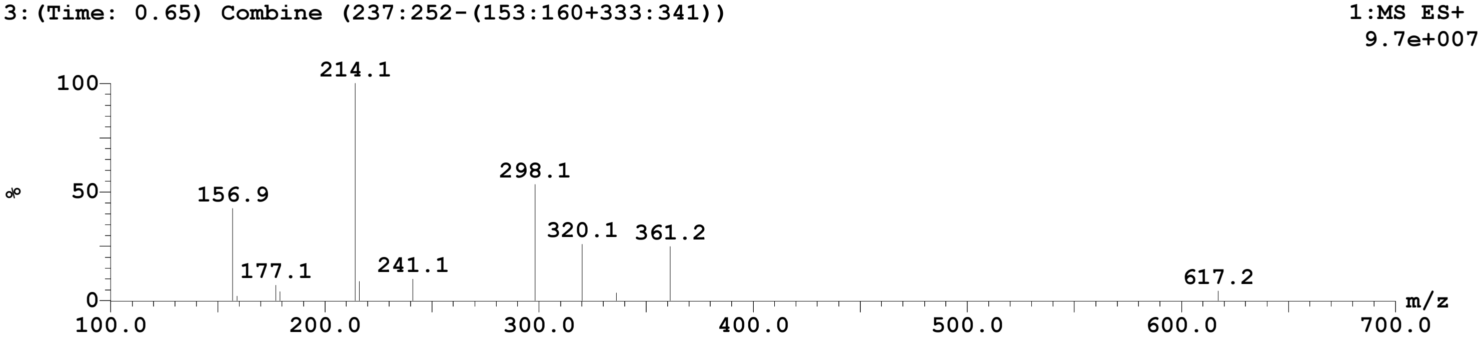

Peak ID **Time**
3 0.64

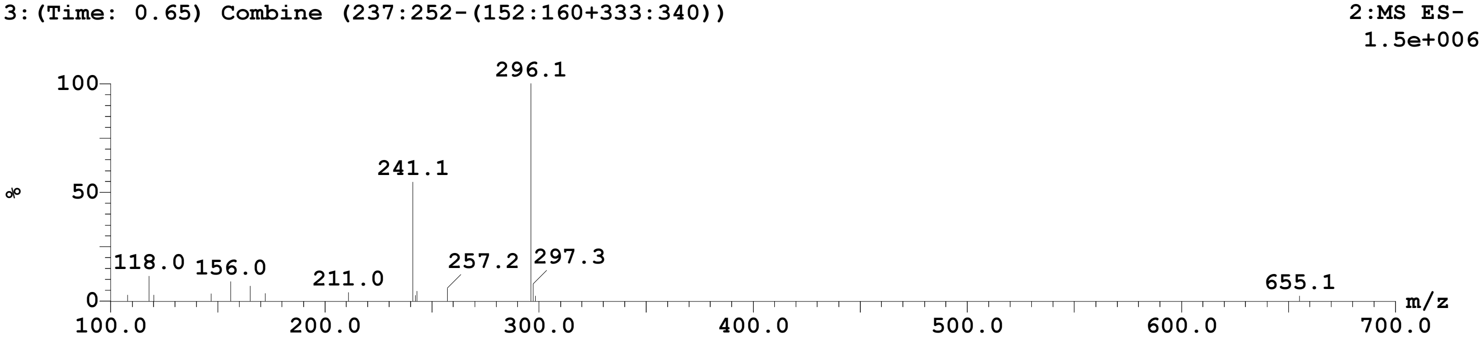

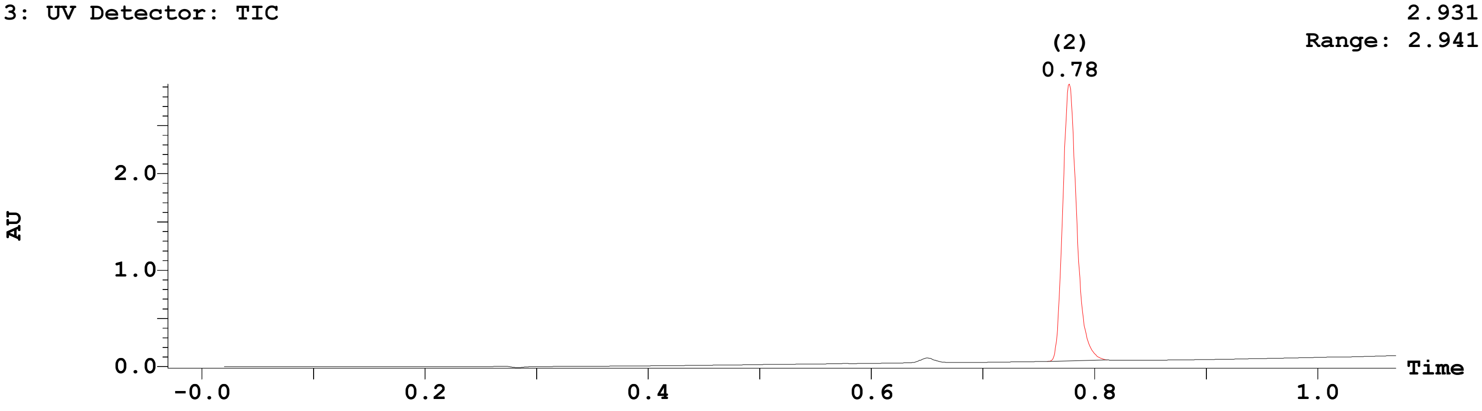
3: UV Detector: TIC
2.931
Range: 2.941
(2)
0.78
AU
2.0
1.0
0.0
-0.0
0.2
0.4
0.6
0.8
1.0
Time

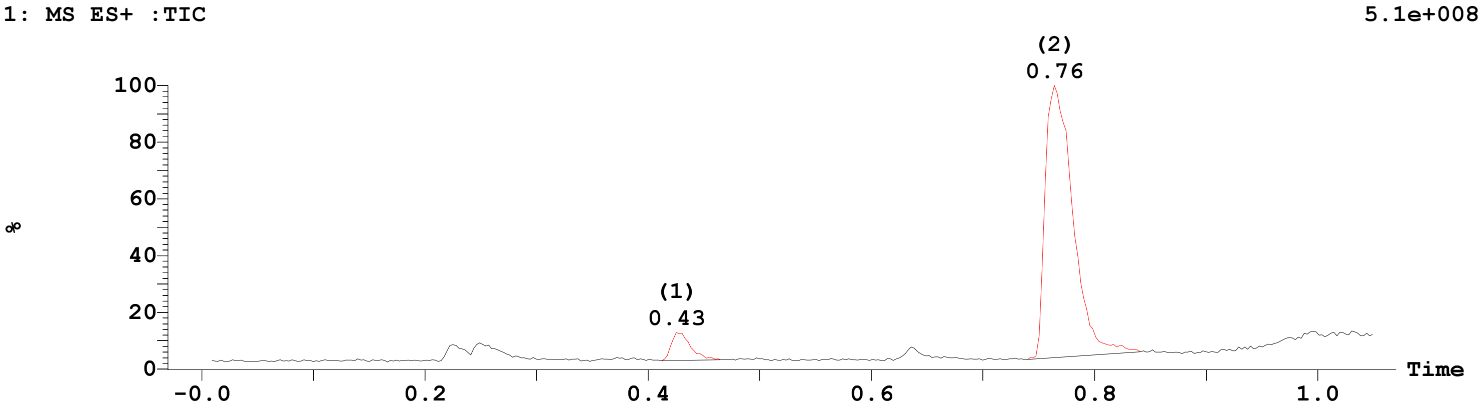
1: MS ES+ :TIC
5.1e+008
(1)
0.43
(2)
0.76
%
100
80
60
40
20
0
-0.0
0.2
0.4
0.6
0.8
1.0
Time

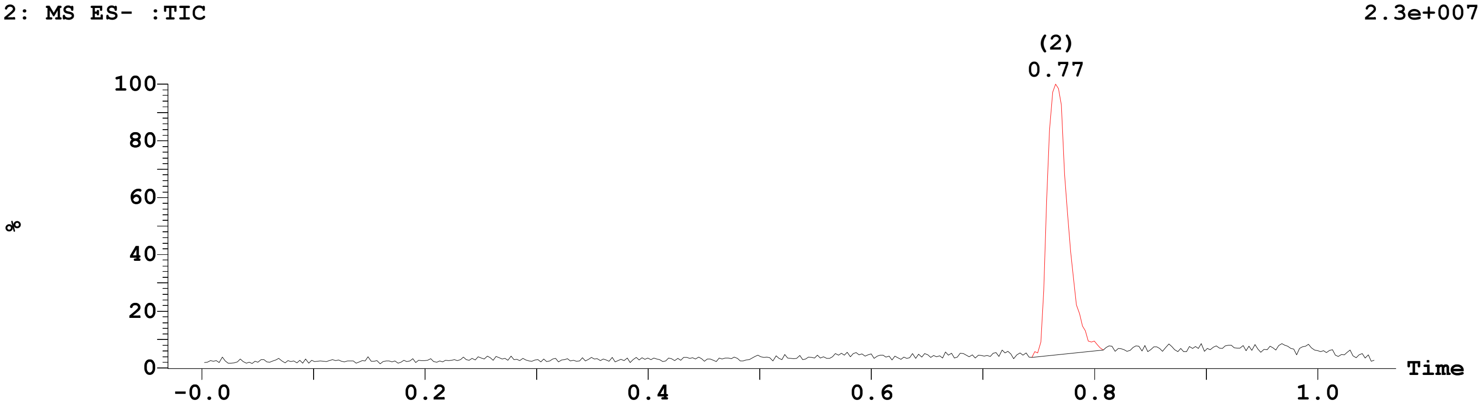
2: MS ES- :TIC
2.3e+007
(2)
0.77
%
100
80
60
40
20
0
-0.0
0.2
0.4
0.6
0.8
1.0
Time

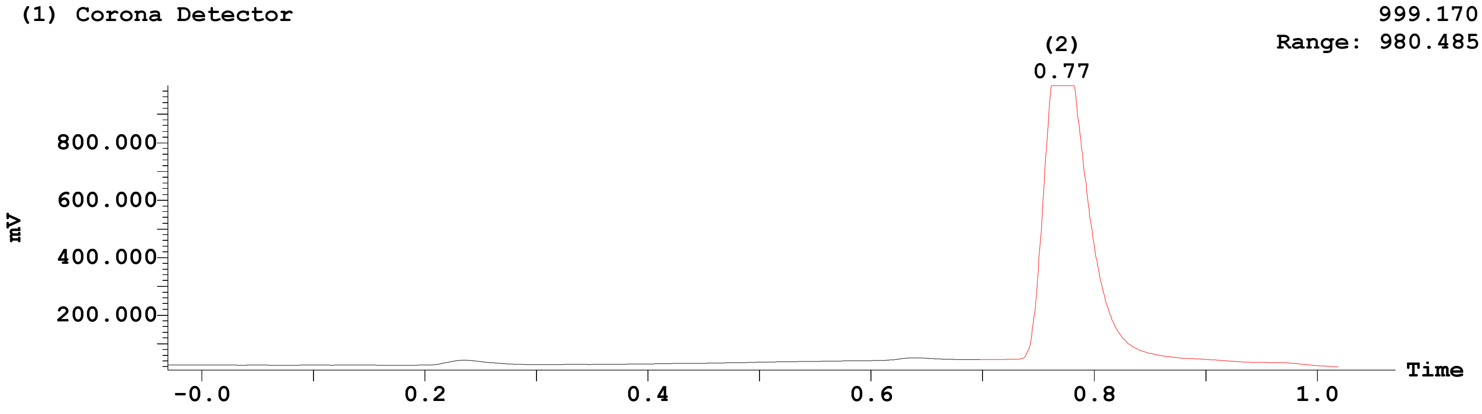
(1) Corona Detector
999.170
Range: 980.485
(2)
0.77
mV
800.000
600.000
400.000
200.000
-0.0
0.2
0.4
0.6
0.8
1.0
Time

File:13zo665l2 Vial:5:47 ID:C9
Method:C:\MASSLYNX\1minLC_MS.olp

Peak ID	Time
1	0.43

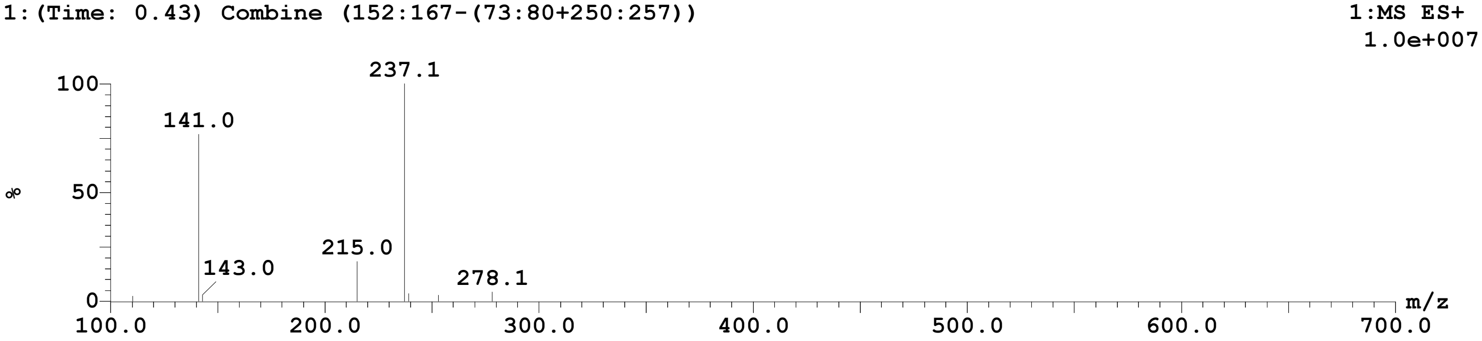

Peak ID	Time
2	0.76

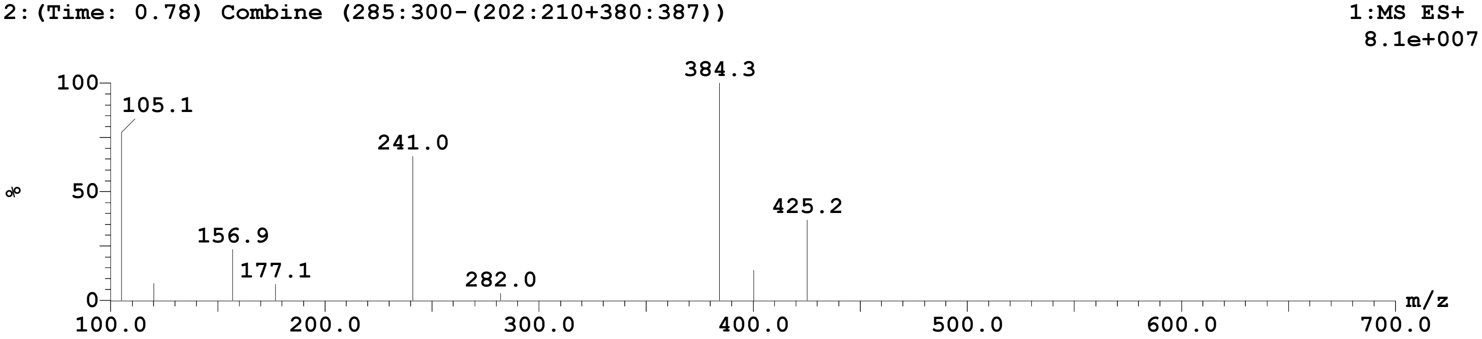

Peak ID	Time
2	0.76

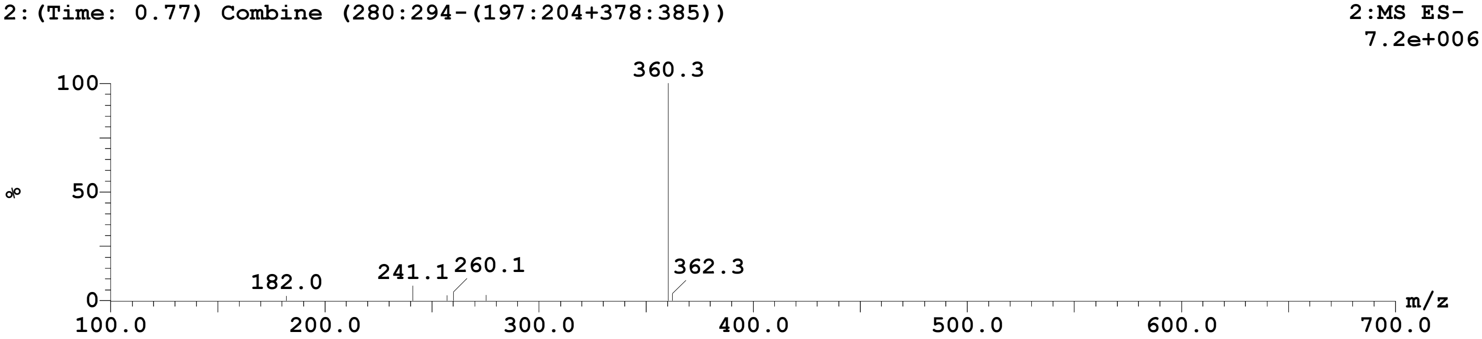

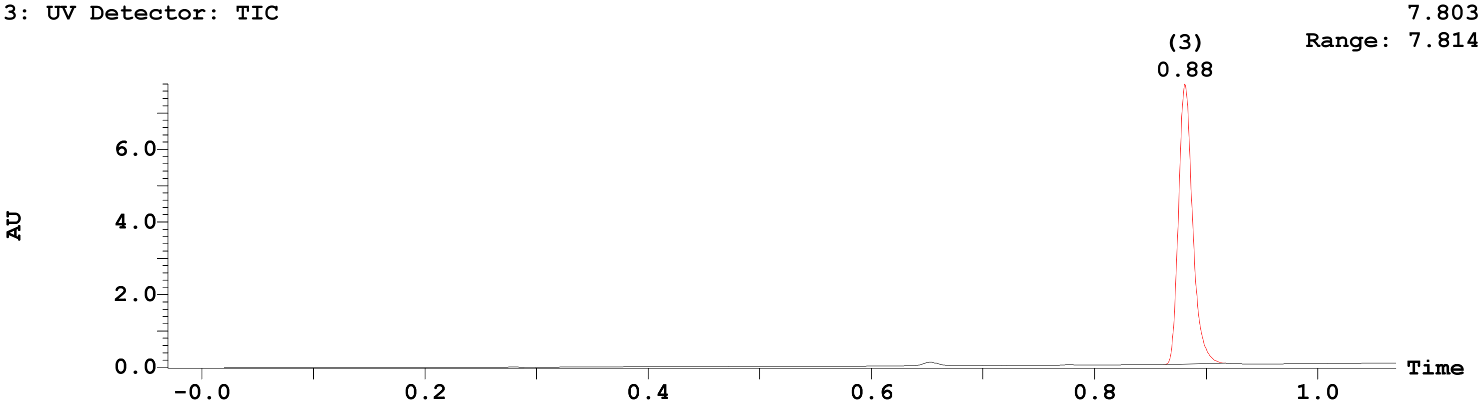
3: UV Detector: TIC
7.803
Range: 7.814
(3)
0.88
AU
6.0
4.0
2.0
0.0
Time
-0.0
0.2
0.4
0.6
0.8
1.0

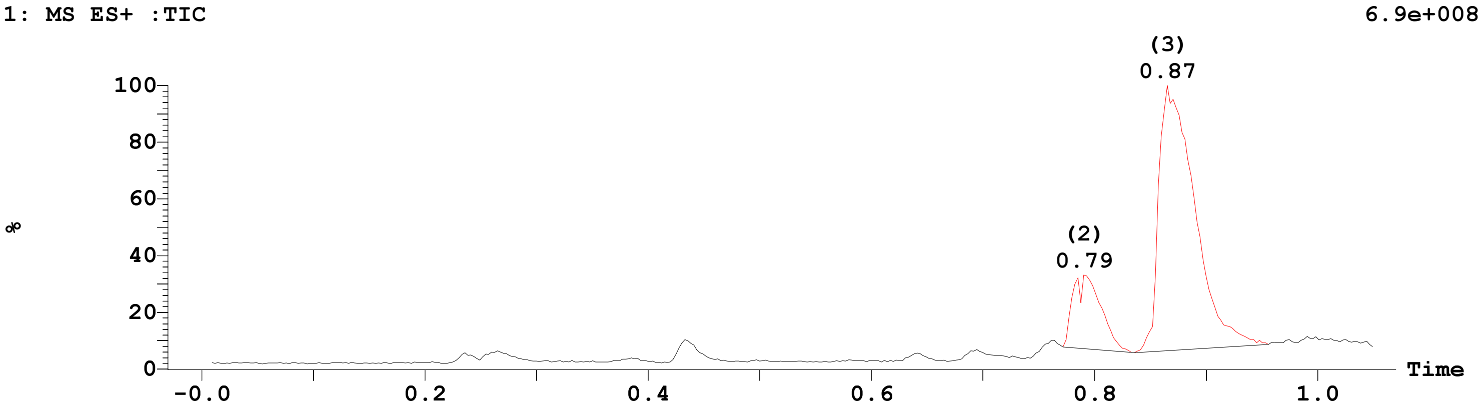
1: MS ES+ :TIC
6.9e+008
(3)
0.87
(2)
0.79
%
100
80
60
40
20
0
Time
-0.0
0.2
0.4
0.6
0.8
1.0

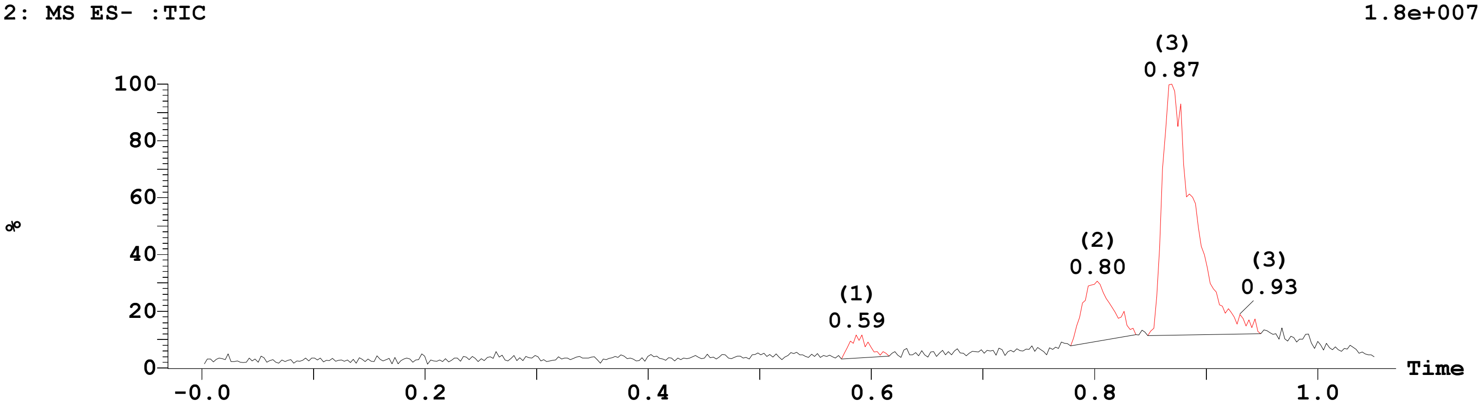
2: MS ES- :TIC
1.8e+007
(3)
0.87
(2)
0.80
(3)
0.93
(1)
0.59
%
100
80
60
40
20
0
Time
-0.0
0.2
0.4
0.6
0.8
1.0

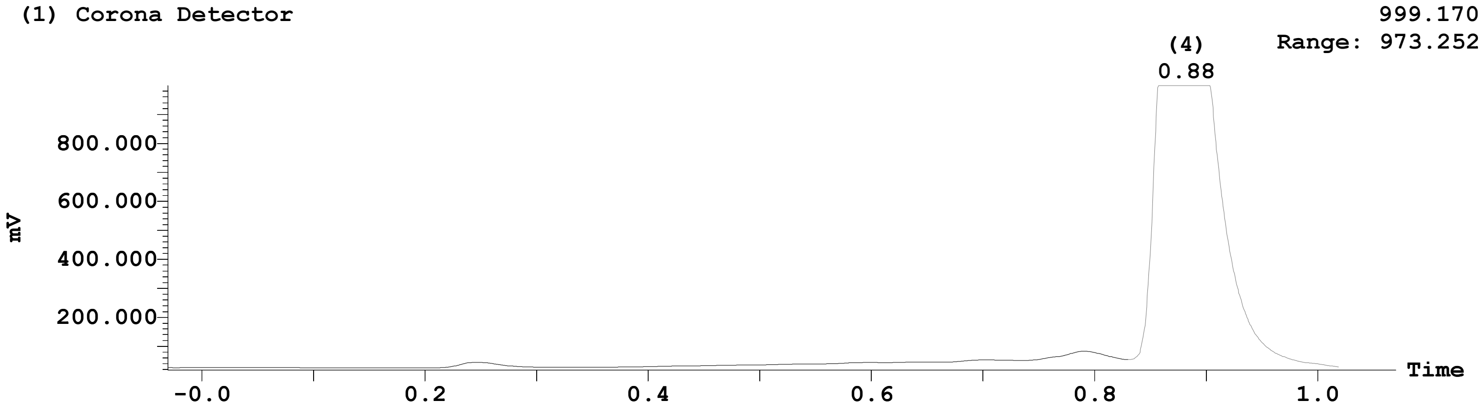
(1) Corona Detector
999.170
Range: 973.252
(4)
0.88
mV
800.000
600.000
400.000
200.000
Time
-0.0
0.2
0.4
0.6
0.8
1.0

File:13zp619l2 Vial:5:48 ID:C10
Method:C:\MASSLYNX\1minLC_MS.olp

Peak ID **Time**
1 0.59

1:(Time: 0.59) Combine (213:228-(133:140+306:313)) 2:MS ES-

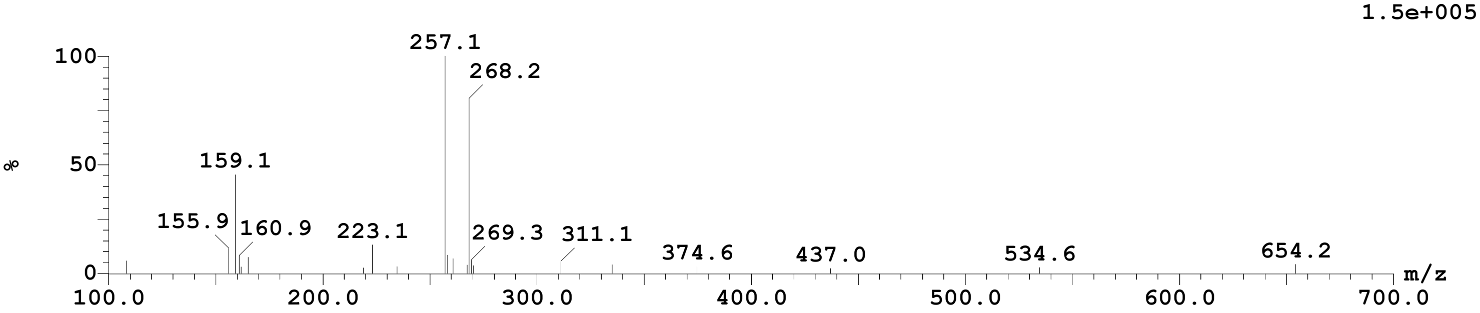

Peak ID **Time**
2 0.79

2:(Time: 0.79) Combine (290:304-(208:215+388:395)) 1:MS ES+

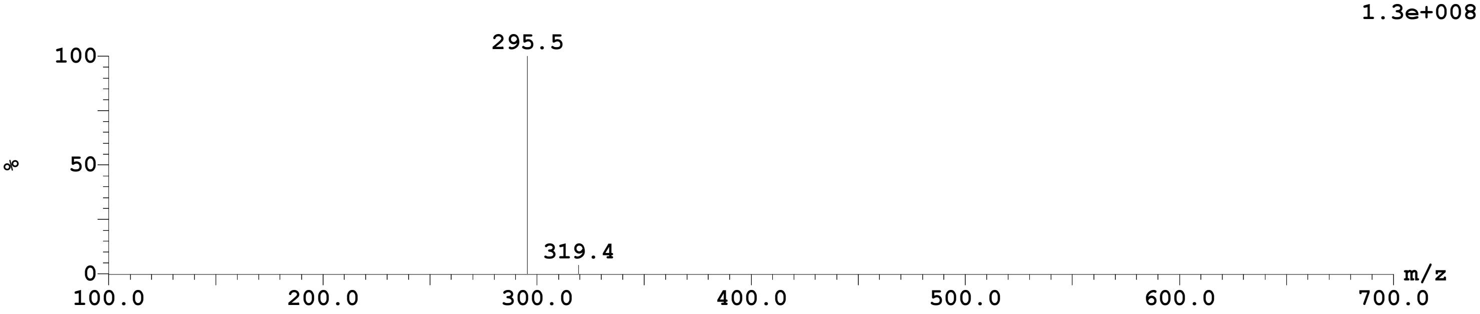

Peak ID **Time**
2 0.79

2:(Time: 0.80) Combine (294:308-(210:217+389:396)) 2:MS ES-

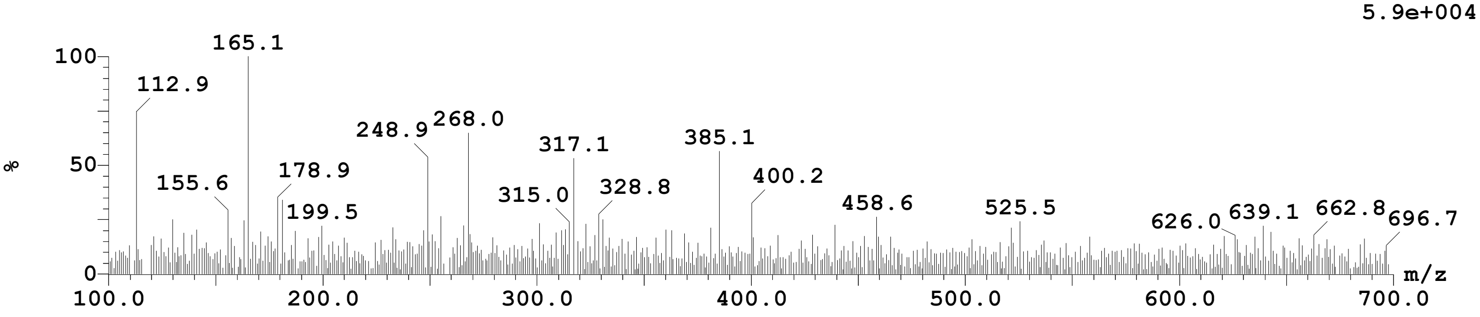

Peak ID **Time**
3 0.87

3:(Time: 0.88) Combine (323:338-241:249) 1:MS ES+

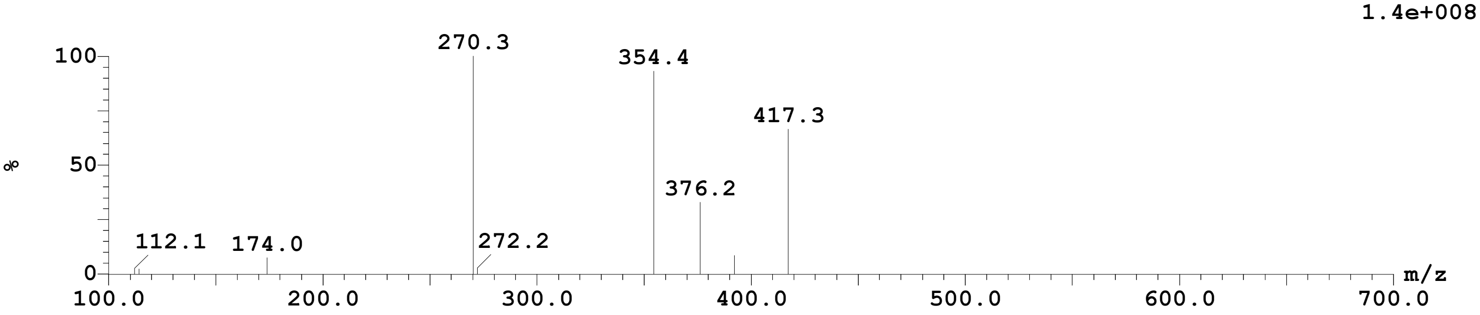

File:13zp619l2 Vial:5:48 ID:C10
Method:C:\MASSLYNX\1minLC_MS.olp

Peak ID **Time**
3 0.87

3: (Time: 0.88) Combine (323:338-241:248) 2:MS ES- 4.2e+006

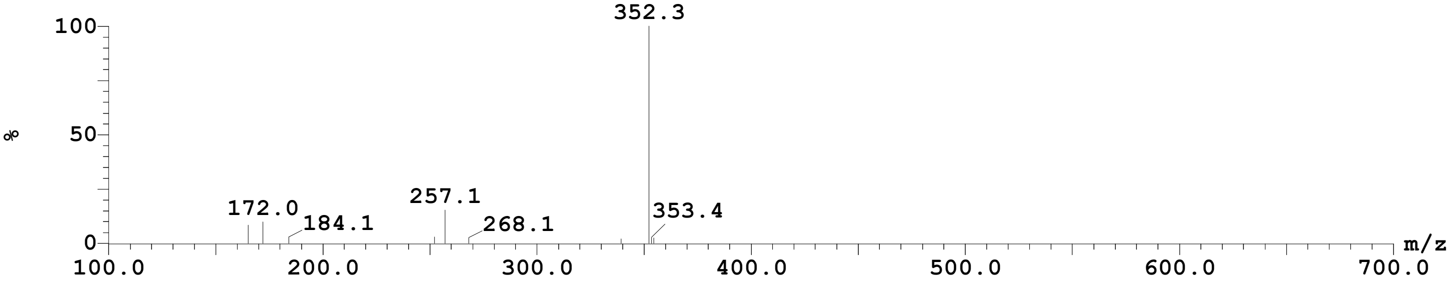

SYNGENTA SQD OPEN ACCESS LC-MS Page 1

File:13zn234l2 Vial:5:51 ID:**D1**

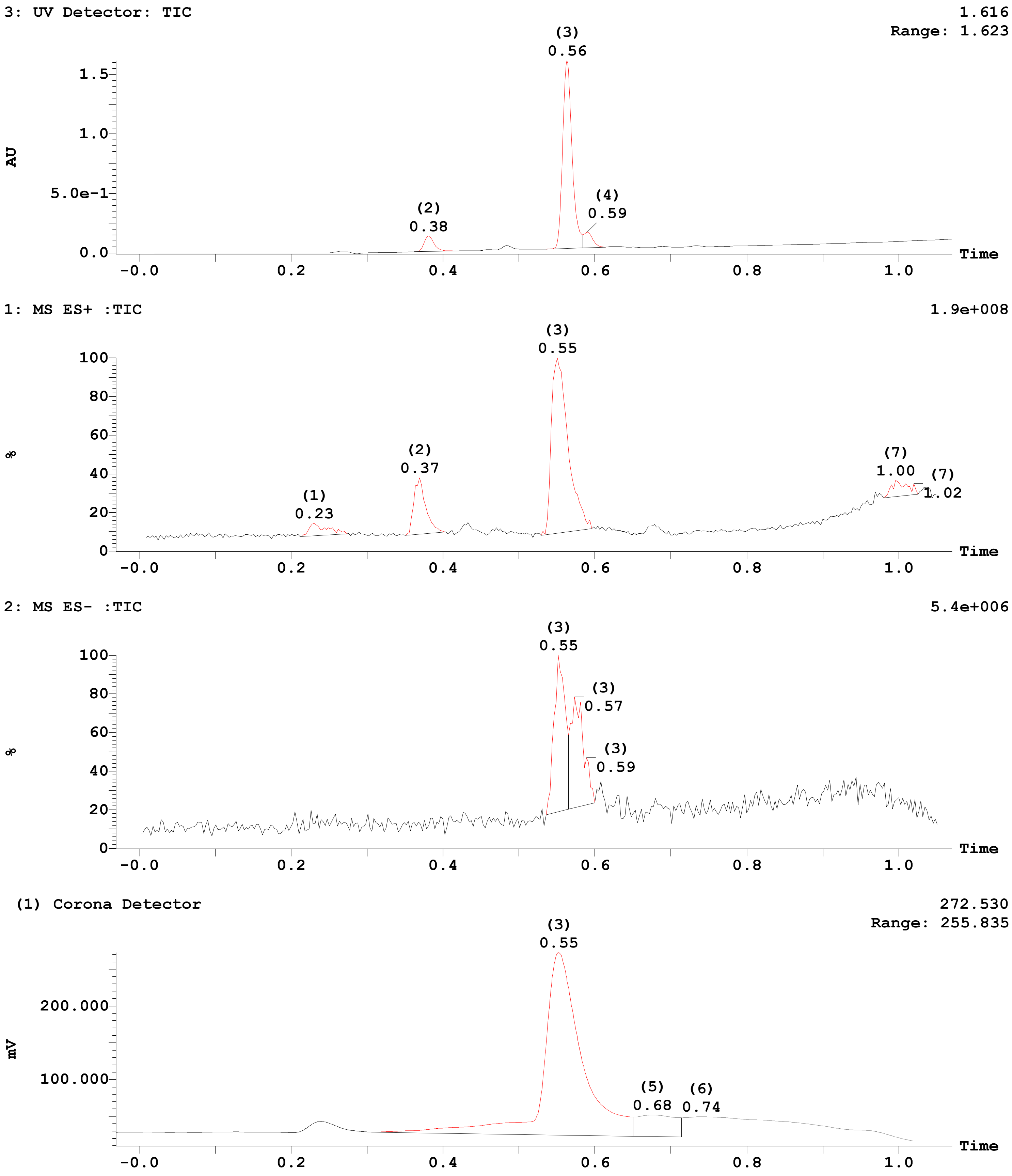

File:13zn234l2 Vial:5:51 ID:D1
Method:C:\MASSLYNX\1minLC_MS.olp

Peak ID 1 **Time** 0.23

1:(Time: 0.23) Combine (79:94-(1:6+178:185)) 1:MS ES+ 1.8e+006

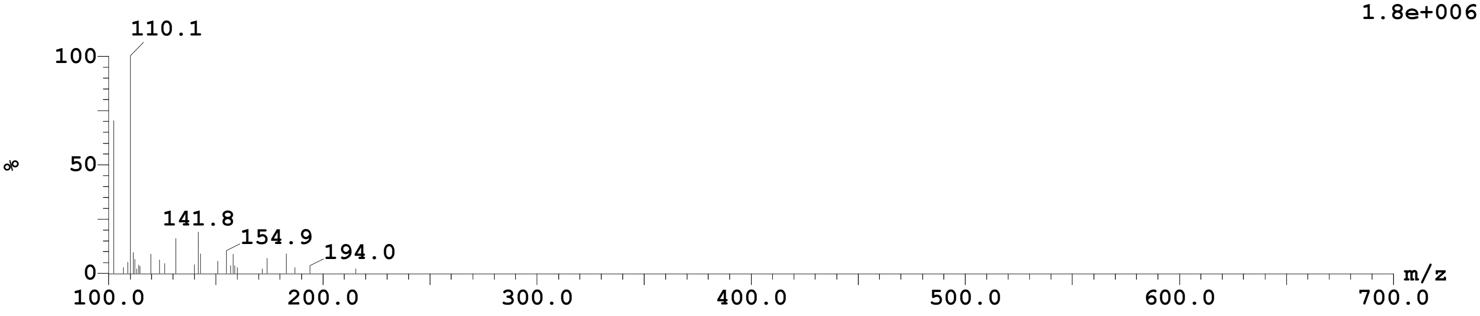

Peak ID 2 **Time** 0.37

2:(Time: 0.37) Combine (132:146-(50:57+227:234)) 1:MS ES+ 2.5e+007

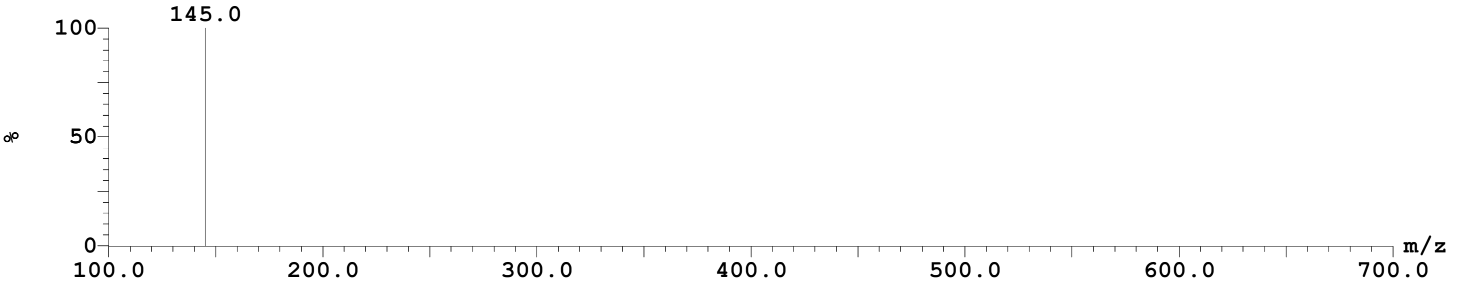

Peak ID 2 **Time** 0.37

2:(Time: 0.38) Combine (135:150-(54:61+233:240)) 2:MS ES- 5.8e+003

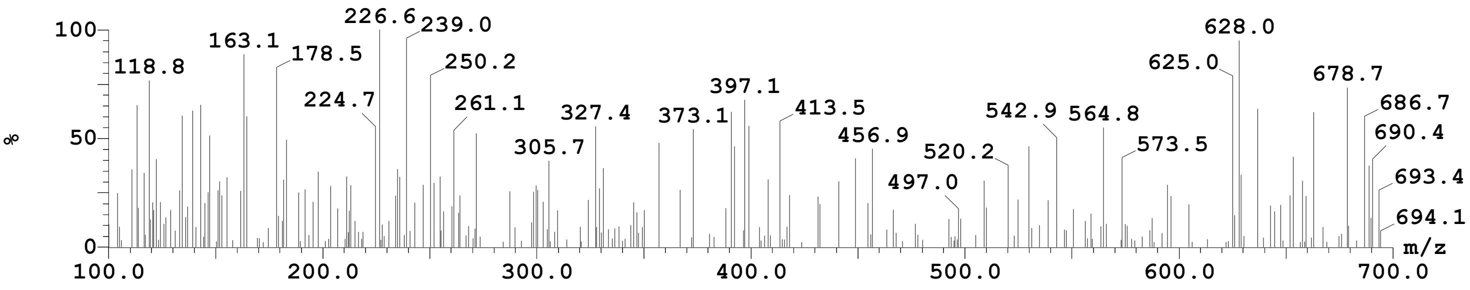

Peak ID 3 **Time** 0.55

3:(Time: 0.56) Combine (204:219-(119:127+295:302)) 1:MS ES+ 7.8e+007

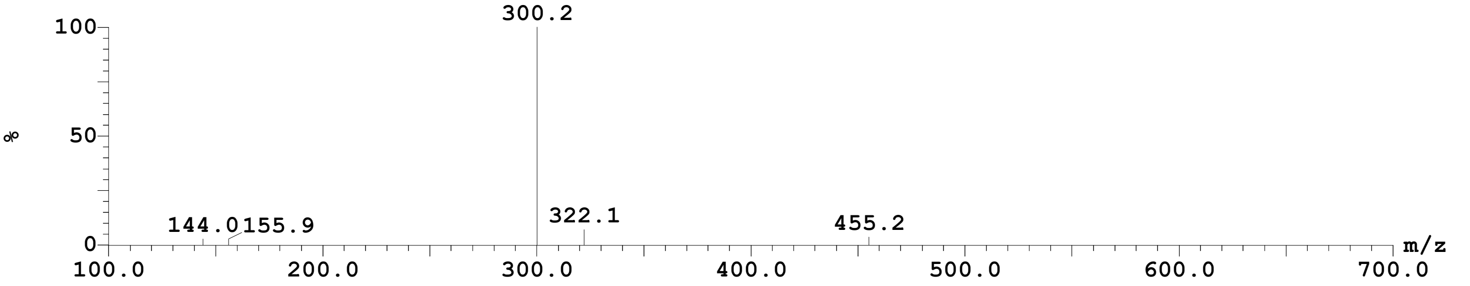

File:13zn234l2 Vial:5:51 ID:D1
Method:C:\MASSLYNX\1minLC_MS.olp

Peak ID	Time
3	0.55

3:(Time: 0.56) Combine (204:219-(119:126+294:302)) 2:MS ES- 8.7e+005

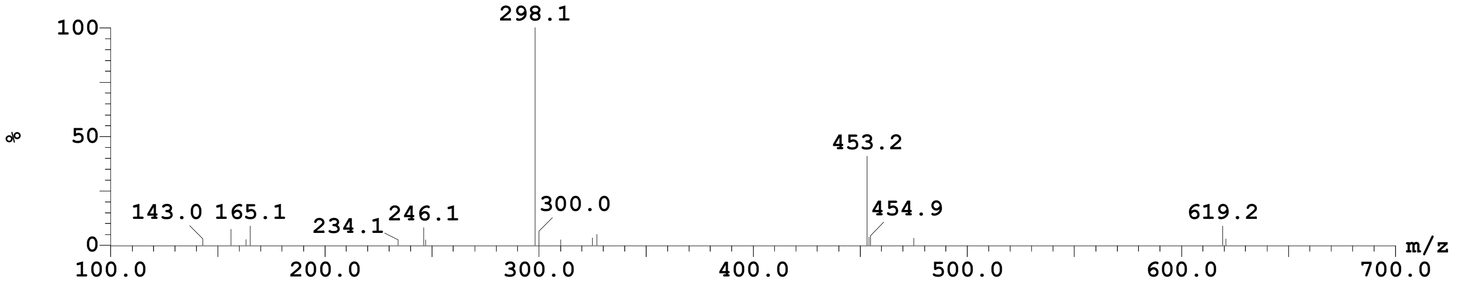

Peak ID	Time
4	0.59

4:(Time: 0.59) Combine (214:229-(137:145+306:313)) 1:MS ES+ 1.1e+007

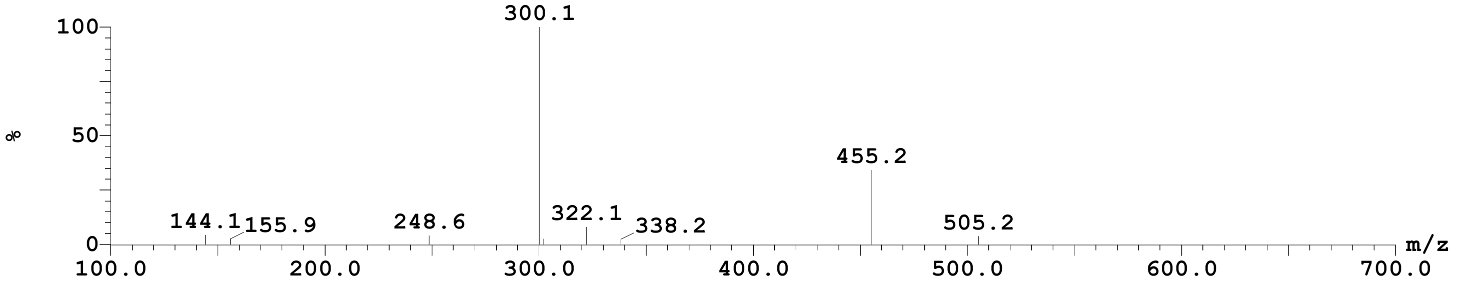

Peak ID	Time
4	0.59

4:(Time: 0.59) Combine (214:229-(137:144+305:313)) 2:MS ES- 4.2e+005

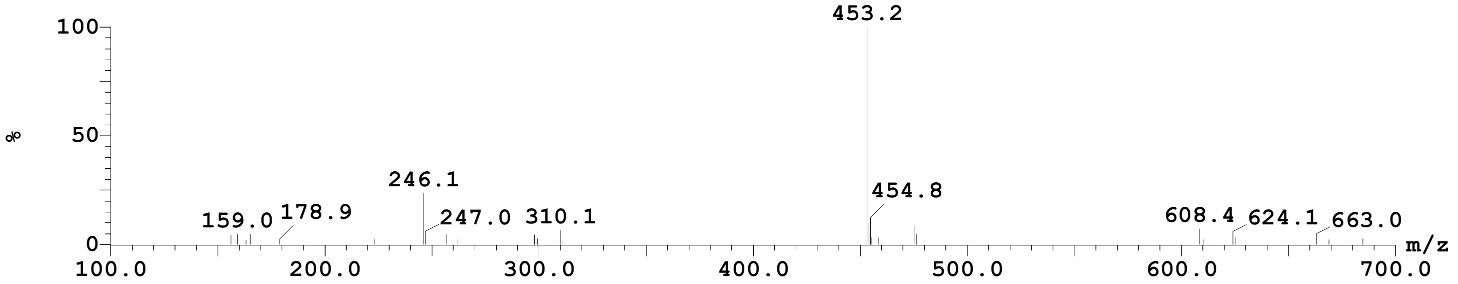

Peak ID	Time
7	1.00

7:(Time: 1.00) Combine (367:382-286:293) 1:MS ES+ 2.5e+007

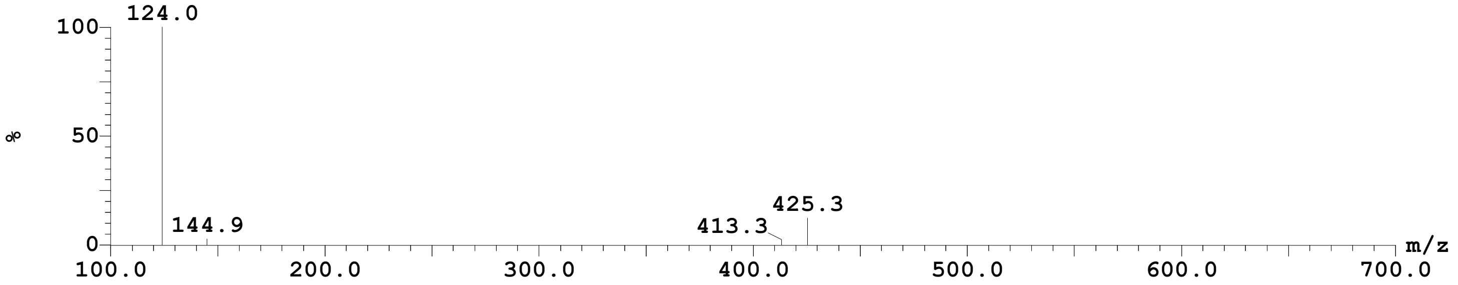

SYNGENTA SQD OPEN ACCESS LC-MS Page 1

File:13zn254l2 Vial:5:52 ID:**D2**

3: UV Detector: TIC 7.423

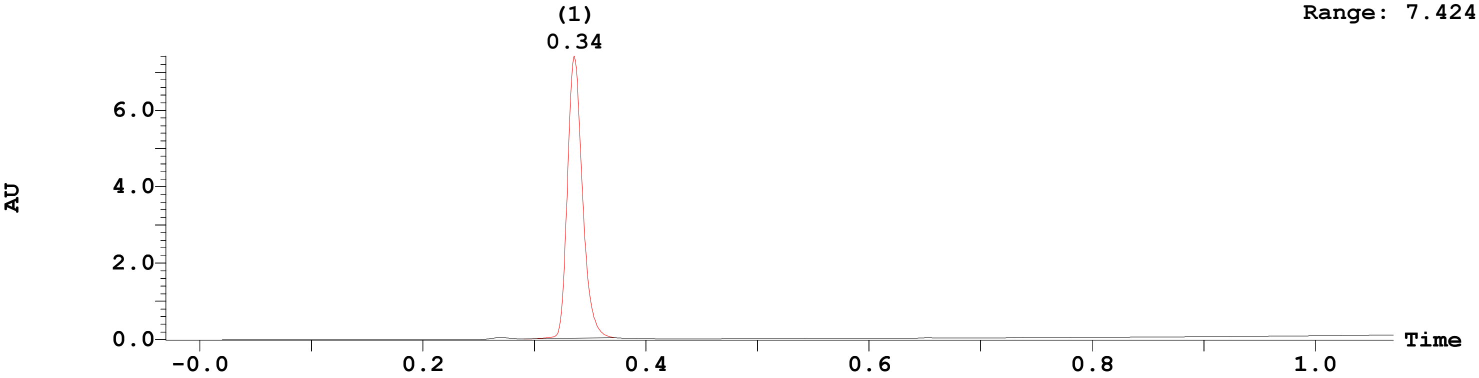

1: MS ES+ :TIC 3.7e+008

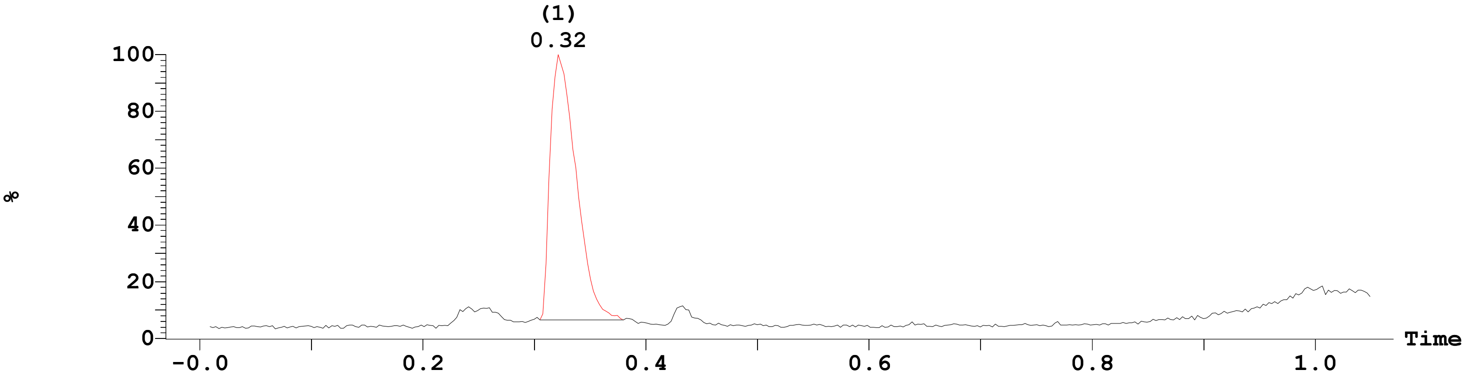

2: MS ES- :TIC 5.6e+006

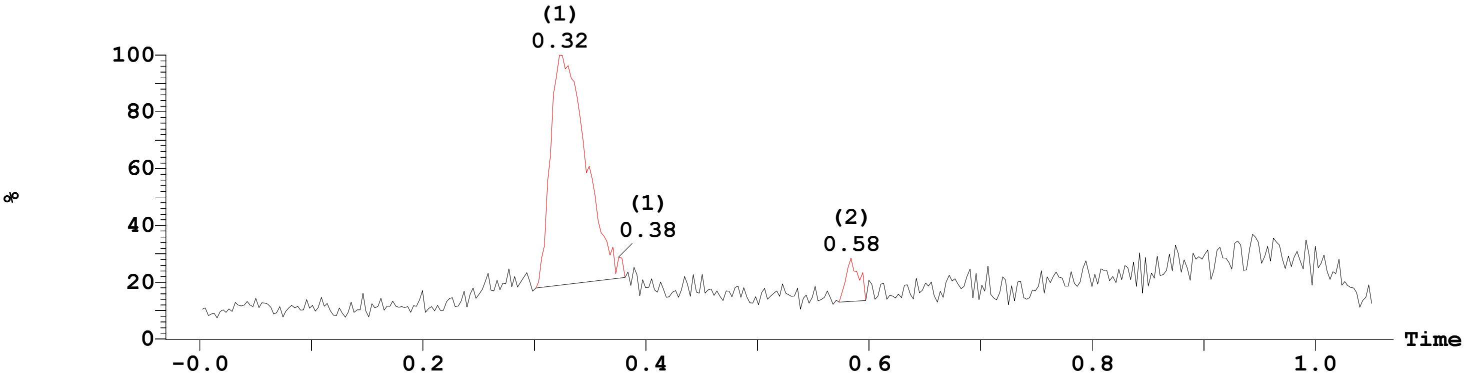

(1) Corona Detector 738.380

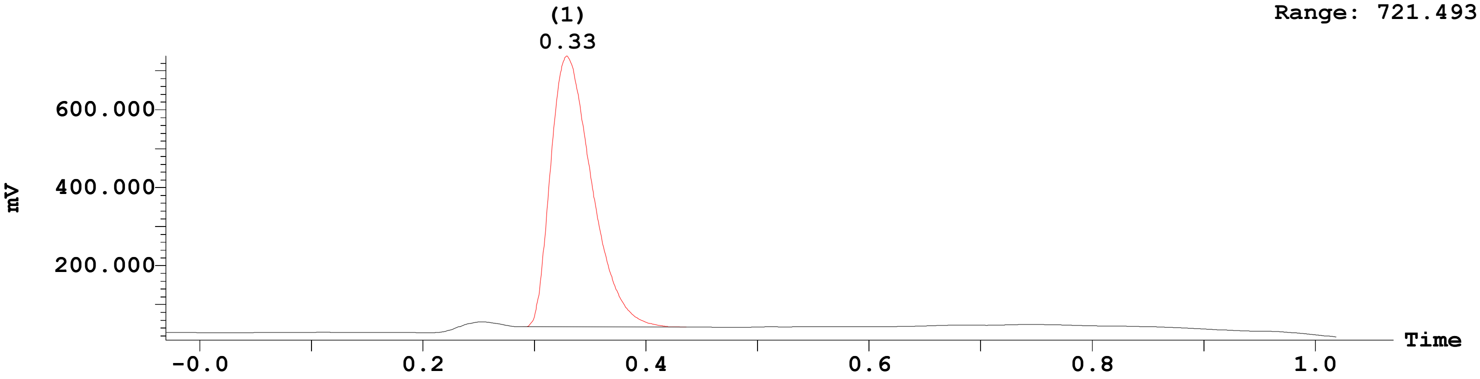

File:13zn254l2 Vial:5:52 ID: D2
Method:C:\MASSLYNX\1minLC_MS.olp

Peak ID **Time**
1 0.32

1:(Time: 0.32) Combine (114:129-(33:40+218:225)) 1:MS ES+ 1.1e+008

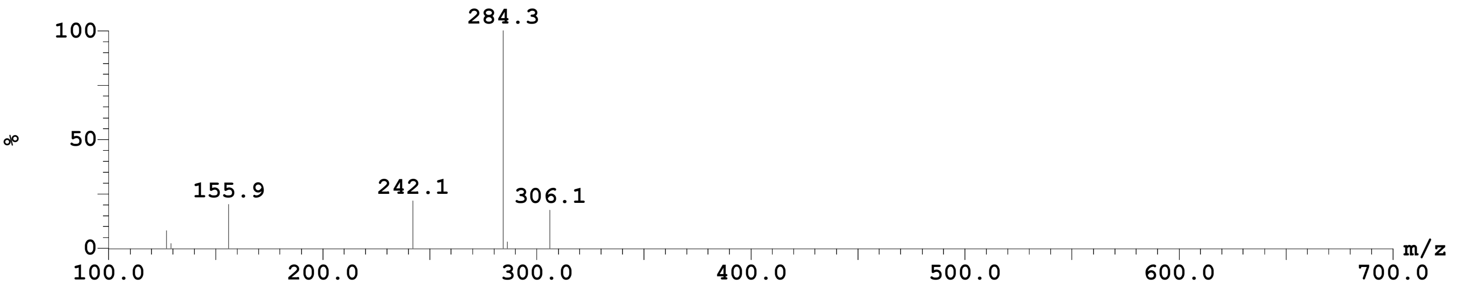

Peak ID **Time**
1 0.32

1:(Time: 0.34) Combine (119:134-(27:34+215:222)) 2:MS ES- 2.3e+005

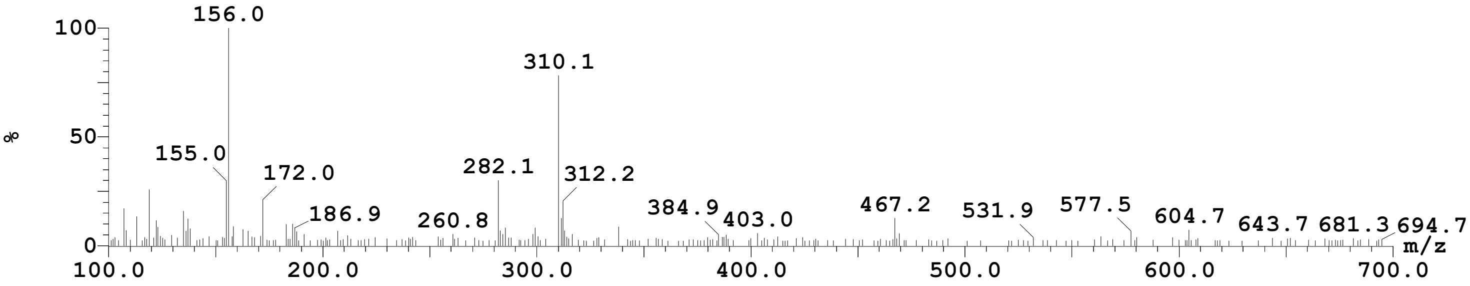

Peak ID **Time**
2 0.58

2:(Time: 0.58) Combine (212:226-(133:140+299:306)) 2:MS ES- 1.1e+005

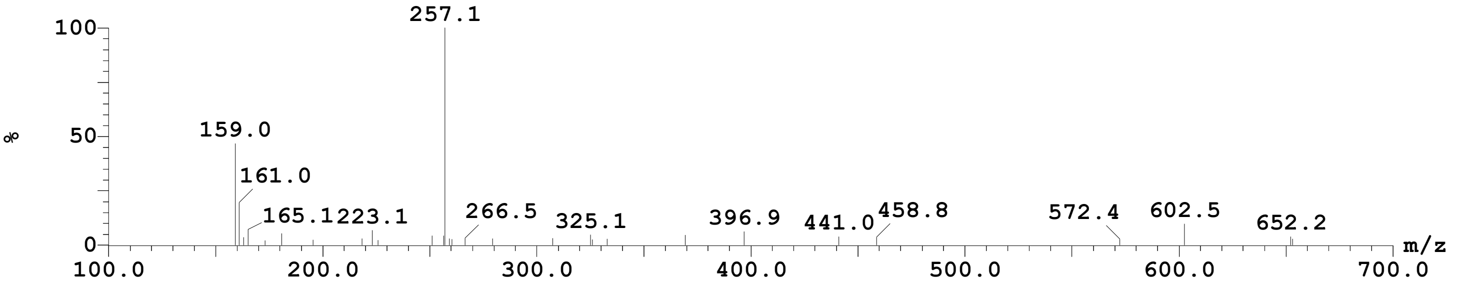

SYNGENTA SQD OPEN ACCESS LC-MS

File:13zn281l2 Vial:5:53 ID: **D3**

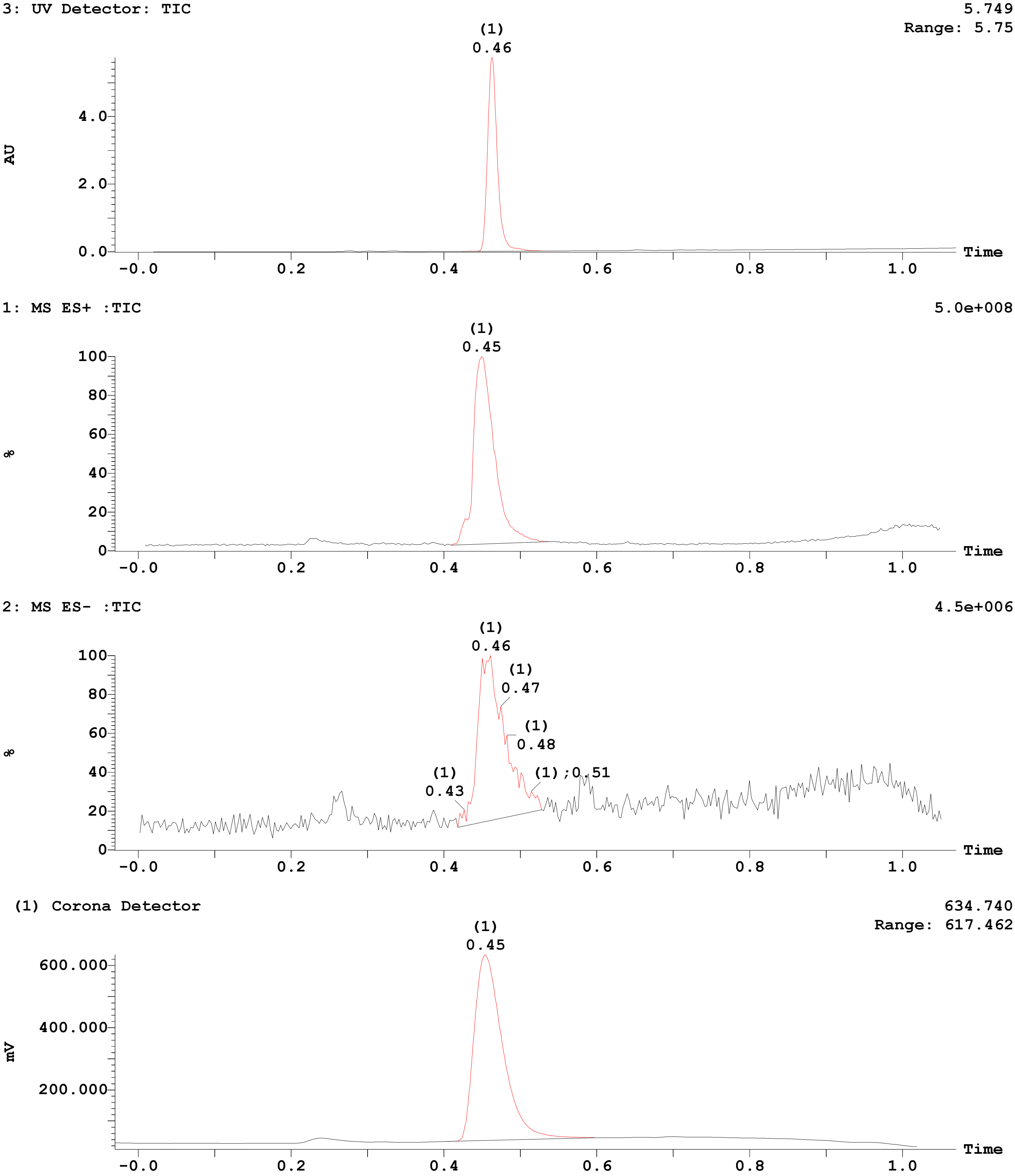

File:13zn281l2 Vial:5:53 ID:D3
Method:C:\MASSLYNX\1minLC_MS.olp

Peak ID **Time**
1 0.45

1:(Time: 0.45) Combine (161:176-(72:79+277:284)) 1:MS ES+ 1.1e+008

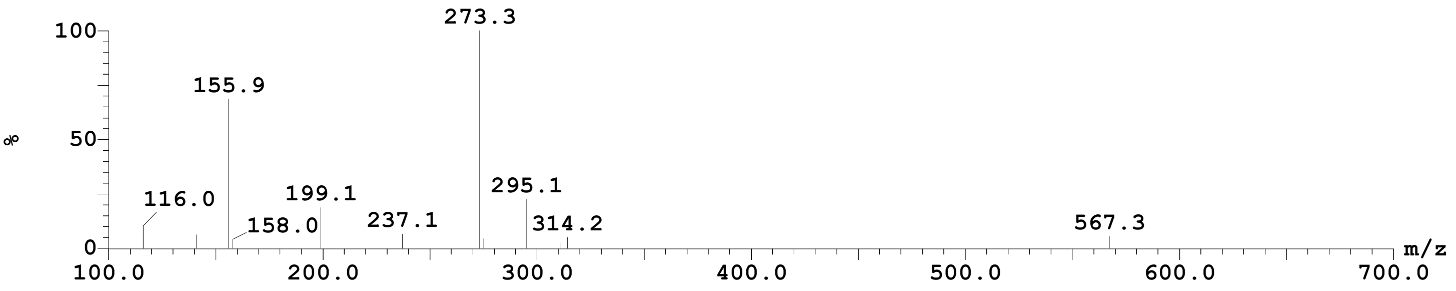

Peak ID **Time**
1 0.45

1:(Time: 0.46) Combine (166:181-(75:82+273:280)) 2:MS ES- 1.7e+005

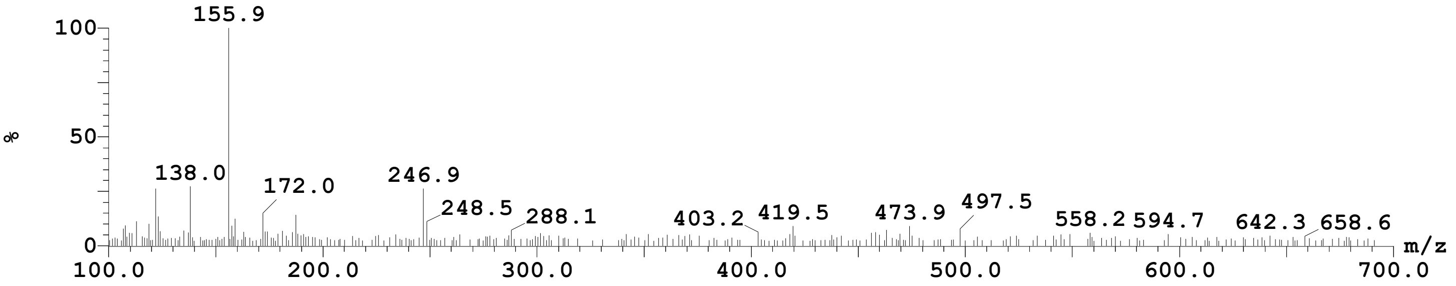

SYNGENTA SQD OPEN ACCESS LC-MS Page 1

File:13zo666l2 Vial:5:50 ID: **D4**

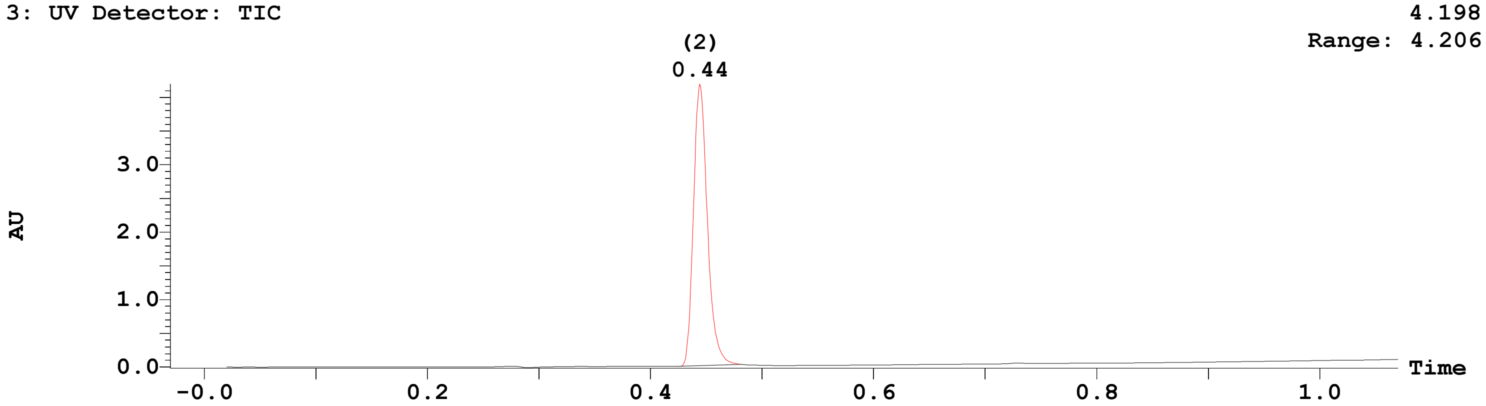

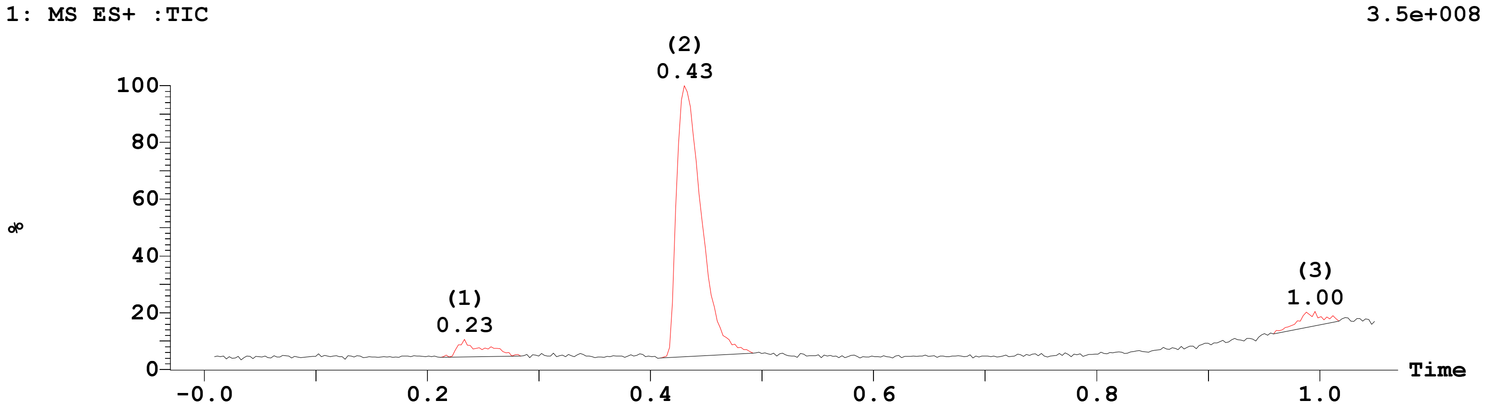

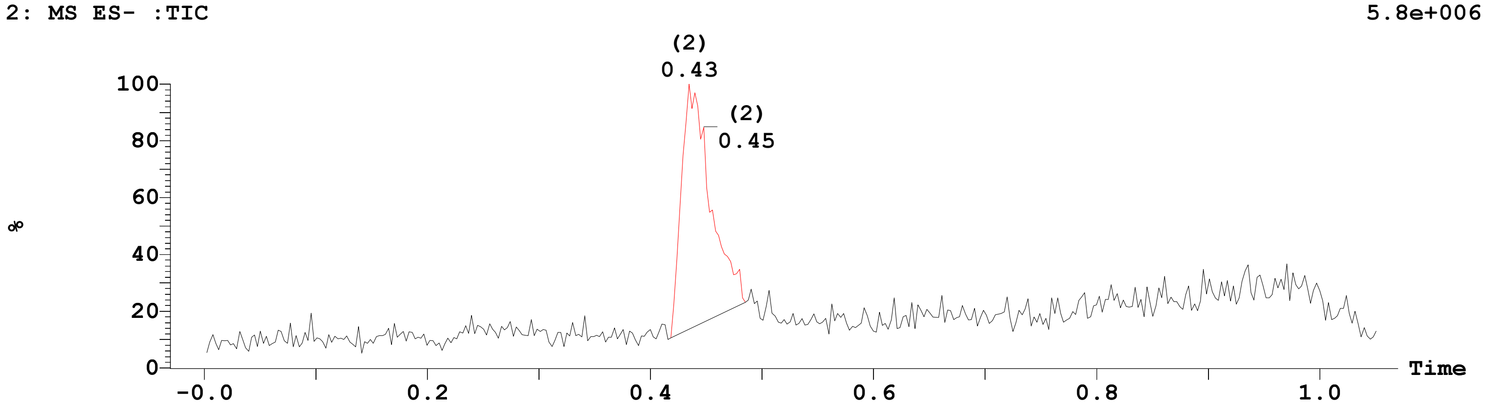

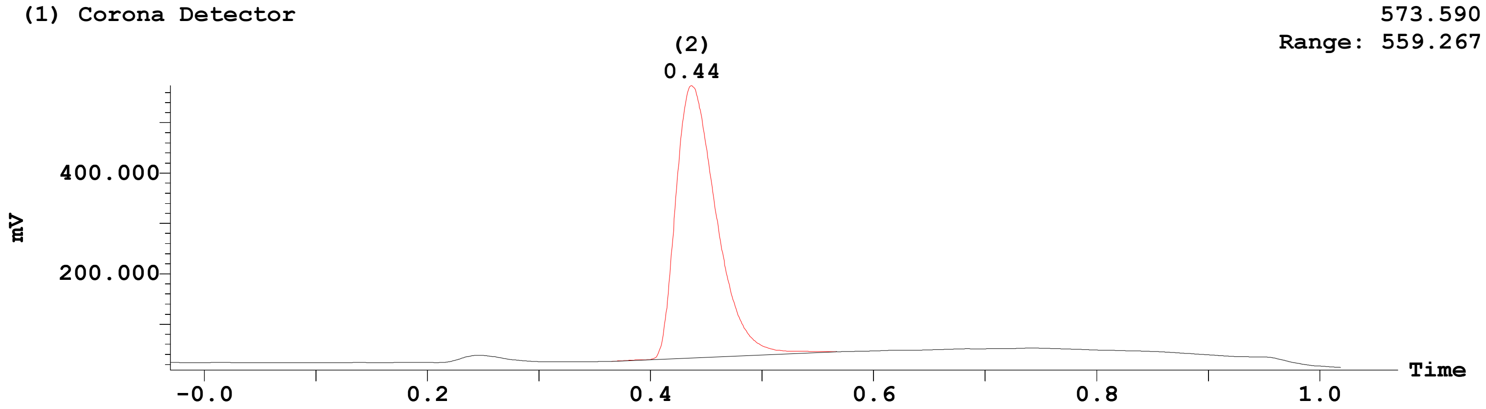

File:13zo666l2 Vial:5:50 ID:D4
Method:C:\MASSLYNX\1minLC_MS.olp

Peak ID	Time
1	0.23

1:(Time: 0.23) Combine (81:95-(1:5+182:189)) 1:MS ES+ 3.2e+006

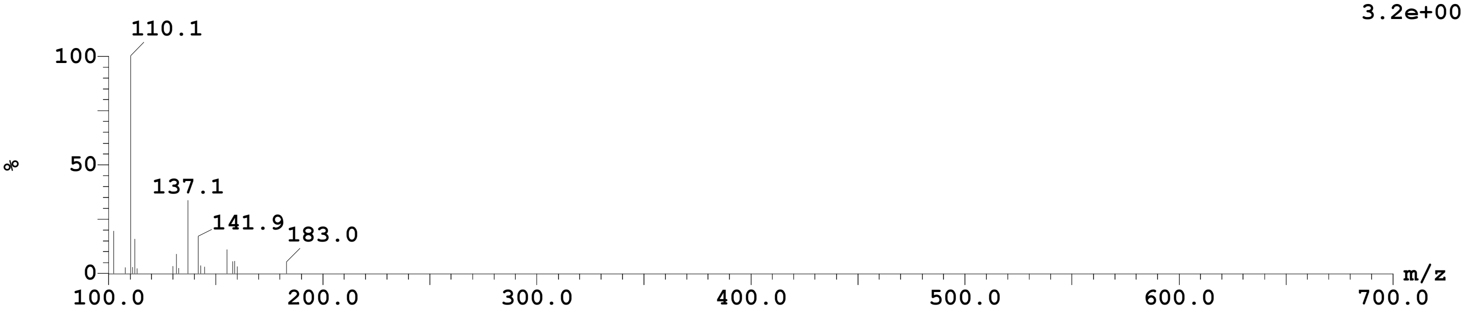

Peak ID	Time
2	0.43

2:(Time: 0.44) Combine (160:175-(77:85+256:264)) 1:MS ES+ 9.5e+007

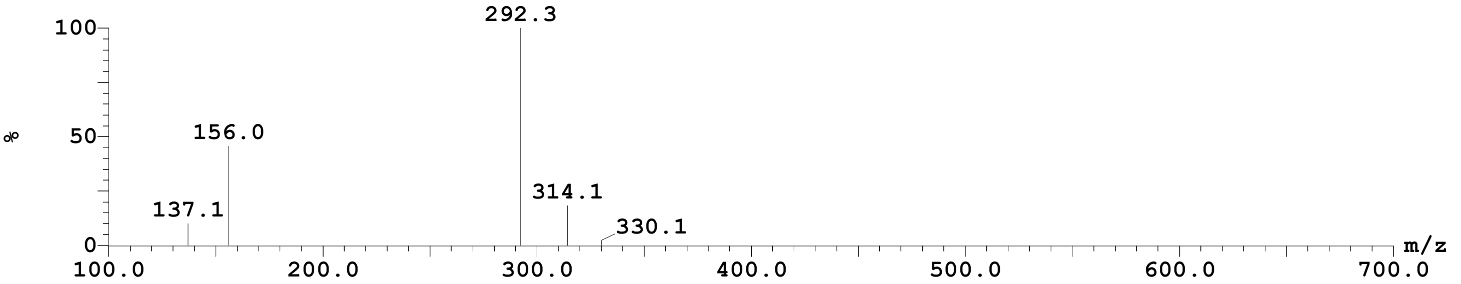

Peak ID	Time
2	0.43

2:(Time: 0.43) Combine (155:170-(75:82+257:264)) 2:MS ES- 2.4e+005

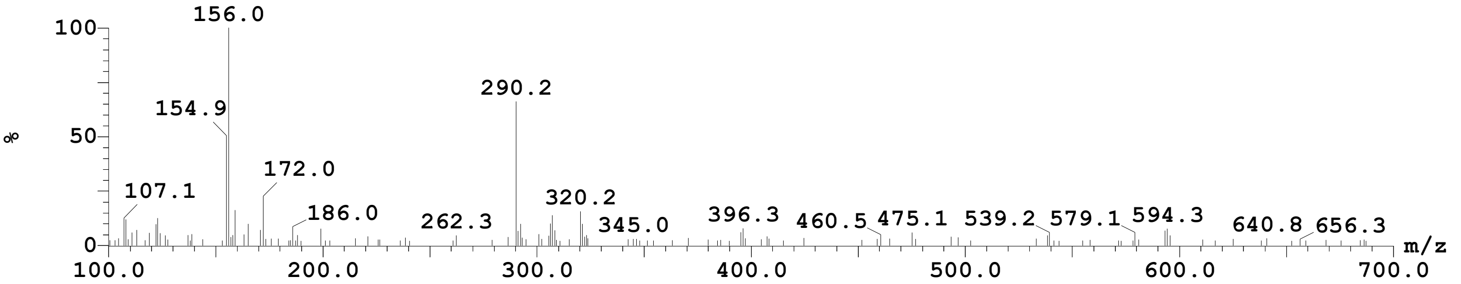

Peak ID	Time
3	1.00

3:(Time: 1.00) Combine (366:381-278:285) 1:MS ES+ 2.5e+007

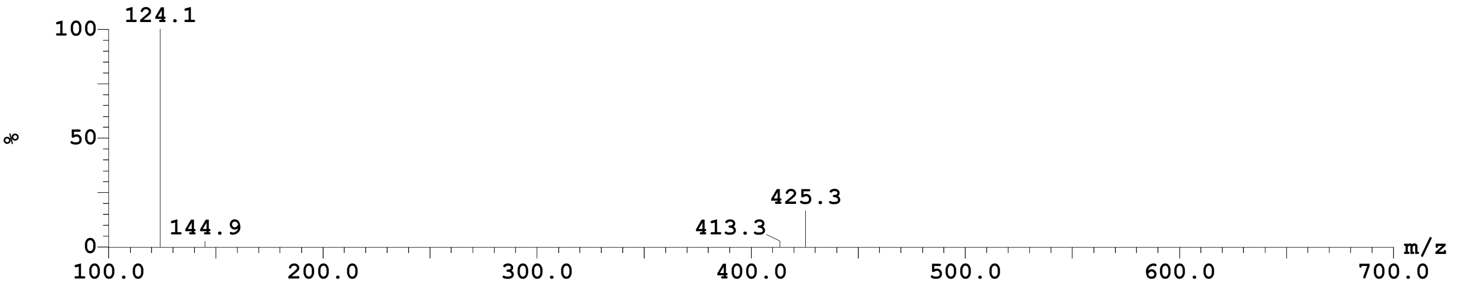

File:13zn482l2 Vial:5:51 ID: **D5**

3: UV Detector: TIC 1.472e+1
Range: 1.472e+1

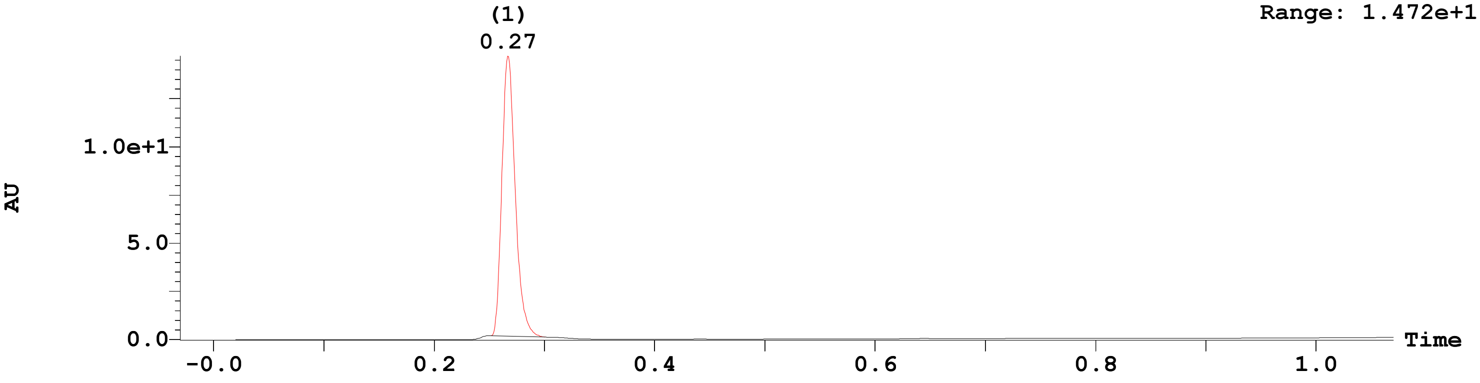

1: MS ES+ :TIC 3.3e+008

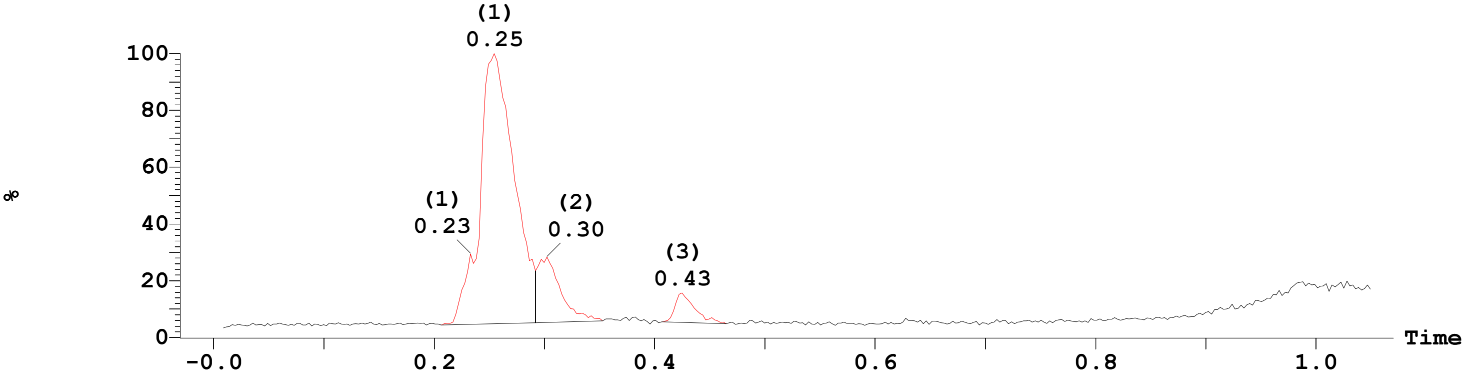

2: MS ES- :TIC 1.3e+007

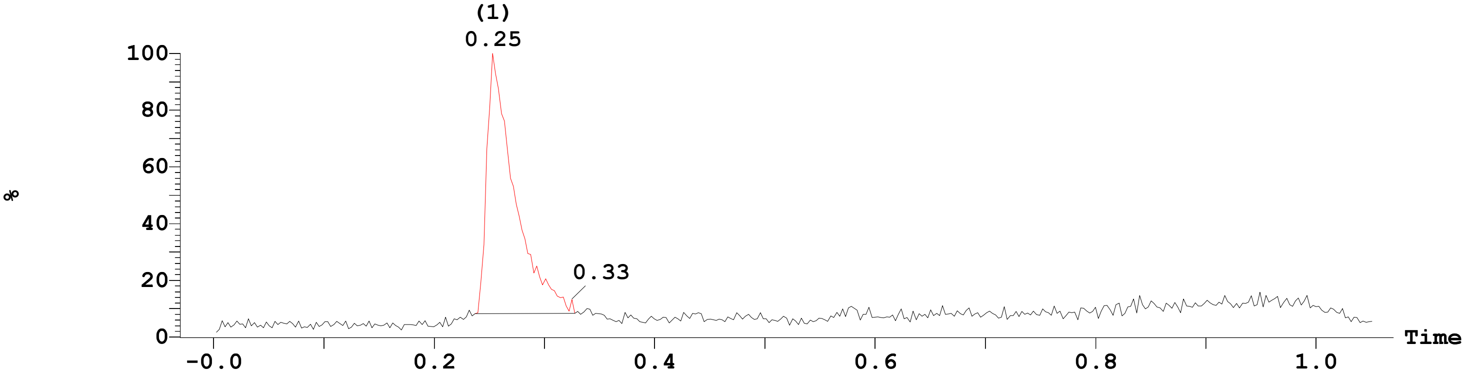

(1) Corona Detector 894.790
Range: 875.615

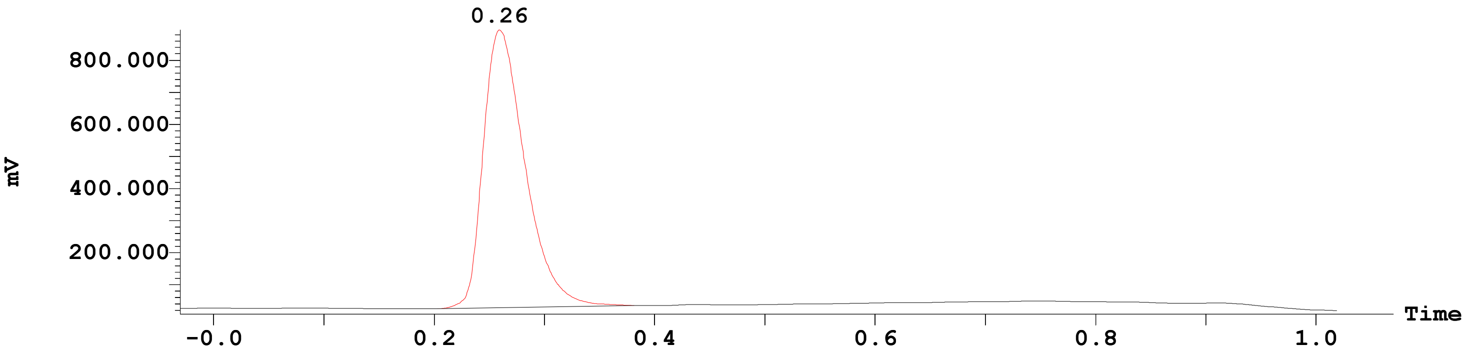

File:13zn482l2 Vial:5:51 ID:D5
Method:C:\MASSLYNX\1minLC_MS.olp

Peak ID **Time**
1 0.25

1:(Time: 0.27) Combine (93:108-(12:20+188:196)) 1:MS ES+
1.6e+008

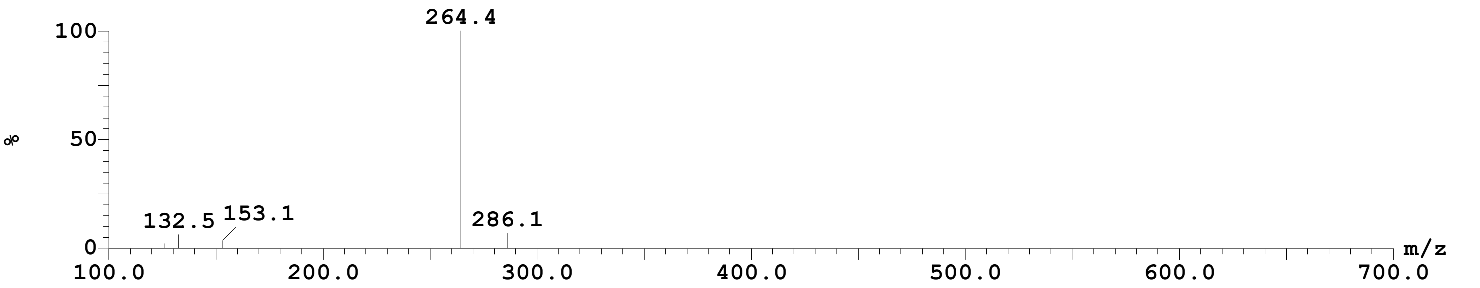

Peak ID **Time**
1 0.25

1:(Time: 0.25) Combine (87:102-(7:14+198:205)) 2:MS ES-
2.2e+006

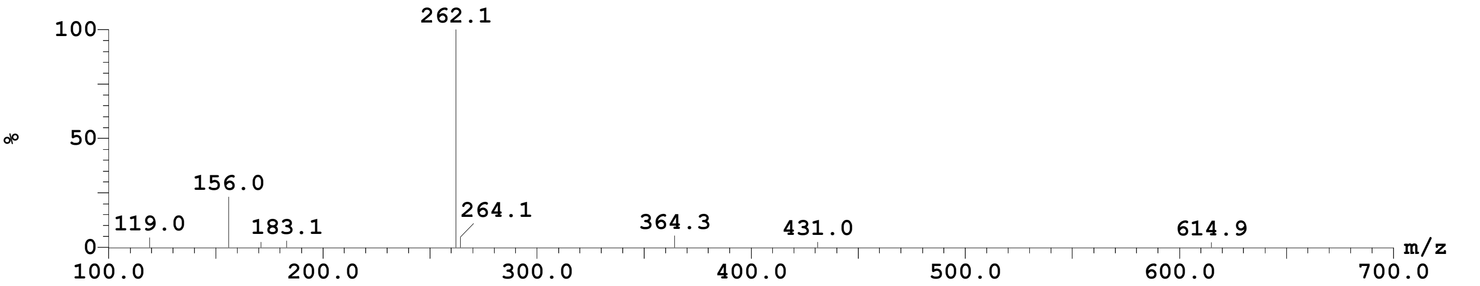

Peak ID **Time**
2 0.30

2:(Time: 0.30) Combine (106:121-(28:35+208:215)) 1:MS ES+
2.7e+007

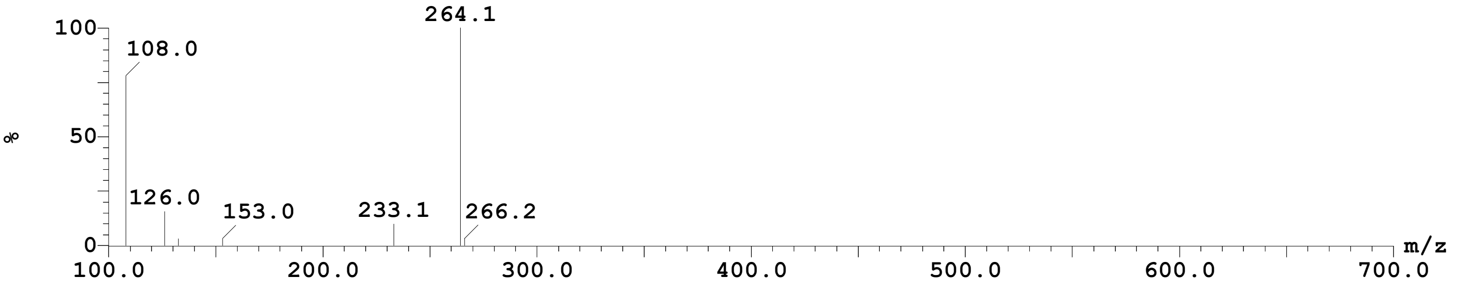

Peak ID **Time**
3 0.43

3:(Time: 0.43) Combine (152:168-(71:78+250:257)) 1:MS ES+
6.1e+006

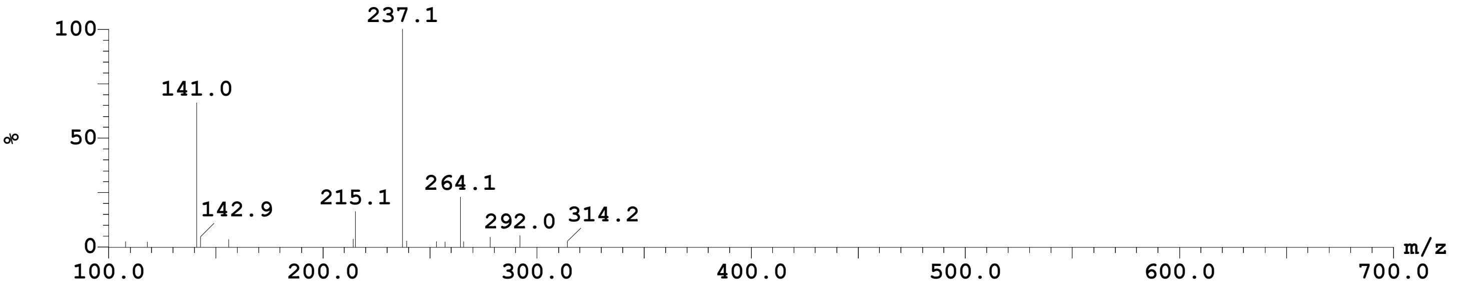

SYNGENTA SQD OPEN ACCESS LC-MS

File:13zn259l4 Vial:5:52 ID: **D6**

3: UV Detector: TIC

8.94
Range: 8.942

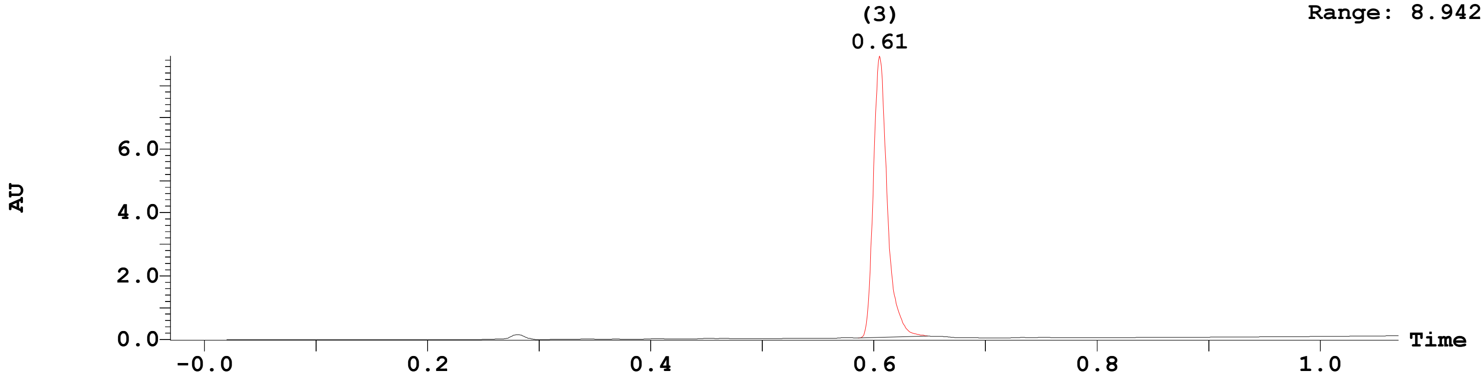

1: MS ES+ :TIC

6.9e+008

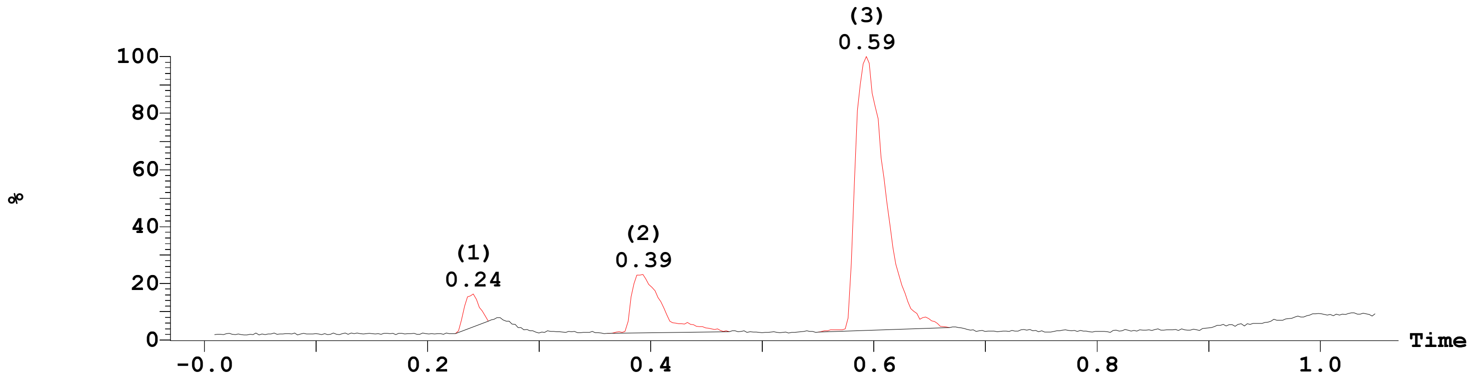

2: MS ES- :TIC

2.9e+007

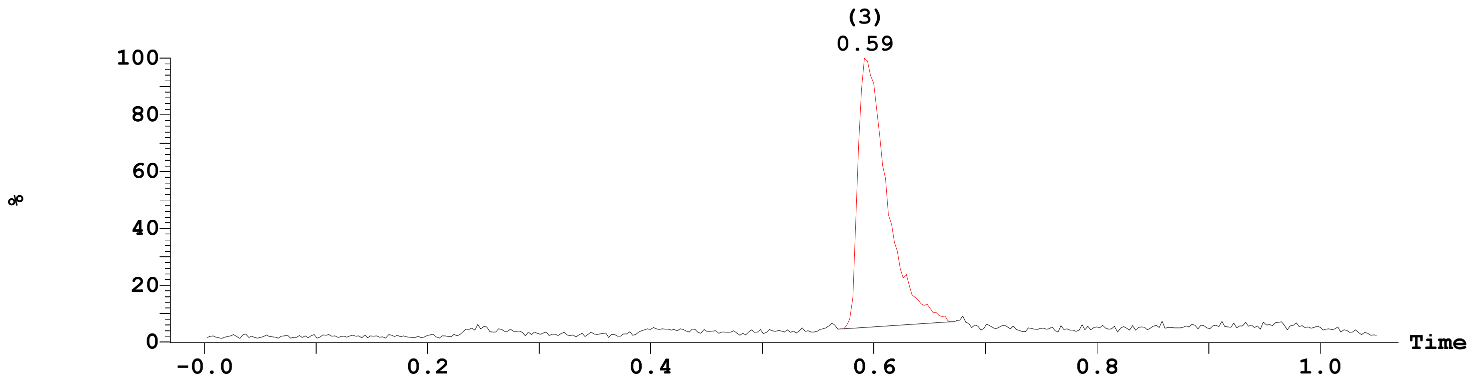

(1) Corona Detector

999.160
Range: 980.196

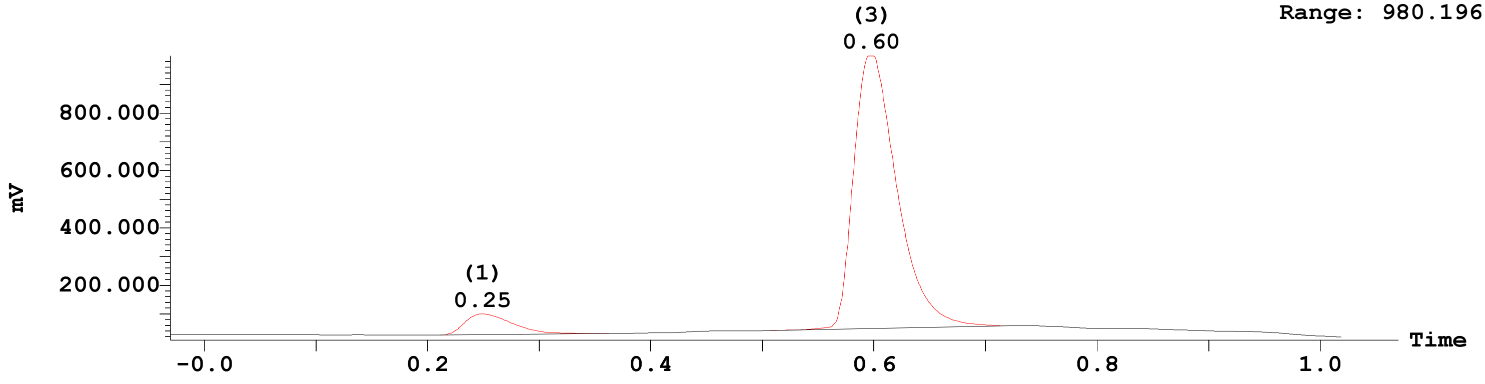

File:13zn259l4 Vial:5:52 ID:D6
Method:C:\MASSLYNX\1minLC_MS.olp

Peak ID	Time
1	0.24

1:(Time: 0.24) Combine (84:99-(3:10+171:178)) 1:MS ES+ 1.8e+007

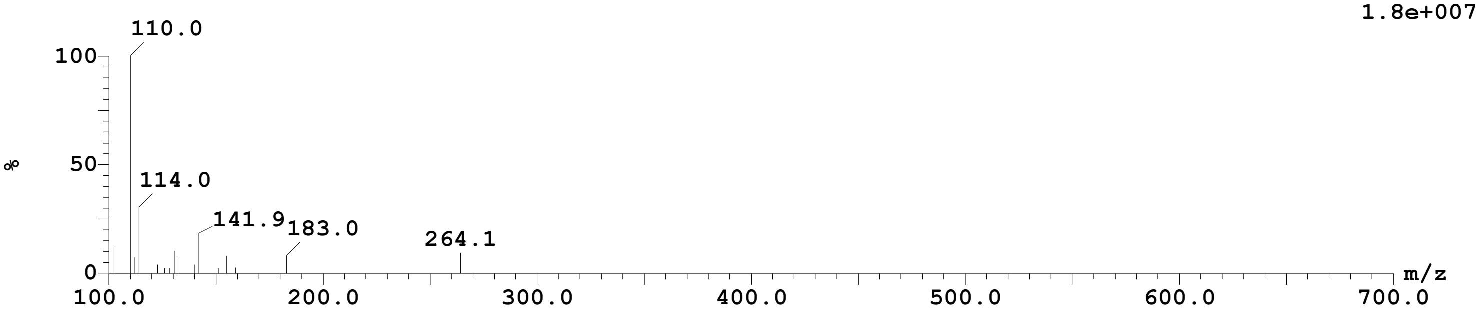

Peak ID	Time
2	0.39

2:(Time: 0.39) Combine (141:156-(54:61+252:259)) 1:MS ES+ 8.6e+007

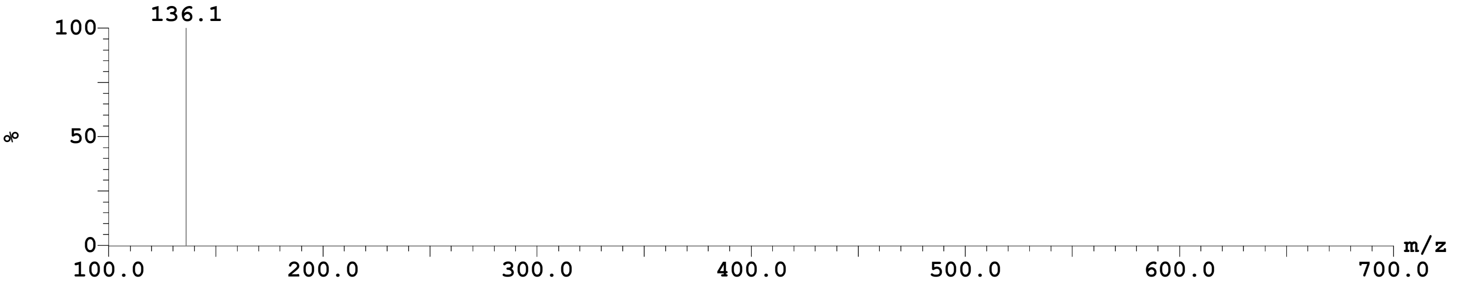

Peak ID	Time
3	0.59

3:(Time: 0.61) Combine (220:235-(138:145+319:326)) 1:MS ES+ 1.6e+008

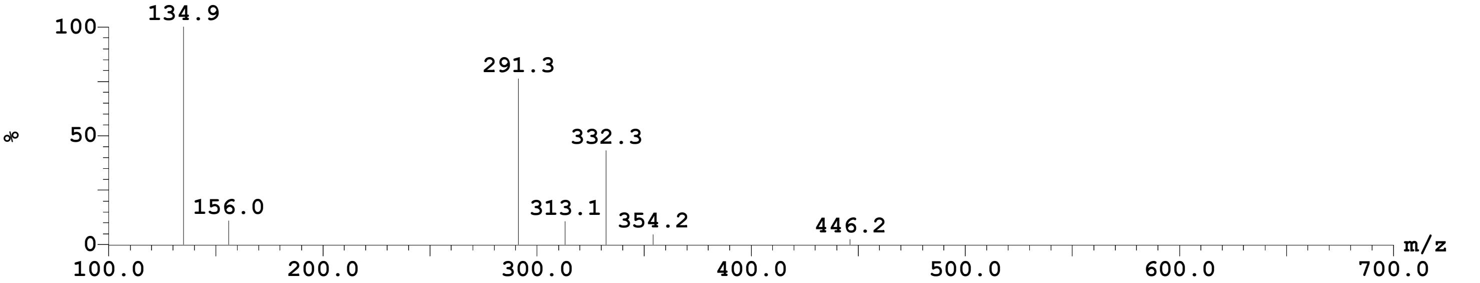

Peak ID	Time
3	0.59

3:(Time: 0.59) Combine (215:229-(133:140+326:333)) 2:MS ES- 5.1e+006

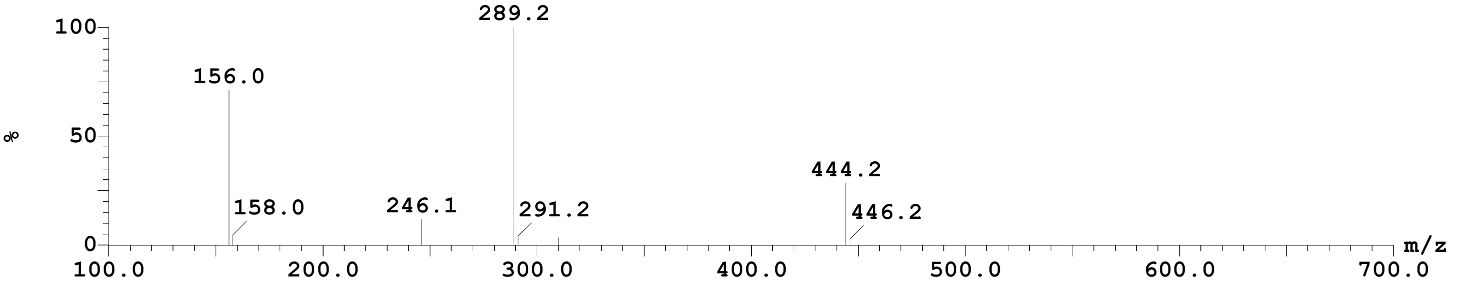

File:13zp581l1 Vial:5:53 ID: **D7**

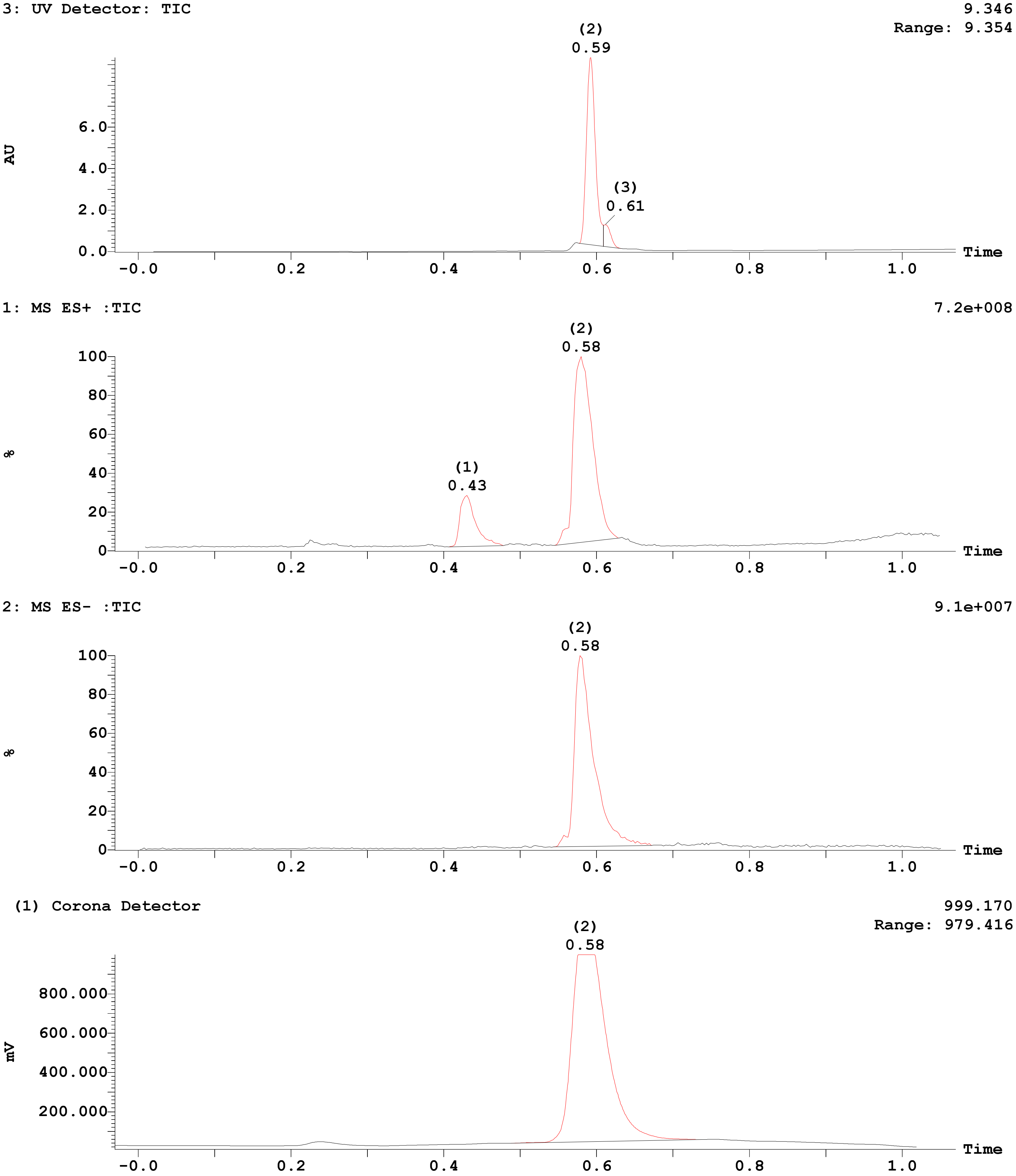

File:13zp581l1 Vial:5:53 ID: D7
Method:C:\MASSLYNX\1minLC_MS.olp

Peak ID **Time**
1 0.43

1:(Time: 0.43) Combine (155:170-(71:78+255:262)) 1:MS ES+ 4.2e+007

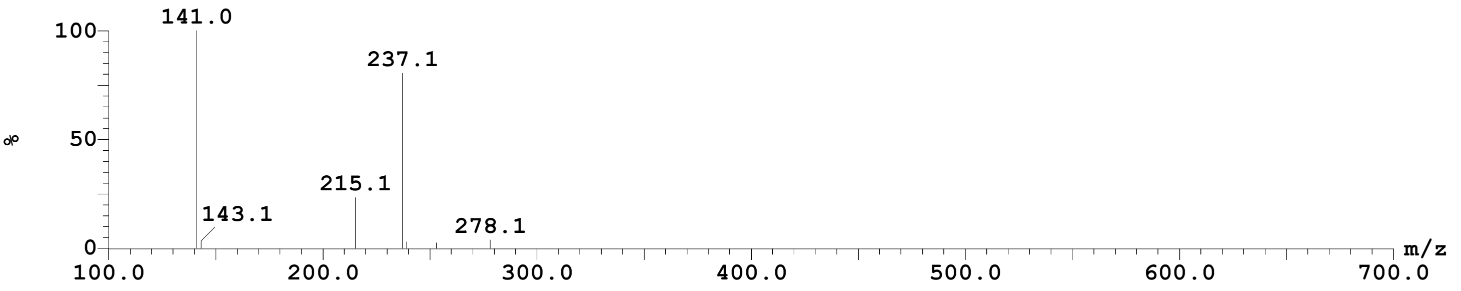

Peak ID **Time**
2 0.58

2:(Time: 0.59) Combine (215:230-(134:142+304:312)) 1:MS ES+ 9.7e+007

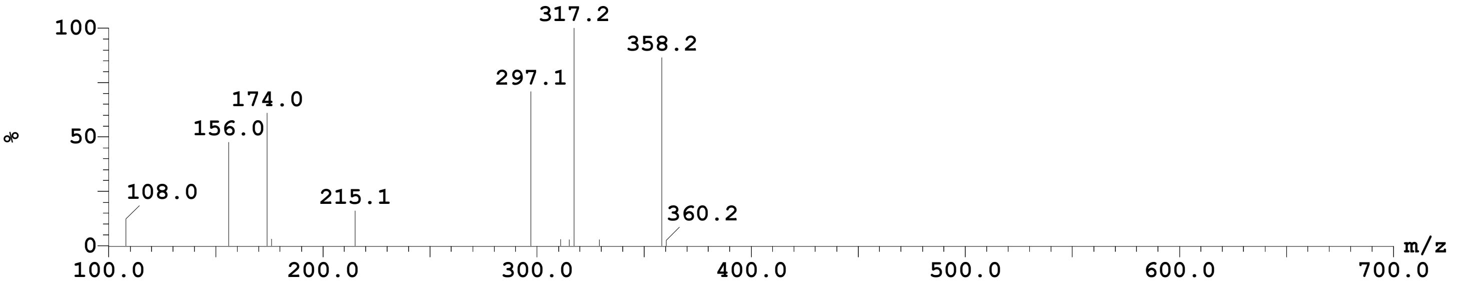

Peak ID **Time**
2 0.58

2:(Time: 0.58) Combine (210:225-(122:129+327:334)) 2:MS ES- 3.2e+007

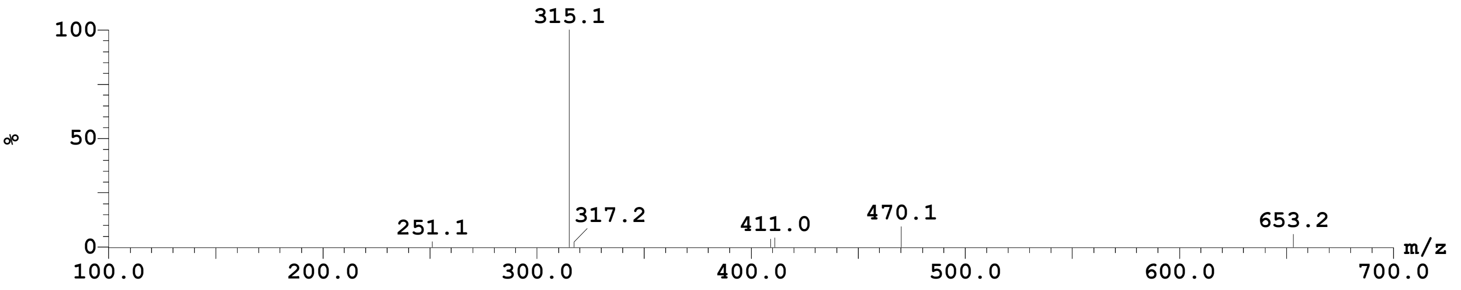

Peak ID **Time**
3 0.61

3:(Time: 0.61) Combine (222:237-(147:154+313:320)) 1:MS ES+ 3.8e+007

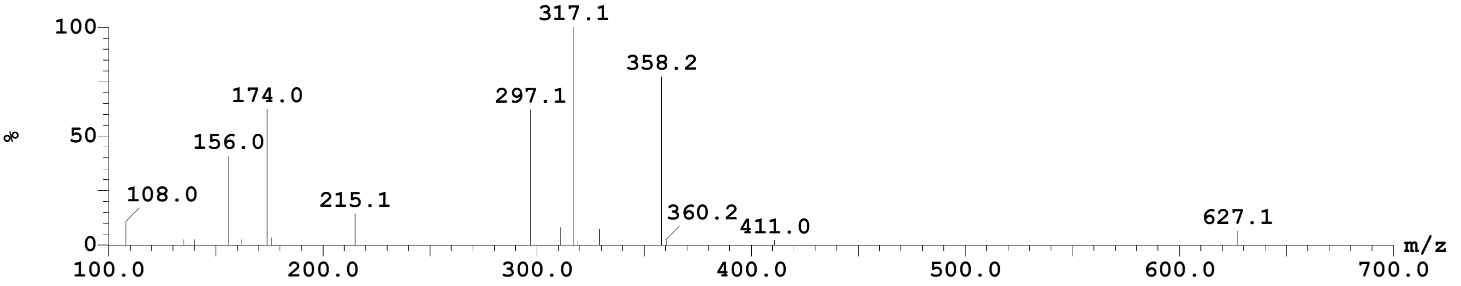

File:13zp581l1 Vial:5:53 ID:D7
Method:C:\MASSLYNX\1minLC_MS.olp

Peak ID	Time
3	0.61

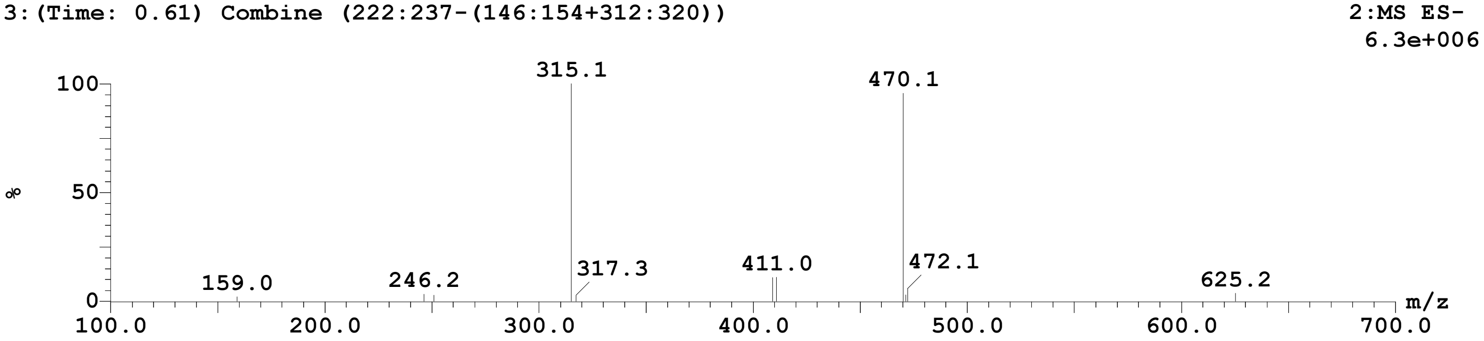

SYNGENTA SQD OPEN ACCESS LC-MS Page 1
File:13zp582l1 Vial:5:50 ID: **D8**

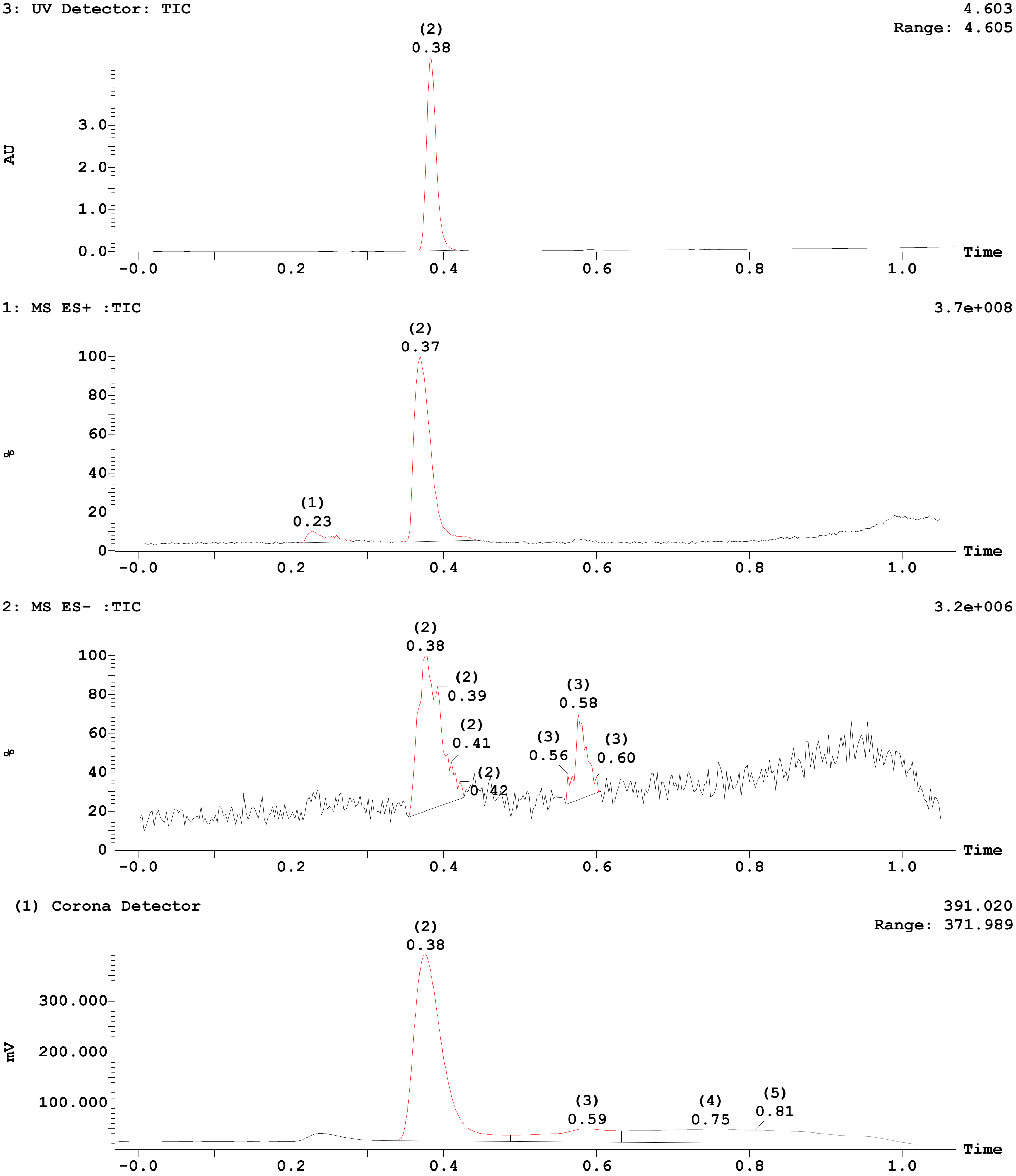

File:13zp582l1 Vial:5:50 ID:D8
Method:C:\MASSLYNX\1minLC_MS.olp

Peak ID Time
1 0.23

1:(Time: 0.23) Combine (78:93-(1:5+181:188)) 1:MS ES+ 3.9e+006

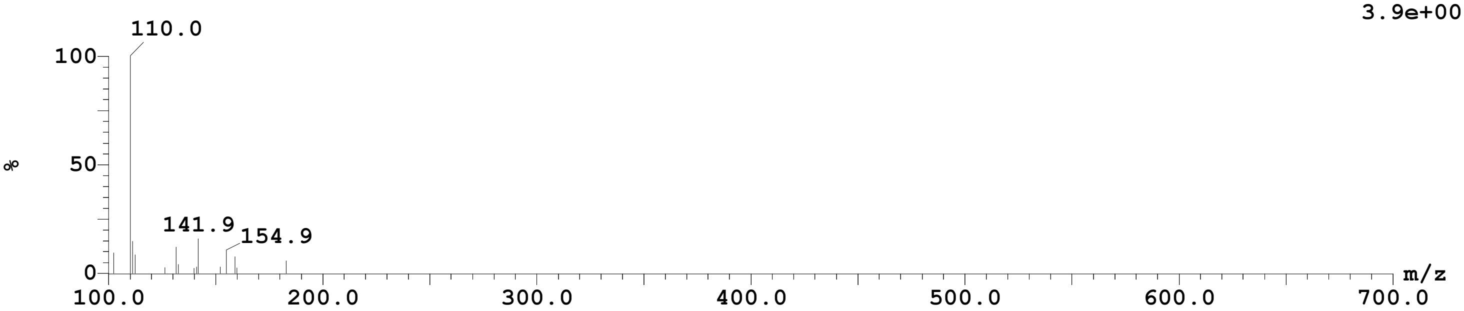

Peak ID Time
2 0.37

2:(Time: 0.38) Combine (137:152-(55:62+233:240)) 1:MS ES+ 6.9e+007

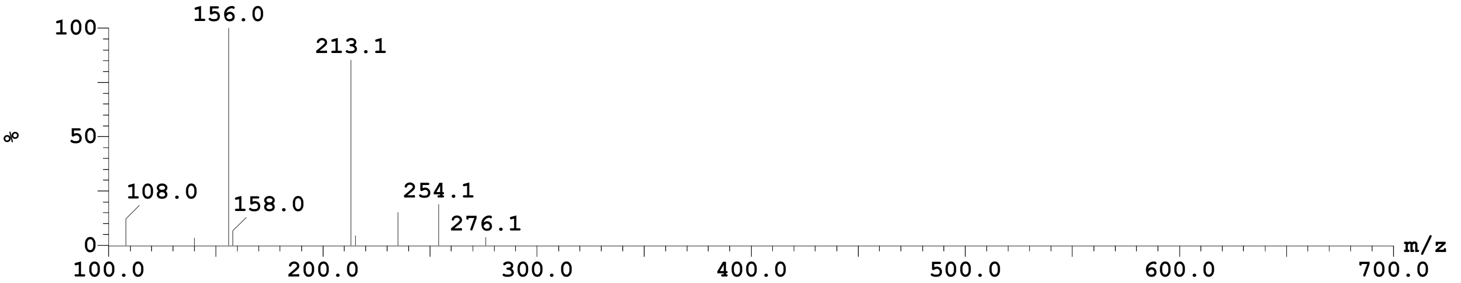

Peak ID Time
2 0.37

2:(Time: 0.38) Combine (136:151-(54:62+232:240)) 2:MS ES- 1.4e+005

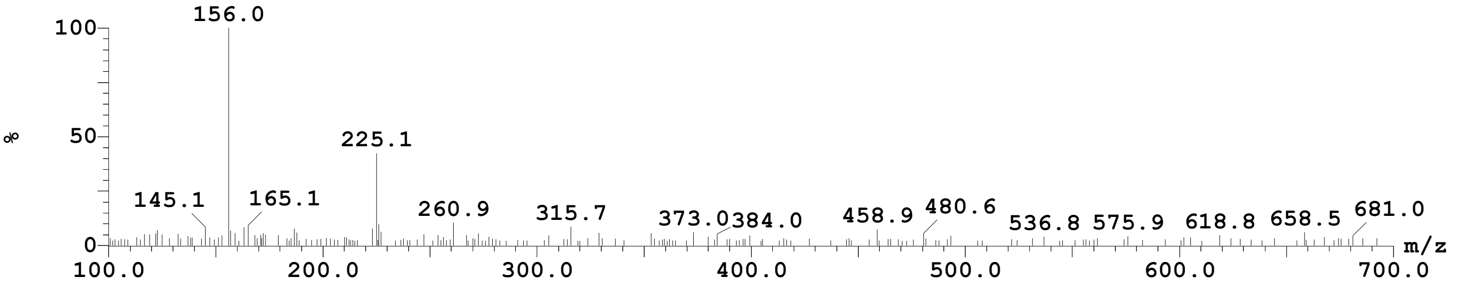

Peak ID Time
3 0.58

3:(Time: 0.58) Combine (208:223-(128:135+302:309)) 2:MS ES- 3.9e+005

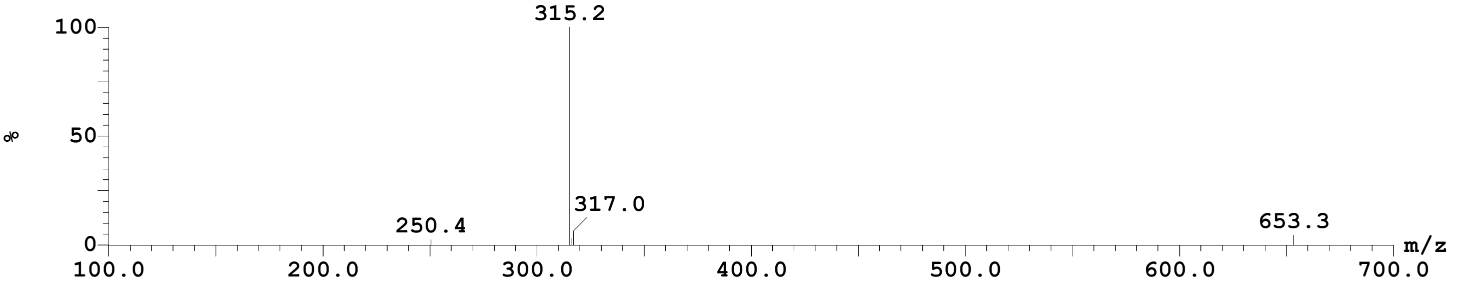

File:13zp583l1 Vial:5:51 ID: **D9**

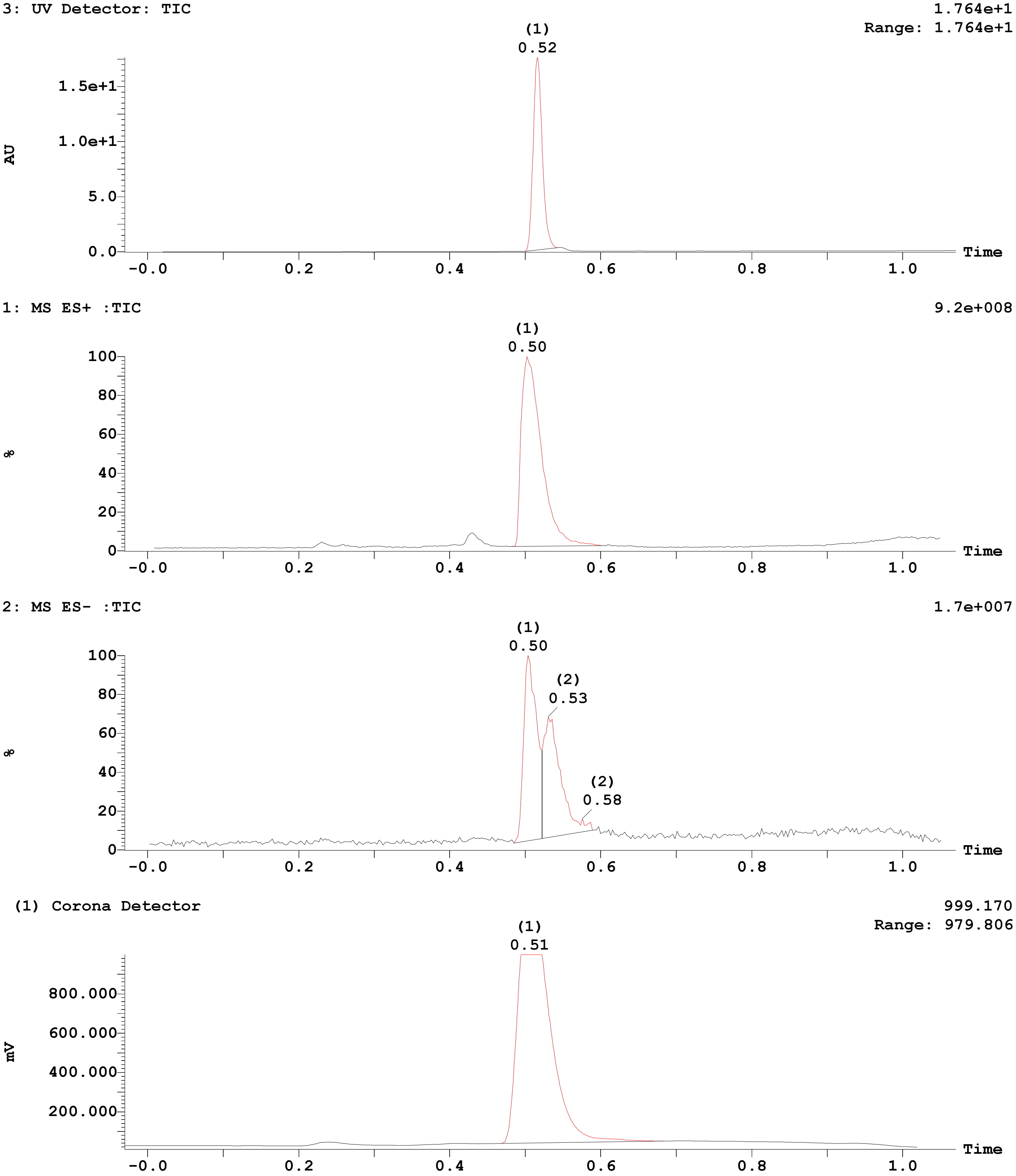

File:13zp583l1 Vial:5:51 ID:D9
Method:C:\MASSLYNX\1minLC_MS.olp

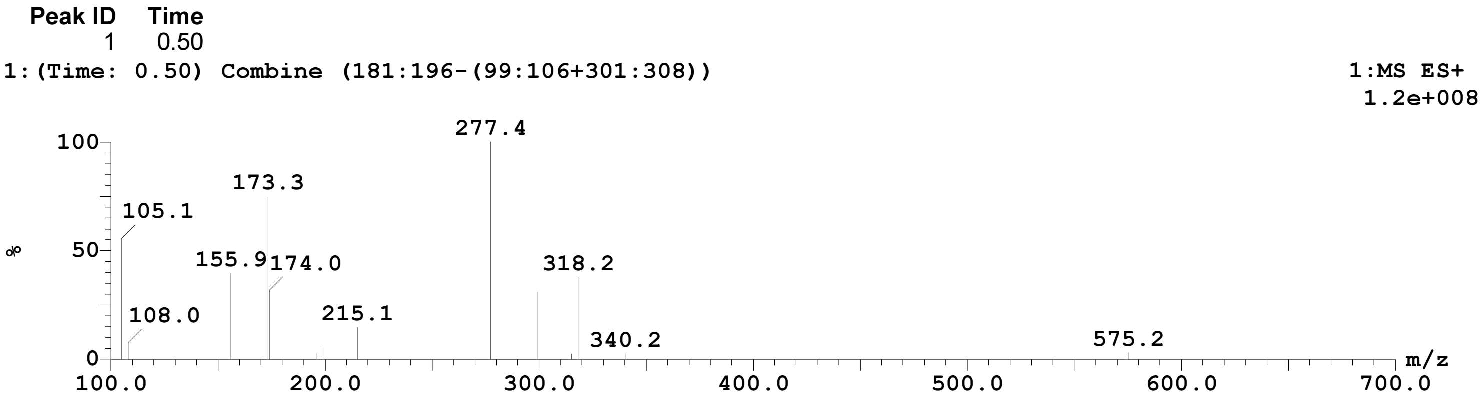

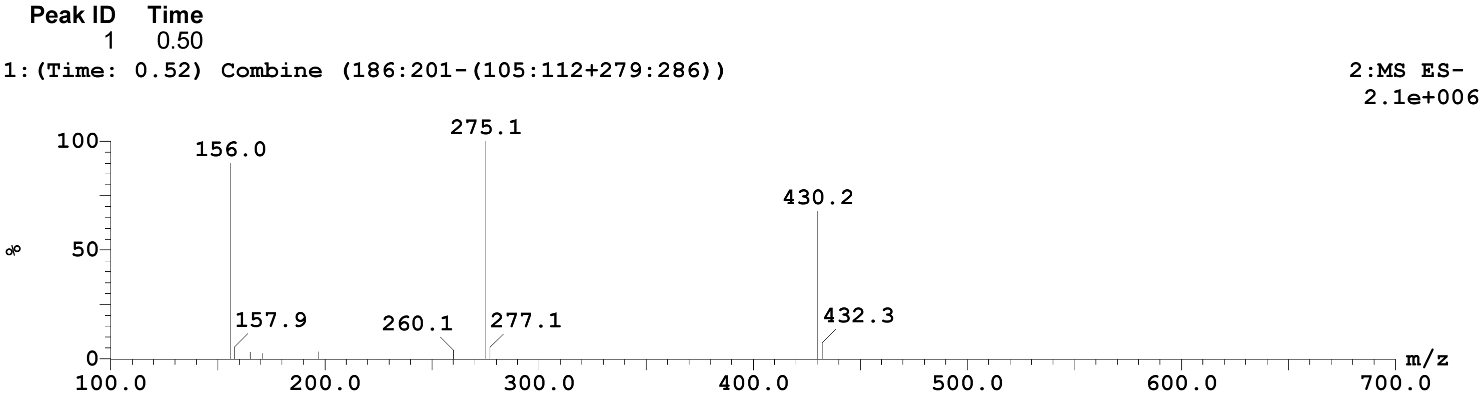

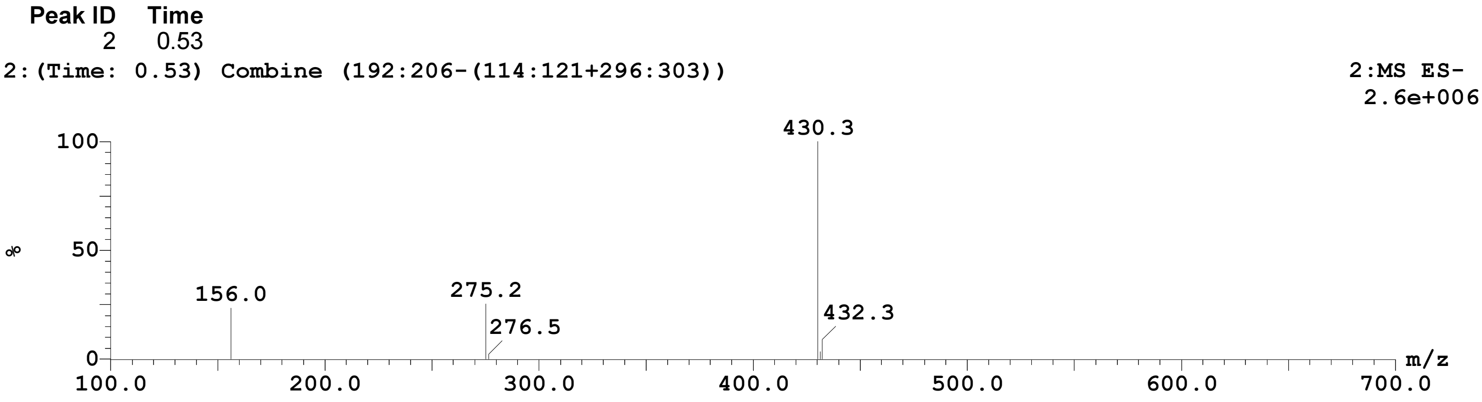

File:13zp584l1 Vial:5:52 ID: **D10**

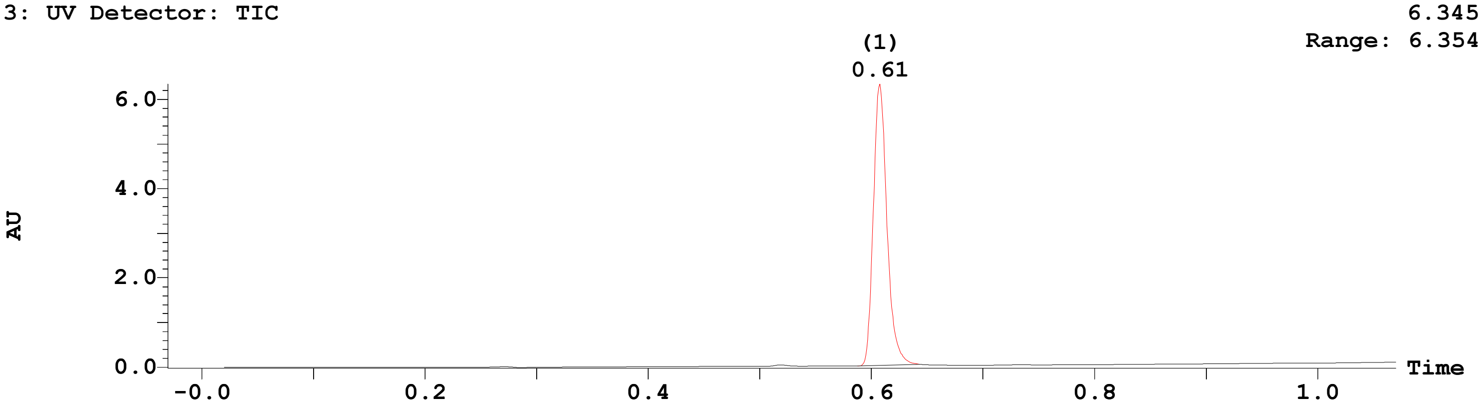

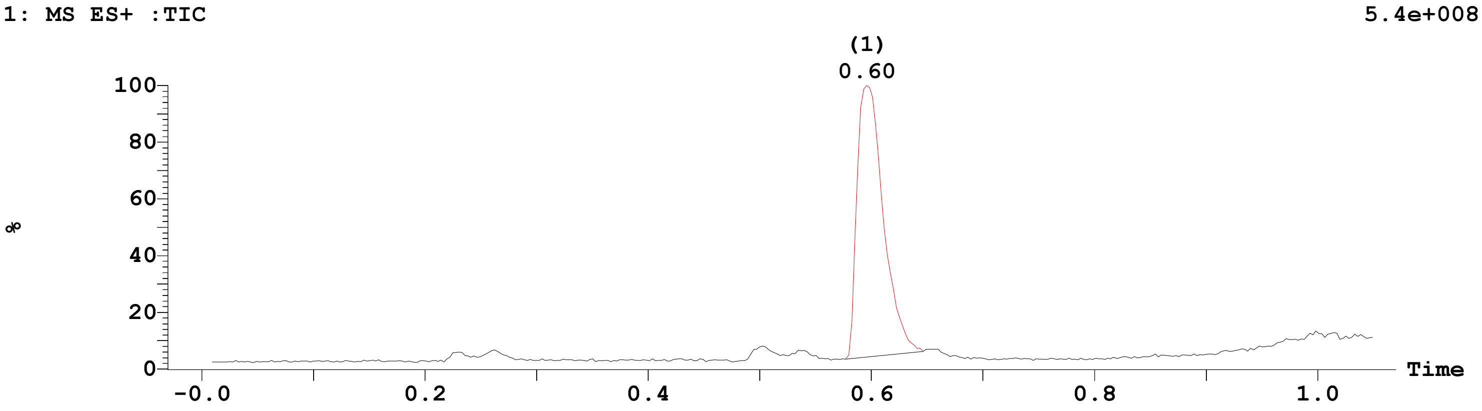

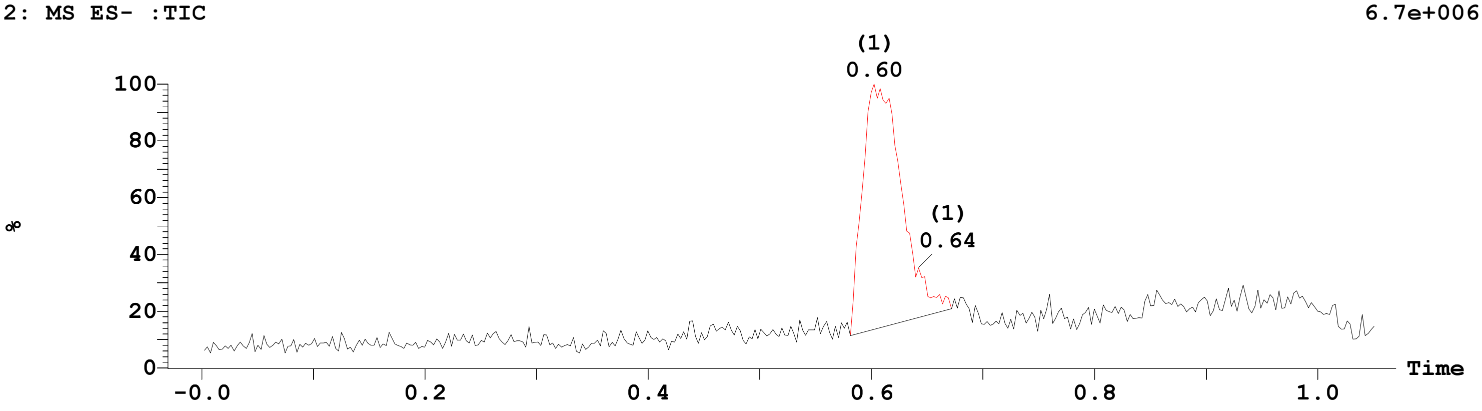

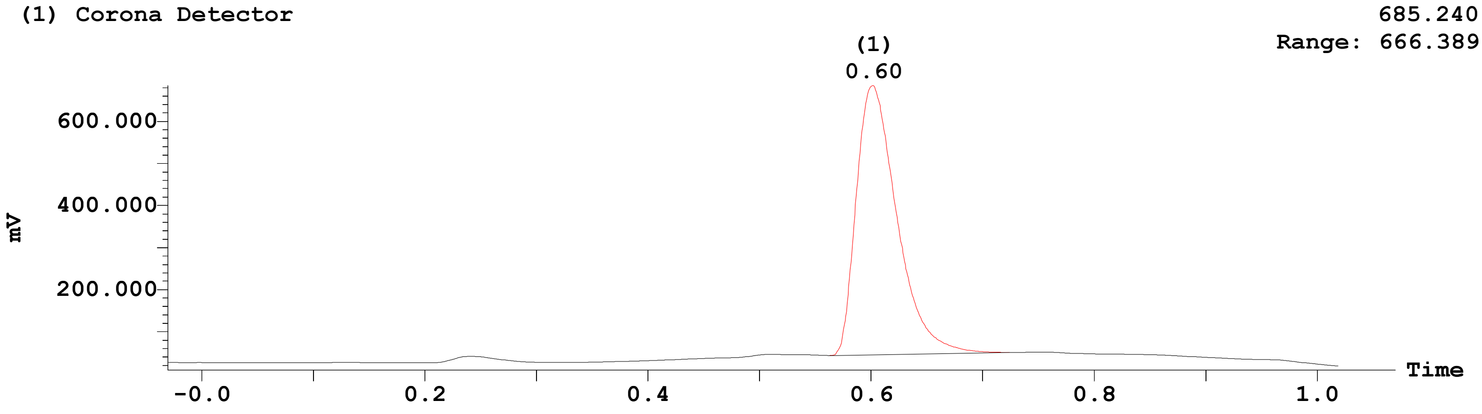

File:13zp584l1 Vial:5:52 ID:D10
Method:C:\MASSLYNX\1minLC_MS.olp

Peak ID	Time
1	0.60

1:(Time: 0.60) Combine (217:231-(135:142+318:325)) 1:MS ES+ 1.5e+008

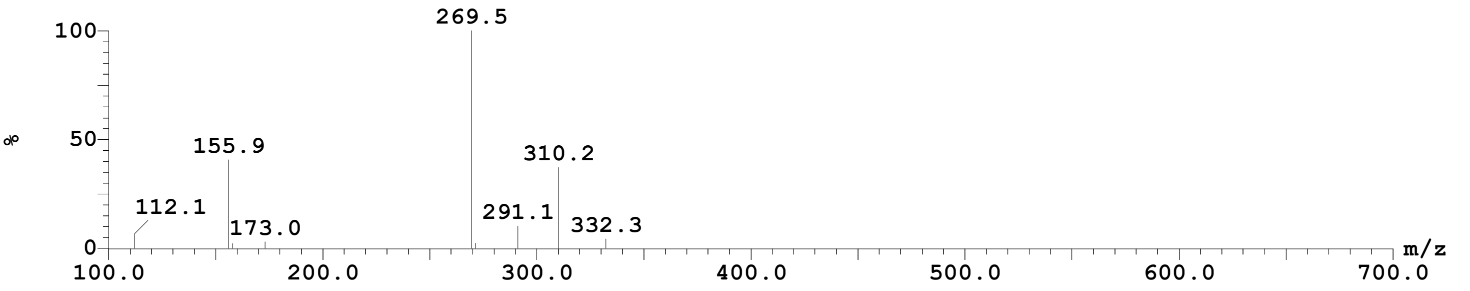

Peak ID	Time
1	0.60

1:(Time: 0.60) Combine (219:234-(136:143+327:334)) 2:MS ES- 3.1e+005

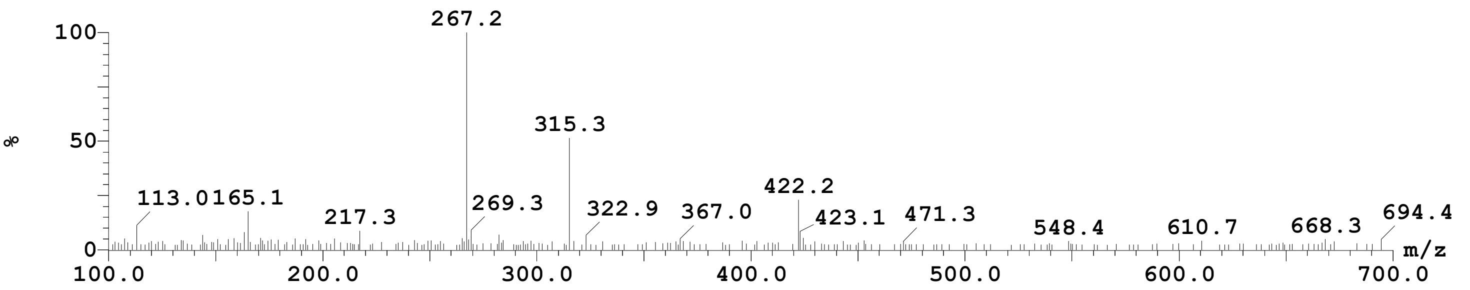

SYNGENTA SQD OPEN ACCESS LC-MS Page 1
File:13zo147l2 Vial:5:23 ID: **E2**

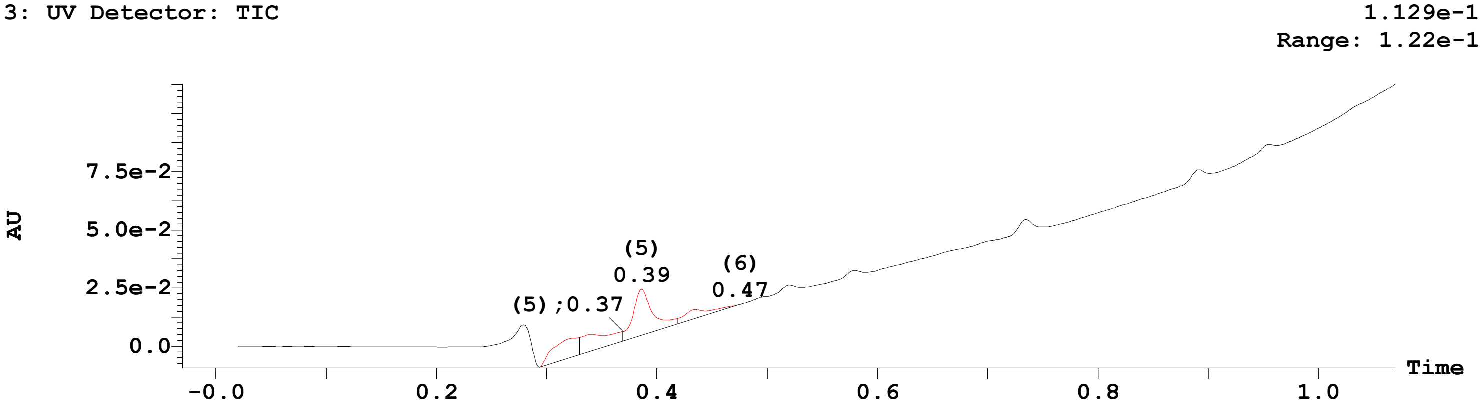

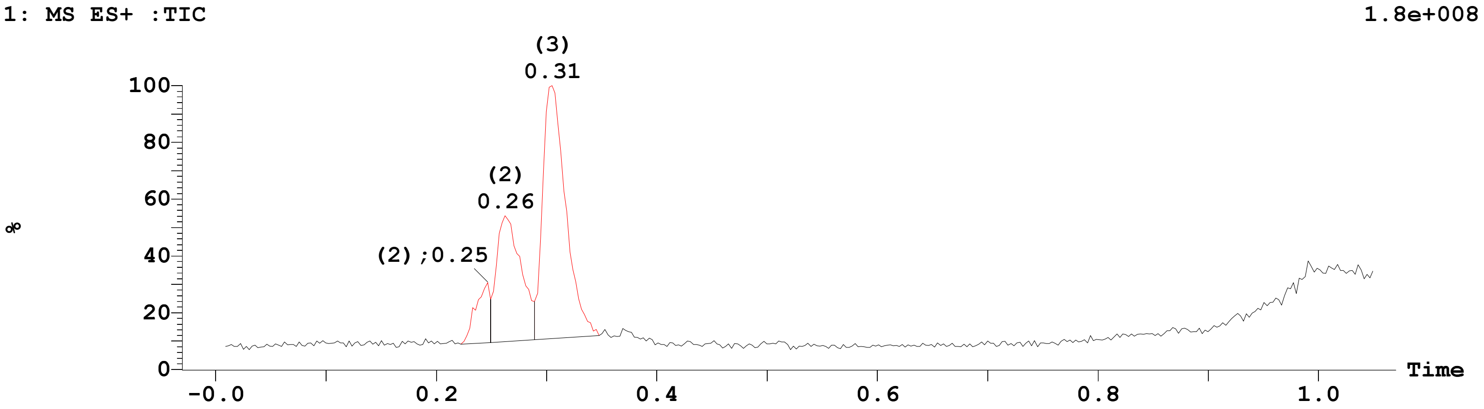

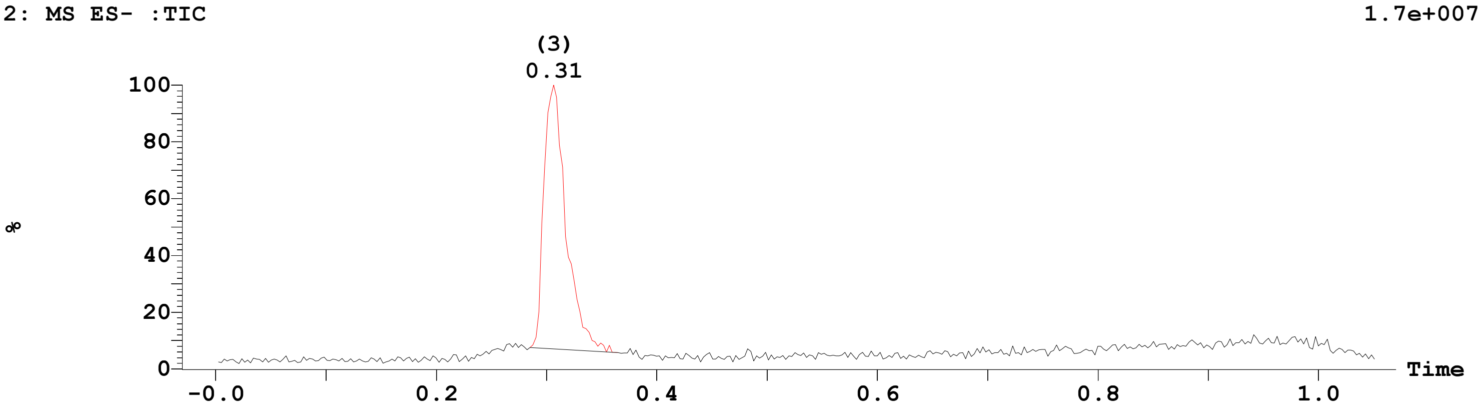

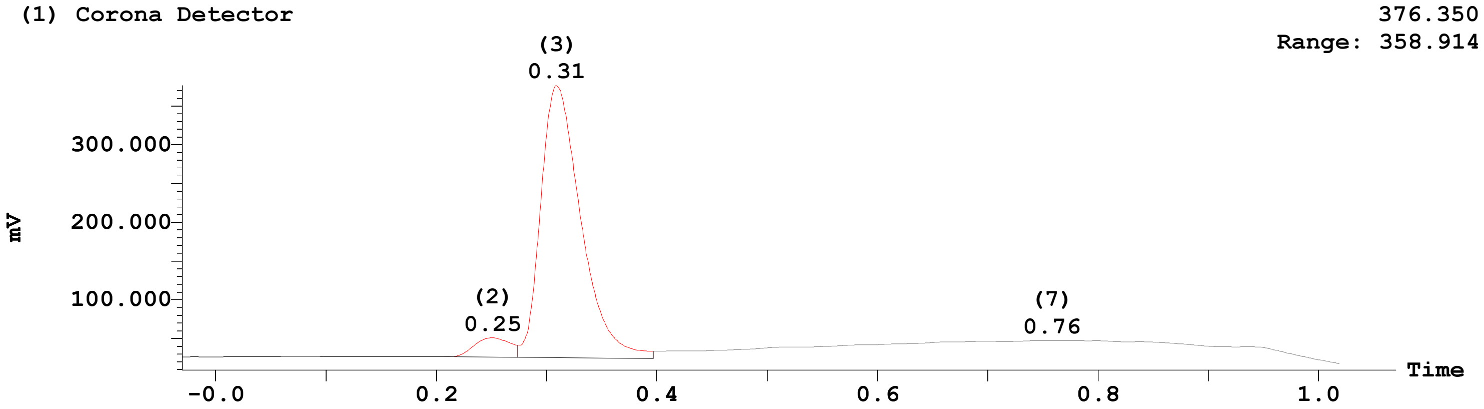

File:13zo147l2 Vial:5:23 ID:E2
Method:C:\MASSLYNX\1minLC_MS.olp

Peak ID **Time**
1 0.25

1:(Time: 0.25) Combine (86:101-(2:9+169:176)) 1:MS ES+
1.1e+007

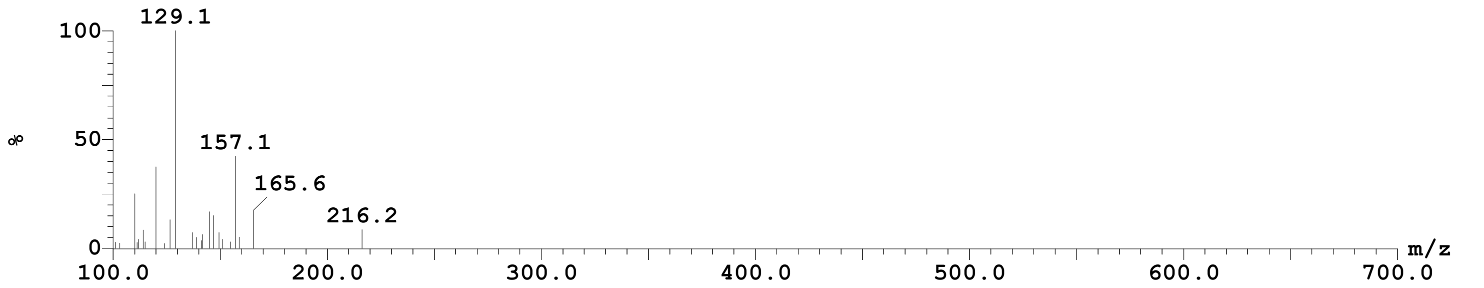

Peak ID **Time**
2 0.26

2:(Time: 0.26) Combine (92:107-(12:19+184:191)) 1:MS ES+
9.3e+006

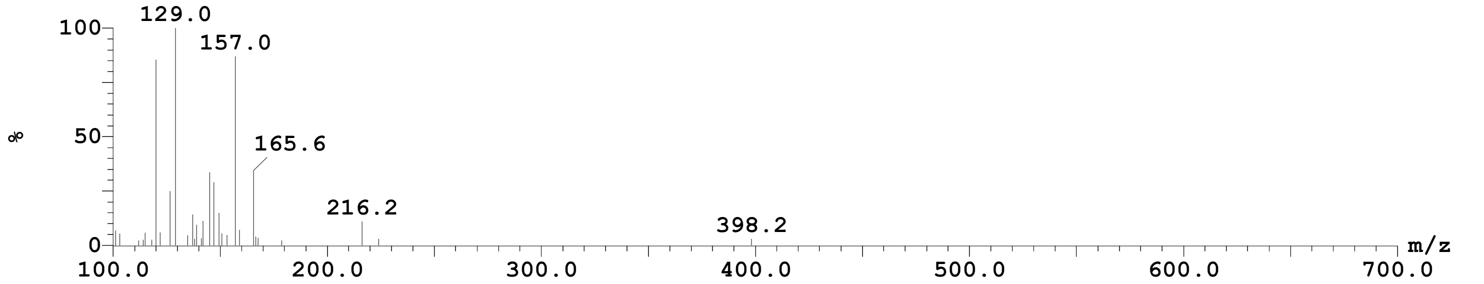

Peak ID **Time**
3 0.31

3:(Time: 0.31) Combine (107:122-(27:34+206:213)) 1:MS ES+
4.6e+007

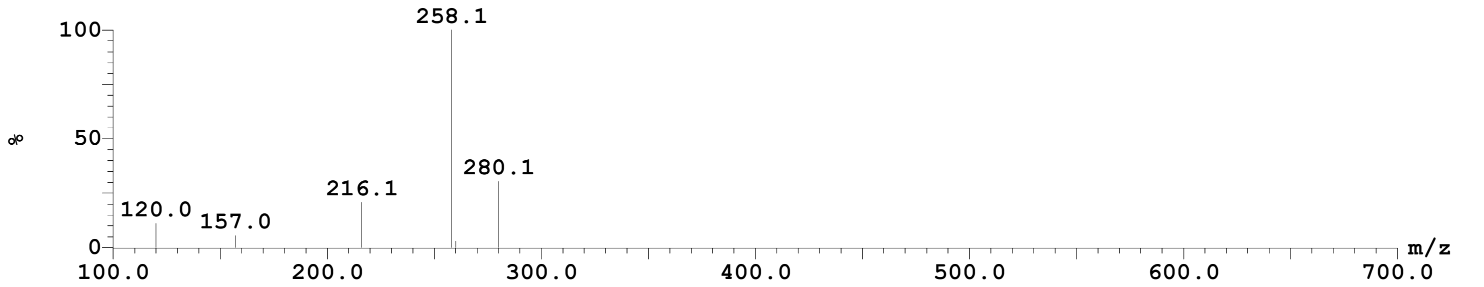

Peak ID **Time**
3 0.31

3:(Time: 0.31) Combine (107:122-(25:32+212:219)) 2:MS ES-
3.9e+006

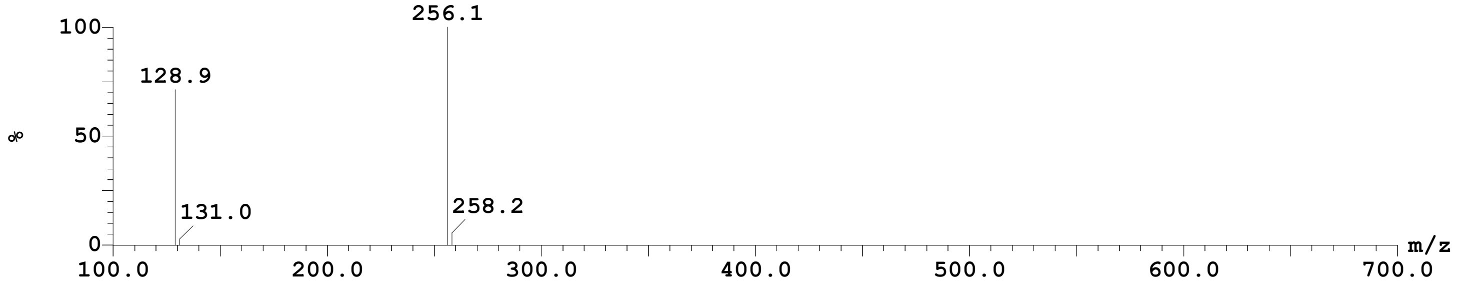

File:13zo147l2 Vial:5:23 ID:E2
Method:C:\MASSLYNX\1minLC_MS.olp

Peak ID **Time**
4 0.37

4:(Time: 0.37) Combine (132:147-(42:49+214:222)) 1:MS ES+ 2.2e+006

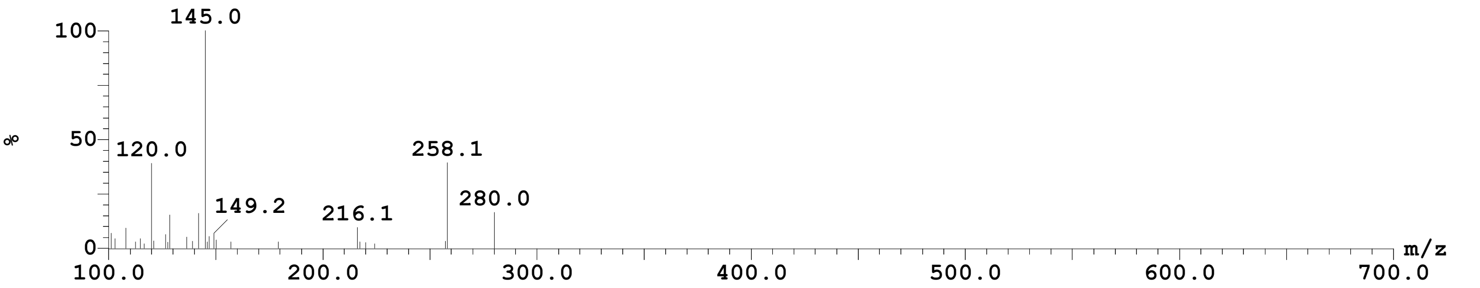

Peak ID **Time**
4 0.37

4:(Time: 0.37) Combine (131:146-(41:49+214:221)) 2:MS ES- 8.9e+004

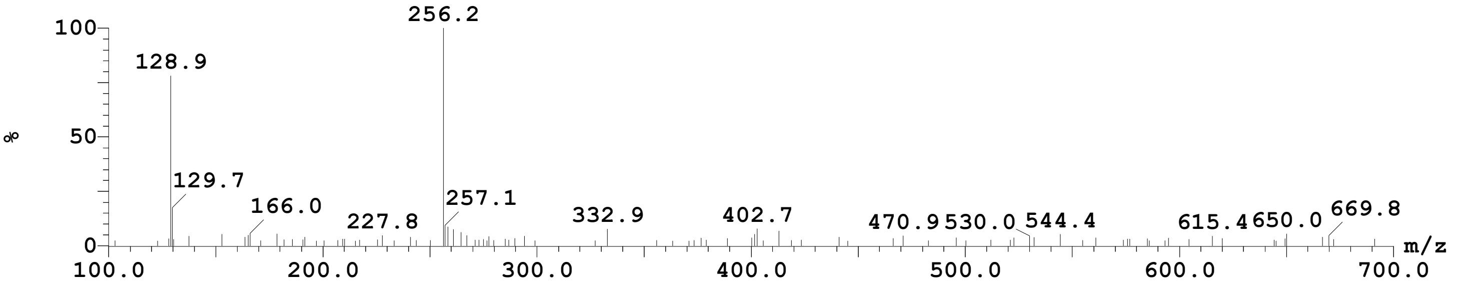

Peak ID **Time**
5 0.39

5:(Time: 0.39) Combine (138:153-(57:64+233:240)) 1:MS ES+ 2.2e+006

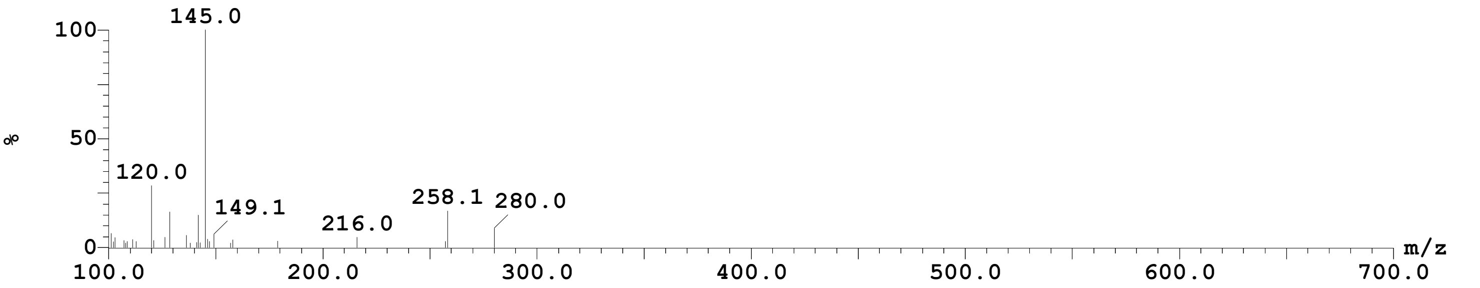

Peak ID **Time**
5 0.39

5:(Time: 0.39) Combine (137:152-(56:64+232:240)) 2:MS ES- 4.0e+004

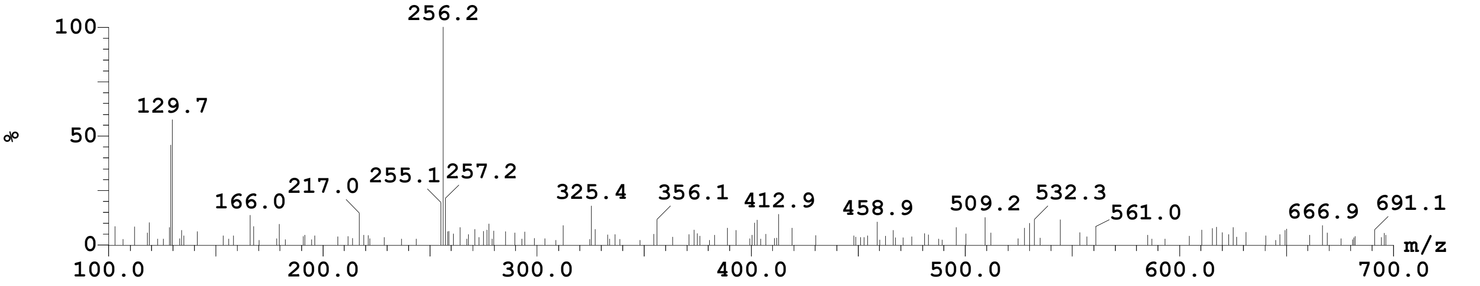

File:13zo147l2 Vial:5:23 ID:E2
Method:C:\MASSLYNX\1minLC_MS.olp

Peak ID **Time**
6 0.47

6:(Time: 0.47) Combine (171:186-(75:83+254:261)) 1:MS ES+ 3.2e+005

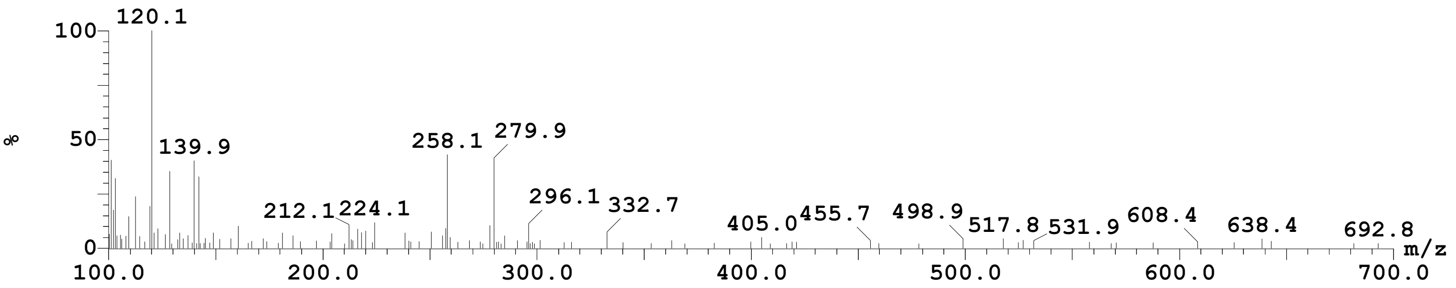

Peak ID **Time**
6 0.47

6:(Time: 0.47) Combine (171:186-(75:82+253:261)) 2:MS ES- 1.4e+004

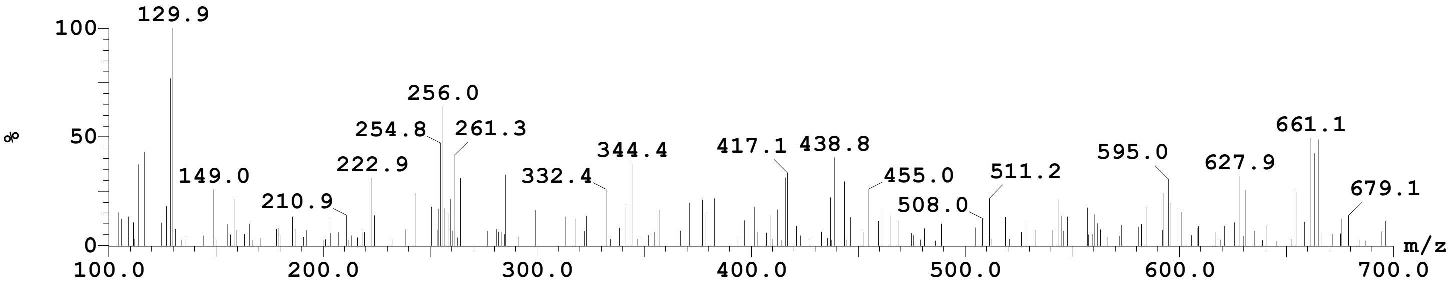

File:13zp820l1 Vial:5:37 ID:**E3**

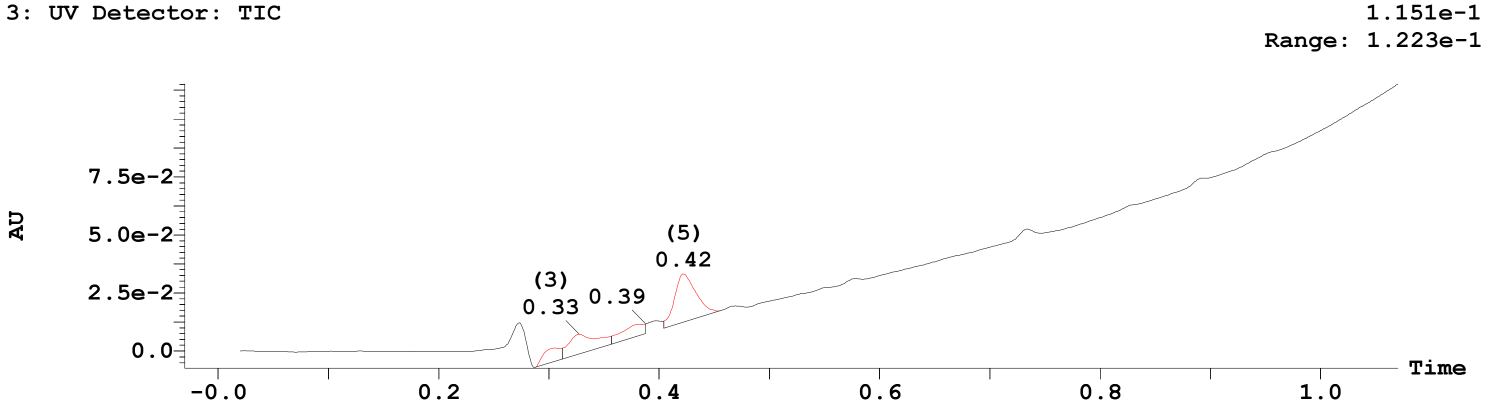

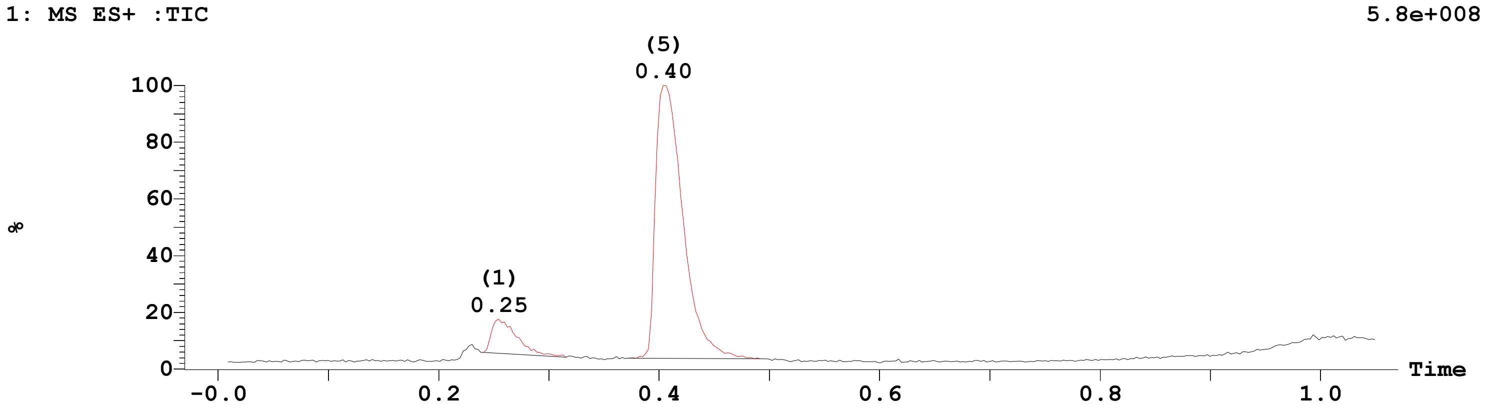

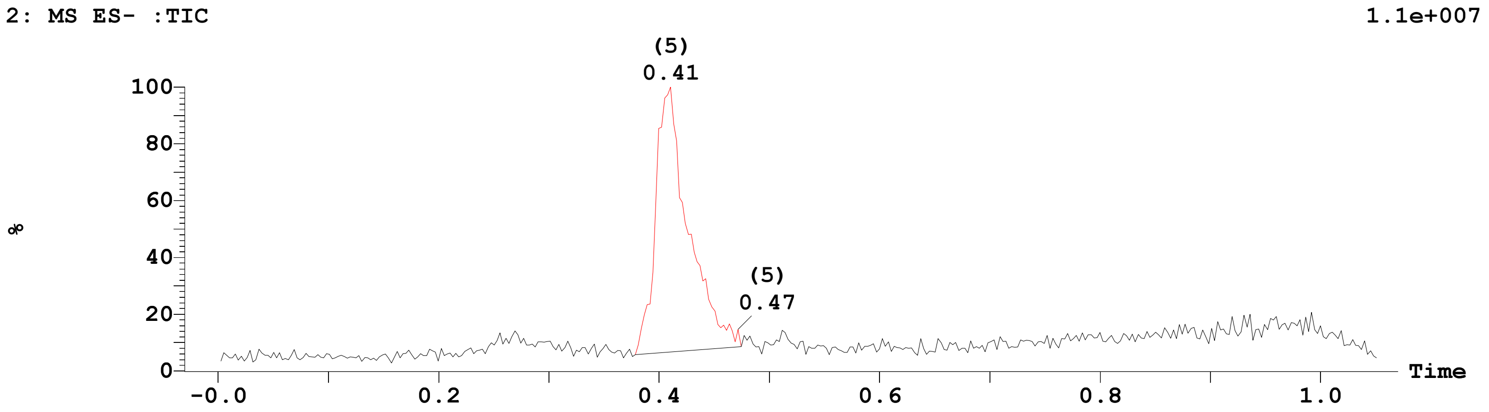

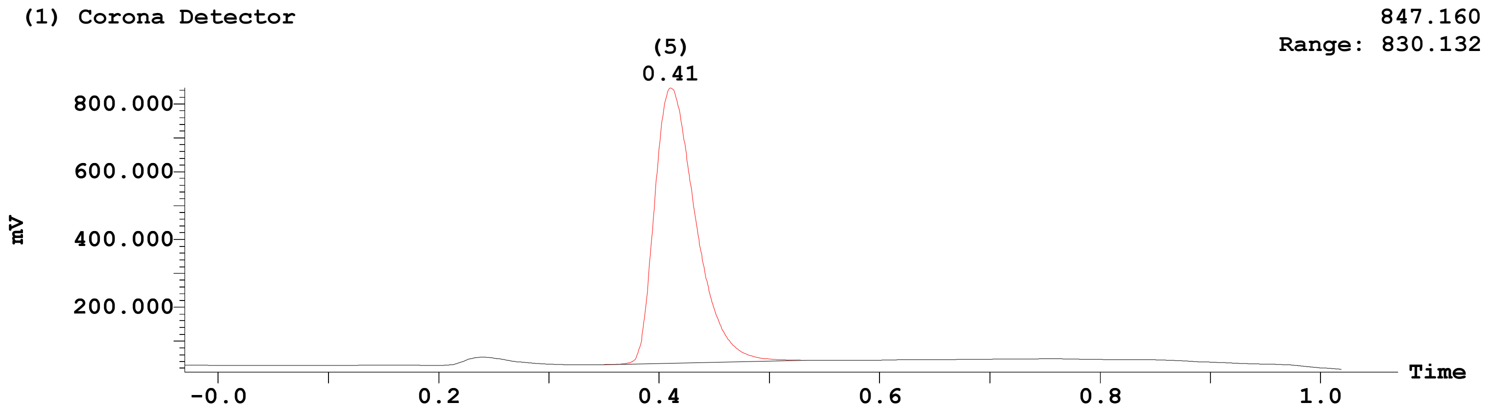

File:13zp820l1 Vial:5:37 ID:E3
Method:C:\MASSLYNX\1minLC_MS.olp

Peak ID **Time**
1 0.25

1:(Time: 0.25) Combine (89:103-(9:16+194:201)) 1:MS ES+
8.9e+006

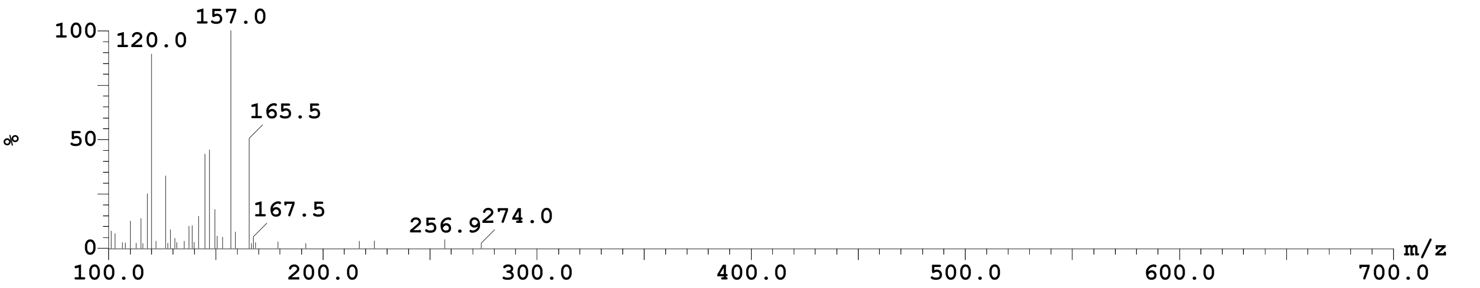

Peak ID **Time**
2 0.31

2:(Time: 0.31) Combine (107:122-(26:34+193:200)) 1:MS ES+
3.8e+006

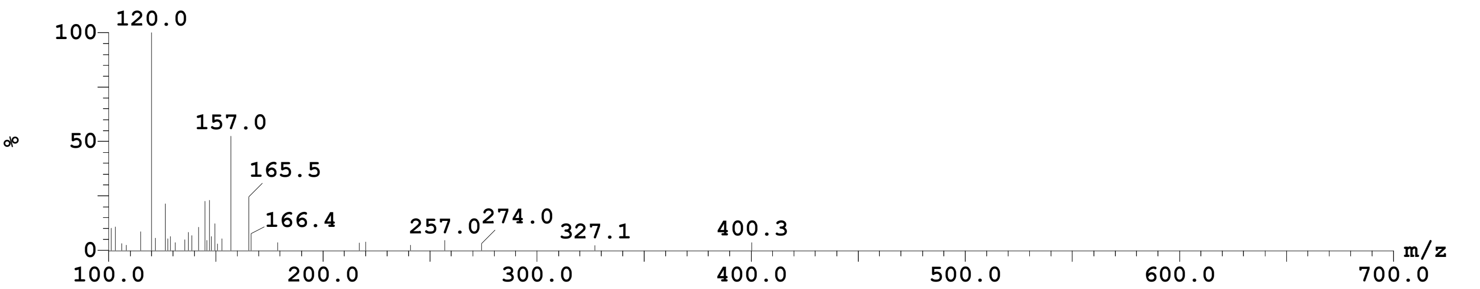

Peak ID **Time**
2 0.31

2:(Time: 0.31) Combine (107:122-(26:33+192:200)) 2:MS ES-
7.8e+003

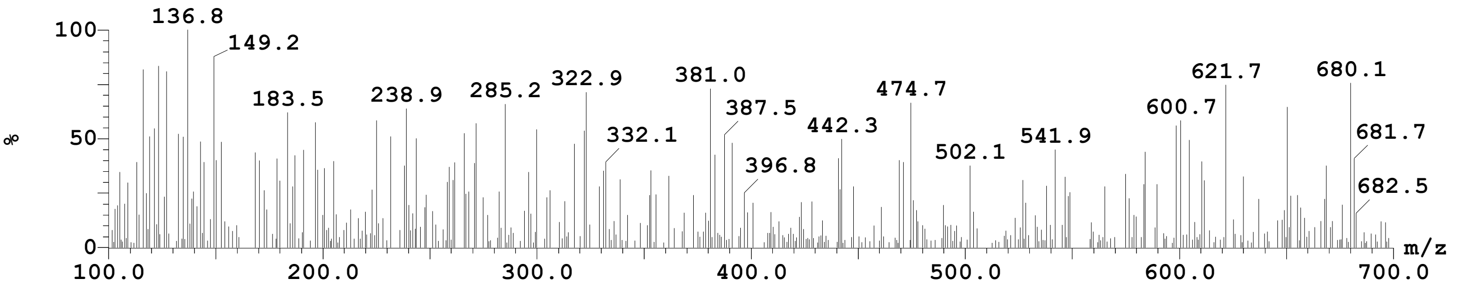

Peak ID **Time**
3 0.33

3:(Time: 0.33) Combine (116:131-(35:43+209:217)) 1:MS ES+
2.3e+006

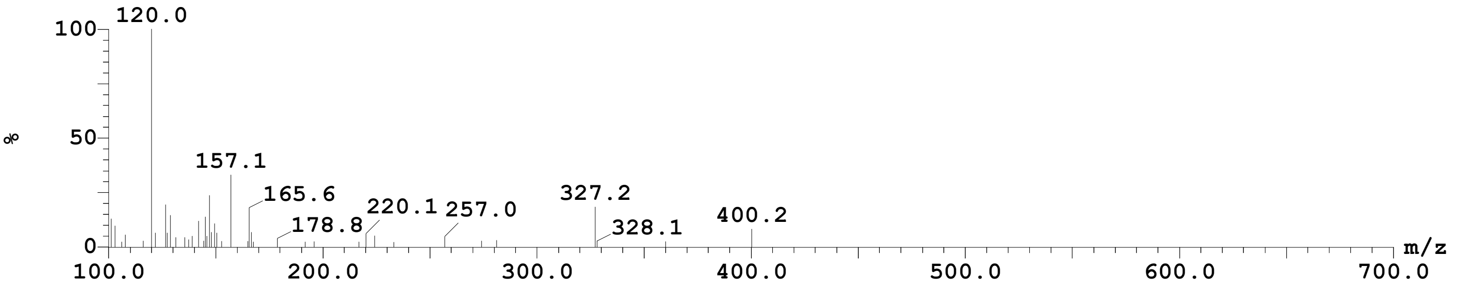

File:13zp820l1 Vial:5:37 ID:E3
Method:C:\MASSLYNX\1minLC_MS.olp

Peak ID **Time**
3 0.33

3:(Time: 0.33) Combine (115:130-(35:42+209:216)) 2:MS ES-
9.6e+003

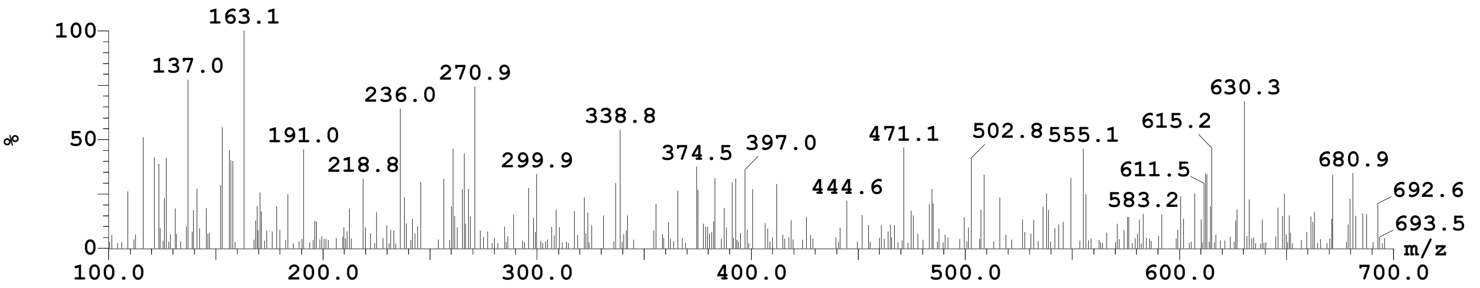

Peak ID **Time**
4 0.39

4:(Time: 0.39) Combine (138:153-(52:59+221:228)) 1:MS ES+
4.3e+007

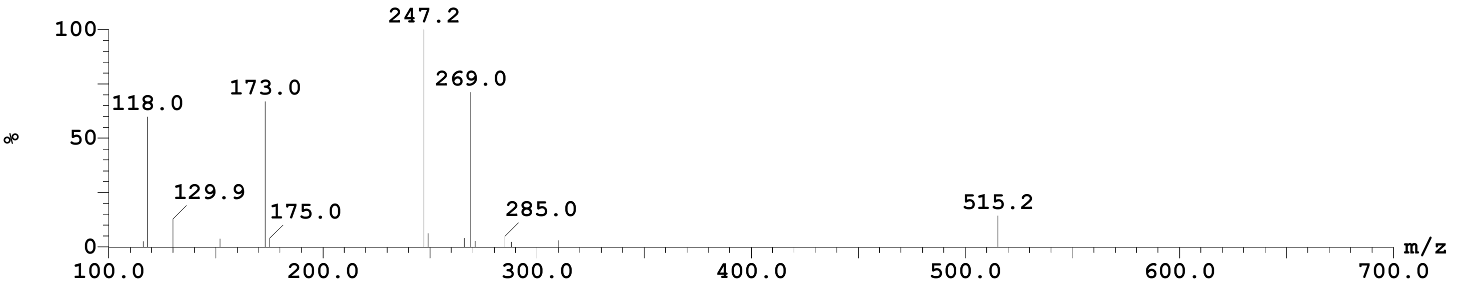

Peak ID **Time**
4 0.39

4:(Time: 0.39) Combine (138:153-(51:59+220:228)) 2:MS ES-
1.7e+006

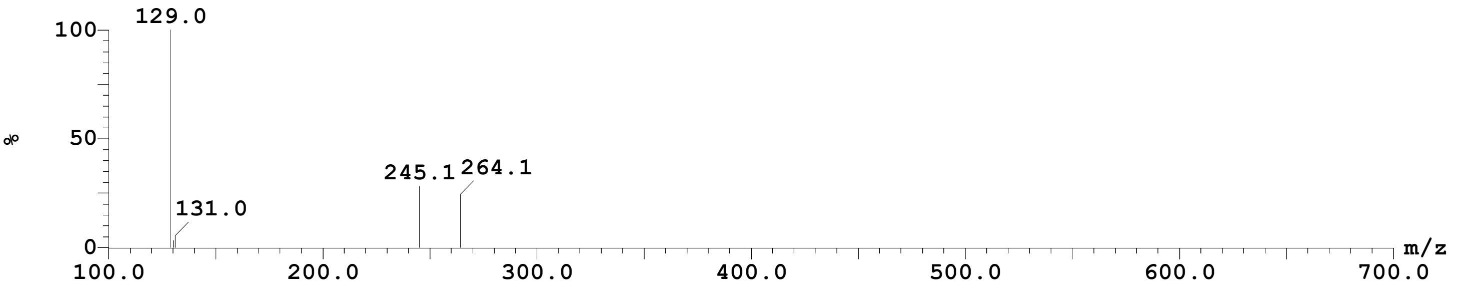

Peak ID **Time**
5 0.40

5:(Time: 0.40) Combine (144:160-(58:65+260:267)) 1:MS ES+
8.4e+007

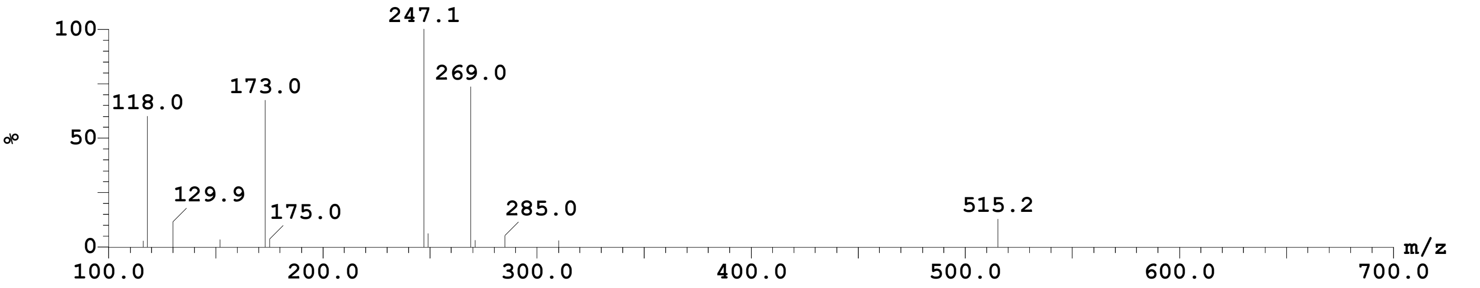

File:13zp820l1 Vial:5:37 ID:E3
Method:C:\MASSLYNX\1minLC_MS.olp

Peak ID	Time
5	0.40

5: (Time: 0.41) Combine (146:161-(60:67+253:260))

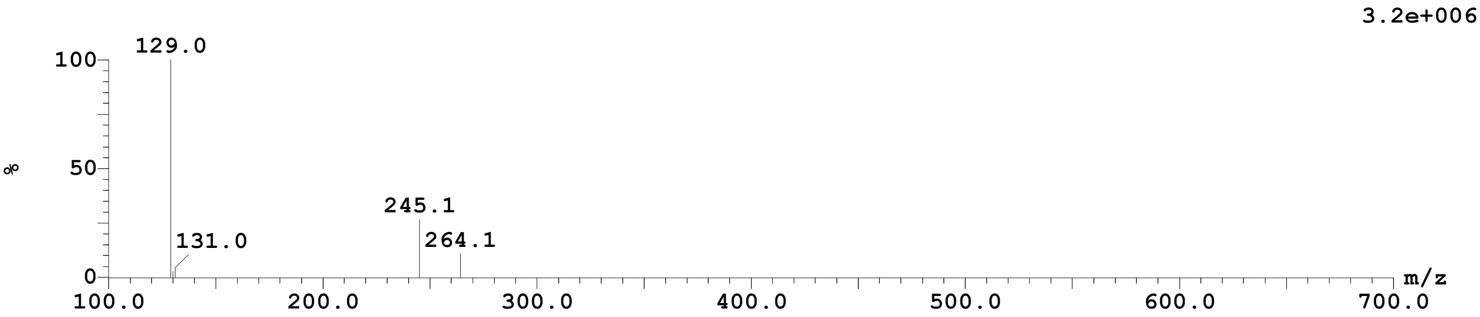

SYNGENTA SQD OPEN ACCESS Page 1

File:13zo178l3 Vial:5:38 ID: **E4**

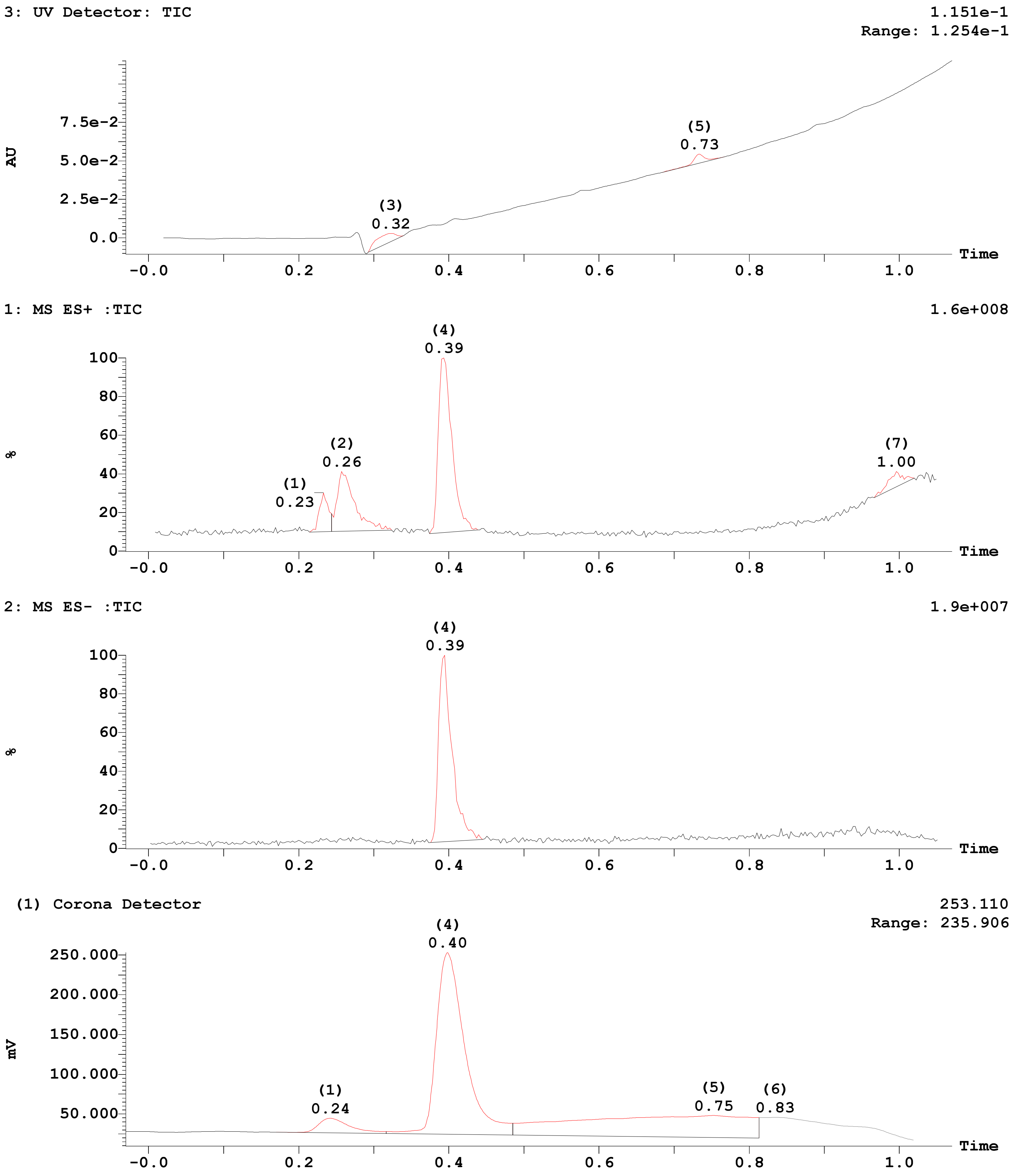

File:13zo178l3 Vial:5:38 ID:E4
Method:C:\MASSLYNX\1minLC_MS.olp

Peak ID **Time**
1 0.23

1:(Time: 0.23) Combine (81:95-(1:6+167:174)) 1:MS ES+

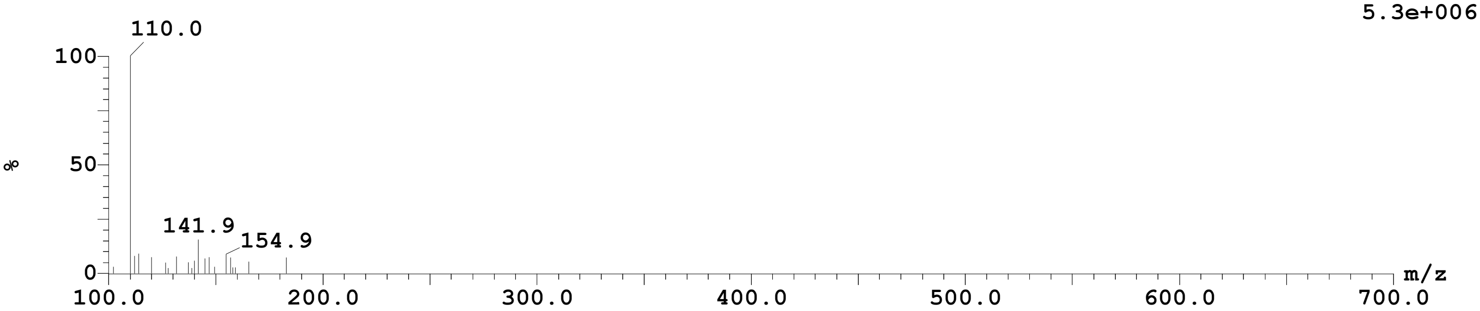

Peak ID **Time**
2 0.26

2:(Time: 0.26) Combine (90:105-(10:17+197:204)) 1:MS ES+

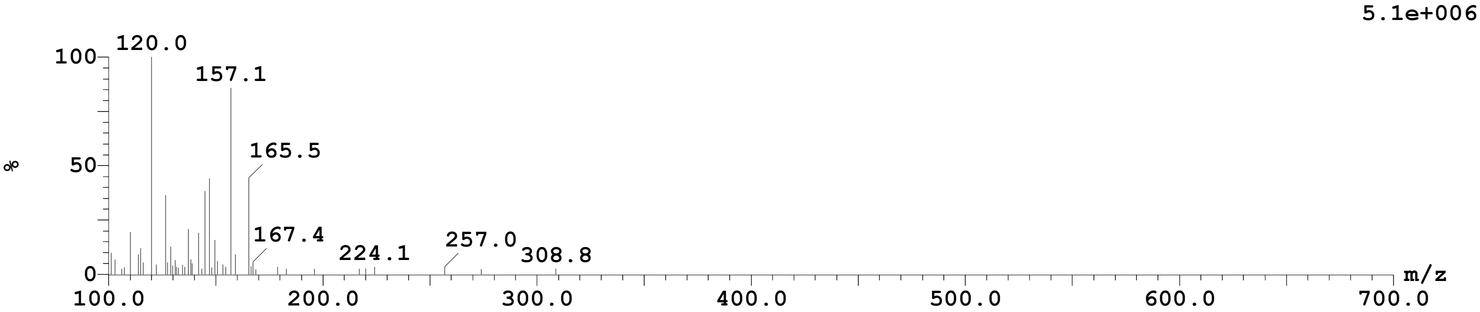

Peak ID **Time**
3 0.32

3:(Time: 0.32) Combine (114:129-(28:35+203:211)) 1:MS ES+

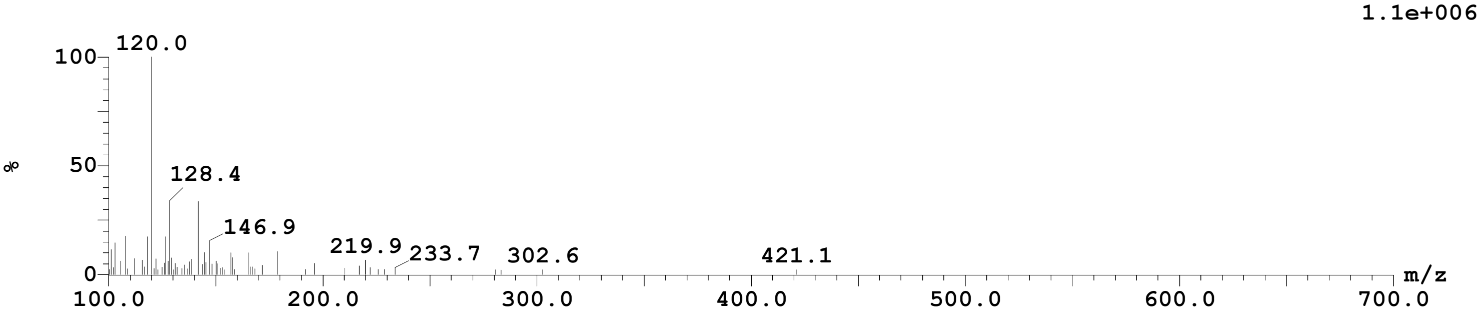

Peak ID **Time**
3 0.32

3:(Time: 0.32) Combine (114:129-(27:35+203:210)) 2:MS ES-

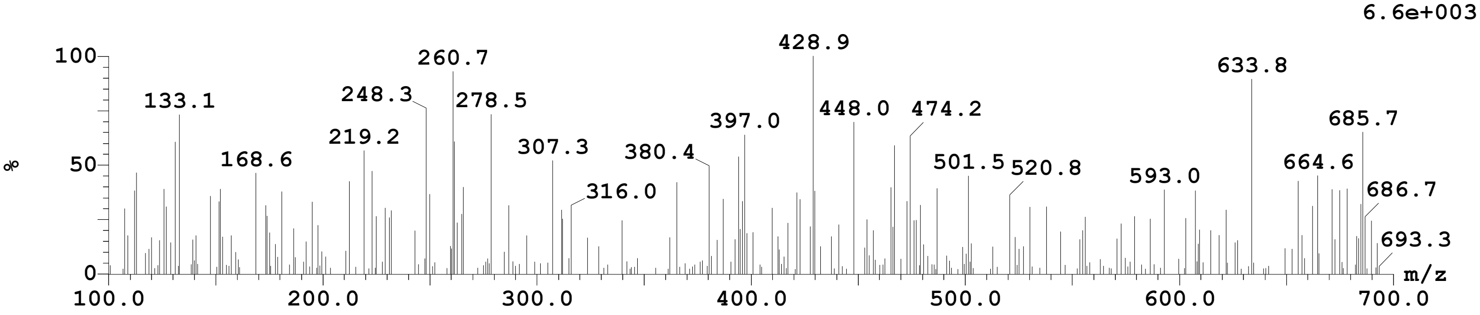

File:13zo178l3 Vial:5:38 ID:E4
Method:C:\MASSLYNX\1minLC_MS.olp

Peak ID **Time**
4 0.39

4:(Time: 0.39) Combine (140:156-(59:66+240:247)) 1:MS ES+ 3.8e+007

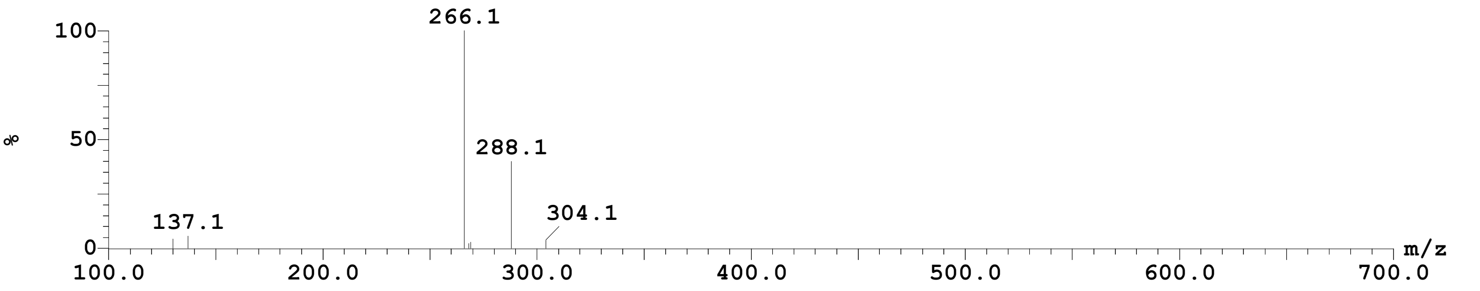

Peak ID **Time**
4 0.39

4:(Time: 0.39) Combine (141:155-(59:66+242:249)) 2:MS ES- 6.0e+006

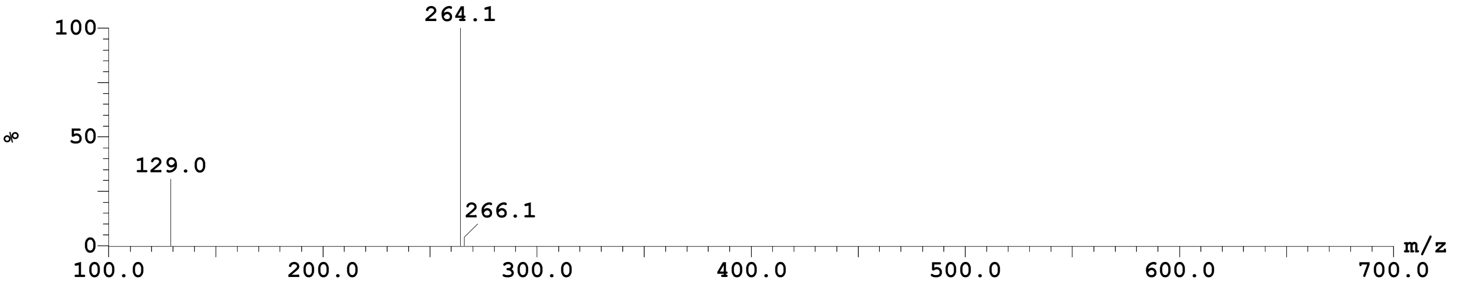

Peak ID **Time**
5 0.73

5:(Time: 0.73) Combine (268:283-(175:182+363:371)) 1:MS ES+ 1.4e+005

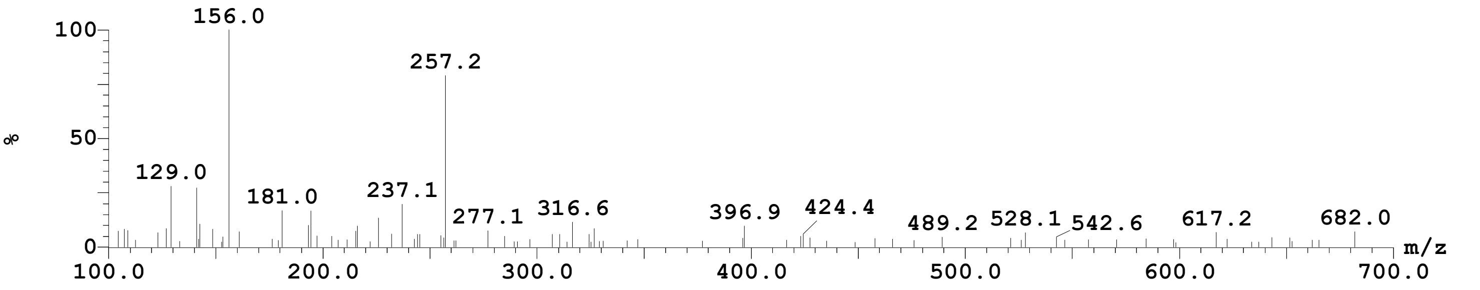

Peak ID **Time**
5 0.73

5:(Time: 0.73) Combine (268:283-(174:182+363:370)) 2:MS ES- 1.5e+004

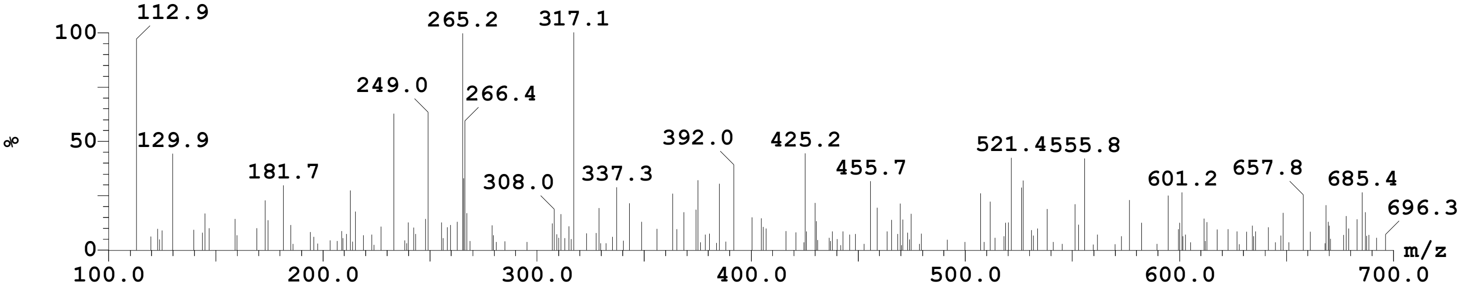

File:13zo178l3 Vial:5:38 ID:E4
Method:C:\MASSLYNX\1minLC_MS.olp

Peak ID **Time**
7 1.00

7: (Time: 1.00) Combine (367:382-281:288) 1:MS ES+ 2.8e+007

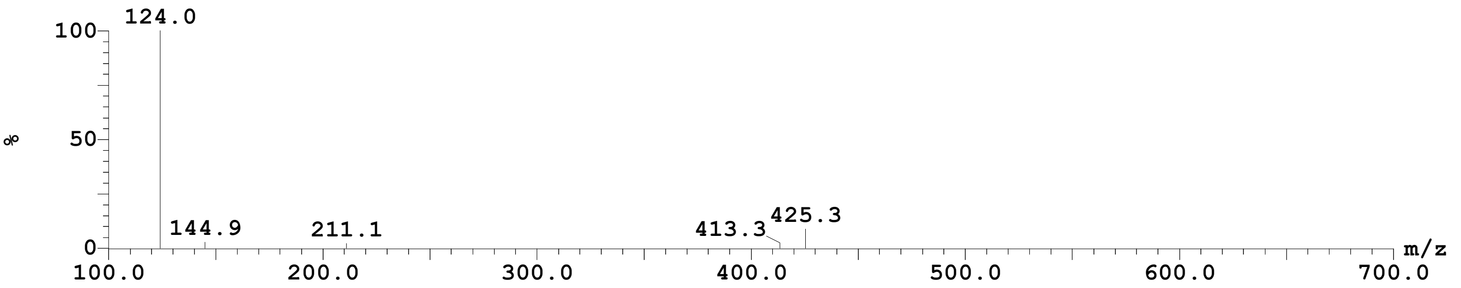

File:13zo392l2 Vial:5:39 ID: **E5**

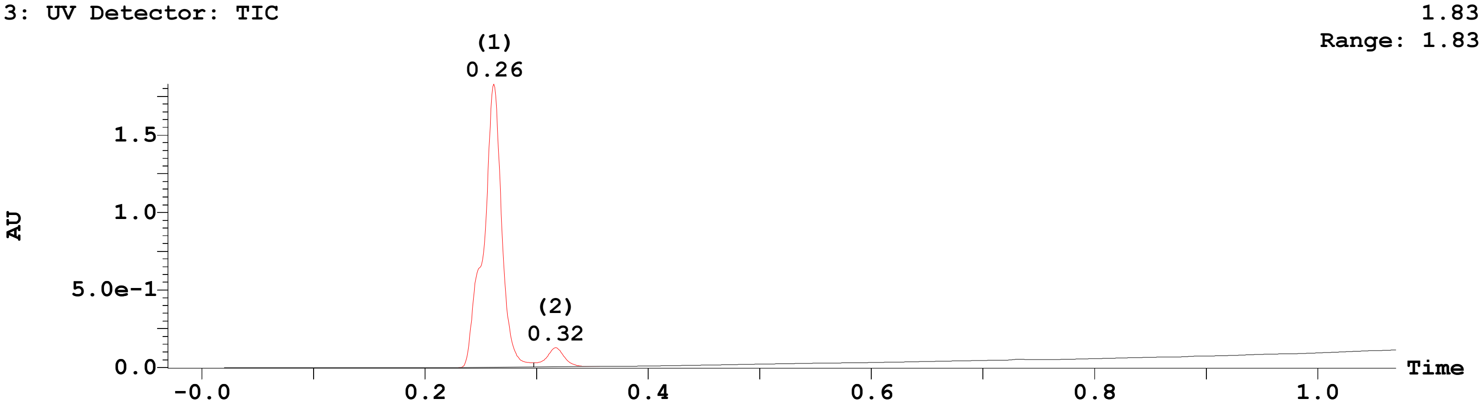

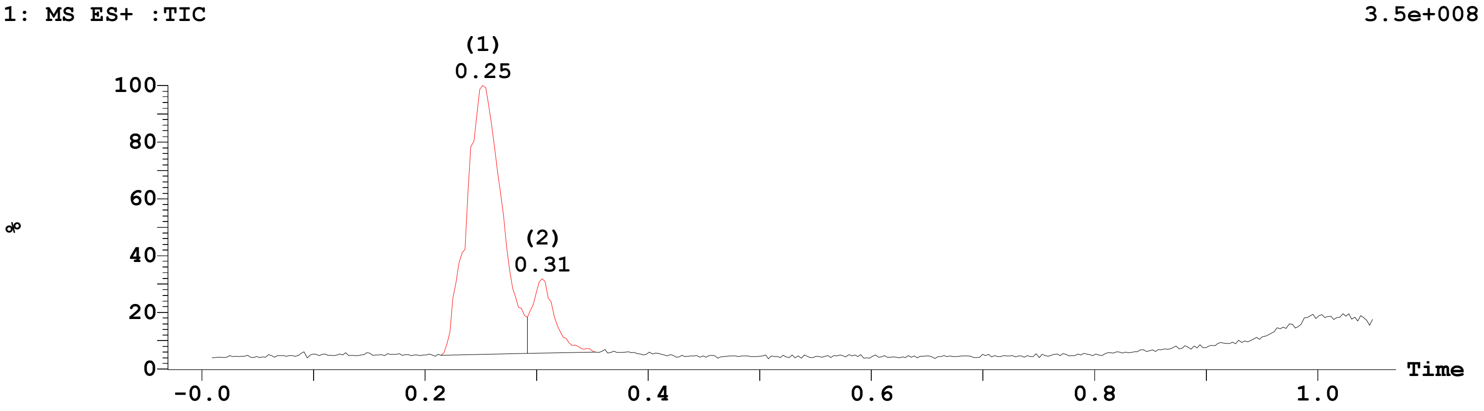

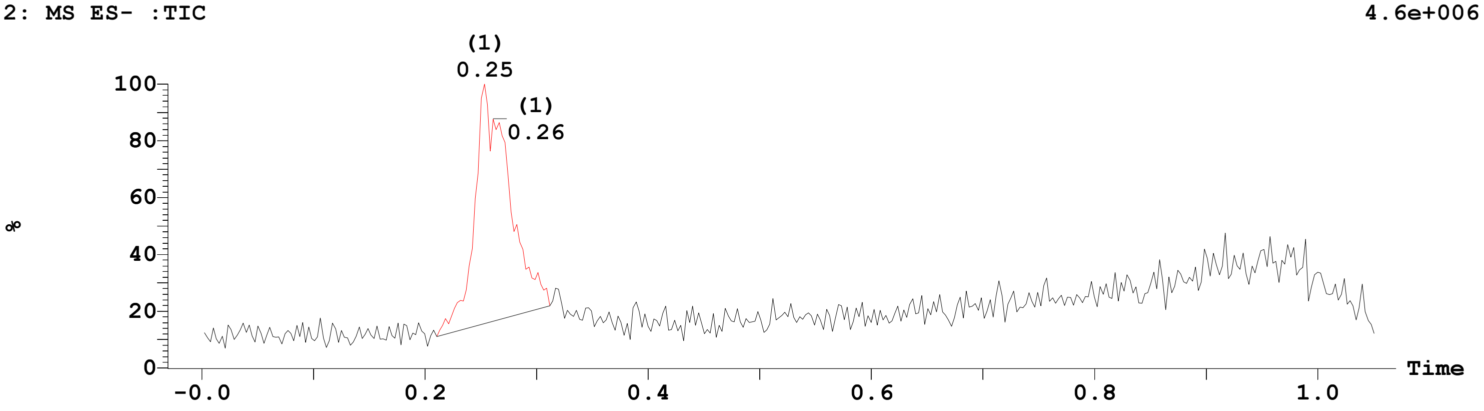

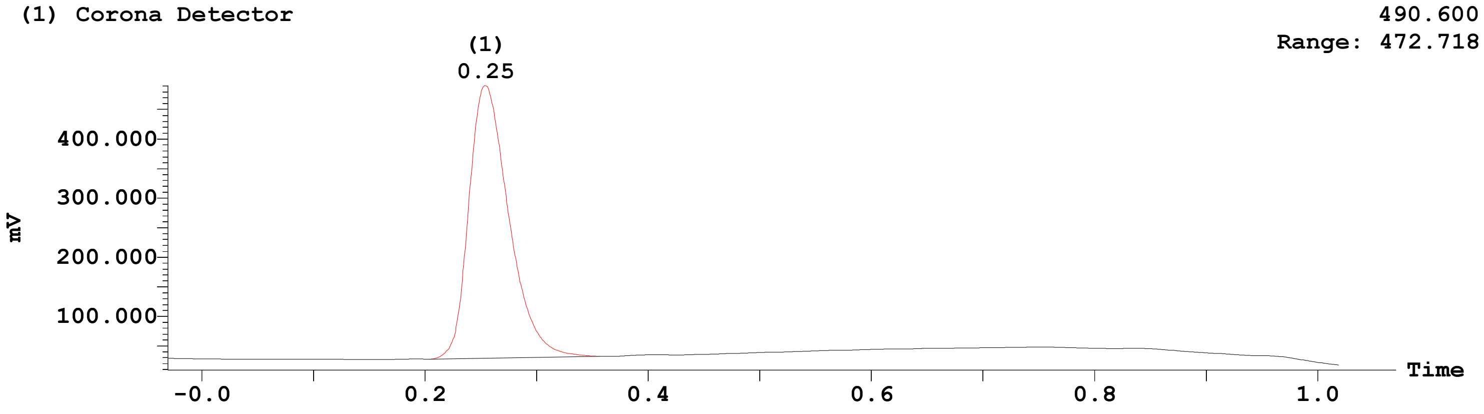

File:13zo392l2 Vial:5:39 ID:E5
Method:C:\MASSLYNX\1minLC_MS.olp

Peak ID **Time**
1 0.25

1:(Time: 0.26) Combine (91:106-(4:12+187:195)) 1:MS ES+ 1.2e+008

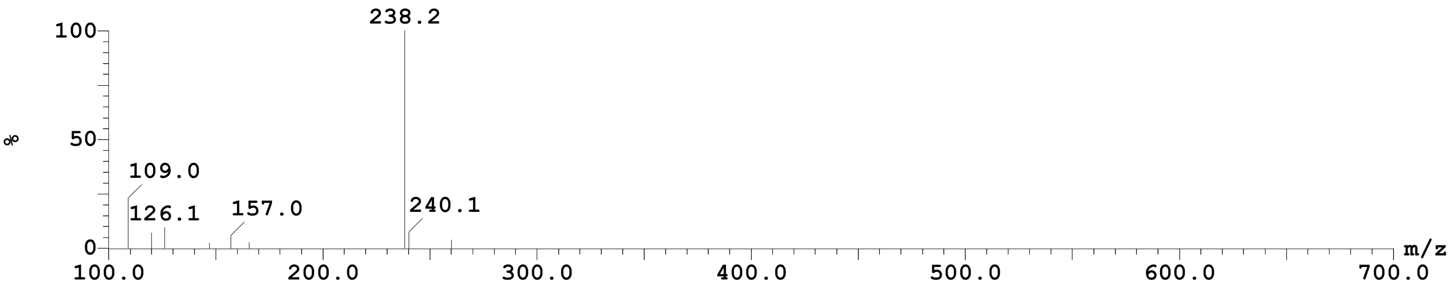

Peak ID **Time**
1 0.25

1:(Time: 0.25) Combine (88:103-(1:4+192:199)) 2:MS ES- 4.0e+005

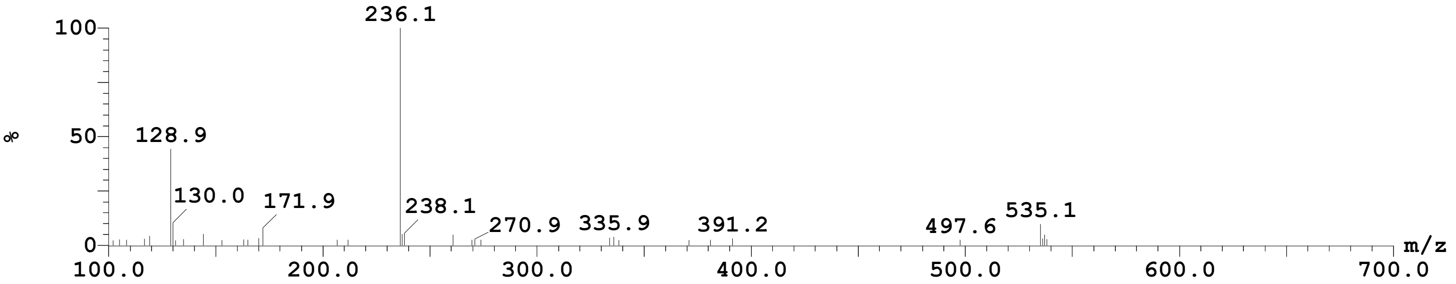

Peak ID **Time**
2 0.31

2:(Time: 0.31) Combine (107:123-(28:35+208:215)) 1:MS ES+ 3.1e+007

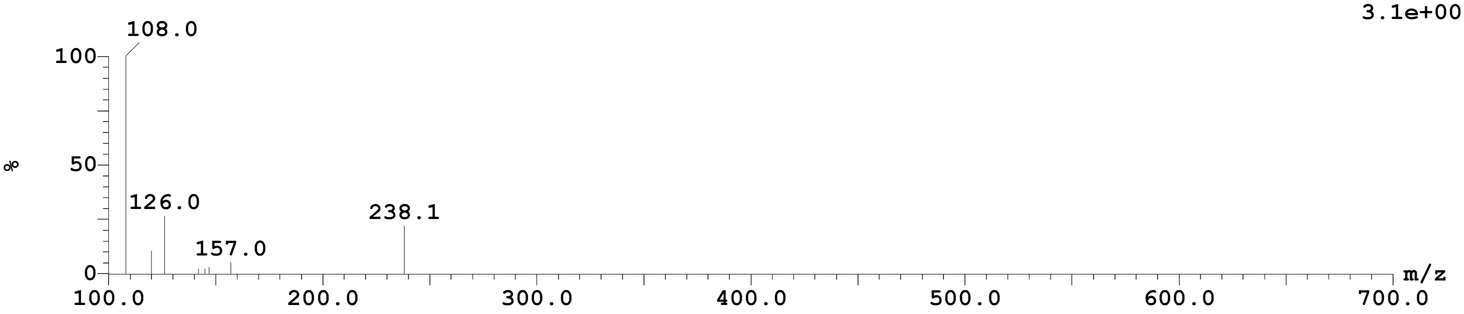

Peak ID **Time**
2 0.31

2:(Time: 0.32) Combine (111:126-(29:37+209:216)) 2:MS ES- 1.2e+004

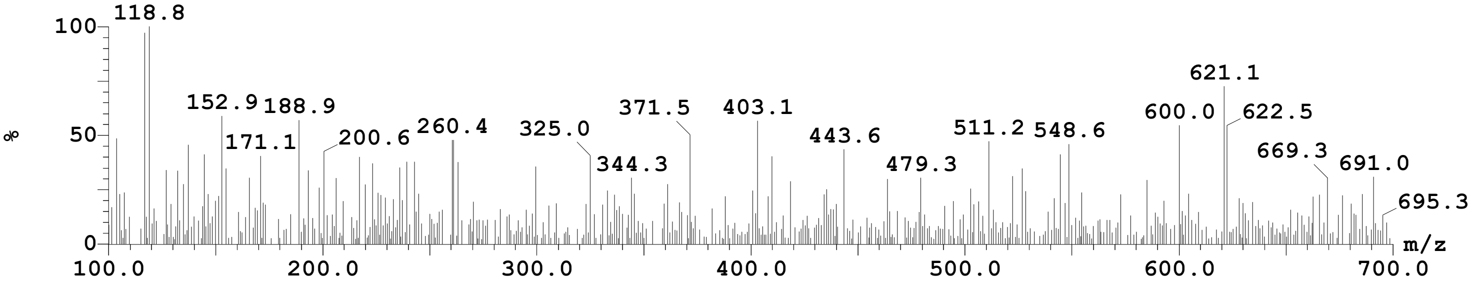

File:13zq136l1 Vial:5:17 ID: **E6**

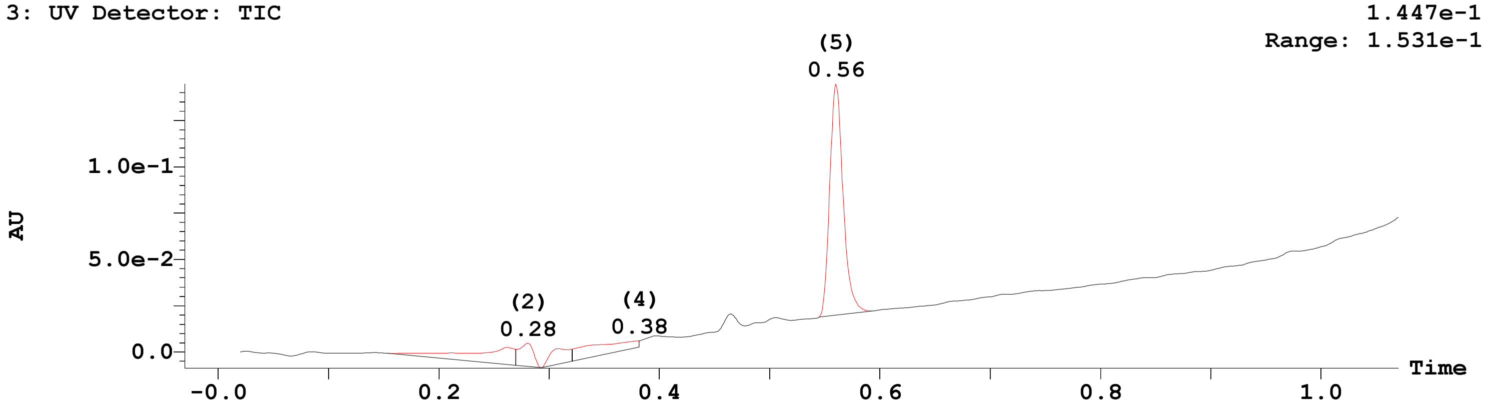

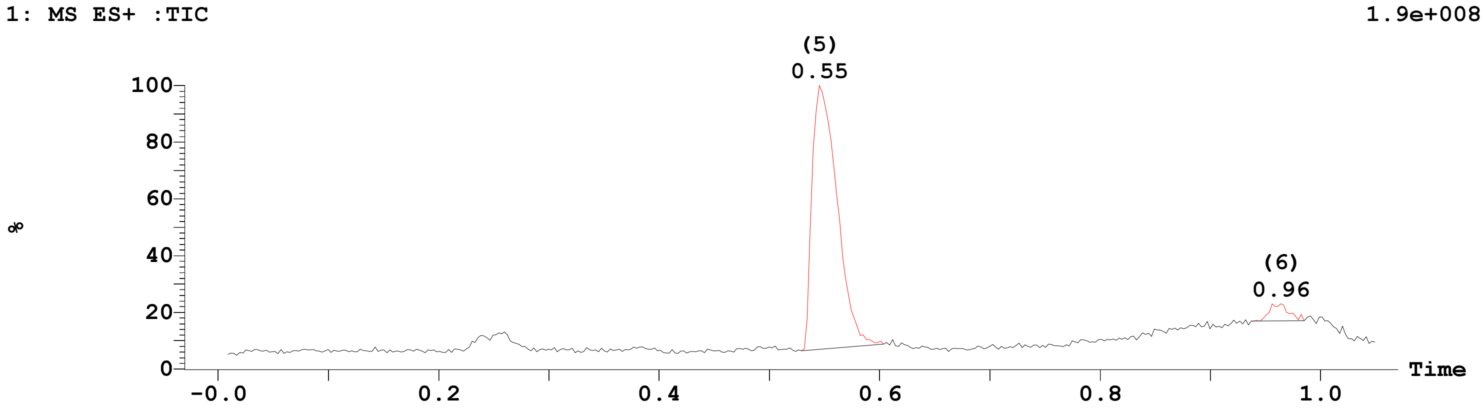

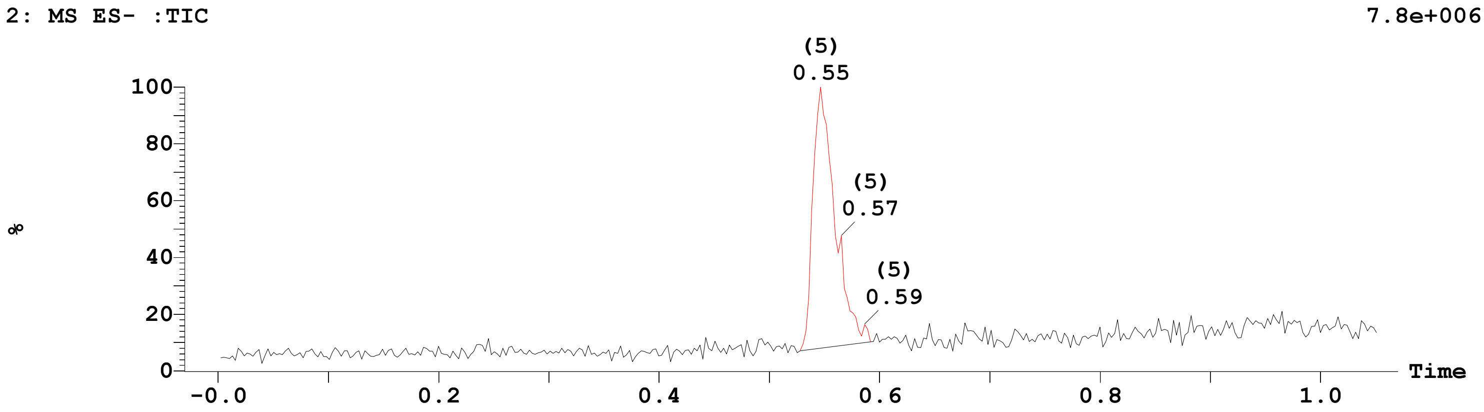

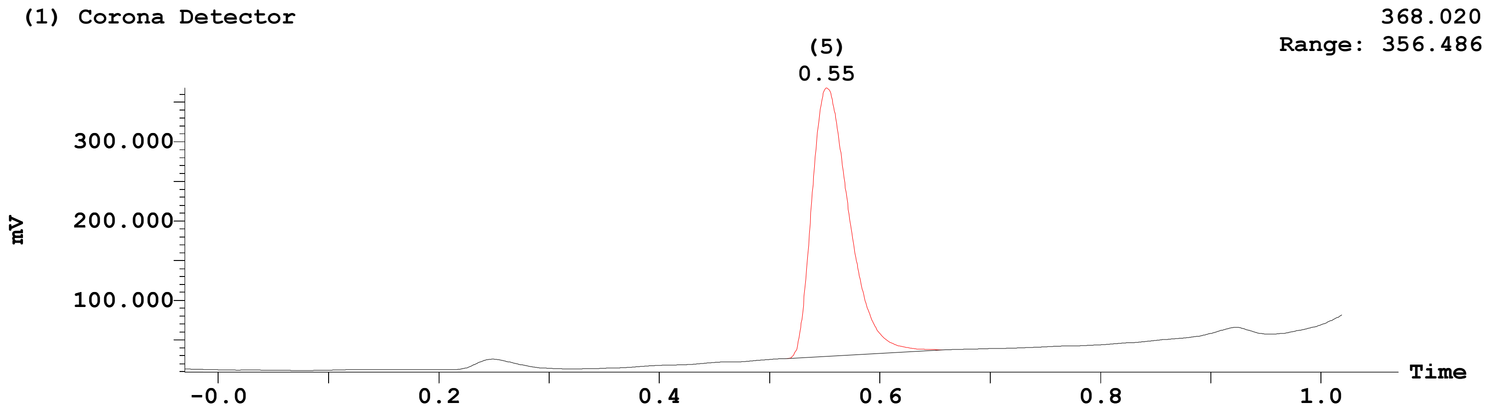

File:13zq136l1 Vial:5:17 ID:E6
Method:C:\MASSLYNX\1minLC_MS.olp

Peak ID **Time**
1 0.26

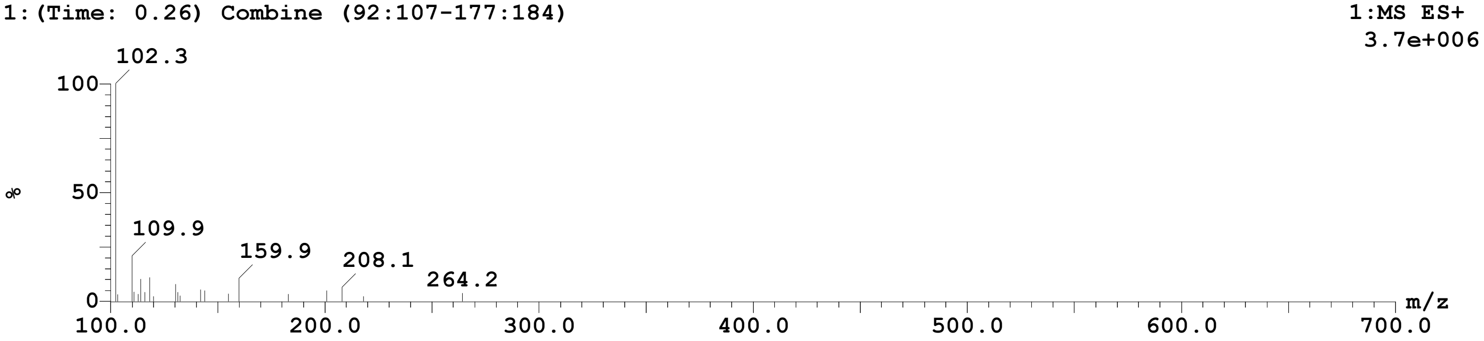

Peak ID **Time**
1 0.26

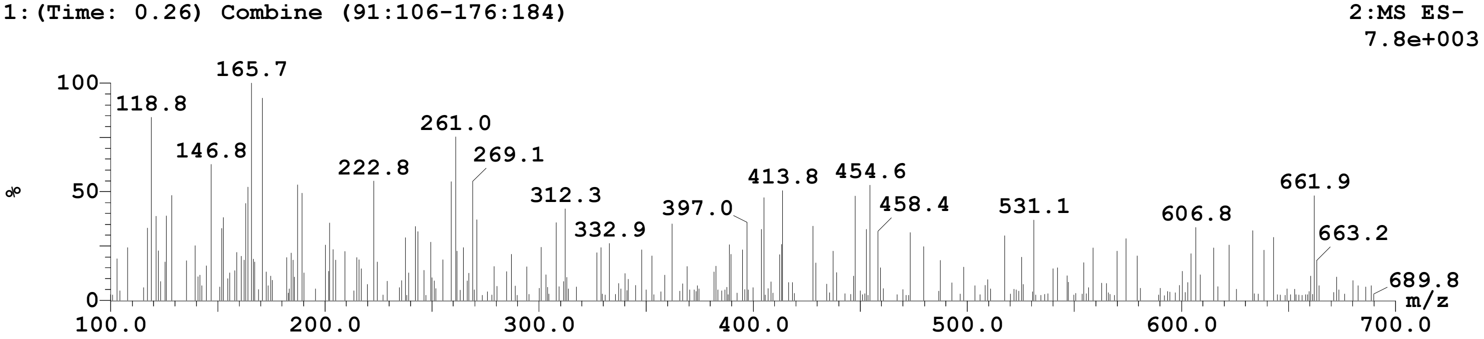

Peak ID **Time**
2 0.28

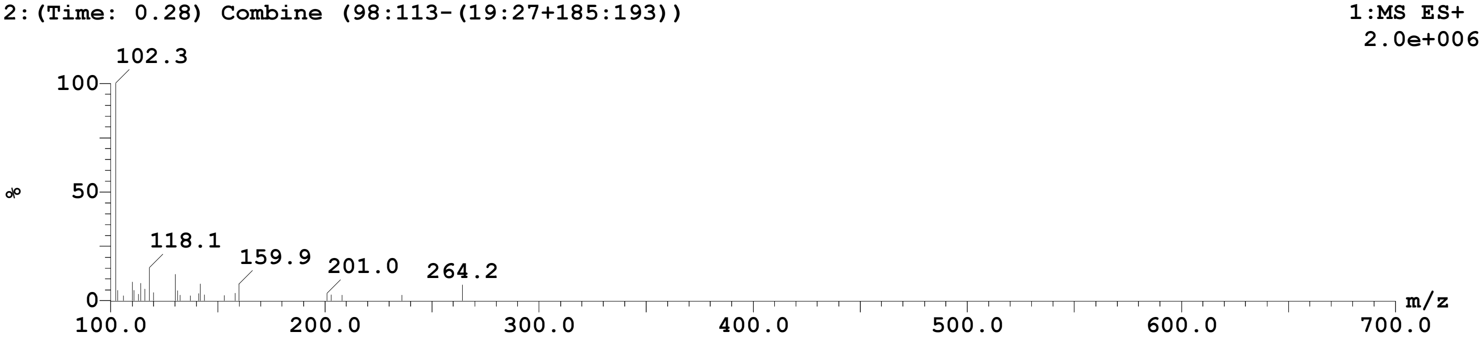

Peak ID **Time**
2 0.28

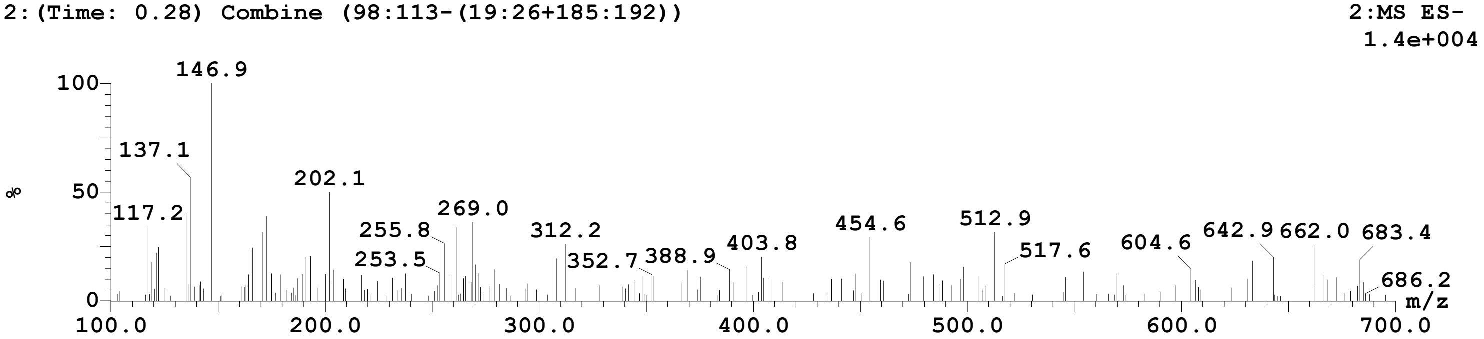

File:13zq136l1 Vial:5:17 ID:E6
Method:C:\MASSLYNX\1minLC_MS.olp

Peak ID **Time**
3 0.31

3:(Time: 0.31) Combine (109:124-(28:36+196:203)) 1:MS ES+
3.0e+005

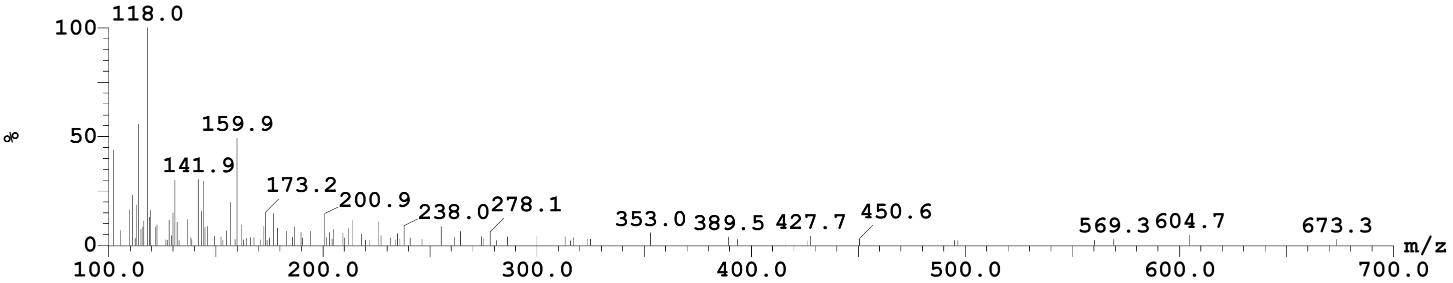

Peak ID **Time**
4 0.38

4:(Time: 0.38) Combine (136:151-(38:46+219:226)) 1:MS ES+
8.9e+005

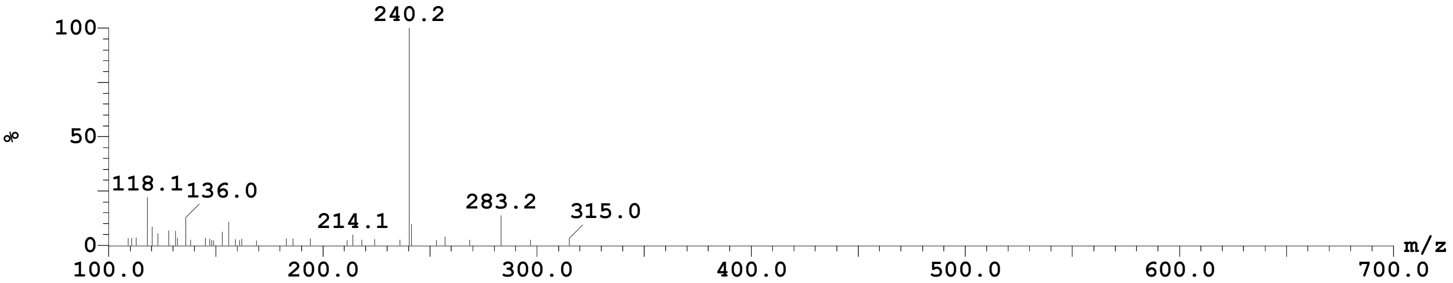

Peak ID **Time**
4 0.38

4:(Time: 0.38) Combine (136:151-(38:45+218:226)) 2:MS ES-
7.5e+003

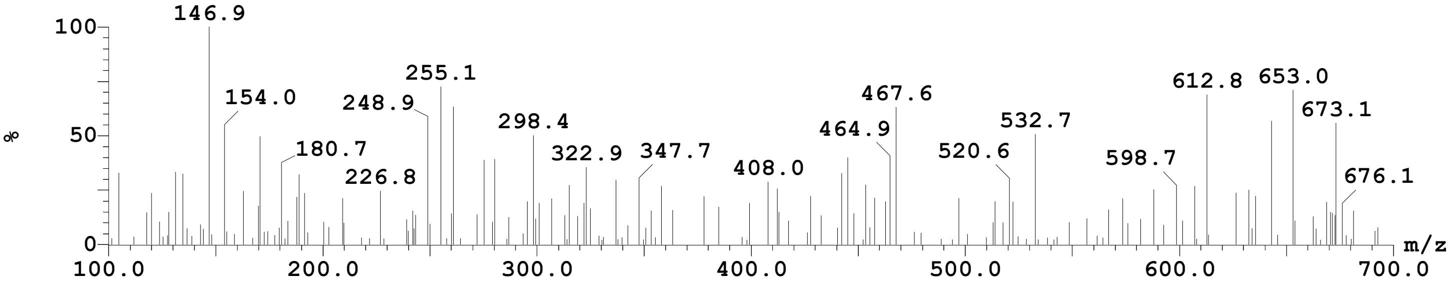

Peak ID **Time**
5 0.55

5:(Time: 0.55) Combine (197:212-(117:124+302:309)) 1:MS ES+
7.1e+007

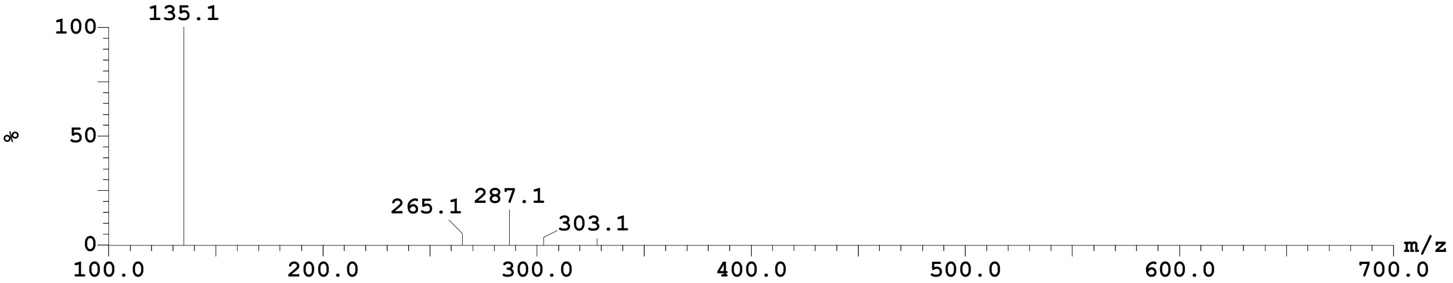

File:13zq136l1 Vial:5:17 ID:E6
Method:C:\MASSLYNX\1minLC_MS.olp

Peak ID Time
5 0.55

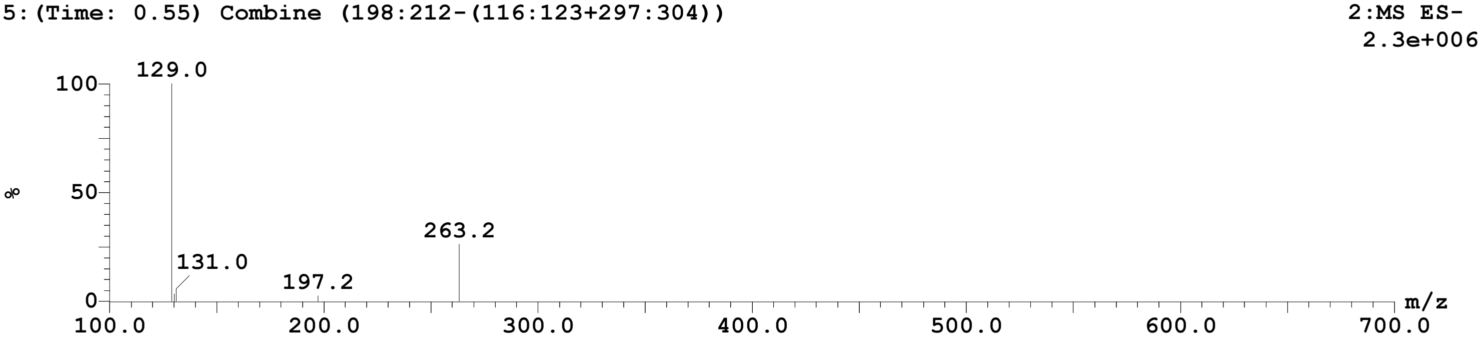

Peak ID Time
6 0.96

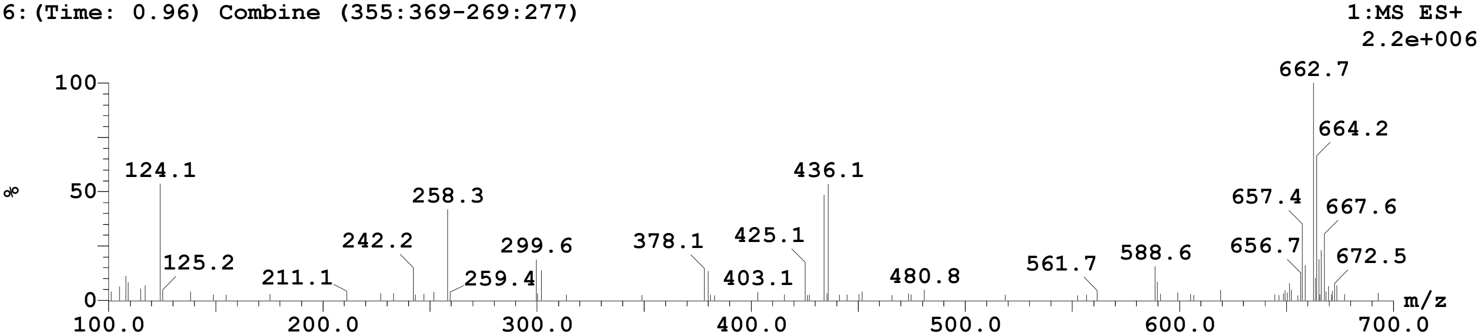

File:13zo374l2 Vial:5:47 ID: **E7**

3: UV Detector: TIC 2.7e-1
Range: 2.792e-1

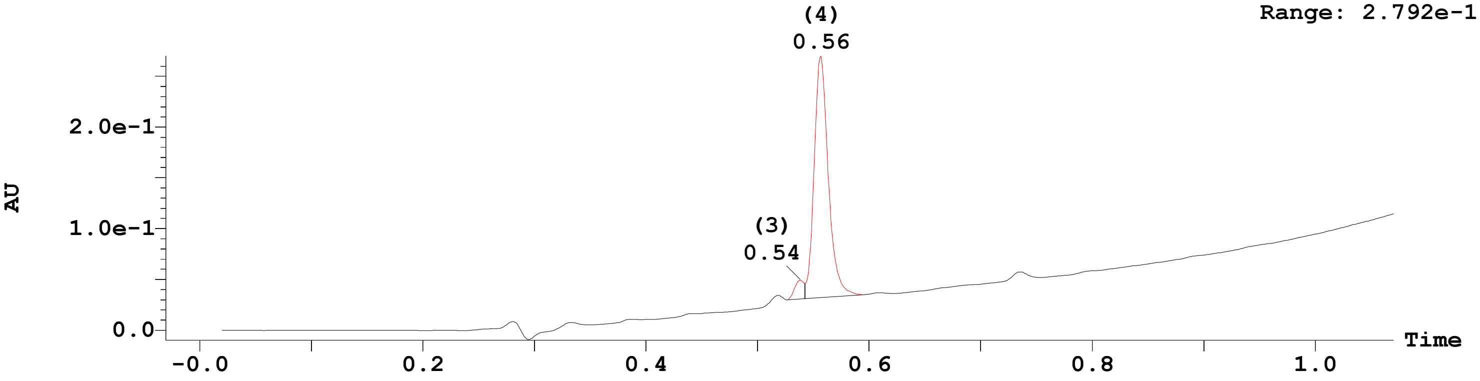

1: MS ES+ :TIC 1.3e+008

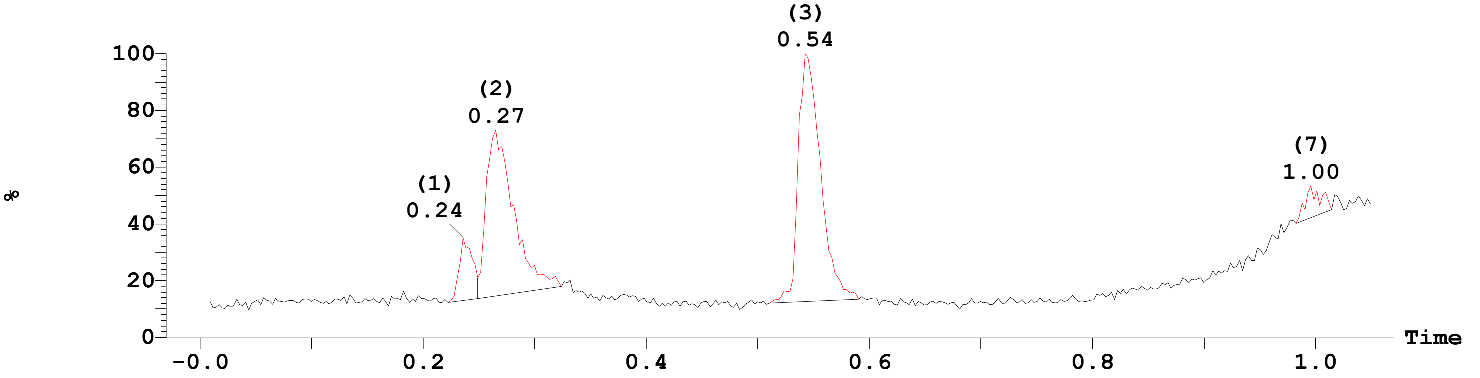

2: MS ES- :TIC 3.1e+007

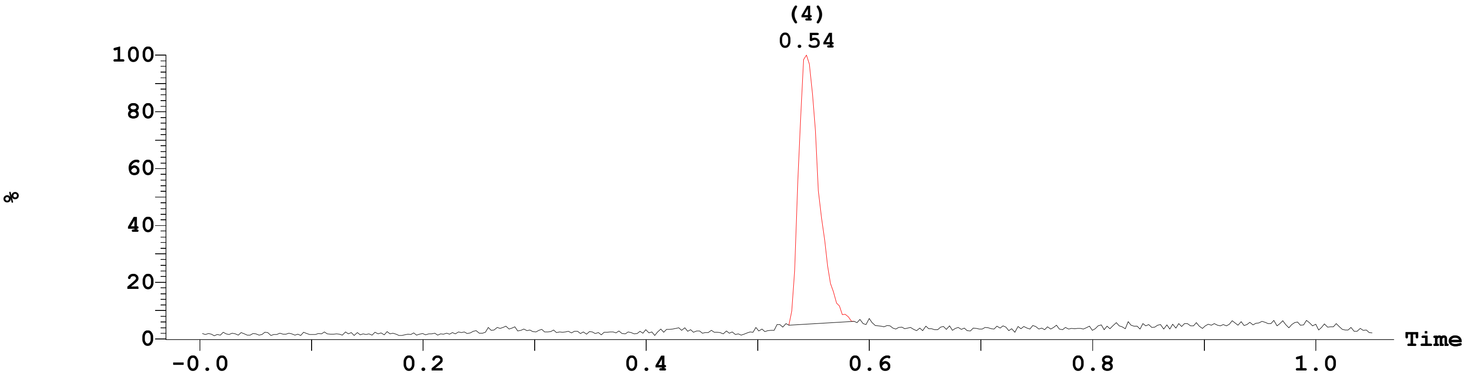

(1) Corona Detector 352.980
Range: 336.281

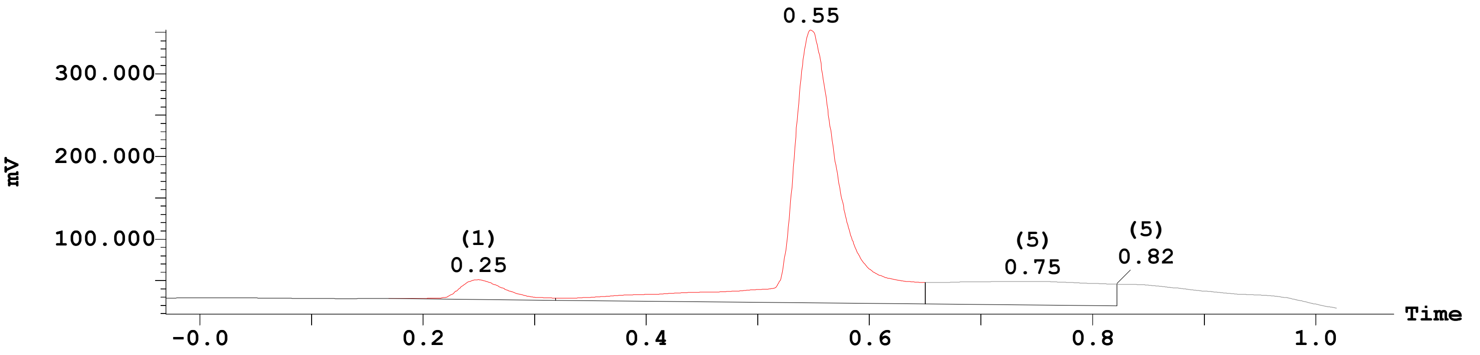

File:13zo374l2 Vial:5:47

Peak ID **Time**
1 0.24

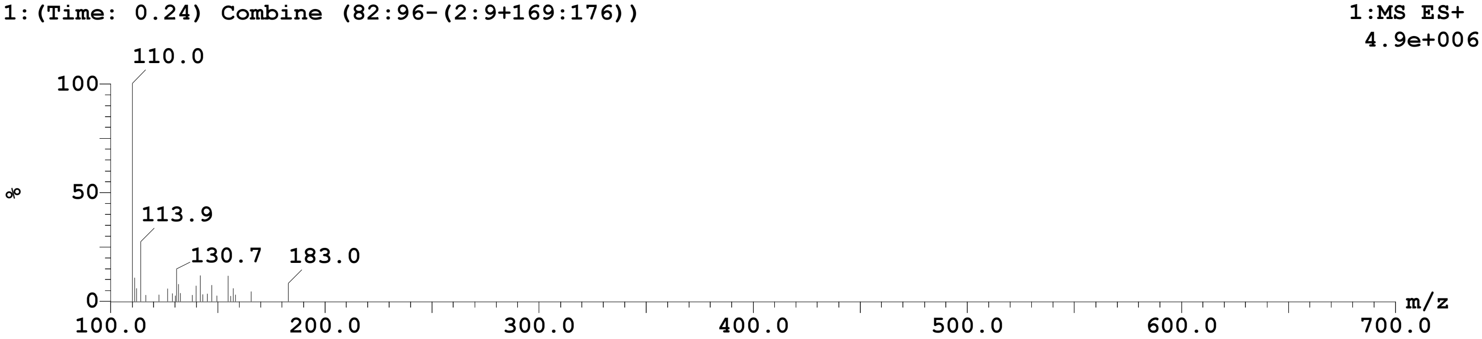

Peak ID **Time**
2 0.27

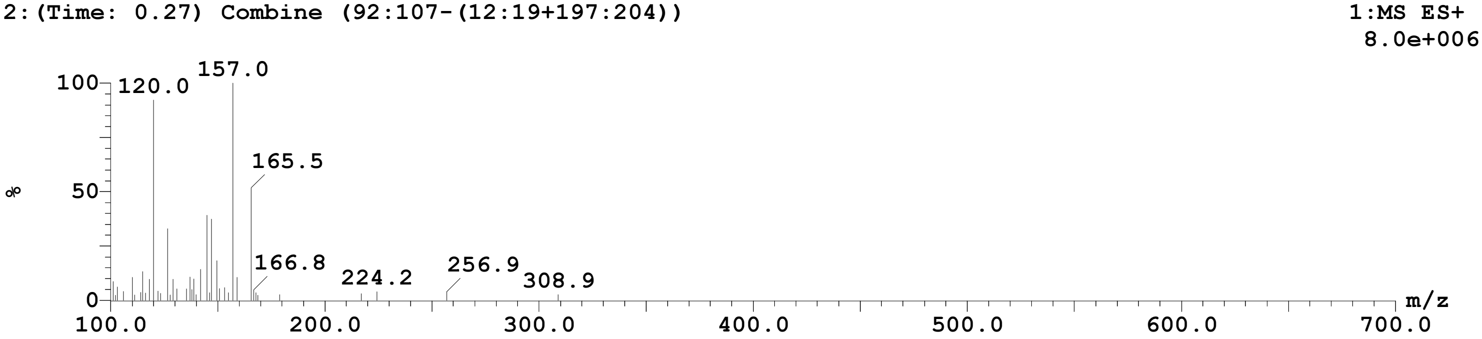

Peak ID **Time**
3 0.54

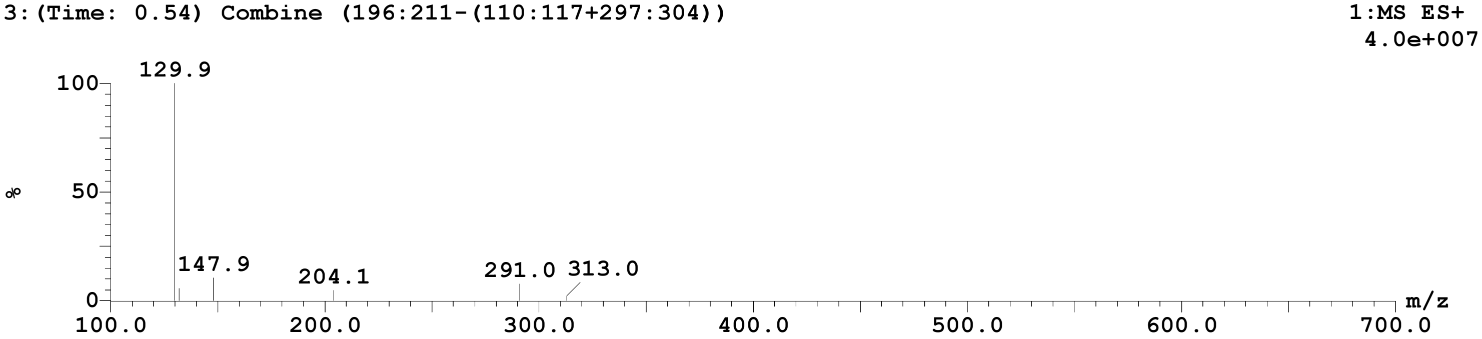

Peak ID **Time**
3 0.54

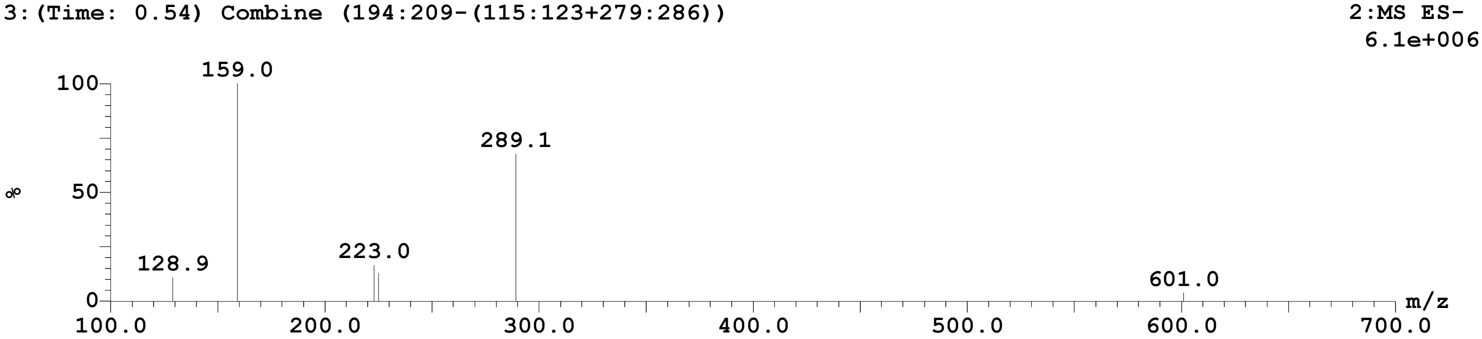

File:13zo374l2 Vial:5:47 ID:E7
Method:C:\MASSLYNX\1minLC_MS.olp

Peak ID **Time**
4 0.54

4:(Time: 0.56) Combine (202:217-(122:129+299:306)) 1:MS ES+ 4.1e+007

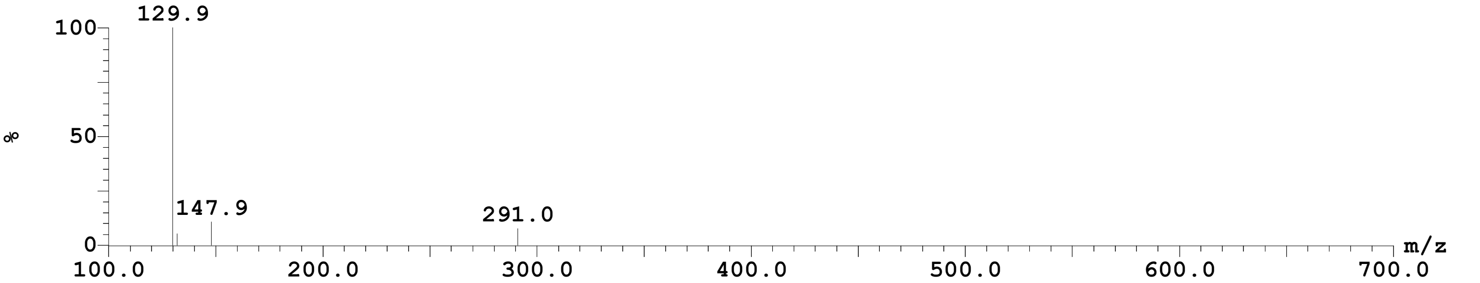

Peak ID **Time**
4 0.54

4:(Time: 0.54) Combine (197:211-(116:123+295:302)) 2:MS ES- 7.0e+006

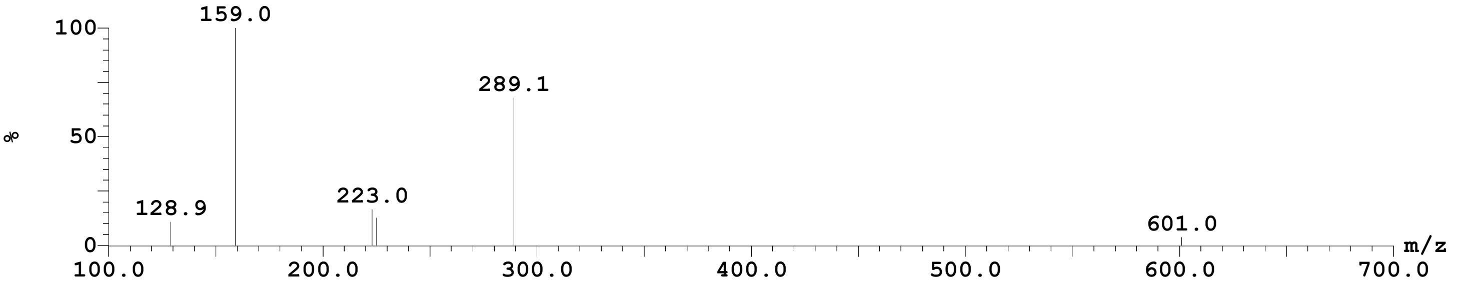

Peak ID **Time**
7 1.00

7:(Time: 1.00) Combine (366:381-287:294) 1:MS ES+ 2.7e+007

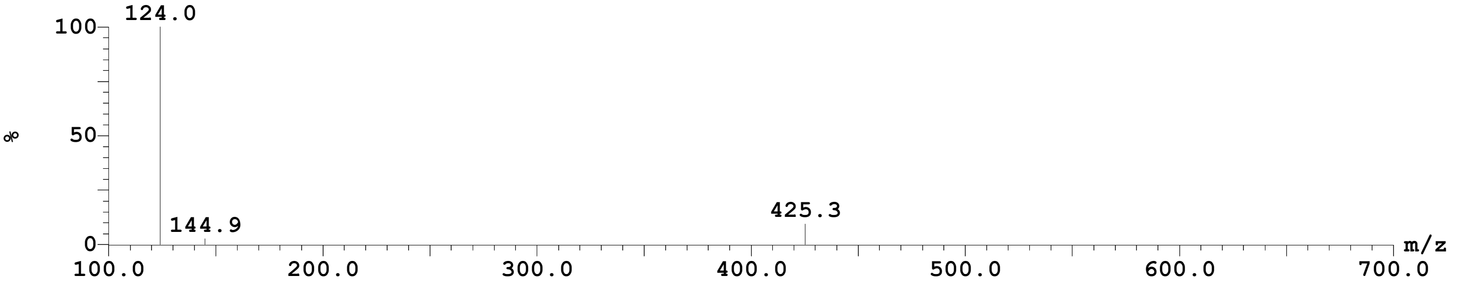

File:13zp825l1 Vial:5:48 ID: **E8**

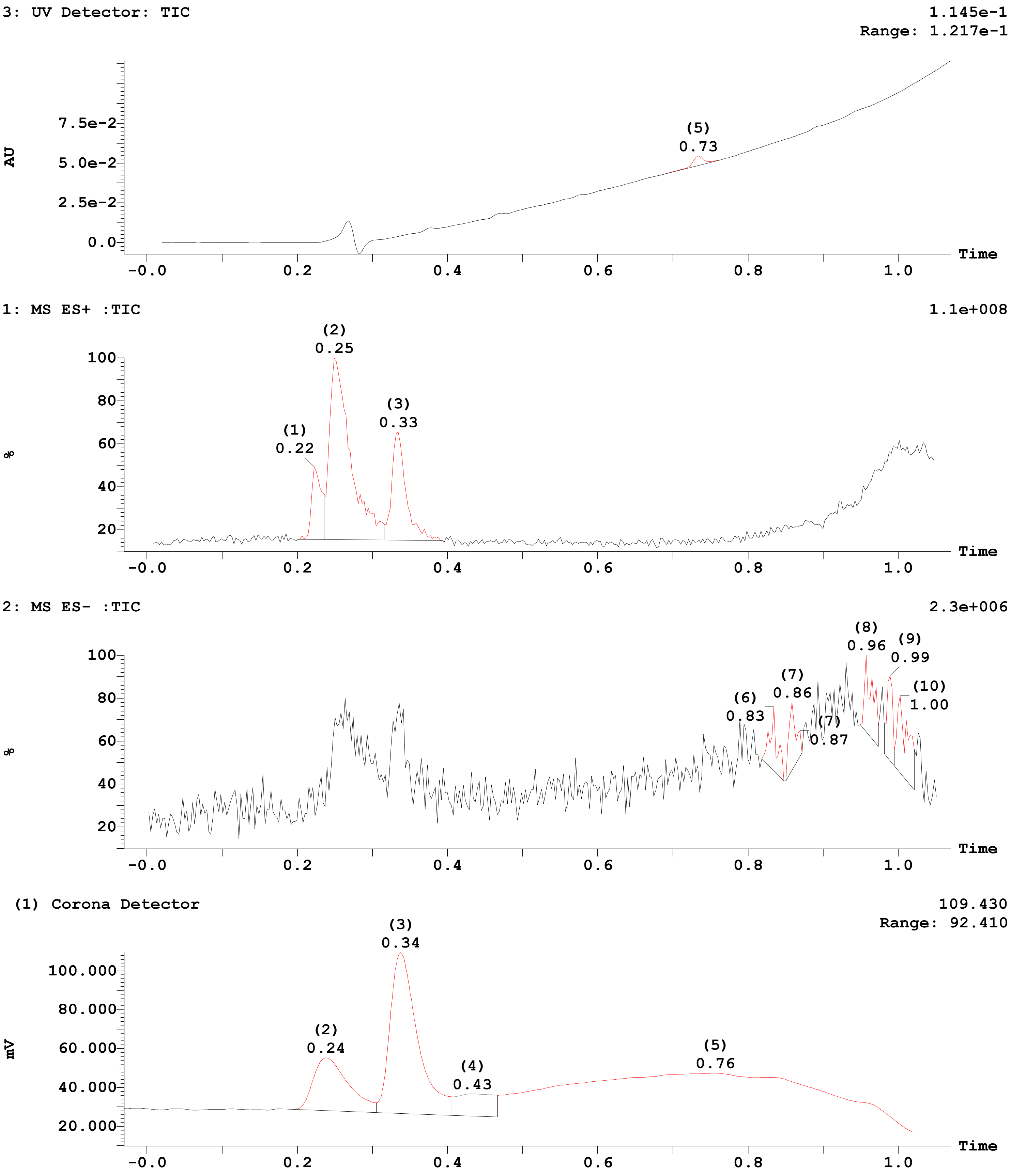

File:13zp825l1 Vial:5:48 ID:E8
Method:C:\MASSLYNX\1minLC_MS.olp

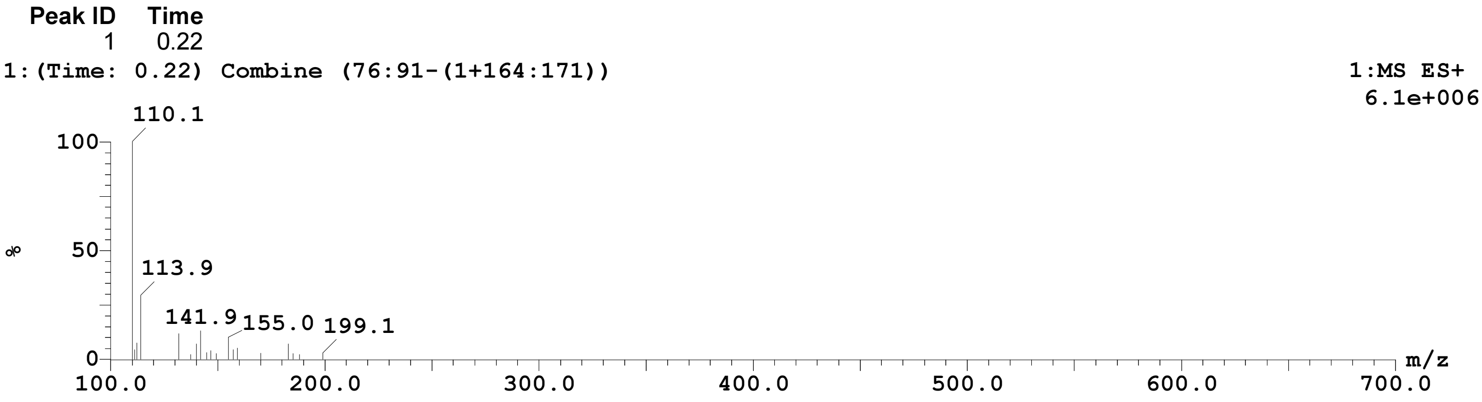

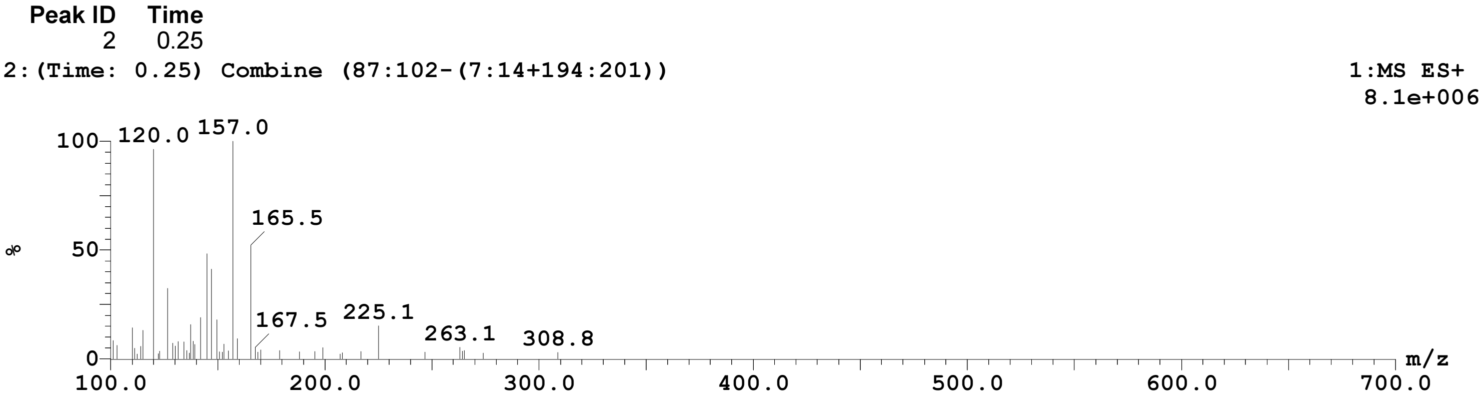

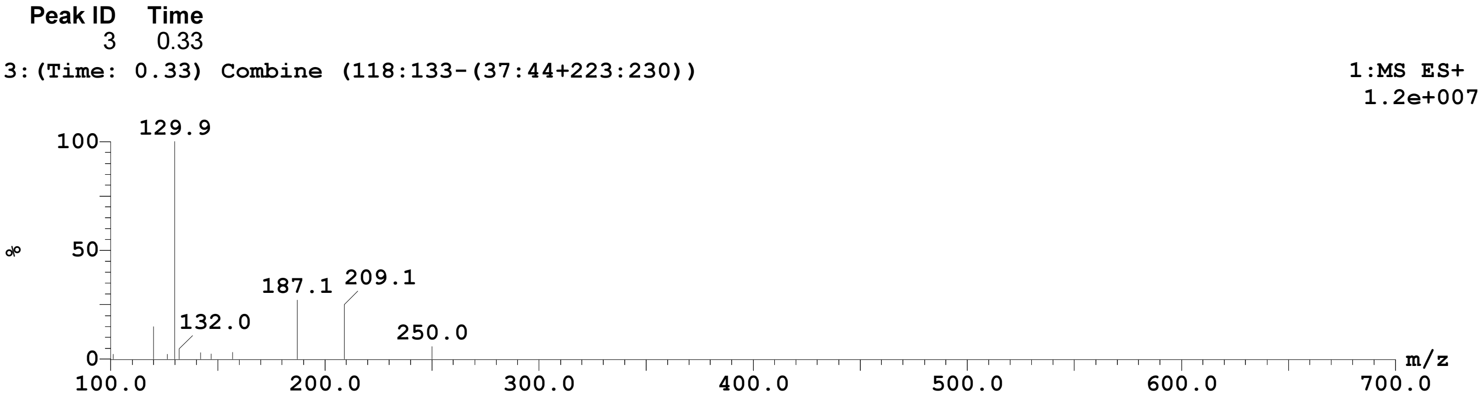

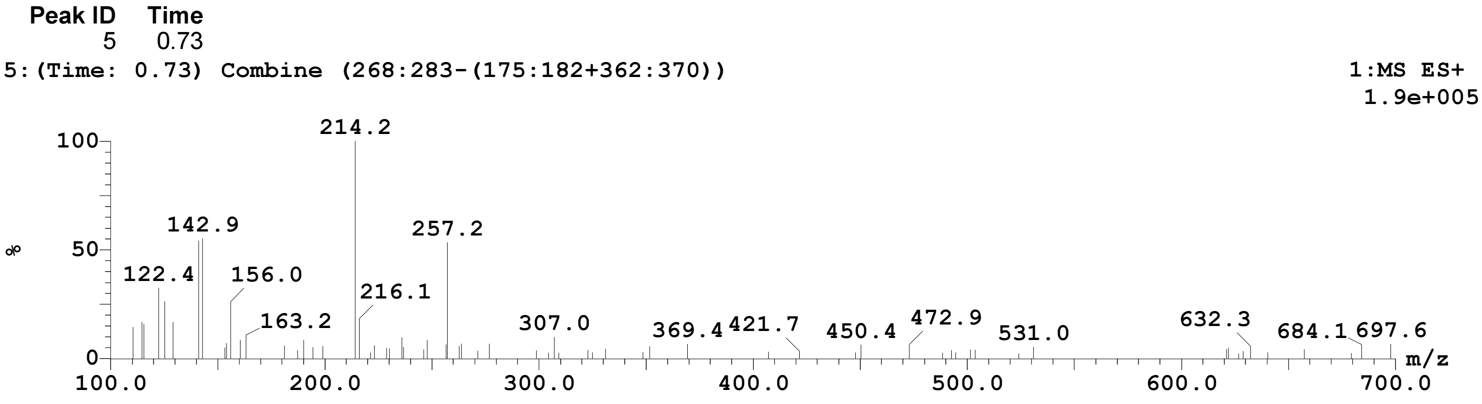

File:13zp825l1 Vial:5:48 ID:E8
Method:C:\MASSLYNX\1minLC_MS.olp

Peak ID **Time**
5 0.73

5:(Time: 0.73) Combine (268:283-(174:182+362:369)) 2:MS ES- 2.3e+004

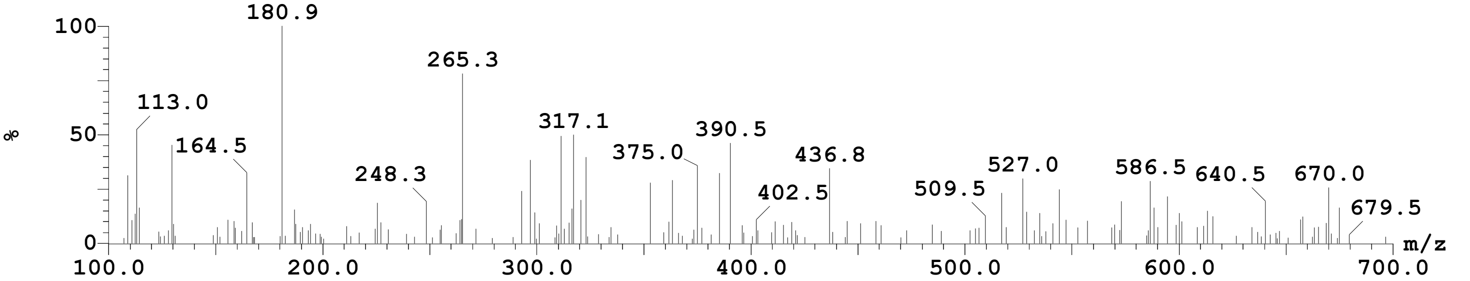

Peak ID **Time**
6 0.83

6:(Time: 0.83) Combine (306:320-(225:232+393:400)) 2:MS ES- 5.3e+004

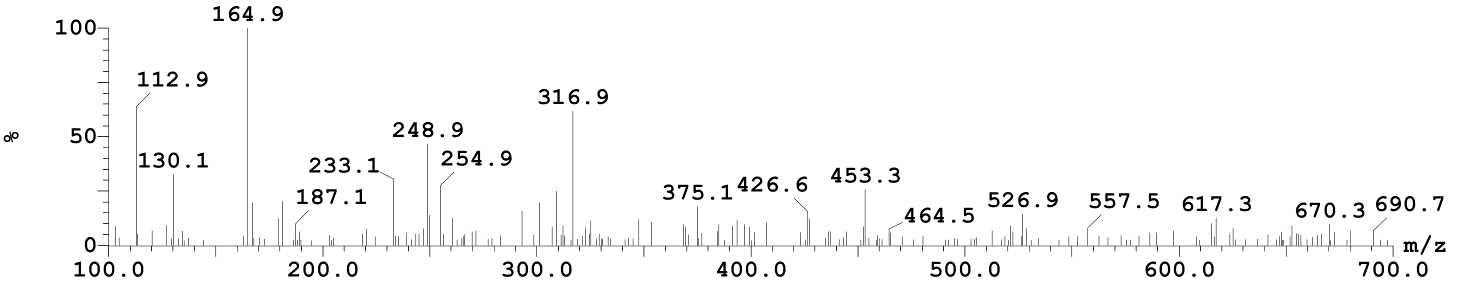

Peak ID **Time**
7 0.86

7:(Time: 0.86) Combine (315:329-(237:244+402:409)) 2:MS ES- 6.1e+004

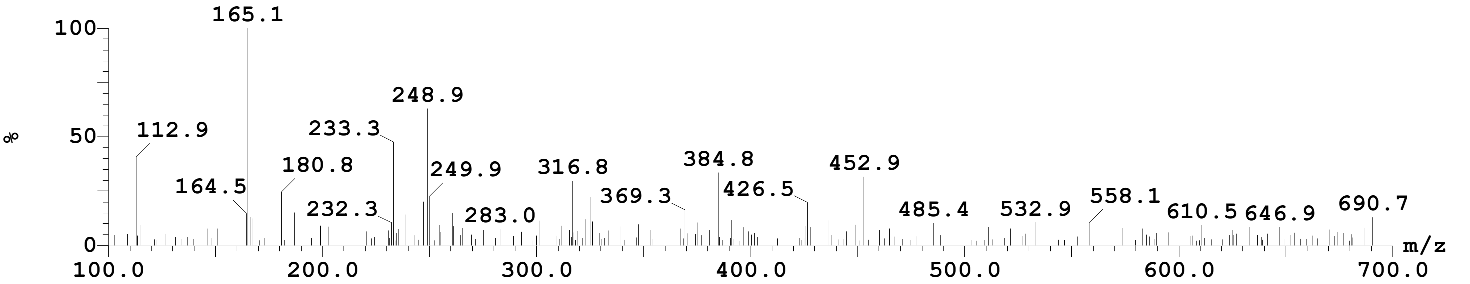

Peak ID **Time**
8 0.96

8:(Time: 0.96) Combine (351:366-274:281) 2:MS ES- 1.7e+005

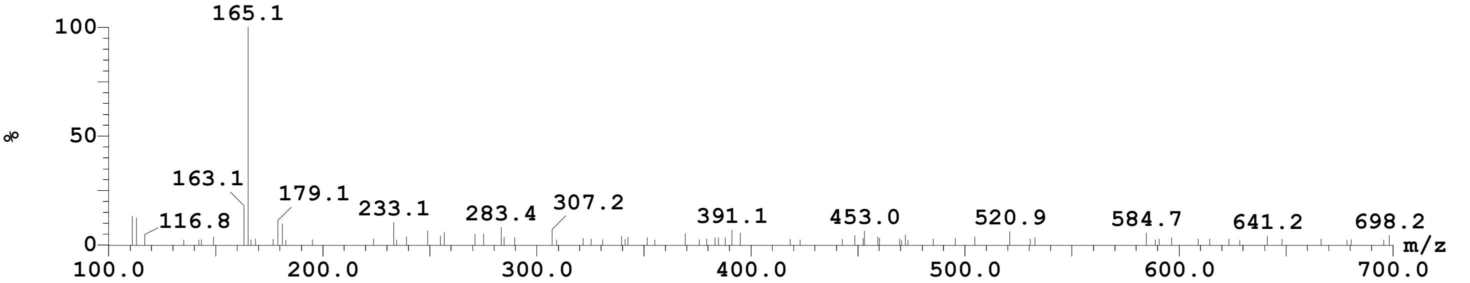

File:13zp825l1 Vial:5:48 ID:E8
Method:C:\MASSLYNX\1minLC_MS.olp

Peak ID	Time
9	0.99

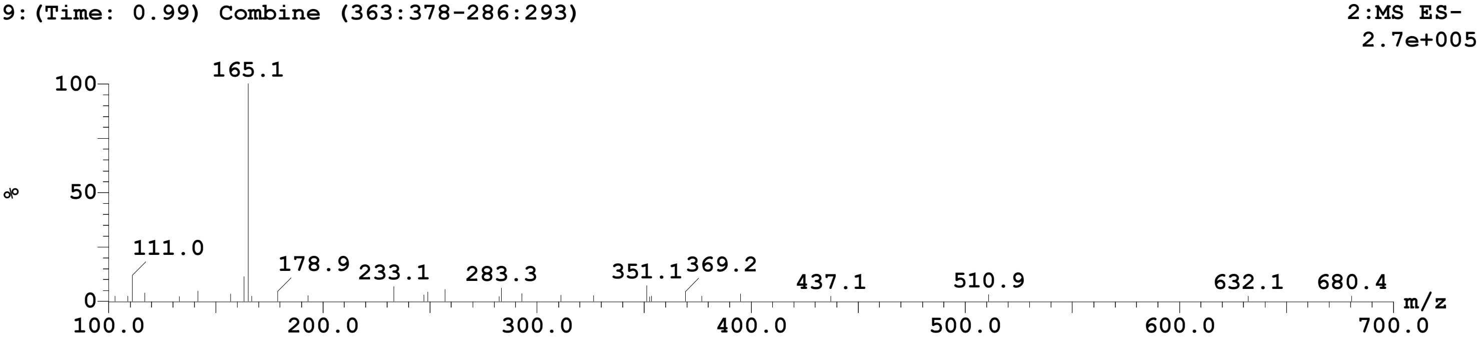

Peak ID	Time
10	1.00

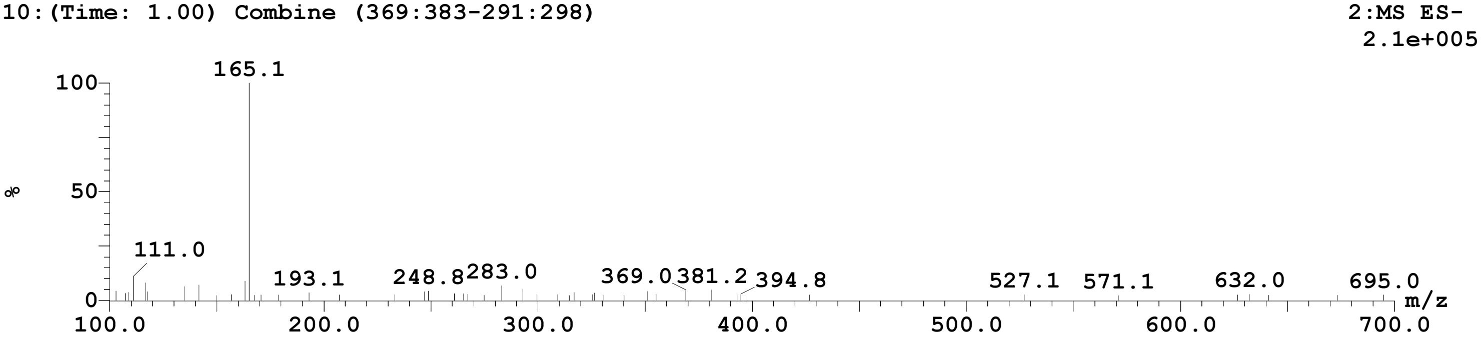

SYNGENTA SQD OPEN ACCESS LC-MS

File:13zo191l2 Vial:5:52 ID: **E9**

3: UV Detector: TIC 1.163e-1
Range: 1.249e-1

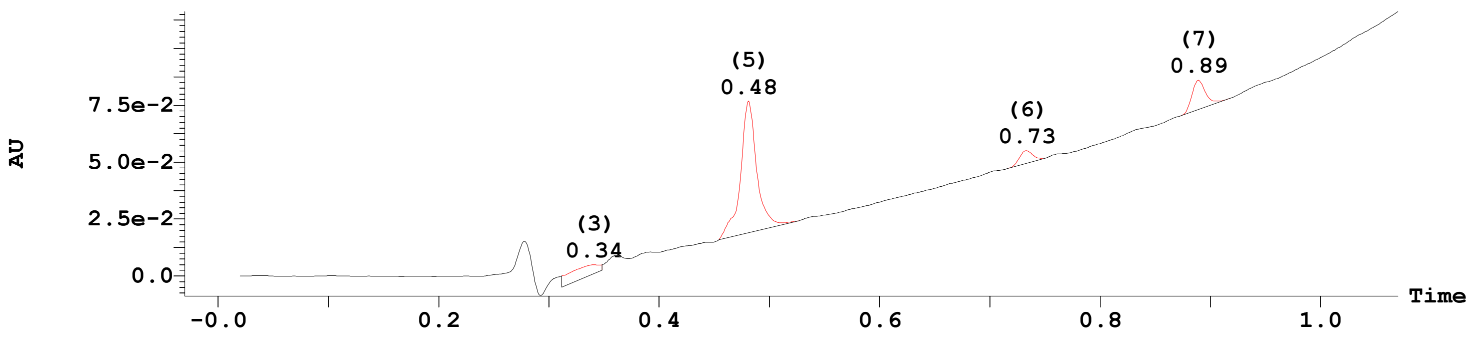

1: MS ES+ :TIC 3.0e+008

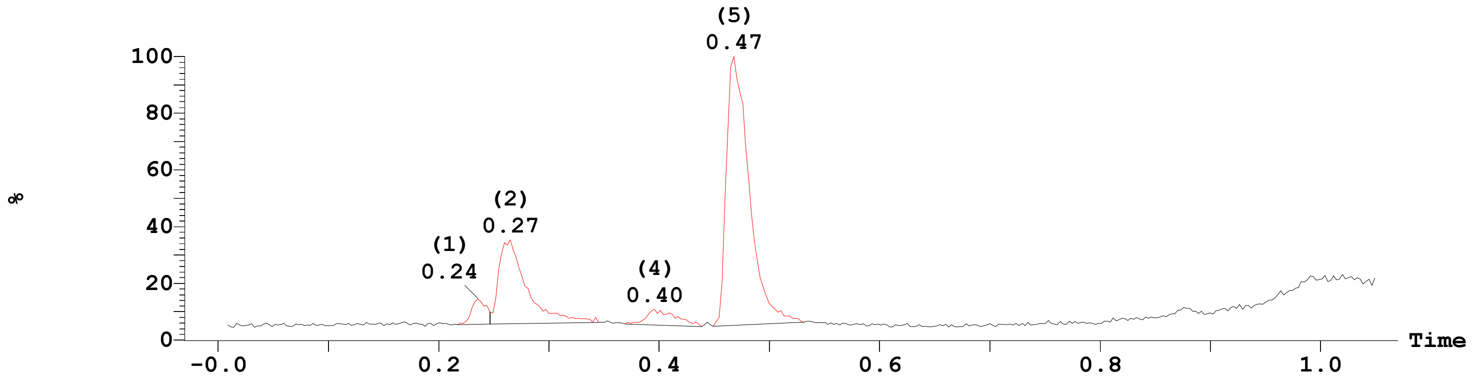

2: MS ES- :TIC 6.5e+006

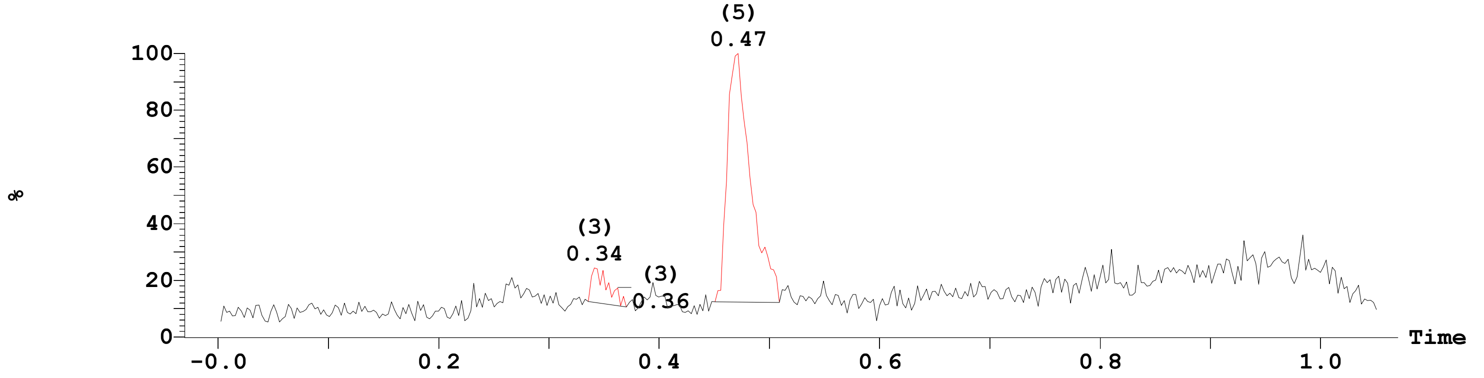

(1) Corona Detector 508.410
Range: 491.948

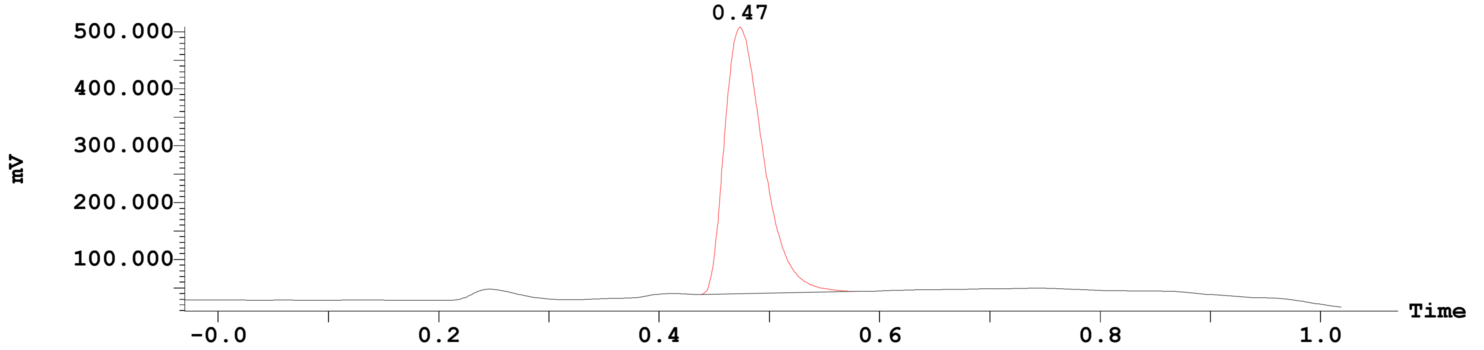

File:13zo191l2 Vial:5:52 ID:E9
Method:C:\MASSLYNX\1minLC_MS.olp

Peak ID	Time
1	0.24

1:(Time: 0.24) Combine (82:96-(1:7+168:175)) 1:MS ES+ 4.8e+006

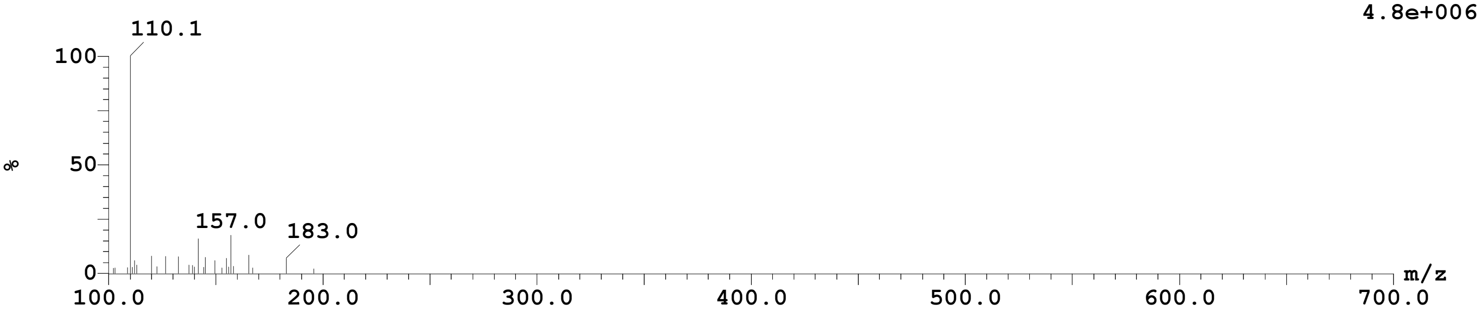

Peak ID	Time
2	0.27

2:(Time: 0.27) Combine (92:107-(11:18+205:212)) 1:MS ES+ 8.3e+006

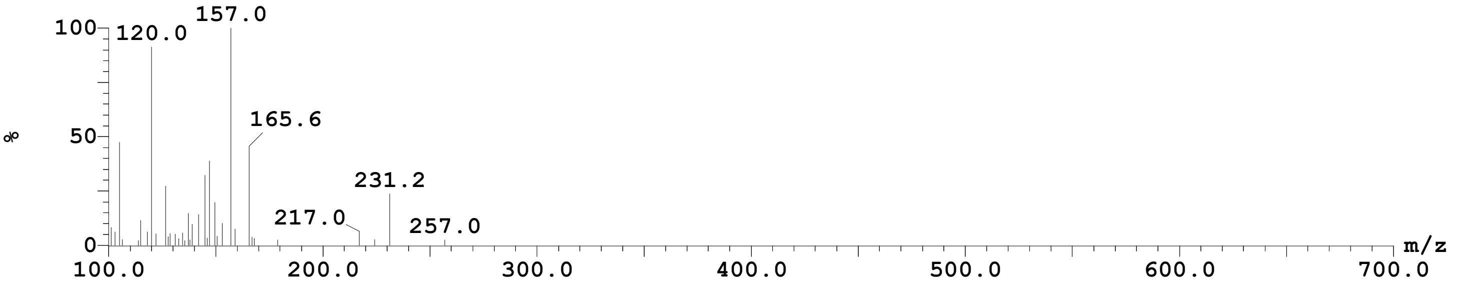

Peak ID	Time
3	0.34

3:(Time: 0.34) Combine (121:136-(35:42+206:214)) 1:MS ES+ 1.6e+006

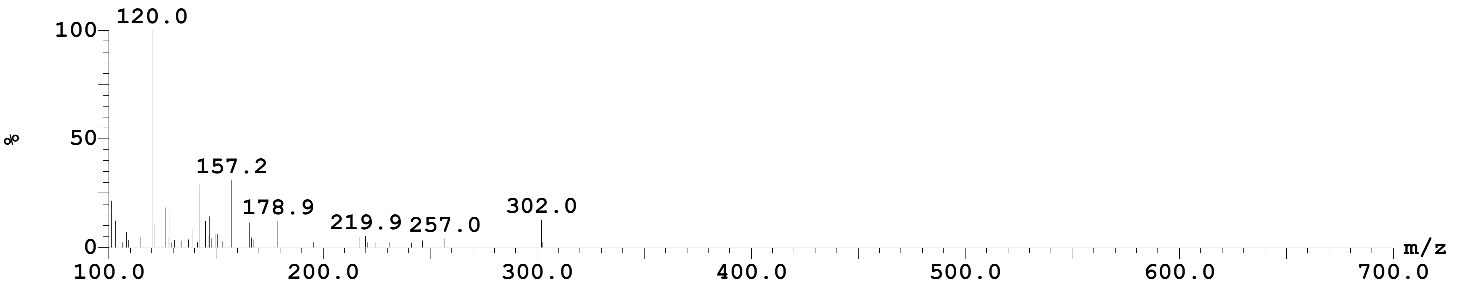

Peak ID	Time
3	0.34

3:(Time: 0.34) Combine (120:135-(44:51+214:221)) 2:MS ES- 2.5e+005

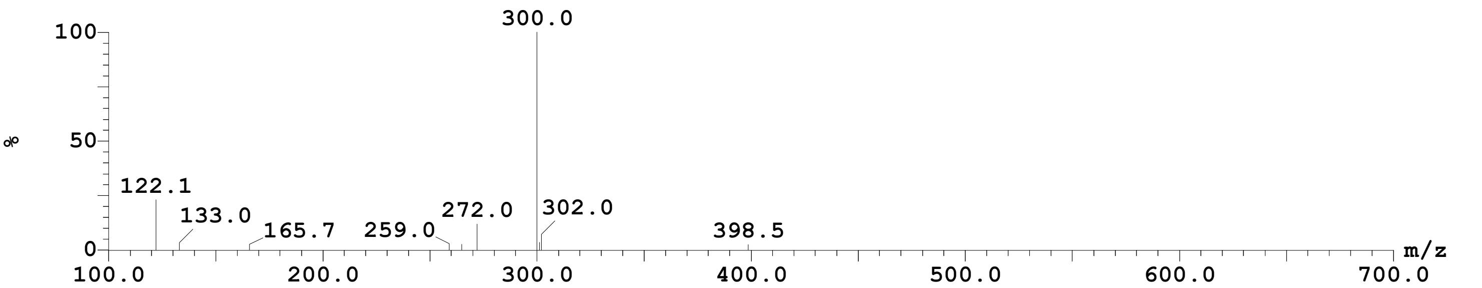

File:13zo191l2 Vial:5:52 ID:E9
Method:C:\MASSLYNX\1minLC_MS.olp

Peak ID **Time**
4 0.40

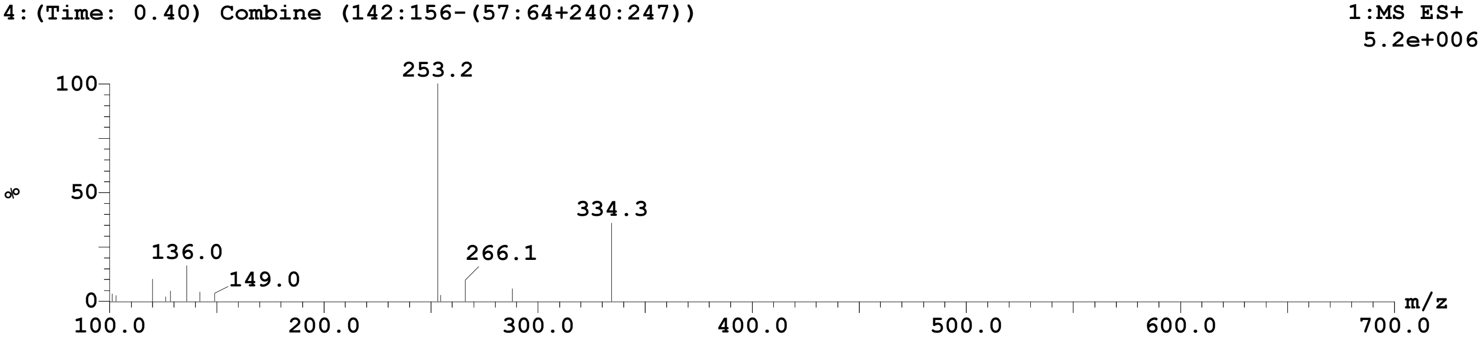

Peak ID **Time**
5 0.47

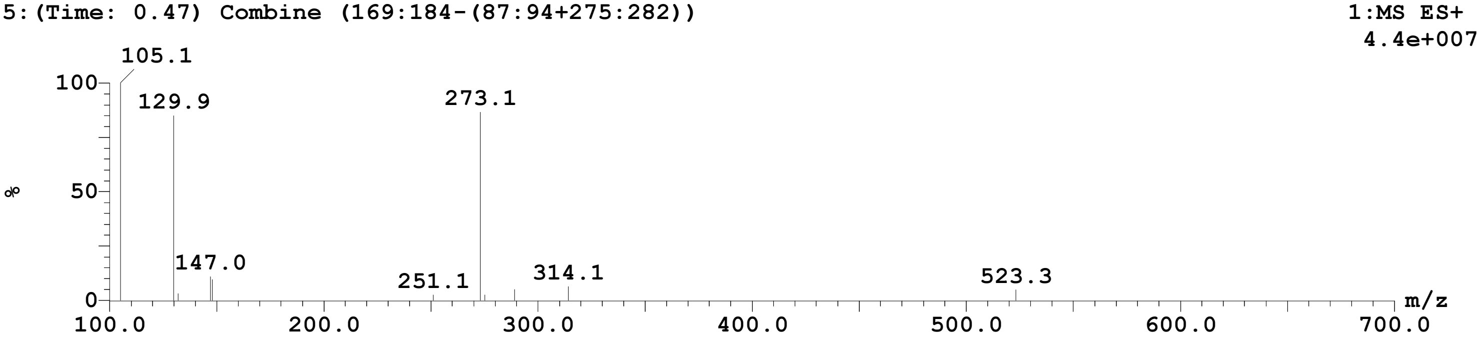

Peak ID **Time**
5 0.47

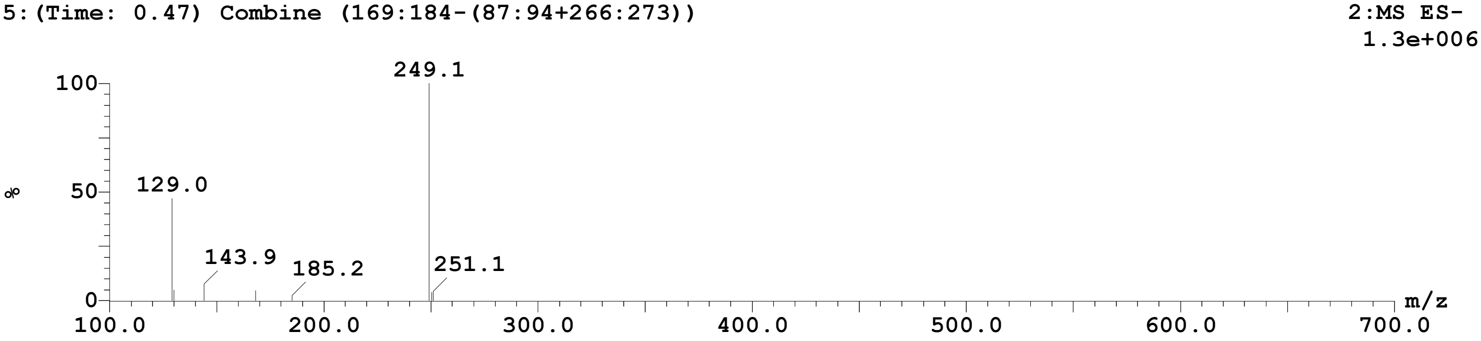

Peak ID **Time**
6 0.73

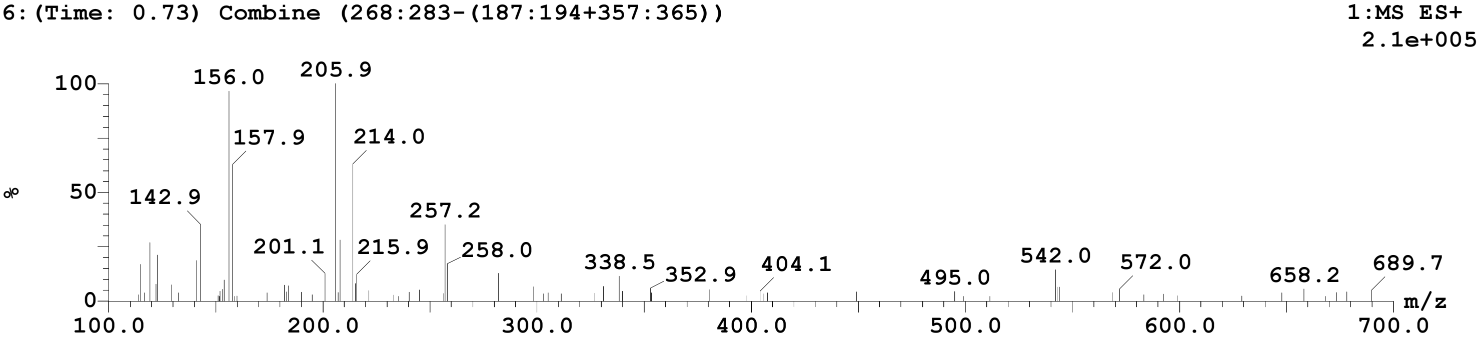

File:13zo191l2 Vial:5:52 ID:E9
Method:C:\MASSLYNX\1minLC_MS.olp

Peak ID **Time**
7 0.89

7: (Time: 0.89) Combine (327:342-246:253) 1:MS ES+ 5.1e+006

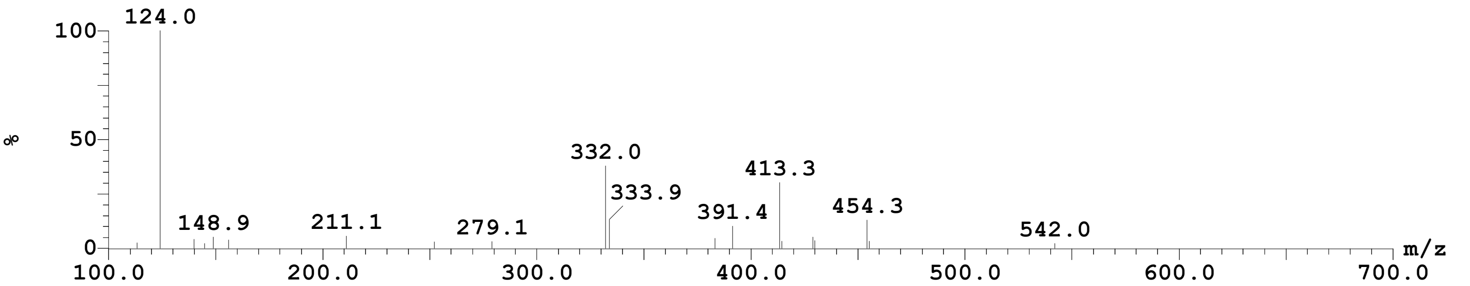

Peak ID **Time**
7 0.89

7: (Time: 0.89) Combine (326:341-245:253) 2:MS ES- 6.0e+004

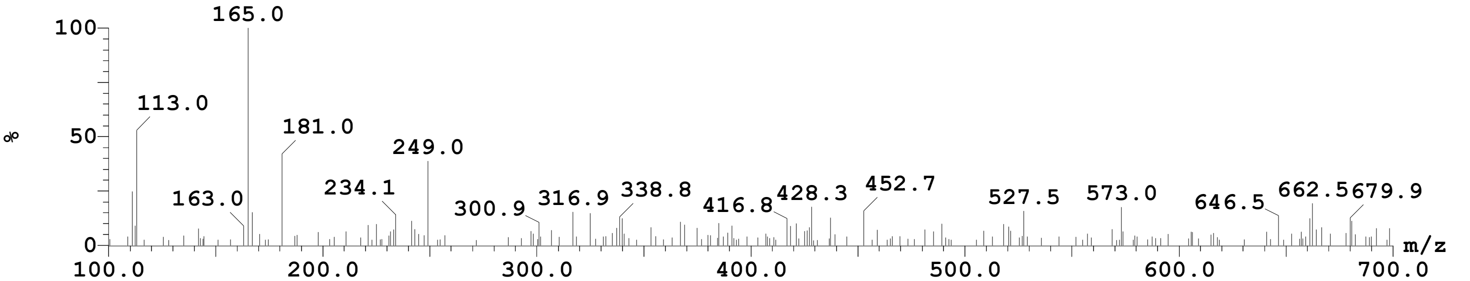

SYNGENTA SQD OPEN ACCESS LC-MS Page 1

File:13zo240l2 Vial:5:53 ID: **E10**

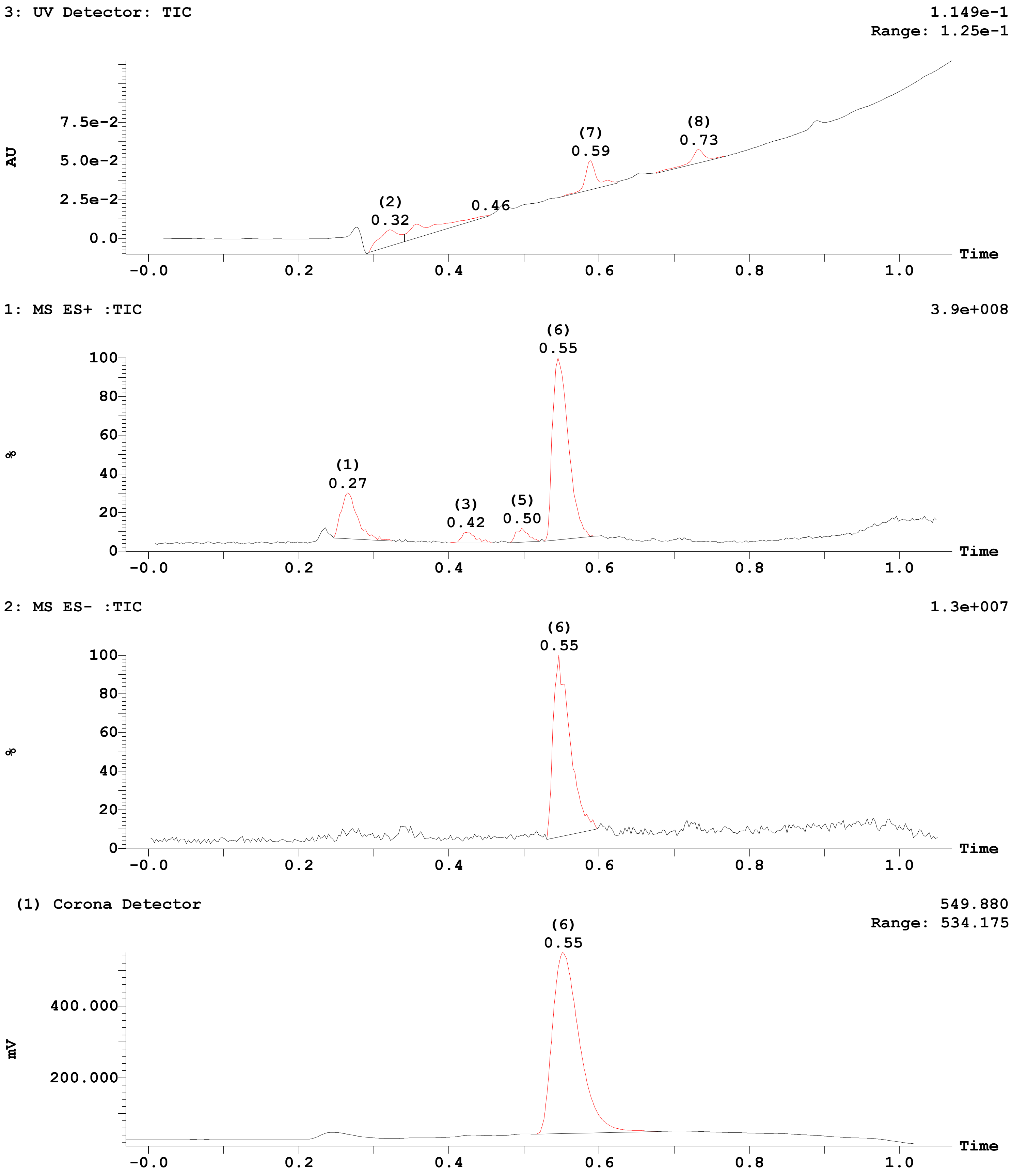

File:13zo240l2 Vial:5:53 ID:E10
Method:C:\MASSLYNX\1minLC_MS.olp

Peak ID **Time**
1 0.27

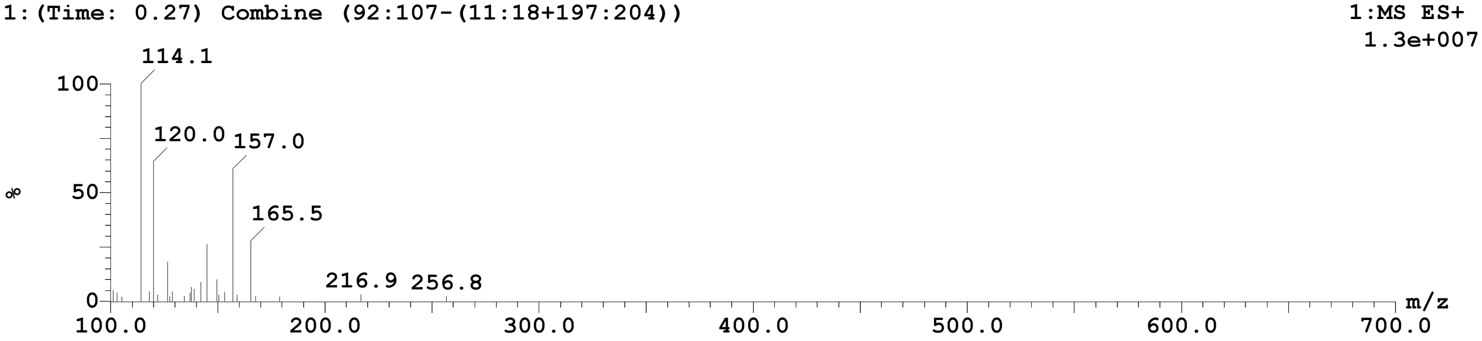

Peak ID **Time**
2 0.32

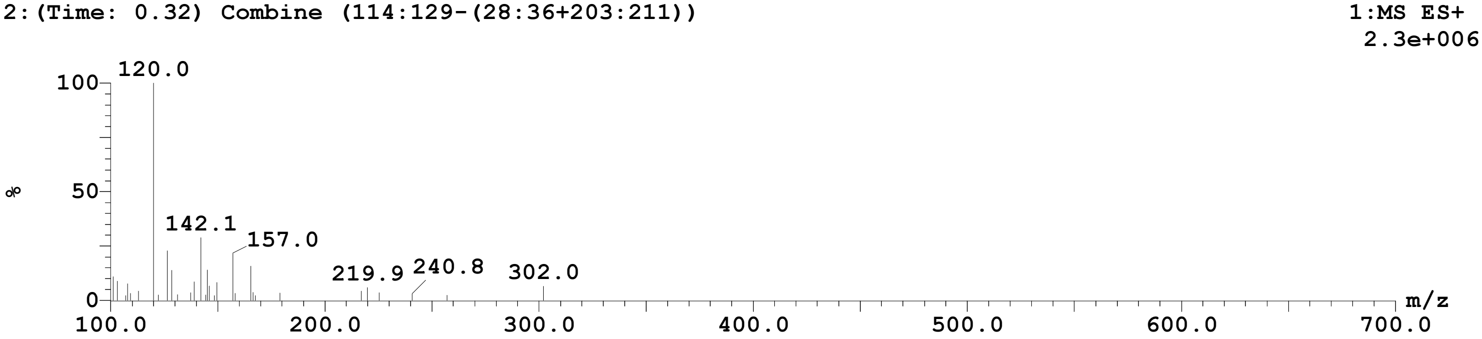

Peak ID **Time**
3 0.42

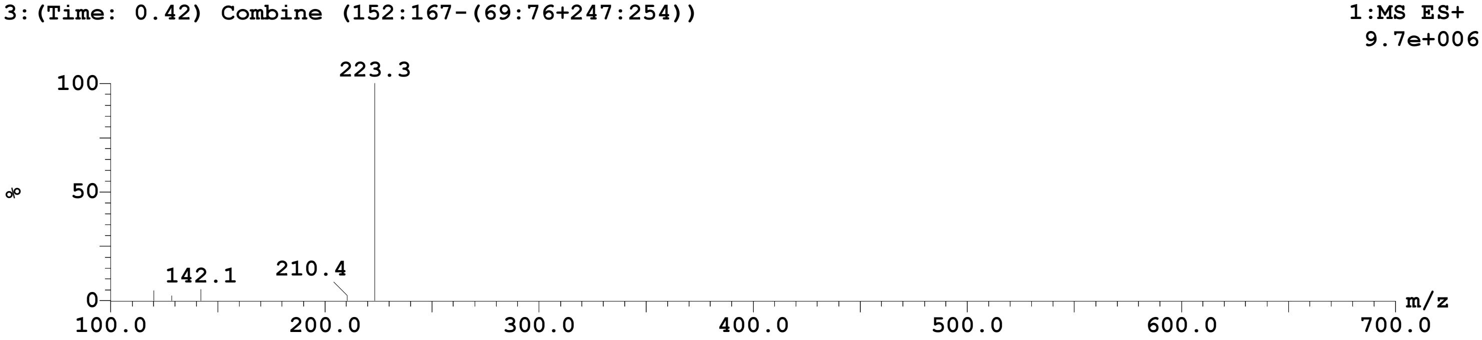

Peak ID **Time**
4 0.46

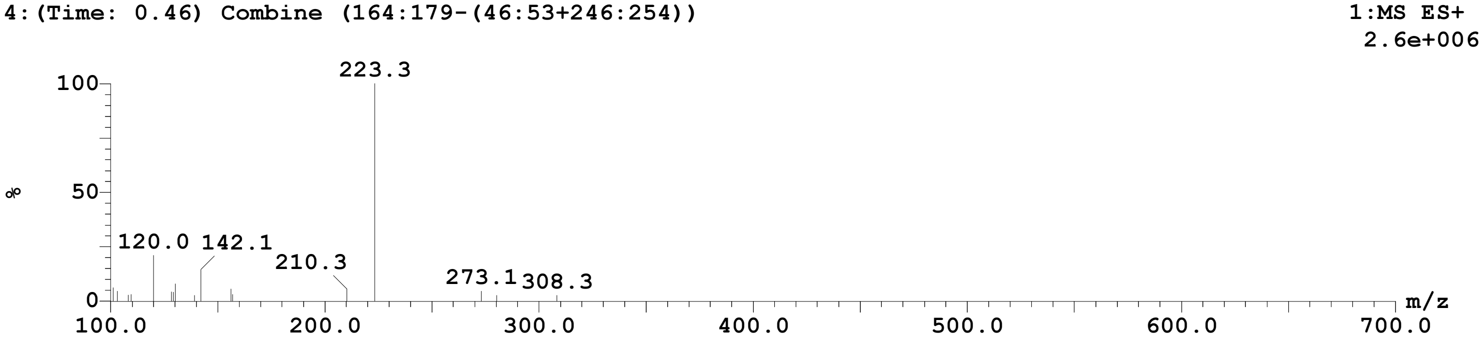

File:13zo240l2 Vial:5:53 ID:E10
Method:C:\MASSLYNX\1minLC_MS.olp

Peak ID **Time**
5 0.50

5:(Time: 0.50) Combine (179:194-(99:106+271:278)) 1:MS ES+
1.3e+007

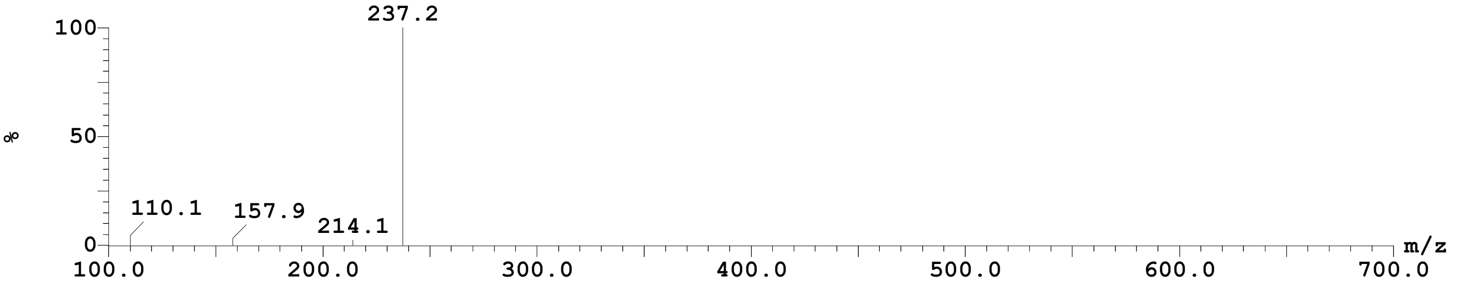

Peak ID **Time**
6 0.55

6:(Time: 0.55) Combine (197:212-(116:123+299:306)) 1:MS ES+
7.9e+007

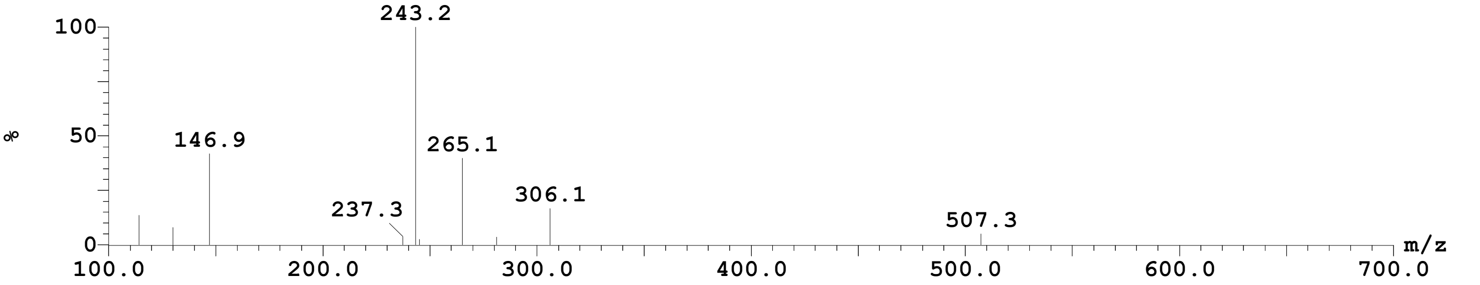

Peak ID **Time**
6 0.55

6:(Time: 0.55) Combine (198:212-(117:124+299:306)) 2:MS ES-
4.4e+006

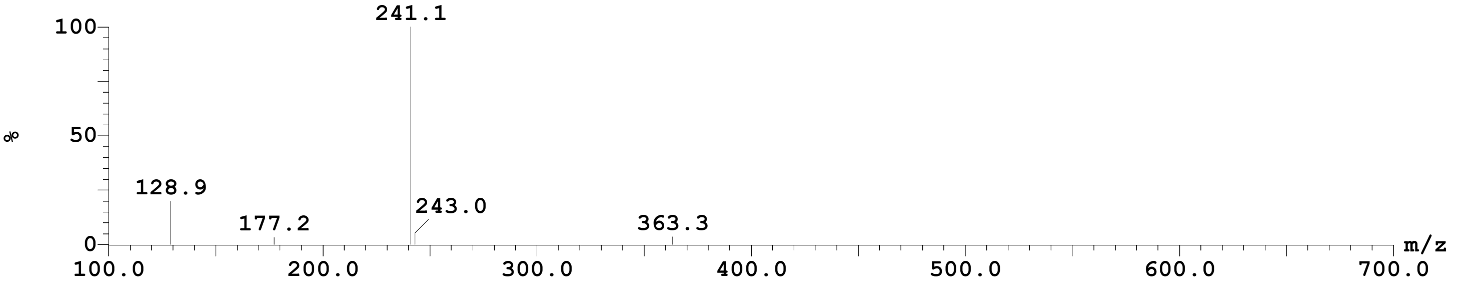

Peak ID **Time**
7 0.59

7:(Time: 0.59) Combine (214:229-(123:131+310:317)) 1:MS ES+
9.7e+006

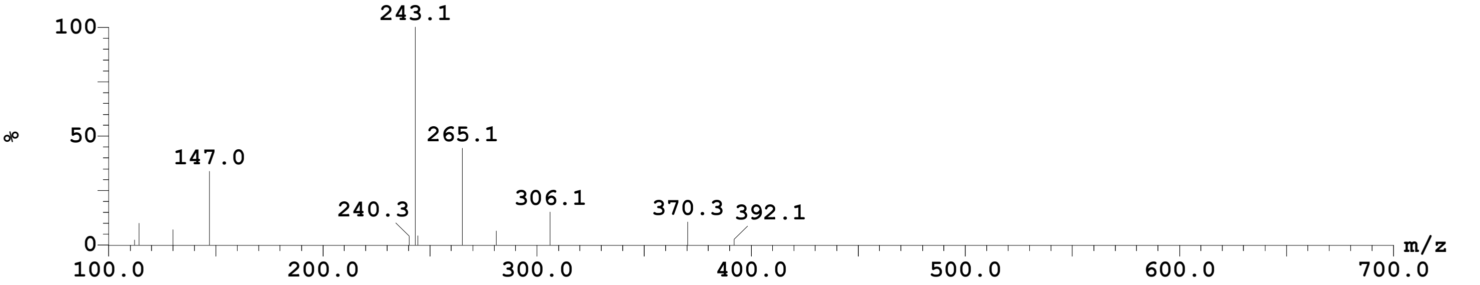

File:13zo240l2 Vial:5:53 ID:E10
Method:C:\MASSLYNX\1minLC_MS.olp

Peak ID **Time**
7 0.59

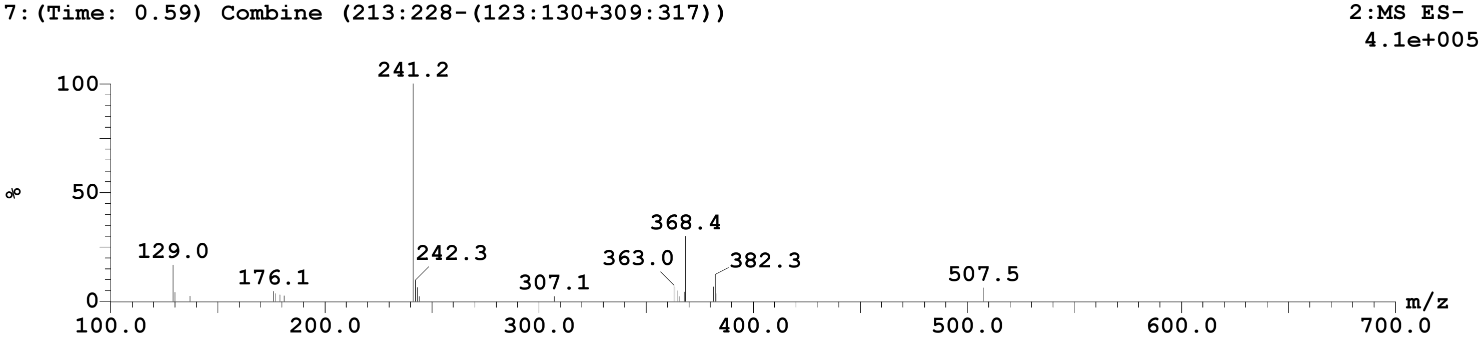

Peak ID **Time**
8 0.73

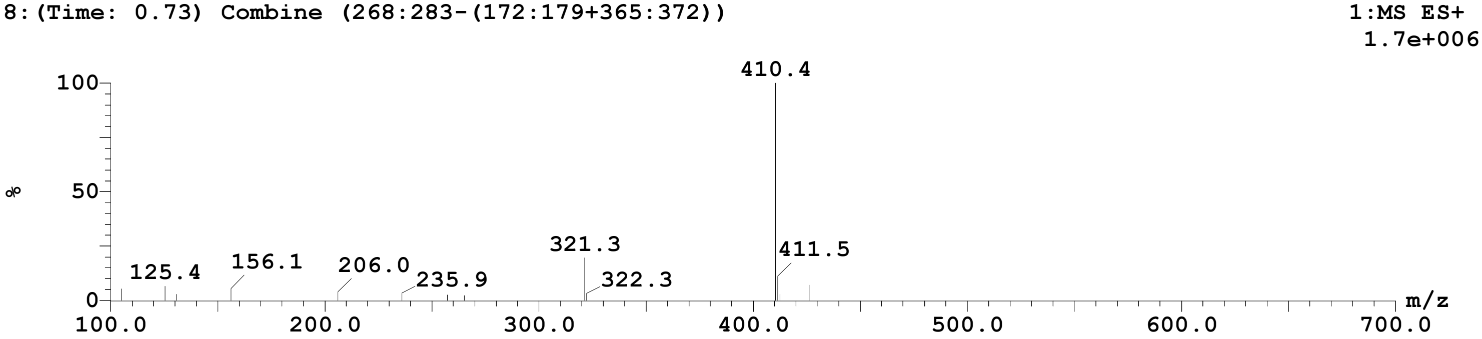

SYNGENTA SQD OPEN ACCESS LC-MS Page 1

File:13zo308l2 Vial:5:47 ID: **F2**

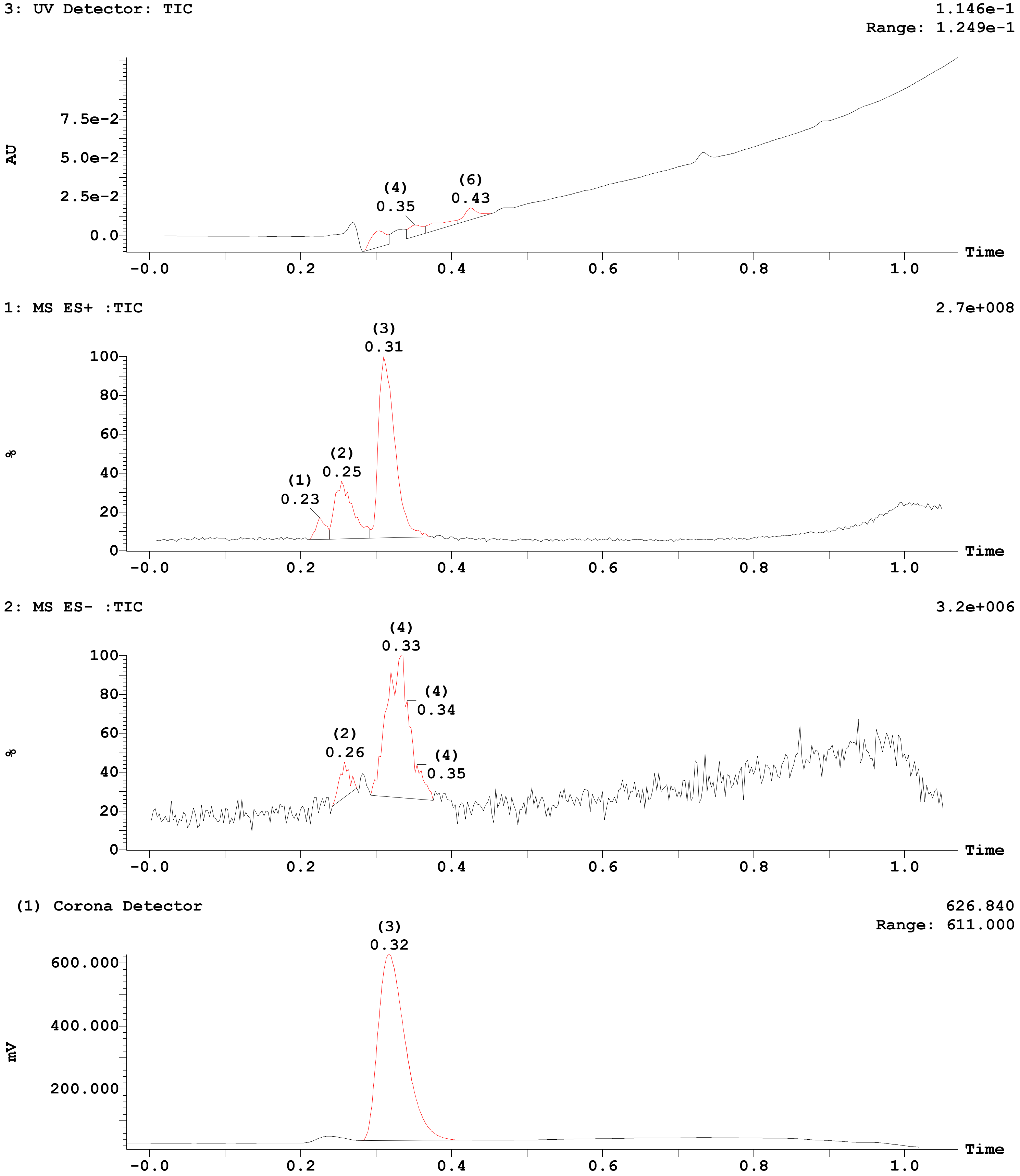

File:13zo308l2 Vial:5:47 ID:F2
Method:C:\MASSLYNX\1minLC_MS.olp

Peak ID	Time
1	0.23

1:(Time: 0.23) Combine (77:92-(1:5+165:172)) 1:MS ES+ 4.5e+006

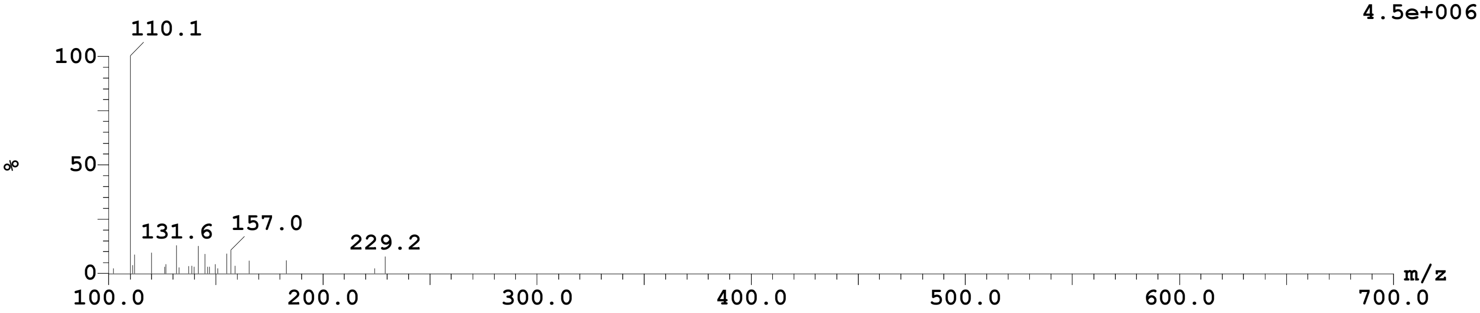

Peak ID	Time
2	0.25

2:(Time: 0.25) Combine (89:103-(8:15+185:192)) 1:MS ES+ 8.3e+006

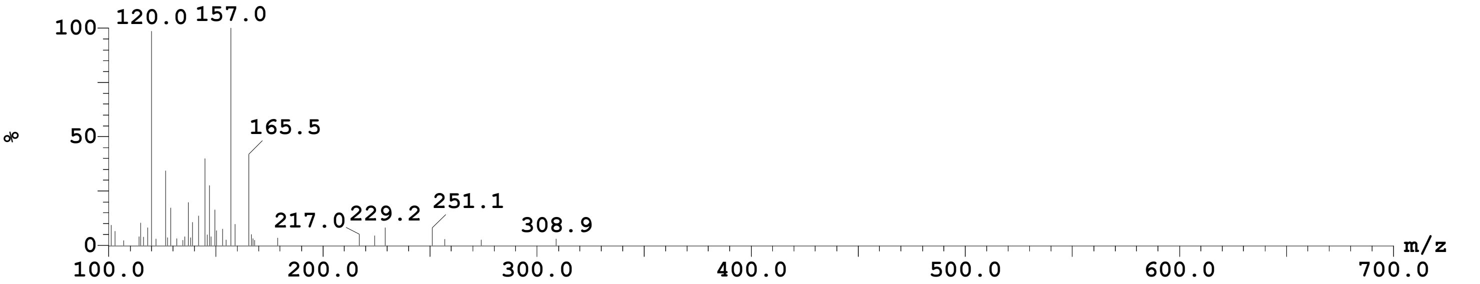

Peak ID	Time
2	0.25

2:(Time: 0.26) Combine (90:105-(9:16+178:185)) 2:MS ES- 1.5e+004

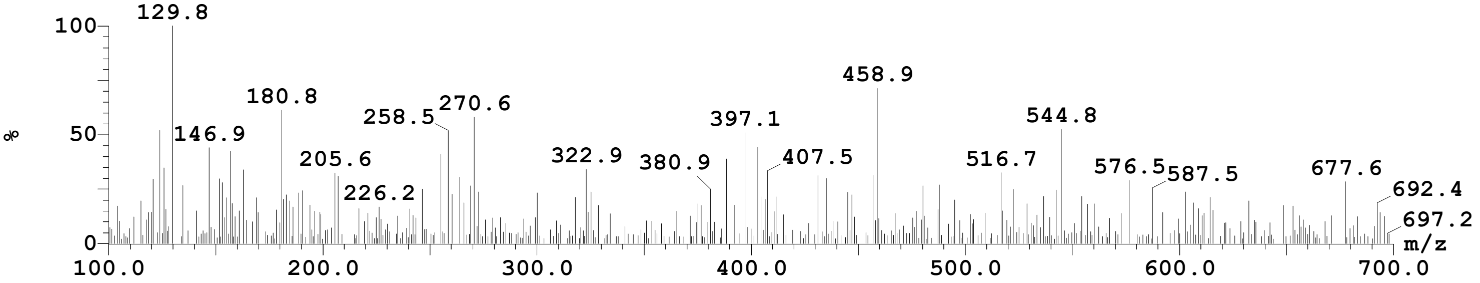

Peak ID	Time
3	0.31

3:(Time: 0.31) Combine (109:124-(28:35+215:222)) 1:MS ES+ 6.9e+007

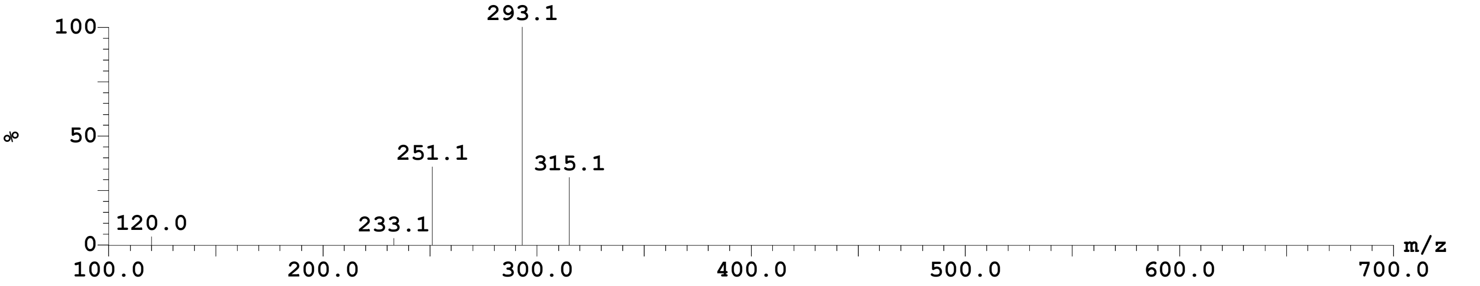

Peak ID **Time**
3 0.31

3:(Time: 0.30) Combine (107:122-(24:32+194:202)) 2:MS ES- 3.5e+004

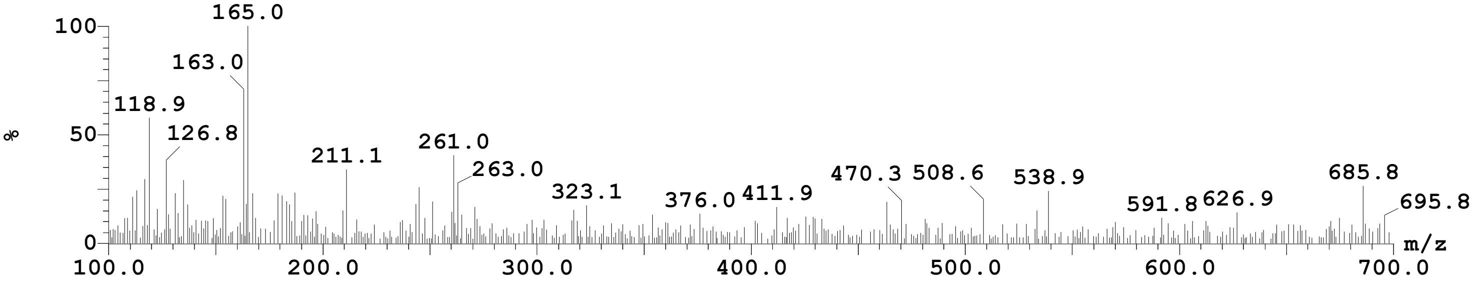

Peak ID **Time**
4 0.33

4:(Time: 0.35) Combine (125:140-(46:53+213:220)) 1:MS ES+ 9.3e+006

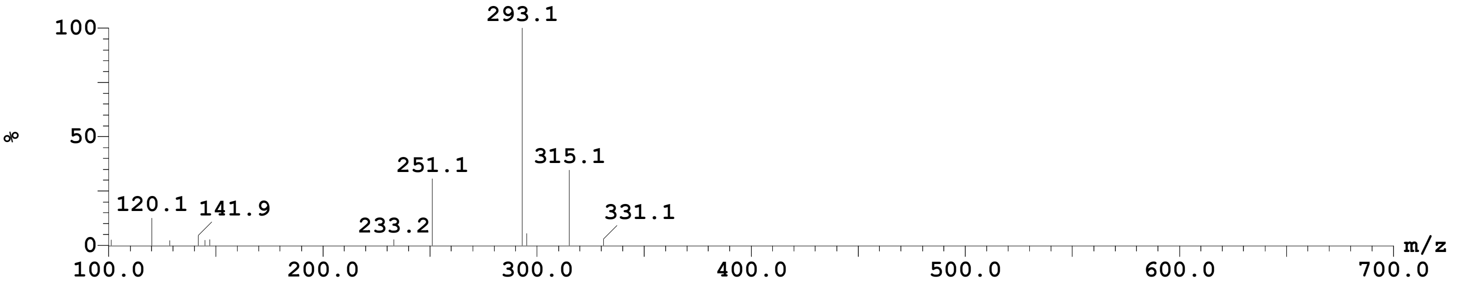

Peak ID **Time**
4 0.33

4:(Time: 0.33) Combine (118:132-(28:35+216:223)) 2:MS ES- 2.3e+005

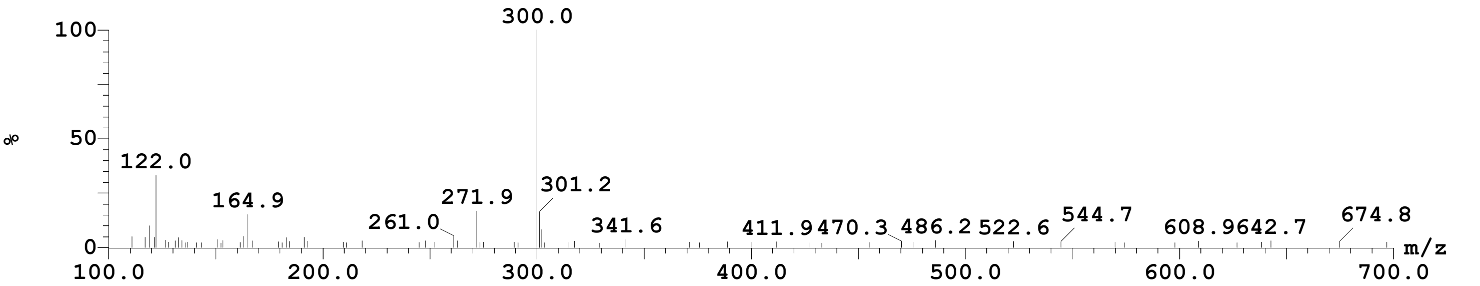

Peak ID **Time**
5 0.41

5:(Time: 0.41) Combine (146:161-(55:63+229:236)) 1:MS ES+ 4.8e+005

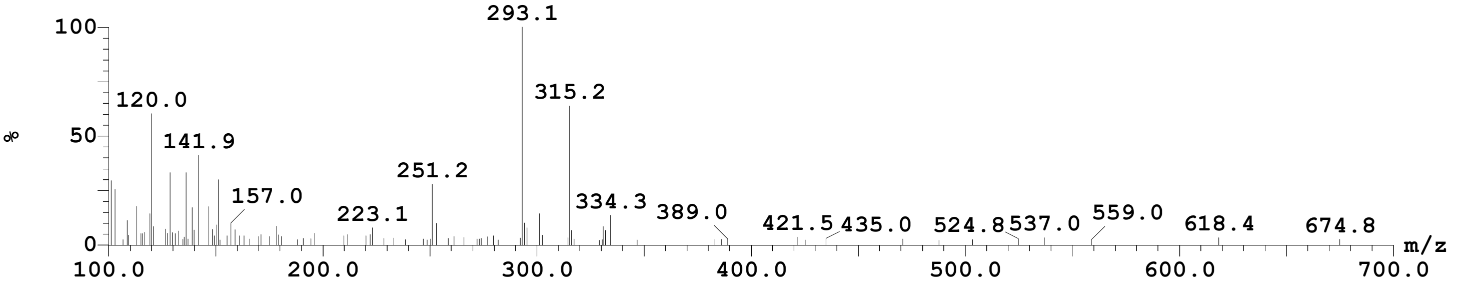

File:13zo308I2 Vial:5:47 ID:F2
Method:C:\MASSLYNX\1minLC_MS.olp

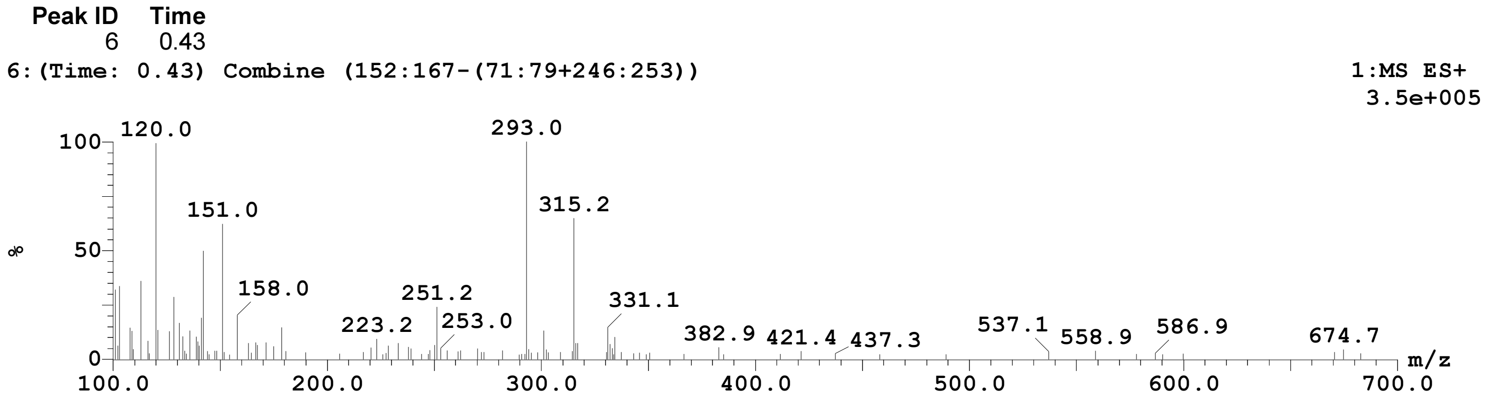

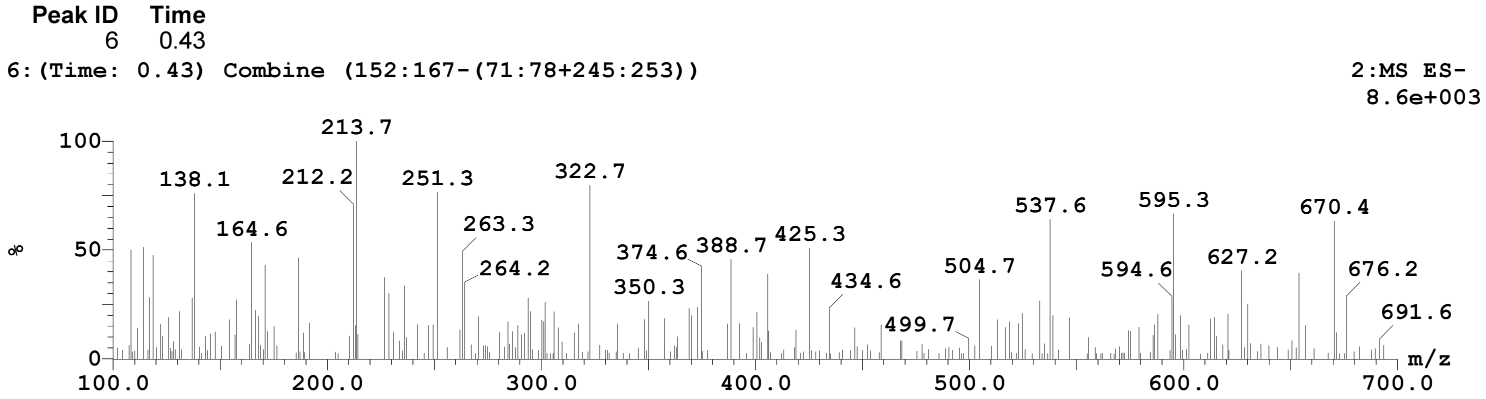

SYNGENTA SQD OPEN ACCESS LC-MS Page 1
File:13zp848l1 Vial:5:48 ID: **F3**

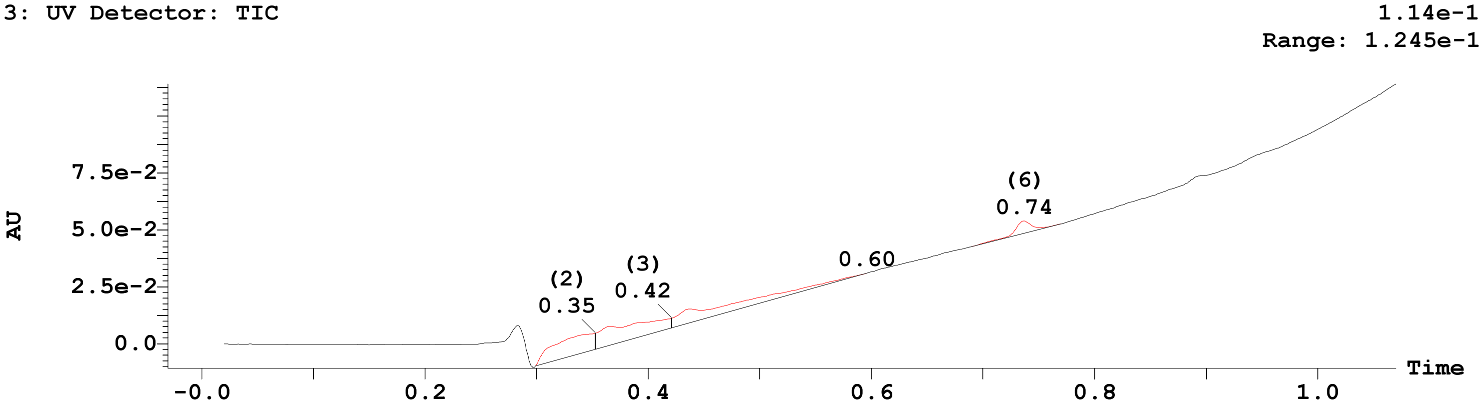

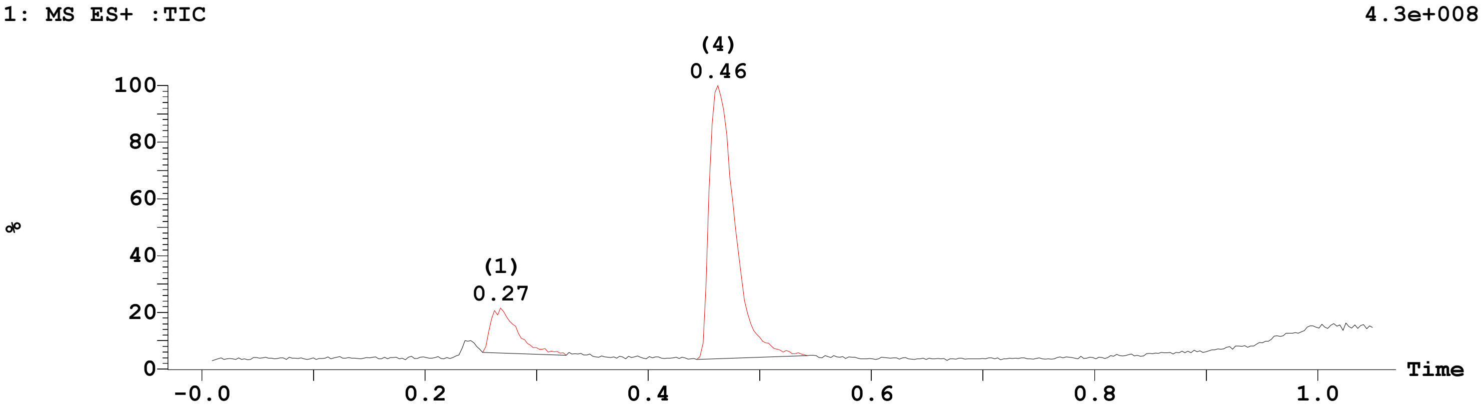

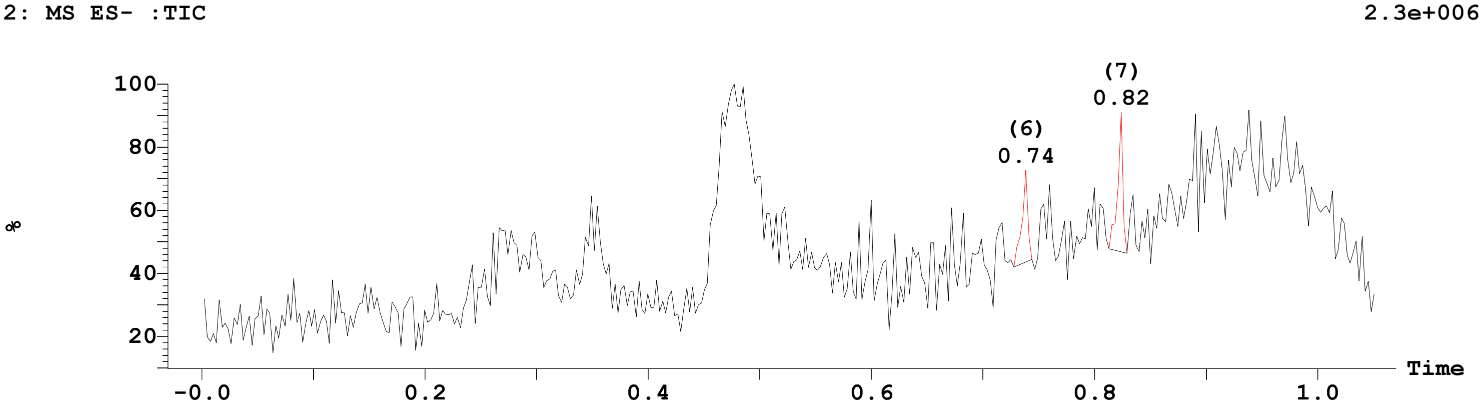

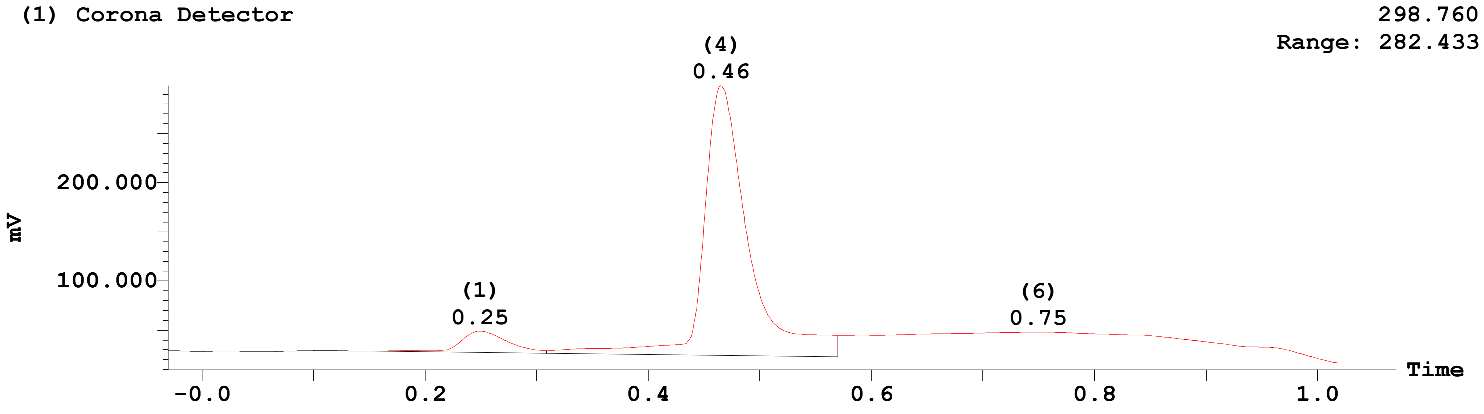

File:13zp848l1 Vial:5:48 ID:F3
Method:C:\MASSLYNX\1minLC_MS.olp

Peak ID **Time**
1 0.27

1:(Time: 0.27) Combine (94:108-(13:20+198:205)) 1:MS ES+

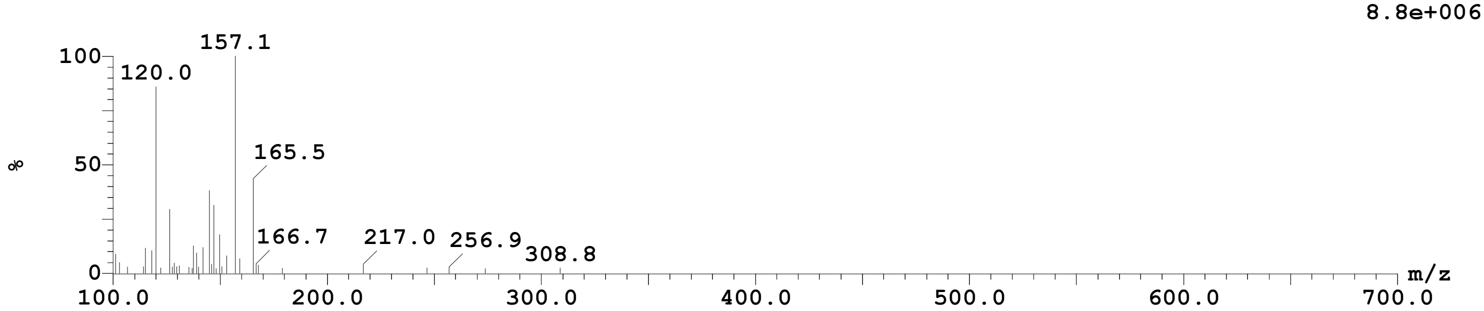

Peak ID **Time**
2 0.35

2:(Time: 0.35) Combine (125:140-(30:38+208:215)) 1:MS ES+

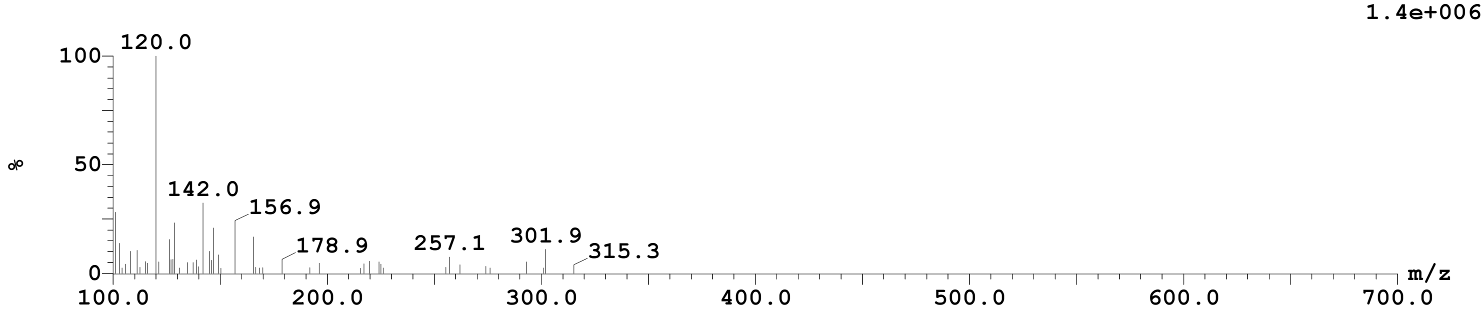

Peak ID **Time**
2 0.35

2:(Time: 0.35) Combine (125:140-(30:37+207:215)) 2:MS ES-

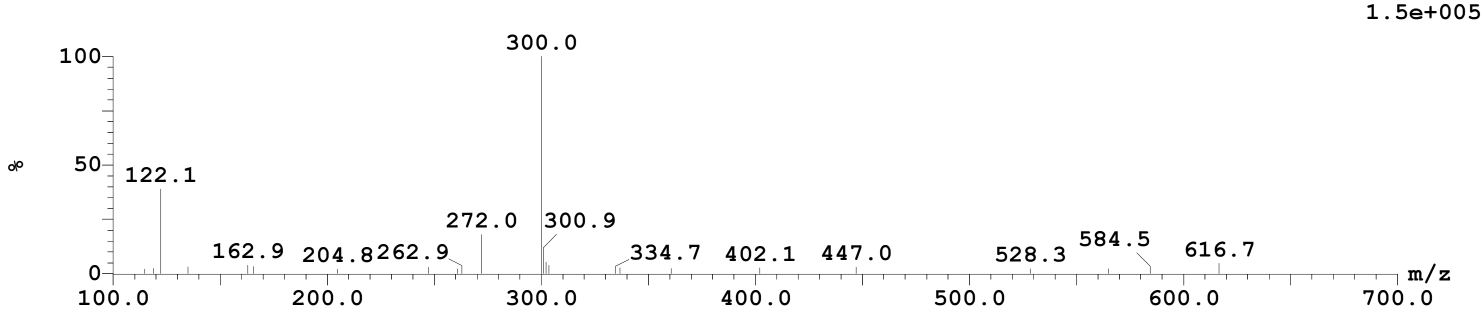

Peak ID **Time**
3 0.42

3:(Time: 0.42) Combine (151:166-(50:58+233:241)) 1:MS ES+

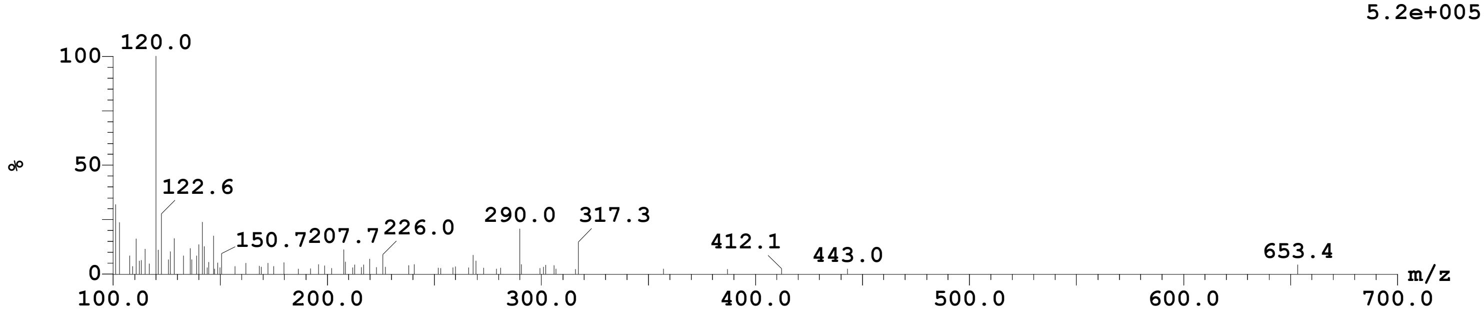

File:13zp848l1 Vial:5:48 ID:F3
Method:C:\MASSLYNX\1minLC_MS.olp

Peak ID 3 **Time** 0.42

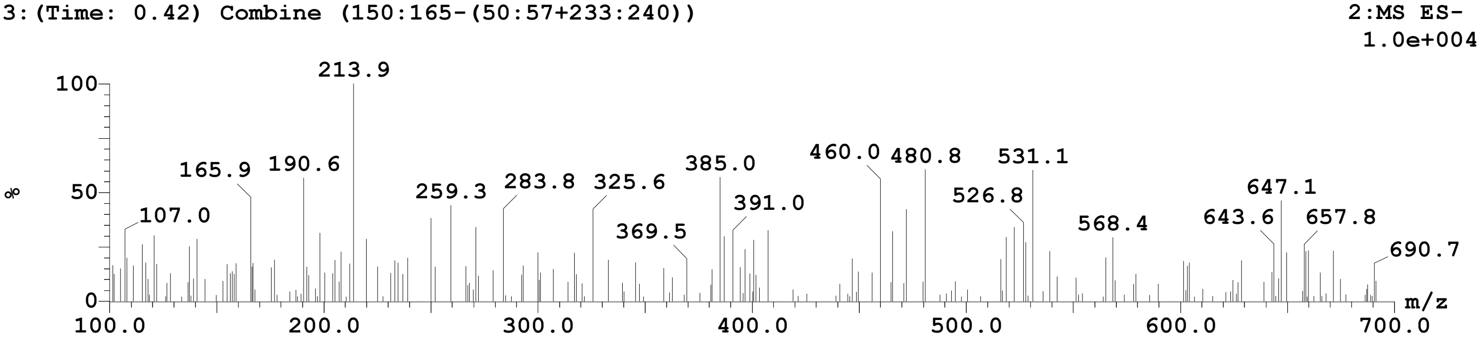

Peak ID 4 **Time** 0.46

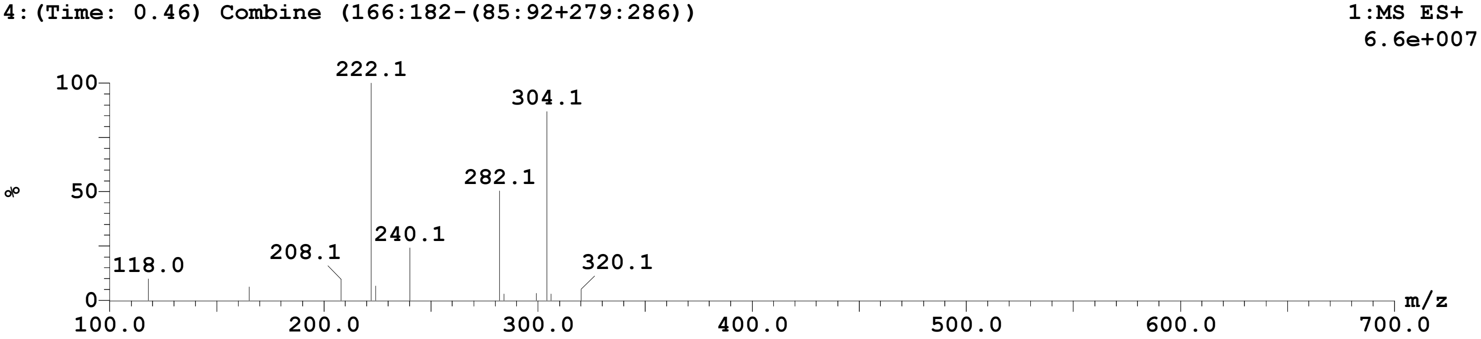

Peak ID 5 **Time** 0.60

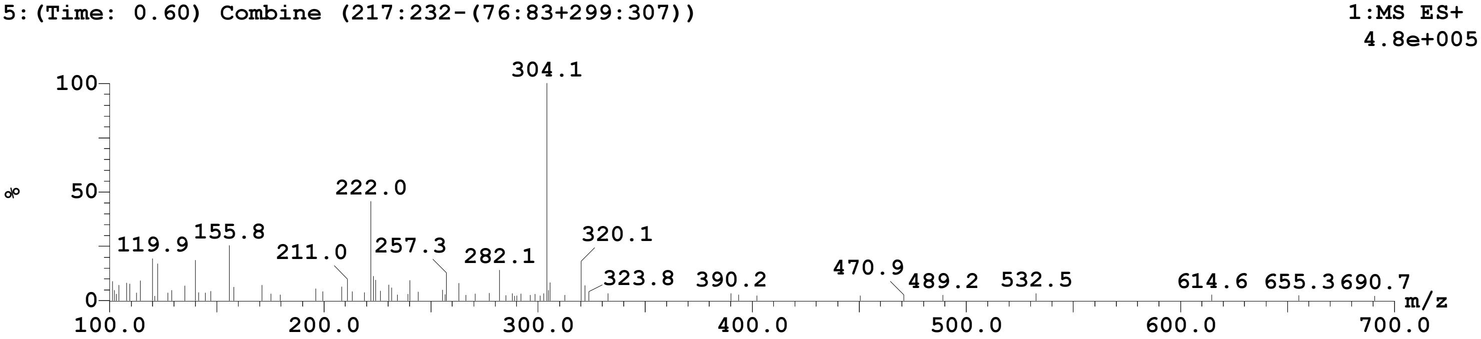

Peak ID 6 **Time** 0.74

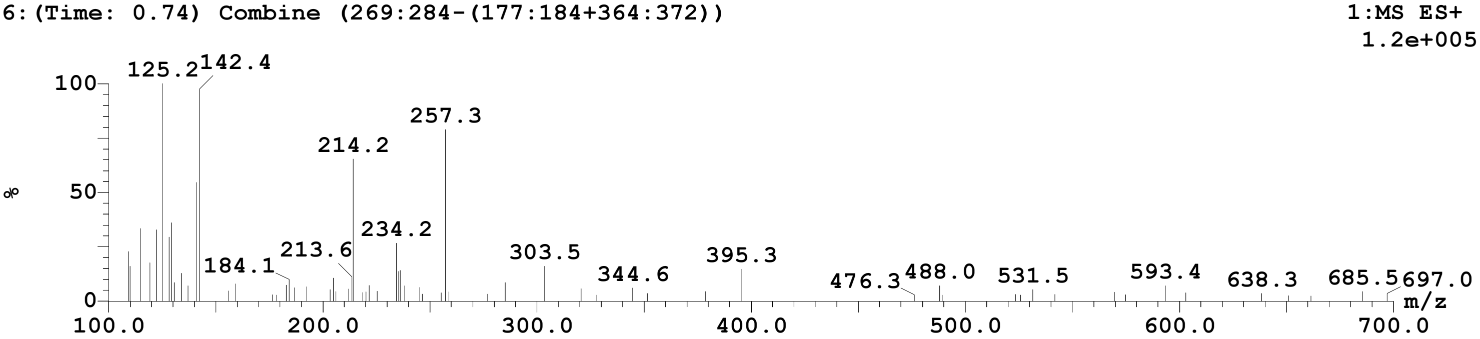

File:13zp848l1 Vial:5:48 ID:F3
Method:C:\MASSLYNX\1minLC_MS.olp

Peak ID 6 **Time** 0.74

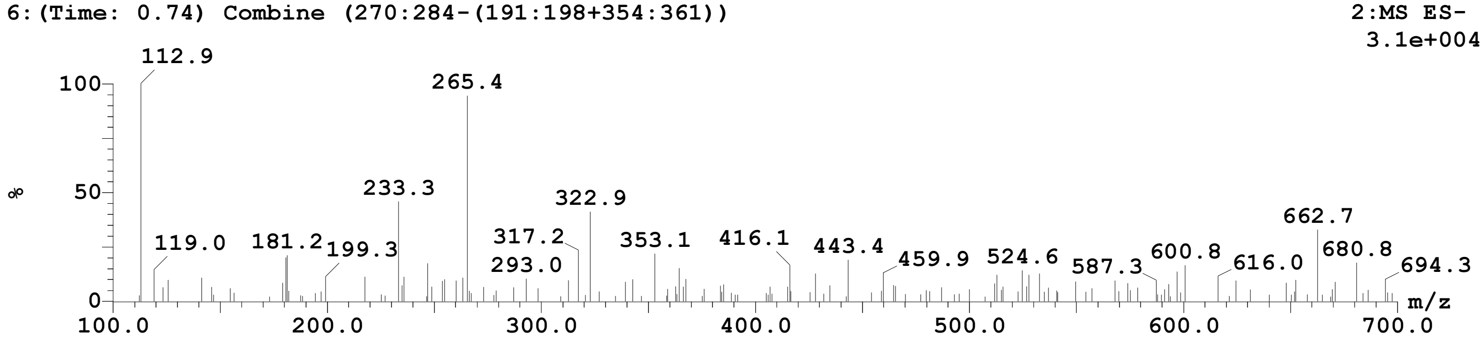

Peak ID 7 **Time** 0.82

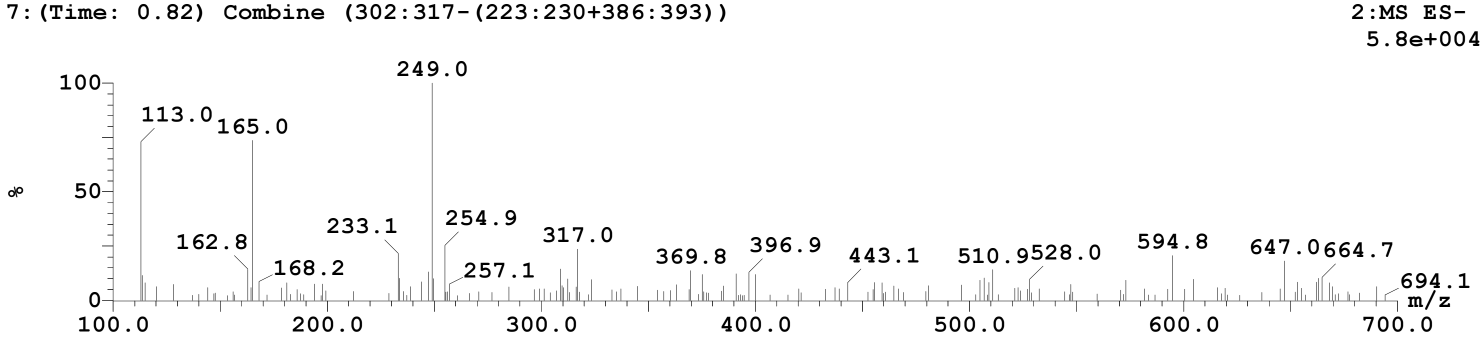

SYNGENTA SQD OPEN ACCESS LC-MS Page 1
File:13zp860l1 Vial:5:46 ID: **F4**

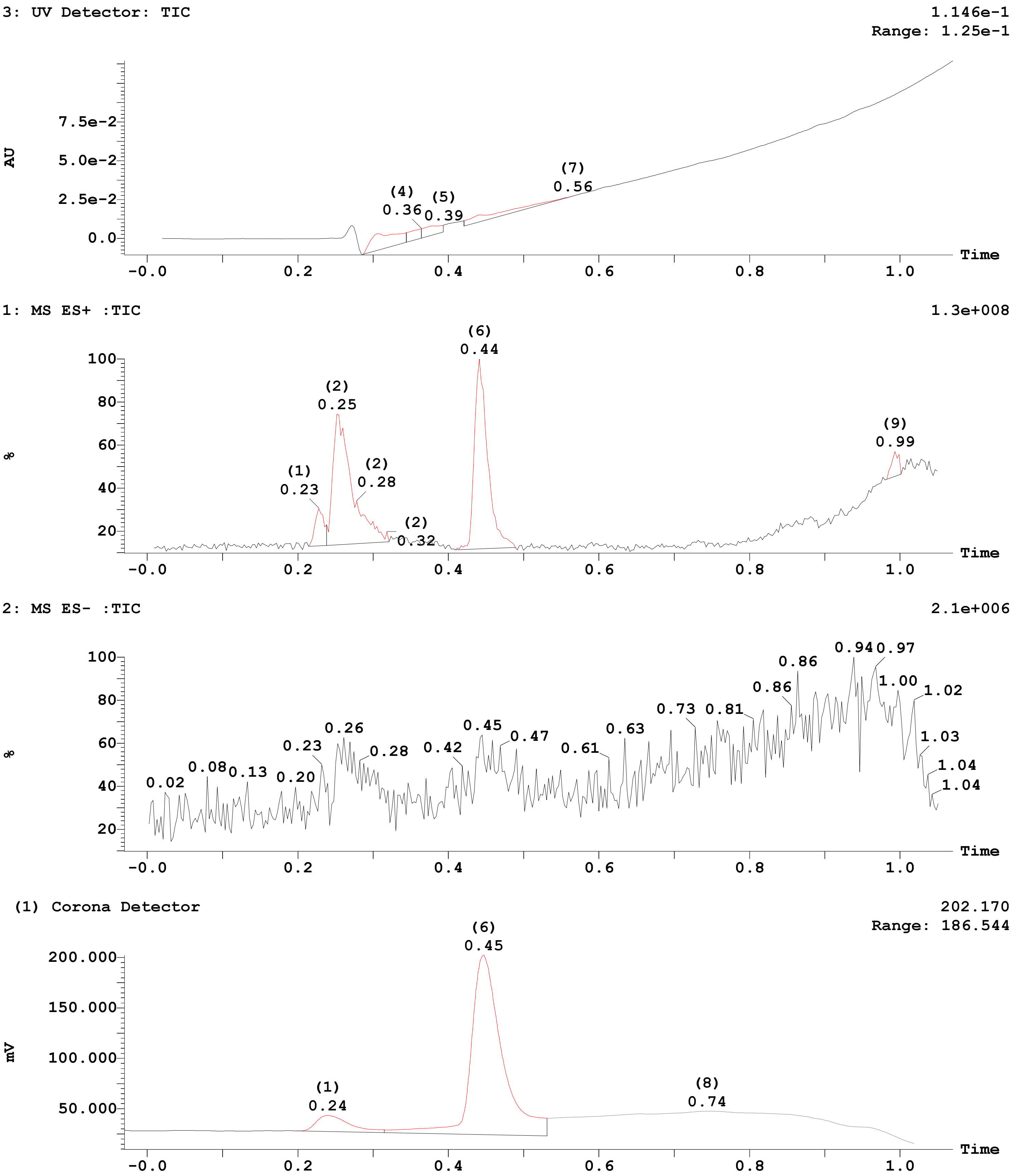

File:13zp860l1 Vial:5:46

Peak ID **Time**
1 0.23

1:(Time: 0.23) Combine (78:93-(1:6+165:172)) 1:MS ES+
3.9e+006

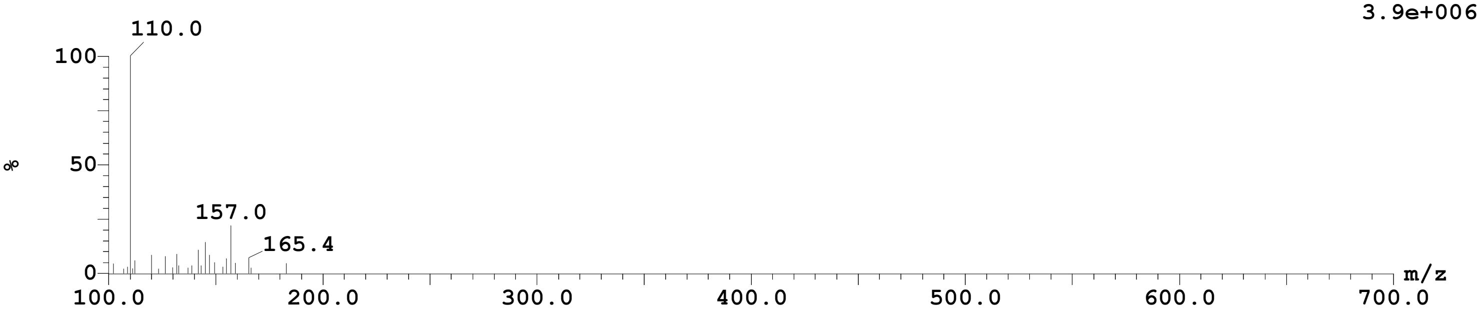

Peak ID **Time**
2 0.25

2:(Time: 0.25) Combine (88:103-(8:15+196:203)) 1:MS ES+
8.6e+006

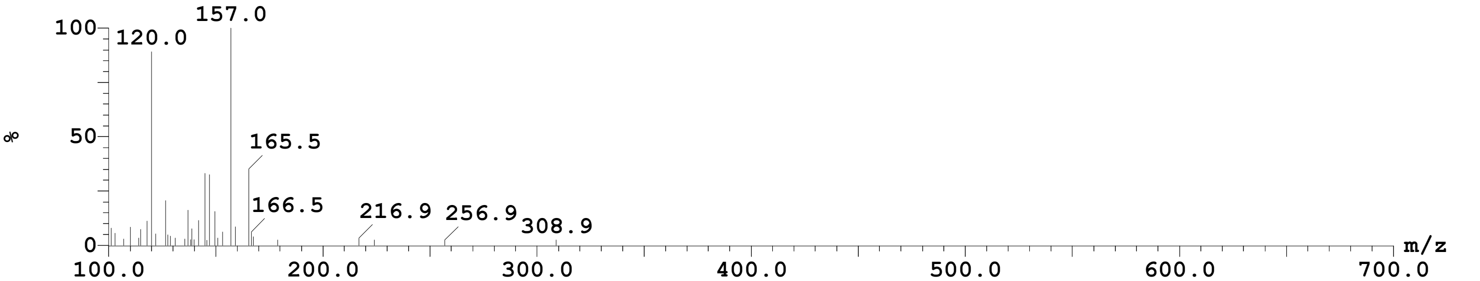

Peak ID **Time**
3 0.34

3:(Time: 0.34) Combine (122:137-(26:33+205:212)) 1:MS ES+
1.5e+006

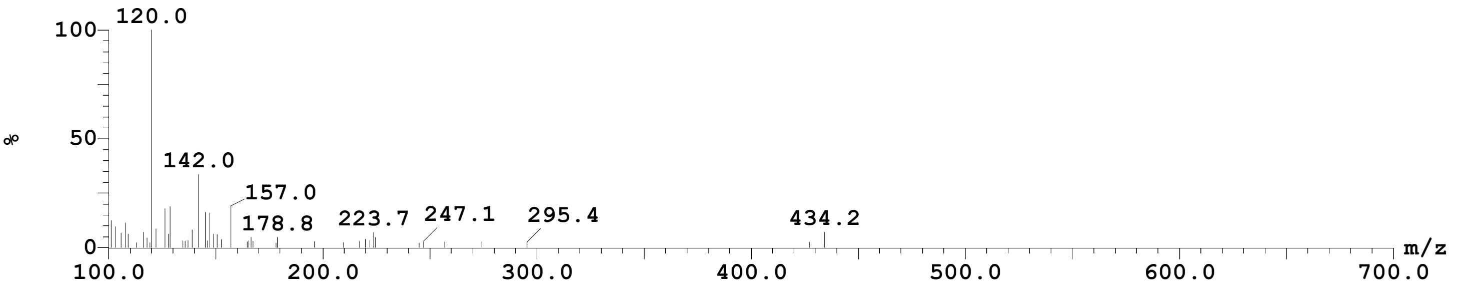

Peak ID **Time**
3 0.34

3:(Time: 0.34) Combine (122:137-(25:33+204:212)) 2:MS ES-
1.4e+004

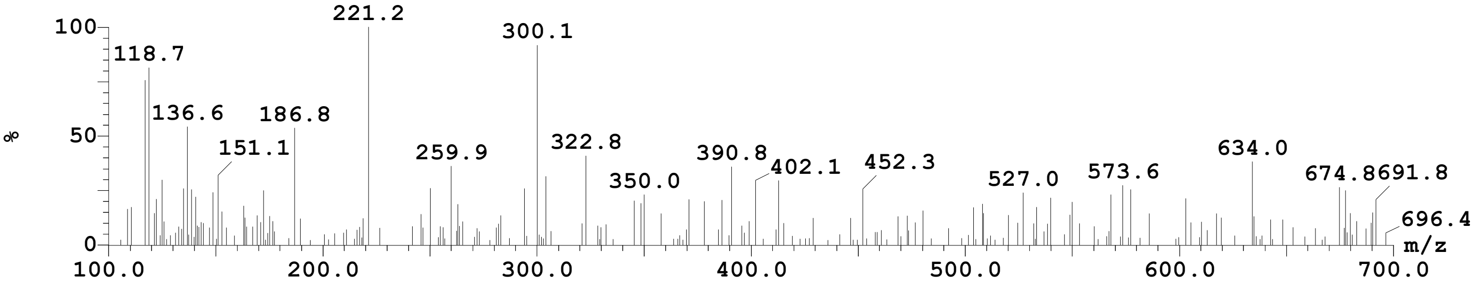

File:13zp860l1 Vial:5:46 ID:F4
Method:C:\MASSLYNX\1minLC_MS.olp

Peak ID 4 **Time** 0.36

4:(Time: 0.36) Combine (130:145-(47:55+212:220)) 1:MS ES+ 1.1e+006

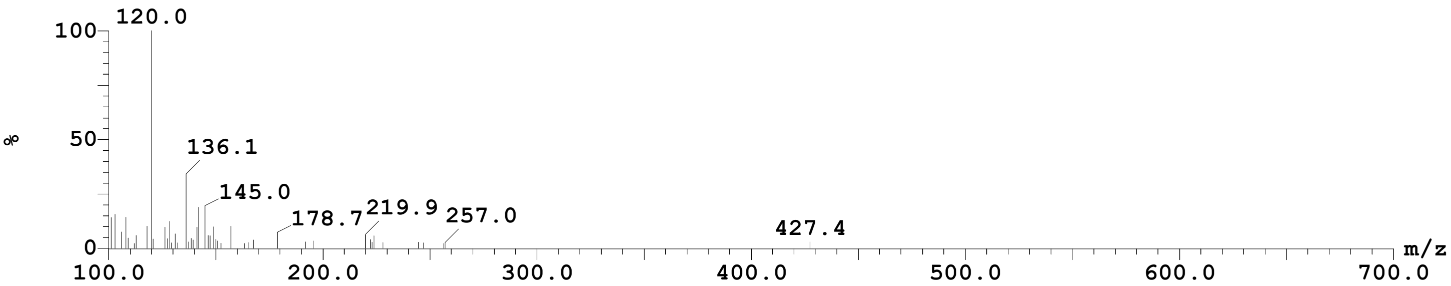

Peak ID 5 **Time** 0.39

5:(Time: 0.39) Combine (141:156-(55:62+223:231)) 1:MS ES+ 5.6e+005

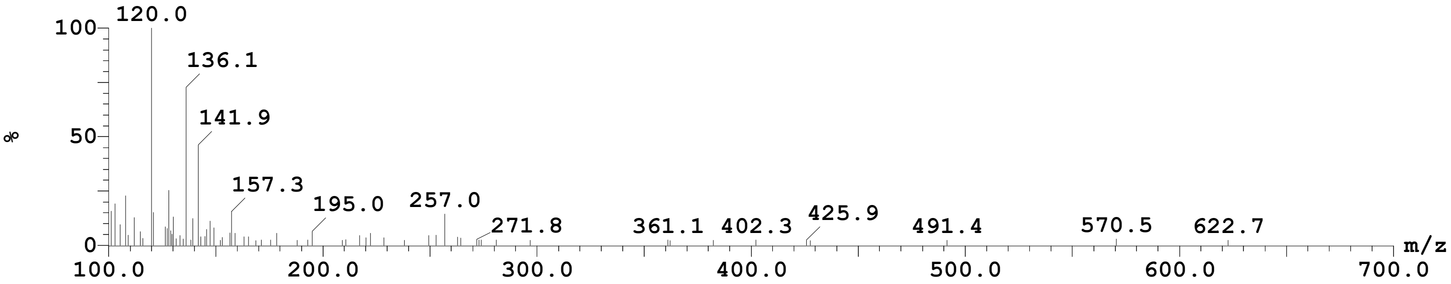

Peak ID 5 **Time** 0.39

5:(Time: 0.39) Combine (140:155-(54:62+223:230)) 2:MS ES- 1.4e+004

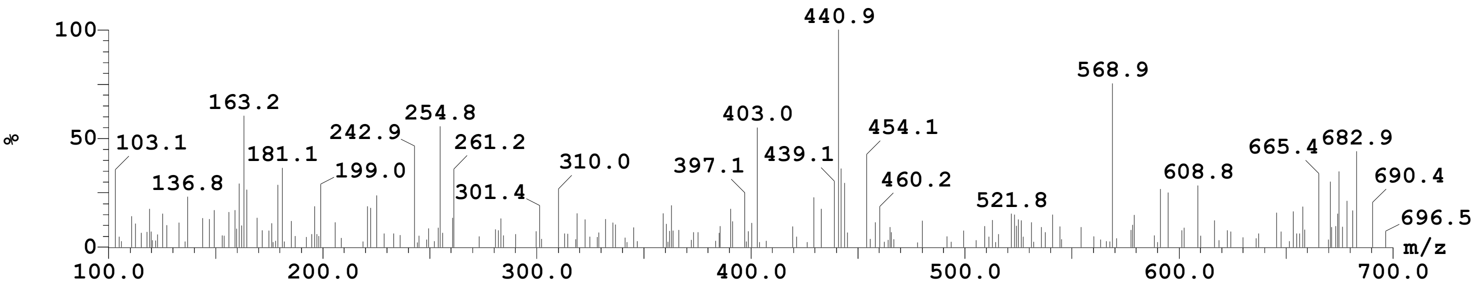

Peak ID 6 **Time** 0.44

6:(Time: 0.44) Combine (158:173-(72:79+259:266)) 1:MS ES+ 1.7e+007

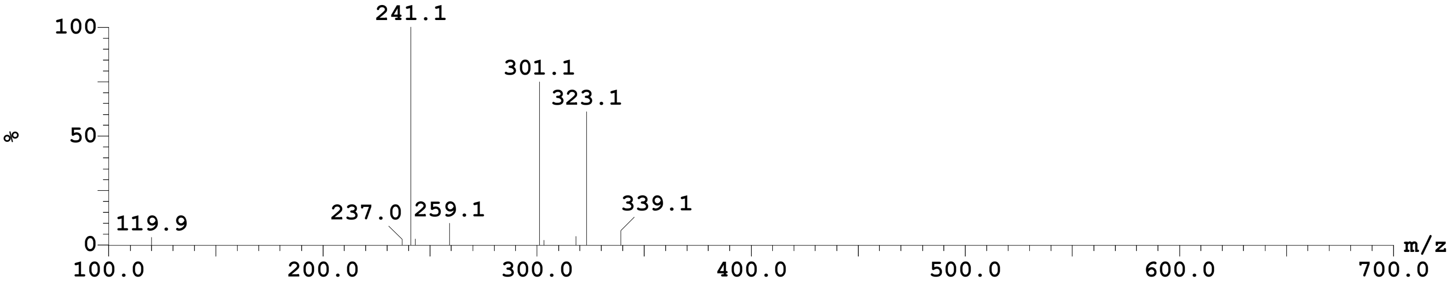

File:13zp860l1 Vial:5:46 ID:F4
Method:C:\MASSLYNX\1minLC_MS.olp

Peak ID	Time
7	0.56

7: (Time: 0.56) Combine (205:220-(76:83+287:295)) 1:MS ES+ 1.9e+005

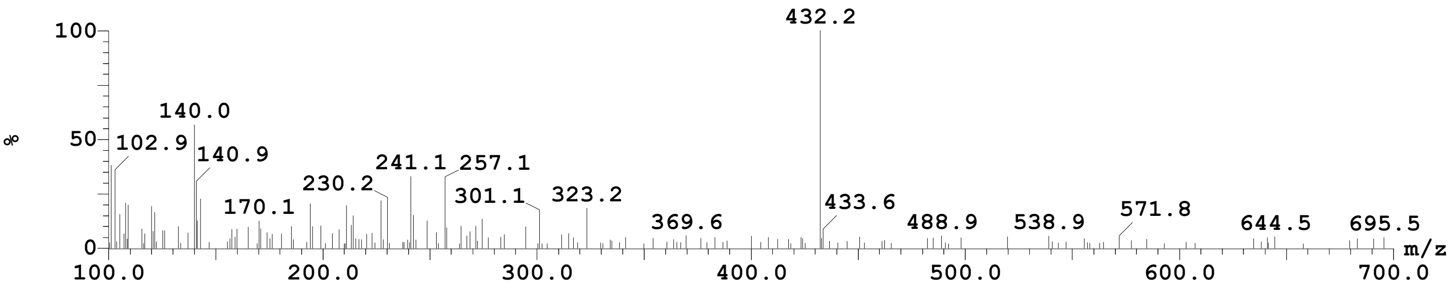

Peak ID	Time
9	0.99

9: (Time: 0.99) Combine (366:380-287:294) 1:MS ES+ 2.8e+007

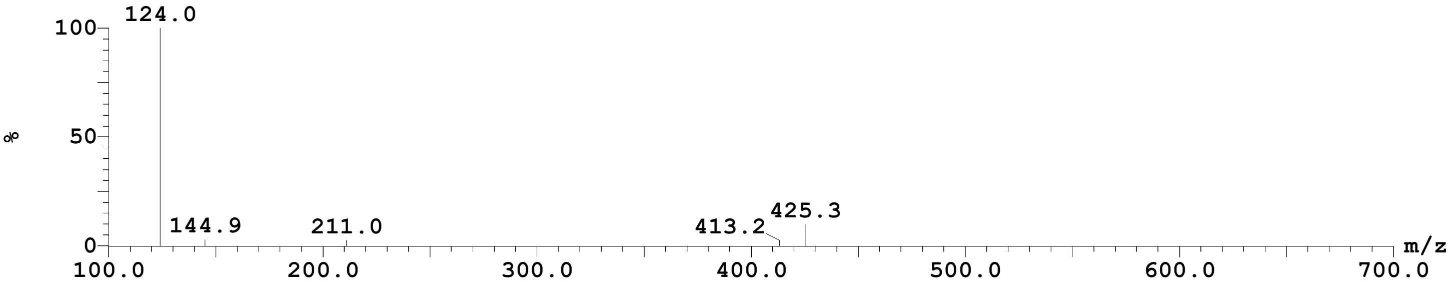

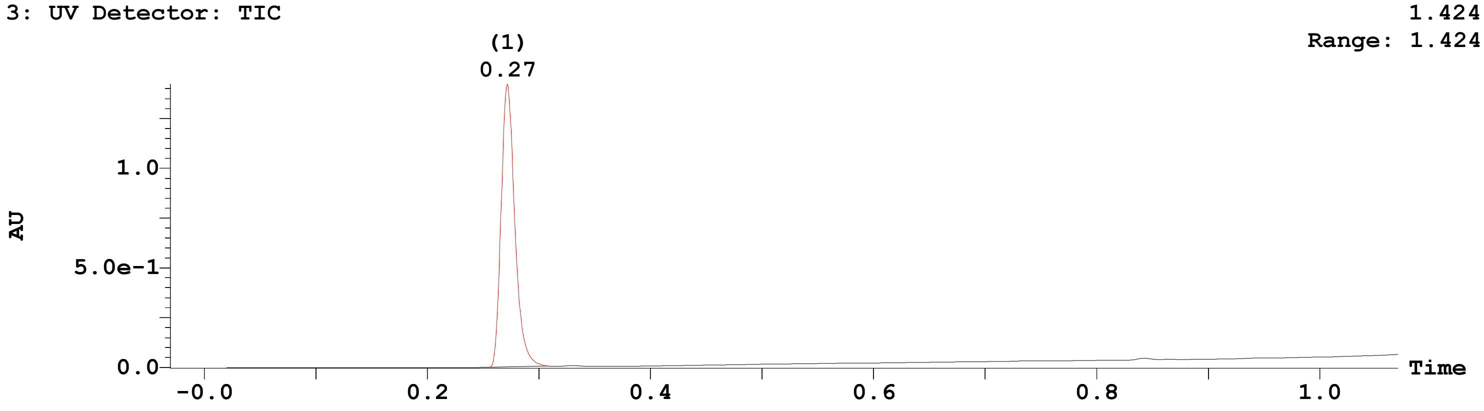
3: UV Detector: TIC
1.424
Range: 1.424
(1)
0.27
AU
1.0
5.0e-1
0.0
-0.0
0.2
0.4
0.6
0.8
1.0
Time

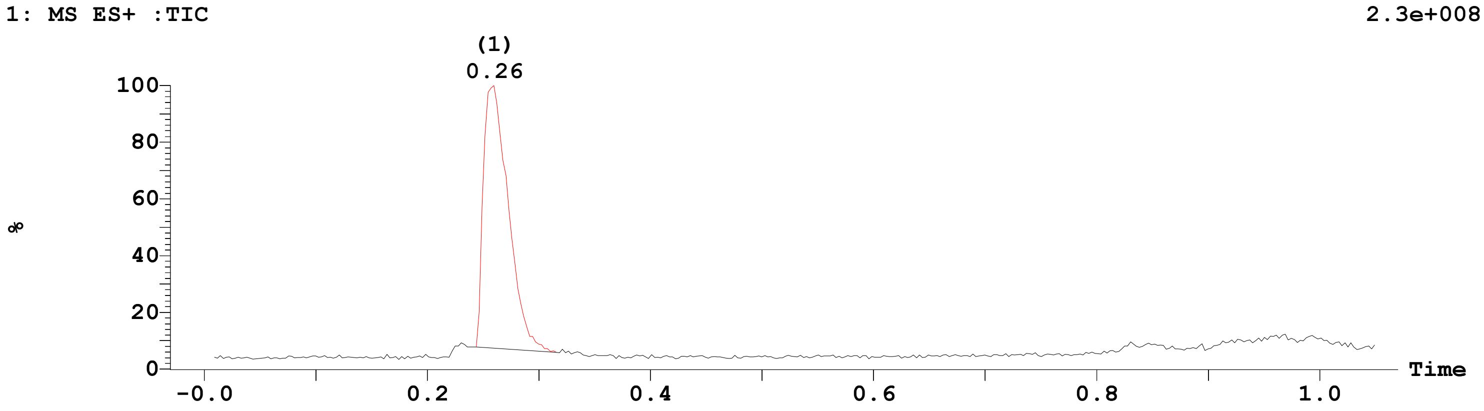
1: MS ES+ :TIC
2.3e+008
(1)
0.26
%
100
80
60
40
20
0
-0.0
0.2
0.4
0.6
0.8
1.0
Time

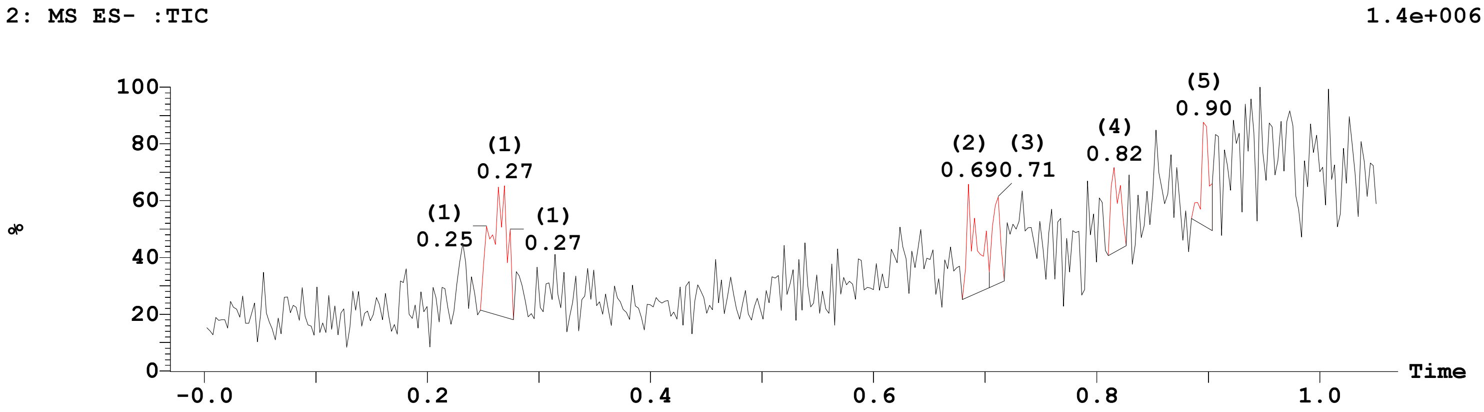
2: MS ES- :TIC
1.4e+006
(1)
0.25
(1)
0.27
(1)
0.27
(2) (3)
0.690.71
(4)
0.82
(5)
0.90
%
100
80
60
40
20
0
-0.0
0.2
0.4
0.6
0.8
1.0
Time

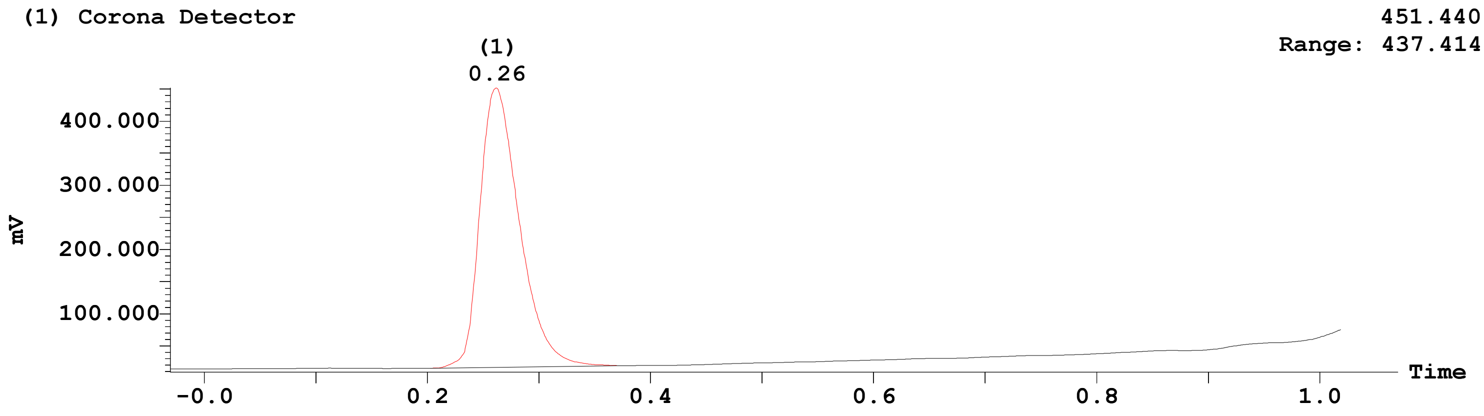
(1) Corona Detector
451.440
Range: 437.414
(1)
0.26
mV
400.000
300.000
200.000
100.000
-0.0
0.2
0.4
0.6
0.8
1.0
Time

File:13zo241l3 Vial:5:19 ID:F5
Method:C:\MASSLYNX\1minLC_MS.olp

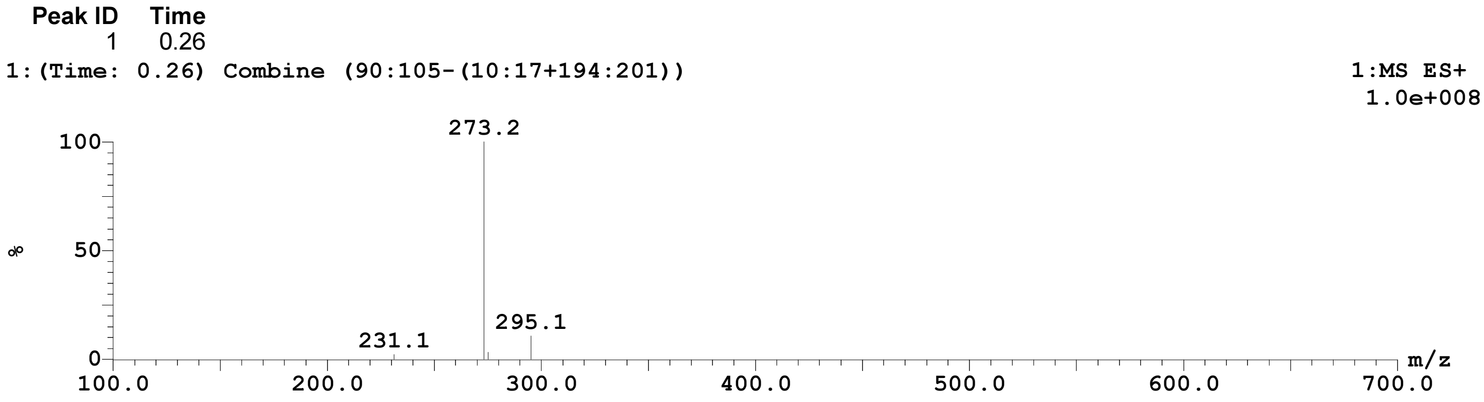

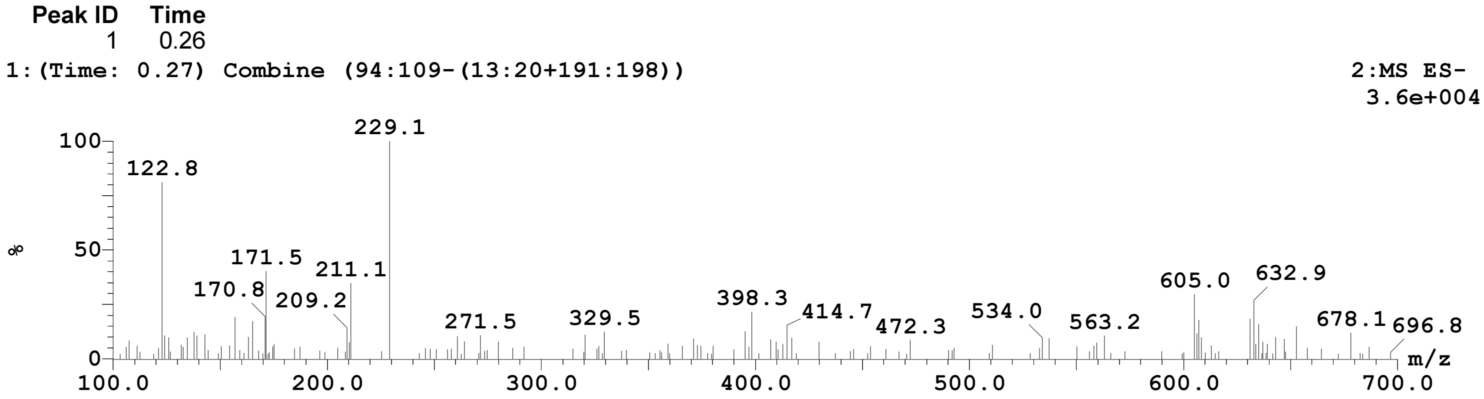

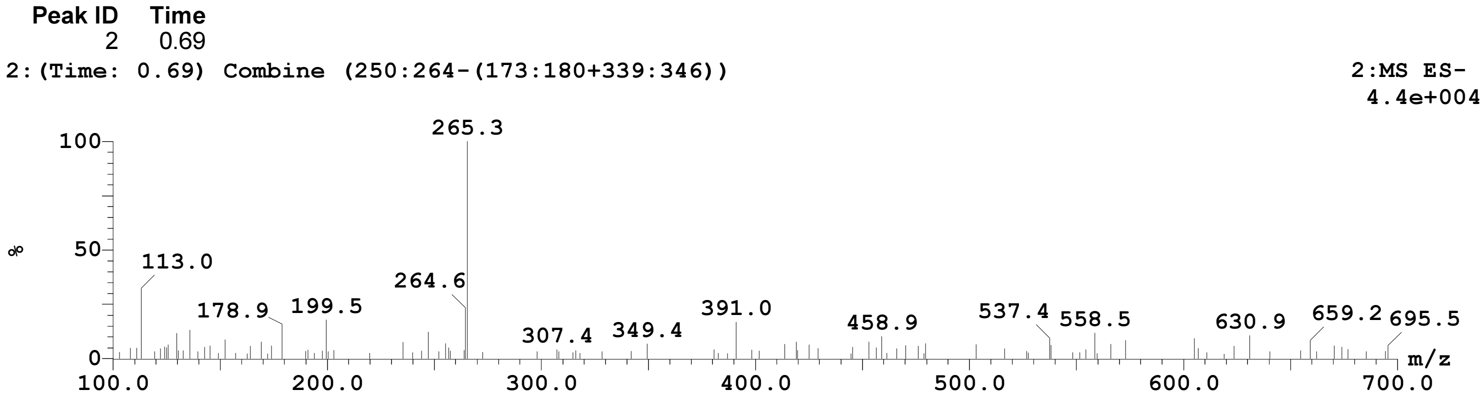

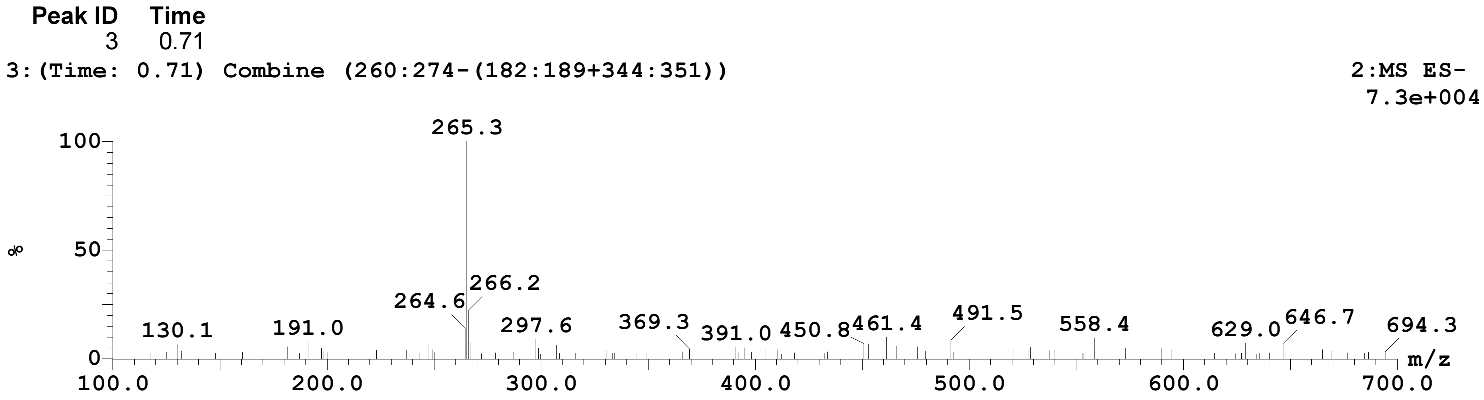

File:13zo241l3 Vial:5:19 ID:F5
Method:C:\MASSLYNX\1minLC_MS.olp

Peak ID **Time**
4 0.82

4:(Time: 0.82) Combine (298:313-(222:229+385:392)) 2:MS ES- 3.3e+004

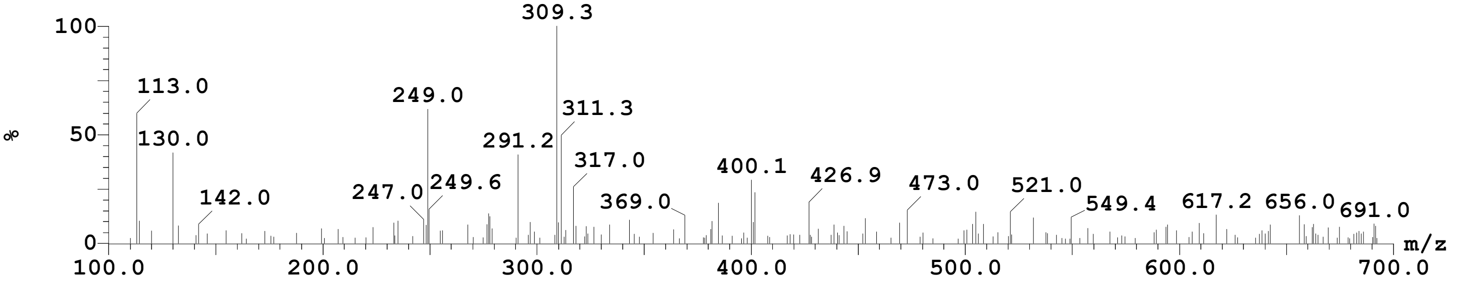

Peak ID **Time**
5 0.90

5:(Time: 0.90) Combine (329:344-250:257) 2:MS ES- 4.3e+004

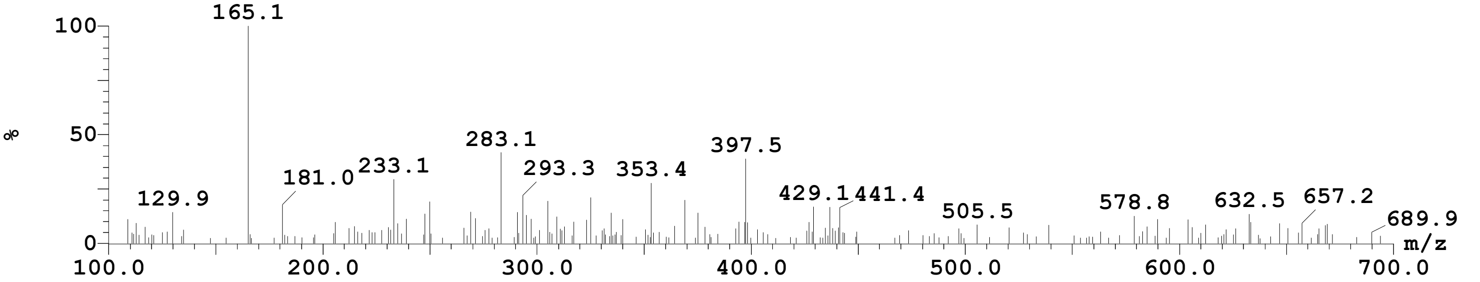

SYNGENTA SQD OPEN ACCESS LC-MS

File:13zo375l2 Vial:5:48 ID: **F6**

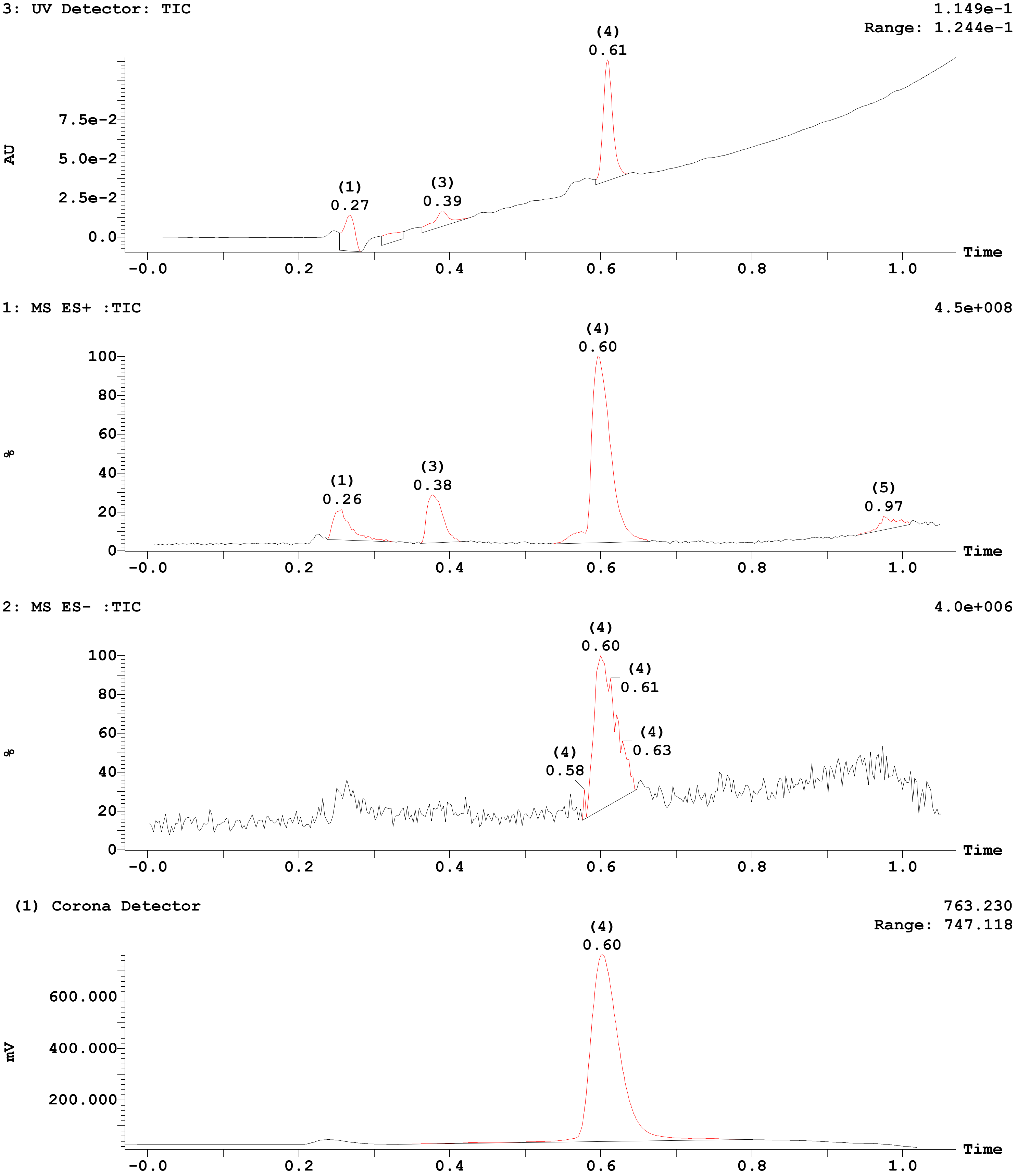

File:13zo375l2 Vial:5:48 ID:F6
Method:C:\MASSLYNX\1minLC_MS.olp

Peak ID **Time**
1 0.26

1:(Time: 0.27) Combine (93:108-(13:21+182:189)) 1:MS ES+ 9.2e+006

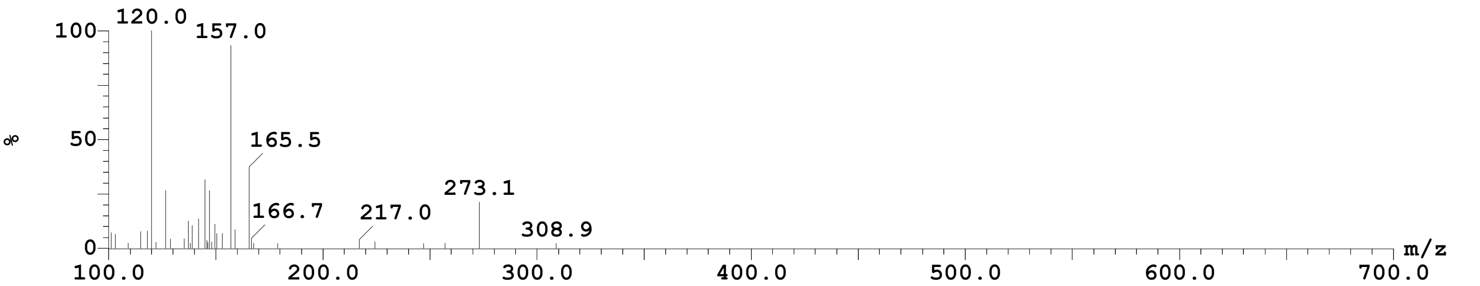

Peak ID **Time**
1 0.26

1:(Time: 0.27) Combine (93:108-(13:20+181:189)) 2:MS ES- 1.0e+004

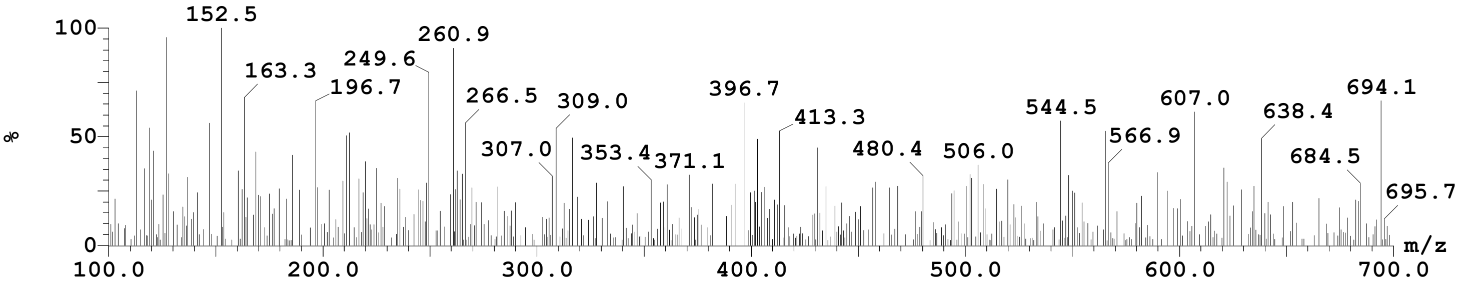

Peak ID **Time**
2 0.34

2:(Time: 0.34) Combine (120:135-(34:42+202:210)) 1:MS ES+ 1.3e+006

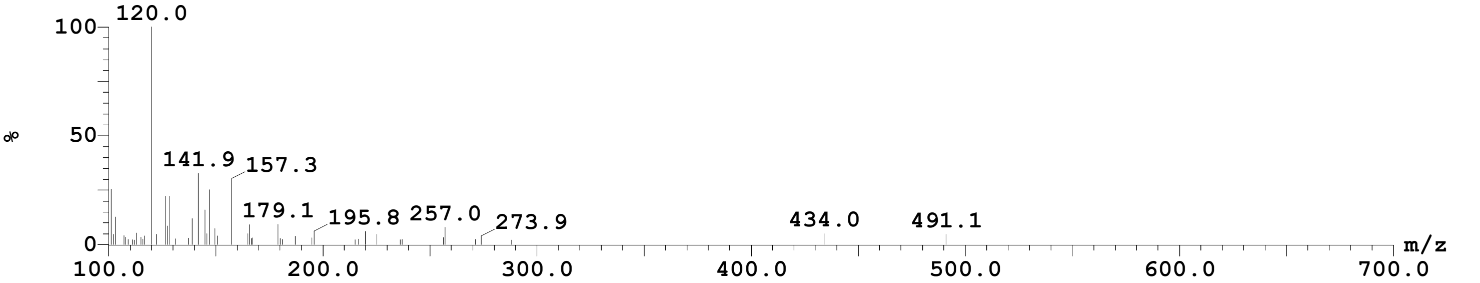

Peak ID **Time**
2 0.34

2:(Time: 0.34) Combine (119:134-(34:41+202:209)) 2:MS ES- 2.0e+004

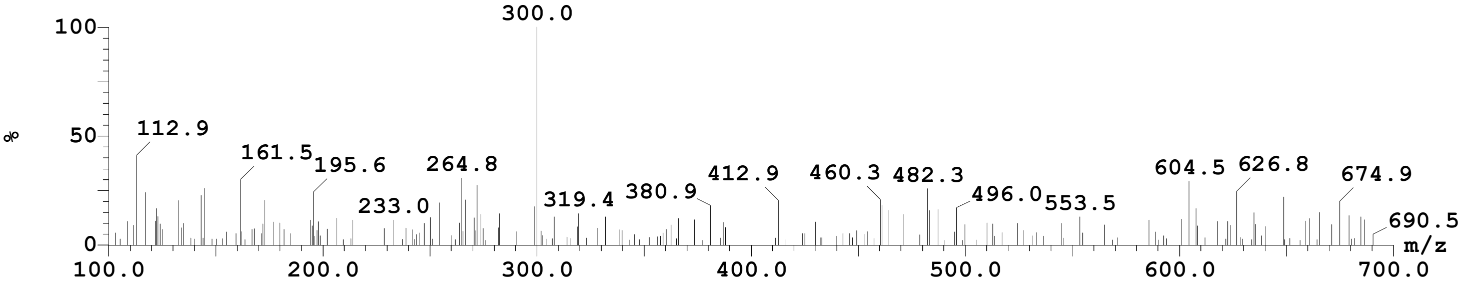

File:13zo375l2 Vial:5:48 ID:F6
Method:C:\MASSLYNX\1minLC_MS.olp

Peak ID Time
3 0.38

3:(Time: 0.38) Combine (135:150-(54:61+231:238)) 1:MS ES+ 6.2e+007

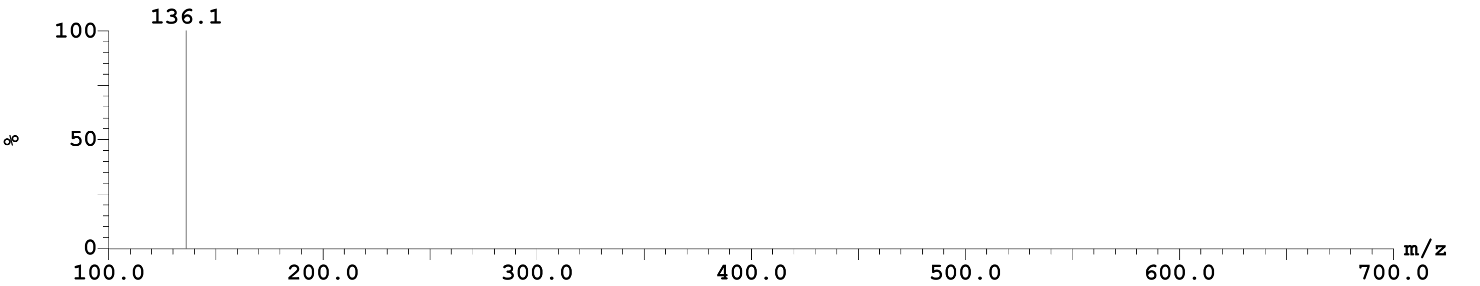

Peak ID Time
3 0.38

3:(Time: 0.39) Combine (139:154-(54:61+235:243)) 2:MS ES- 1.0e+004

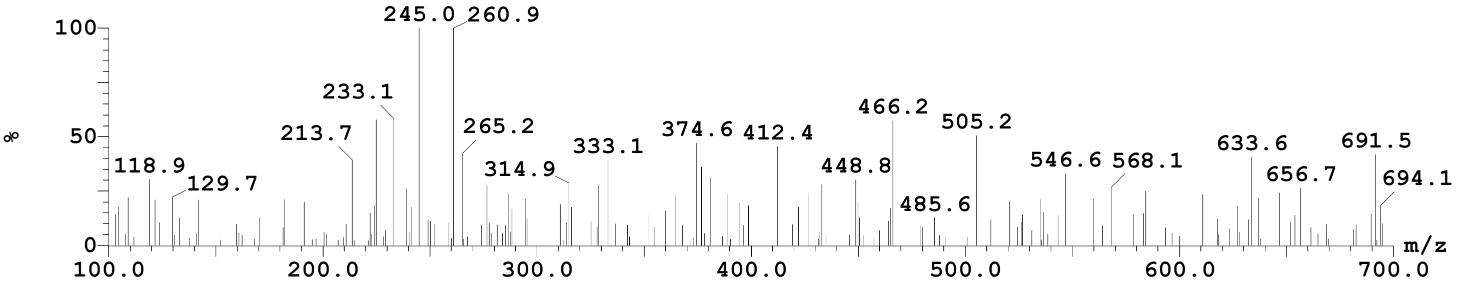

Peak ID Time
4 0.60

4:(Time: 0.60) Combine (216:231-(120:127+325:332)) 1:MS ES+ 8.0e+007

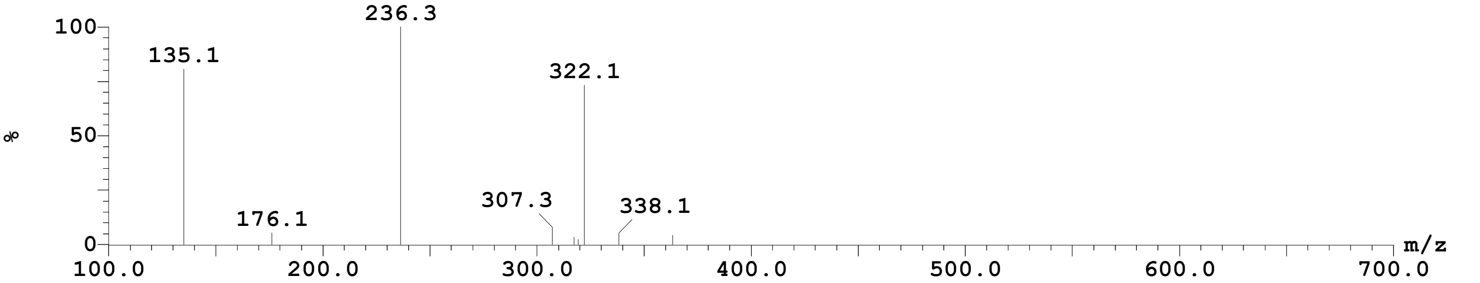

Peak ID Time
4 0.60

4:(Time: 0.60) Combine (218:233-(134:141+318:325)) 2:MS ES- 2.1e+005

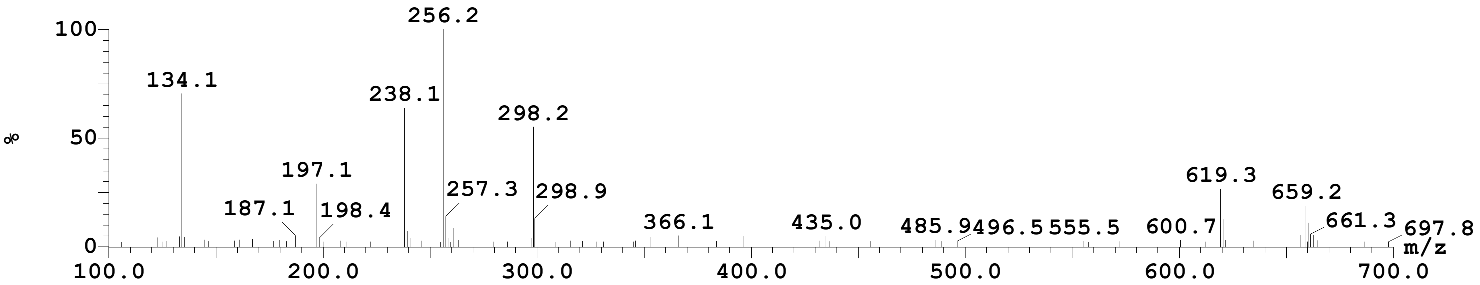

File:13zo375l2 Vial:5:48 ID:F6
Method:C:\MASSLYNX\1minLC_MS.olp

Peak ID	Time
5	0.97

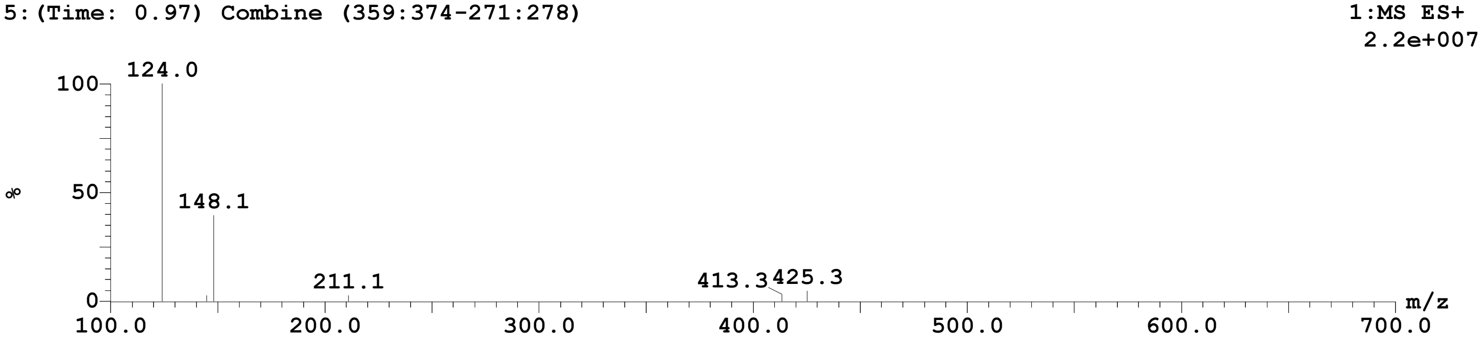

SYNGENTA SQD OPEN ACCESS LC-MS Page 1

File:13zp863l1 Vial:5:46 ID: **F7**

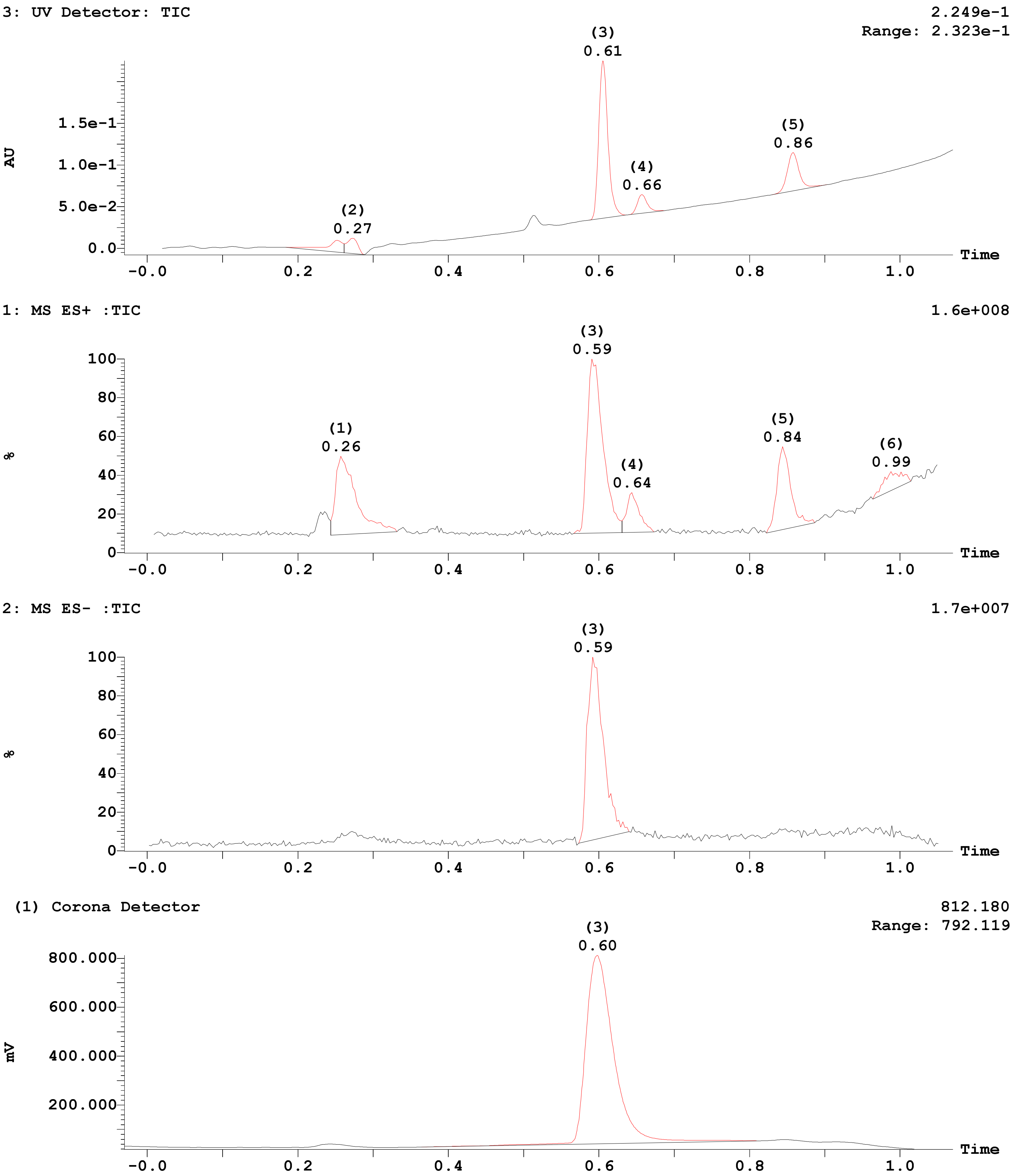

File:13zp863l1 Vial:5:46 ID:F7
Method:C:\MASSLYNX\1minLC_MS.olp

Peak ID	Time
1	0.26

1:(Time: 0.26) Combine (90:105-(10:17+200:207)) 1:MS ES+ 7.8e+006

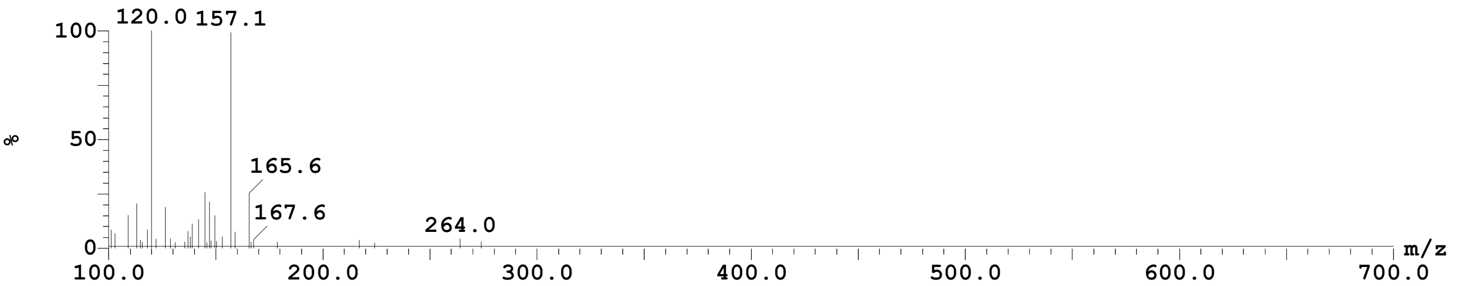

Peak ID	Time
2	0.27

2:(Time: 0.27) Combine (95:110-(16:24+183:191)) 1:MS ES+ 8.6e+006

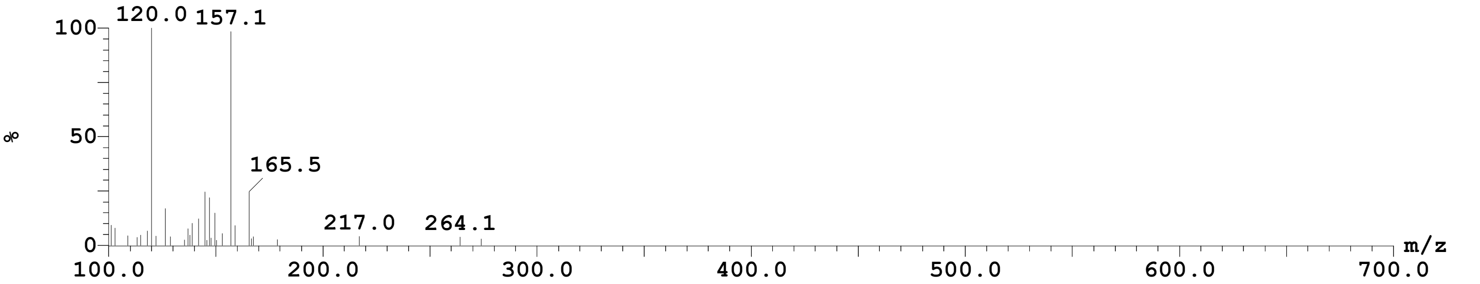

Peak ID	Time
2	0.27

2:(Time: 0.27) Combine (95:110-(16:23+183:190)) 2:MS ES- 1.9e+004

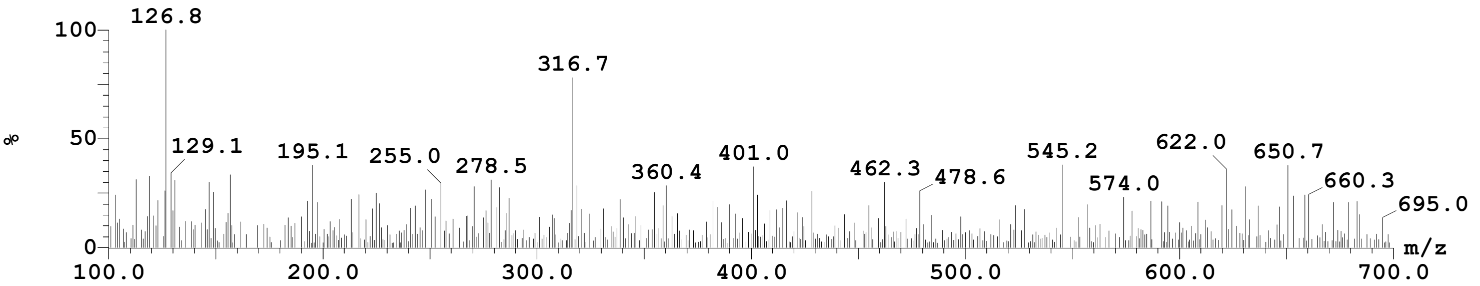

Peak ID	Time
3	0.59

3:(Time: 0.61) Combine (220:235-(138:146+314:322)) 1:MS ES+ 2.3e+007

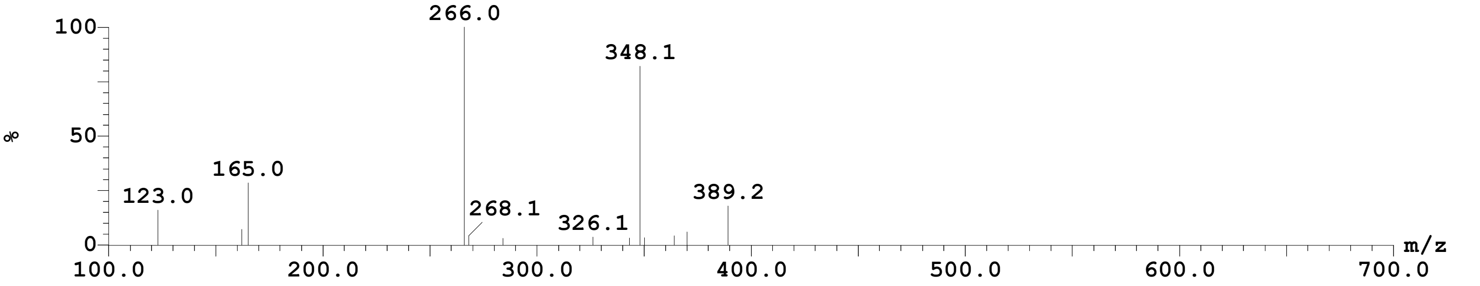

File:13zp863l1 Vial:5:46 ID:F7
Method:C:\MASSLYNX\1minLC_MS.olp

Peak ID **Time**
3 0.59

3:(Time: 0.59) Combine (214:229-(133:140+315:322)) 2:MS ES-
2.7e+006

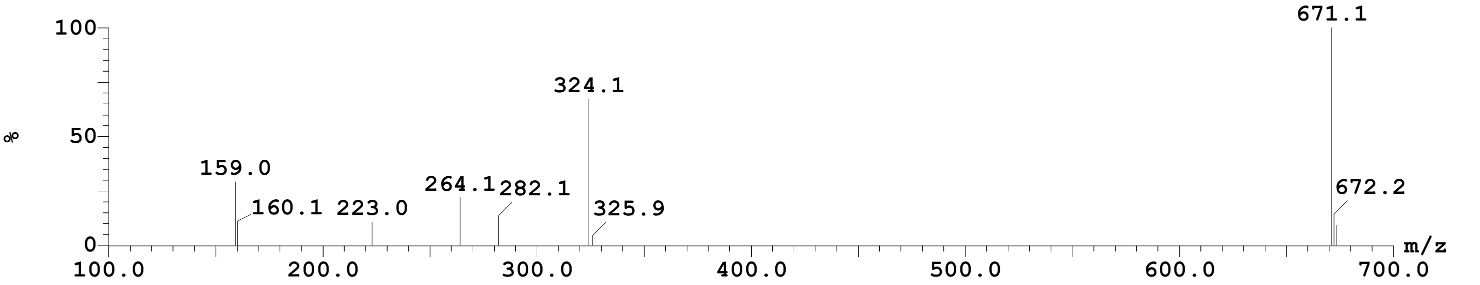

Peak ID **Time**
4 0.64

4:(Time: 0.64) Combine (235:249-(155:162+328:335)) 1:MS ES+
3.7e+006

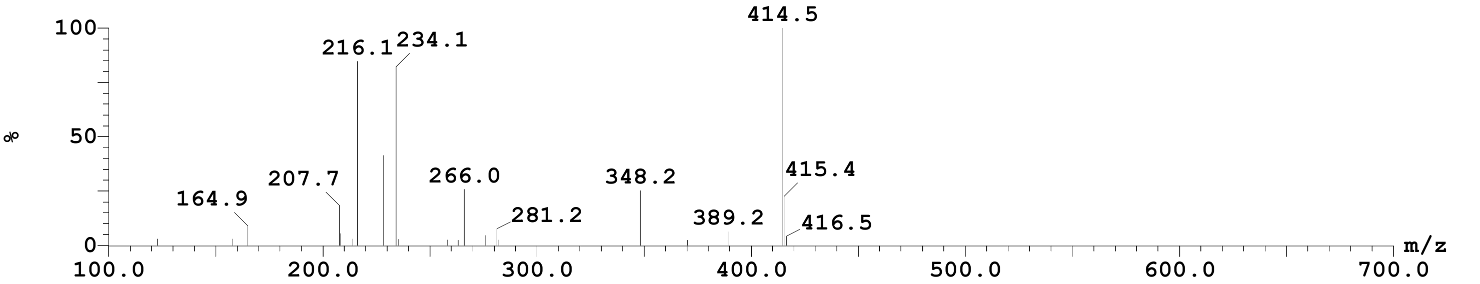

Peak ID **Time**
4 0.64

4:(Time: 0.66) Combine (239:254-(157:165+333:341)) 2:MS ES-
1.4e+005

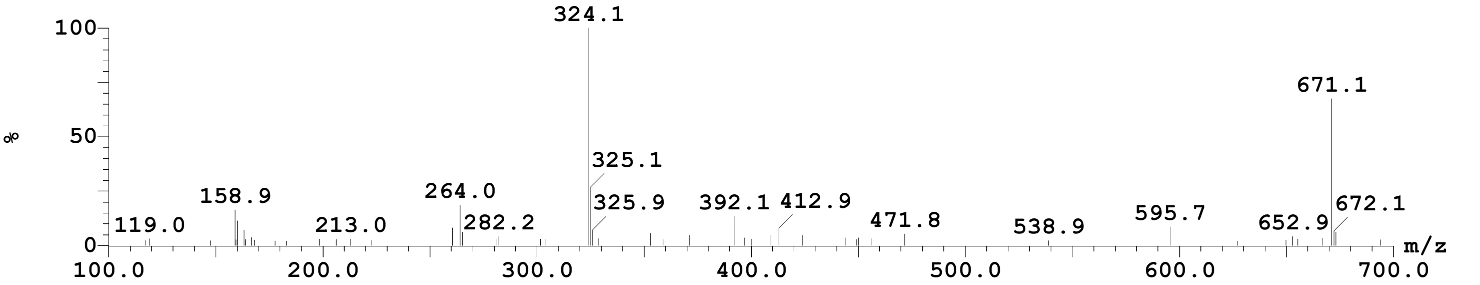

Peak ID **Time**
5 0.84

5:(Time: 0.86) Combine (315:330-229:237) 1:MS ES+
2.4e+007

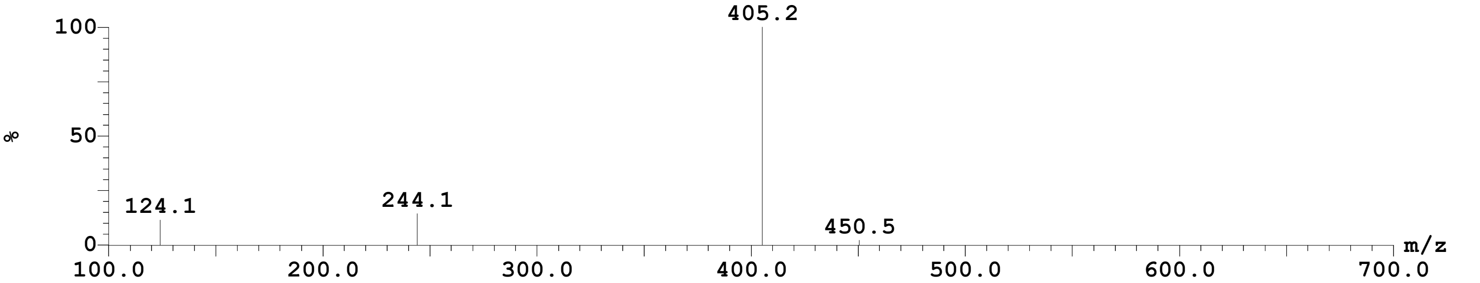

File:13zp863l1 Vial:5:46 ID:F7
Method:C:\MASSLYNX\1minLC_MS.olp

Peak ID **Time**
5 0.84

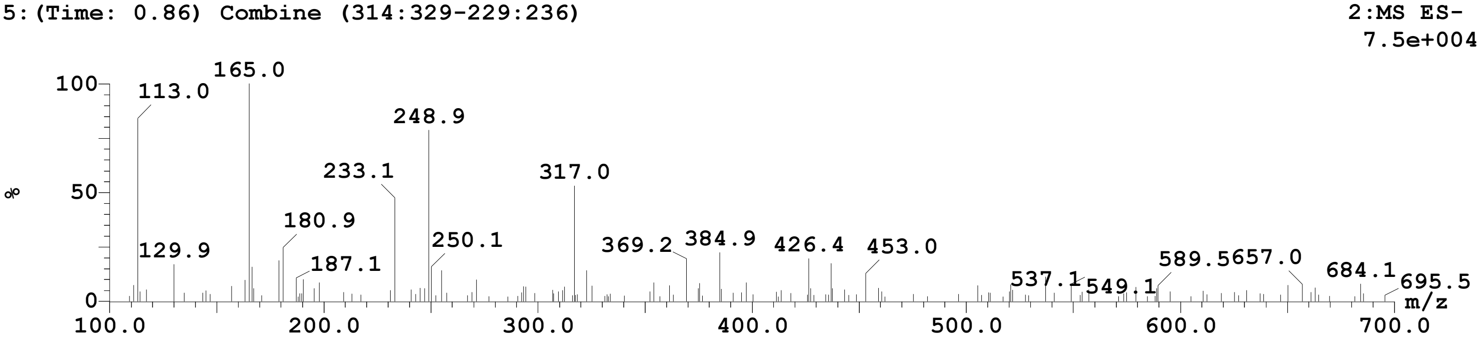

Peak ID **Time**
6 0.99

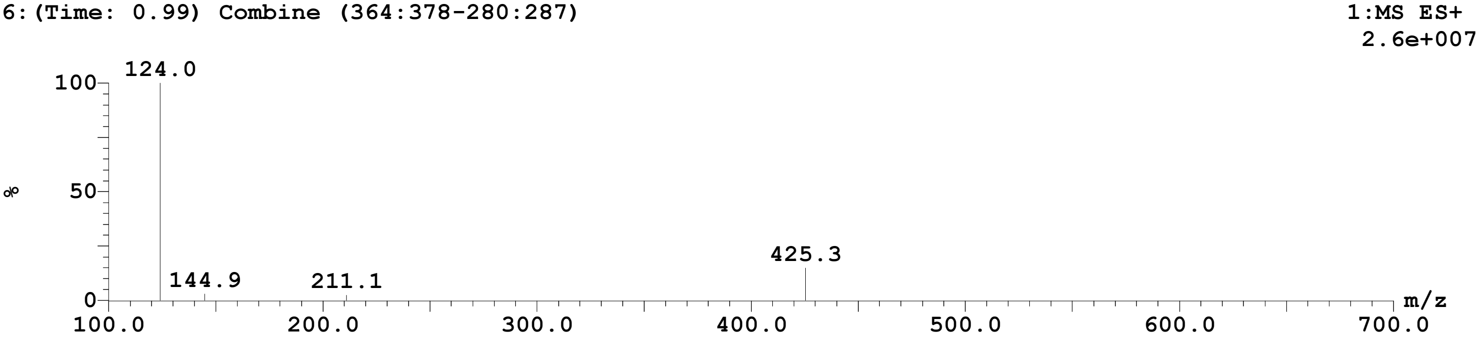

SYNGENTA SQD OPEN ACCESS LC-MS Page 1

File:13zp864l1 Vial:5:47 ID: **F8**

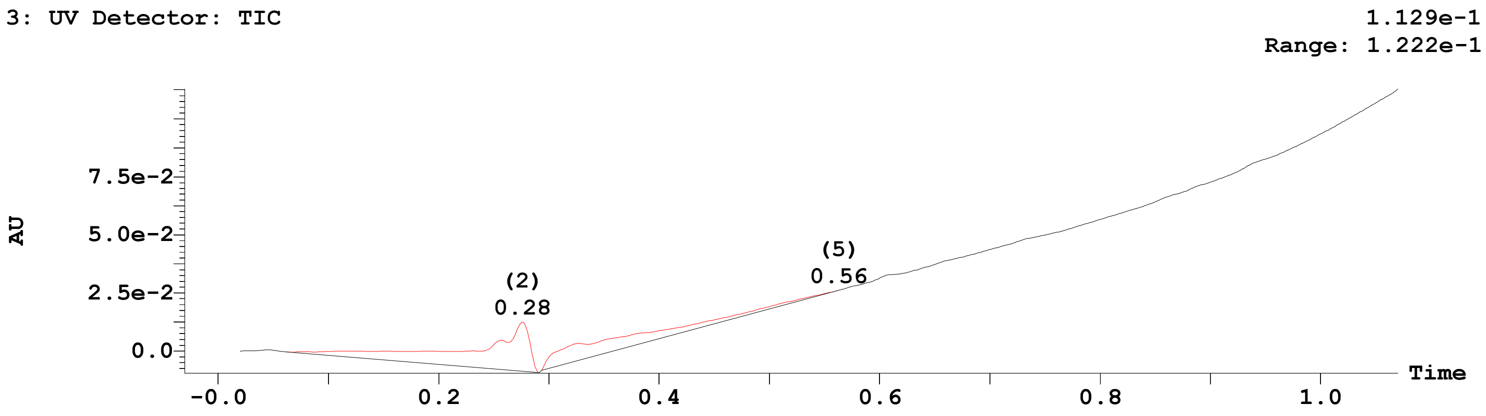

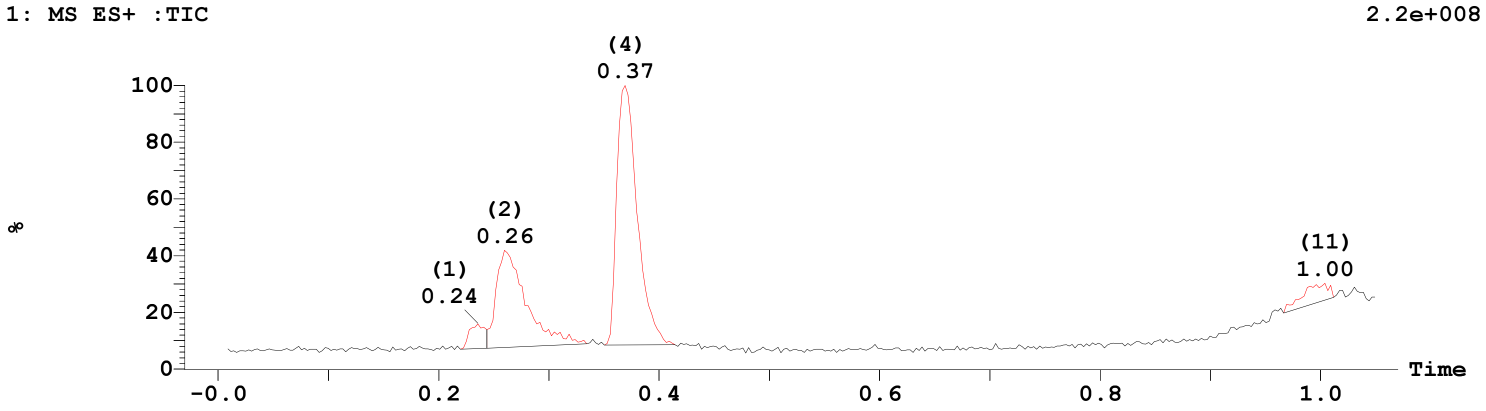

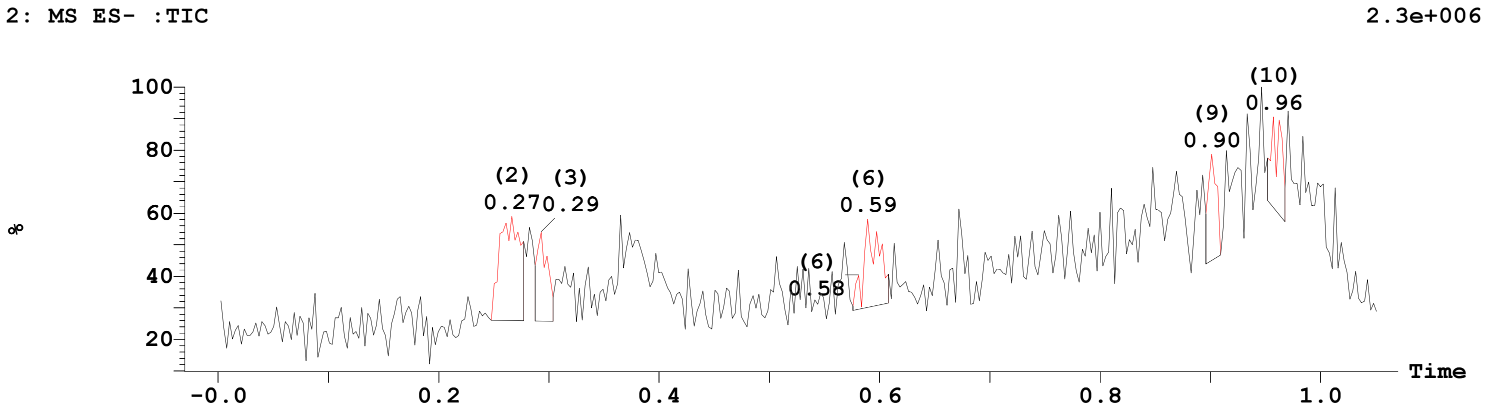

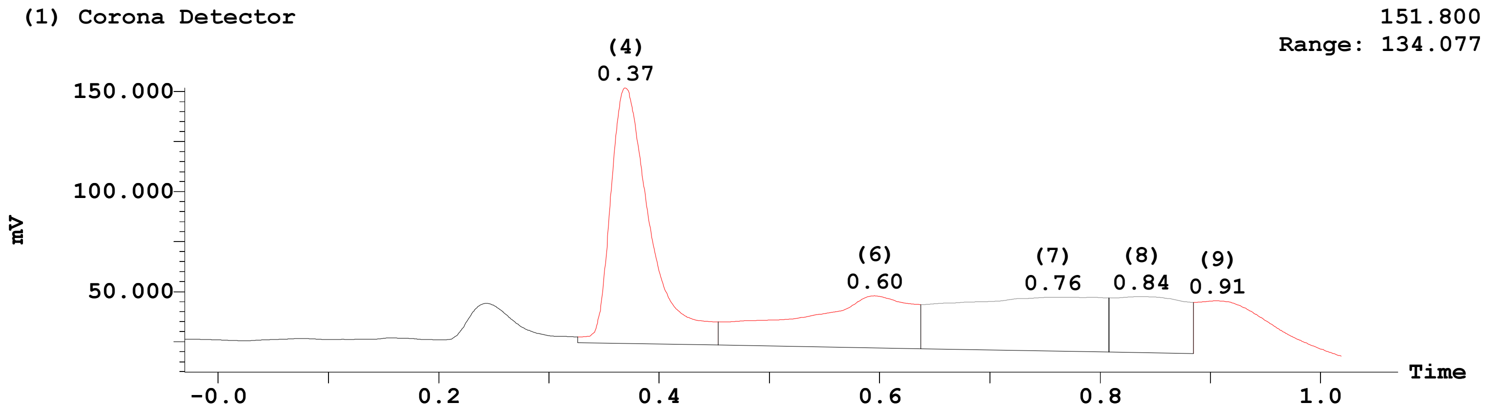

File:13zp864l1 Vial:5:47 ID:F8
Method:C:\MASSLYNX\1minLC_MS.olp

Peak ID	Time
1	0.24

1:(Time: 0.24) Combine (82:96-(1:8+167:174)) 1:MS ES+ 3.5e+006

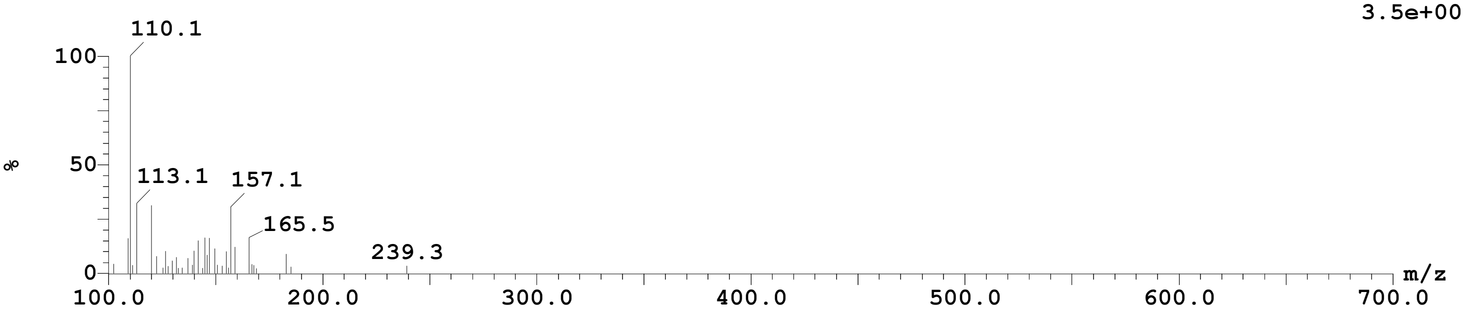

Peak ID	Time
2	0.26

2:(Time: 0.28) Combine (97:112-185:192) 1:MS ES+ 8.6e+006

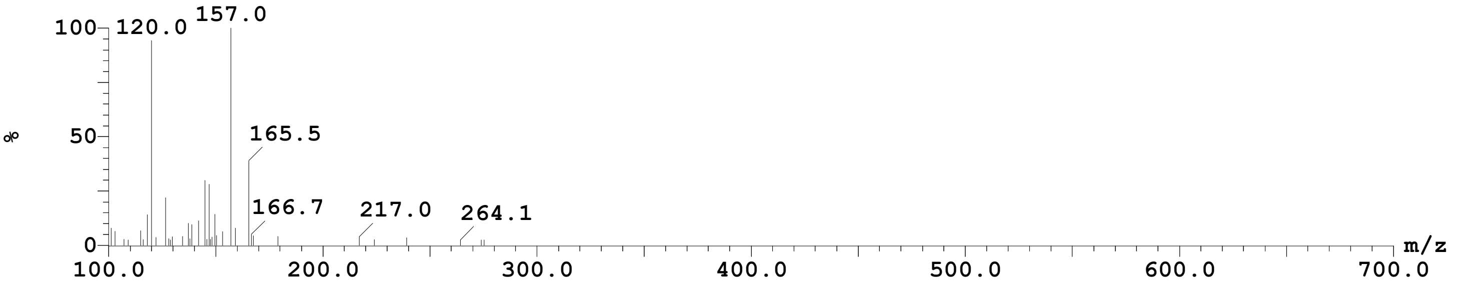

Peak ID	Time
2	0.26

2:(Time: 0.28) Combine (96:111-184:192) 2:MS ES- 1.1e+004

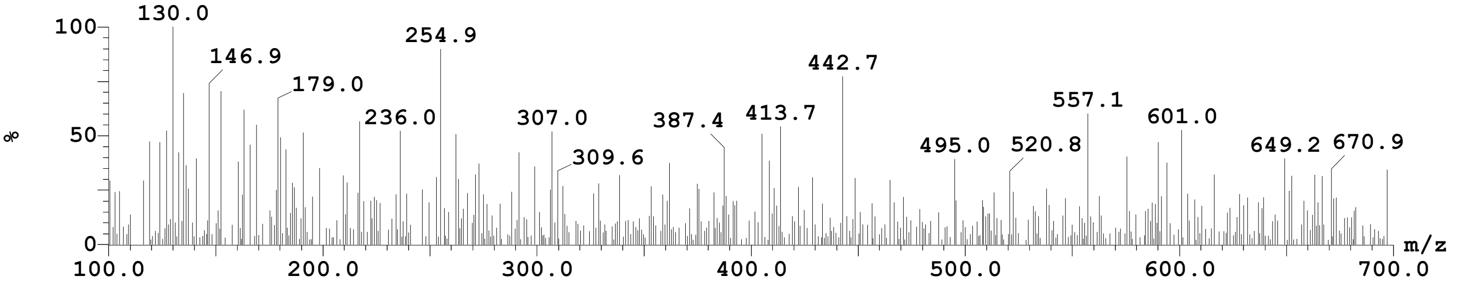

Peak ID	Time
3	0.29

3:(Time: 0.29) Combine (102:117-(26:33+189:196)) 2:MS ES- 1.3e+004

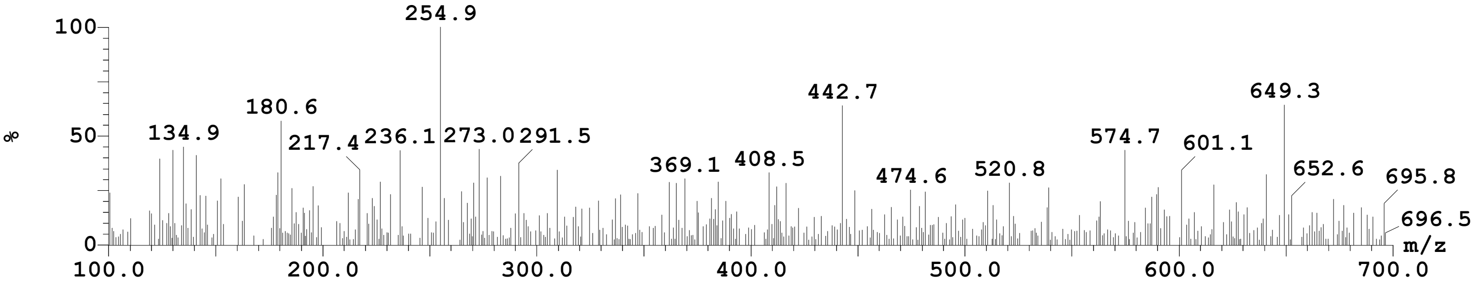

File:13zp864l1 Vial:5:47 ID:F8
Method:C:\MASSLYNX\1minLC_MS.olp

Peak ID **Time**
4 0.37

4:(Time: 0.37) Combine (131:146-(50:57+231:238)) 1:MS ES+ 4.4e+007

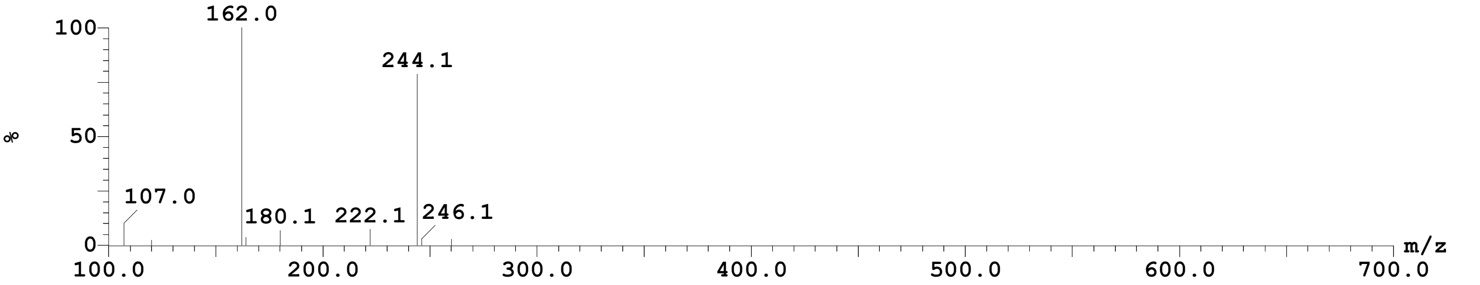

Peak ID **Time**
5 0.56

5:(Time: 0.56) Combine (204:219-(28:36+287:294)) 1:MS ES+ 1.5e+005

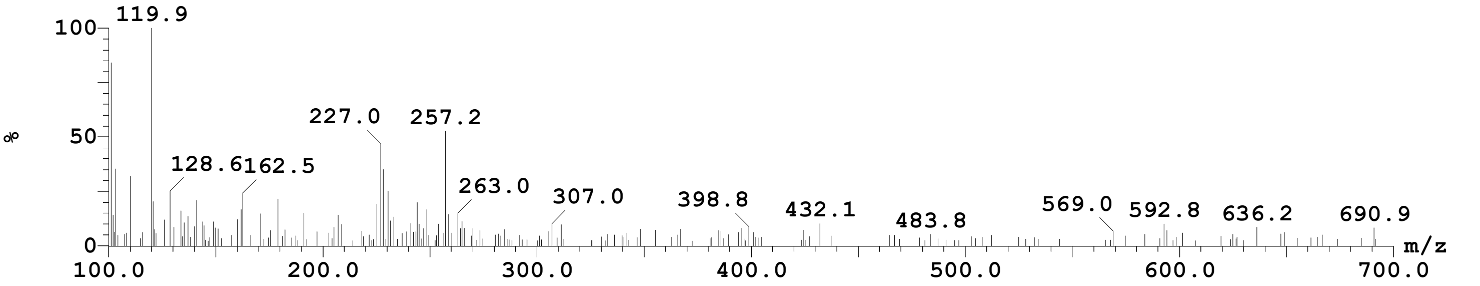

Peak ID **Time**
6 0.59

6:(Time: 0.59) Combine (213:228-(134:141+303:310)) 2:MS ES- 6.3e+004

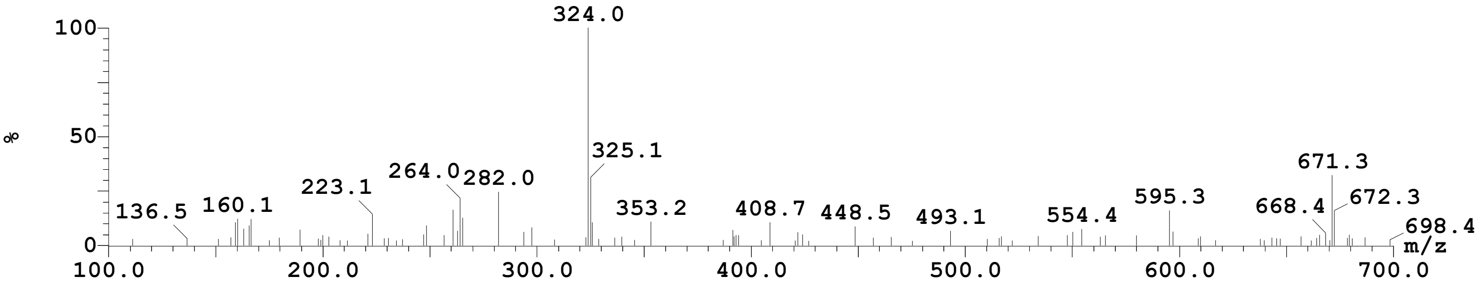

Peak ID **Time**
9 0.90

9:(Time: 0.90) Combine (331:345-254:261) 2:MS ES- 6.5e+004

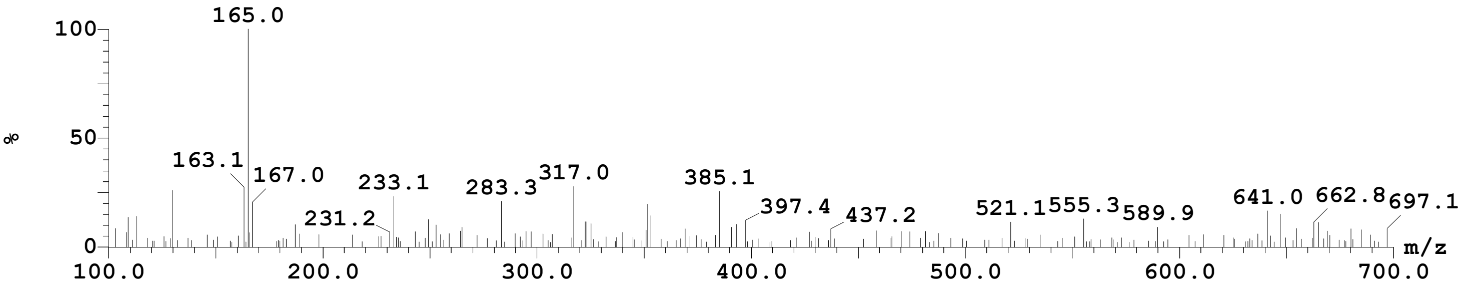

Peak ID **Time**
10 0.96

10:(Time: 0.96) Combine (352:366-275:282) 2:MS ES- 1.8e+005

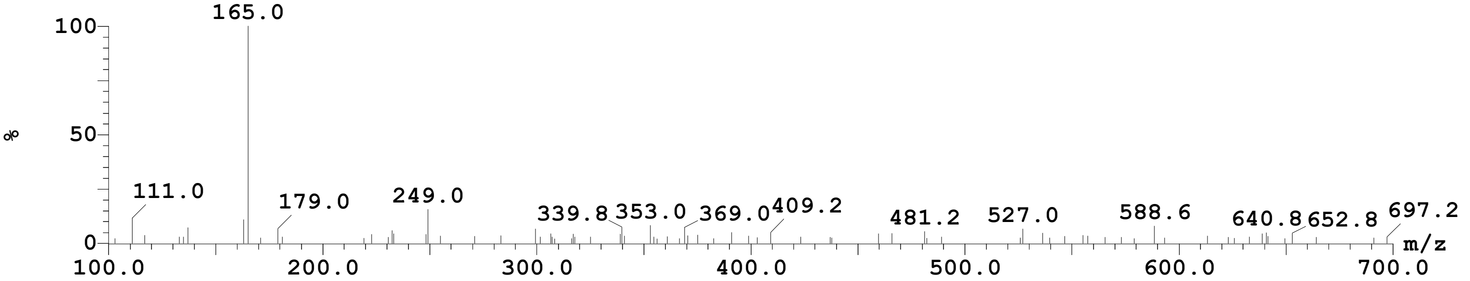

Peak ID **Time**
11 1.00

11:(Time: 1.00) Combine (370:384-281:288) 1:MS ES+ 3.0e+007

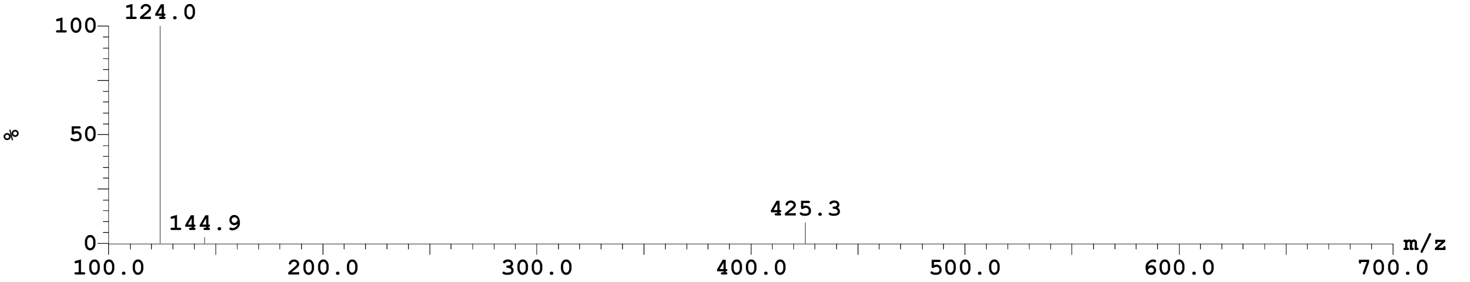

File:13zo393l2 Vial:5:48 ID: **F9**

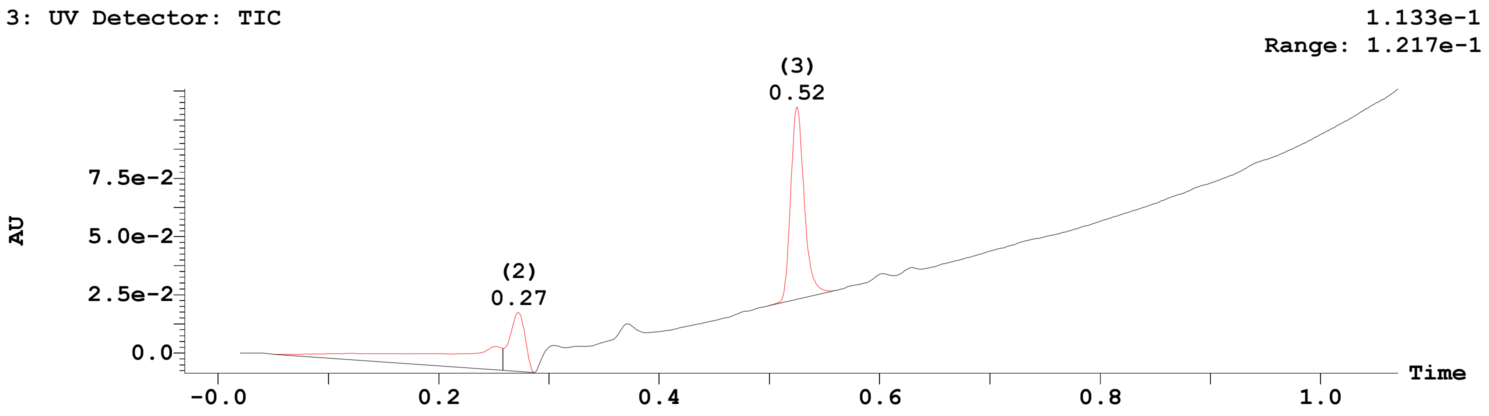

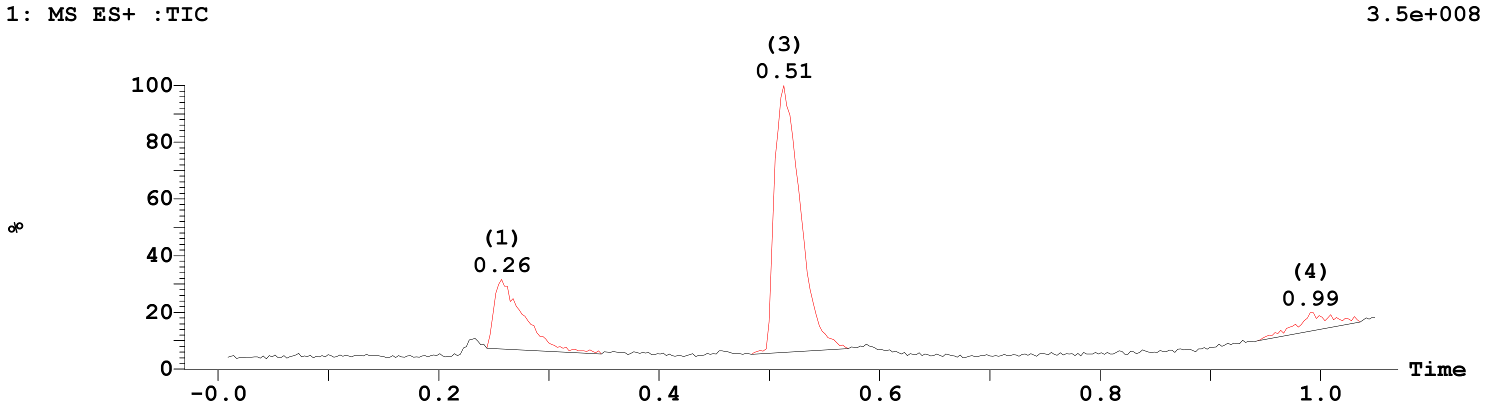

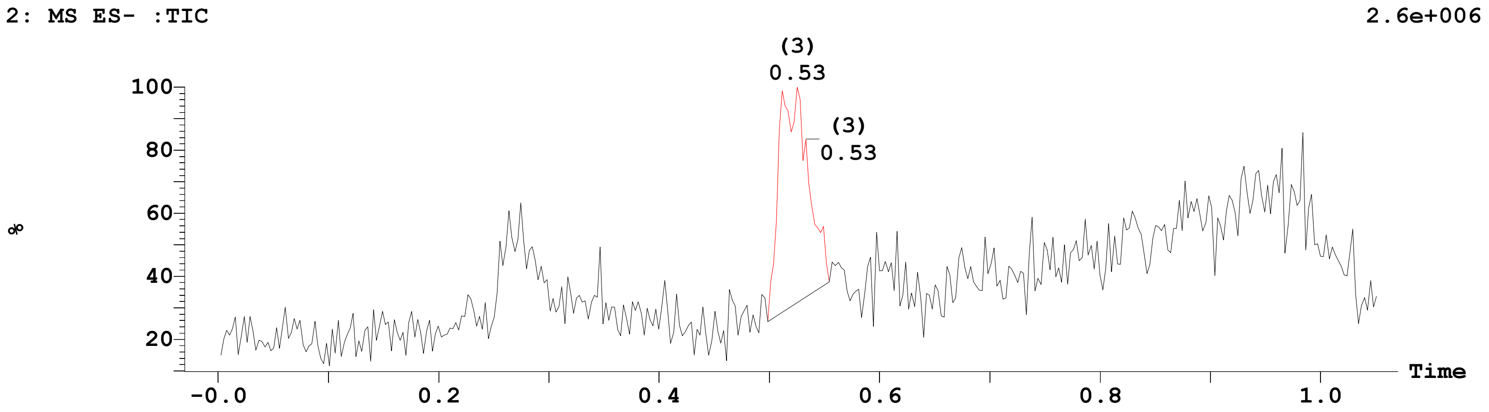

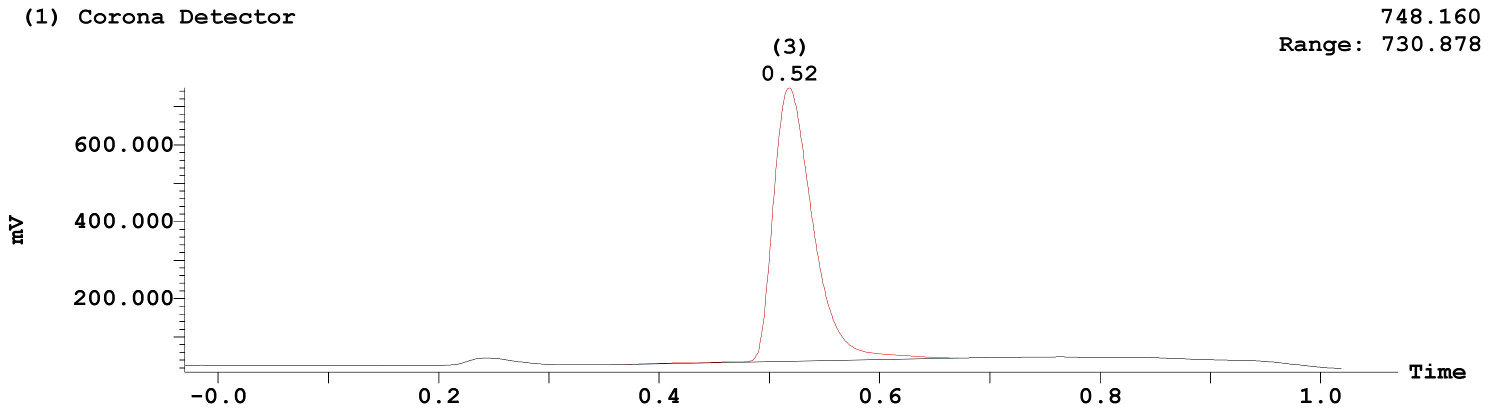

File:13zo393l2 Vial:5:48 ID:F9
Method:C:\MASSLYNX\1minLC_MS.olp

Peak ID **Time**
1 0.26
1: (Time: 0.25) Combine (87:102-172:180) 1:MS ES+ 6.3e+006

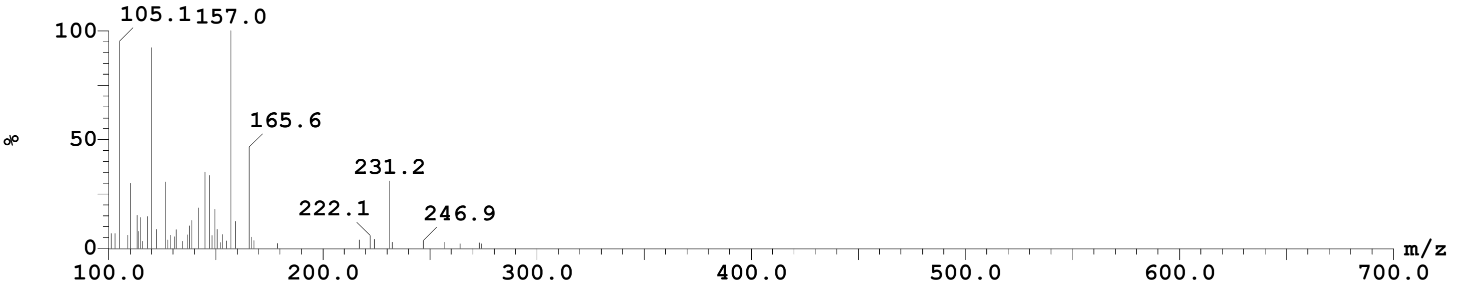

Peak ID **Time**
1 0.26
1: (Time: 0.25) Combine (87:102-172:179) 2:MS ES- 2.6e+004

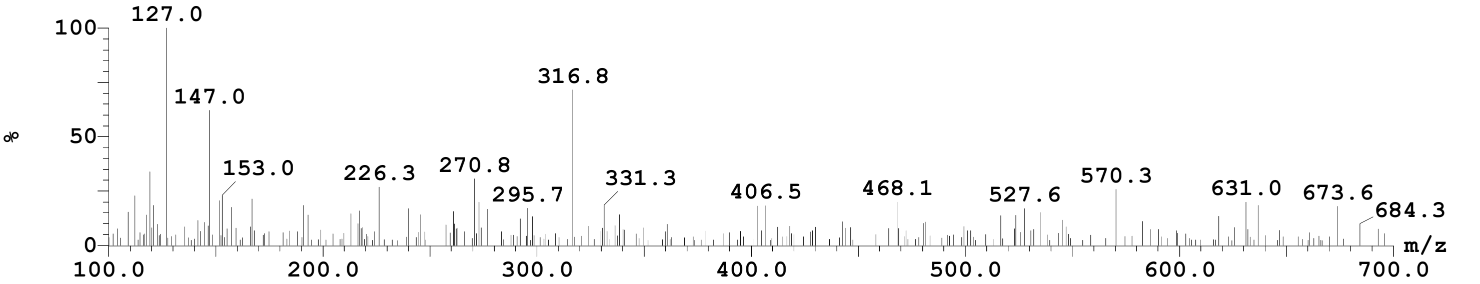

Peak ID **Time**
2 0.27
2: (Time: 0.27) Combine (95:110-(15:22+183:191)) 1:MS ES+ 8.6e+006

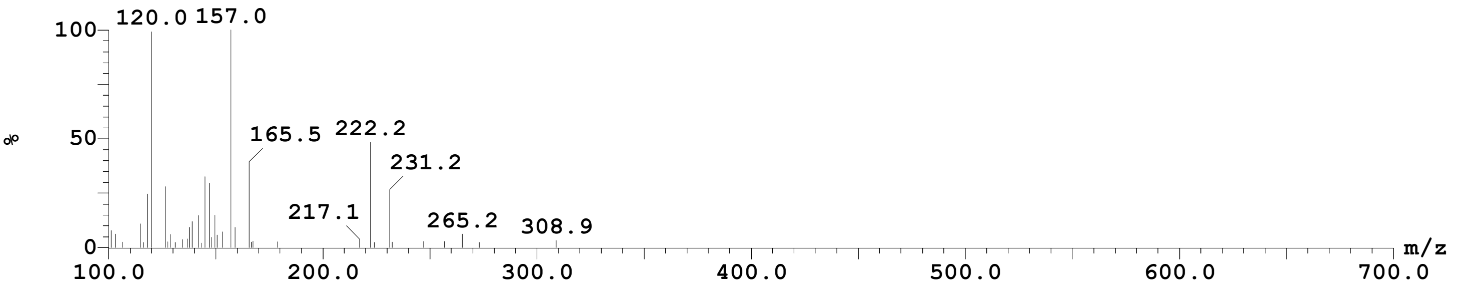

Peak ID **Time**
2 0.27
2: (Time: 0.27) Combine (95:110-(14:22+183:190)) 2:MS ES- 4.0e+004

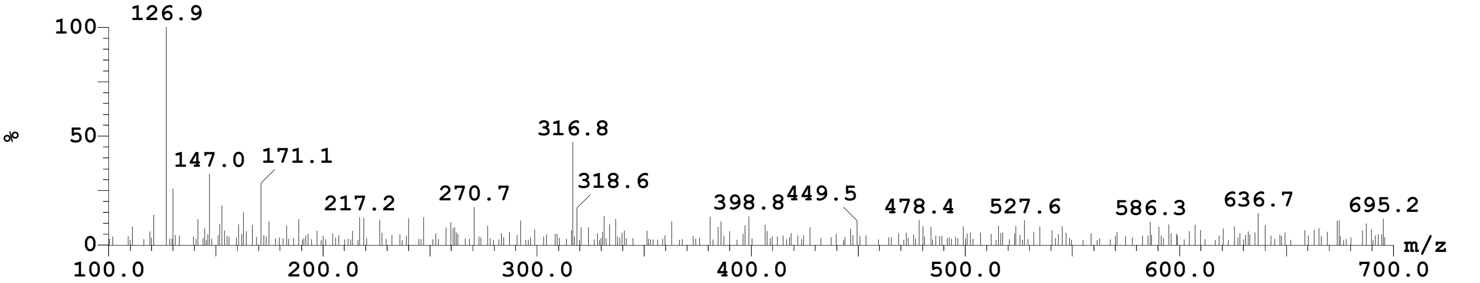

File:13zo393l2 Vial:5:48 ID:F9
Method:C:\MASSLYNX\1minLC_MS.olp

Peak ID **Time**
3 0.51

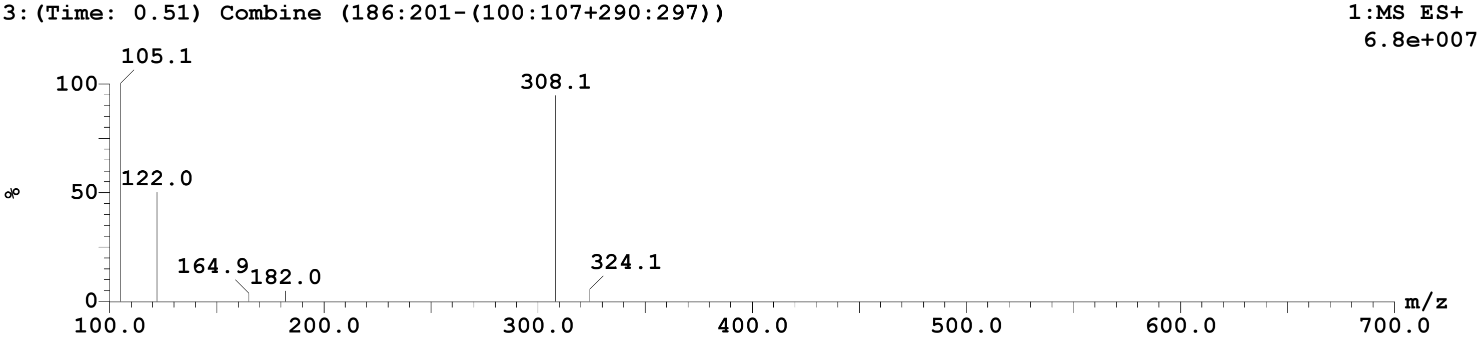

Peak ID **Time**
3 0.51

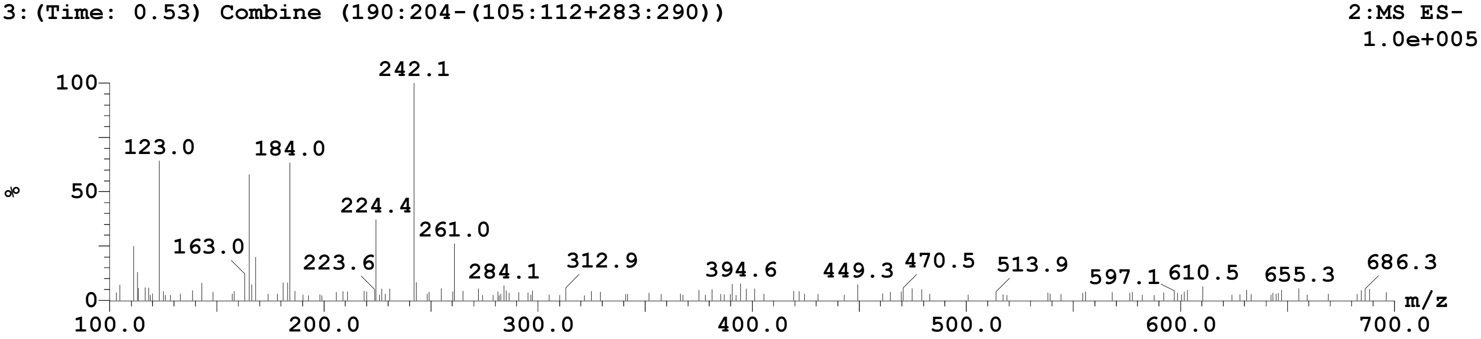

Peak ID **Time**
4 0.99

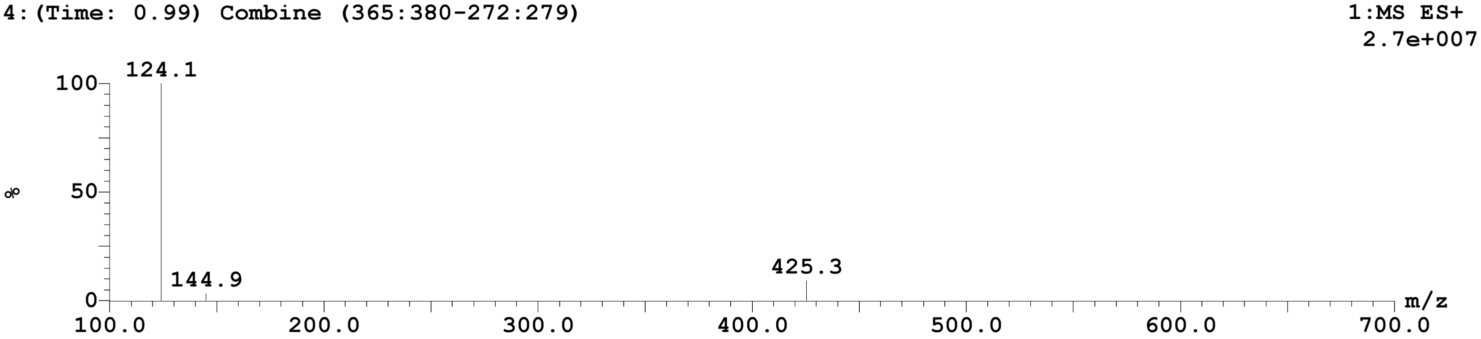

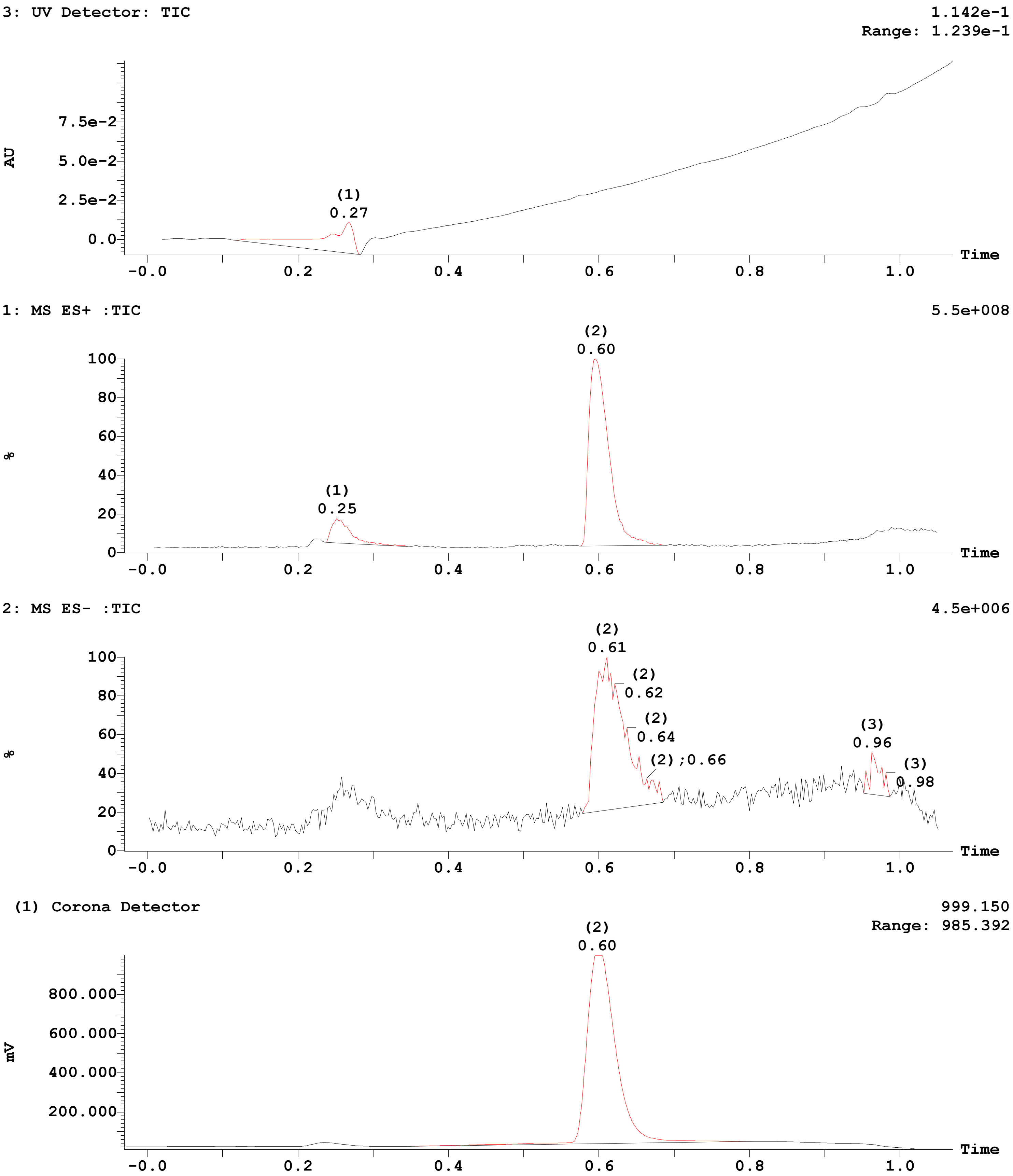
3: UV Detector: TIC
1.142e-1
Range: 1.239e-1
AU
7.5e-2
5.0e-2
2.5e-2
0.0
(1)
0.27
Time
-0.0
0.2
0.4
0.6
0.8
1.0
1: MS ES+ :TIC
5.5e+008
%
100
80
60
40
20
0
(1)
0.25
(2)
0.60
2: MS ES- :TIC
4.5e+006
(2)
0.61
(2)
0.62
(2)
0.64
(2);0.66
(3)
0.96
(3)
0.98
(1) Corona Detector
999.150
Range: 985.392
mV
800.000
600.000
400.000
200.000
(2)
0.60

File:13zp866l1 Vial:5:46 ID:F10
Method:C:\MASSLYNX\1minLC_MS.olp

Peak ID **Time**
1 0.25

1:(Time: 0.27) Combine (93:108-182:189) 1:MS ES+ 9.2e+006

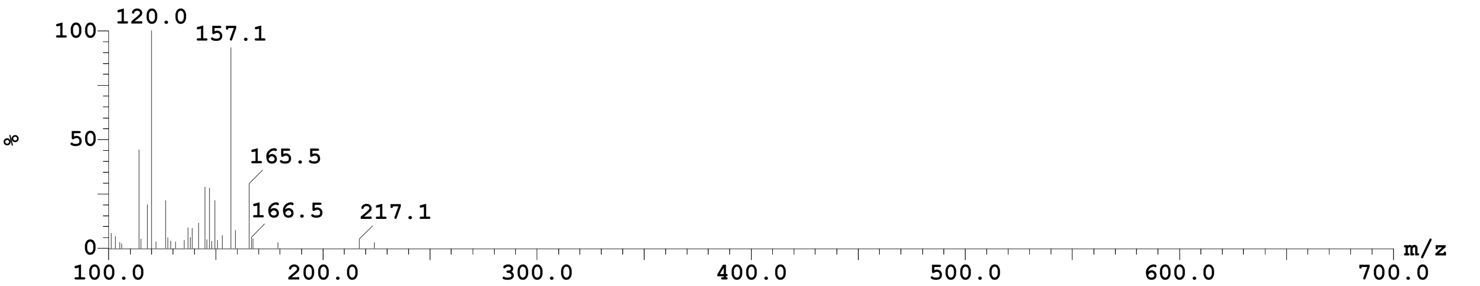

Peak ID **Time**
1 0.25

1:(Time: 0.27) Combine (93:108-181:189) 2:MS ES- 1.9e+004

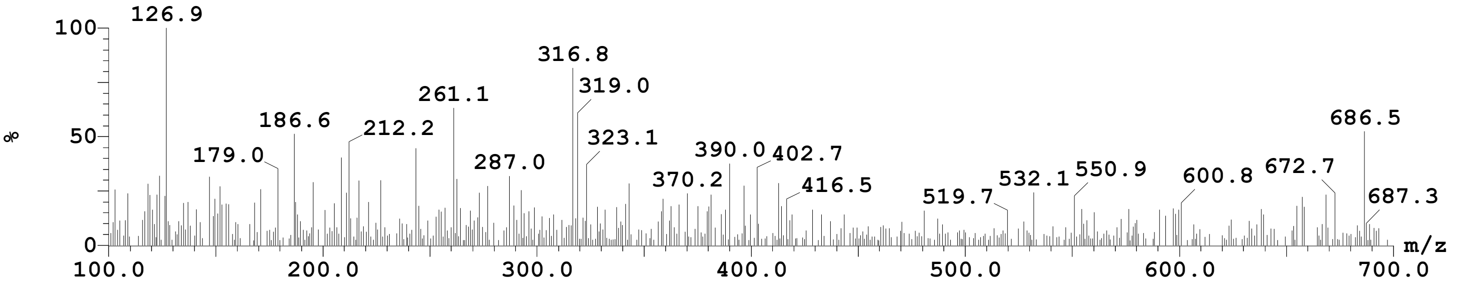

Peak ID **Time**
2 0.60

2:(Time: 0.60) Combine (217:232-(134:141+333:340)) 1:MS ES+ 9.8e+007

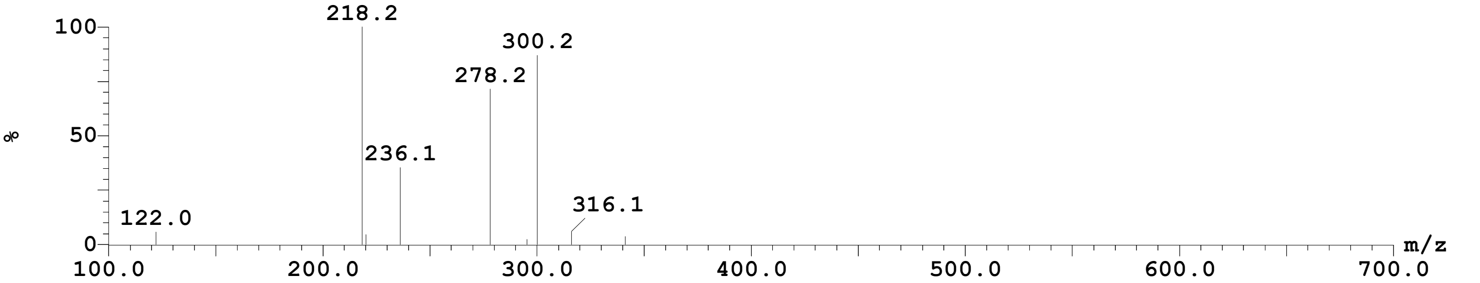

Peak ID **Time**
2 0.60

2:(Time: 0.61) Combine (222:236-(135:142+332:339)) 2:MS ES- 9.2e+004

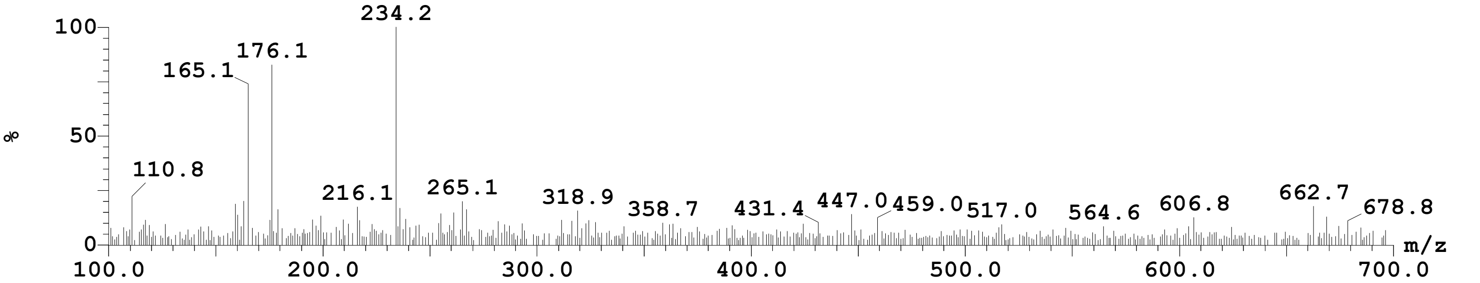

File:13zp866l1 Vial:5:46 ID:F10
Method:C:\MASSLYNX\1minLC_MS.olp

Peak ID	Time
3	0.96

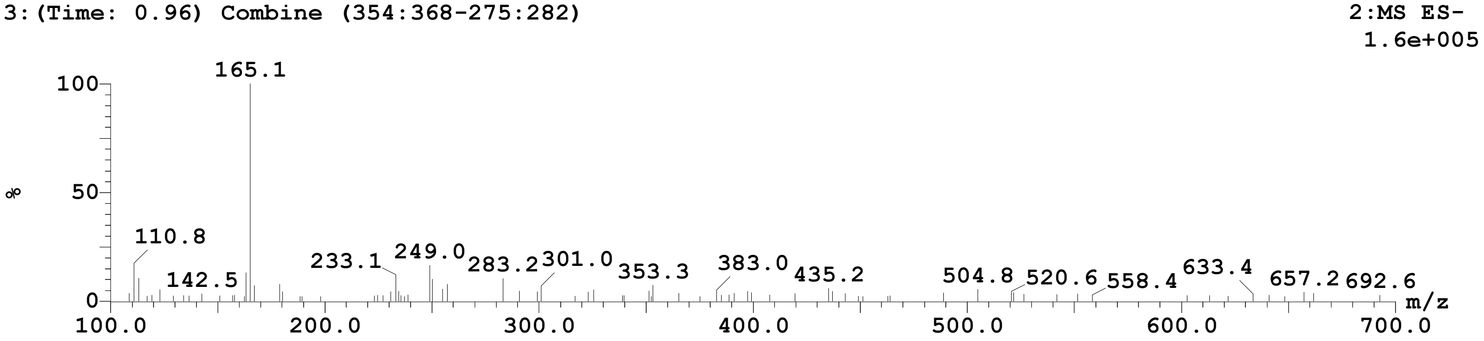

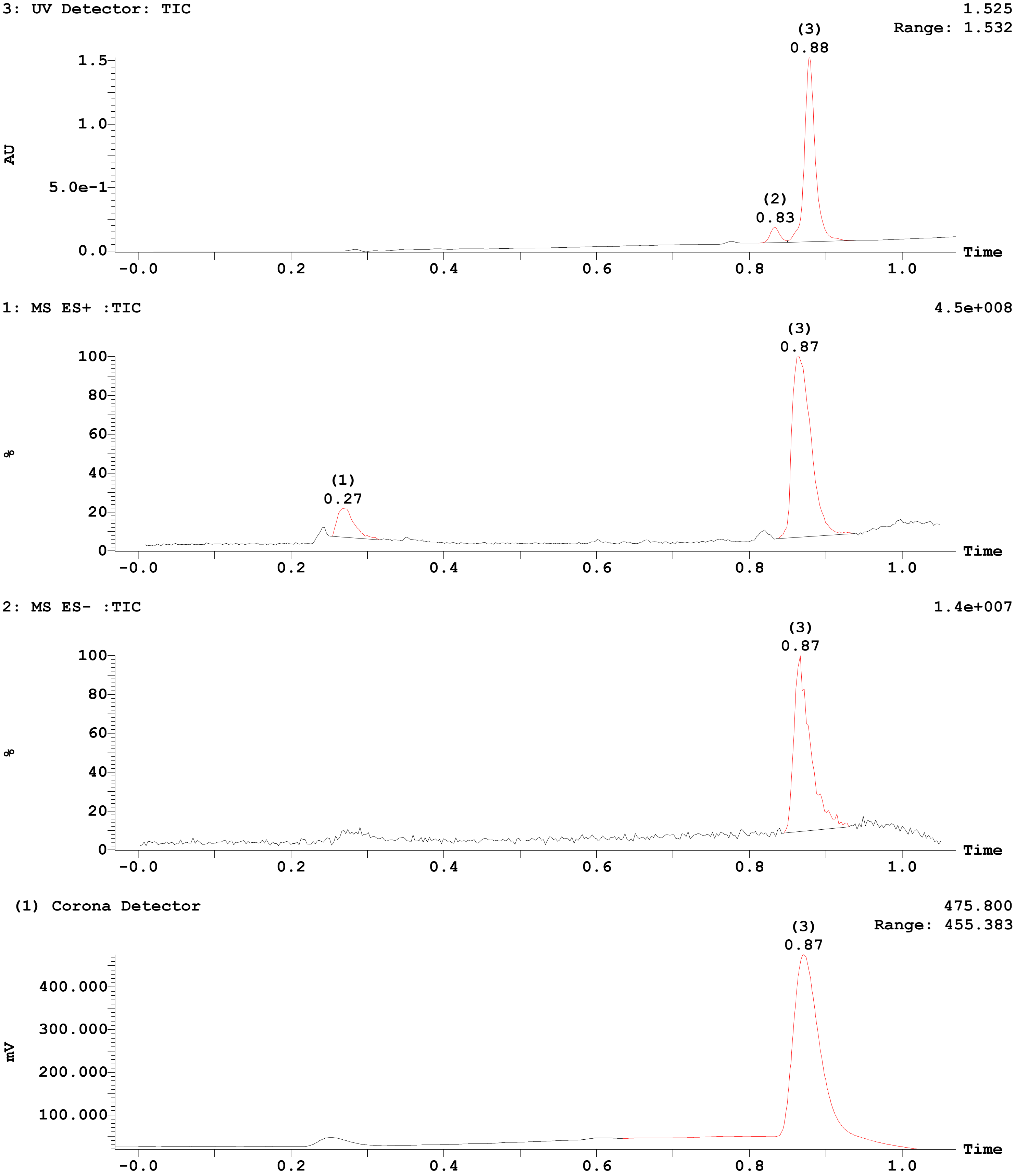
3: UV Detector: TIC
1.525
Range: 1.532
(3) 0.88
(2) 0.83
AU
Time
1: MS ES+ :TIC
4.5e+008
(3) 0.87
(1) 0.27
%
Time
2: MS ES- :TIC
1.4e+007
(3) 0.87
%
Time
(1) Corona Detector
475.800
Range: 455.383
(3) 0.87
mV
Time

File:13zp867l1 Vial:5:47 ID:G1
Method:C:\MASSLYNX\1minLC_MS.olp

Peak ID **Time**
1 0.27

1:(Time: 0.27) Combine (94:108-(13:20+194:201)) 1:MS ES+ 8.0e+006

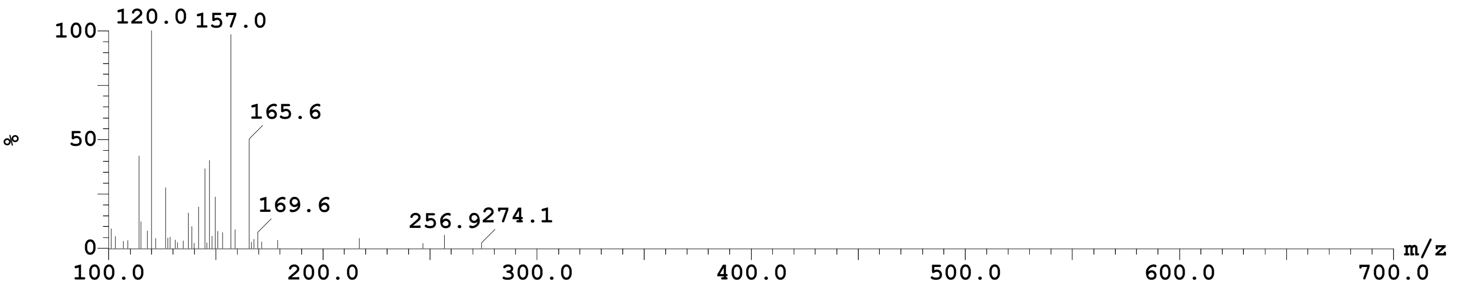

Peak ID **Time**
2 0.83

2:(Time: 0.83) Combine (306:321-(222:230+394:402)) 1:MS ES+ 8.3e+006

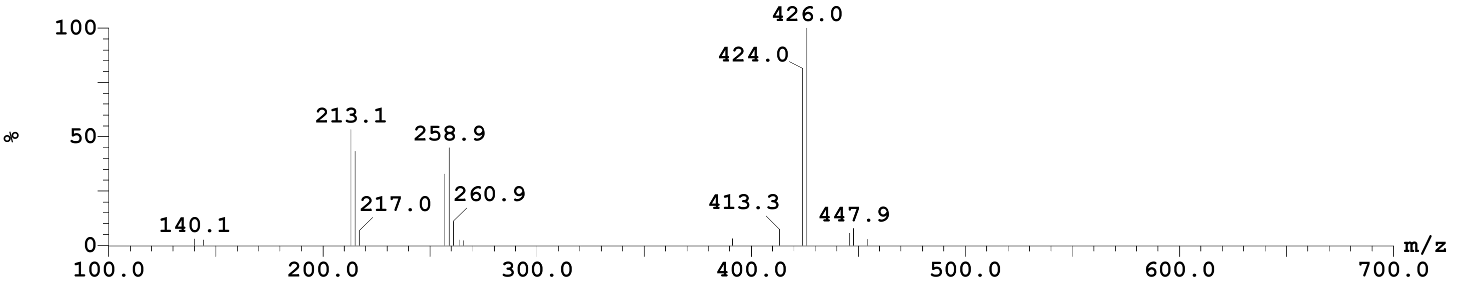

Peak ID **Time**
2 0.83

2:(Time: 0.83) Combine (305:320-(222:229+394:401)) 2:MS ES- 8.6e+004

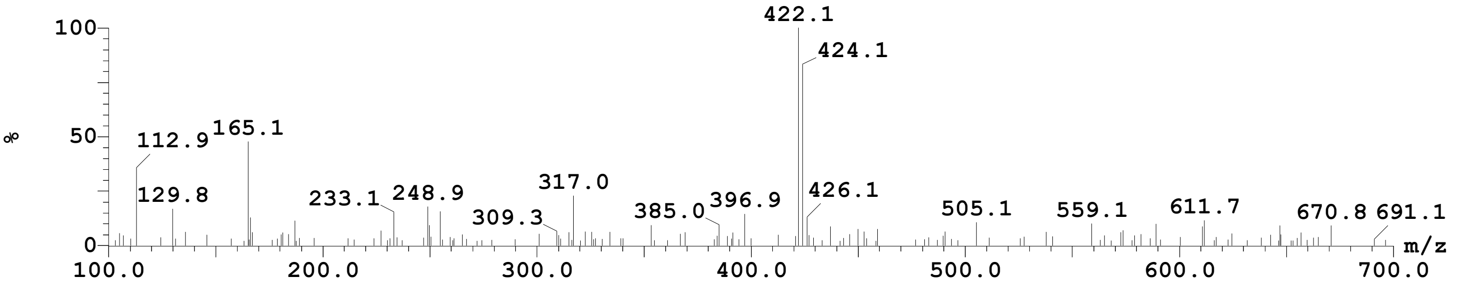

Peak ID **Time**
3 0.87

3:(Time: 0.88) Combine (322:337-237:244) 1:MS ES+ 1.2e+008

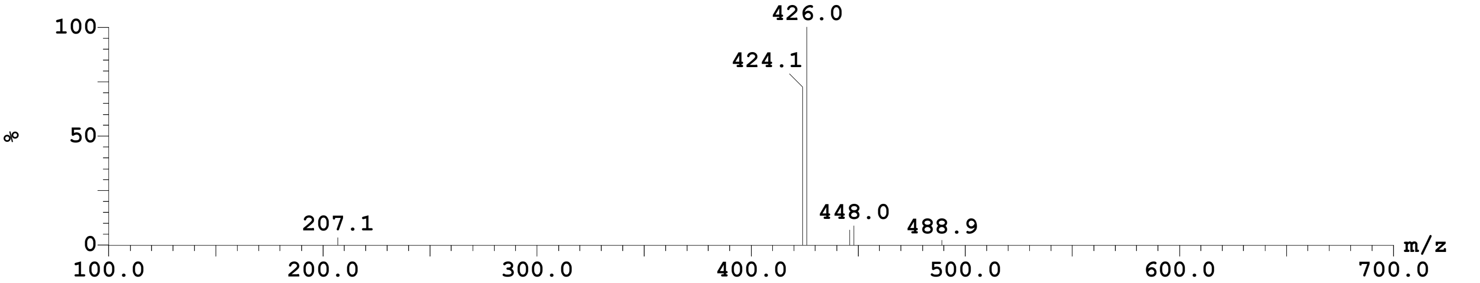

File:13zp867l1 | Vial:5:47 | ID:G1
Method:C:\MASSLYNX\1minLC_MS.olp

Peak ID	Time
3	0.87

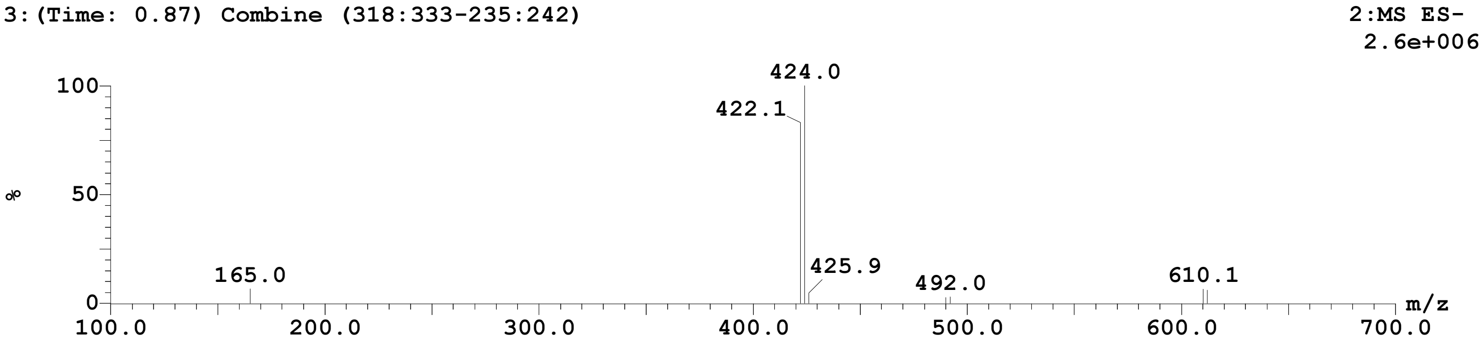

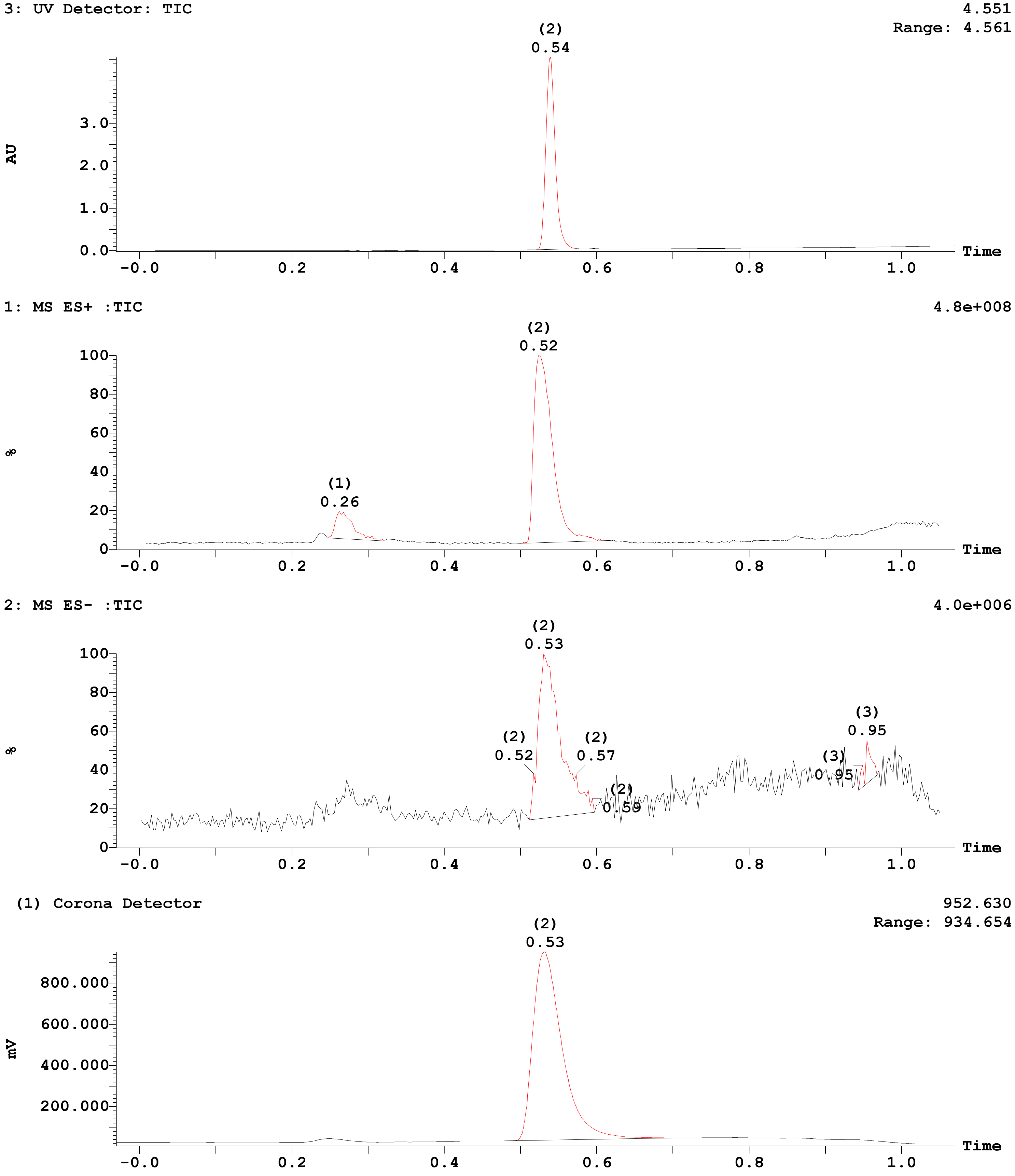
3: UV Detector: TIC
4.551
Range: 4.561
(2)
0.54
AU
Time
1: MS ES+ :TIC
4.8e+008
(1)
0.26
(2)
0.52
%
Time
2: MS ES- :TIC
4.0e+006
(2)
0.53
(2)
0.52
(2)
0.57
(2)
0.59
(3)
0.95
(3)
0.95
%
Time
(1) Corona Detector
952.630
Range: 934.654
(2)
0.53
mV
Time

File:13zp868l1 Vial:5:48 ID:G2
Method:C:\MASSLYNX\1minLC_MS.olp

Peak ID	Time
1	0.26

1:(Time: 0.26) Combine (92:107-(11:18+196:203)) 1:MS ES+ 7.9e+006

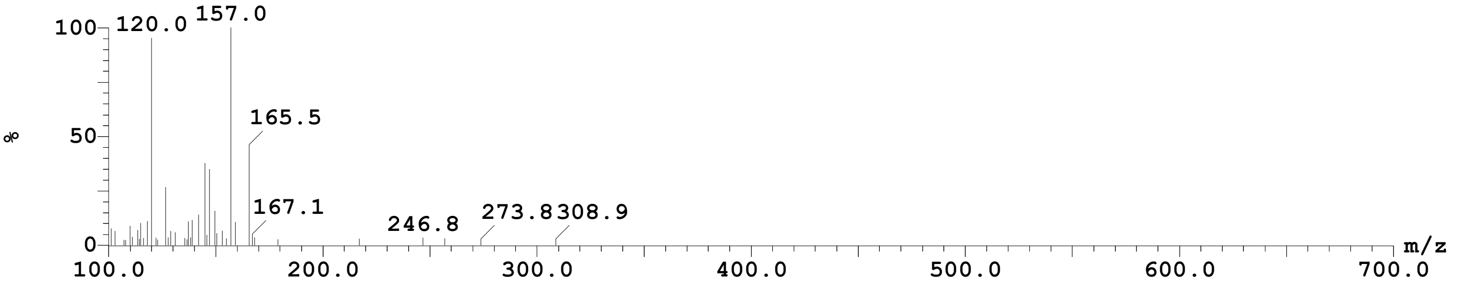

Peak ID	Time
2	0.52

2:(Time: 0.54) Combine (195:210-(113:121+291:298)) 1:MS ES+ 1.0e+008

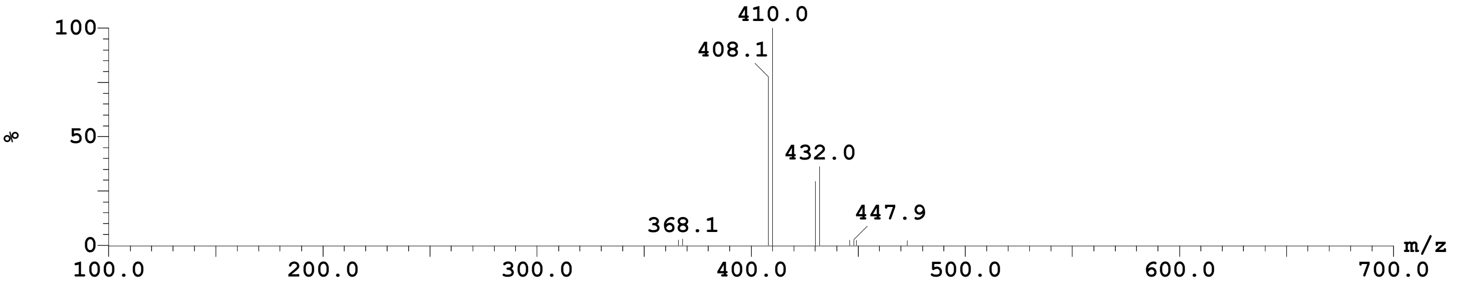

Peak ID	Time
2	0.52

2:(Time: 0.54) Combine (195:210-(113:120+290:298)) 2:MS ES- 9.2e+004

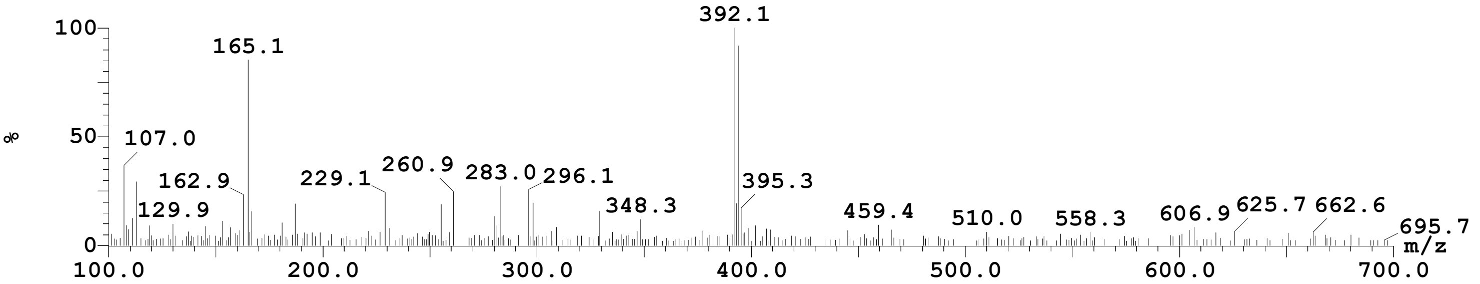

Peak ID	Time
3	0.95

3:(Time: 0.95) Combine (350:365-272:279) 2:MS ES- 1.6e+005

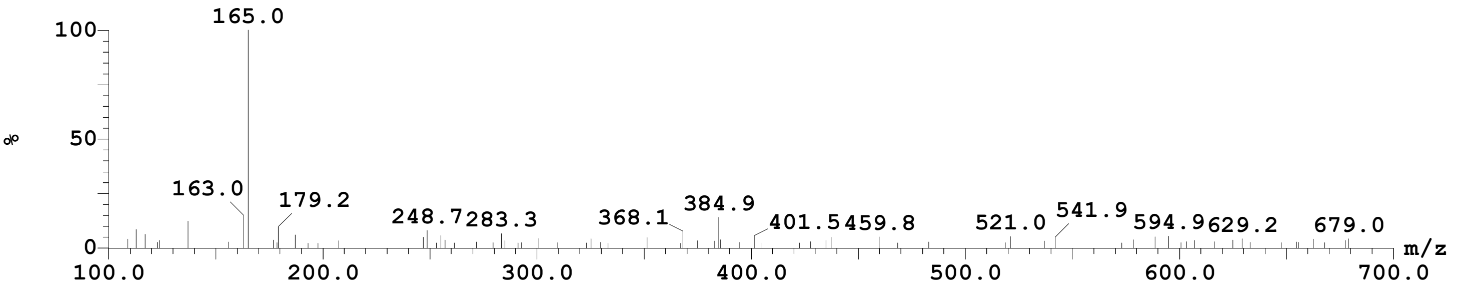

File:13zo156l2
Vial:5:46
ID: **G3**

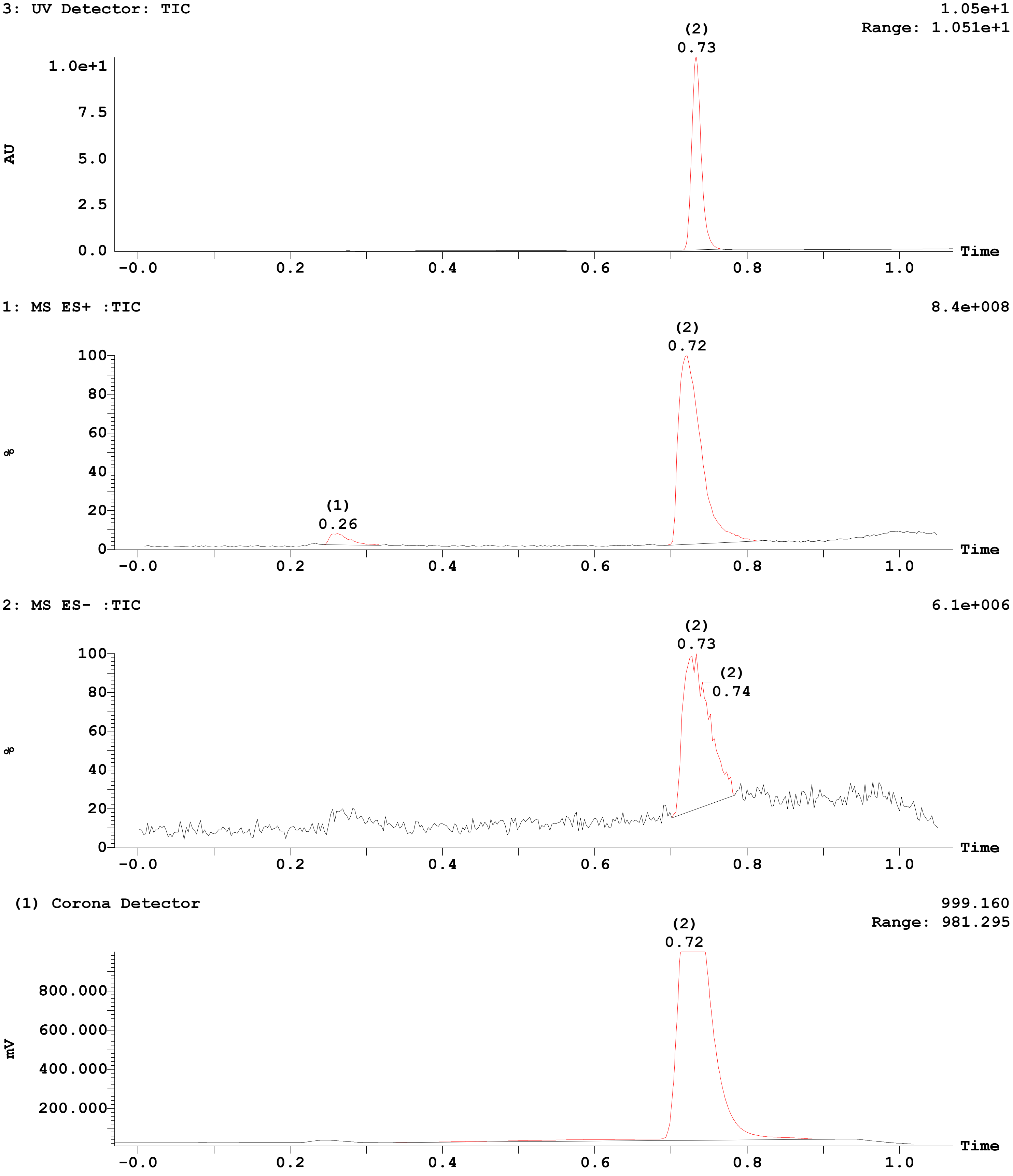

File:13zo156l2 Vial:5:46 ID:G3
Method:C:\MASSLYNX\1minLC_MS.olp

Peak ID	Time
1	0.26

1:(Time: 0.26) Combine (92:106-(10:17+195:202))

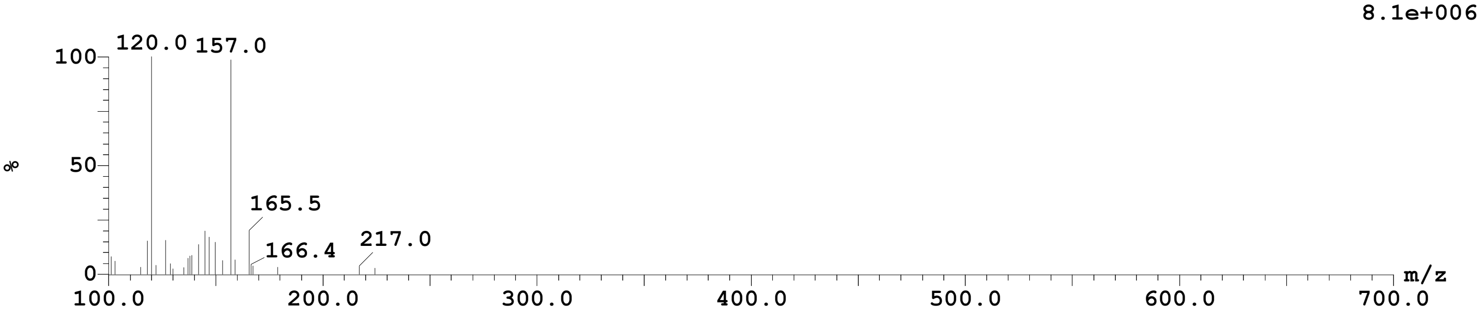

Peak ID	Time
2	0.72

2:(Time: 0.73) Combine (268:283-(186:193+363:371))

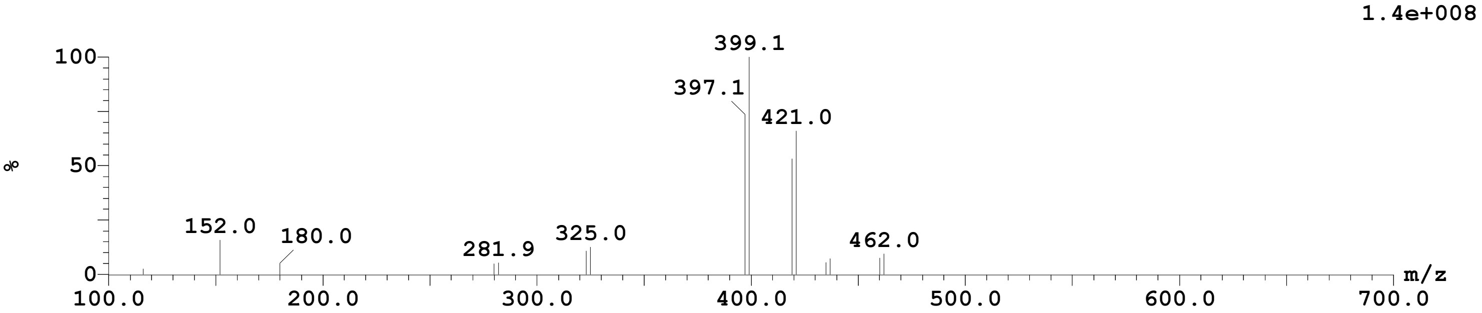

Peak ID	Time
2	0.72

2:(Time: 0.73) Combine (268:282-(181:188+369:376))

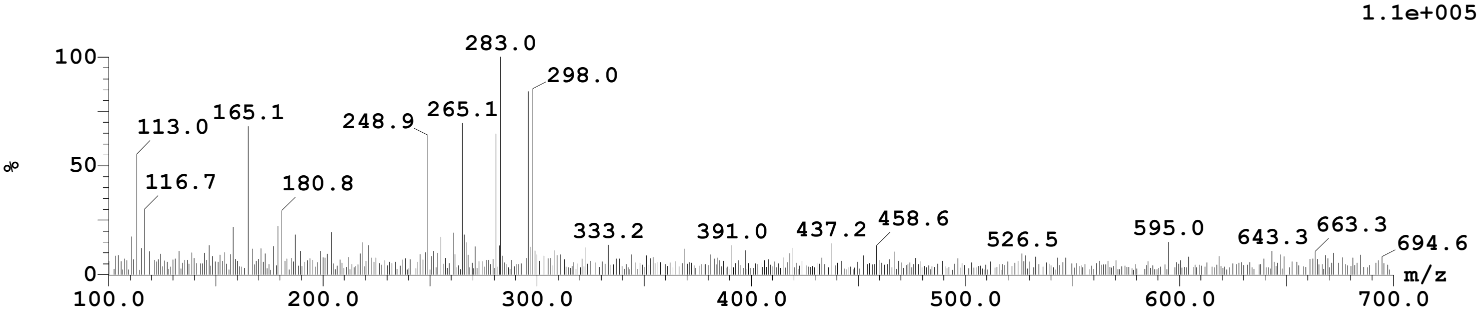

File:13zo190l3 Vial:5:47 ID: **G4**

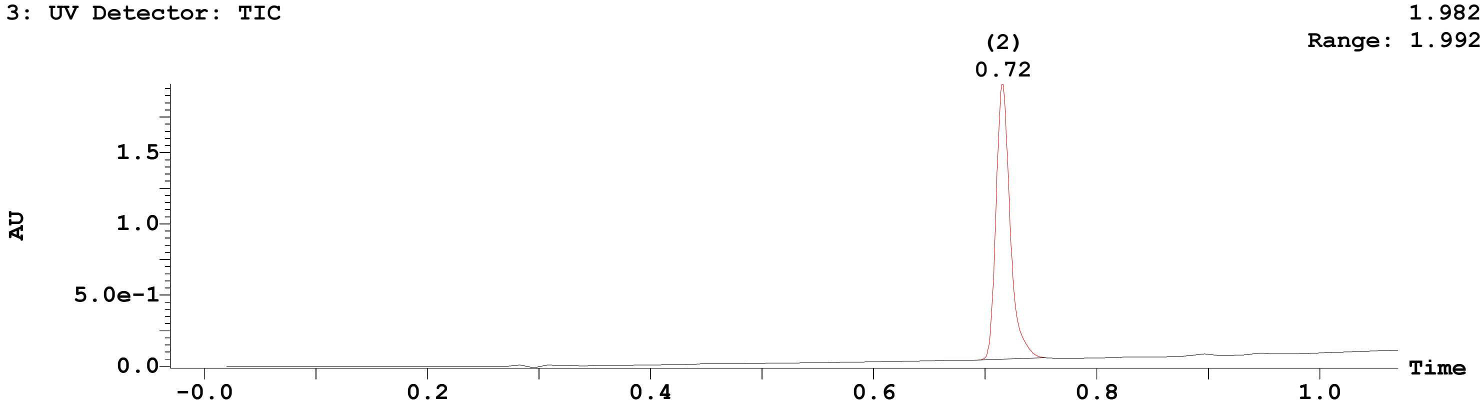

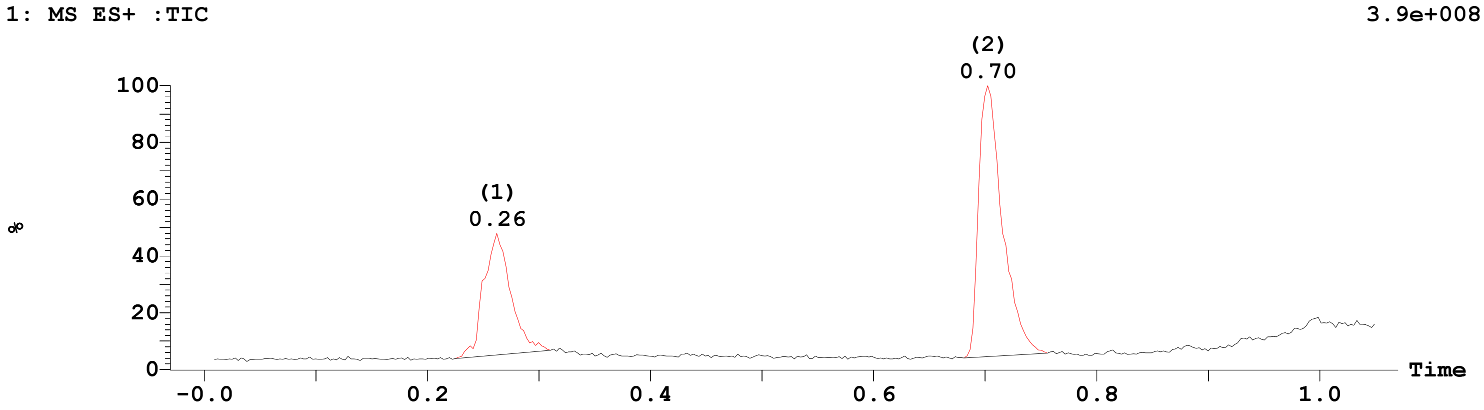

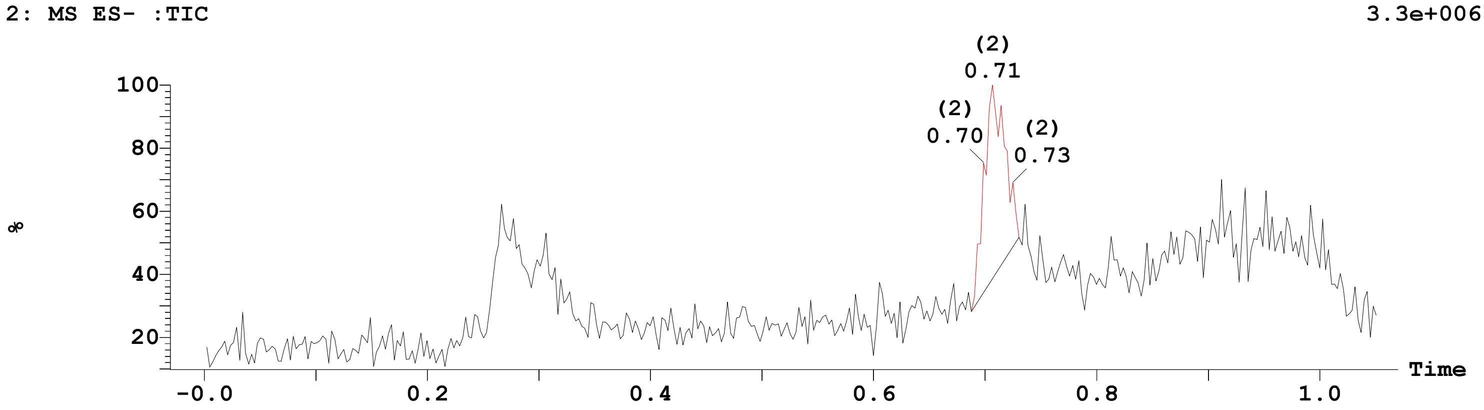

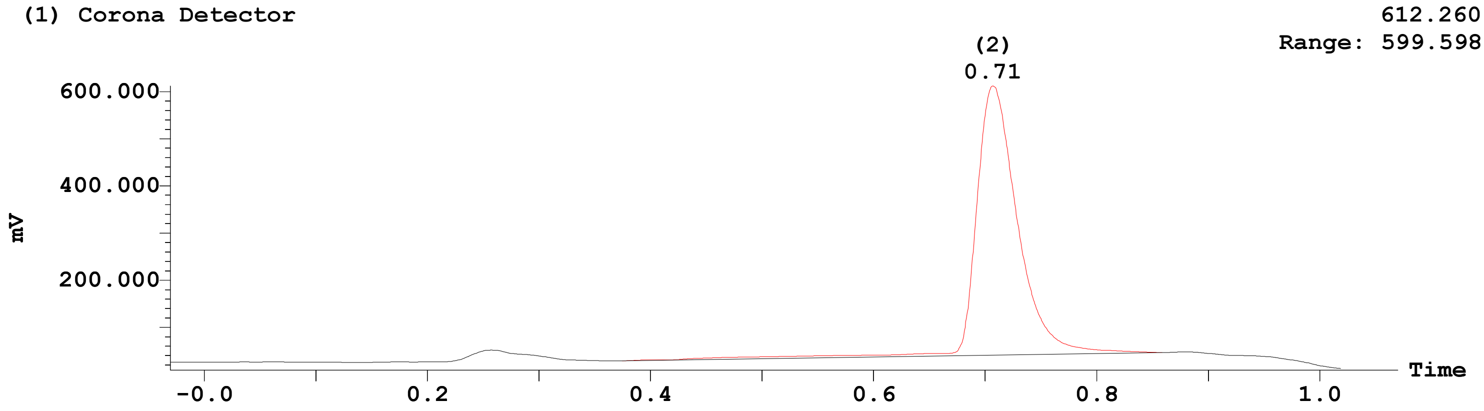

File:13zo190l3 Vial:5:47 ID:G4
Method:C:\MASSLYNX\1minLC_MS.olp

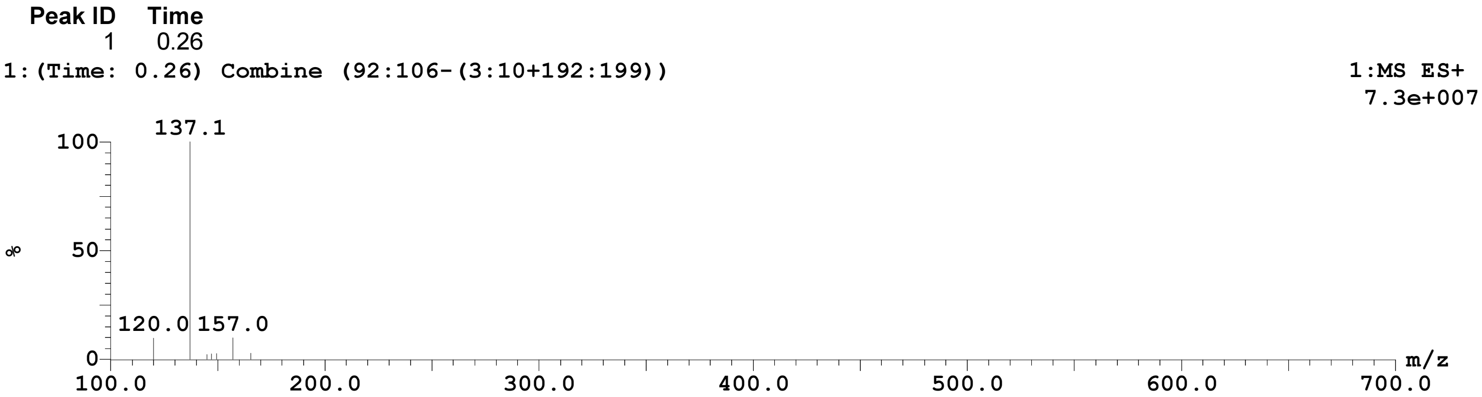

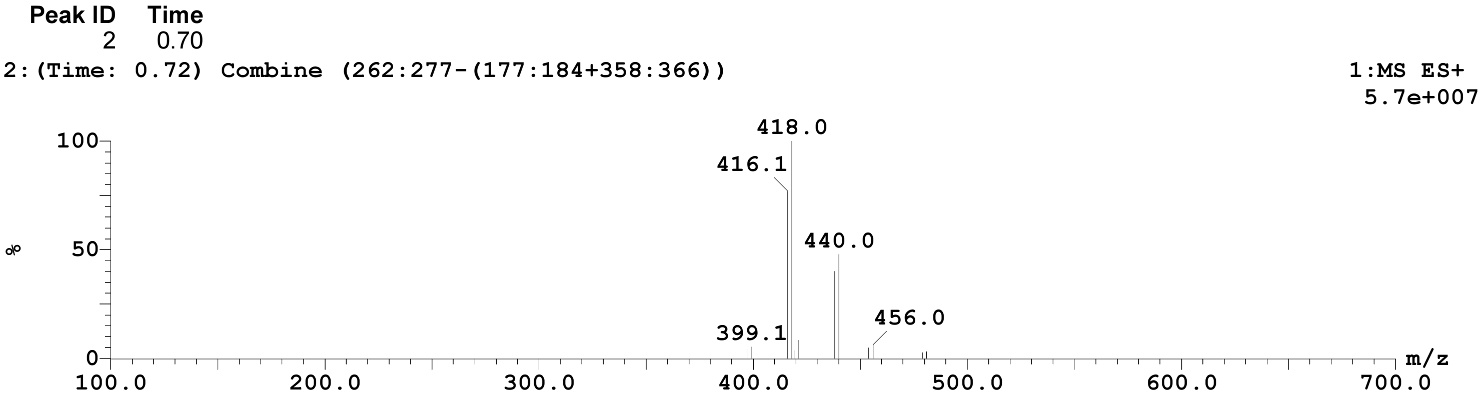

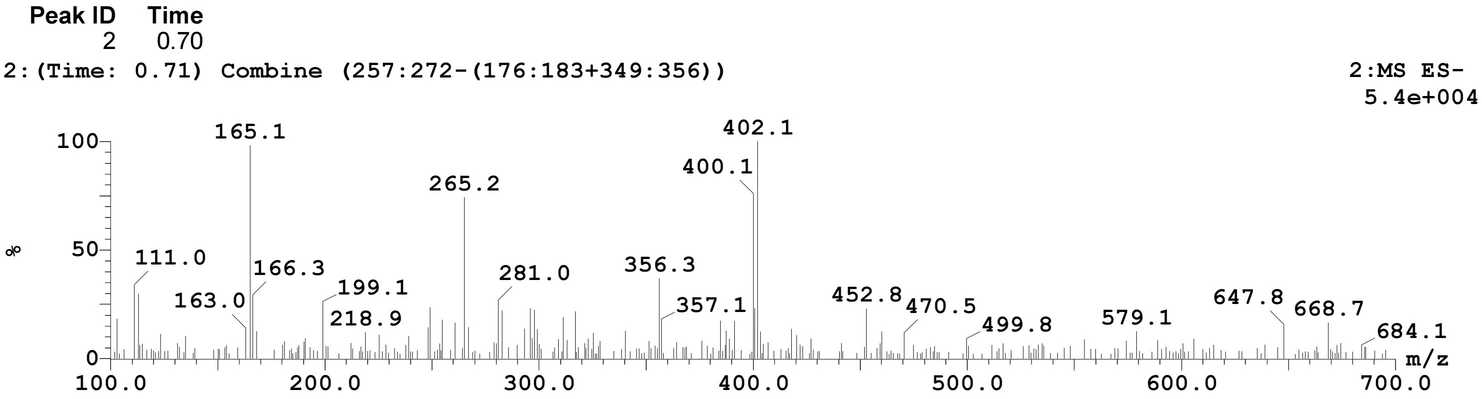

SYNGENTA SQD OPEN ACCESS LC-MS Page 1
File:13zo161l2 Vial:5:48 ID: **G5**

3: UV Detector: TIC 1.667

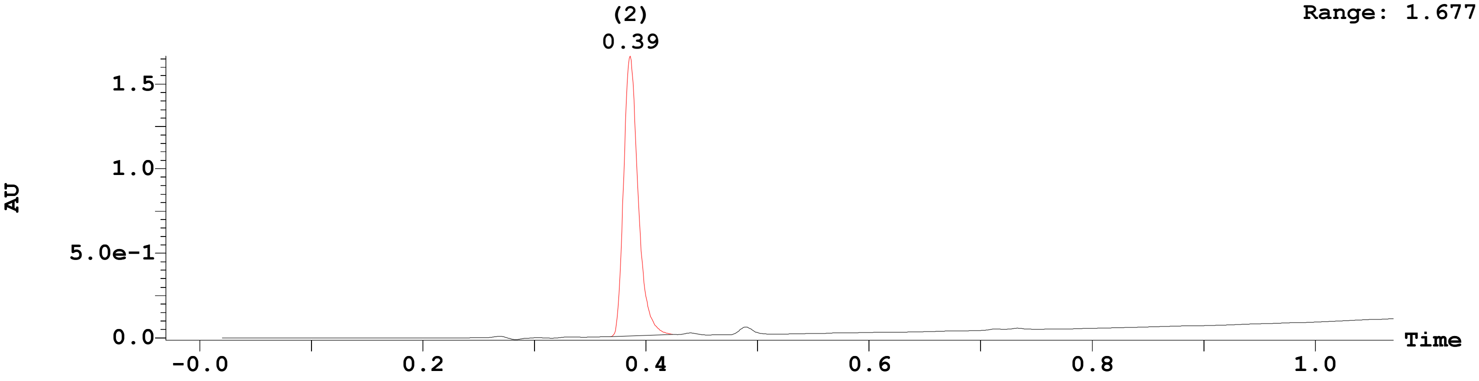

1: MS ES+ :TIC 3.0e+008

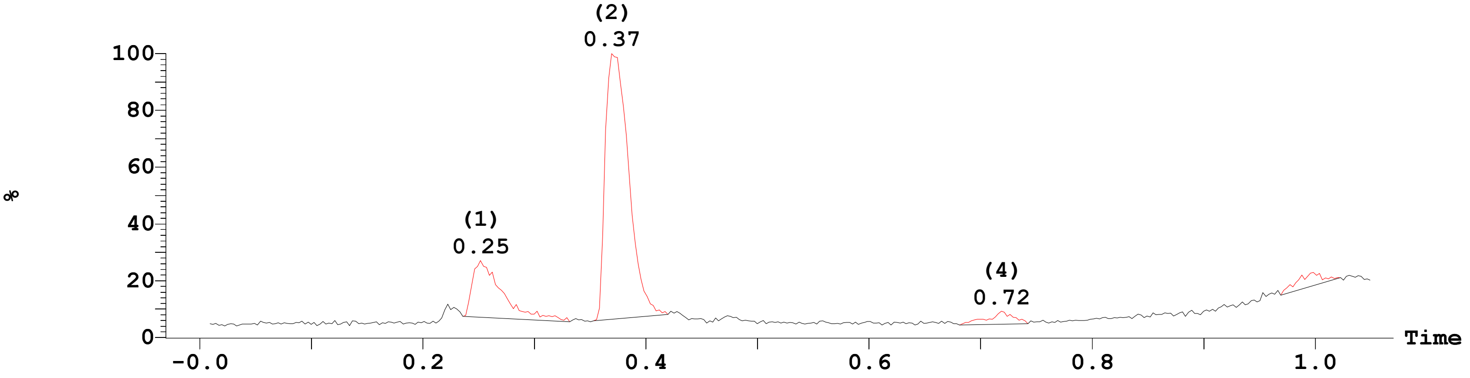

2: MS ES- :TIC 9.4e+006

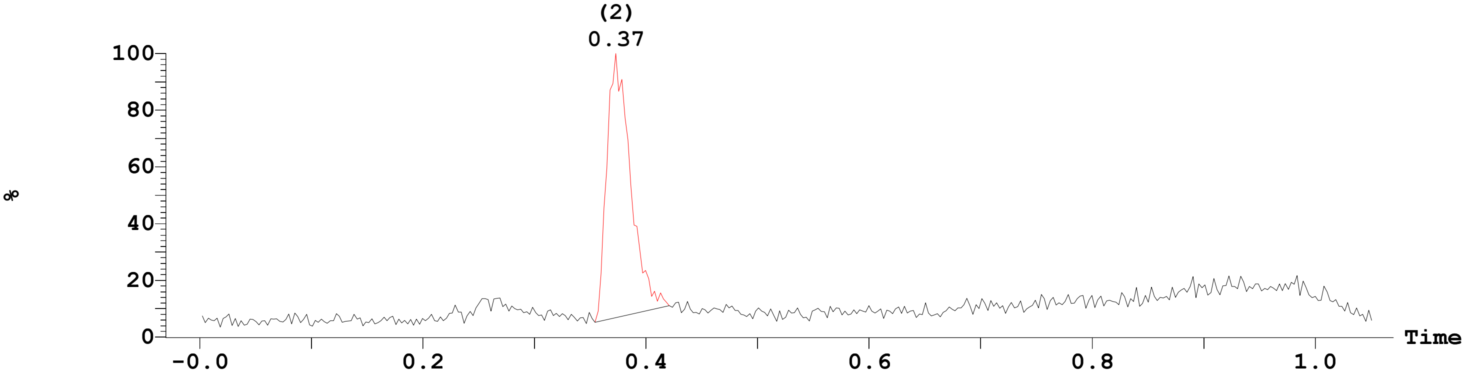

(1) Corona Detector 314.680

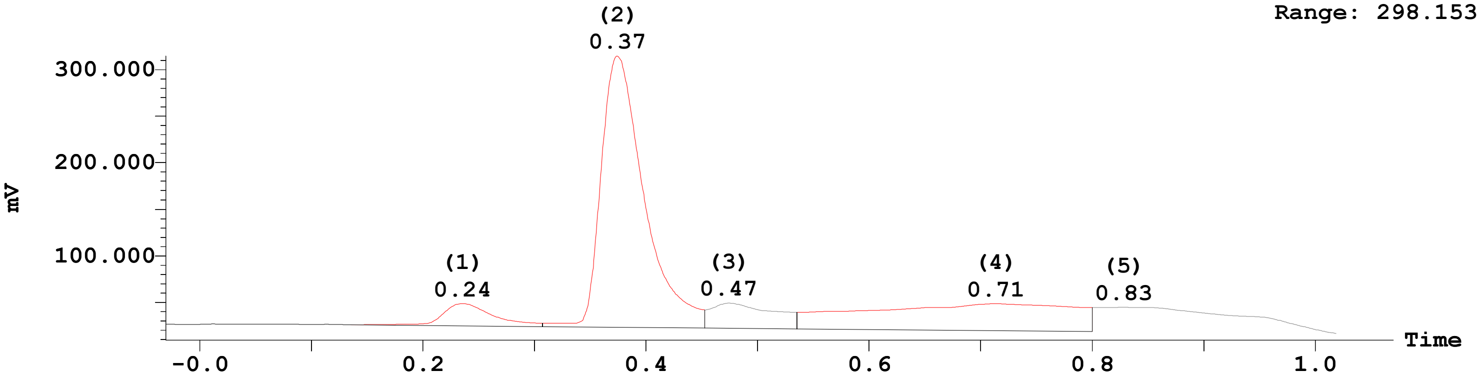

File:13zo161l2 Vial:5:48 ID:G5
Method:C:\MASSLYNX\1minLC_MS.olp

Peak ID	Time
1	0.25

1:(Time: 0.25) Combine (88:102-(7:14+200:207)) 1:MS ES+ 8.0e+006

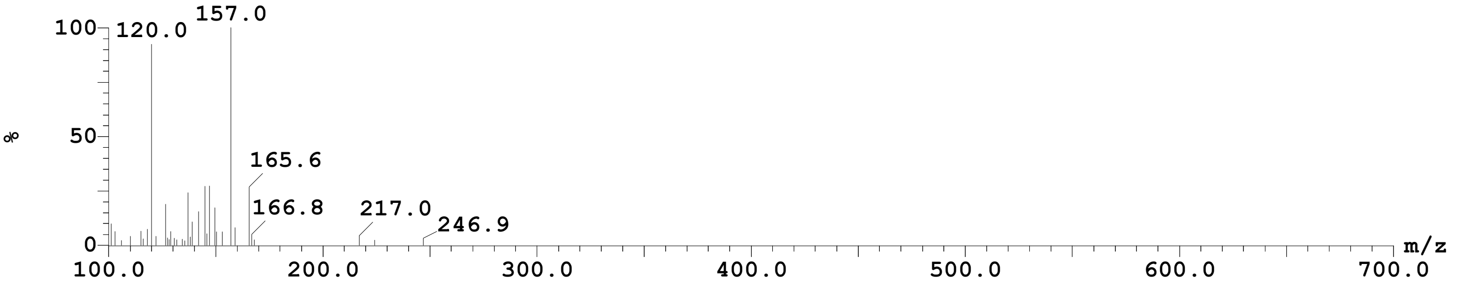

Peak ID	Time
2	0.37

2:(Time: 0.39) Combine (138:153-(56:63+235:242)) 1:MS ES+ 7.8e+007

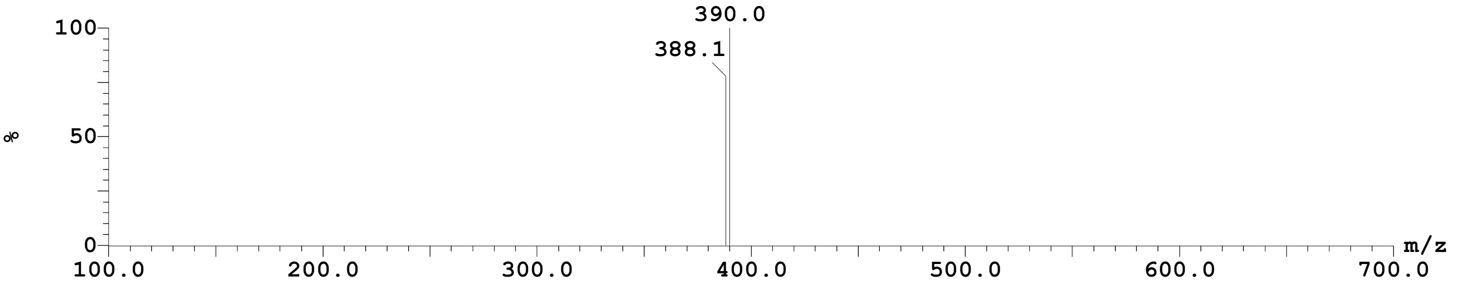

Peak ID	Time
2	0.37

2:(Time: 0.37) Combine (132:147-(51:58+233:240)) 2:MS ES- 1.3e+006

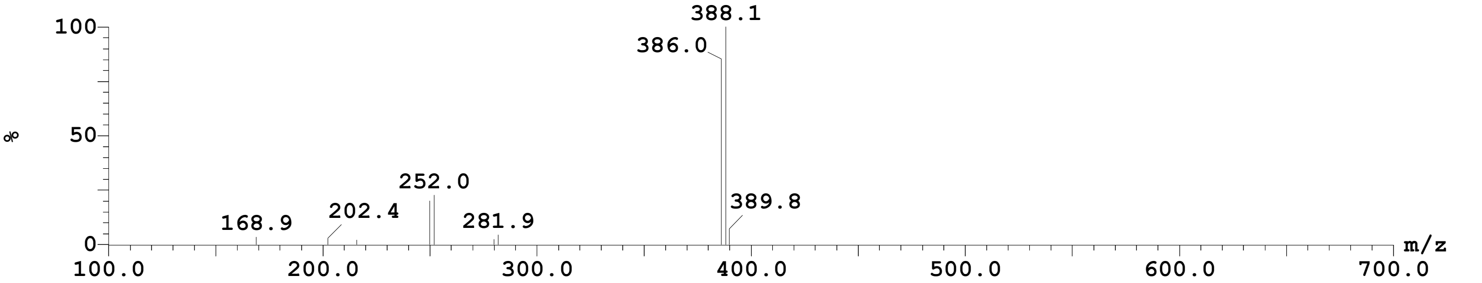

Peak ID	Time
4	0.72

4:(Time: 0.72) Combine (263:277-(174:181+354:361)) 1:MS ES+ 1.0e+006

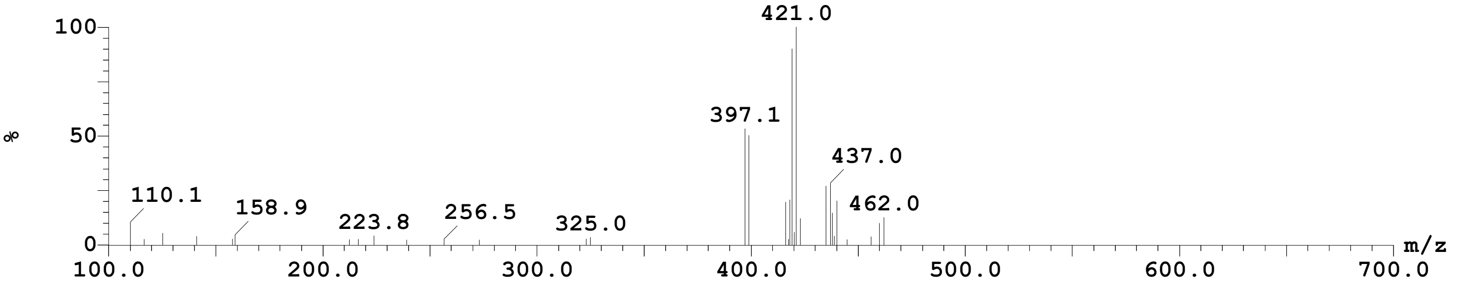

File:13zo161l2 Vial:5:48 ID:G5

Peak ID	Time
6	1.00

6: (Time: 1.00) Combine (367:383-282:289)

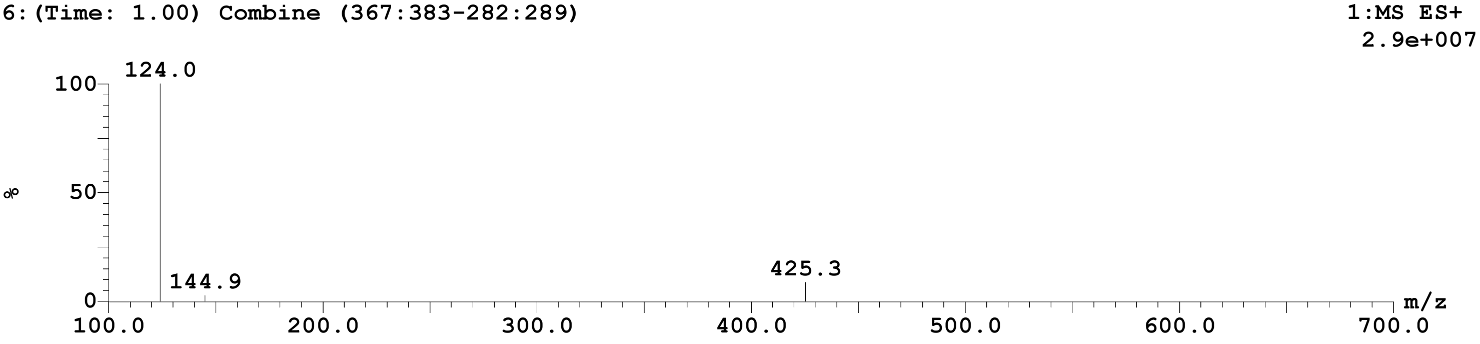

File:13zo179l3 Vial:5:49 ID: **G6**

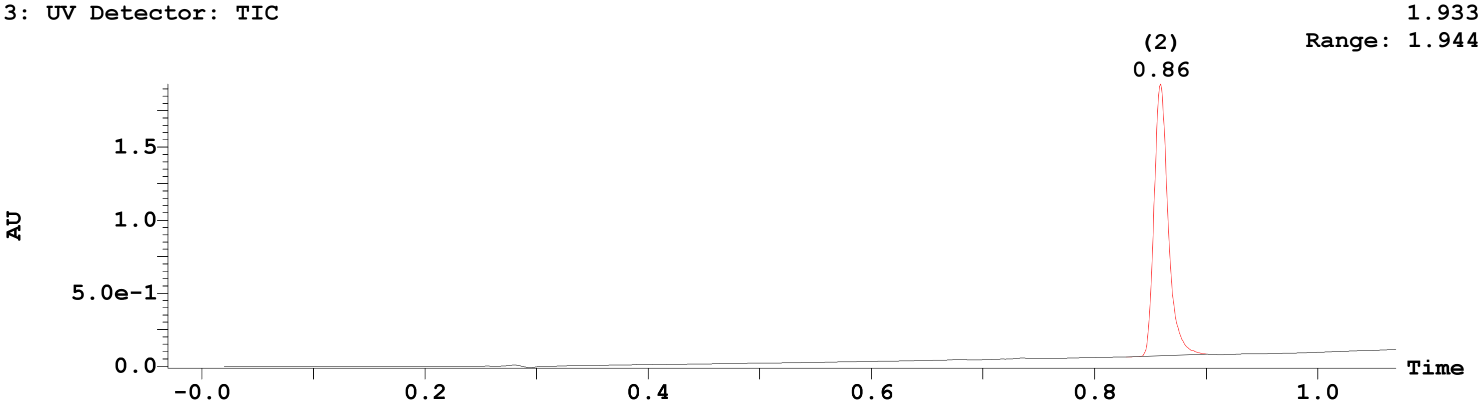

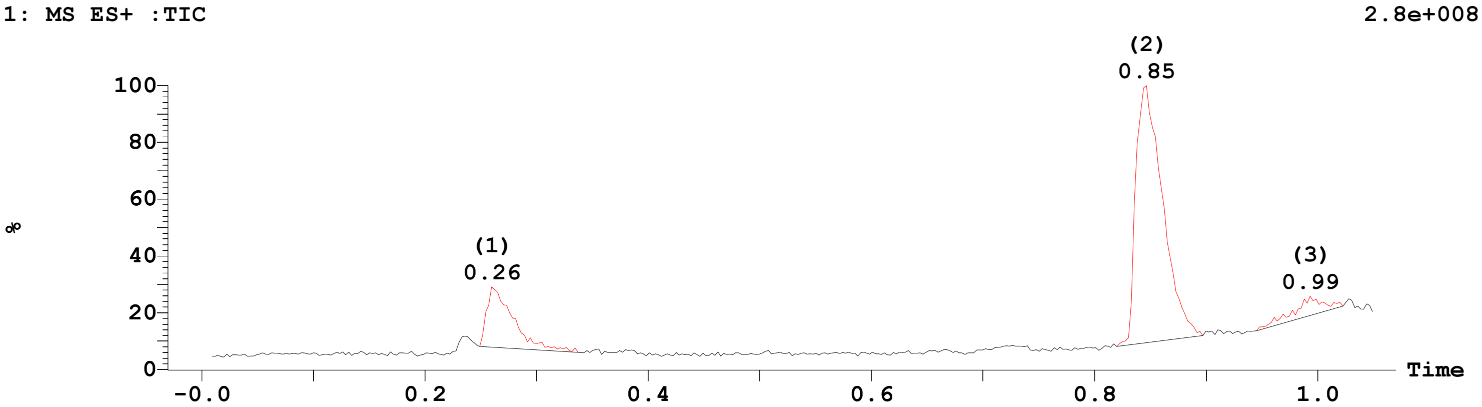

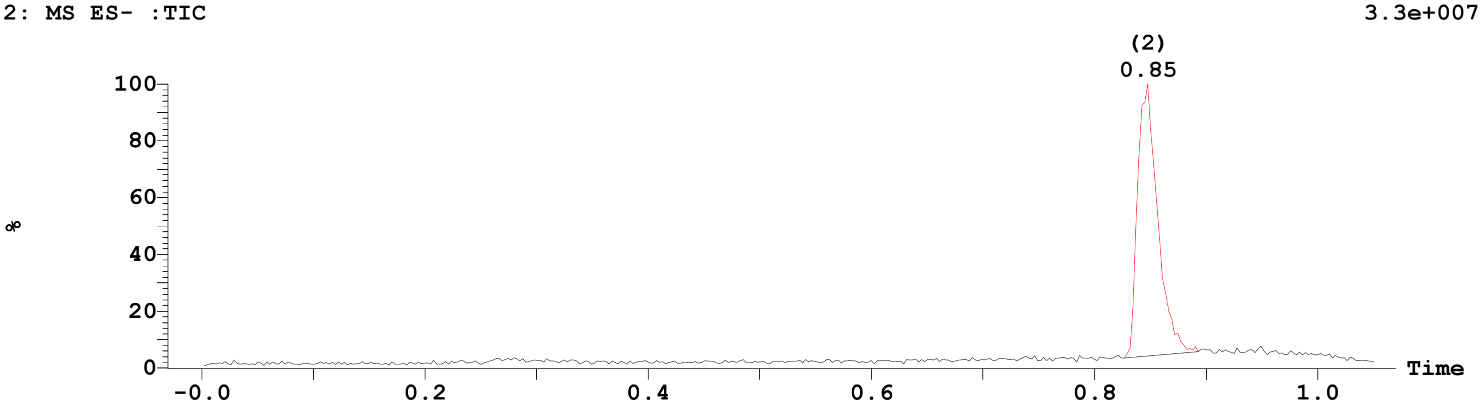

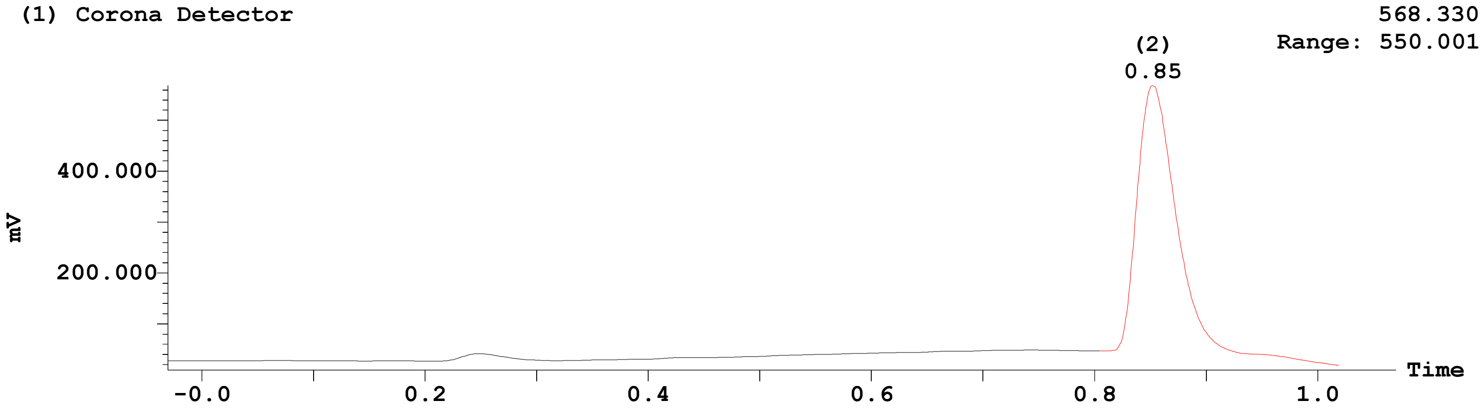

File:13zo179l3 Vial:5:49 ID:G6
Method:C:\MASSLYNX\1minLC_MS.olp

Peak ID	Time
1	0.26

1:(Time: 0.26) Combine (90:105-(12:19+203:210)) 1:MS ES+

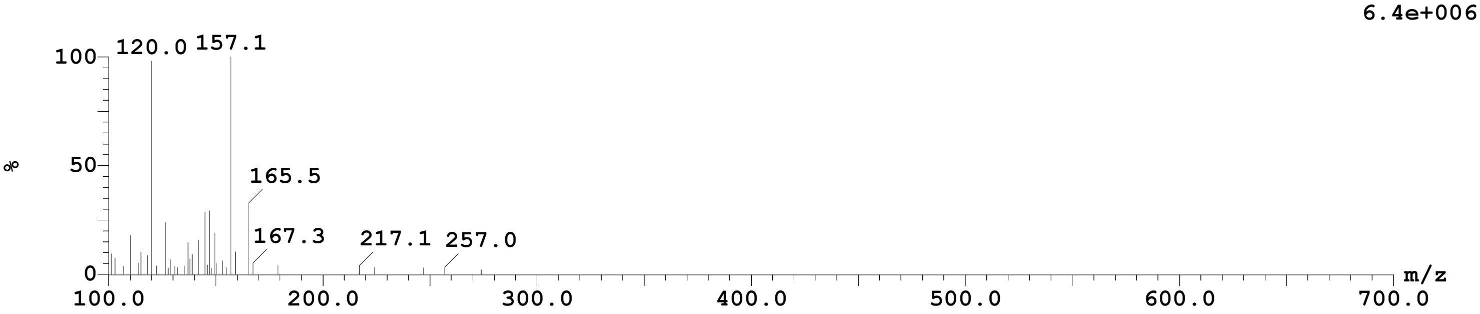

Peak ID	Time
2	0.85

2:(Time: 0.86) Combine (315:330-229:236) 1:MS ES+

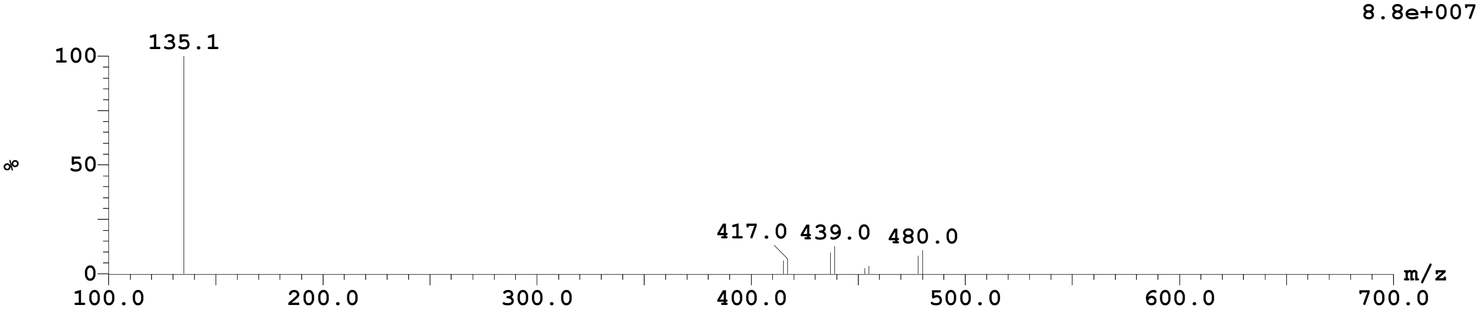

Peak ID	Time
2	0.85

2:(Time: 0.85) Combine (311:326-(228:235+410:413)) 2:MS ES-

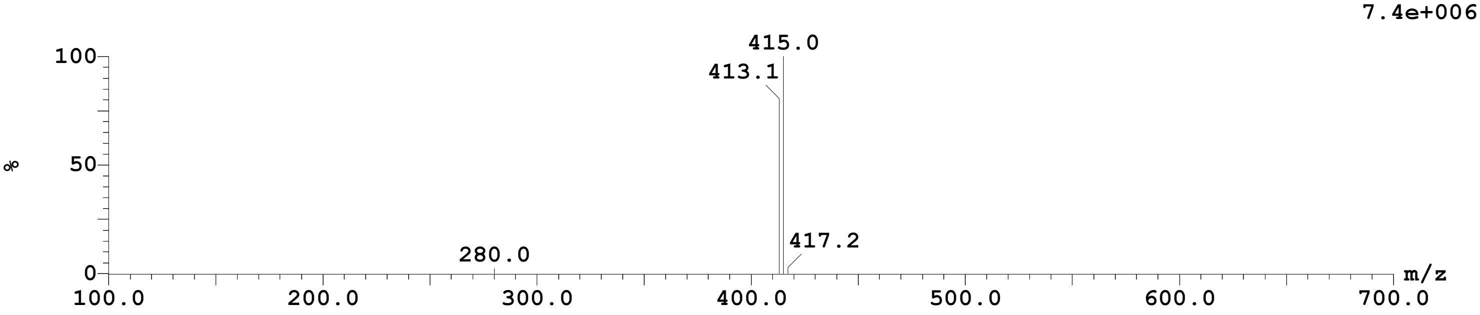

Peak ID	Time
3	0.99

3:(Time: 0.99) Combine (366:381-273:280) 1:MS ES+

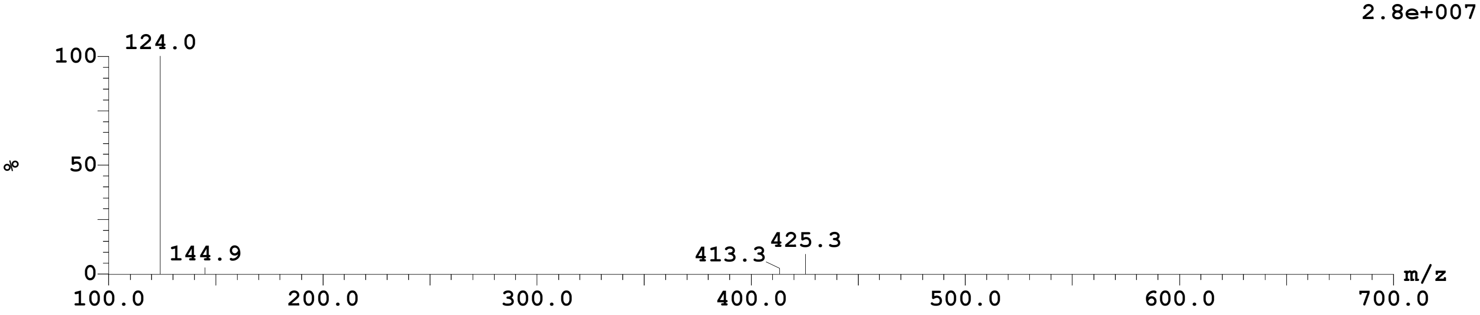

SYNGENTA SQD OPEN ACCESS LC-MS Page 1

File:13zo242l2 Vial:5:46 ID: **G7**

3: UV Detector: TIC 9.172

Range: 9.181

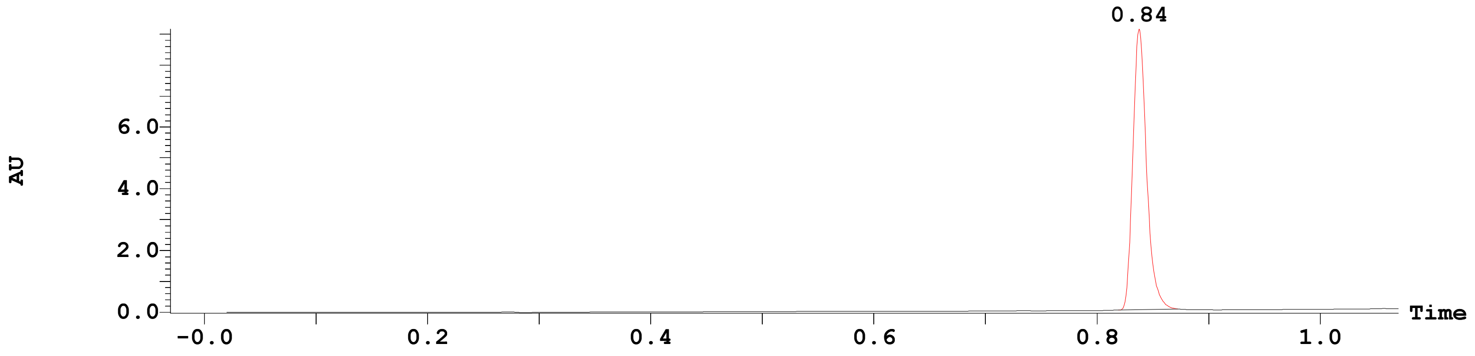

1: MS ES+ :TIC 2.7e+008

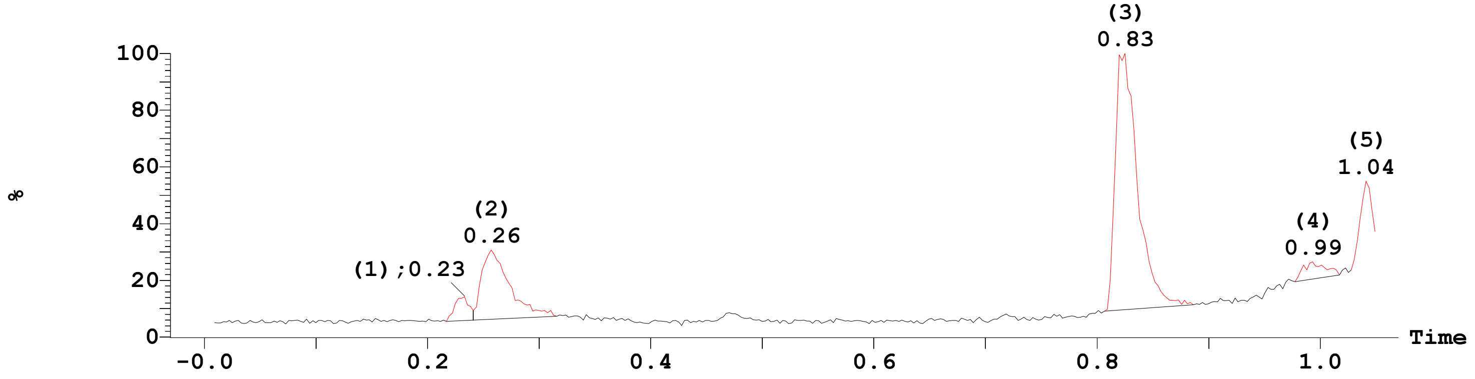

2: MS ES- :TIC 1.5e+008

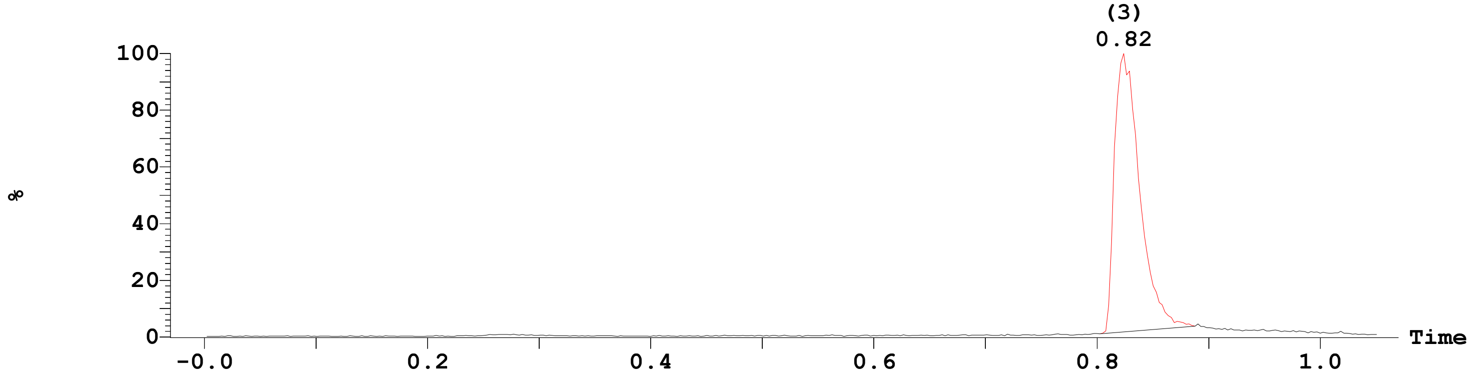

(1) Corona Detector 999.160

Range: 978.987

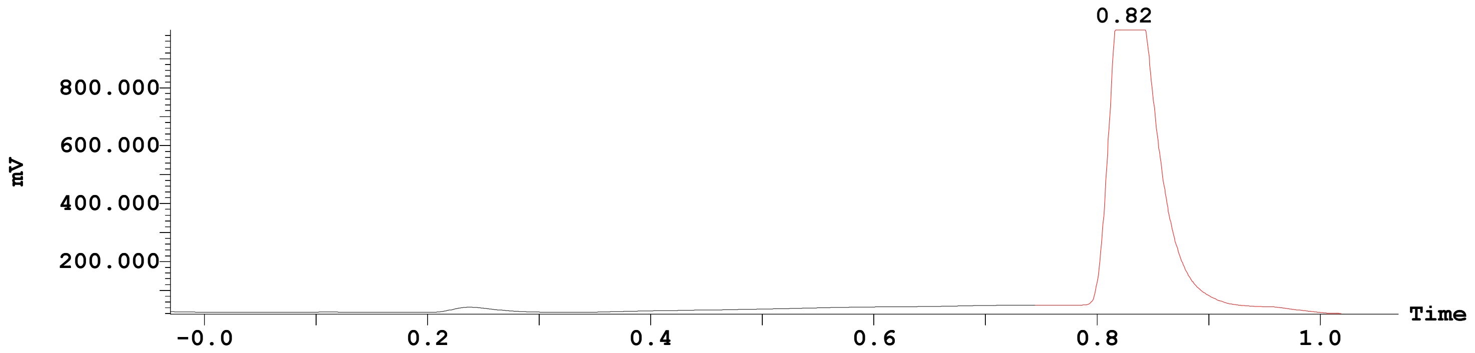

File:13zo242l2 Vial:5:46 ID:G7
Method:C:\MASSLYNX\1minLC_MS.olp

Peak ID **Time**
1 0.23

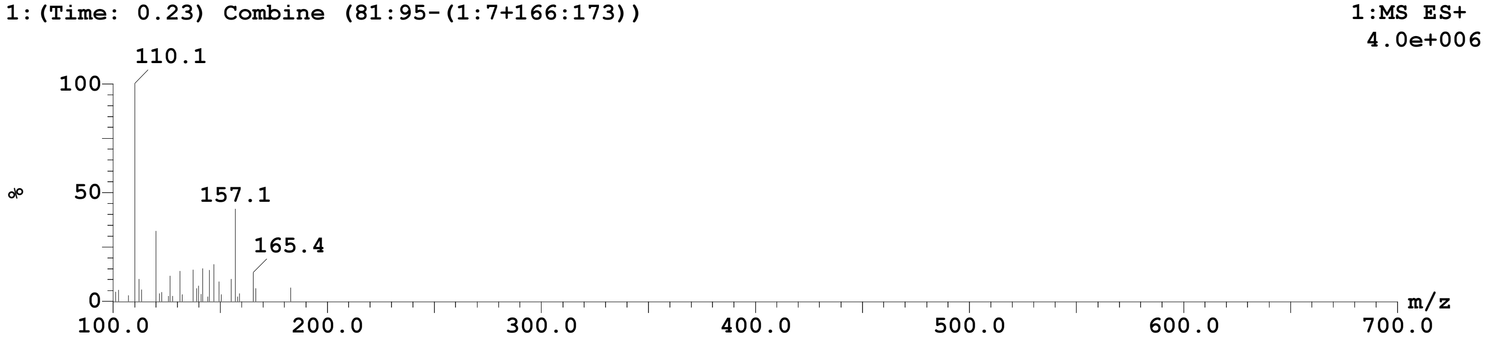

Peak ID **Time**
2 0.26

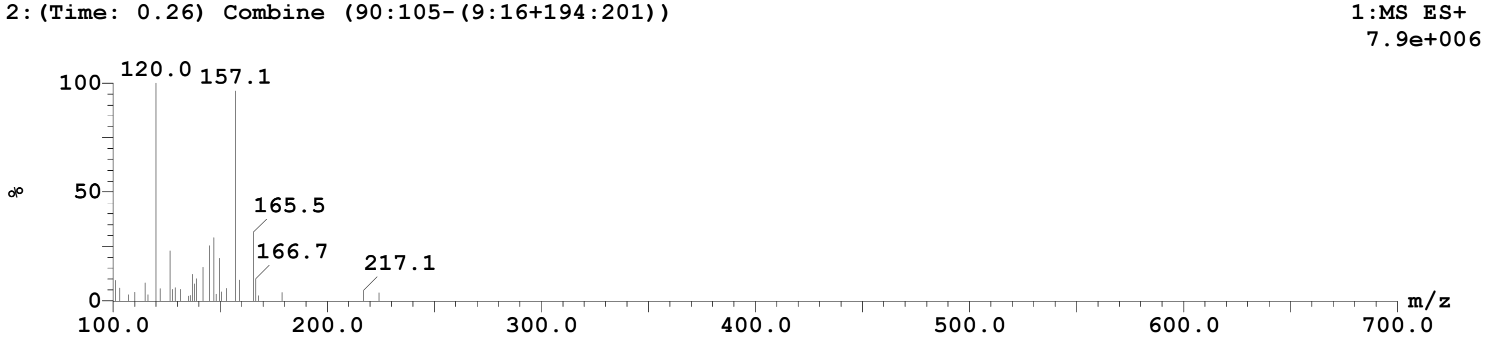

Peak ID **Time**
3 0.83

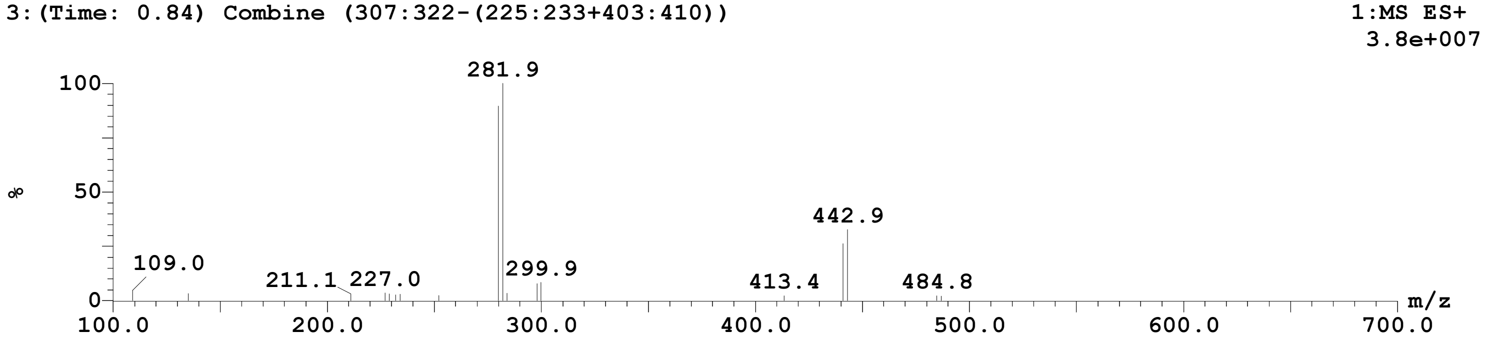

Peak ID **Time**
3 0.83

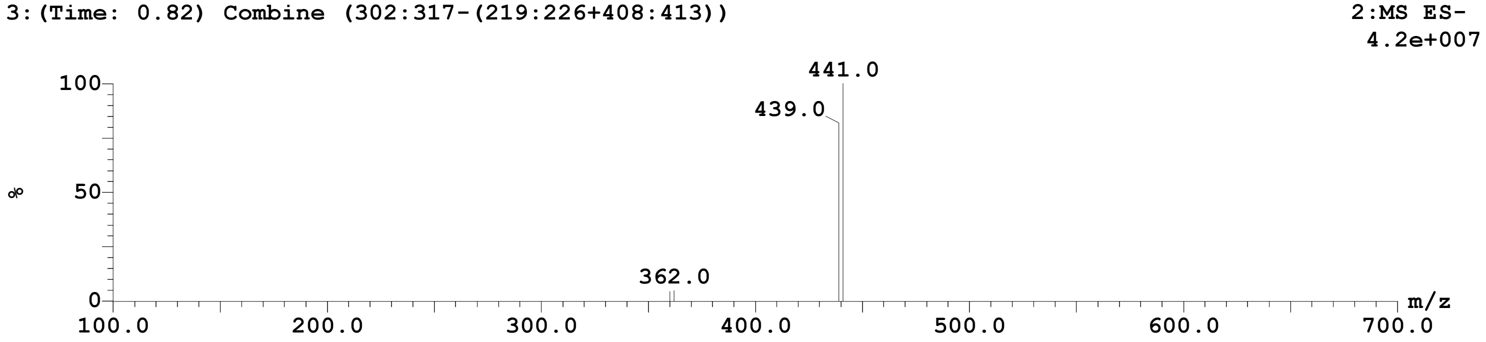

File:13zo242l2 Vial:5:46 ID:G7
Method:C:\MASSLYNX\1minLC_MS.olp

Peak ID **Time**
4 0.99

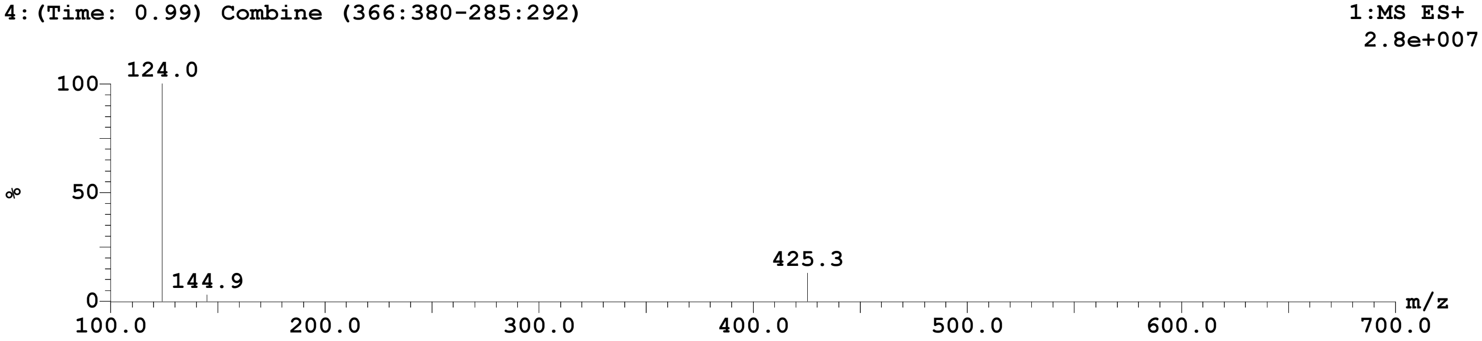

Peak ID **Time**
5 1.04

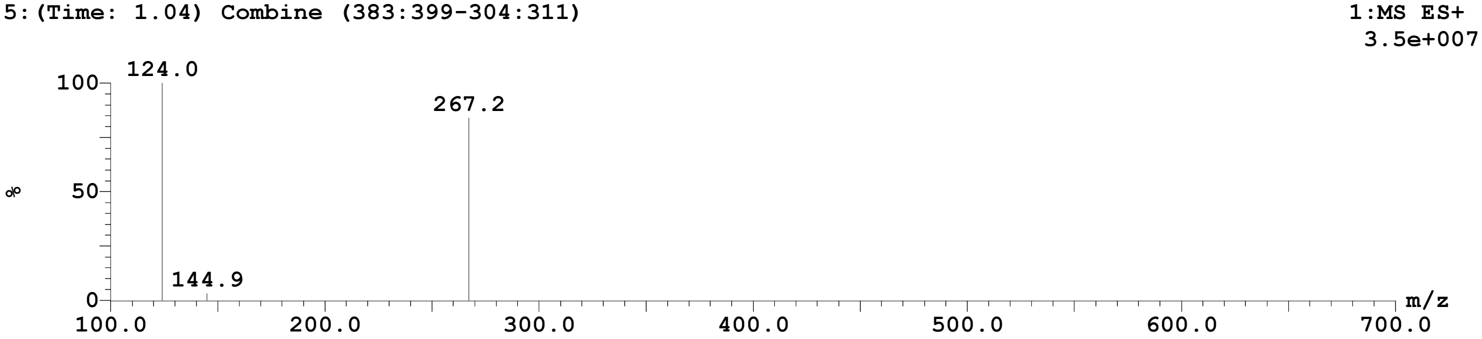

File:13zo310l2 Vial:5:47 ID: **G8**

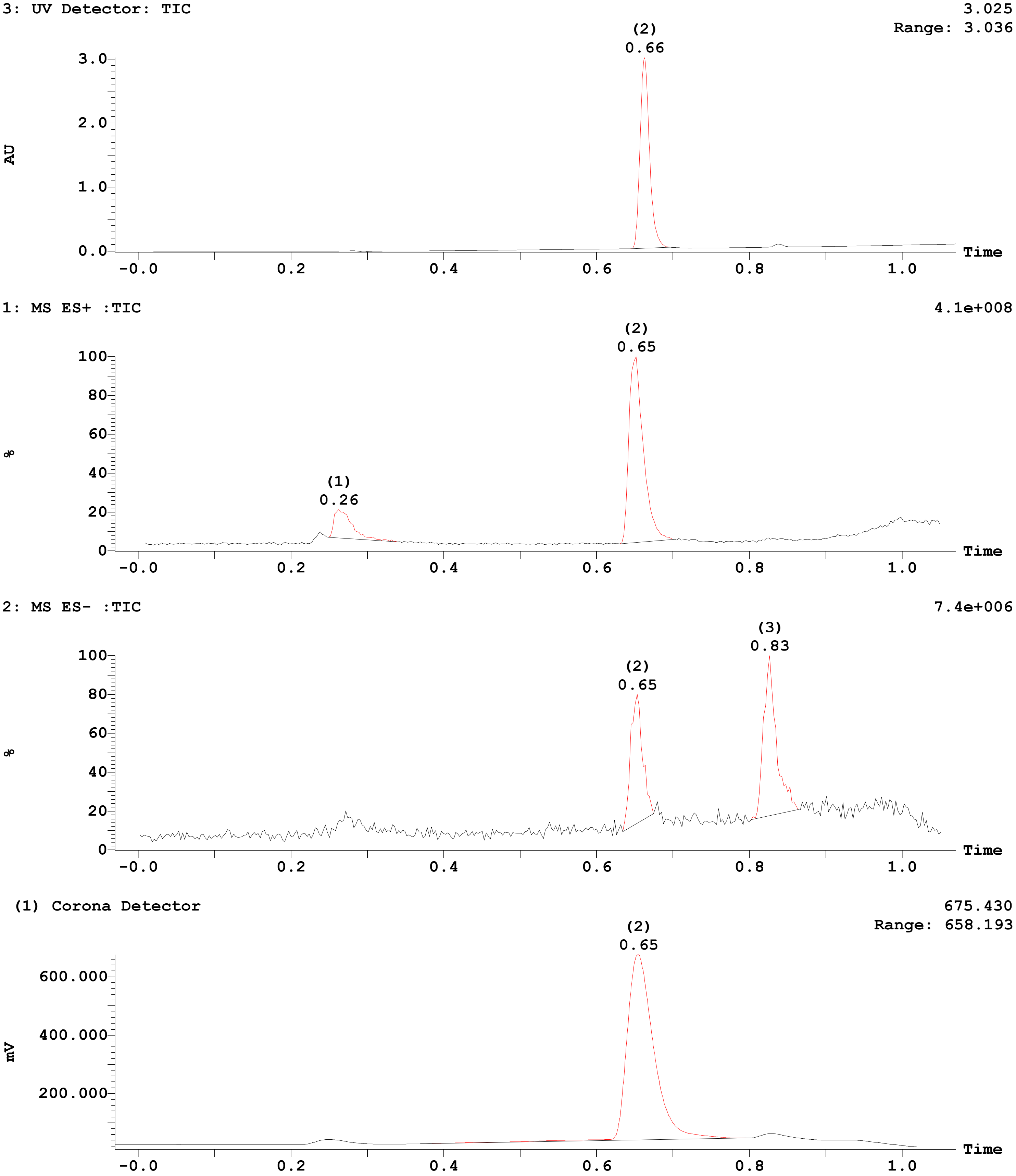

File:13zo310l2 Vial:5:47 ID:G8
Method:C:\MASSLYNX\1minLC_MS.olp

Peak ID **Time**
1 0.26

1:(Time: 0.26) Combine (92:107-(12:19+203:210)) 1:MS ES+ 7.6e+006

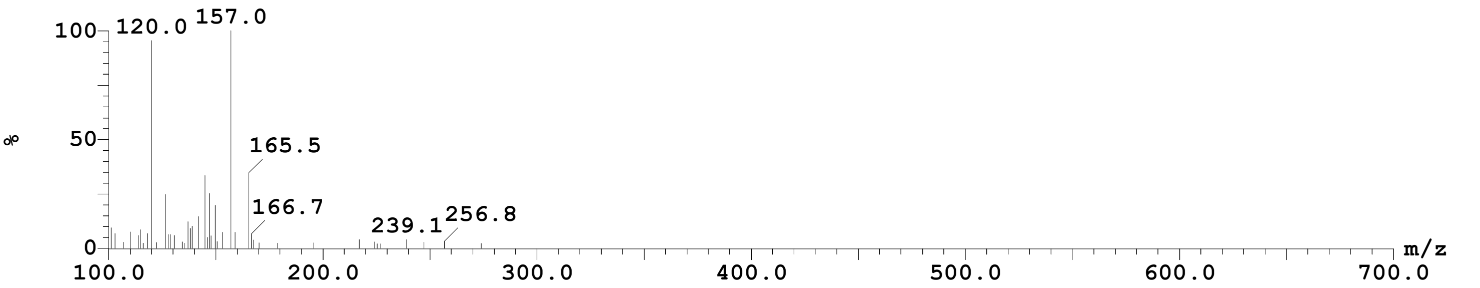

Peak ID **Time**
2 0.65

2:(Time: 0.66) Combine (242:257-(160:167+337:344)) 1:MS ES+ 4.5e+007

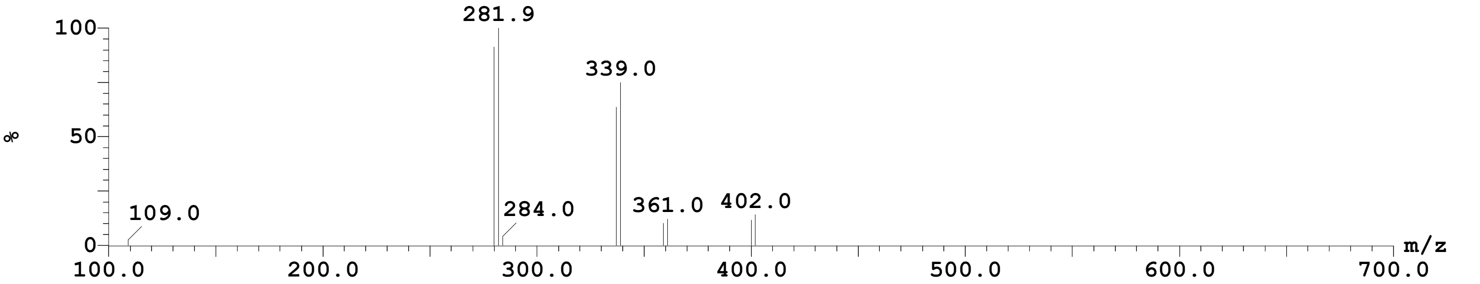

Peak ID **Time**
2 0.65

2:(Time: 0.66) Combine (241:256-(159:167+336:344)) 2:MS ES- 3.6e+005

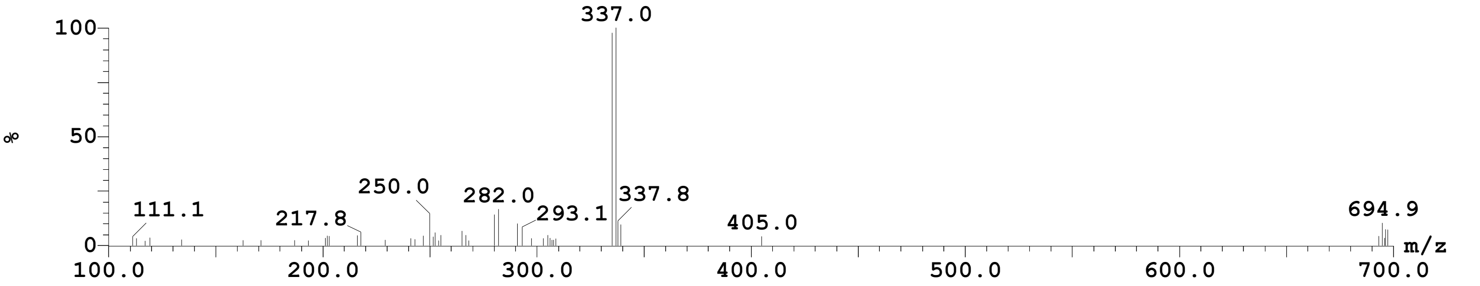

Peak ID **Time**
3 0.83

3:(Time: 0.83) Combine (303:317-(219:226+399:406)) 2:MS ES- 1.5e+006

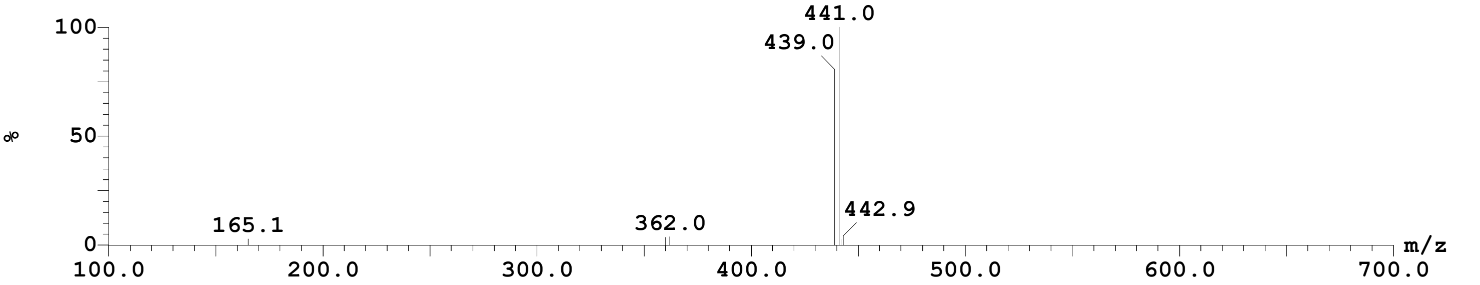

SYNGENTA SQD OPEN ACCESS LC-MS Page 1

File:13zp871I1 Vial:5:48 ID: **G9**

3: UV Detector: TIC 6.997

Range: 7.006

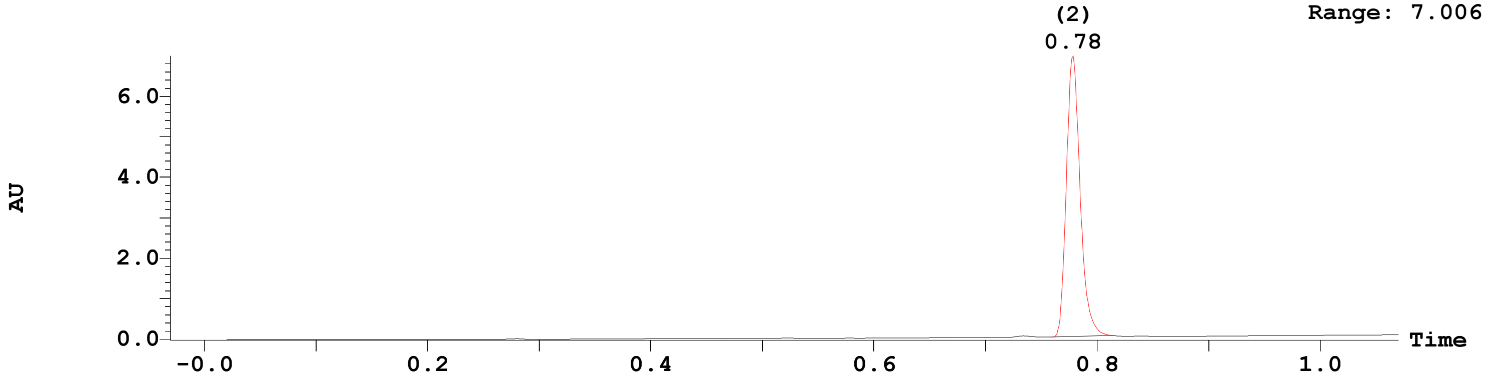

1: MS ES+ :TIC 4.5e+008

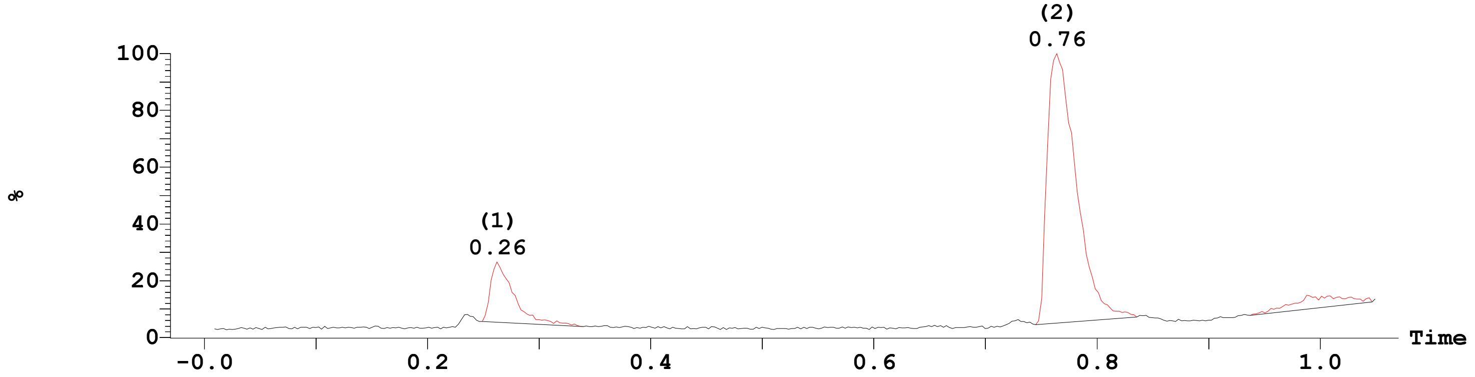

2: MS ES- :TIC 4.1e+007

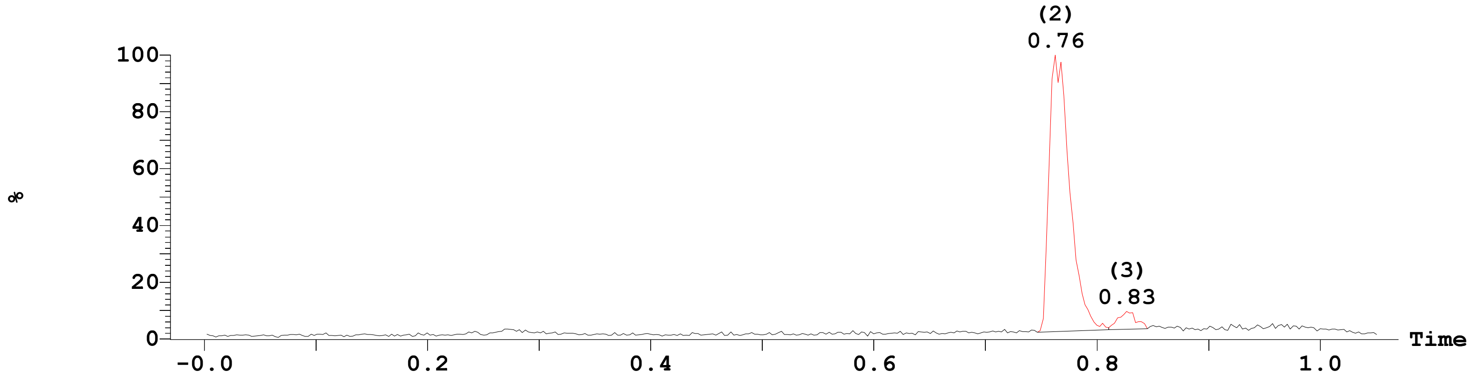

(1) Corona Detector 999.180

Range: 981.215

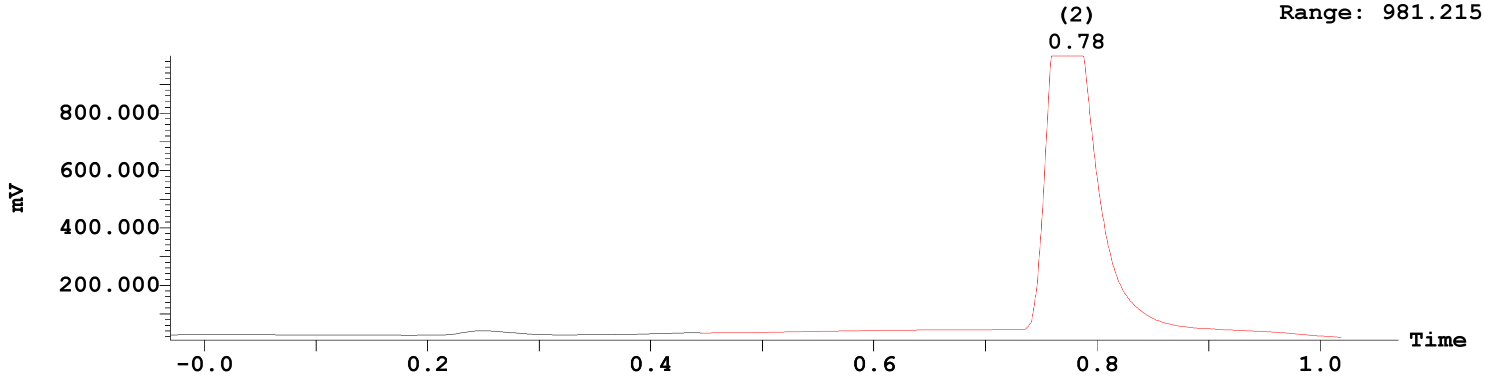

File:13zp871l1 Vial:5:48 ID:G9
Method:C:\MASSLYNX\1minLC_MS.olp

Peak ID **Time**
1 0.26

1:(Time: 0.26) Combine (92:107-(12:19+203:210)) 1:MS ES+ 1.5e+007

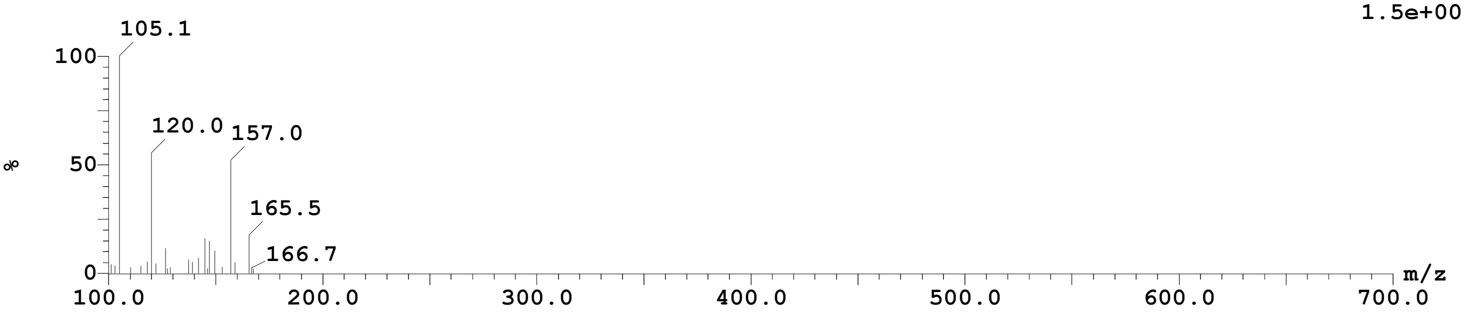

Peak ID **Time**
2 0.76

2:(Time: 0.78) Combine (285:300-(202:210+381:388)) 1:MS ES+ 9.1e+007

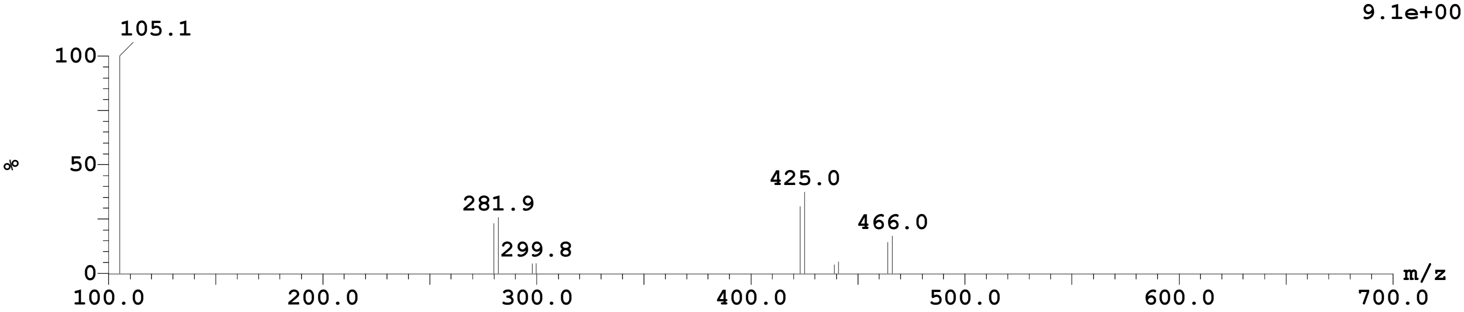

Peak ID **Time**
2 0.76

2:(Time: 0.78) Combine (284:299-(202:209+380:388)) 2:MS ES- 7.8e+006

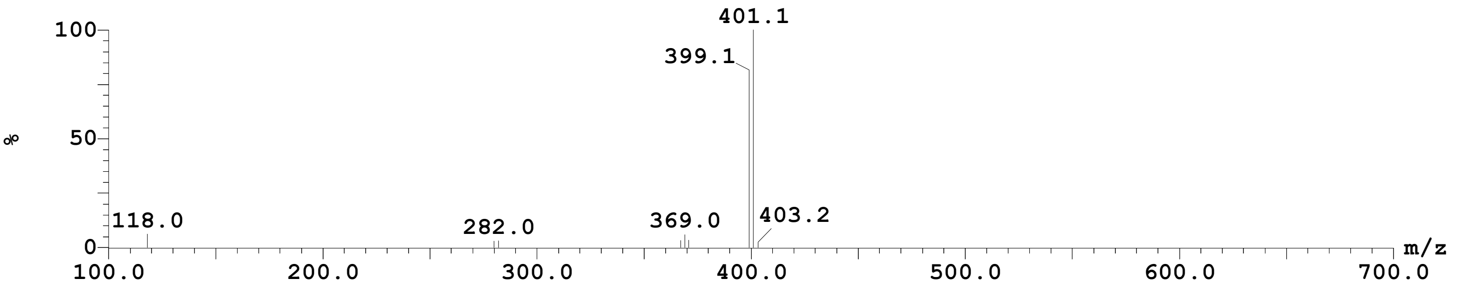

Peak ID **Time**
3 0.83

3:(Time: 0.83) Combine (303:318-(222:229+392:399)) 2:MS ES- 4.9e+005

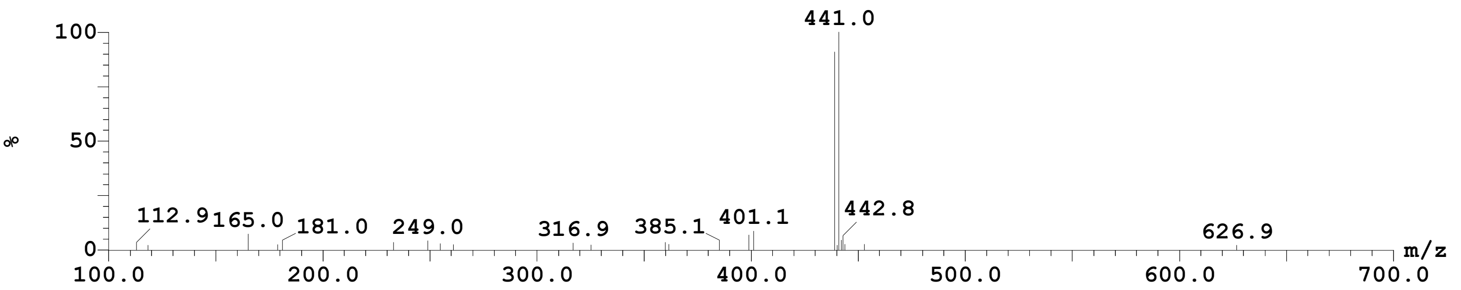

File:13zp871I1 Vial:5:48 ID:G9
Method:C:\MASSLYNX\1minLC_MS.olp

Peak ID **Time**
4 0.99

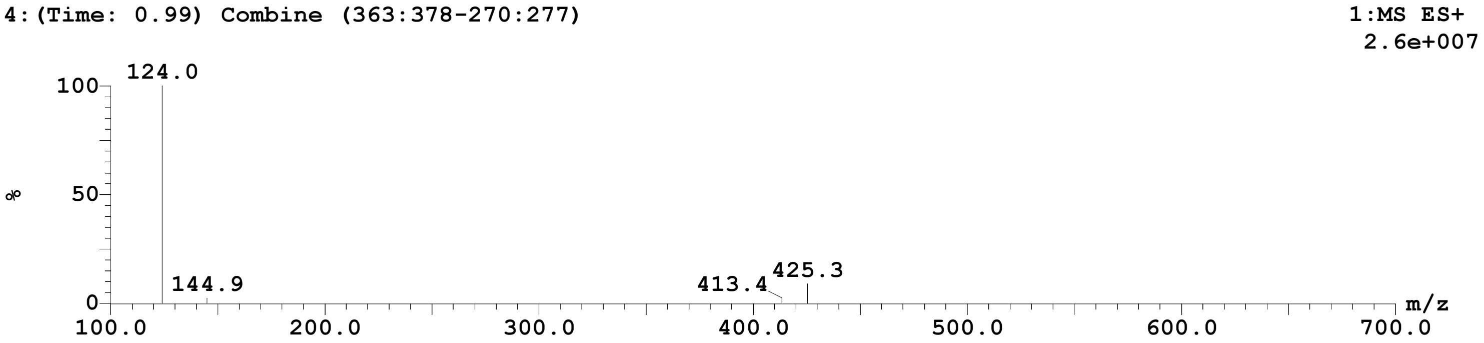

File:13zp872I1 Vial:5:49 ID: **G10**

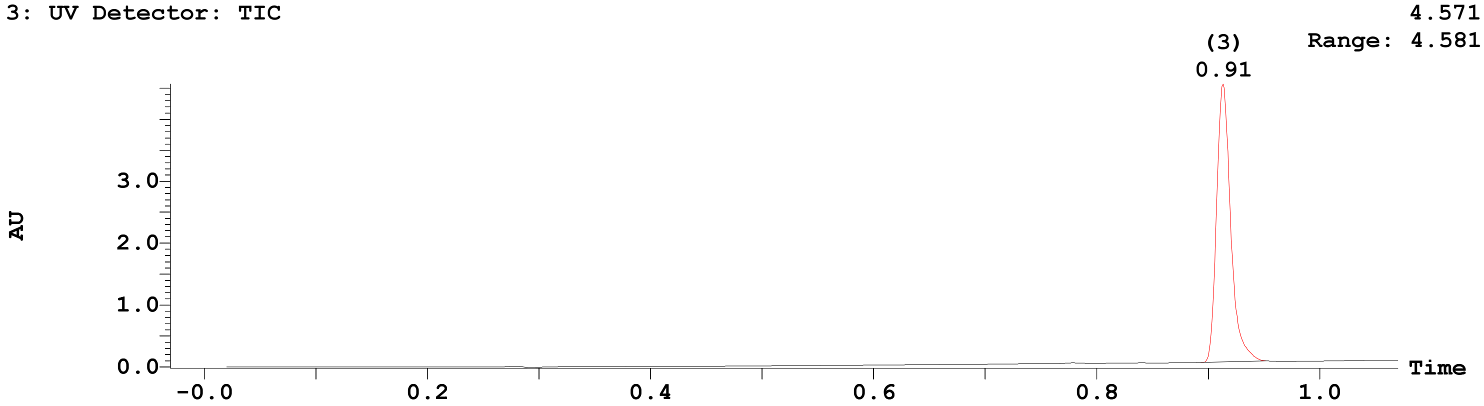

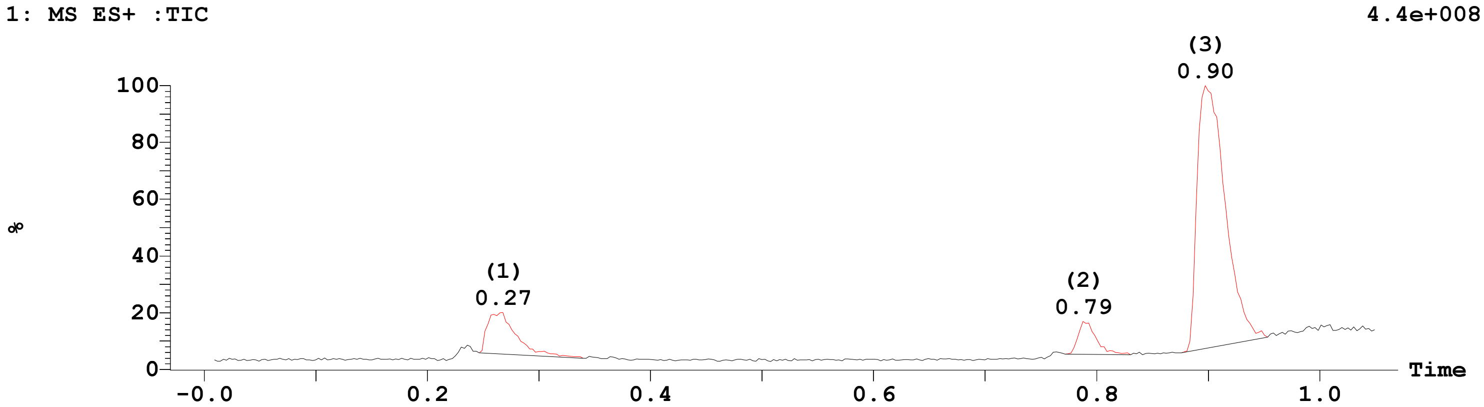

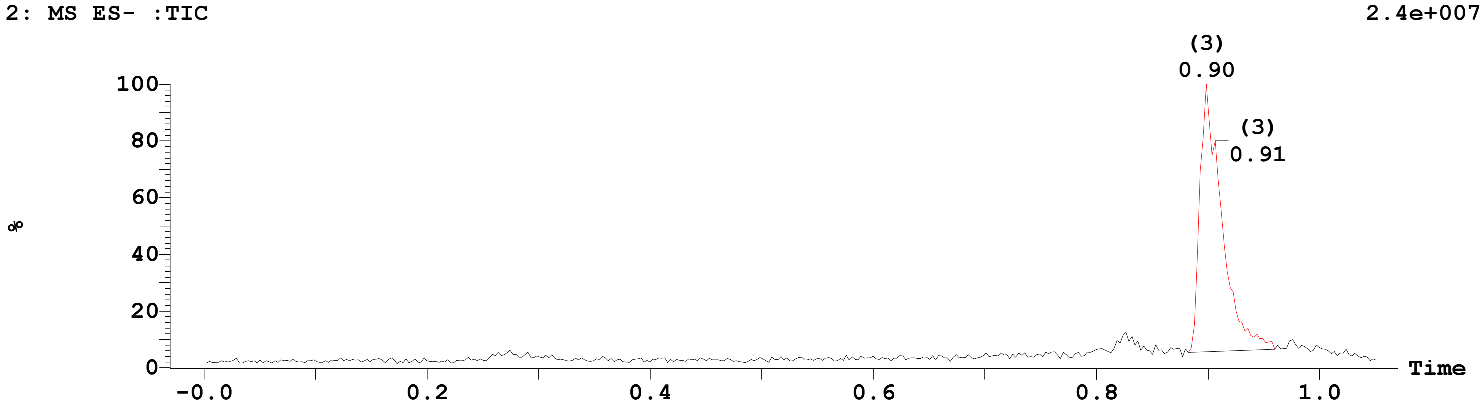

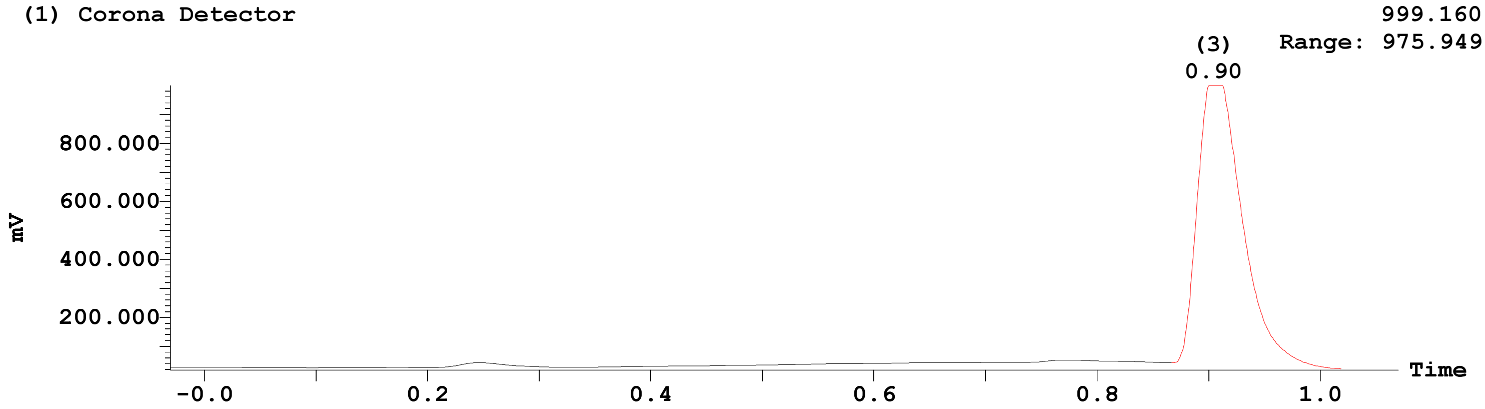

File:13zp872l1 Vial:5:49 ID:G10
Method:C:\MASSLYNX\1minLC_MS.olp

Peak ID	Time
1	0.27

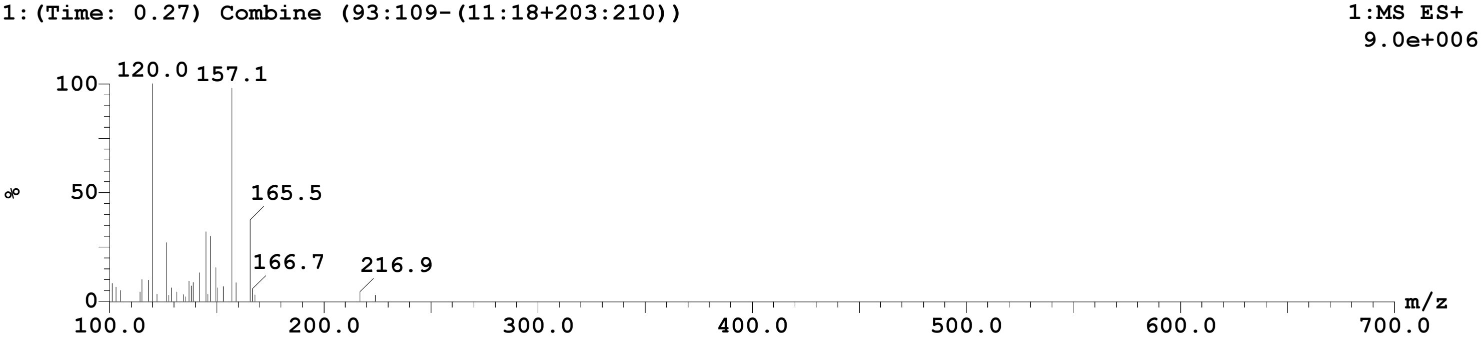

Peak ID	Time
2	0.79

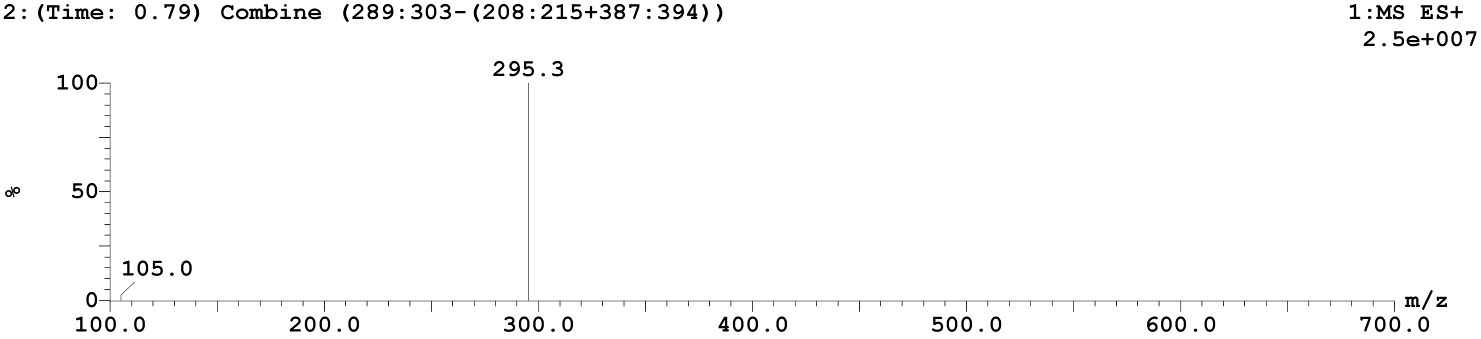

Peak ID	Time
3	0.90

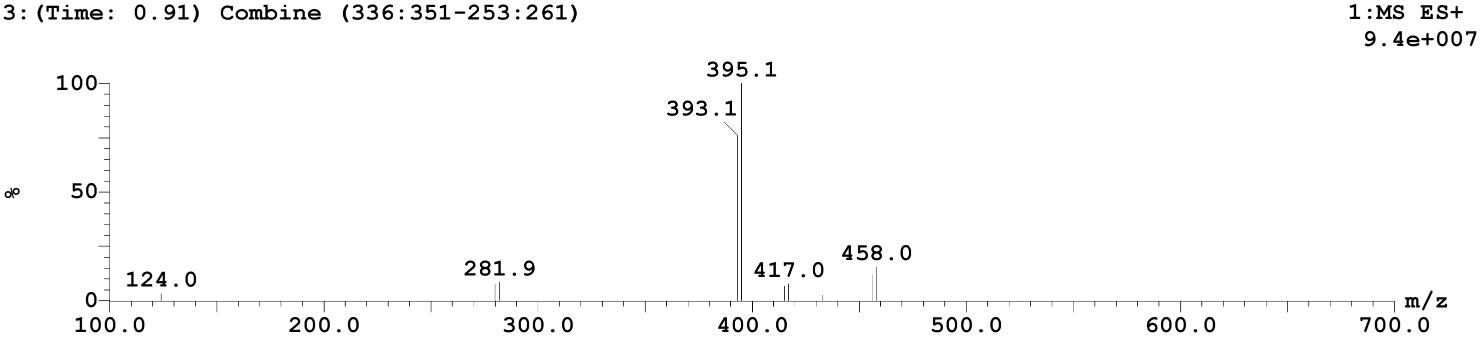

Peak ID	Time
3	0.90

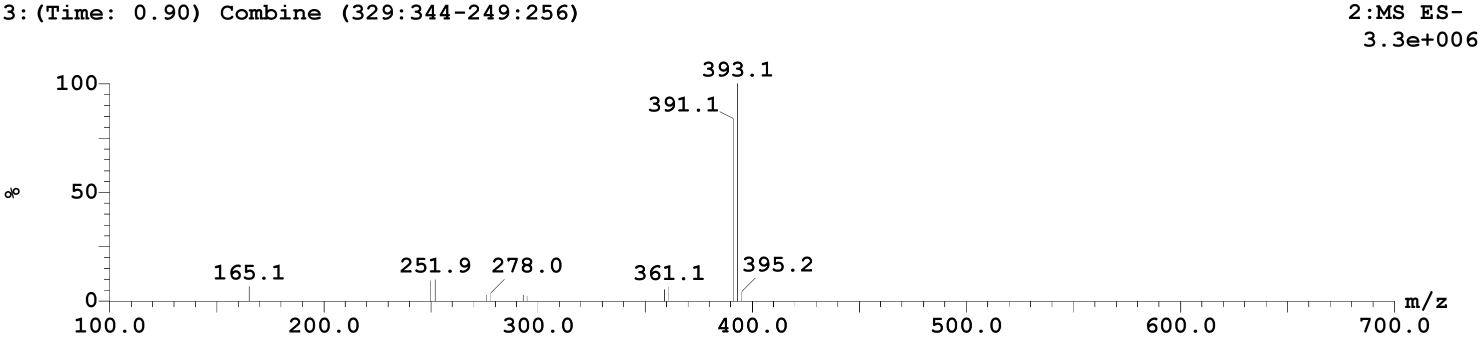

8. Fungicide, Herbicide and Insecticide Assay

Scores are given for numbered replicates, and average scores for the replicates (**avg**); Scores are
99- any herbicide effect (Herbicide assays); >70% mortality (Insecticide assays); complete control of pathogen in that replicate (Fungicide assays)
55- Partial control of pathogen (Fungicide assays only)
0- no activity
NC- data not captured for this replicate (test failure)

			Fungicide Assays																					Herbicide Assays						Insecticide Assays															
			Phytophthora infestans (tomato)			*Zymoseptoria tritici* (wheat)				*Uromyces viciae-fabae* (bean)			*Pythium dissimile*			*Alternaria solani*			*Botryotinia fuckeliana*			*Gibberella zeae*			*Arabidopsis thaliana*			*Poa annua*			Aphid species				*Heliothis virescens*				*Plutella xylostella*				*Diabrotica balteata*			
			Disease control			Disease control				Disease control			Disease control			Disease control			Disease control			Disease control			Herbicide effect			Herbicide effect			Mixed population				Egg/Larval mortality				Larval mortality				Larval mortality			
Compound ID	Smile	Rate	Rep 1	Rep 2	Avg	Rep 1	Rep 2	Rep 3	Avg	Rep 1	Rep 2	Avg	Rep 1	Rep 2	Avg	Rep 1	Rep 2	Avg	Rep 1	Rep 2	Avg	Rep 1	Rep 2	Avg	Rep 1	Rep 2	Avg	Rep 1	Rep 2	Avg	Rep 1	Rep 2	Rep 3	Avg	Rep 1	Rep 2	Rep 3	Avg	Rep 1	Rep 2	Rep 3	Avg	Rep 1	Rep 2	Rep 3	Avg
D10	Nc1ccc(cc1)S(=O	2High	0	0	0	0	0	0	0	0	0	0	55	55	55	0	0	0	0	0	0	0	0	0	0	0	0	0	0	0	0	0	0	0	0	0	0	0					0	0	0	0
D6	Cc1c(c(C)cc(c1)C	2High	0	0	0	0	0	55	18	0	0	0	0	0	0	0	0	0	0	0	0	0	0	0	0	0	0	0	0	0	0	0	0	0	0	0	0	0					0	0	0	0
D7	Nc1ccc(S(Nc2cccc	2High	0	0	0	0	0	0	0	0	0	0	99	55	77	0	0	0	0	0	0	0	0	0	0	0	0	0	0	0	0	0	0	0	0	0	0	0					0	0	0	0
D5	Nc1ccc(S(=O)(=C	2High	0	0	0	0	0	0	0	0	0	0	0	0	0	0	0	0	0	0	0	0	0	0	0	0	0	0	0	0	0	0	0	0	0	0	0	0					0	0	0	0
D1	Nc1ccc(cc1)S(=O	2High	99	0	49	0	55	0	18	0	0	0	0	0	0	0	0	0	0	0	0	0	0	0	0	0	0	0	0	0	0	0	0	0	0	0	0	0					0	0	0	0
D3	O=C(CNS(=O)(=(	2High	0	0	0	0	0	0	0	0	0	0	0	0	0	0	0	0	0	0	0	0	0	0	0	0	0	0	0	0	0	0	0	0	0	0	0	0					0	0	0	0
D2	O=C(N1CCN(CC1	2High	0	0	0	99	0	99	66	0	0	0	0	0	0	0	0	0	0	0	0	0	0	0	0	0	0	0	0	0	0	0	0	0	0	0	0	0					0	0	0	0
D8	O=S(c1ccc(N)cc1	2High	0	0	0	0	0	0	0	0	0	0	0	0	0	0	0	0	0	0	0	0	0	0	0	0	0	99	99	99	0	0	0	0	0	0	0	0					0	0	0	0
E3	O=C(CN(S(c1c[nH	2High	0	0	0	0	0	0	0	0	0	0	0	0	0	0	0	0	0	0	0	0	0	0	0	0	0	0	0	0	0	0	0	0	0	99	0	33					0	0	0	0
A2	O=S(=O)(c1c(OC	2High	0	0	0	0	0	0	0	0	0	0	0	0	0	0	0	0	0	0	0	0	0	0	0	0	0	0	0	0	0	0	0	0	0	0	0	0					0	0	0	0
A3	CN(S(c1c(nc(OC)(	2High	0	0	0	0	0	0	0	0	0	0	0	0	0	0	0	0	0	0	0	0	0	0	0	0	0	0	0	0	0	0	0	0	0	0	0	0					0	0	0	0
A4	O=S(c1c(nc(OC)r	2High	0	0	0	0	55	99	51	0	0	0	0	0	0	0	0	0	0	0	0	0	0	0	0	0	0	0	0	0	0	0	0	0	NT	NT	NT	NA	0	0	0	0	0	0	0	0
A5	COc1nc(OC)c(S(N	2High	0	0	0	0	0	0	0	0	0	0	0	0	0	0	0	0	0	0	0	0	0	0	0	0	0	0	0	0	0	0	0	0	0	0	0	0					0	0	0	0
A6	O=S(Nc1c(C)cc(c	2High	0	0	0	0	0	0	0	0	0	0	0	0	0	0	0	0	0	0	0	0	0	0	0	0	0	0	0	0	0	0	0	0	0	0	0	0					0	0	0	0
A7	FC(F)(F)c1ccc(cc1	2High	0	0	0	0	0	0	0	0	0	0	0	0	0	0	0	0	0	0	0	0	0	0	0	0	0	0	0	0	0	0	0	0	0	0	99	33					0	0	0	0
A8	COc1c(cnc(OC)n1	2High	0	0	0	0	0	0	0	0	0	0	0	0	0	0	0	0	0	0	0	0	0	0	0	0	0	0	0	0	0	0	0	0	0	0	0	0					0	0	0	0
A9	CC(NS(c1cnc(OC)	2High	0	0	0	0	0	0	0	0	0	0	0	0	0	0	0	0	0	0	0	0	0	0	0	0	0	0	0	0	0	0	0	0	0	0	99	33					0	99	0	33
B2	O=S(N1CCN(C(=(	2High	0	0	0	0	99	0	33	0	0	0	0	0	0	0	0	0	0	0	0	0	0	0	0	0	0	0	0	0	0	0	0	0	0	0	0	0					0	0	0	0
B4	CCS(N1C2CCC1C(	2High	0	0	0	0	0	0	0	0	0	0	0	0	0	0	0	0	0	0	0	0	0	0	0	0	0	0	0	0	0	0	0	0	99	0	0	33					99	0	0	33
B5	CCS(=O)(=O)NC(	2High	0	0	0	0	0	0	0	0	0	0	0	0	0	0	0	0	0	0	0	0	0	0	0	0	0	0	0	0	0	0	0	0	0	0	0	0					0	0	0	0
B6	CCS(=O)(=O)Nc1	2High	0	0	0	0	0	0	0	0	0	0	0	0	0	0	0	0	0	0	0	0	0	0	0	0	0	0	0	0	0	0	0	0	0	0	0	0					0	0	0	0
B7	CCS(Nc1cc(C(F)(F	2High	0	0	0	0	0	55	18	0	0	0	0	0	0	0	0	0	0	0	0	0	0	0	0	0	0	0	0	0	0	0	0	0	99	0	0	33					0	0	0	0
B8	O=S(=O)(CC)NC1	2High	0	0	0	0	55	0	18	0	0	0	0	0	0	0	0	0	0	0	0	0	0	0	0	0	0	0	0	0	0	0	0	0	0	0	0	0					0	0	0	0
B9	CC(c1ccccc1)NS((	2High	0	0	0	0	0	0	0	0	0	0	0	0	0	0	0	0	0	0	0	0	0	0	0	0	0	0	0	0	0	0	0	0	0	0	99	33					0	0	0	0
C1	O=S(c1ccc(cc1)O	2High	0	0	0	0	0	0	0	0	0	0	0	0	0	0	0	0	0	0	0	0	0	0	0	0	0	0	NC	0	0	0	0	0	0	0	0	0					NC	0	0	0
C2	O=C(N1CCN(CC1	2High	0	0	0	0	0	99	33	0	0	0	0	0	0	0	0	0	0	0	0	0	0	0	0	0	0	0	0	0	0	0	0	0	0	0	0	0					0	0	0	0
C3	CCOC(=O)CN(S(c	2High	0	0	0	55	0	0	18	0	0	0	0	0	0	0	0	0	0	0	0	0	0	0	0	0	0	0	0	0	0	0	0	0	0	0	0	0					0	0	0	0
C4	N#CC1CC2N(C(C	2High	0	0	0	0	0	0	0	0	0	0	0	0	0	0	0	0	0	0	0	0	0	0	0	0	0	0	0	0	0	0	0	0	0	0	0	0					0	0	0	0
C5	O=S(=O)(NCc1cc	2High	55	0	27	0	0	0	0	0	0	0	0	0	0	0	0	0	0	0	0	0	0	0	0	0	0	0	0	0	0	0	0	0	99	0	0	33					0	0	0	0
C6	Cc1cc(cc(C)c1NS(	2High	0	0	0	0	55	0	18	0	0	0	0	0	0	0	0	0	0	0	0	0	0	0	0	0	0	0	0	0	0	0	0	0	0	0	99	33					0	0	0	0
C7	O=S(c1ccc(cc1)O	2High	0	0	0	0	NC	0	0	0	0	0	99	55	77	0	0	0	0	0	0	0	0	0	0	0	0	0	0	0	0	0	0	0	0	0	0	0					0	0	0	0
C8	O=S(c1ccc(cc1)O	2High	0	0	0	0	0	0	0	0	99	49	0	0	0	0	0	0	0	0	0	0	0	0	0	0	0	0	0	0	0	0	0	0	0	0	0	0					0	0	0	0
C9	CC(NS(c1ccc(cc1)	2High	0	0	0	0	0	0	0	0	0	0	55	55	55	0	0	0	0	0	0	0	0	0	0	0	0	0	0	0	0	0	0	0	0	0	0	0					0	0	0	0
C10	O=S(NCC1CCCCC	2High	99	0	49	0	0	0	0	0	0	0	55	55	55	0	0	0	0	0	0	0	0	0	0	0	0	0	0	0	0	0	0	0	0	0	0	0					0	0	0	0
D4	Nc1ccc(S(=O)(=C	2High	0	0	0	0	0	0	0	0	0	0	0	0	0	0	0	0	0	0	0	0	0	0	0	0	0	0	0	0	0	0	0	0	0	0	0	0					0	0	0	0
D9	Nc1ccc(cc1)S(=O	2High	0	0	0	0	0	0	0	0	0	0	0	0	0	0	0	0	0	0	0	0	0	0	0	0	0	0	0	0	0	0	0	0	0	0	0	0					0	0	0	0
E2	O=S(N1CCN(C(C)	2High	0	0	0	0	0	0	0	0	0	0	0	0	0	0	55	27	0	0	0	0	0	0	0	0	0	0	0	0	0	0	0	0	0	0	0	0					0	0	0	0
E4	N#CC1CC2N(C(C	2High	0	NC	0	0	0	0	0	0	0	0	0	0	0	0	0	0	0	0	0	0	0	0	0	0	0	0	0	0	0	0	0	0	0	0	0	0					99	0	0	33
E5	O=S(=O)(NCc1cc	2High	0	0	0	0	0	0	0	0	0	0	0	0	0	0	0	0	0	0	0	0	0	0	0	0	0	0	0	0	0	0	0	0	0	0	0	0					0	0	0	0
E6	Cc1cc(C)c(c(C)c1	2High	0	0	0	0	0	99	33	0	0	0	0	0	0	0	0	0	0	0	0	0	0	0	0	0	0	0	0	0	0	0	0	0	0	0	99	33					0	0	0	0
E7	O=S(=O)(Nc1ccc	2High	0	0	0	0	0	0	0	0	0	0	0	0	0	0	0	0	0	0	0	0	0	0	0	0	0	0	0	0	0	0	0	0	99	0	0	33					0	0	0	0
E8	O=S(NC1CC1)(=(	2High	0	0	0	0	0	55	18	0	0	0	0	0	0	0	0	0	0	0	0	0	0	0	0	0	0	0	0	0	0	0	0	0	99	0	0	33					0	0	0	0
E9	CC(c1ccccc1)NS(=	2High	0	0	0	0	0	0	0	0	0	0	0	0	0	0	0	0	0	0	0	0	0	0	0	0	0	0	0	0	0	0	0	0	0	0	0	0					0	0	0	0
E10	O=S(c1cc[nH]c1)	2High	0	0	0	0	0	0	0	0	0	0	0	0	0	0	0	0	0	0	0	0	0	0	0	0	0	0	0	0	0	0	0	0	0	0	0	0					0	0	0	0
F3	O=C(OCC)CN(C)S	2High	0	0	0	0	0	0	0	0	0	0	0	0	0	0	0	0	0	0	0	0	0	0	0	0	0	0	0	0	0	0	0	0	99	0	0	33					0	0	0	0
F4	N#CC1CC2N(S(=	2High	0	0	0	99	0	0	33	0	0	0	0	0	0	0	0	0	0	0	0	0	0	0	0	0	0	0	0	0	0	0	0	0	0	0	0	0					0	0	0	0
F5	O=C(OCCCS(=O)	2High	0	0	0	0	0	0	0	0	0	0	0	0	0	NC	0	0	0	0	0	0	0	0	0	0	0	0	0	0	0	0	0	0	0	0	0	0					0	0	0	0
F6	Cc1cc(C)cc(C)c1N	2High	0	0	0	0	0	0	0	0	0	0	0	0	0	0	0	0	0	0	0	0	0	0	0	99	49	0	0	0	0	0	0	0	0	0	0	0					0	0	0	0
F8	O=S(=O)(NC1CC	2High	0	0	0	0	0	0	0	0	0	0	0	0	0	0	0	0	0	0	0	0	0	0	0	0	0	0	0	0	0	0	0	0	0	0	0	0					0	0	0	0
F9	CC(c1ccccc1)NS((	2High	0	0	0	55	0	0	18	0	0	0	0	0	0	0	0	0	0	0	0	0	0	0	0	0	0	0	0	0	0	0	0	0	0	0	0	0					0	0	0	0
F10	O=C(C)OCCCS(N(	2High	0	0	0	0	0	55	18	0	0	0	0	0	0	0	0	0	0	0	0	0	0	0	0	0	0	0	0	0	0	0	0	0	0	0	0	0					0	0	0	0
G1	COc1nc(c(Br)cc1S	2High	0	0	0	0	55	0	18	0	0	0	99	99	99	0	0	0	0	0	0	0	0	0	0	0	0	0	0	0	0	0	0	0	0	0	0	0					0	0	0	0
G2	COc1nc(OC)c(cc1	2High	0	0	0	0	0	55	18	0	0	0	0	0	0	0	0	0	0	0	0	0	0	0	0	0	0	0	0	0	0	0	0	0	0	0	0	0					0	0	0	0
G3	COc1nc(OC)c(cc1	2High	0	0	0	55	0	0	18	0	0	0	0	0	0	0	0	0	0	0	0	0	0	0	0	0	0	0	0	0	0	0	0	0	0	0	0	0					0	0	0	0
G4	O=S(=O)(c1c(nc(	2High	0	0	0	0	0	0	0	0	0	0	0	0	0	0	0	0	0	0	0	0	0	0	0	0	0	0	0	0	0	0	0	0	0	0	99	33					0	0	0	0
G5	COc1c(cc(Br)c(OC	2High	0	0	0	0	0	0	0	0	0	0	0	0	0	0	0	0	0	0	0	0	0	0	0	0	0	0	0	0	0	0	0	0	0	0	0	0					0	0	0	0
G6	O=S(=O)(Nc1c(C	2High	0	0	0	0	0	0	0	0	0	0	0	0	0	0	0	0	0	0	0	0	0	0	0	0	0	0	0	0	0	0	0	0	0	0	0	0					0	0	0	0
G7	Brc1cc(c(nc1OC)(	2High	0	0	0	0	55	0	18	0	0	0	0	0	0	0	0	0	0	0	0	0	0	0	0	0	0	0	0	0	0	0	0	0	0	0	0	0					0	0	0	0
G8	COc1c(cc(S(NC2C	2High	0	0	0	0	0	0	0	0	0	0	0	0	0	0	0	0	0	0	0	0	0	0	0	99	49	0	0	0	0	0	0	0	0	0	0	0					0	0	0	0
G9	O=S(=O)(NC(c1c	2High	0	0	0	55	0	0	18	0	0	0	55	55	55	0	0	0	0	0	0	0	0	0	0	0	0	0	0	0	0	0	0	0	0	0	0	0					0	0	0	0
G10	COc1c(S(=O)(=O	2High	0	0	0	0	55	0	18	0	0	0	0	0	0	0	0	0	0	0	0	0	0	0	0	0	0	0	NC	0	0	0	0	0	0	0	0	0					0	0	0	0

Materials and Methods

<u>Methodology</u>

Fungicide assays: The compounds were evaluated in mycelial growth tests in artificial media against *Pythium dissimile, Alternaria solani, Botryotinia fuckeliana* and *Gibberella zeae*, at rates of 20ppm.

Test species	Media	Rate (ppm)
Pythium dissimile	Semi-solid	20
Alternaria solani	Semi-solid	20
Botryotinia fuckeliana (*Botrytis cinerea)*	Semi-solid	20
Gibberella zeae (*Fusarium graminearum*)	Semi-solid	20

The compounds were also evaluated against several pathogens on leaf-piece assays at the rate of 100ppm for *Uromyces viciae-fabae* on bean and *Zymoseptoria tritici* on wheat, and at the rate of 200ppm for *Phytophthora infestans* on tomato. The compounds were applied prior to inoculation with the pathogens.

Test species	Host	Rate (ppm)
Zymoseptoria tritici	Wheat	100
Phytophthora infestans	Tomato	200
Uromyces viciae-fabae	Bean	100

Mycelial growth or disease inhibition was assessed visually and scored using a 3 band system (0, 55 and 99 where 99 = total inhibition of hyphal growth/disease development, 55 = partial inhibition, 0 = no inhibition), 4-14d after inoculation depending on the assay.

Herbicide plate assays:

The compounds were tested for herbicidal activity against *Arabidopsis thaliana* at 10 ppm and *Poa annua* at 32ppm. Test plates were stored for seven days in a controlled environment cabinet. They were scored as 0 or 99, where 99 = herbicidal effect, and 0 = no effect.

Test species	Treatment timing	Rate (ppm)
Arabidopsis thaliana	Pre-emergence	10
Poa annua	Pre-emergence	32

Insecticide assays: The compound was tested for activity against an aphid species and *Heliothis virescens* at 1000ppm on a leaf-piece based assay, and against *Plutella xylostella* and *Diabrotica balteata* at 500ppm in artificial diet assays. Chemicals were applied to feeding aphids, or prior to infestation with *P. xylostella, H. virescens* and *D. balteata* larvae.

Mortality was assessed relative to control wells using a 2 band system (0 or 99 where 99 = significant mortality, 0 = no effect), 3-6d after the treatments depending on the assay.

Test species	Treatment type	Media	Rate (ppm)
Aphid species	Feeding/contact	Leaf disc	1000

Plutella xylostella	Feeding/contact	Artificial diet	500
Heliothis virescens	Feeding/contact	Leaf disc	1000
Diabrotica balteata	Feeding/contact	Artificial diet	500

Note: Test 1 used Heliothis virescens as the Lepidoptera species. Test 2 used Plutella xylostella as the Lepidoptera species.

Positive controls: In addition to the test compounds, positive control compounds were included in each test: azoxystrobin and prochloraz for fungicide assays, thiamethoxam and indoxacarb for insecticide assays and norflurazon for herbicide assays.

For all screens, data were recorded for replicates and averaged.
